# Cell colony counter called CoCoNut

**DOI:** 10.1371/journal.pone.0205823

**Published:** 2018-11-07

**Authors:** Mattia Siragusa, Stefano Dall’Olio, Pil M. Fredericia, Mikael Jensen, Torsten Groesser

**Affiliations:** 1 The Hevesy Laboratory, Center for Nuclear Technologies, Technical University of Denmark, Roskilde, Denmark; 2 Department of Energy Conversion and Storage, Technical University of Denmark, Roskilde, Denmark; Chapman University, UNITED STATES

## Abstract

Clonogenic assays are powerful tools for testing cell reproductive death after biological damage caused by, for example, ionizing radiation. Traditionally, the methods require a cumbersome, slow and eye-straining manual counting of viable colonies under a microscope. To speed up the counting process and minimize those issues related to the subjective decisions of the scoring personnel, we developed a semi-automated, image-based cell colony counting setup, named CoCoNut (**Co**lony **Co**unter developed by the **Nut**ech department at the Technical University of Denmark). It consists in an ImageJ macro and a photographic 3D-printed light-box, conceived and demonstrated to work together for Crystal Violet-stained colonies. Careful attention was given to the image acquisition process, which allows background removal (*i*.*e*. any unwanted element in the picture) in a minimally invasive manner. This is mainly achieved by optimal lighting conditions in the light-box and dividing the image of a flask that contains viable colonies by the picture of an empty flask. In this way, CoCoNut avoids using aggressive background removal filters that usually lead to suboptimal colony count recovery. The full method was tested with V79 and HeLa cell survival samples. Results were compared to other freely available tools. CoCoNut proved able to successfully distinguish between single and merged colonies and to identify colonies bordering on flask edges. CoCoNut software calibration is fast; it requires the adjustment of a single parameter that is the smallest colony area to be counted. The employment of a single parameter reduces the risk of subjectivity, providing a robust and user-friendly tool, whose results can be easily compared over time and among different bio-laboratories. The method is inexpensive and easy to obtain. Among its advantages, we highlight the possibility of combining the macro with a perfectly reproducible 3D-printed light-box. The CoCoNut software and the 3D-printer files are provided as supporting information (S1 CoCoNut Files).

## Introduction

The clonogenic assay is a well-established method for measuring cell response to damaging agents, for example ionizing radiation. The response is a relationship between the quantity of the agent (the absorbed dose) and the fraction of cells that retains the ability to reproduce and form colonies within a given amount of time. Traditionally, colonies with more than 50 cells are identified as viable and then counted [1]. The 50-cell threshold is of course arbitrary, but its adoption normally makes viable colonies conveniently detectable to the naked eye, depending on the cell line. To achieve statistical accuracy, it is always necessary to work with a large number of samples, which turns the colony counting process into a time-consuming and eye-straining task. Fast, accurate and automated counting procedures with reproducible and objective outputs are needed. In this paper, we present a new Colony Counter, developed by the Center for Nuclear Technologies (Nutech) at the Technical University of Denmark (DTU). The new tool is named CoCoNut (**Co**lony **Co**unter developed by the **Nut**ech department at DTU).

On one hand, computer vision methods can overcome some of the important limitations of manual procedures: weariness of the counting personnel and the subjectivity of the scoring decisions. At the same time, automated procedures give access to parameters like the colony area and its perimeter that can describe interesting morphological characteristics of the colonies. On the other hand, methods that rely on image analysis have to deal with several issues, for example:

1. Pictures acquisition under uniform lighting conditions to avoid shadows and reflections, which could produce misleading results.
2. Accurate background identification and removal.
3. Separation of merging colonies in order to avoid underestimation of colony counts.
4. Correction for the non-uniformity of the colonies for edges and sidewalls of cell culture containers.
5. Development of a fast calibration procedure.
6. Development of user-friendly and cost-effective methods.
7. Identification of fuzzy colonies.
8. Variation in size and abnormal shape of tumor cells.

In this paper, we address all these points, creating a tool that does not try to compete with commercially available systems but that is congenial with small laboratories with a limited budget. CoCoNut has been tested using the V79 and HeLa cell lines. CoCoNut should be used exclusively for blue/purple-stained colonies (*e*.*g*. Crystal Violet).

Several image-based procedures have been developed over the years, but no single method has achieved a widespread use. To date, no tool has succeeded in solving all the problems listed above.

Some counters perform only in a proper manner when cell colonies are well spaced, distant from the container edges, and show a good contrast with the background. Other counters work only adequately after long calibration procedures, which require the adjustment of several parameters and, therefore, may trespass the subjectivity line. Yet others are not user-friendly, which means they do not give direct access to the code or require advanced programming skills. Finally, commercially available tools can be rather expensive.

Expensive hardware for image acquisition is not always congenial with small laboratories. For this reason, the adoption of flatbed scanners is a common choice for many inexpensive methods [2–4]. Their adoption is however inadvisable as they are usually not located in the bio-laboratory, do not offer total uniform lighting conditions (when thick cell culture vessels are scanned), could covertly apply image filters, and cannot be used in full manual mode. For all these reasons, we do not recommend the use of flatbed scanners. Instead, we entrust CoCoNut with the task of providing a novel and cost-effective alternative: an easily reproducible 3D-printable light-box coupled with a digital camera.

Examples of computer-aided colony counters developed between 1985 and 2008 are [2,5–14], which are briefly described by [4,15,16].

In 2011 Cai *et al*. published a simple ImageJ-based code, dedicated to the clonogenic assay [17]. With that publication, they brought in the field of computer vision based colony counters the idea of exploiting the many merits of ImageJ, moving towards one of the most user-friendly and inspired platforms for scientific image analysis. Cai *et al*. wrote an ImageJ macro (here called CAI) whose worthiness is its simplicity. With ImageJ built-in functions, they succeeded in creating a limited but serviceable tool. Unfortunately, they did not make the whole macro available, but they published only a few lines of code that can be used to recreate their tool. More recently, Guzmán *et al*. presented an ImageJ plugin called ColonyArea [18]. It automatically identifies the percentage of the surface of n-well plates that is covered by viable colonies, but without providing indications on the total number of the cell clones. In fact, to avoid issues related to cell growth abnormalities on the edges of the wells, ColonyArea discards the outermost part of the wells, reducing their diameter by 5% from the edges. As cell colonies tend to show some affinity for the plastic edges, this method cannot grant information on the number of colonies contained in the wells. ColonyArea implements the use of higher order spatial derivatives to separate the background (like empty wells and imaging artefacts) from the foreground. In 2016, Choudhry [16] published another ImageJ plugin, called Colony Edge. The aim of the work was to introduce a novel tool for counting cells and colonies in high-throughput screens with colony forming and cellular assays. Similarly to CAI, but in a more complex way, Colony Edge exploits many ImageJ built-in functions. The drawback is that, for clonogenic assay purposes, this tool requires the adjustment of many parameters before the first run. In this article, we compared our macro with CAI using V79 and HeLa samples. Other software programs used for comparison are OpenCFU [19] and AutoCellSeg [20], which are not ImageJ-based tools. OpenCFU is a completely open source lightweight application designed to enumerate clustered circular objects. AutoCellSeg is a very recently published automatic colony forming unit analyzer. Both OpenCFU and AutoCellSeg have been suggested for use on cell-based images by their respective authors.

CoCoNut is a novel method for fast and semi-automated cell colony scoring, which is applicable to all types of cell culture containers. It has been tested using both well-spaced and merging colonies. It is based on ImageJ and structured as a macro, thus it provides direct access to the code. It is pegged to a low-cost image acquisition system, which is perfectly reproducible and provides uniform lighting conditions. Such system can be 3D-printed (locally or through web services) and used in combination with any standard digital camera. The software calibration of CoCoNut requires only the adjustment of a single parameter, namely the radius of the smallest colony to be counted. The employment of a single parameter minimizes the risk of subjectivity, providing a consistent tool, whose results can be easily compared over time and among different bio-laboratories.

The CoCoNut software and the 3D-printer files are provided as supporting information (S1 CoCoNut Files).

## Materials and methods

### Cell culture of V79 and HeLa cells

Chinese hamster V79 lung fibroblasts were a generous gift from Dr. Priscilla Cooper. HeLa Cells (human cervical cancer cell line, ECACC 93021013) were obtained from ECACC, Salisbury, UK through Sigma-Aldrich (St Louis, MO). Both cell lines were cultured as adherent cell monolayers under atmospheric air and 95% humidity adding 5% CO_2_ in Dulbecco’s Modified Eagles Medium (DMEM) (Sigma-Aldrich, Brøndby, Denmark). DMEM medium was supplemented with 10% Fetal Bovine Serum (FBS; Sigma-Aldrich), 4 mM L-Glutamine (Sigma-Aldrich), and 1% (v/v), antibiotic/antimycotic solution (working concentration: 100 U/ml penicillin, 0,1 mg/ml streptomycin, 0,25 μg/ml amphotericin B; Sigma-Aldrich). Cells were subcultured in cell culture flasks with ventilation caps after the cell monolayer was washed once with 1x Phosphate Buffered Saline (PBS) using 0.1% trypsin (Gibco/ThermoFisher, Waltham, MA, USA) containing 0.5 mM Ethylene Diamine Tetraacetic Acid (EDTA; Sigma-Aldrich) in 1x PBS for about 5 min at 37˚C and plated at appropriate numbers.

### Clonogenic cell survival

**Fig 1:**
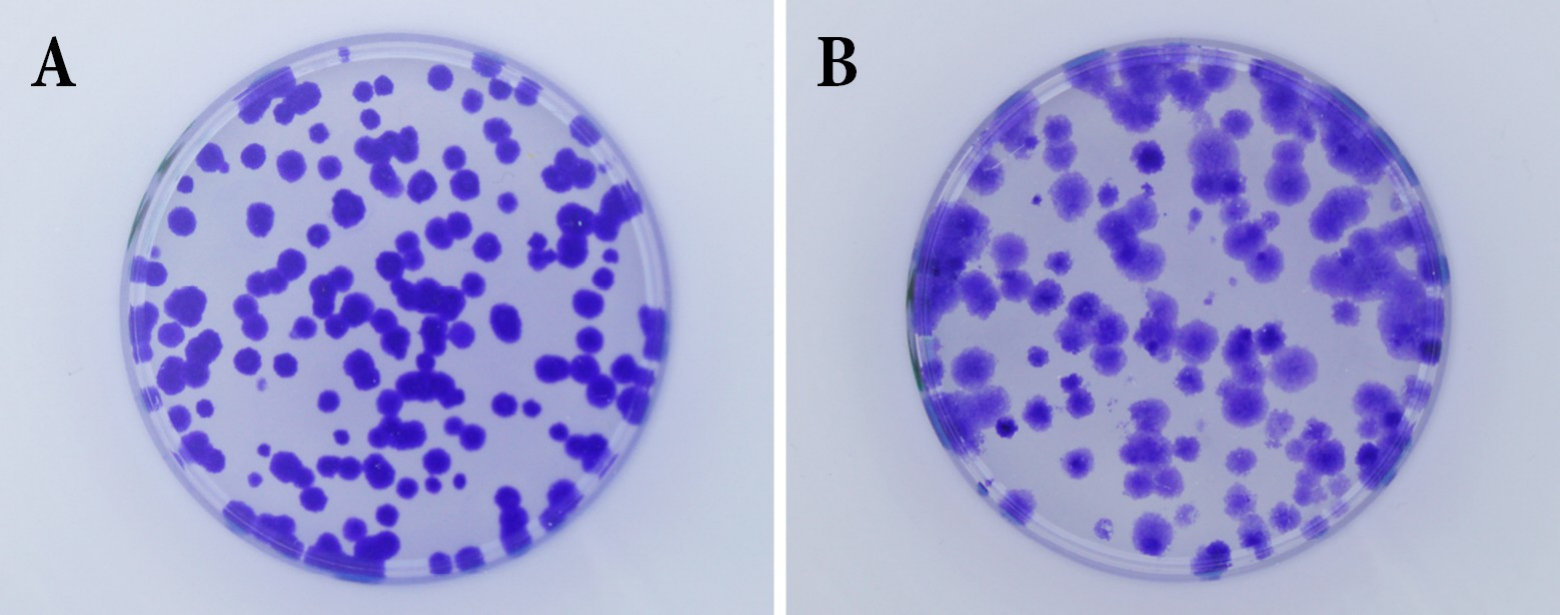
Examples of high cell density samples. High cell density samples for V79 (A) and HeLa (B) cells are shown.

Adherent cells were trypsinized and scored using the count & viability kit for the MUSE compact flow cytometer (Merck Millipore, Darmstadt, Germany). The count for viable cells was used for plaiting cells in either T25 flasks (25 cm^2^ cell culture area; Sarstedt, Skanderborg, Denmark) with ventilated caps containing 6 ml complete medium or in 35 mm cell culture dishes (Sigma-Aldrich) with 3 ml complete medium, respectively. After 7 (V79) or 14 (HeLa) days cells were washed once with 1x PBS before they were fixed and stained with 0.25% - 0.5% (w/v) Crystal Violet (concentration is depending on cell line and quality of the staining solution and should be tested) in methanol for at least 10 min. Afterwards cells were washed three times with tap water and air dried before the number of colonies was counted by eye or image acquisition was performed. Cells that could form a colony of at least 50 cells within the incubation time were considered survivors [21]. In this work, cells were not exposed to killing agents such as ionizing radiation or chemicals. Instead, untreated cells were plated in triplicates at different cell numbers, namely: 25, 70, 140, 280, 550 cells per T25 flask and 10, 25, 50, 100, and 200 per 35 mm cell culture dish for V79 cells and 50, 100, 200, 400, 800 cells per T25 flask and 25, 40, 75, 150, and 300 per 35 mm cell culture dish for HeLa cells. Cell numbers were chosen to achieve the same cell density per cm^2^ for both cell culture containers. High-density samples are shown in Fig 1.

### The ImageJ tool

ImageJ is an open source image processing software, which can be downloaded from a dedicated page (https://imagej.nih.gov/ij/download.html) of the US National Institute of Health (NIH). The main developer is Wayne Rasband from NIH (Bethesda, MD, USA). The software offers several tools for visualization, manipulation, and analysis of digital image files. In addition, it permits extensions by addition of user-written plug-ins and macros. ImageJ itself is written in Java, which makes it a platform-independent tool. The users can rely on a large and active community, which simplifies the learning process and use of the software. In addition to ImageJ itself, Fiji (“Fiji Is Just ImageJ”) is a popular project that extends ImageJ with a significant collection of existing plug-ins. CoCoNut can be executed via ImageJ (current version 1.51n) or Fiji without modifications.

### The photographic light-box

**Fig 2:**
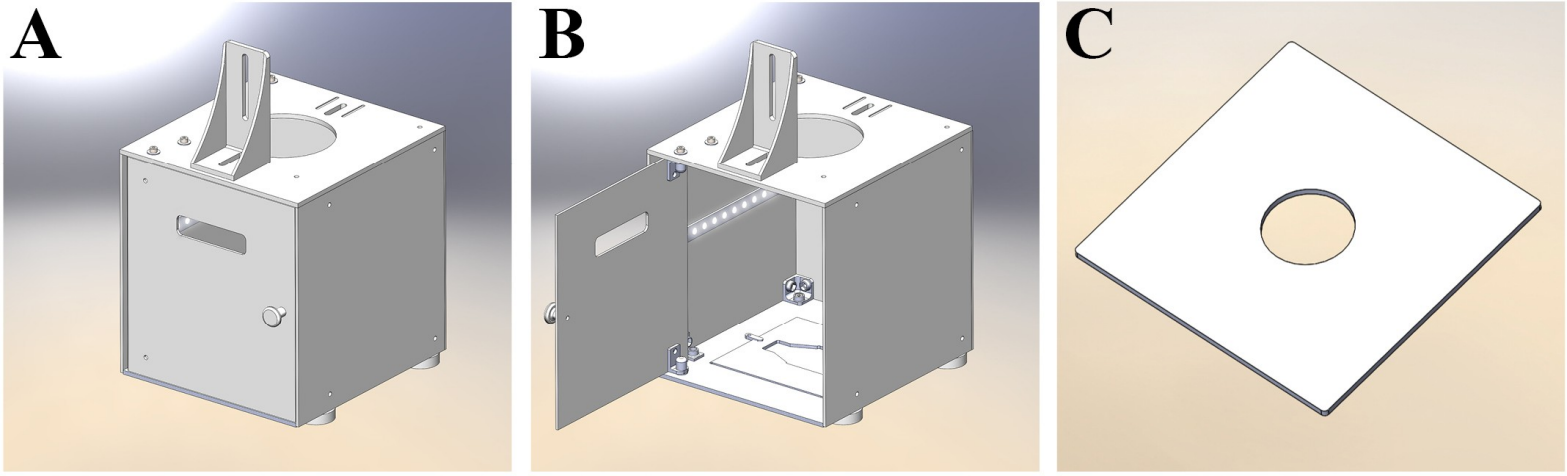
3D model of the light-box. The two picture on the left (A and B) show the three-dimensional representation model of the light-box as it appears in the commercial program SolidWorks (http://www.solidworks.it/), used to design the entire box and all its components. The LED stripe was added in post-production for clarity. The image on the right (C) shows a Petri dish holder that can be inserted in the light-box.

**Fig 3:**
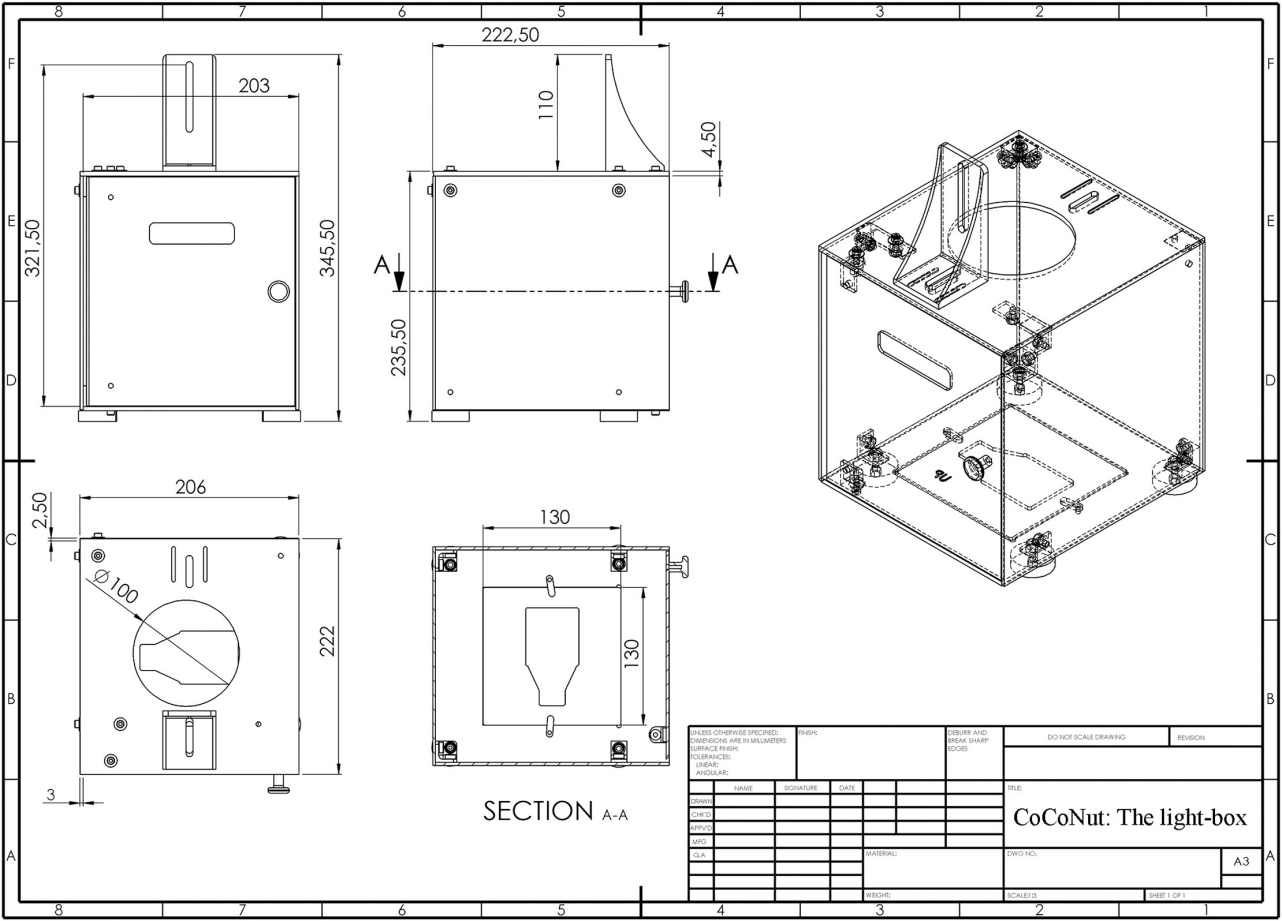
Dimensional drawing of the light-box. The box is shown at different angles. Dimensions are expressed in mm.

To apply the CoCoNut counting method with high efficiency, a light-box has been designed, aiming for a functional and compact shape, but having room to host a culture flask or dish and to provide uniform light distribution throughout the volume of the box (Fig 2). The commercial program SolidWorks (http://www.solidworks.it/) was used to create the 2- and 3-dimensional drawings shown in Fig 3. The box itself consists of 5 plastic plates assembled together with several brackets and screws, and a front door to permit easy access to the flasks during the image-acquisition step.

All components are made of Nylon. The box was 3D-printed using the web service 3D HUBS (https://www.3dhubs.com/). The 3D printing technique selected is called Selective Laser Sintering (SLS), which exploits a laser to shape and form extremely thin layers of powdered material by melting it together to create a solid structure.

The advantage of this process is that the excess of unmelted powder acts as a support to the structure as it is produced. This printing technique allows the creation of functional prototypes and end products, with high strength and stiffness. Moreover, it ensures excellent long-term functionality of the parts and detail resolution.

The outer dimensions of the box are 235 x 222 x 222.5 mm, representing the height, the length, and the width, respectively. All plastic plates have a thickness of 2.5 mm, except the top one that is 1.5 mm thicker, in order to support the camera that is installed on top of the box. Moreover, in the center of the top plate there is a circular hole with a diameter of 100 mm. It is needed to host the camera objective, used to photograph the samples that are contained in the box. To hold up the camera, a customized support has been added on top of the box. It allows vertical movements of the camera and can sustain cameras of different size without modifying the box.

In the upper part of the door, a loophole has been designed, so that the operator can check the correct positioning of the jig and the flask at any time. The correct positioning of the flask in the box is of fundamental importance for achieving accurate results. An embossment has been created in the bottom plate to hold the jig in place.

To light the cells, a white LED (Light Emitting Diode) stripe (ZFS-8500-CW/SEC, JKL Components, Pacoima, CA, USA) has been installed horizontally approximately around the middle height of the box; it covers all three sides of the box excluding the door. A 12V DC power supply provides power to the led, and the total power consumption is 2.5 W.

#### Lighting condition measurement of the light-box

Uniform light condition is a typical requirement for image-based automated cell colony counters. However, estimates of uniformity for cell colony counting methods that make use of common flatbed scanners are usually made by eye. In the here presented work, we provide quantitative information on light exposure conditions inside the CoCoNut light-box. For this purpose, we employed the X-Rite ColorChecker White Balance target (passport edition; Grand Rapids, Michigan, USA), which is a scientifically engineered, spectrally neutral white reference (*i*.*e*. it does not cause color shift under different light conditions). The ColorChecker consists of an 18% gray surface (that is half-way between black and white), designed to provide the same response in each of the RGB channels. After placing the X-Rite target into the light-box, a photograph of the ColorChecker was taken. Analysis of dark areas in the images was carried out through the Interactive 3D Surface Plot plugin (https://imagej.nih.gov/ij/plugins/surface-plot-3d.html), which creates surface plots from given images. This tool interprets the luminance of an image as the height of the plot, *i*.*e*. the plotted surface bends when luminance changes. The White Balance target is 8.8 cm long and 5.7 cm wide, while the cell culture flasks used in our work are slightly smaller.

### Picture acquisition of cell clones

Pictures acquisition was performed using the homemade 3D-printed photo-box and an EOS 500D digital camera (Canon, Amstelveen, The Netherlands). The camera was coupled to a Canon EF 18–55 mm f/3,5–5,6 IS lens, the lens hood (Canon EW-60C) was connected to the camera. The photographic lens was set at the focal length of 48 mm as for the light uniformity test described before, the motion stabilizer was deactivated, and the auto focus was disabled after a single, first focus adjustment. Cell culture flasks or dishes were placed in the light-box with the help of a plastic jig (Fig 2), and covered with a commercial anti-glare film (HD Anti-Glare Screen Protector Film, Power Support International, Seattle, USA), which removed reflections (of the camera lens) and glare. Pictures were taken as RGB .jpg images, using an external remote control (YP-880/E3, SHENZHEN NEEWER TECHNOLOGY Co., Shenzhen, China) connected to the camera and set with a 2-second timer delay to prevent camera shaking. Any letterings on the plastic vessels was made in green.

### Description of the code

**Fig 4:**
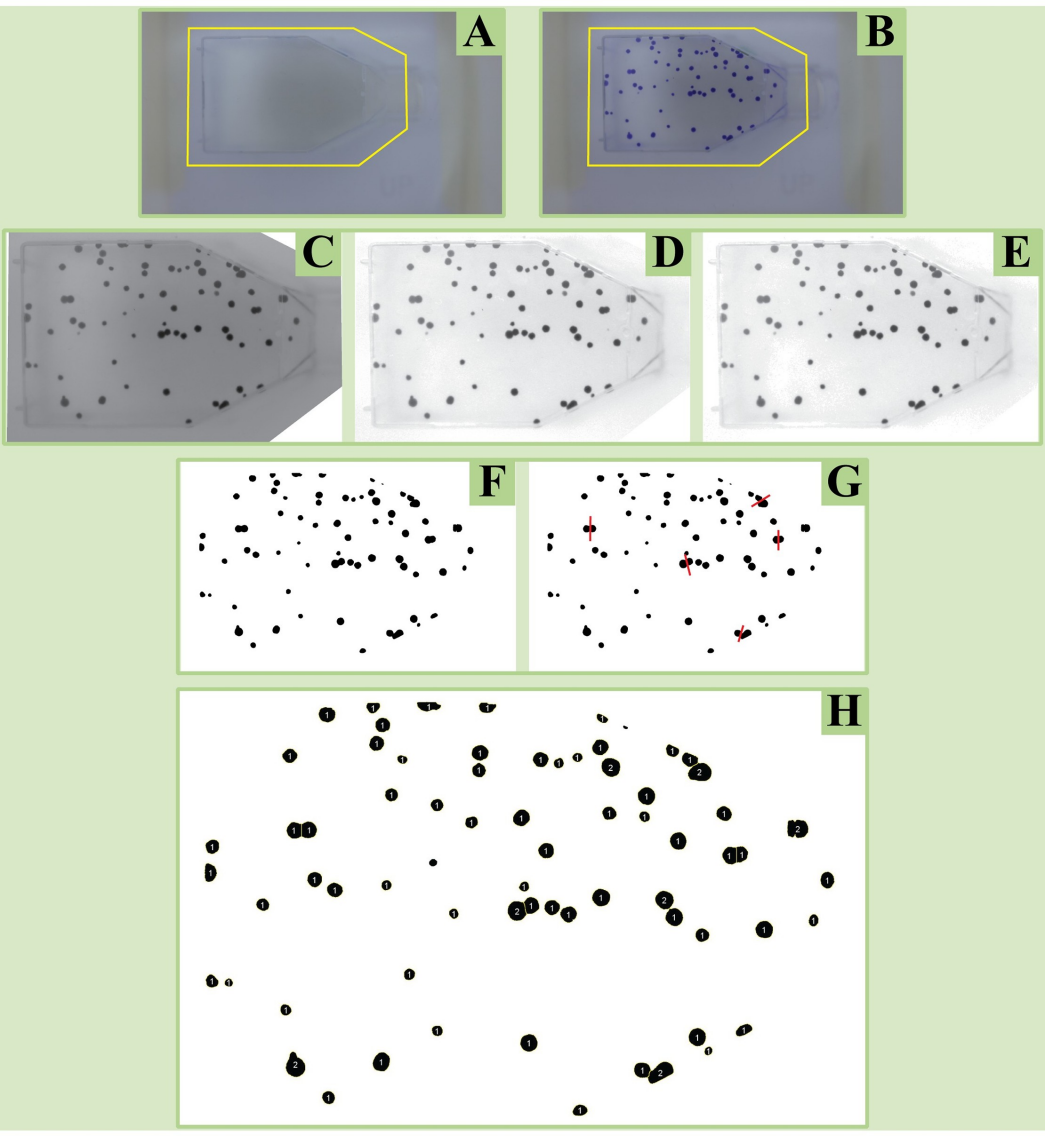
CoCoNut image analysis description. Cell clones are scored in CoCoNut following the steps shown in this figure. In panel A, a Region Of Interest (ROI) is selected using, as an example, the empty flask. In B, the same ROI is applied on the flask containing the cell clones and is kept constant during the entire experiment. In C, the green channel of the RGB image is preserved, while R and B are discarded. In D, the image of the sample is divided by the image of the empty flask. In E, background is reduced by discarding the very right tail of the histogram (as dark colonies are contained in the left tail). The “opening” morphological filter is also applied. In F, a binary picture is created using thresholding. In G, the watershed algorithm separates merging colonies that show a smooth convex shape. Finally, in H cell clones are scored.

In CoCoNut images are analyzed as shown in Fig 4. Pictures area taken in the Adobe RGB color space and flasks (or dishes) are selected identifying a Region Of Interest (ROI) that is larger than the side of the flask containing the cell clones. The same ROI is used for the entire experiment (Fig 4, panels A and B). Subsequently, RGB images are split to their three separate color components, discarding the Red and the Blue channels, but preserving the Green one (Fig 4, panel C). This reflects the fact that cell clones are stained using the Crystal Violet dye. Because the line of purples hue in the CIE chromaticity diagram is located in diametrically opposite position of the green colors, it is reasonable to expect that the signal for the cell clones is darker (in greater contrast to background) in G than in R or B. Since 8 bits are used to represent G, its value can only range between 0 and 255, which ideally assigns 255 to the bright background and 0 (absence of green) to the colonies.

Due to the affinity of cell colonies for cell culture dish, plate and flask edges, one of the biggest issues of image-based counters is the digital separation of cell clones from plastic. It has been shown that algorithms based on gray-level image segmentation techniques (thresholding) alone cannot properly identify the edges [11]. Moreover, an extensive employment of background removal filters may lead to detrimental operations, producing artifacts and changing the morphology of the cell colonies. Since no digital filter has been created to remove completely the background while sparing the structure of the cell clones, it is necessary to put efforts in the image acquisition process. With this in mind, Barber *et al*. [11] partially solved the edge problem by dividing the image of a sample by the image of an empty flask. In CoCoNut the division process (Fig 4, panel D) is performed after normalization of the gray-level scale from 0–255 to 0–1. After normalization, 255 becomes 1, but 0 remains at 0. In this way, after the division of a flask containing cells by an empty one, similar objects, namely the structure of the flask itself, turn white, while the cell clones stay black. The two requirements for conducting a successful division process are the accurate positioning of the flasks, which must be placed every time at the exactly same location, and their exchangeability, in the sense that they should be identical in shape and size. Despite the fact that we have no control on the quality of the cell culture containers, which are usually not perfectly interchangeable, we can provide accurate positioning by using plastic jigs (Fig 2B and 2C). Our division method largely, but not entirely, removes the background. Significant scoring improvements have been accomplished by using a stained empty cell culture container for background subtraction. This step prevented residual stain in the corners and along the edges of the flasks to be picked up as false colonies.

To accentuate the difference between the bright background and the dark colonies we adjust the tonal range, namely we increase the intensity of the image, for example setting 0.95 (instead of 1) as pure white. The loss of detail in the lightest area of the pictures should be considered as a form of background reduction, which does not affect the cell clones that are seen as dark spots in the image. In other words, the background is reduced (Fig 4, panel E) by discarding the very right tail of the histogram (as dark colonies are contained in the left tail).

Further background removal and noise reduction is given by the use of morphological filters, which can now be used in a minimally invasive manner to remove small structures from the image without altering the morphological properties of the cell clones. Morphological filters are applied calculating the dilation of the erosion of an image (Panel E. This process is conveniently called opening) through the same structuring element, which specifies the properties of the filters. The fundamental result of the opening is that all elements, including digital noise, that are smaller than the structuring element itself are discarded via erosion. In addition, cell colonies are smoothed by erosion, but almost preserve their size thanks to successive dilation. In CoCoNut the structuring element is defined by the radius, in pixel, of the smallest viable colony. This value is defined by the experimenter and represents the only needed input.

Next, CoCoNut creates a binary picture using thresholding (Fig 4, panel F), which is a common technique to isolate the foreground (the cells) of an image from its background (the flask). The threshold is automatically calculated using ImageJ default technique, which is an application of the IsoData method described by Ridler et al. in 1978 [22].

**Fig 5:**
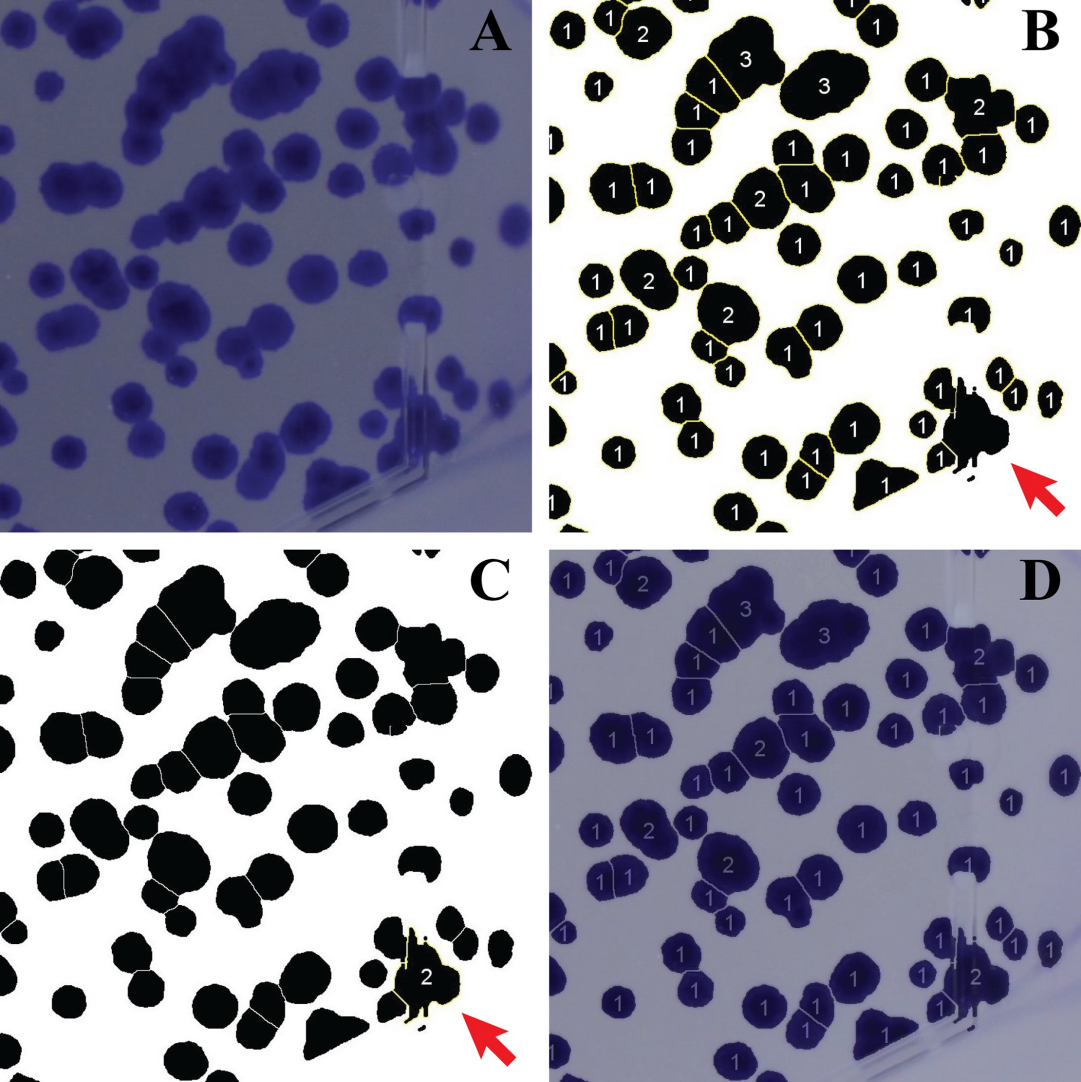
Counting rounds. A sample image showing V79 cells (panel A) is used to illustrate how the two counting rounds contained in the CoCoNut macro are performed. In panel B, the first round is executed to detect roundish colonies (size: from “smallest colony area” to “infinity”; circularity: from 0.5 to 1). In panel C, the second round detects an object characterized by a strange morphology (size: from “average colony area” to “infinity”; circularity: from 0 to 0.49). In D, panels B and C are added together and superimposed to A with an opacity of 30%. Digits always correspond to the number of colonies contained in each region.

After thresholding, the ImageJ built-in watershed algorithm is applied to separate merging colonies that show a smooth convex shape (Fig 4, panel G). Finally, the ImageJ command “Analyze particles” is executed to count the colonies (Fig 4, panel H), selecting on the basis of two parameters, called size and circularity. Circularity is defined as 4*π* ∙ (*Area/Perimeter*^2^). A circularity of 1 implies a perfect circle. As the value goes to 0, it typically indicates increasingly elongated polygons. For each sample, the “Analyze particles” command is executed twice. The first time (Fig 5, panel B) it aims to detect roundish colonies. Size values can range from a minimum, decided by the operator, to “infinity”. At the same time, circularity goes from 0.5 to 1. The second counting round (Fig 5, panel C) is necessary for finding colonies with unconventional morphology and whose area is bigger than that of the average particle contained in the analyzed flask. In this case, the minimum size is defined by the area of the average colony contained in the sample while the maximum remains “infinity”. Circularity ranges between 0 and 0.49.

Large colony counts are adjusted on the basis of the typical (average) size of a clone in the cell container under analysis (*e*.*g*. clones four times the average clone size will be counted as 4 individual clones).

At the end of the analysis of each dataset, CoCoNut provides the number of counts for each sample (results are saved in a .txt file) and generates separate output pictures. Two images (Fig 5, panels B and C) correspond to the two counting rounds performed by the macro and show CoCoNut scoring decisions, *i*.*e*. area-adjusted counts are displayed inside individual scoring regions. For example, the number “1” is displayed when only one colony is detected while the number “2” indicates that ImageJ detected a big colony that was scored as 2 on the basis of its dimensions. A third output image (Fig 5, panel D) superimposes the results of the counting rounds to the original sample image.

## Results and discussion

### Uniformity check

**Fig 6:**
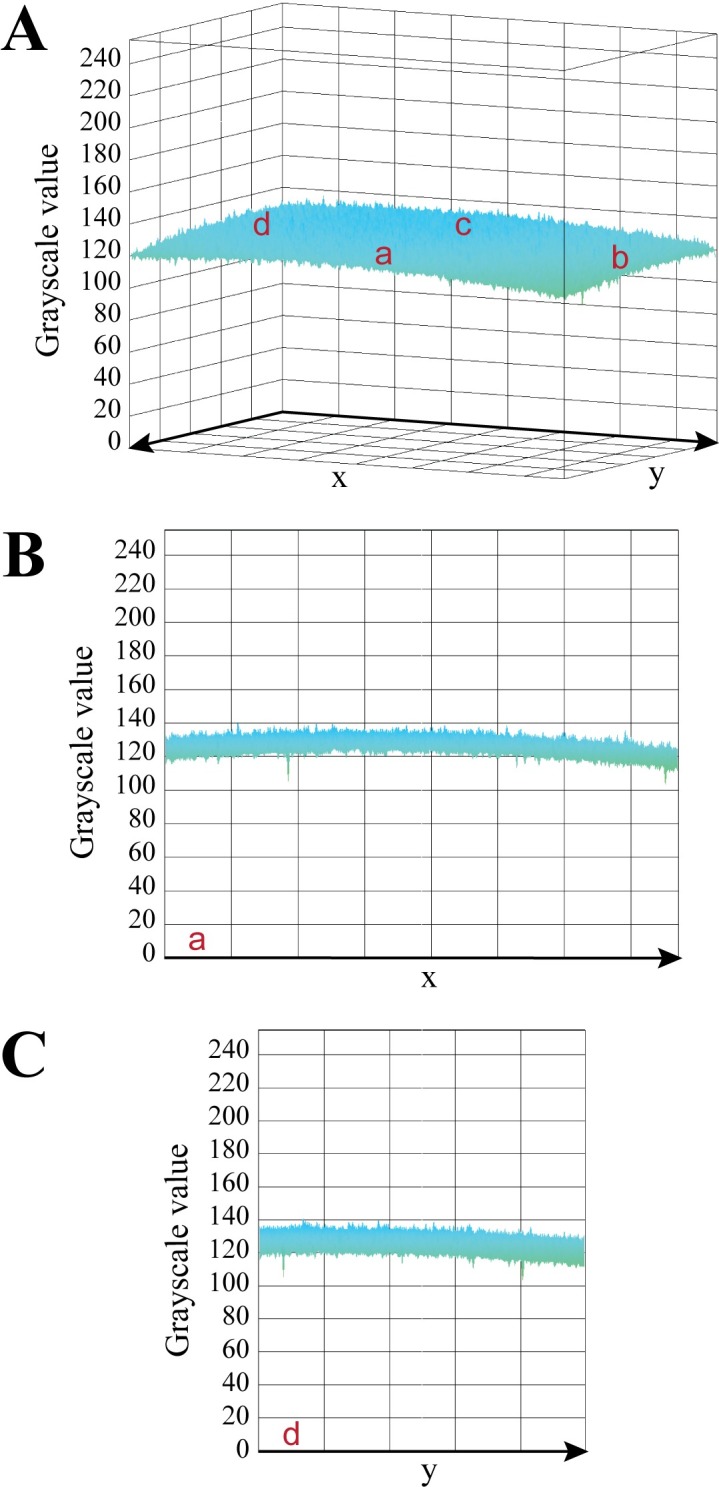
Uniformity check. Uniform lighting conditions have been tested inside the photo box using the X-Rite White balance target. After positioning the tool into the light-box, pictures of the target were taken, and images analyzed through the interactive 3D surface plot ImageJ plugin. The luminance of an image is interpreted as the height of the plot and it is measured across the whole surface (A). The viewing angle is adjusted in B and C to show the grayscale profile from two different sides: a and d, respectively.

Uniform lighting conditions have been tested for the photo-box using the X-Rite ColorChecker White Balance target. The tool was positioned inside the light-box; pictures of the target have been realized by using the interactive 3D surface plot ImageJ plugin. The mean grayscale value has been found to be 130.66 ± 3.91. Being the ColorChecker with an 18% gray surface, this result is in agreement with the expected theoretical value of 128. Results show a uniform distribution of light, with a reasonable signal drop from side *c* toward side *a* (Fig 6), which can be explained by the absence of a LED strip on the side of the door. A comparison between an office flatbed scanner with the CoCoNut light-box is presented in Fig A in S1 Fig.

### Scoring of the colonies

**Fig 7:**
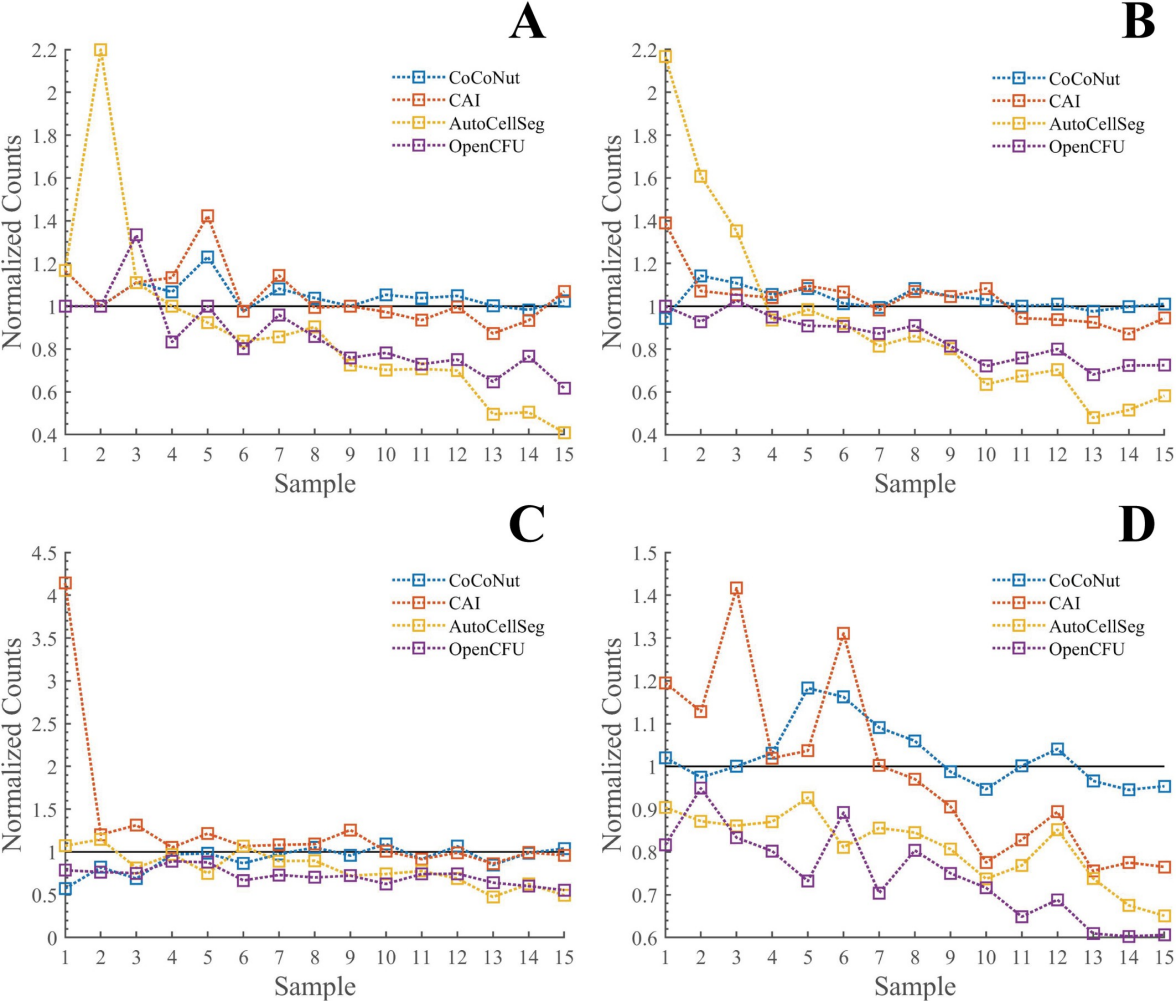
Automate counting. The scoring is performed with 35 mm cell culture dishes for V79 (A) and HeLa (C) cells. T25 cell culture flasks are used in panels B and D for V79 and HeLa cells, respectively. Plotted automated counts are normalized to the manual scorings, namely the average of the pooled data of three scorers. Dotted lines are displayed only to guide the eye and do not imply any kind of prediction.

Cell clones for V79 and HeLa cells seeded in T25 cell culture flasks and 35 mm cell culture dishes have been scored. Counts obtained by means of the automated counter have been compared to average counts achieved via standard manual counting by three scores (Fig 7). Manual scoring of V79 cells could be performed without the permanent use of a microscope due to the well-formed and stained colonies. HeLa cells required a much more extensive use of the microscope.

In Fig 7, CoCoNut results are compared with manual counting and to results obtained using CAI, AutoCellSeg, and OpenCFU. Results were obtained for V79 (panels A and B) and HeLa (panels C and D) cells. Cell clones were seeded in dishes (panels A and C) and flasks (panels B and D). The same ROI was employed in all tools but AutoCellSeg, which always analyzes the whole pictures. Automated counts are normalized to the manual scorings, namely the average of the pooled data of three scorers. Being error bars (calculated as Standard Error of the Mean (SEM) for the human counts) very small, they are not displayed in the plots. However, such information are included in S1–S4 Datasets.

Both CoCoNut and CAI can successfully reproduce manual counting results for the V79 cell line across the whole range of seeding densities (Fig 7A and 7B), although the latter shows a lower accuracy. Overall, CoCoNut performs better than CAI and its results are very similar to those obtained by the laboratory personnel. AutoCellSeg shows for both scenarios (dishes and flasks) a clear trend that goes from overestimating colonies at the lower densities to underestimating them at the higher ones. OpenCFU tends to underestimate scorings at the higher densities too, but in a more limited way.

V79 cells usually provide well-formed, well-distributed, and well-stained colonies (Fig 1A). To challenge CoCoNut (and the other tools) we have analyzed datasets of HeLa cells, a widely used human cancer cell line. We have challenged our scoring tools by overseeding our cell culture vessels to create cases where colonies overlap (Fig 1B). This was done to provide evidence that CoCoNut can separate cell clusters efficiently.

CoCoNut counting decisions are accurate for HeLa cells, especially when cells are seeded in cell culture flasks (panel D). Under the same circumstances, CAI overestimates colony numbers at the lower densities and underestimate them at the higher ones. Both AutoCellSeg and OpenCFU underestimate HeLa colony numbers for almost all the conditions (Fig 7C and 7D).

Although CoCoNut results for HeLa cells seeded in dishes are satisfactory (panel C), for this case discrepancies between manual and automated counts are more noticeable. Surprisingly, CAI shows an outlier for one of the very low-density samples, which are usually easier to score due to the absence of merging colonies. The outlier originates from a number of extra counts (false positive counts) on the edges of the dish (supporting information S1 Comparison to others\CAI\180501 HeLa Dish\1 Results.jpg).

Colonies on the sidewalls are always missed because they are never visible in the pictures.

Table 1 provides a way for summarizing all results of Fig 7 by calculating the Sum of Squared Errors (SSE), which is a measure of the discrepancy between the automated scorings and the manual counts. A small value indicates a good similarity to human counts and the smallest values always belong to CoCoNut.

**Table 1:** 

	V79 Dish	V79 Flask	HeLa Dish	HeLa Flask
CoCoNut	0,09 (1)	0,06 (1)	0,38 (1)	0,08 (1)
CAI	0,29 (2)	0,22 (2)	10,18 (4)	0,59 (2)
AutoCellSeg	2,73 (4)	2,96 (4)	1,12 (2)	0,63 (3)
OpenCFU	0,77 (3)	0,51 (3)	1,3 (3)	1,14 (4)

SSE values are calculated for all scenarios. Ranking is presented within parenthesis on a 1 to 4 scale, where 1 corresponds to the best rating and 4 to the worst.

### Sum of Squared Errors (SSE)

**Fig 8:**
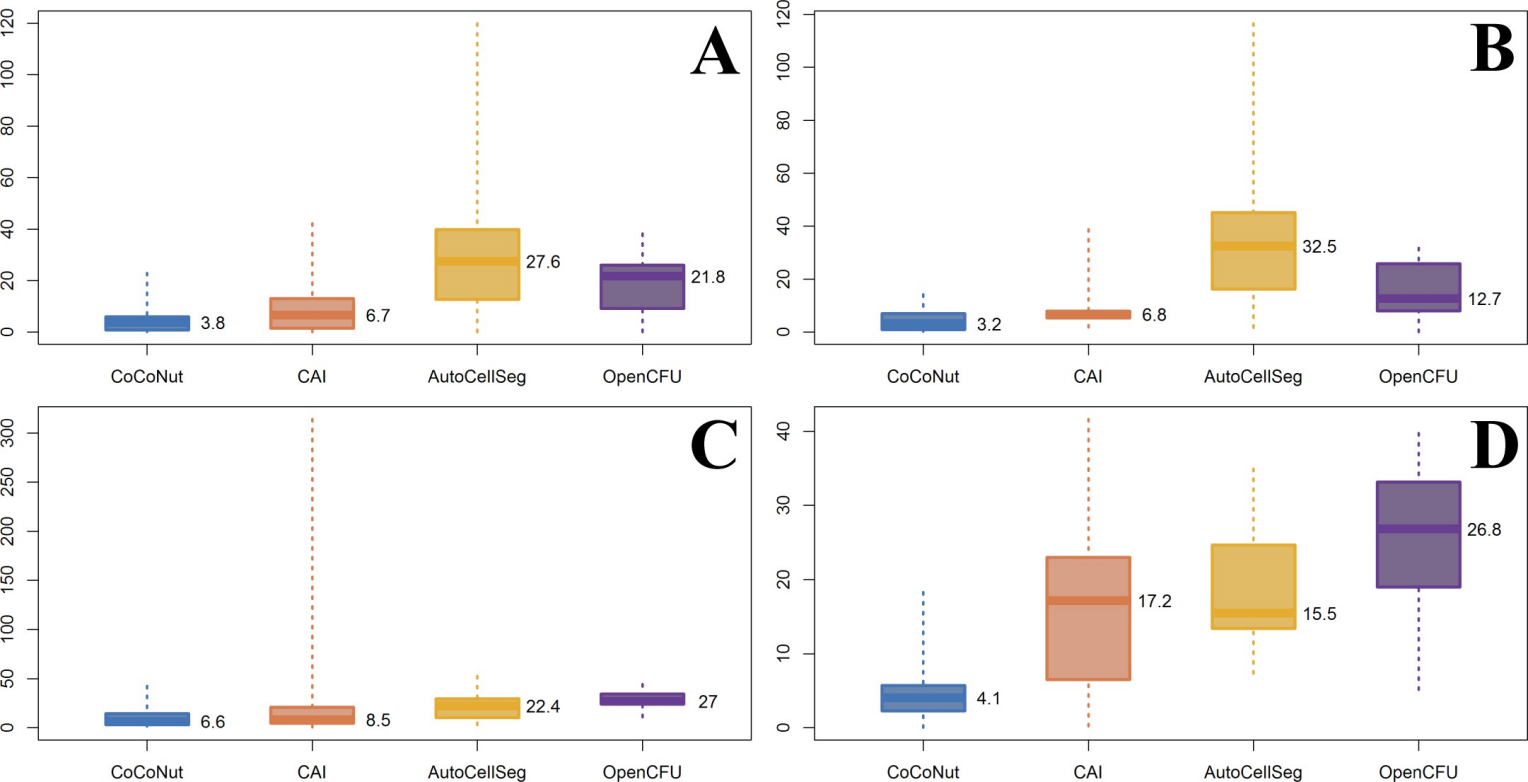
Boxplots. Relative errors are calculated for all experiments and expressed as percentages. A value of 100 means that the difference between automated and manual counts is as big as the “real” value (the manual count). Error distributions are summarized through the use of boxplots. The bottom and top of the boxes are the 25^th^ and 75^th^ percentile, respectively. The thick bands within the boxes show the 50^th^ percentile (the median). “Whiskers” represent minimum and maximum values. Panel A: V79 cells, dishes. Panel B: V79 cells, flasks. Panel C: HeLa cells, dishes. Panel D: HeLa cells, flasks.

For all scenarios, with no exception, boxplots (Fig 8) indicate that CoCoNut outperforms the other tools. Our macro results are very satisfactory, having maximum relative errors below 23% in panels A, B, and D. In Panel C, the top of the of the box (3^rd^ quartile) indicates that the 75% of measurements are given with a relative error smaller than 14.3%, which is still a very good result and the best one for that scenario. We agree with Geissmann [19] when he writes that moderate average deviations are often negligible compared to noise generated by other experimental factors.

Every provided analysis was based on the final scoring numbers given by the different tools. A more in-depth analysis would require some output images where the scored colonies are superimposed to the input files. This would allow the identification of true negatives, false negatives, together with true and false positives. Such feature is available in CoCoNut, AutoCellSeg, and OpenCFU. We decided to implement it also in CAI. By looking at the superimposed pictures we could see how scoring decisions vary among the tools (Fig B in S1 Fig). Moreover, we could confirm that CAI, as well as AutoCellSeg and OpenCFU, tends to underestimate the number of cell clones. This mainly happens because that macro does not include corrections based on the area of big colonies. However, due to the presence of many false positives near the edges, final numbers are generally close to the manual scoring (counterbalancing errors). A clarifying picture is presented as Fig C in S1 Fig.

Colony Edge is another ImageJ-based tool for colony counting experiments. It is more recent than CAI and we have briefly described it in the introduction. We tested Colony Edge with all our datasets but unfortunately, in our hands and for our samples, we could not calibrate it. Although calibration was successful for a test image provided by the author together with the article, we could not use Colony Edge for any of our samples. After multiple futile attempts, we decided to renounce the use of Colony Edge.

Problems during automated scoring can arise due to structural differences between cell culture containers (that are not all identical to each other) or because of careless positioning (the scope of our plastic jigs is to minimize this aspect). These issues are hardly detectable by the naked eye, but they are important in CoCoNut, which relies on a perfect alignment of their boundaries to perform accurate background removal. The division step is particularly sensitive to this aspect. Under poor positioning conditions, some colonies may disappear from the digitally polished background and others may be artificially generated by counting the flask borders as colonies.

According to our experience, it is more difficult to position flasks than dishes. We have tested the accuracy of our plastic jig by position 21 times the same empty flask into the light-box. Pictures were taken and CoCoNut was executed after randomly selecting one of the pictures to act as the control, namely the denominator of the division procedure. As the flask was empty, eventual counts were due to suboptimal positioning of the flask. Under such circumstances, CoCoNut returned 1 false positive out of 20 attempts (1 flask was used as the control).

The same experiment was repeated using 21 different empty flasks, obtaining a false positive rate of 8 out of 20.

This experiment suggests that CoCoNut scoring results could be further improved by labeling and imaging the vessels before their use, *i*.*e*. before seeding the cells. In this way, each sample has an individual control, avoiding issues related to structural differences among flasks.

Full datasets, CoCoNut results and summary tables containing the results of all the experiments are provided as supporting information in S1–S4 Datasets. Detailed information on the comparisons with CAI, AutoCellSeg, and OpenCFU can be found in S1 Comparison to others. At this point we would like to remind that Cai *et al*. did not provide a fully working macro and its use was made possible only by lines of code that we have written ourselves. Therefore, output images provided as supporting information in S1 Comparison to others are generated by lines of code that are not contained in Cai’s publication.

## Conclusions

ImageJ has been proven to be a good platform to accomplish an easy and successful development of computer-vision tools, such as the here introduced CoCoNut tool. CoCoNut will certainly simplify future radiobiological experiments, especially those of small laboratories that cannot afford to buy expensive commercial equipment for automated cell colony scoring. Reproducibility of results among different laboratories has been made easier thanks to the use of the 3D-printed light-box. We have presented convincing results for V79 and HeLa cells, using cell culture dishes and flasks and comparing our tool to other freely available programs. We have also shown the caveat that the accuracy of the results may depend on the quality of the cell containers, which is determined by the manufacturer. Future improvements of the CoCoNut tool will focus on the analysis of other cell lines and will further improve the quality of the jigs.

## Supporting information

S1 CoCoNut Files The supporting information file contains the CoCoNut macro and the 3D-printer files. (ZIP)

S1 Datasets The supporting information file contains datasets and CoCoNut results for V79 cells cultured in dishes. It also contains a text file where results achieved by automated (CoCoNut, CAI, AutoCellSeg, and OpenCFU) and manual methods are summarized.
(ZIP)

S2 Datasets The supporting information file contains datasets and CoCoNut results for V79 cells cultured in flasks. It also contains a text file where results achieved by automated (CoCoNut, CAI, AutoCellSeg, and OpenCFU) and manual methods are summarized.
(ZIP)

S3 Datasets The supporting information file contains datasets and CoCoNut results for HeLa cells cultured in dishes. It also contains a text file where results achieved by automated (CoCoNut, CAI, AutoCellSeg, and OpenCFU) and manual methods are summarized.
(ZIP)

S4 Datasets The supporting information file contains datasets and CoCoNut results for HeLa cells cultured in flasks. It also contains a text file where results achieved by automated (CoCoNut, CAI, AutoCellSeg, and OpenCFU) and manual methods are summarized.
(ZIP)

S1 Fig The supporting information file contains three figures, labeled A, B, and C.
Fig A. In the figure, we compare our own office flatbed scanner (A, on the left, WorkCentre 7775, Xerox, Ballerup, Denmark) with the CoCoNut light-box (B, on the right), which was coupled to the Canon camera described in the paper. We have tested uniform lighting using the X-Rite ColorChecker White Balance target (on the top) as described in the article.
Fig B. 171214 V79 Dish/1.jpg is used as an example to show how scoring decisions change among different tools. Red circles were added in post-production in correspondence to the counted cell clones in order to simplify the comparison. The yellow circle in A shows the region of interest selected in CoCoNut for the analysis. The same region was used in B and D, while AutoCellSeg does not provide such feature but it always analyzes the whole picture.
Fig C. This example (180501 HeLa Flask/7.jpg) illustrates how false positives by CAI (bottom) can counterbalance underestimated colony counts. In CoCoNut (top), numbers indicate the number of colonies contained within each scoring region, while they have just an ordering function in CAI, where regions can contain only 1 colony
(ZIP)

S1 Comparison to others Includes results and detailed information regarding CAI, AutoCellSeg, and OpenCFU experiments. (ZIP)
