# Supplementary figures and images for: Cell colony counter called CoCoNut (part 2 of 5)
Source: PLoS One. 2018 Nov 7;13(11):e0205823. doi: 10.1371/journal.pone.0205823 (PMC6221277; doi:10.1371/journal.pone.0205823)

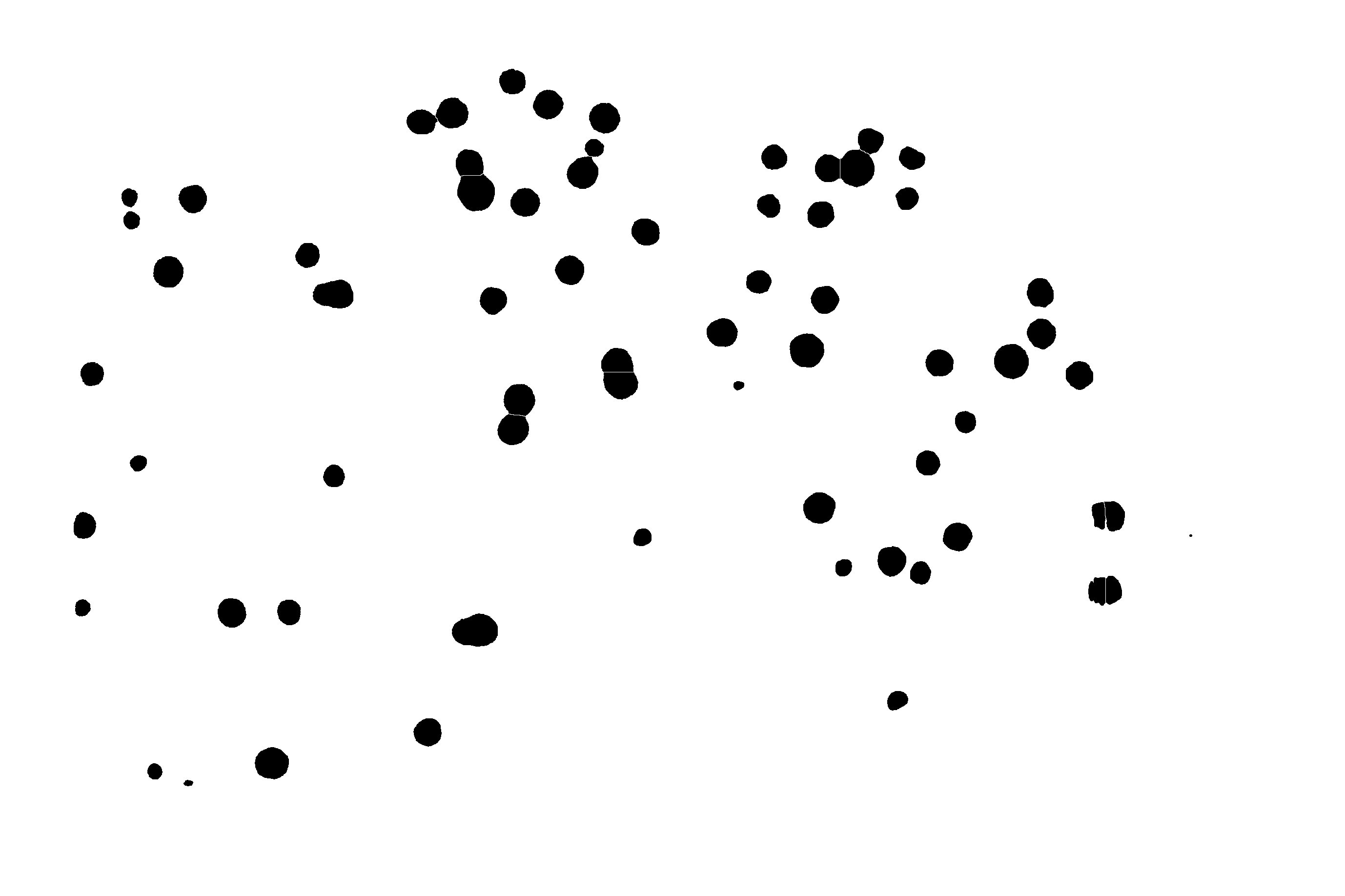

Supplement: S2 Datasets — It also contains a text file where results achieved by automated (CoCoNut, CAI, AutoCellSeg, and OpenCFU) and manual methods are summarized. (ZIP) [file pone.0205823.s003.zip › 171214 V79 Flask/4 Second counting.jpg]

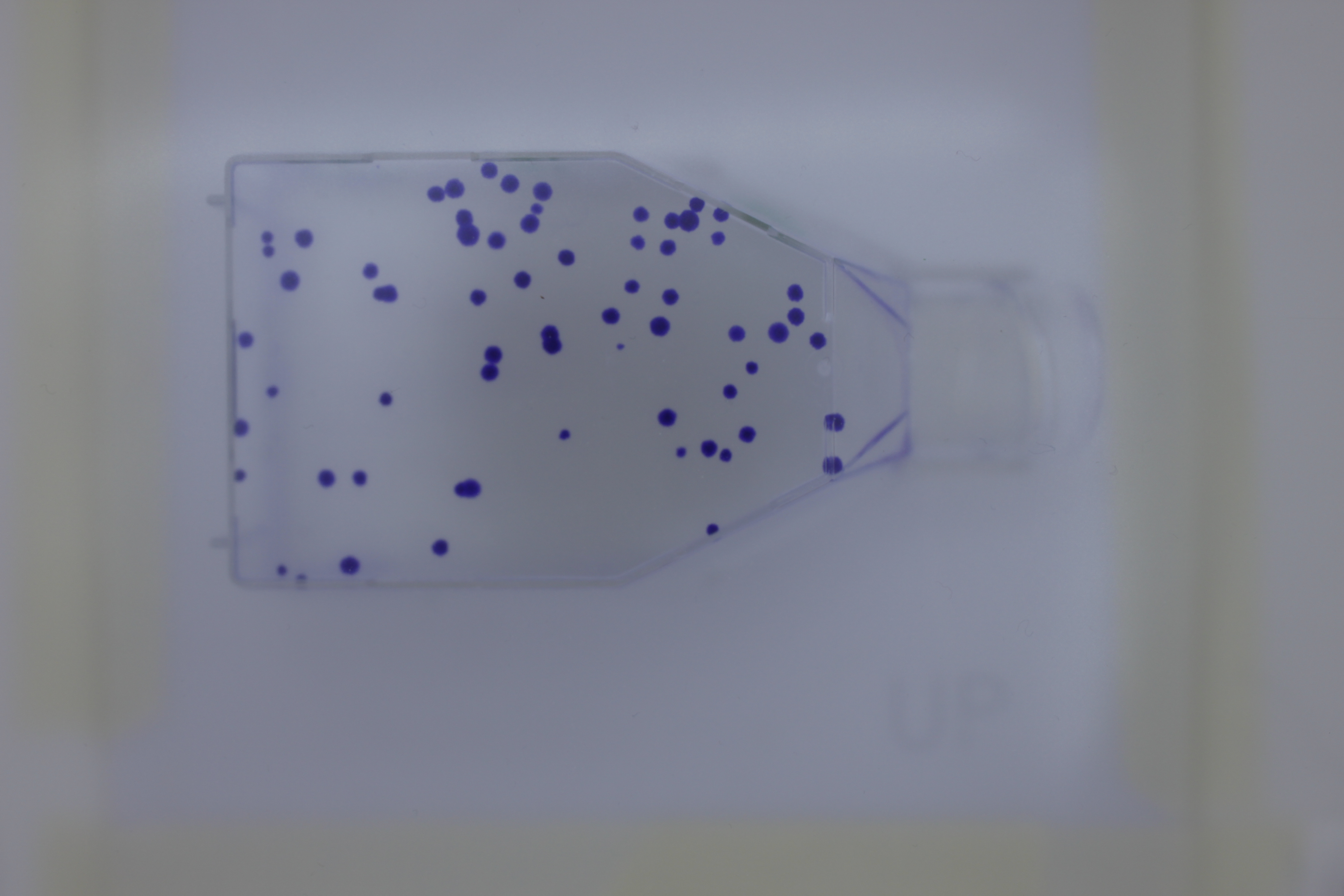

Supplement: S2 Datasets — It also contains a text file where results achieved by automated (CoCoNut, CAI, AutoCellSeg, and OpenCFU) and manual methods are summarized. (ZIP) [file pone.0205823.s003.zip › 171214 V79 Flask/4.JPG]

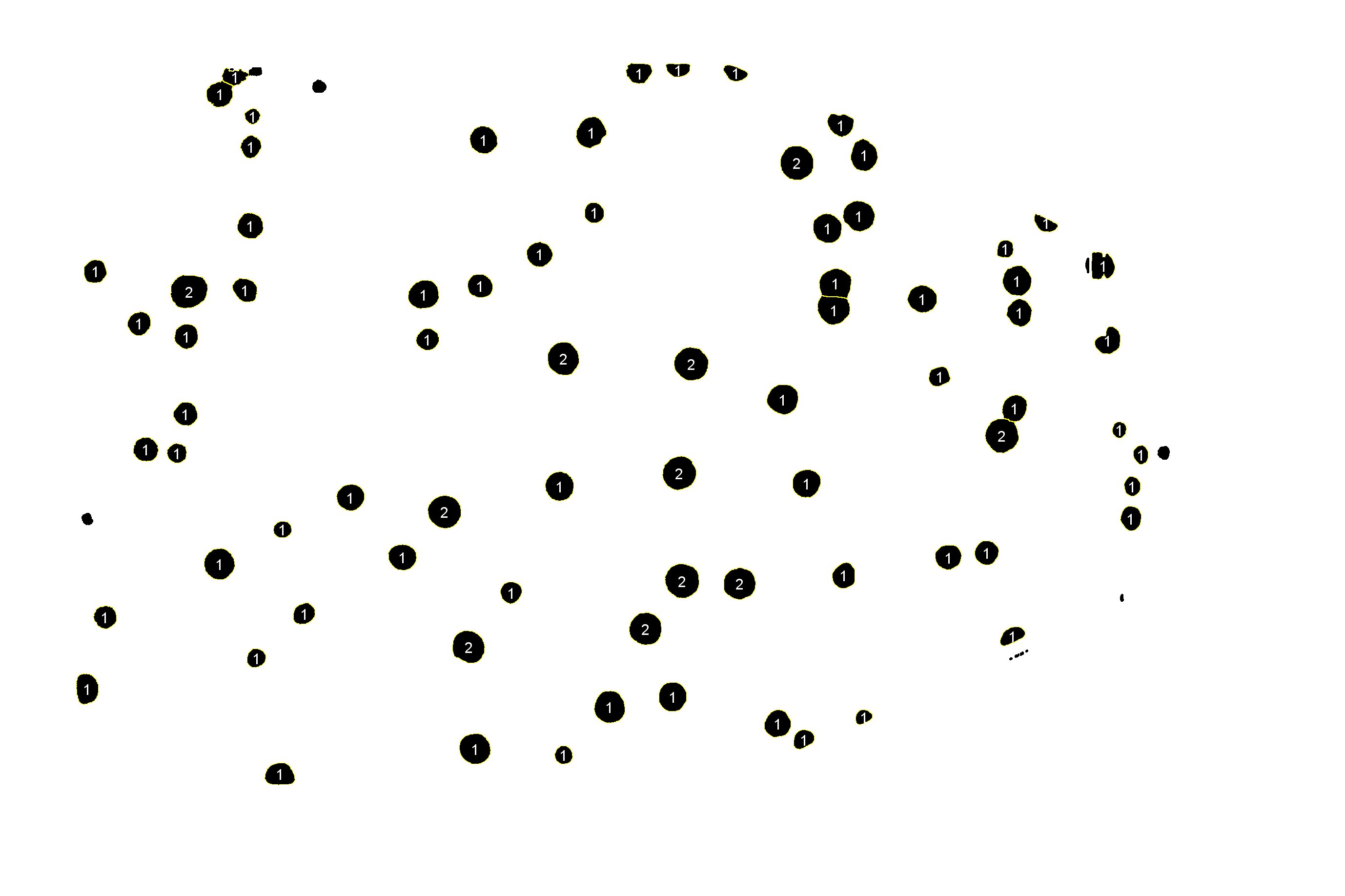

Supplement: S2 Datasets — It also contains a text file where results achieved by automated (CoCoNut, CAI, AutoCellSeg, and OpenCFU) and manual methods are summarized. (ZIP) [file pone.0205823.s003.zip › 171214 V79 Flask/5 First counting.jpg]

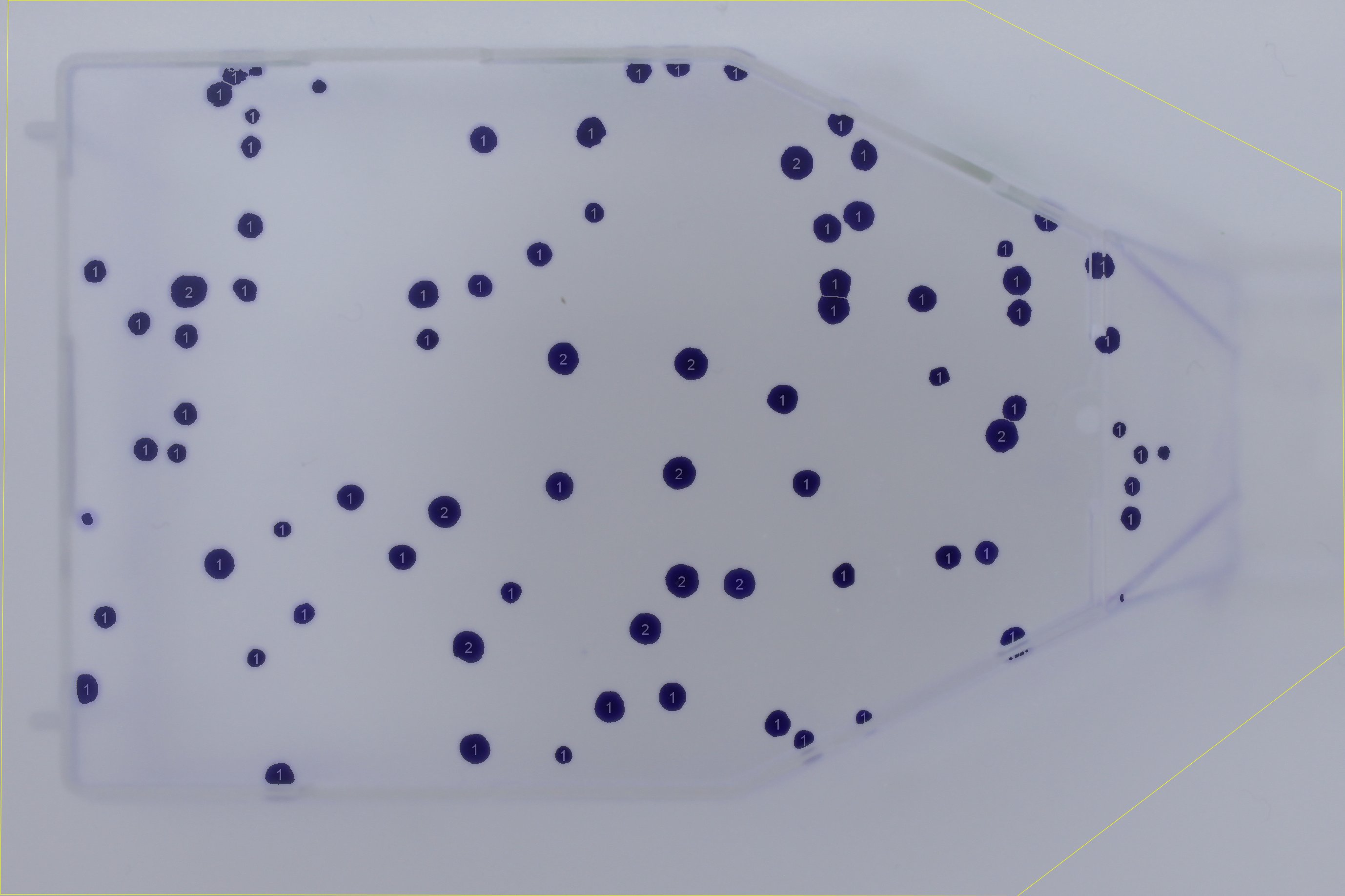

Supplement: S2 Datasets — It also contains a text file where results achieved by automated (CoCoNut, CAI, AutoCellSeg, and OpenCFU) and manual methods are summarized. (ZIP) [file pone.0205823.s003.zip › 171214 V79 Flask/5 Results.jpg]

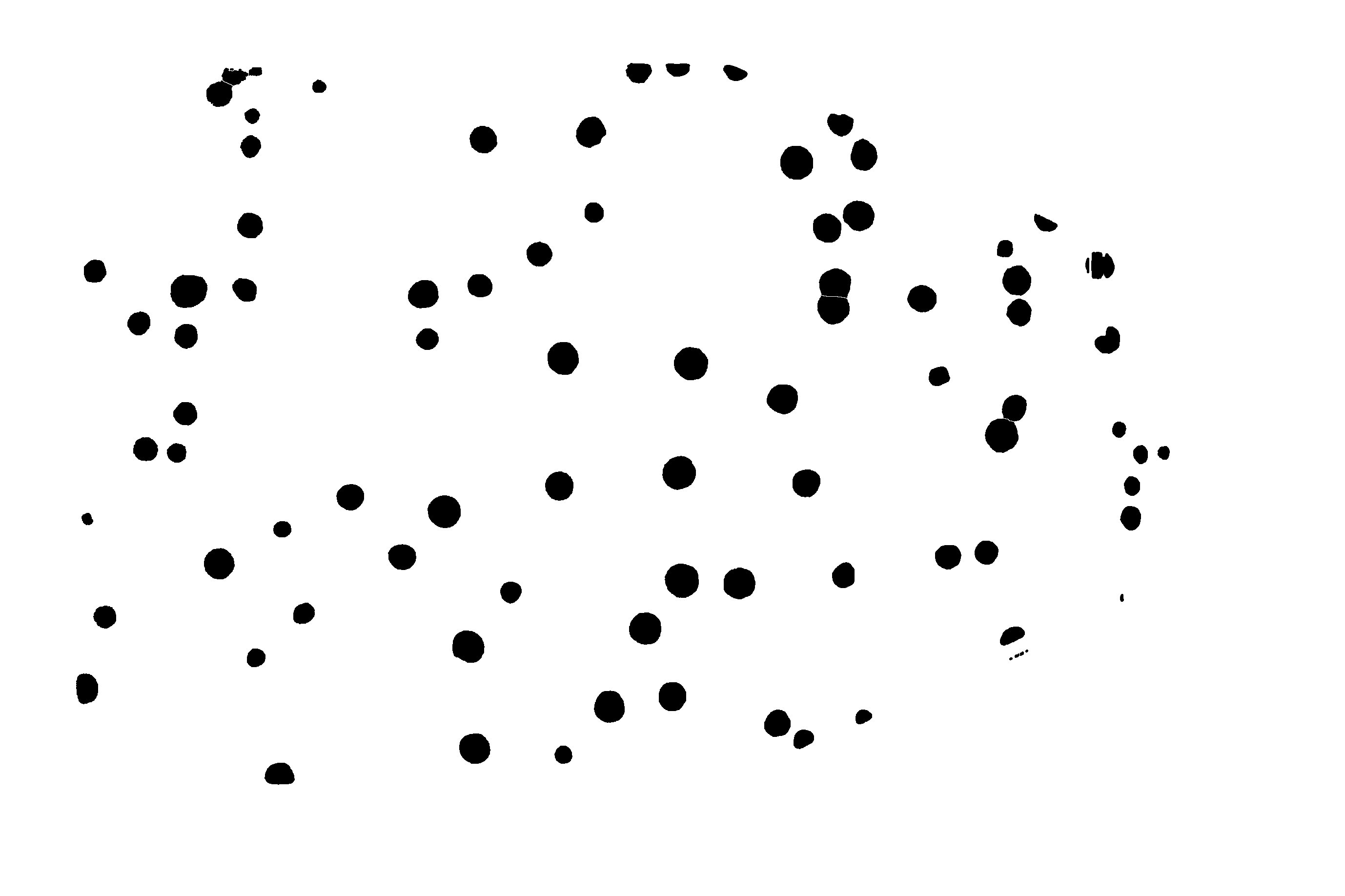

Supplement: S2 Datasets — It also contains a text file where results achieved by automated (CoCoNut, CAI, AutoCellSeg, and OpenCFU) and manual methods are summarized. (ZIP) [file pone.0205823.s003.zip › 171214 V79 Flask/5 Second counting.jpg]

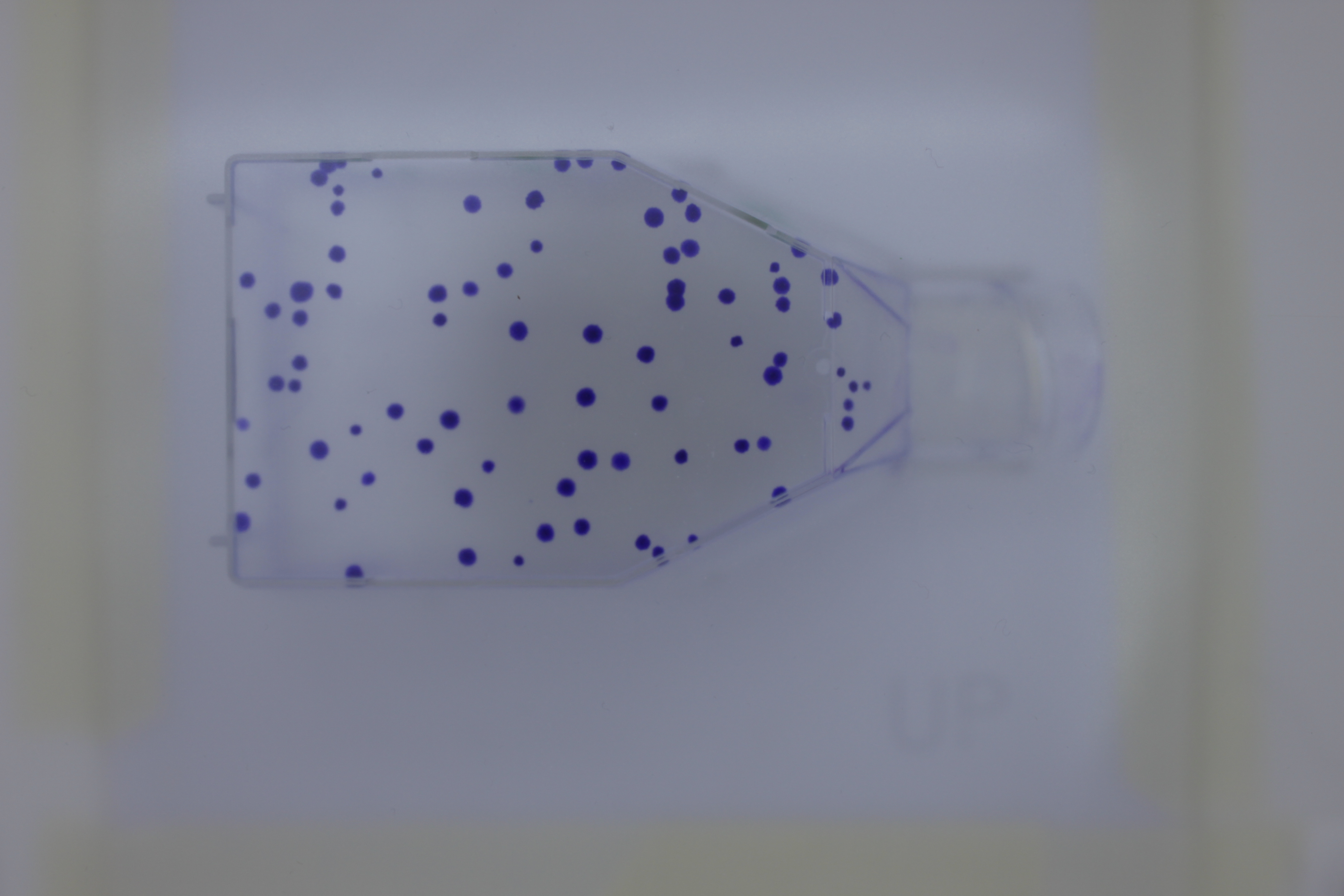

Supplement: S2 Datasets — It also contains a text file where results achieved by automated (CoCoNut, CAI, AutoCellSeg, and OpenCFU) and manual methods are summarized. (ZIP) [file pone.0205823.s003.zip › 171214 V79 Flask/5.JPG]

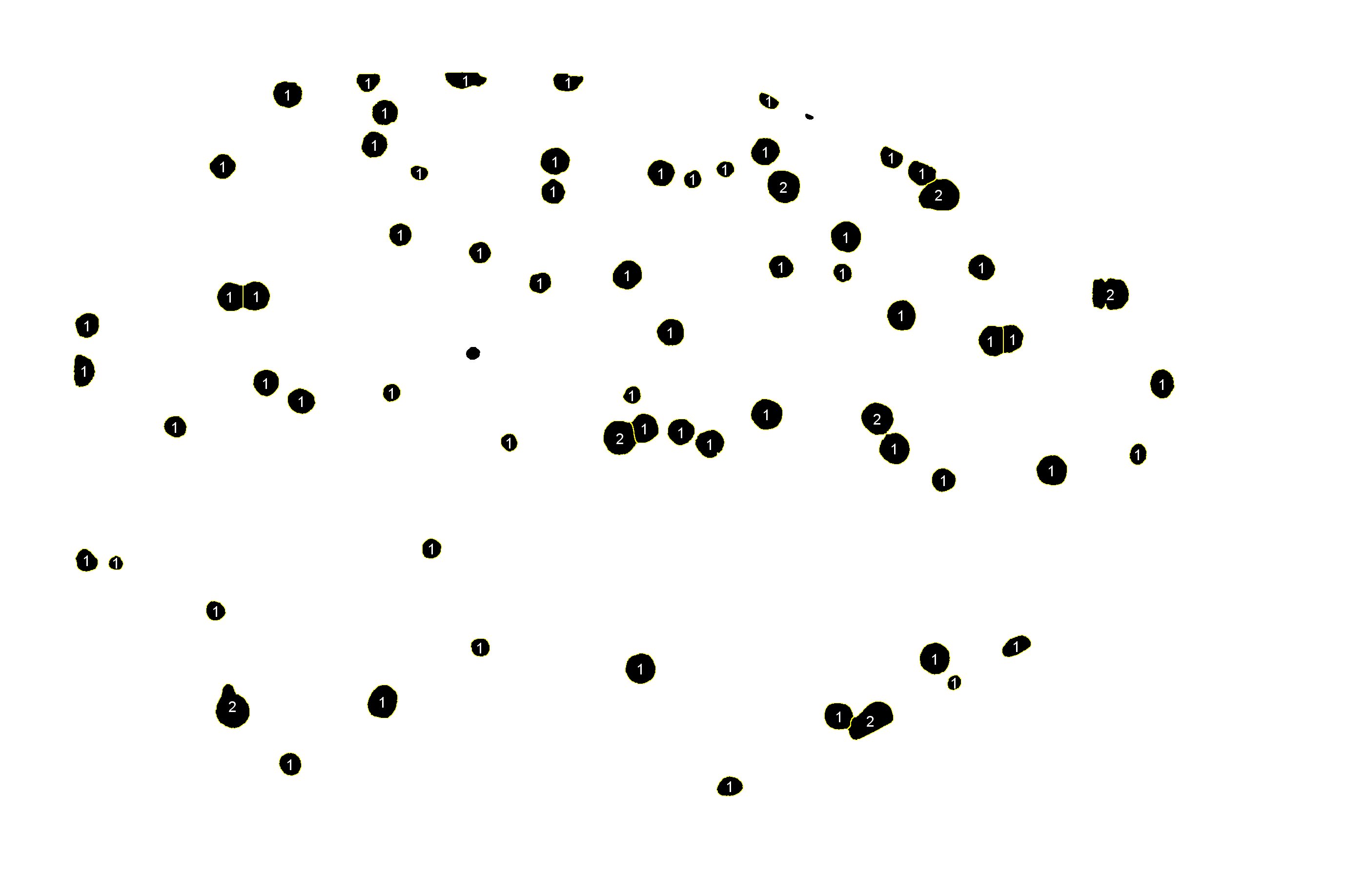

Supplement: S2 Datasets — It also contains a text file where results achieved by automated (CoCoNut, CAI, AutoCellSeg, and OpenCFU) and manual methods are summarized. (ZIP) [file pone.0205823.s003.zip › 171214 V79 Flask/6 First counting.jpg]

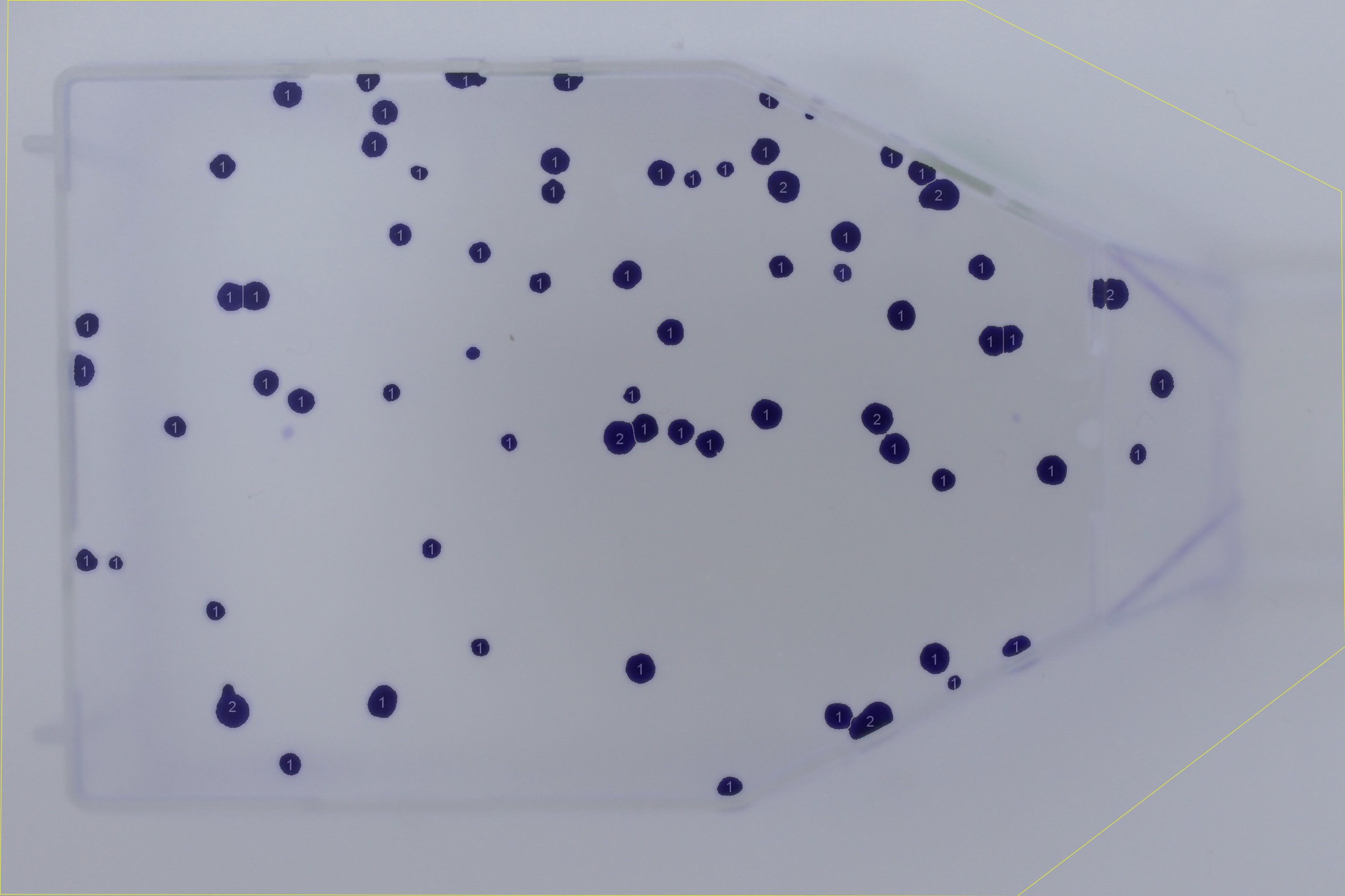

Supplement: S2 Datasets — It also contains a text file where results achieved by automated (CoCoNut, CAI, AutoCellSeg, and OpenCFU) and manual methods are summarized. (ZIP) [file pone.0205823.s003.zip › 171214 V79 Flask/6 Results.jpg]

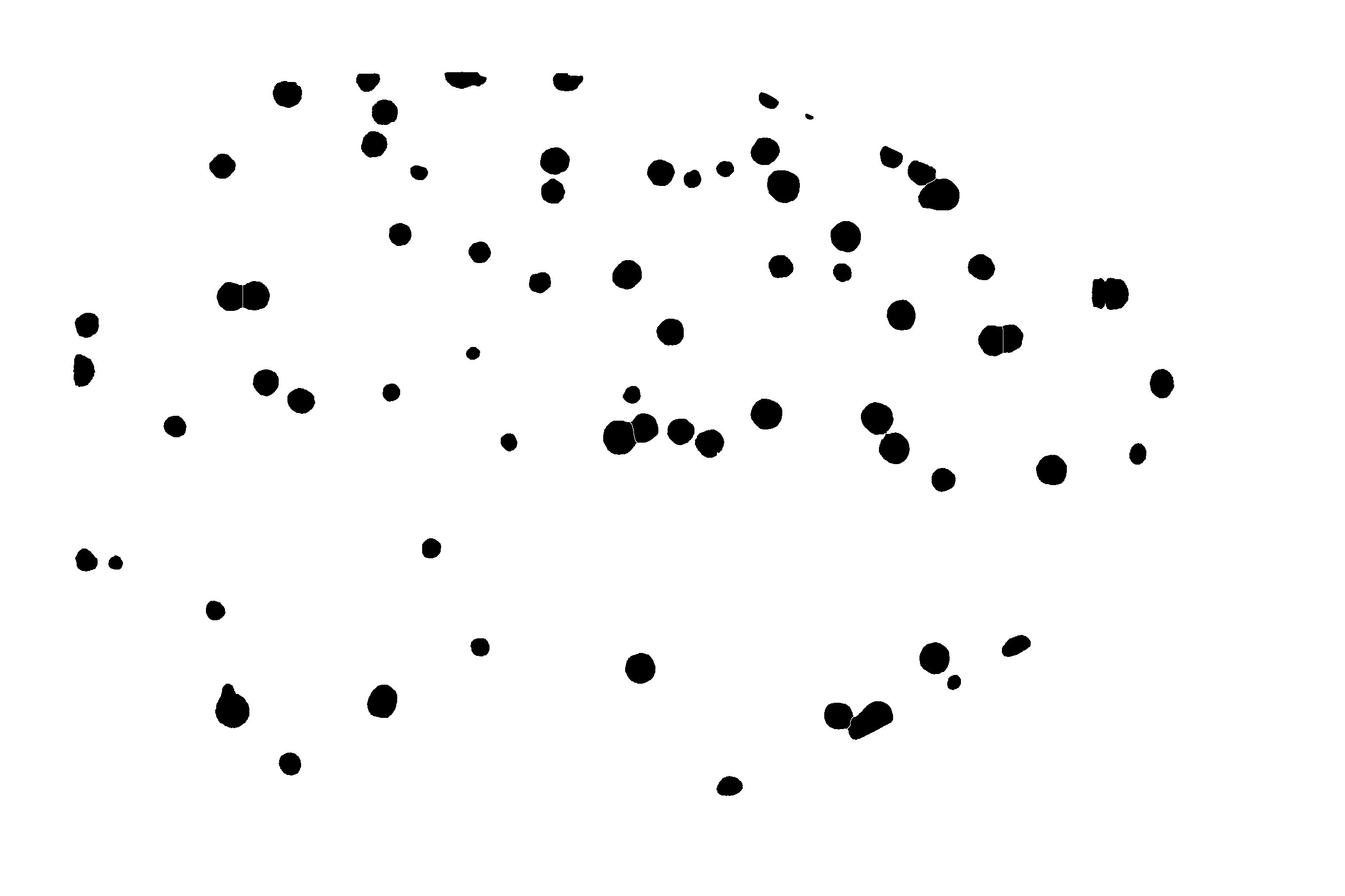

Supplement: S2 Datasets — It also contains a text file where results achieved by automated (CoCoNut, CAI, AutoCellSeg, and OpenCFU) and manual methods are summarized. (ZIP) [file pone.0205823.s003.zip › 171214 V79 Flask/6 Second counting.jpg]

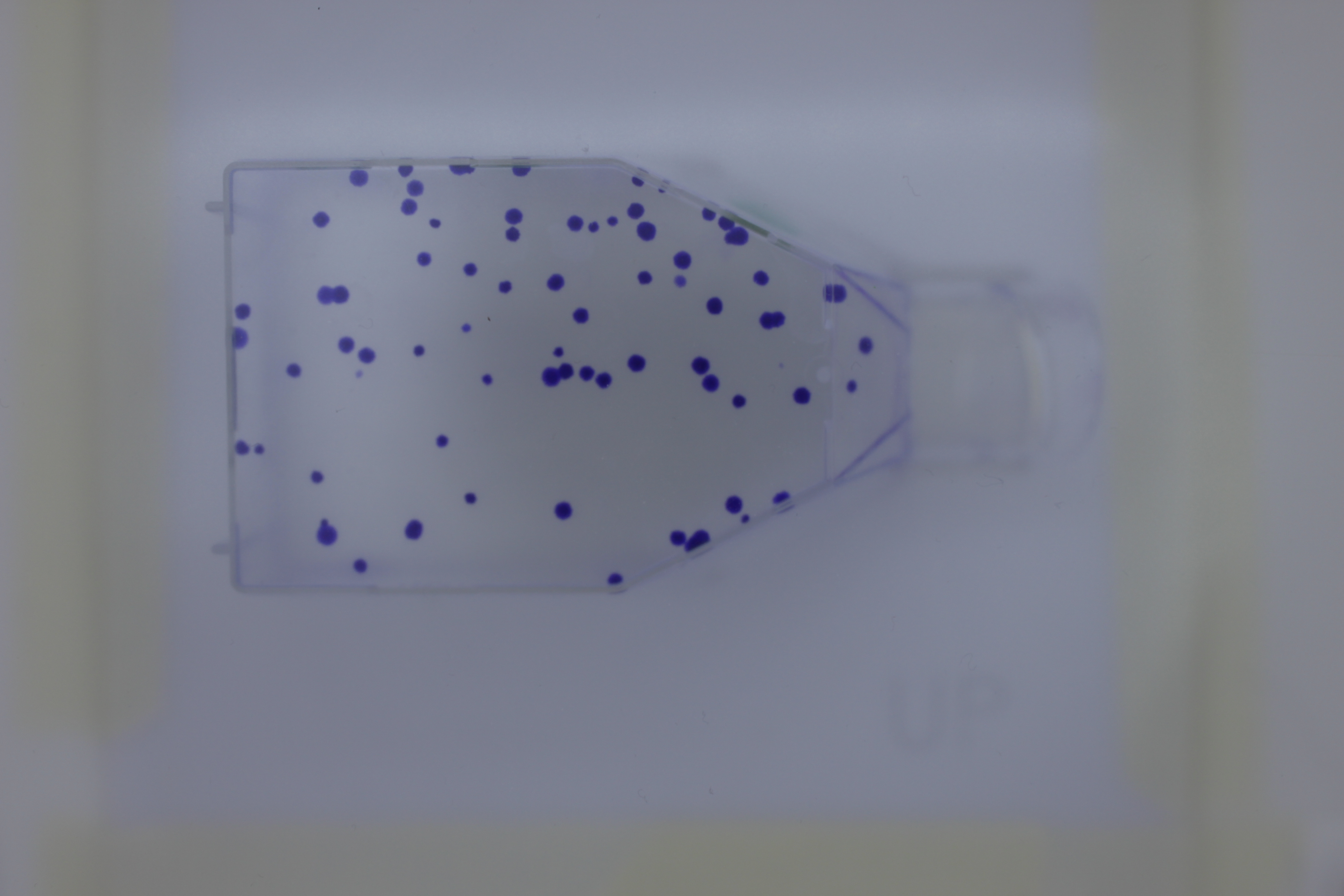

Supplement: S2 Datasets — It also contains a text file where results achieved by automated (CoCoNut, CAI, AutoCellSeg, and OpenCFU) and manual methods are summarized. (ZIP) [file pone.0205823.s003.zip › 171214 V79 Flask/6.JPG]

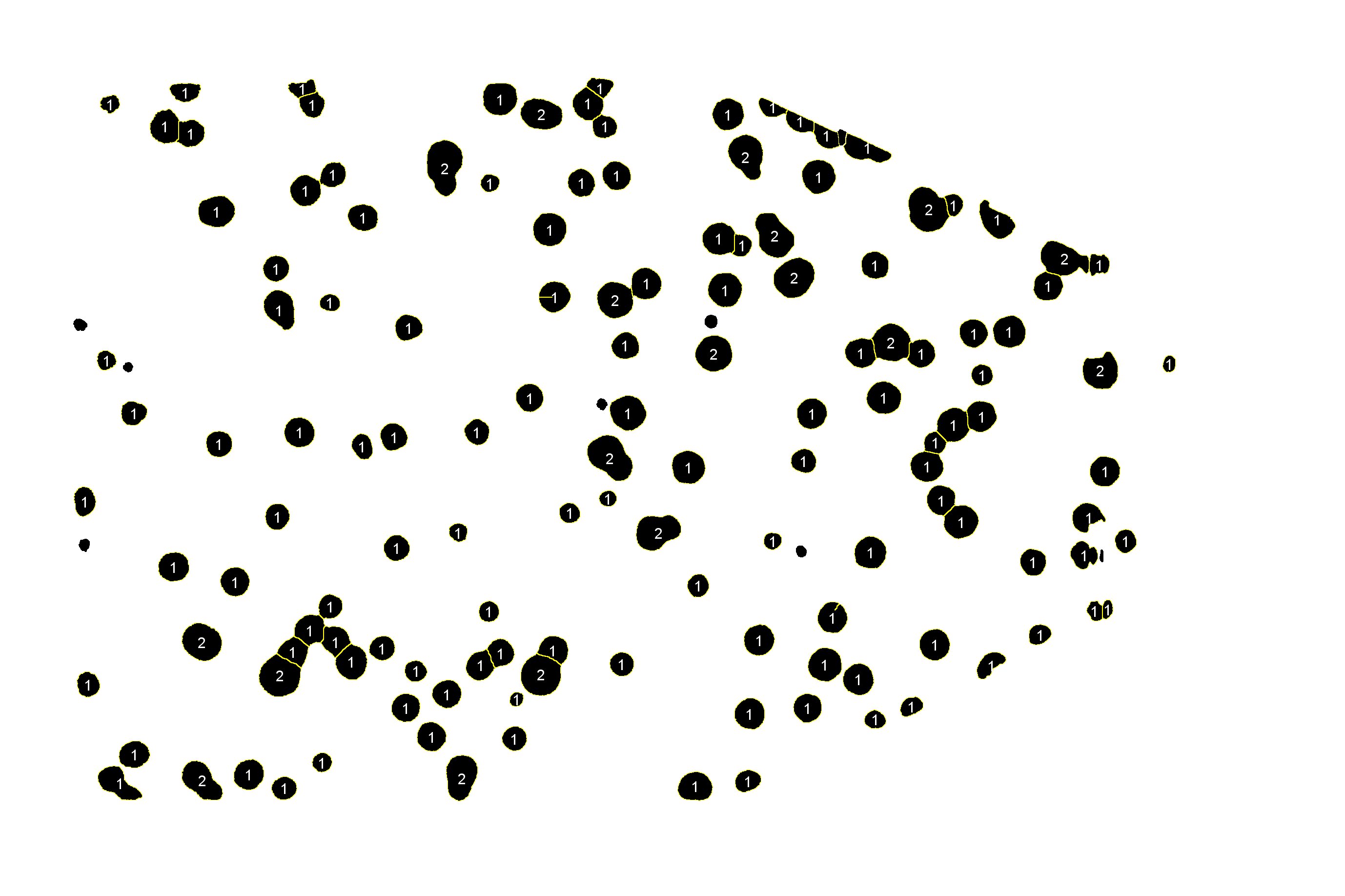

Supplement: S2 Datasets — It also contains a text file where results achieved by automated (CoCoNut, CAI, AutoCellSeg, and OpenCFU) and manual methods are summarized. (ZIP) [file pone.0205823.s003.zip › 171214 V79 Flask/7 First counting.jpg]

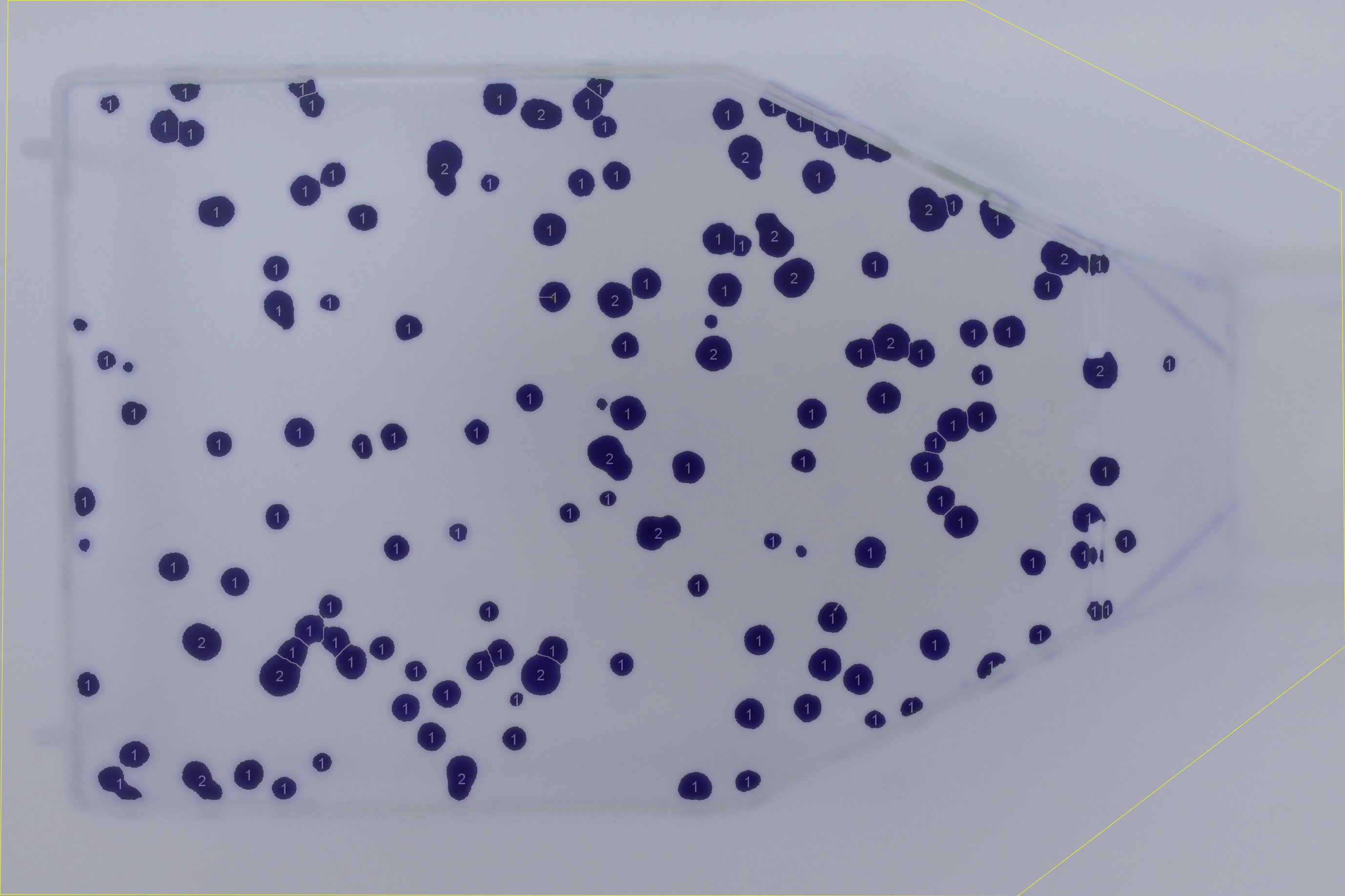

Supplement: S2 Datasets — It also contains a text file where results achieved by automated (CoCoNut, CAI, AutoCellSeg, and OpenCFU) and manual methods are summarized. (ZIP) [file pone.0205823.s003.zip › 171214 V79 Flask/7 Results.jpg]

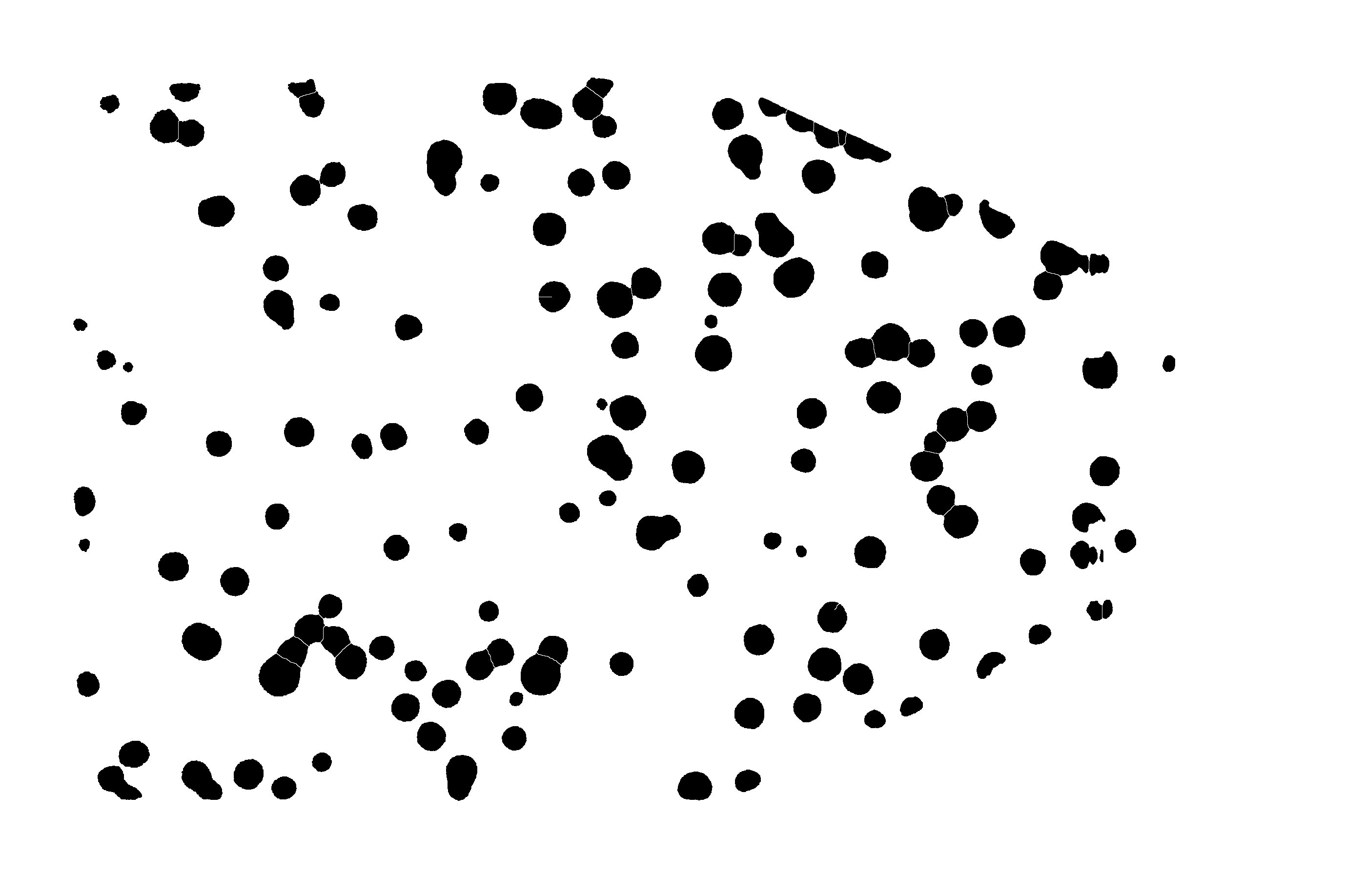

Supplement: S2 Datasets — It also contains a text file where results achieved by automated (CoCoNut, CAI, AutoCellSeg, and OpenCFU) and manual methods are summarized. (ZIP) [file pone.0205823.s003.zip › 171214 V79 Flask/7 Second counting.jpg]

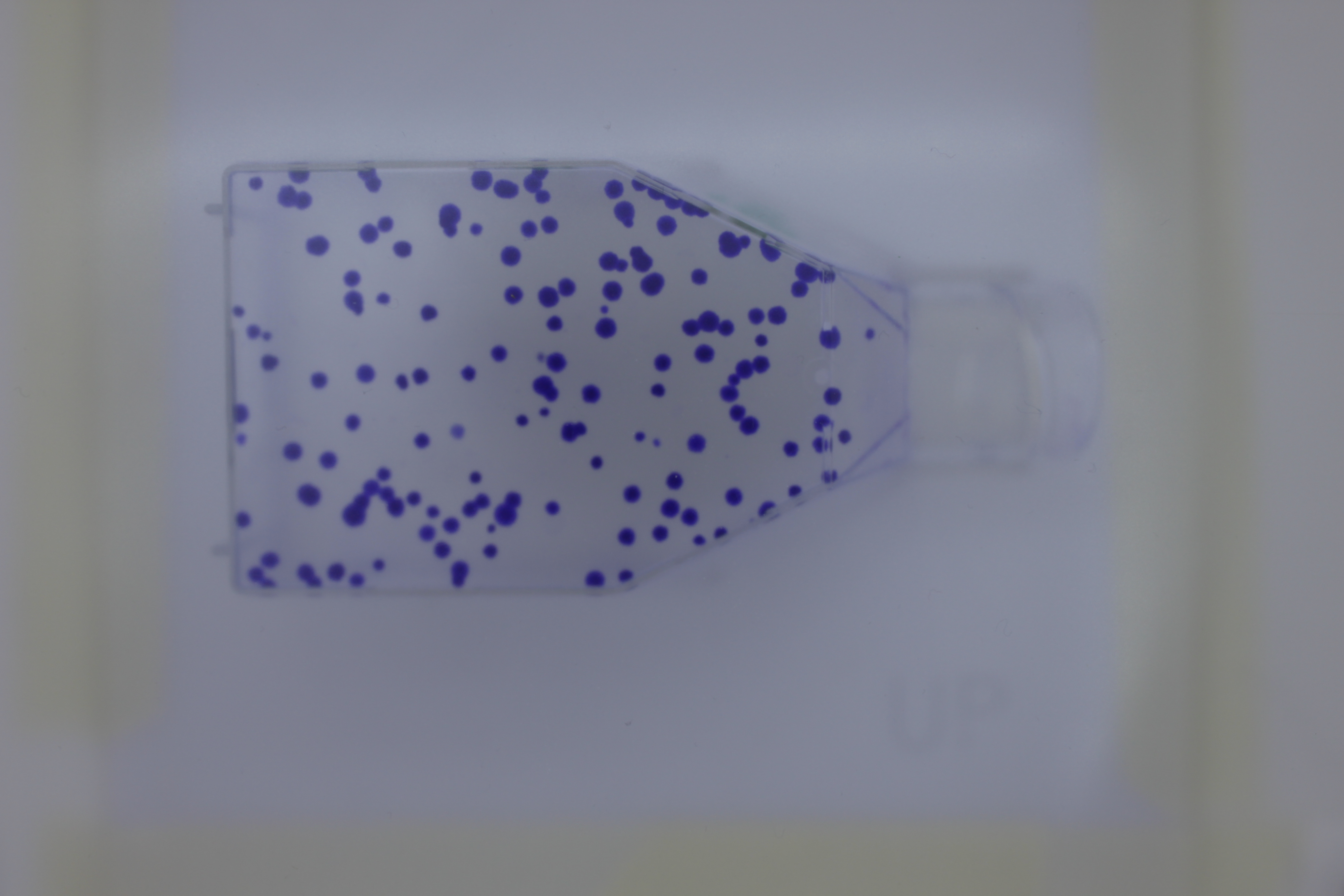

Supplement: S2 Datasets — It also contains a text file where results achieved by automated (CoCoNut, CAI, AutoCellSeg, and OpenCFU) and manual methods are summarized. (ZIP) [file pone.0205823.s003.zip › 171214 V79 Flask/7.JPG]

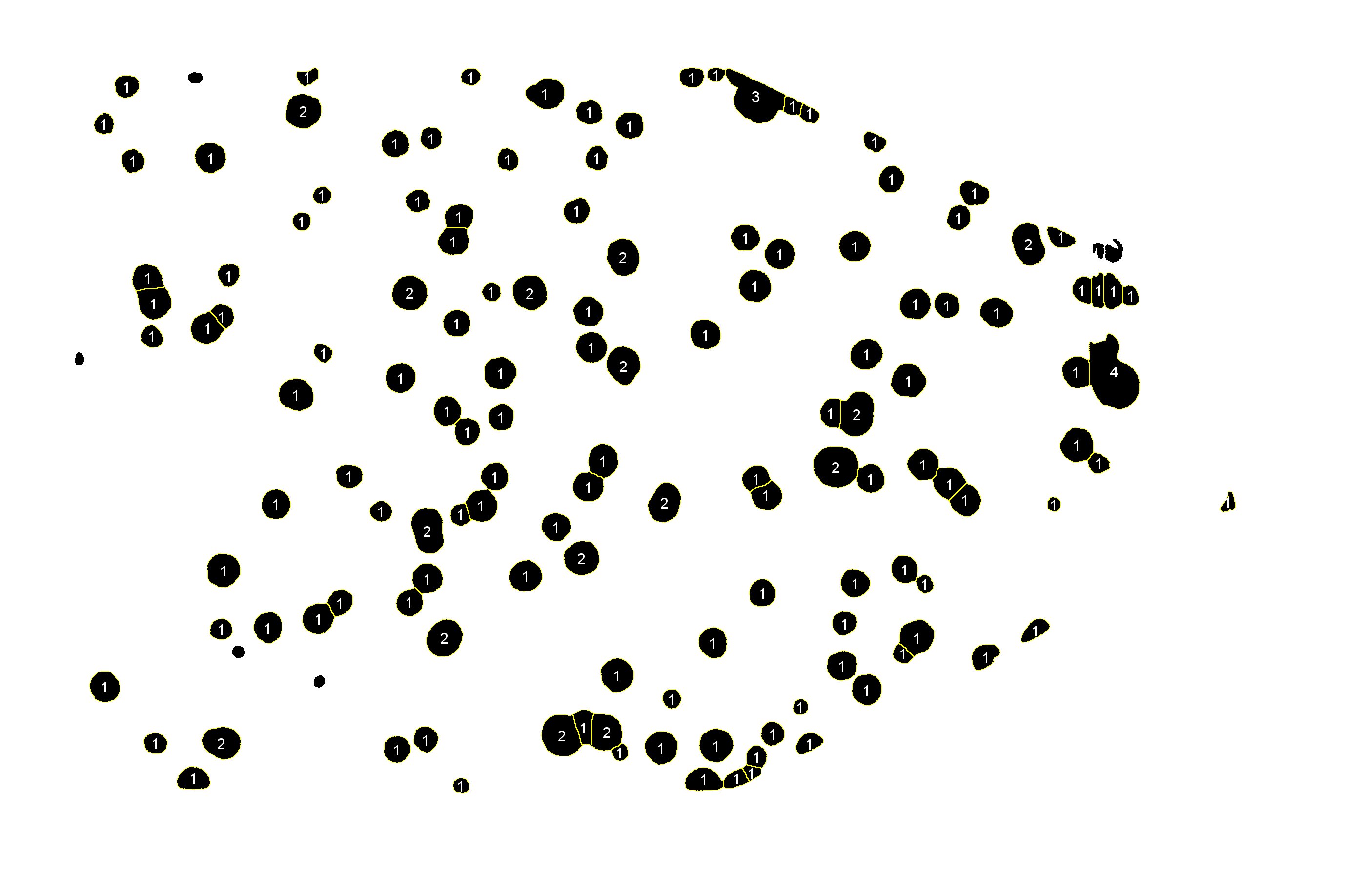

Supplement: S2 Datasets — It also contains a text file where results achieved by automated (CoCoNut, CAI, AutoCellSeg, and OpenCFU) and manual methods are summarized. (ZIP) [file pone.0205823.s003.zip › 171214 V79 Flask/8 First counting.jpg]

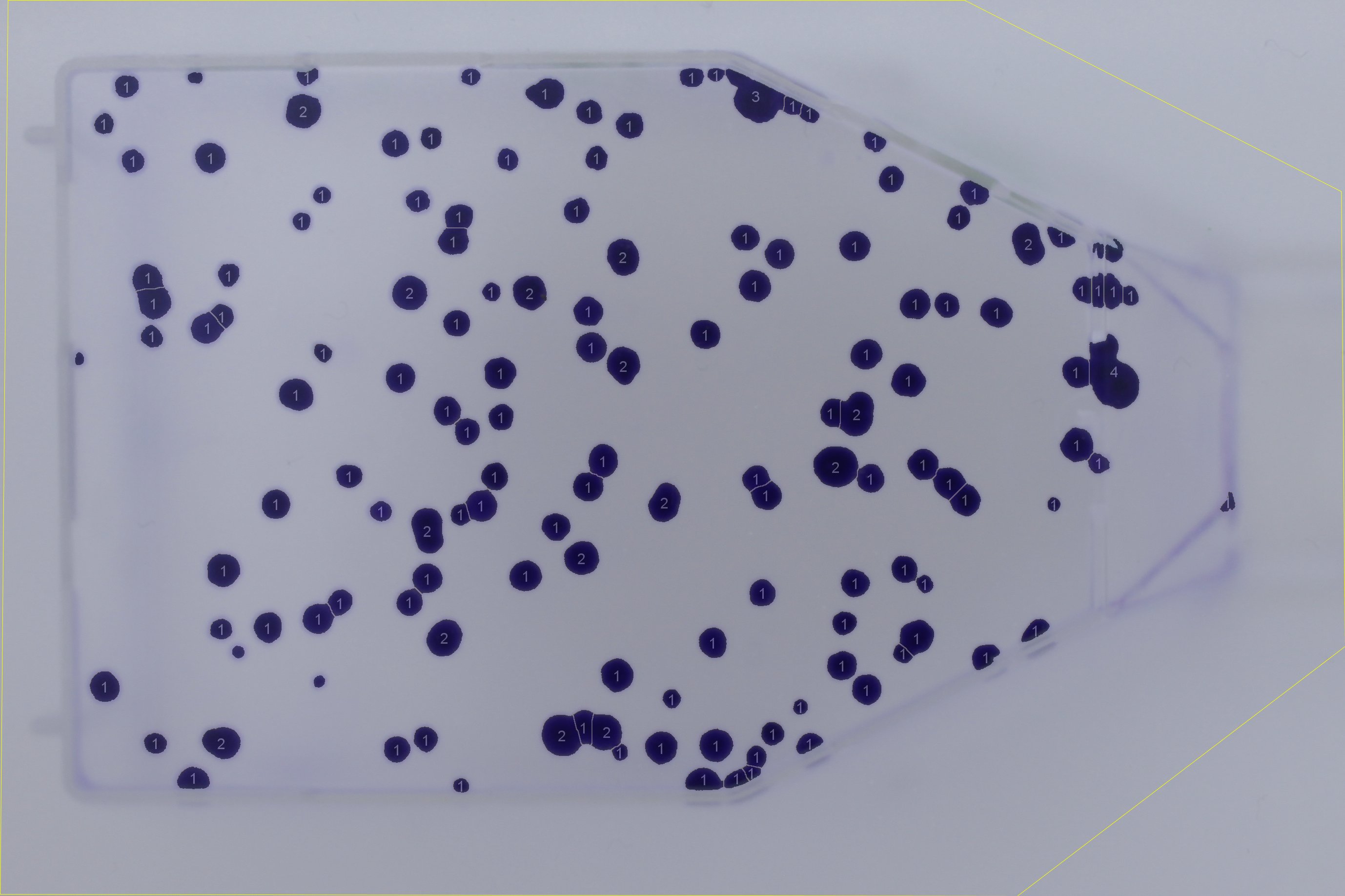

Supplement: S2 Datasets — It also contains a text file where results achieved by automated (CoCoNut, CAI, AutoCellSeg, and OpenCFU) and manual methods are summarized. (ZIP) [file pone.0205823.s003.zip › 171214 V79 Flask/8 Results.jpg]

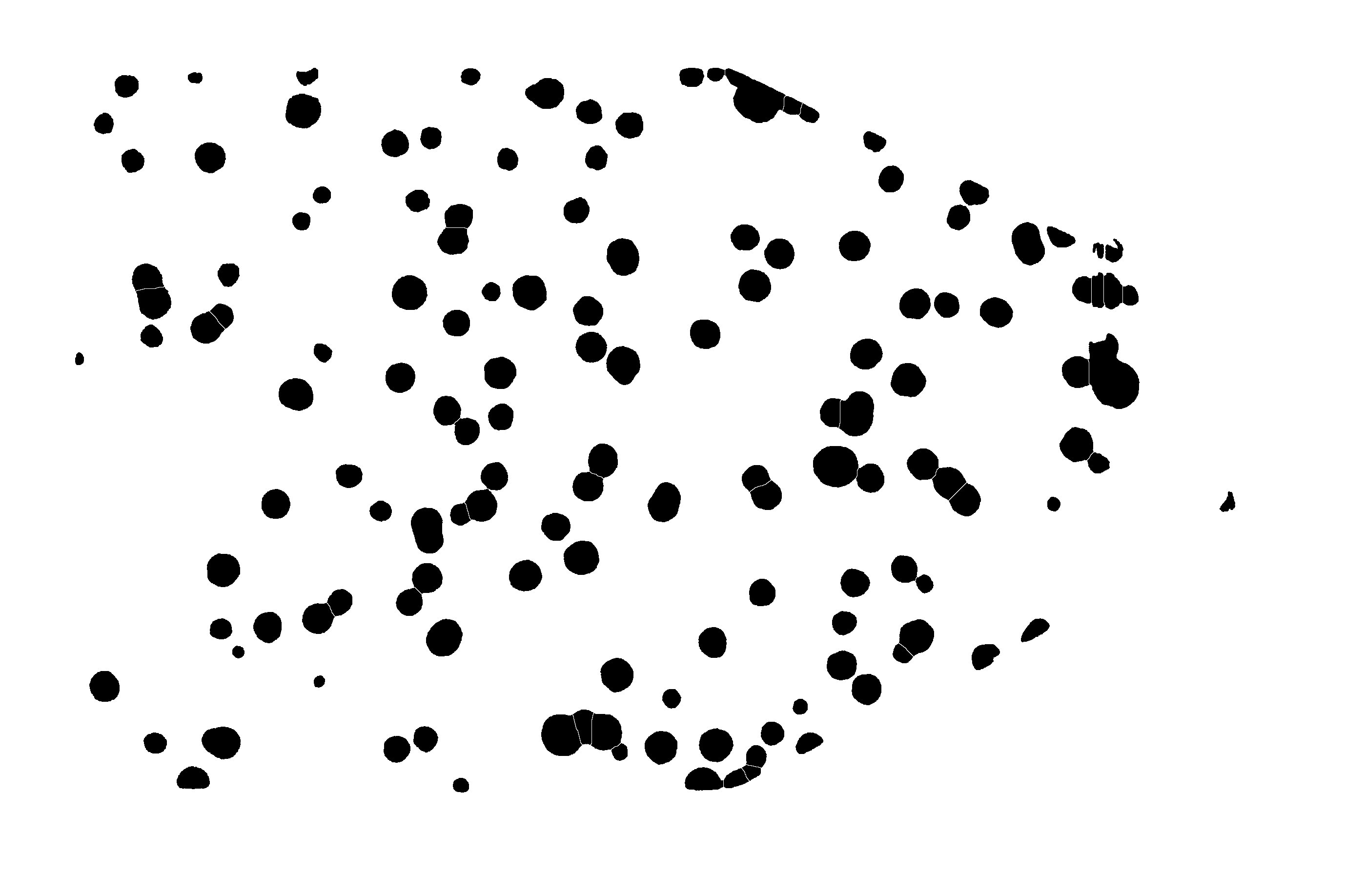

Supplement: S2 Datasets — It also contains a text file where results achieved by automated (CoCoNut, CAI, AutoCellSeg, and OpenCFU) and manual methods are summarized. (ZIP) [file pone.0205823.s003.zip › 171214 V79 Flask/8 Second counting.jpg]

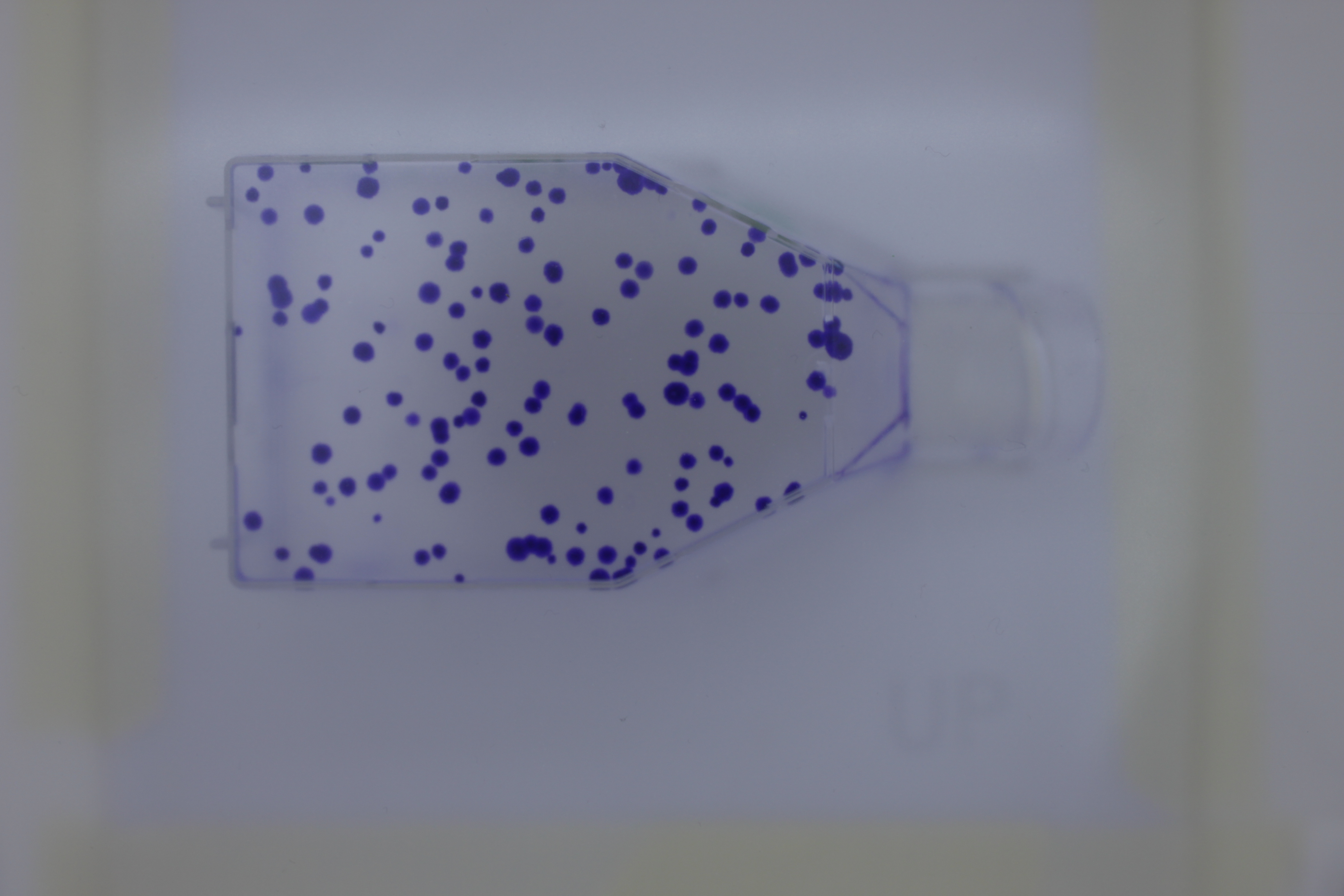

Supplement: S2 Datasets — It also contains a text file where results achieved by automated (CoCoNut, CAI, AutoCellSeg, and OpenCFU) and manual methods are summarized. (ZIP) [file pone.0205823.s003.zip › 171214 V79 Flask/8.JPG]

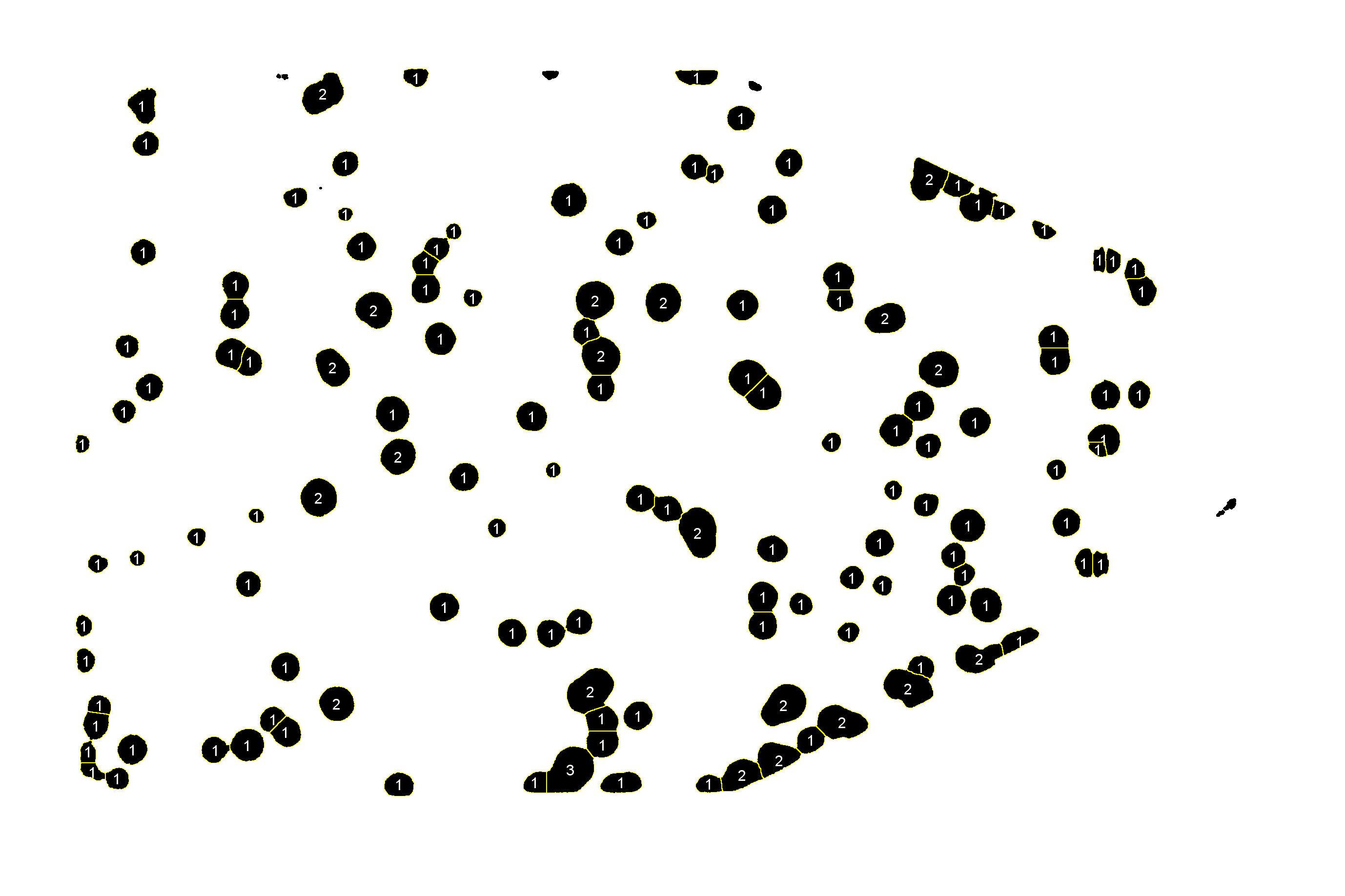

Supplement: S2 Datasets — It also contains a text file where results achieved by automated (CoCoNut, CAI, AutoCellSeg, and OpenCFU) and manual methods are summarized. (ZIP) [file pone.0205823.s003.zip › 171214 V79 Flask/9 First counting.jpg]

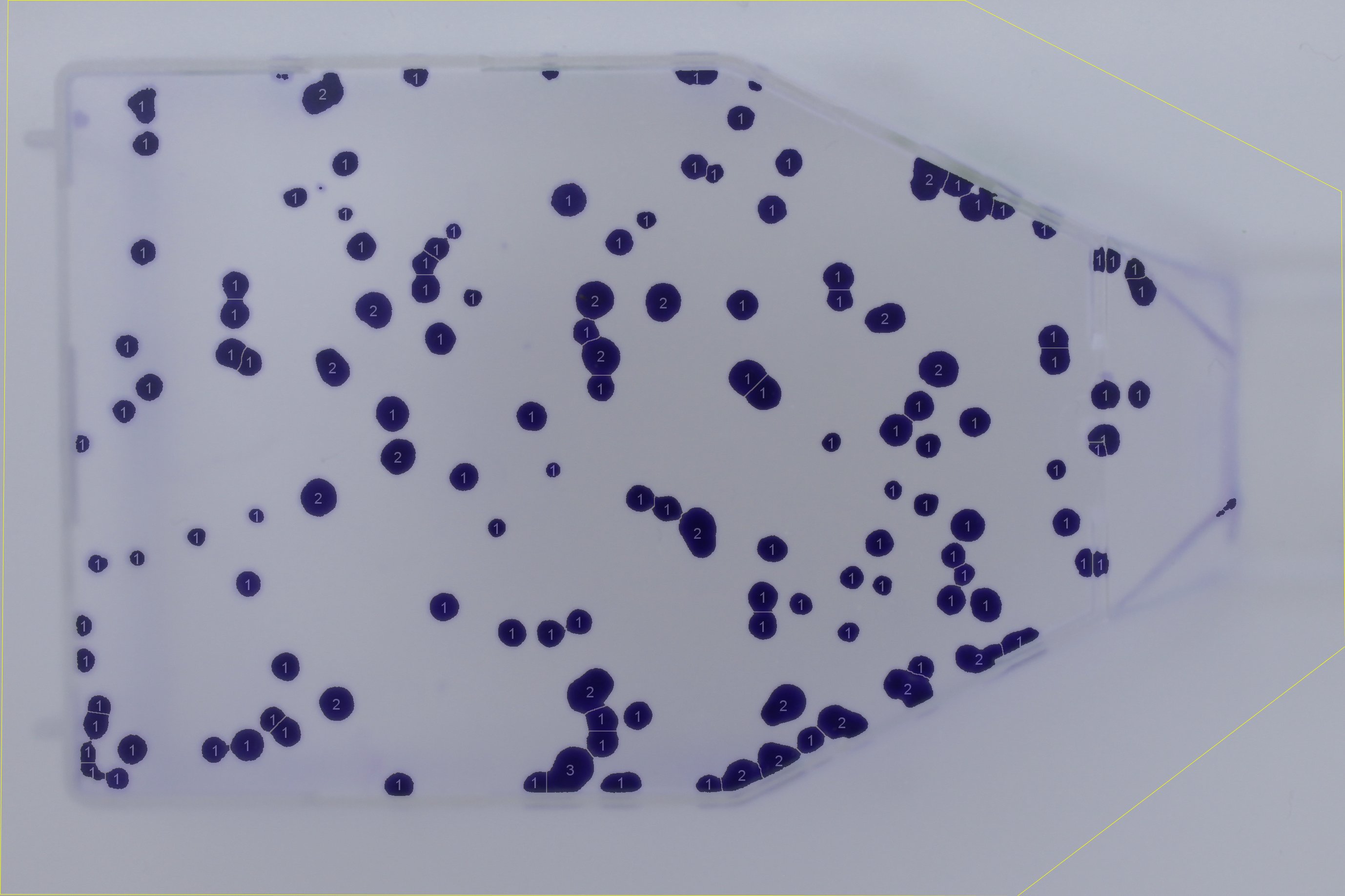

Supplement: S2 Datasets — It also contains a text file where results achieved by automated (CoCoNut, CAI, AutoCellSeg, and OpenCFU) and manual methods are summarized. (ZIP) [file pone.0205823.s003.zip › 171214 V79 Flask/9 Results.jpg]

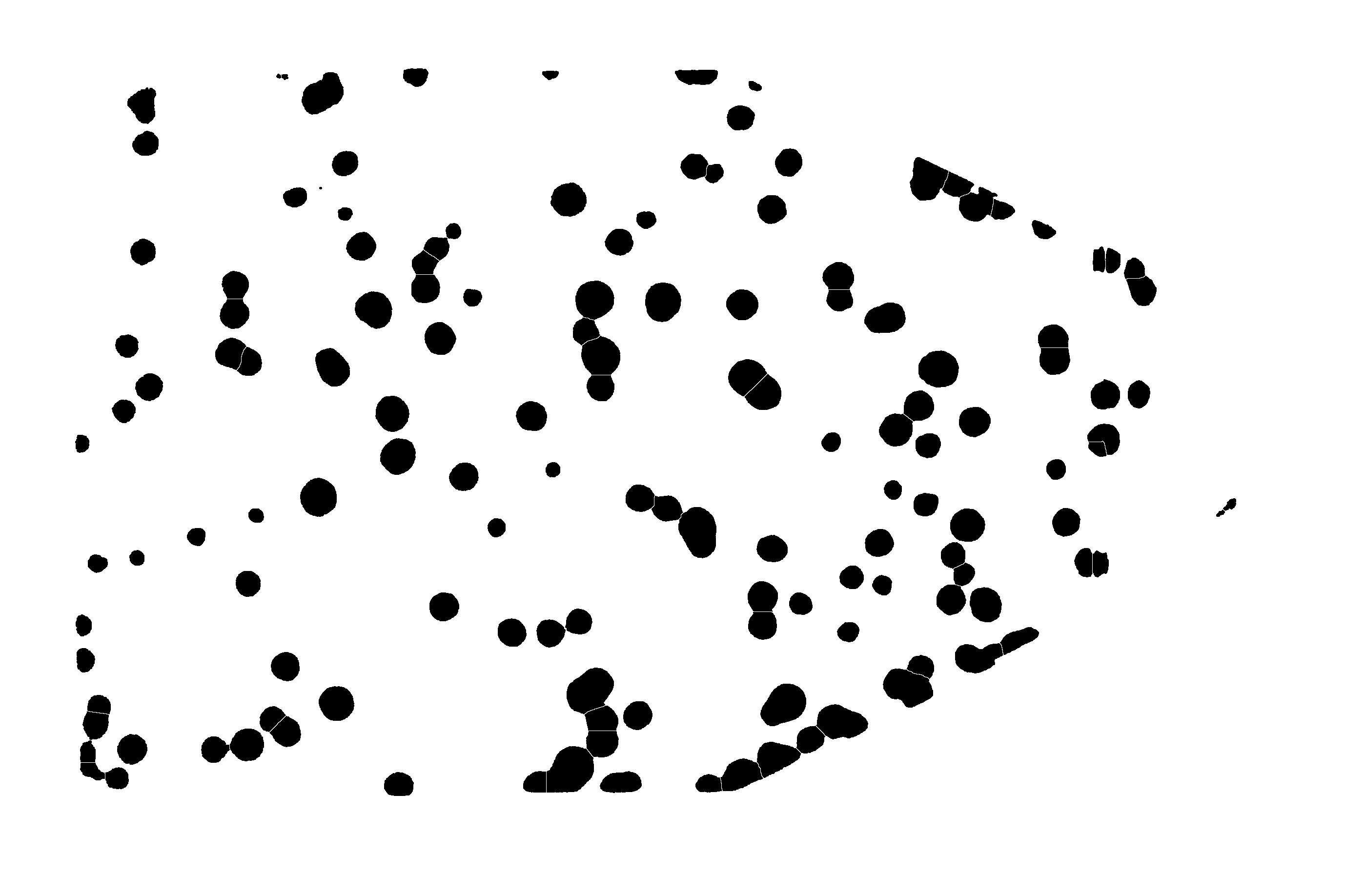

Supplement: S2 Datasets — It also contains a text file where results achieved by automated (CoCoNut, CAI, AutoCellSeg, and OpenCFU) and manual methods are summarized. (ZIP) [file pone.0205823.s003.zip › 171214 V79 Flask/9 Second counting.jpg]

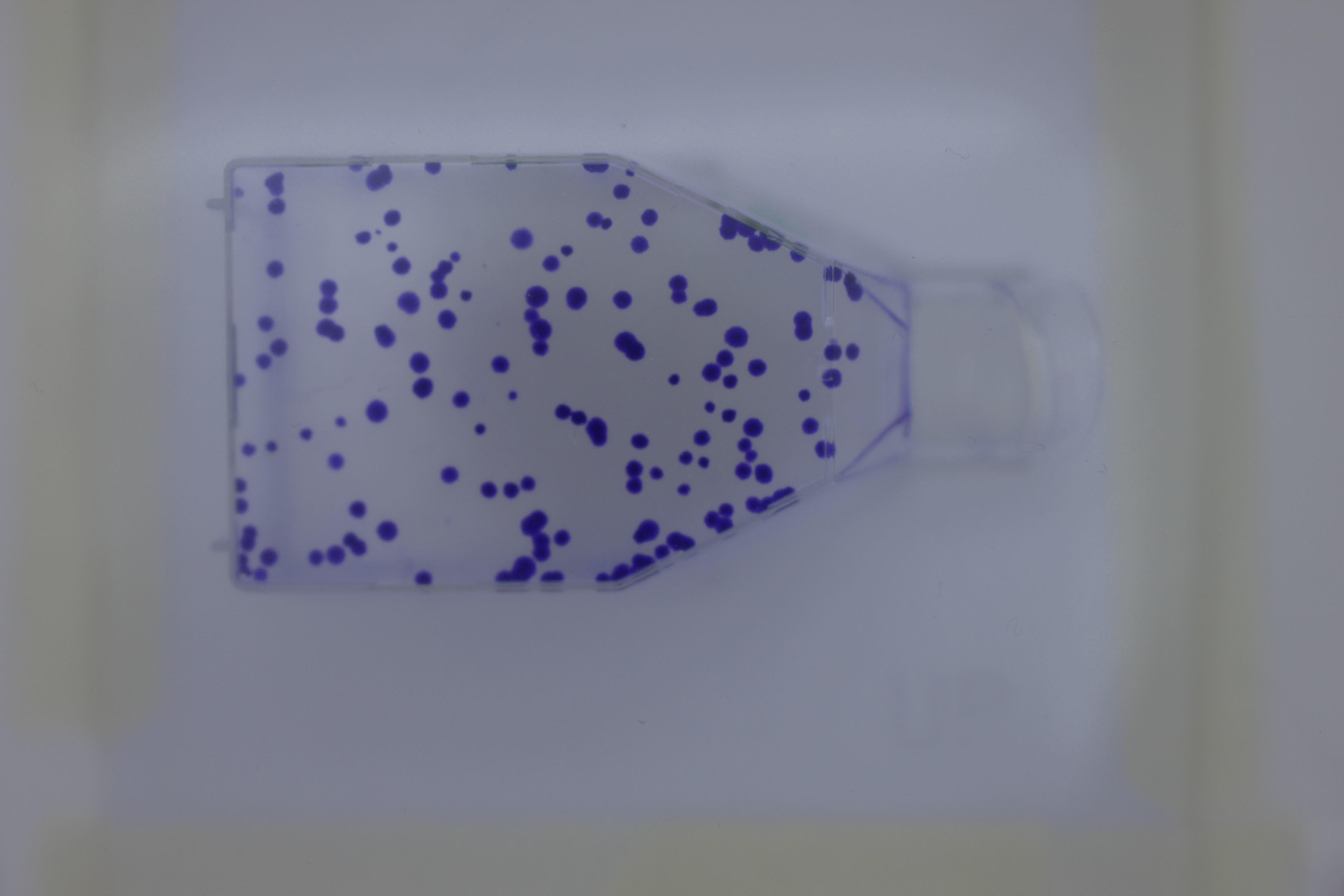

Supplement: S2 Datasets — It also contains a text file where results achieved by automated (CoCoNut, CAI, AutoCellSeg, and OpenCFU) and manual methods are summarized. (ZIP) [file pone.0205823.s003.zip › 171214 V79 Flask/9.JPG]

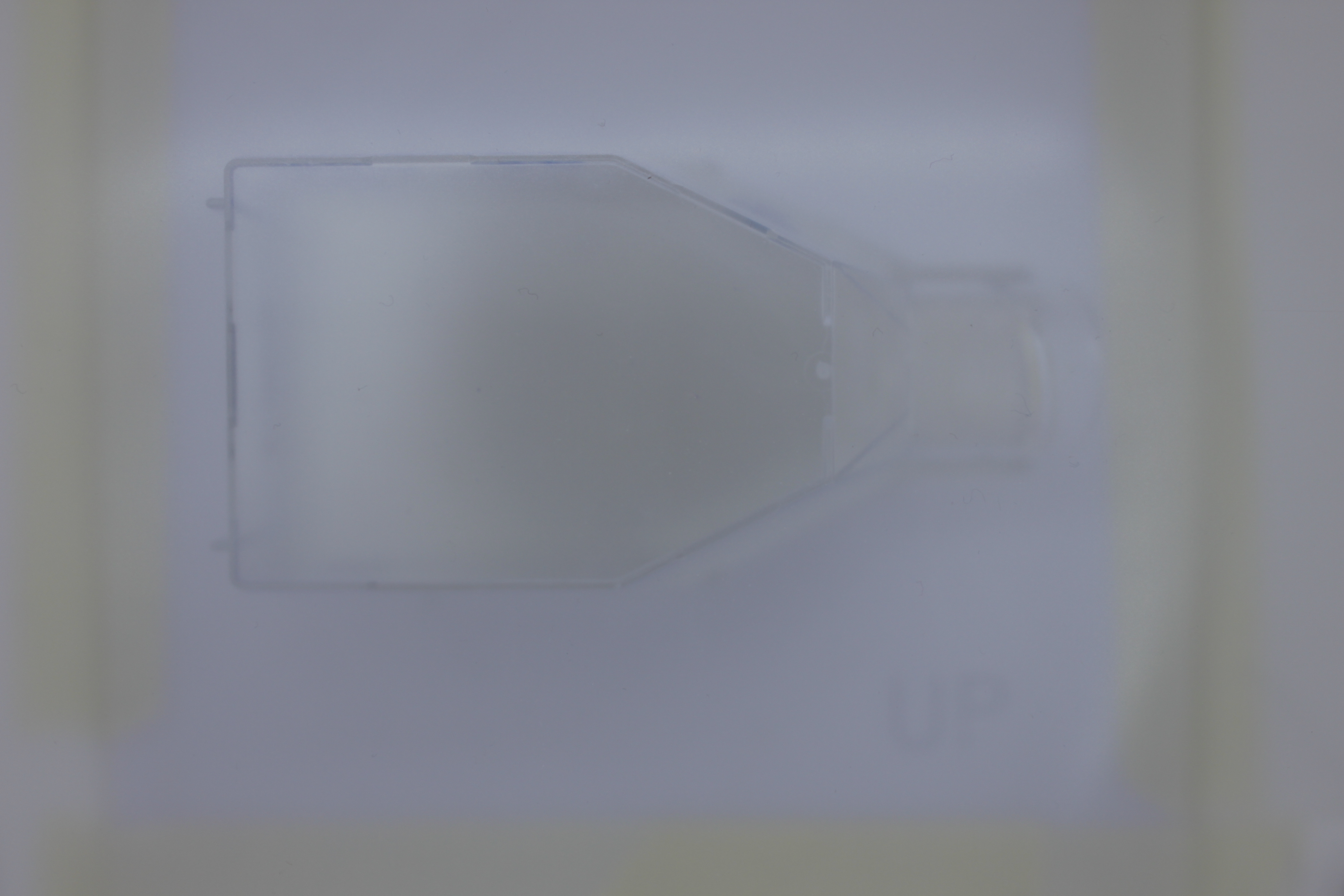

Supplement: S2 Datasets — It also contains a text file where results achieved by automated (CoCoNut, CAI, AutoCellSeg, and OpenCFU) and manual methods are summarized. (ZIP) [file pone.0205823.s003.zip › 171214 V79 Flask/background.JPG]

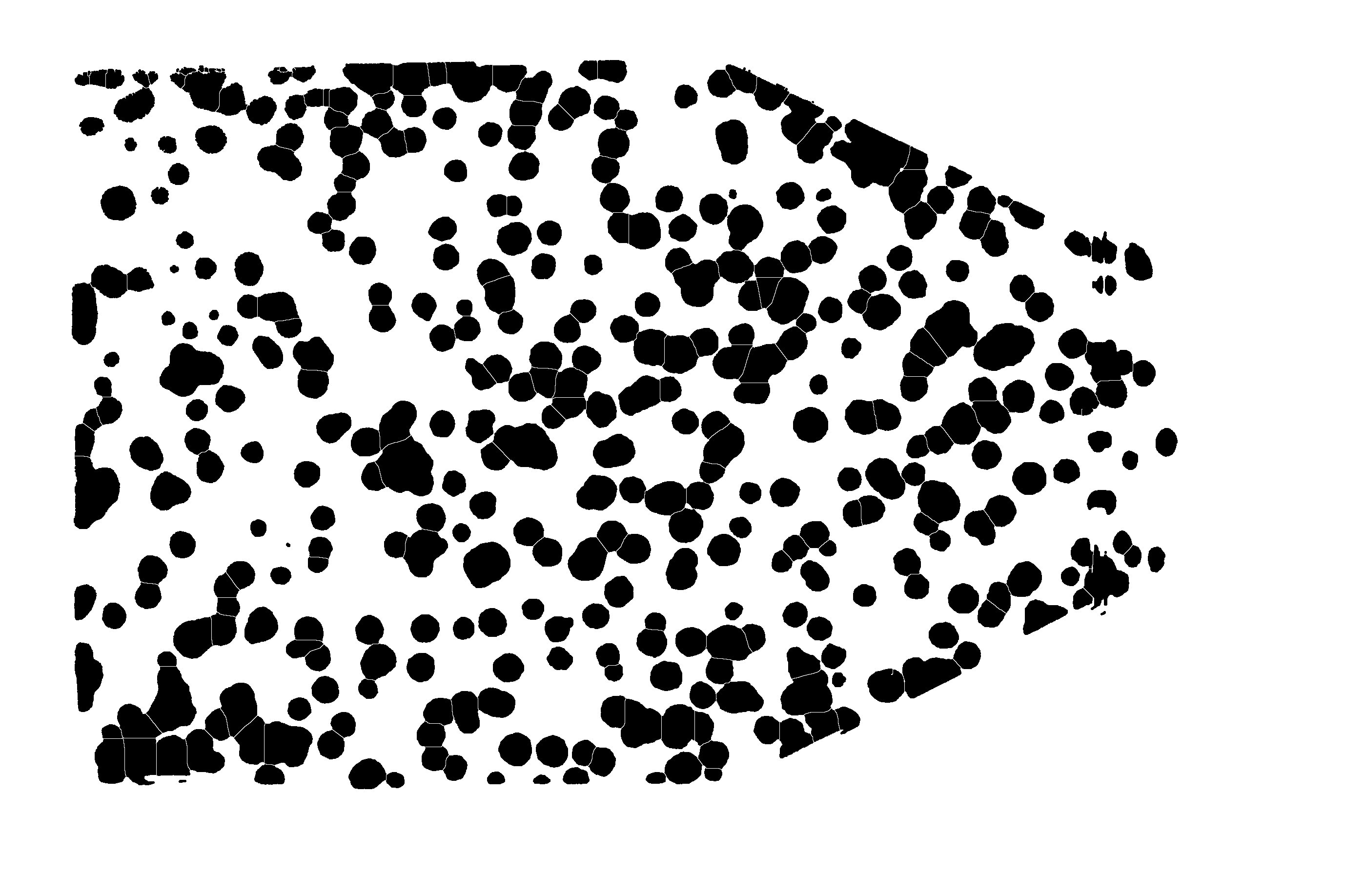

Supplement: S2 Datasets — It also contains a text file where results achieved by automated (CoCoNut, CAI, AutoCellSeg, and OpenCFU) and manual methods are summarized. (ZIP) [file pone.0205823.s003.zip › 171214 V79 Flask/binary13.jpg]

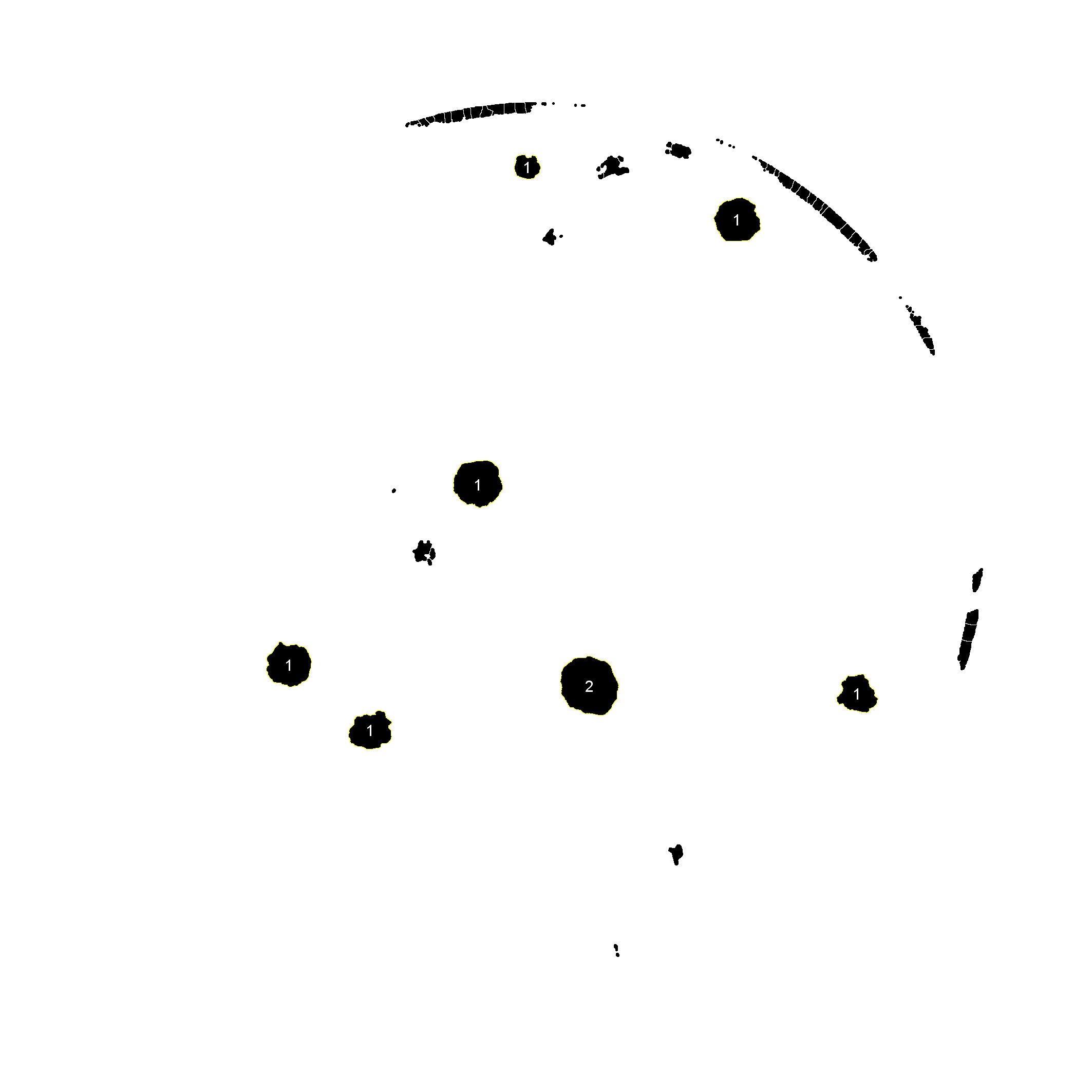

Supplement: S3 Datasets — It also contains a text file where results achieved by automated (CoCoNut, CAI, AutoCellSeg, and OpenCFU) and manual methods are summarized. (ZIP) [file pone.0205823.s004.zip › 180501 HeLa Dish/1 First counting.jpg]

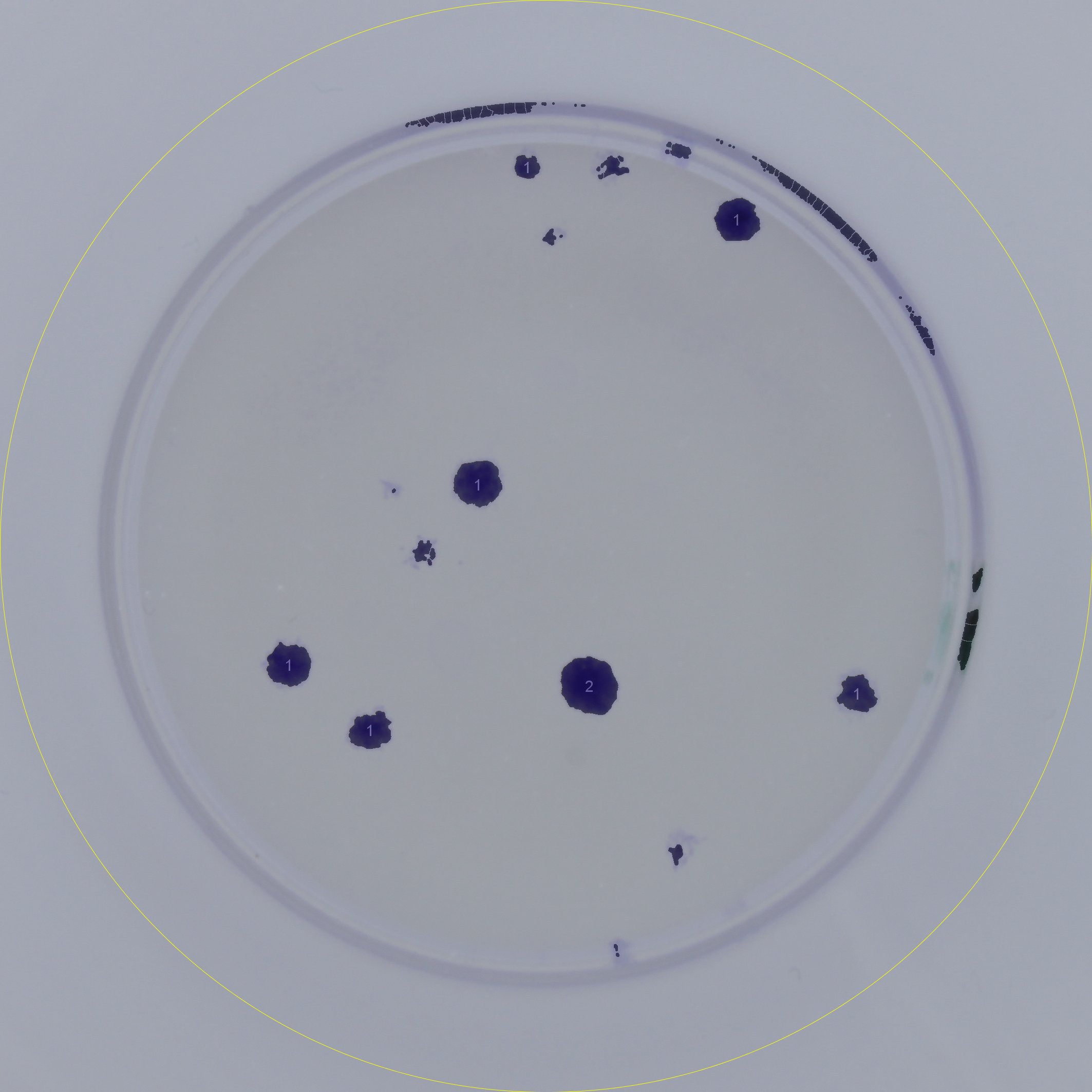

Supplement: S3 Datasets — It also contains a text file where results achieved by automated (CoCoNut, CAI, AutoCellSeg, and OpenCFU) and manual methods are summarized. (ZIP) [file pone.0205823.s004.zip › 180501 HeLa Dish/1 Results.jpg]

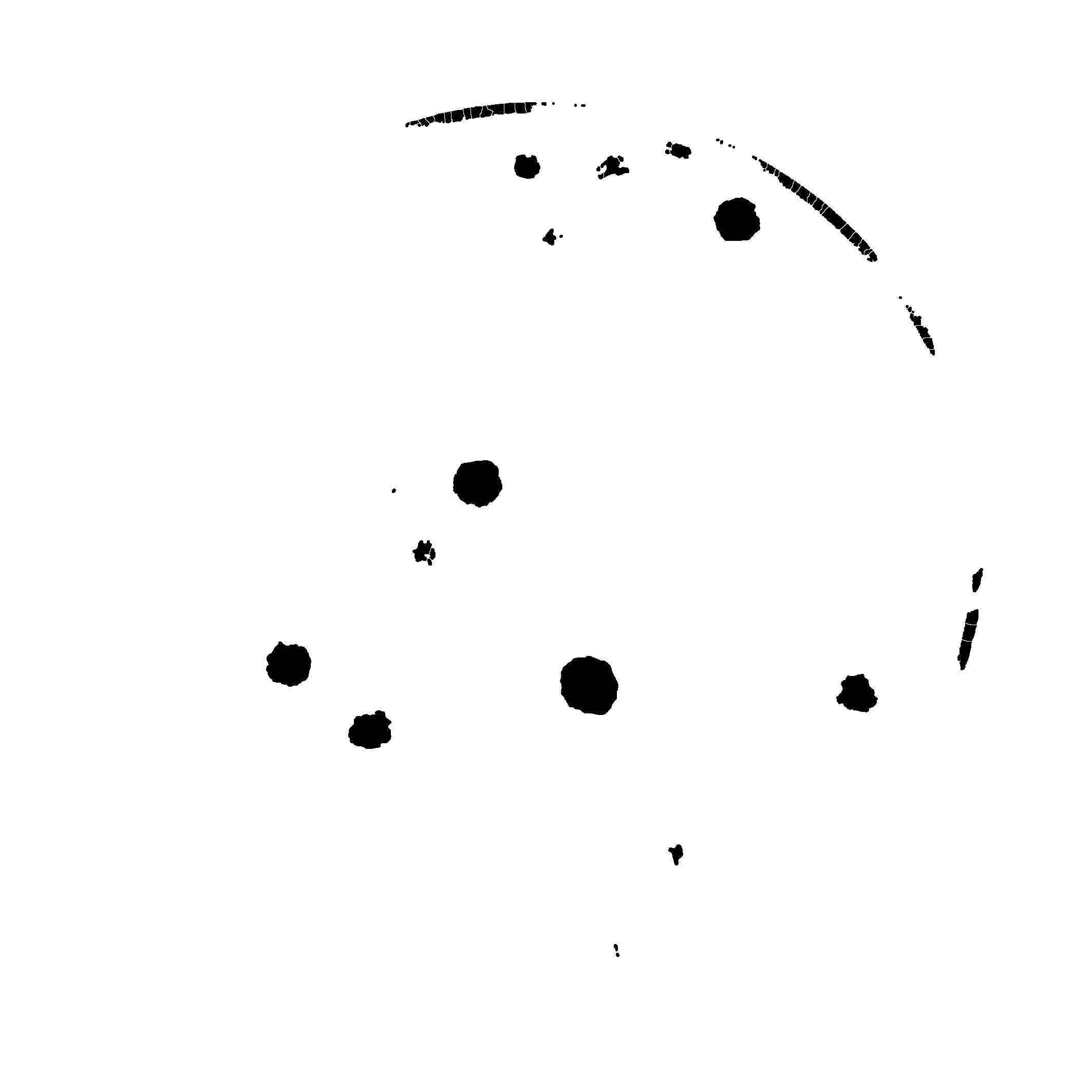

Supplement: S3 Datasets — It also contains a text file where results achieved by automated (CoCoNut, CAI, AutoCellSeg, and OpenCFU) and manual methods are summarized. (ZIP) [file pone.0205823.s004.zip › 180501 HeLa Dish/1 Second counting.jpg]

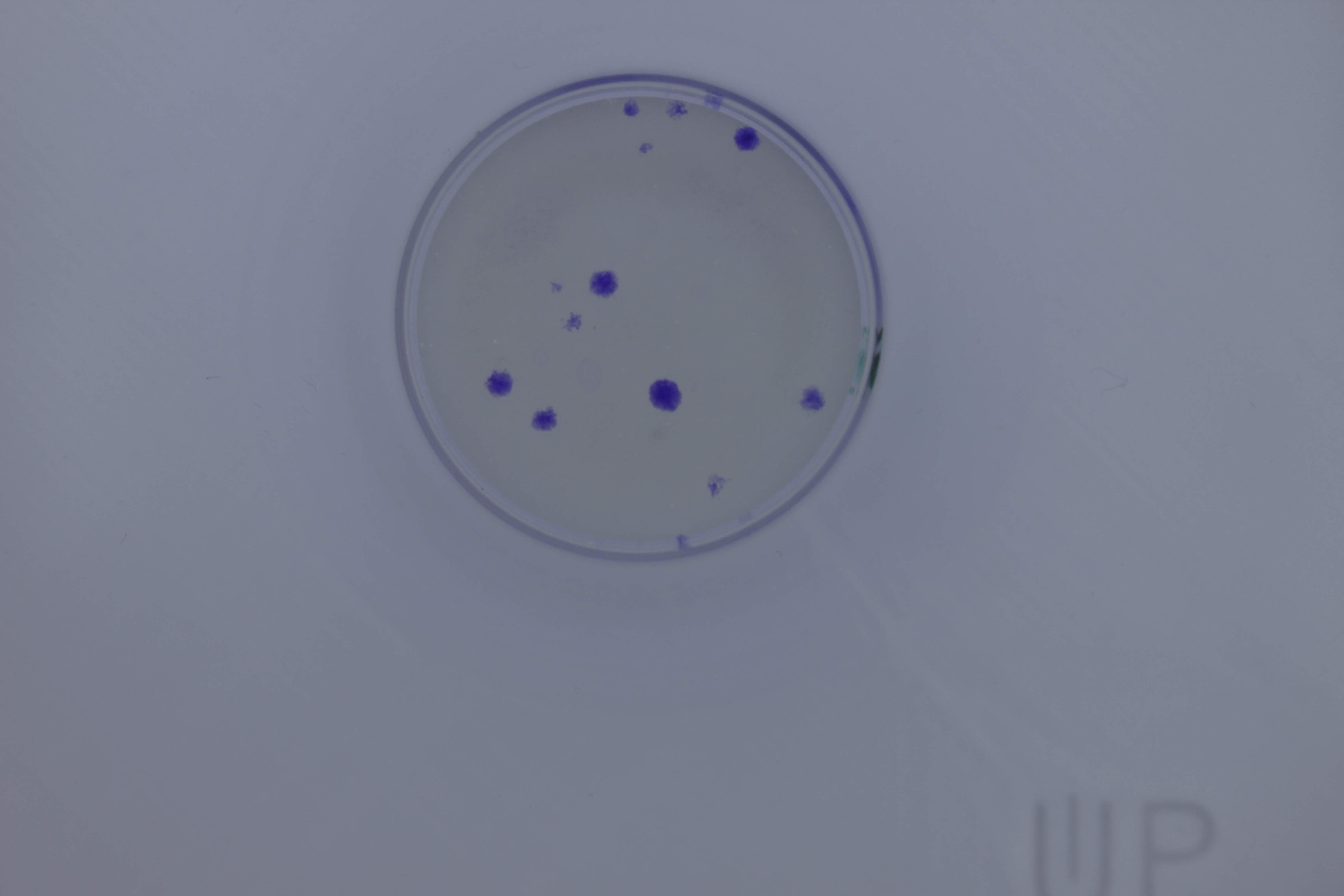

Supplement: S3 Datasets — It also contains a text file where results achieved by automated (CoCoNut, CAI, AutoCellSeg, and OpenCFU) and manual methods are summarized. (ZIP) [file pone.0205823.s004.zip › 180501 HeLa Dish/1.JPG]

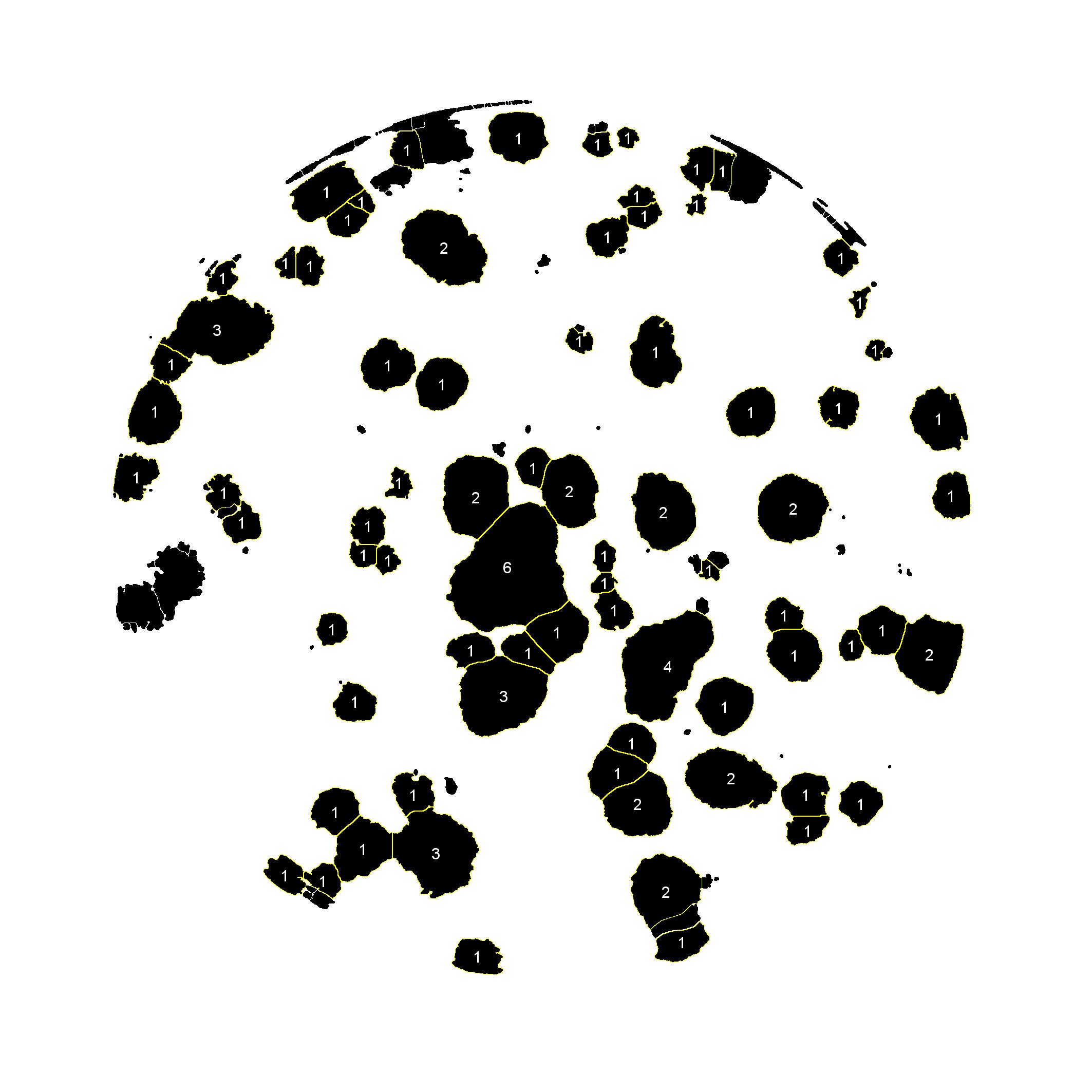

Supplement: S3 Datasets — It also contains a text file where results achieved by automated (CoCoNut, CAI, AutoCellSeg, and OpenCFU) and manual methods are summarized. (ZIP) [file pone.0205823.s004.zip › 180501 HeLa Dish/10 First counting.jpg]

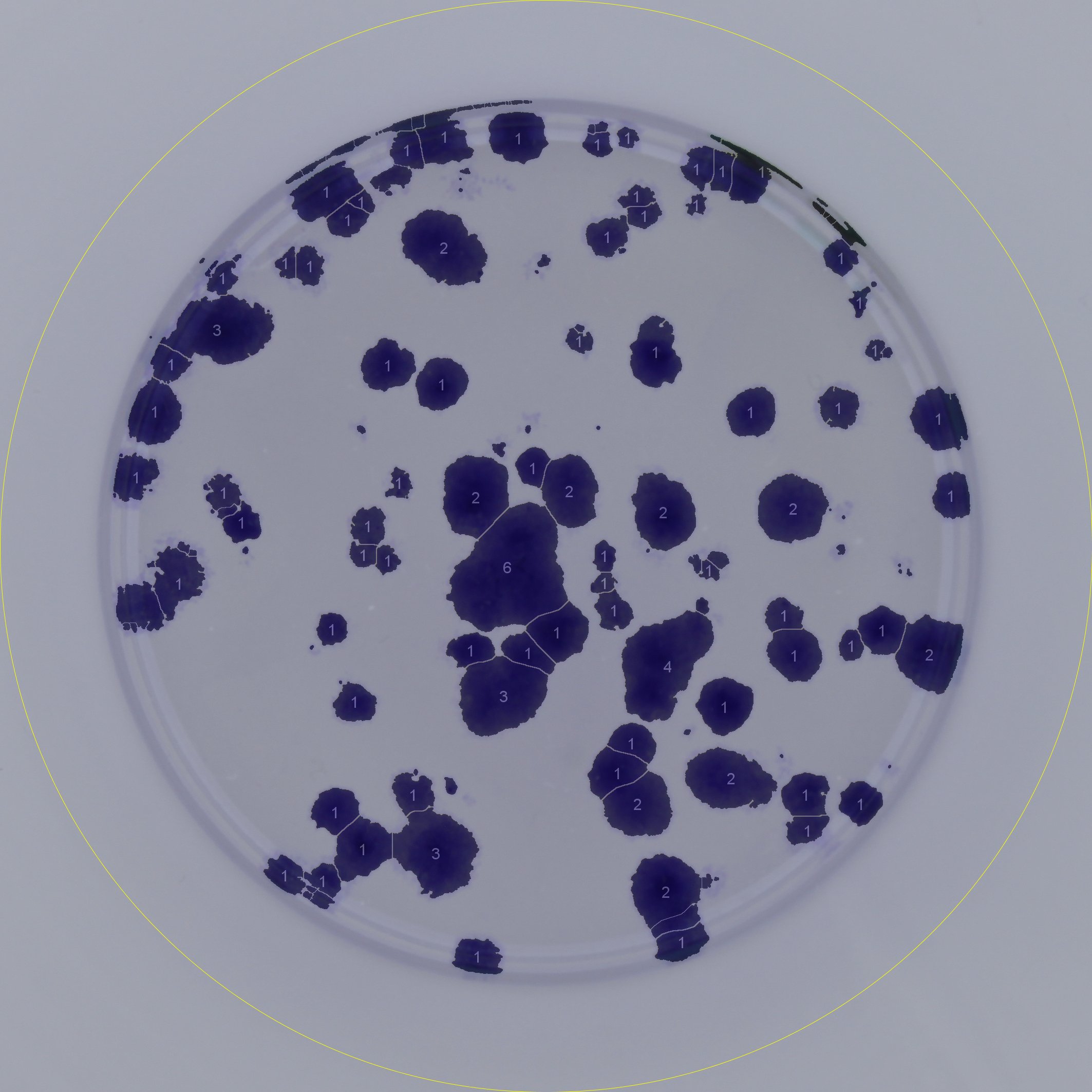

Supplement: S3 Datasets — It also contains a text file where results achieved by automated (CoCoNut, CAI, AutoCellSeg, and OpenCFU) and manual methods are summarized. (ZIP) [file pone.0205823.s004.zip › 180501 HeLa Dish/10 Results.jpg]

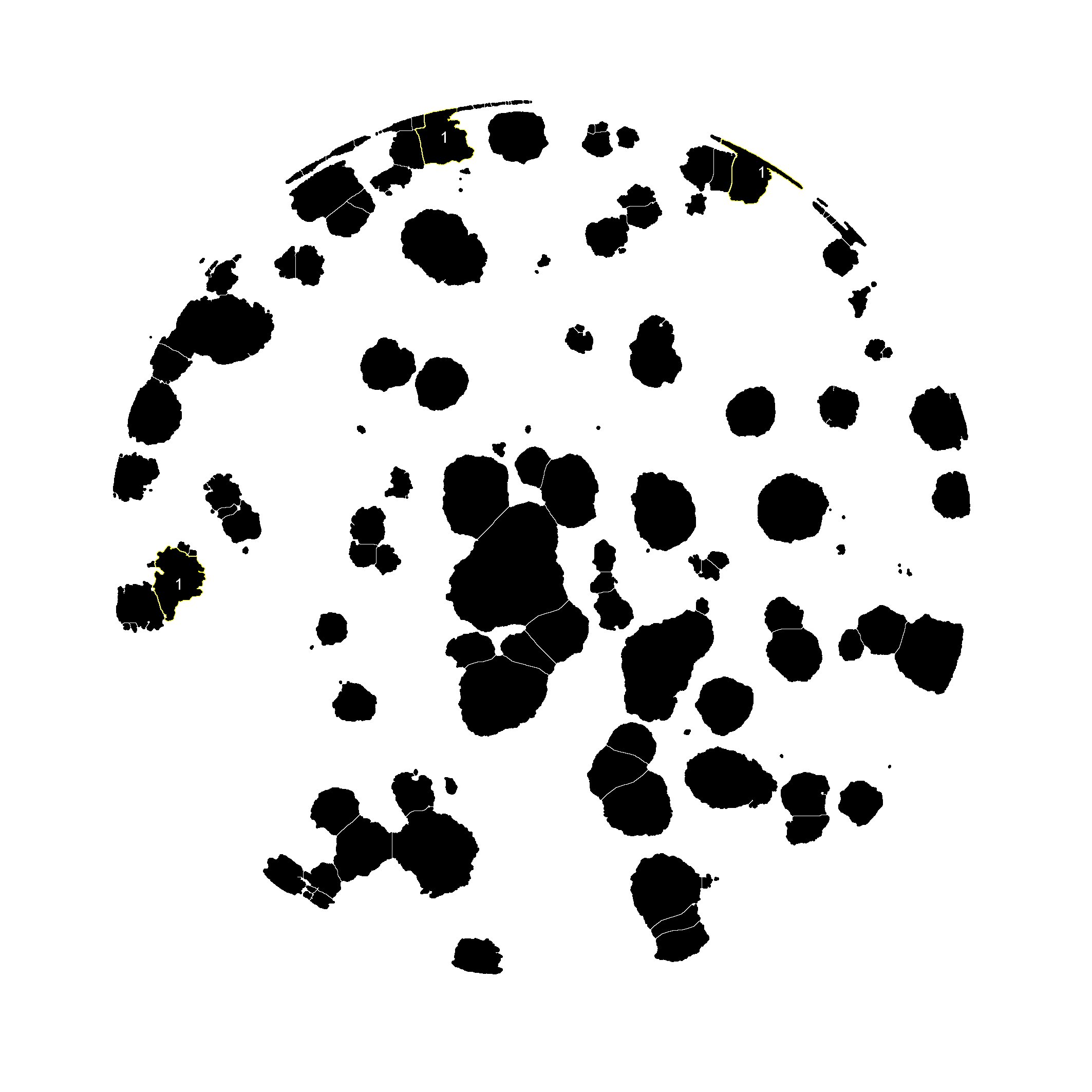

Supplement: S3 Datasets — It also contains a text file where results achieved by automated (CoCoNut, CAI, AutoCellSeg, and OpenCFU) and manual methods are summarized. (ZIP) [file pone.0205823.s004.zip › 180501 HeLa Dish/10 Second counting.jpg]

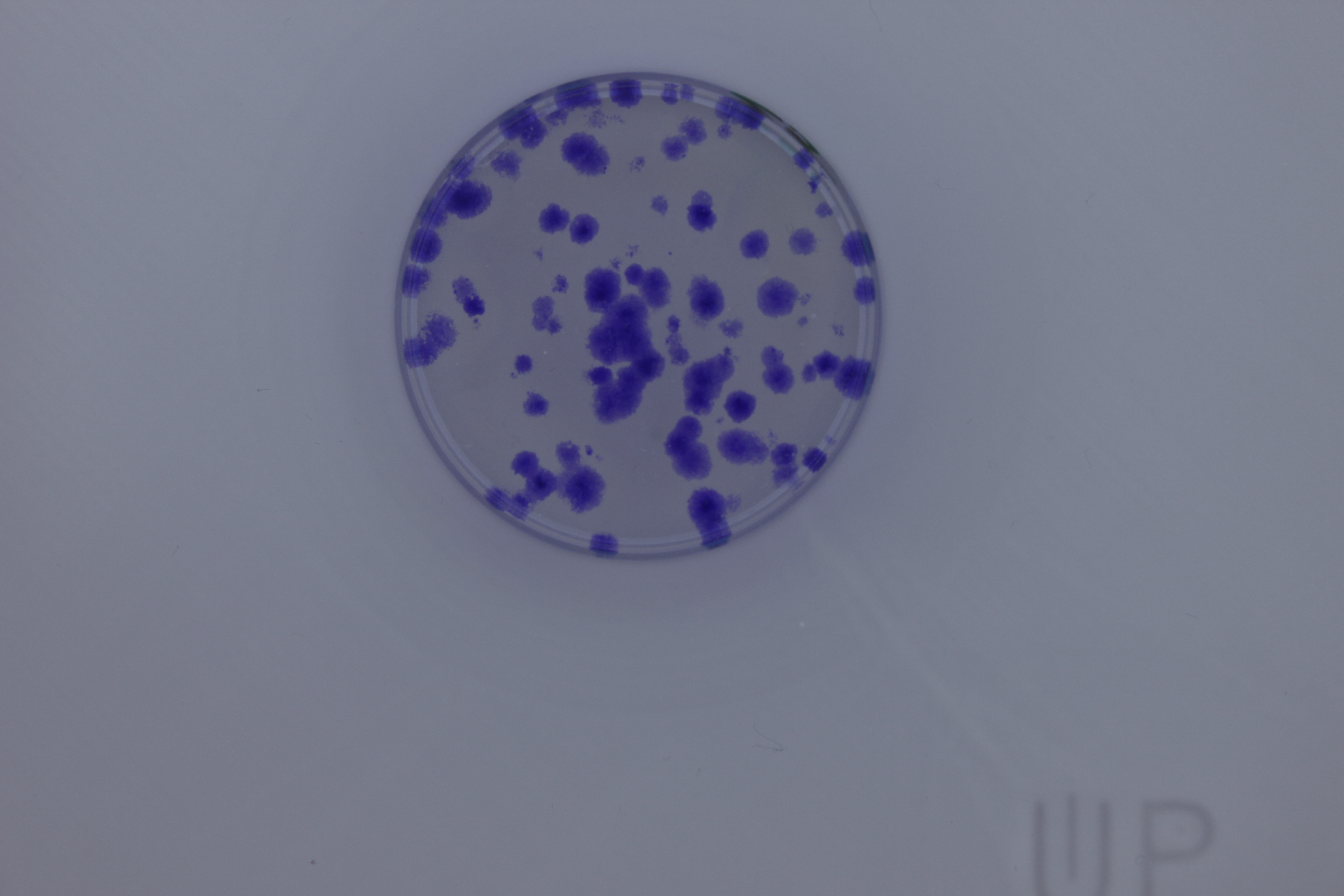

Supplement: S3 Datasets — It also contains a text file where results achieved by automated (CoCoNut, CAI, AutoCellSeg, and OpenCFU) and manual methods are summarized. (ZIP) [file pone.0205823.s004.zip › 180501 HeLa Dish/10.JPG]

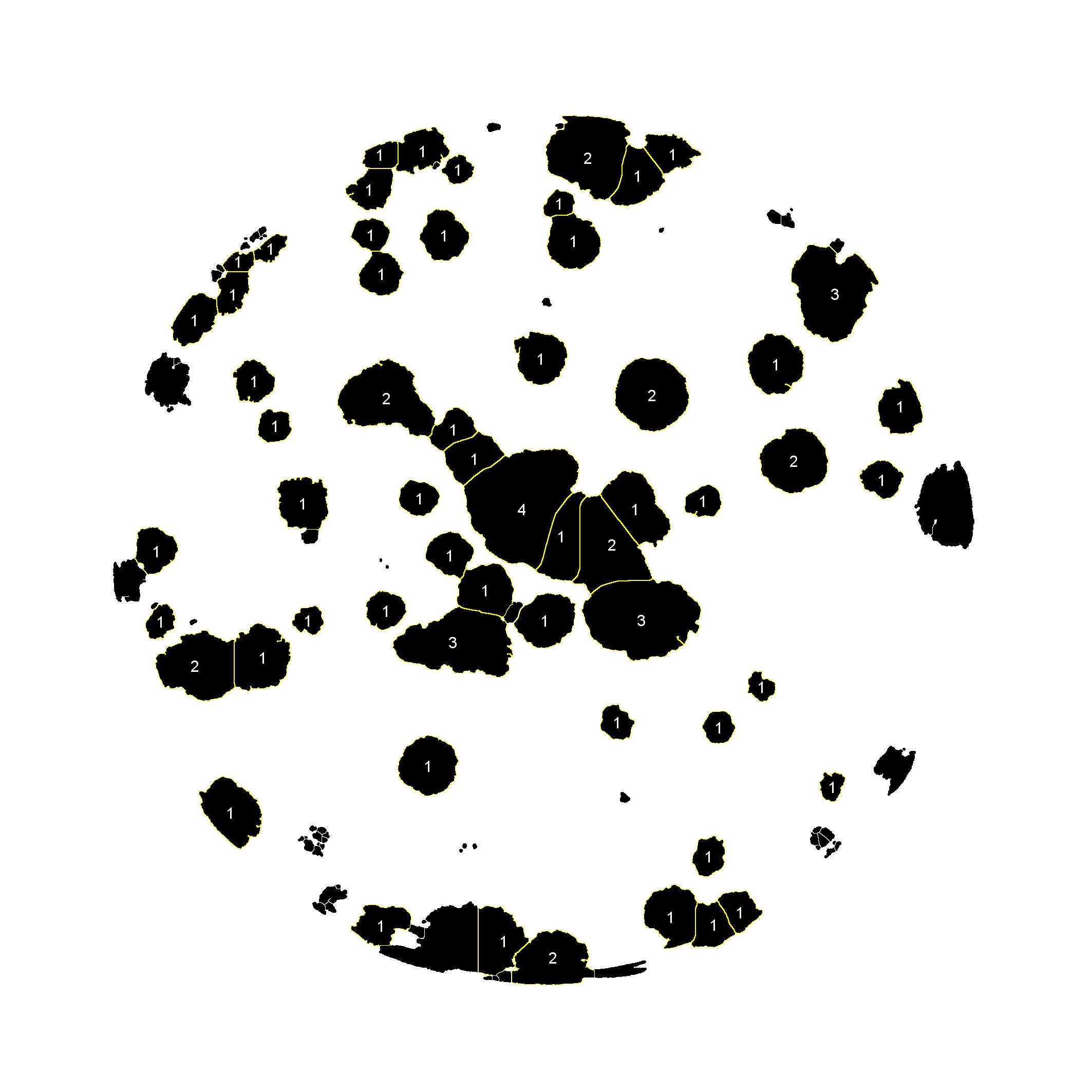

Supplement: S3 Datasets — It also contains a text file where results achieved by automated (CoCoNut, CAI, AutoCellSeg, and OpenCFU) and manual methods are summarized. (ZIP) [file pone.0205823.s004.zip › 180501 HeLa Dish/11 First counting.jpg]

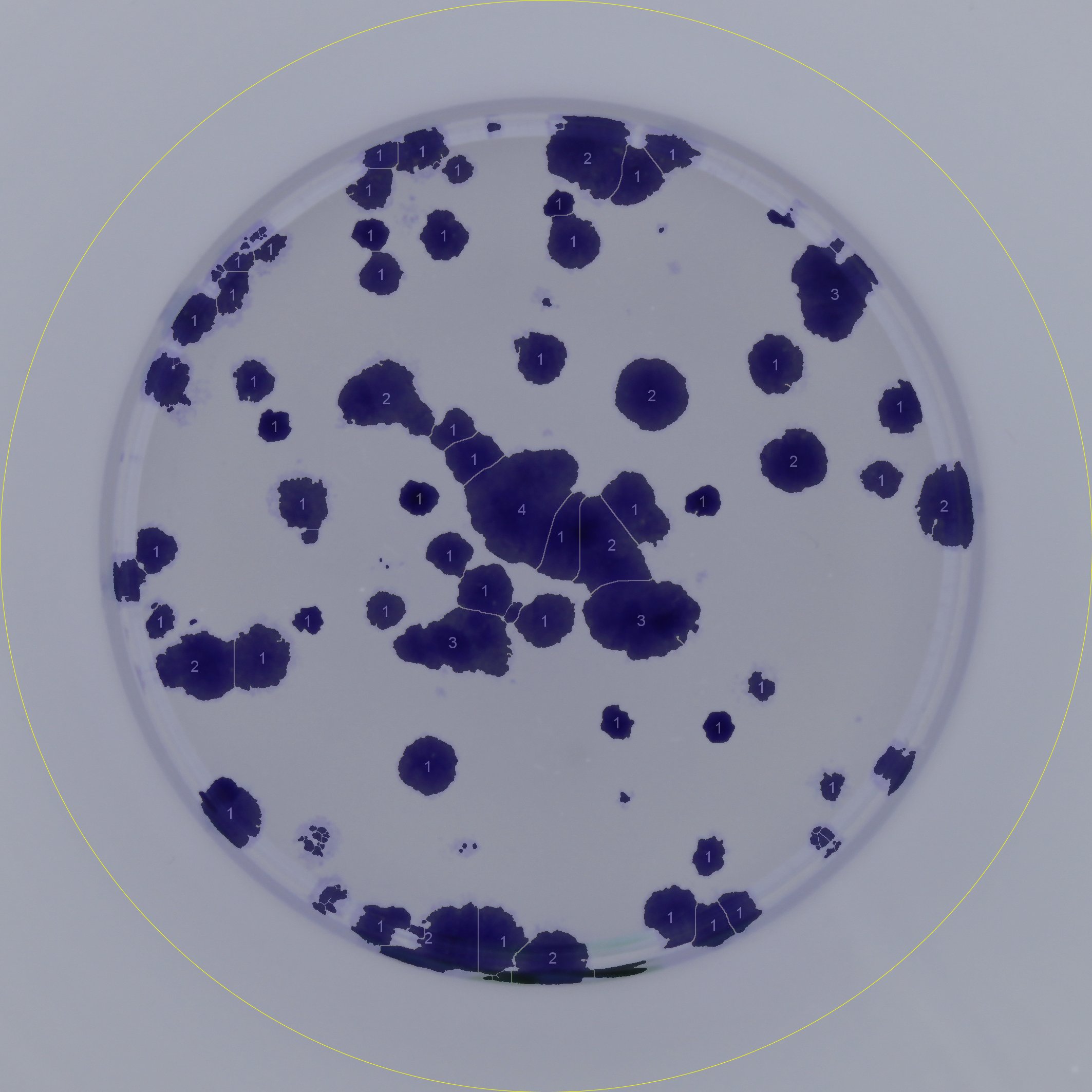

Supplement: S3 Datasets — It also contains a text file where results achieved by automated (CoCoNut, CAI, AutoCellSeg, and OpenCFU) and manual methods are summarized. (ZIP) [file pone.0205823.s004.zip › 180501 HeLa Dish/11 Results.jpg]

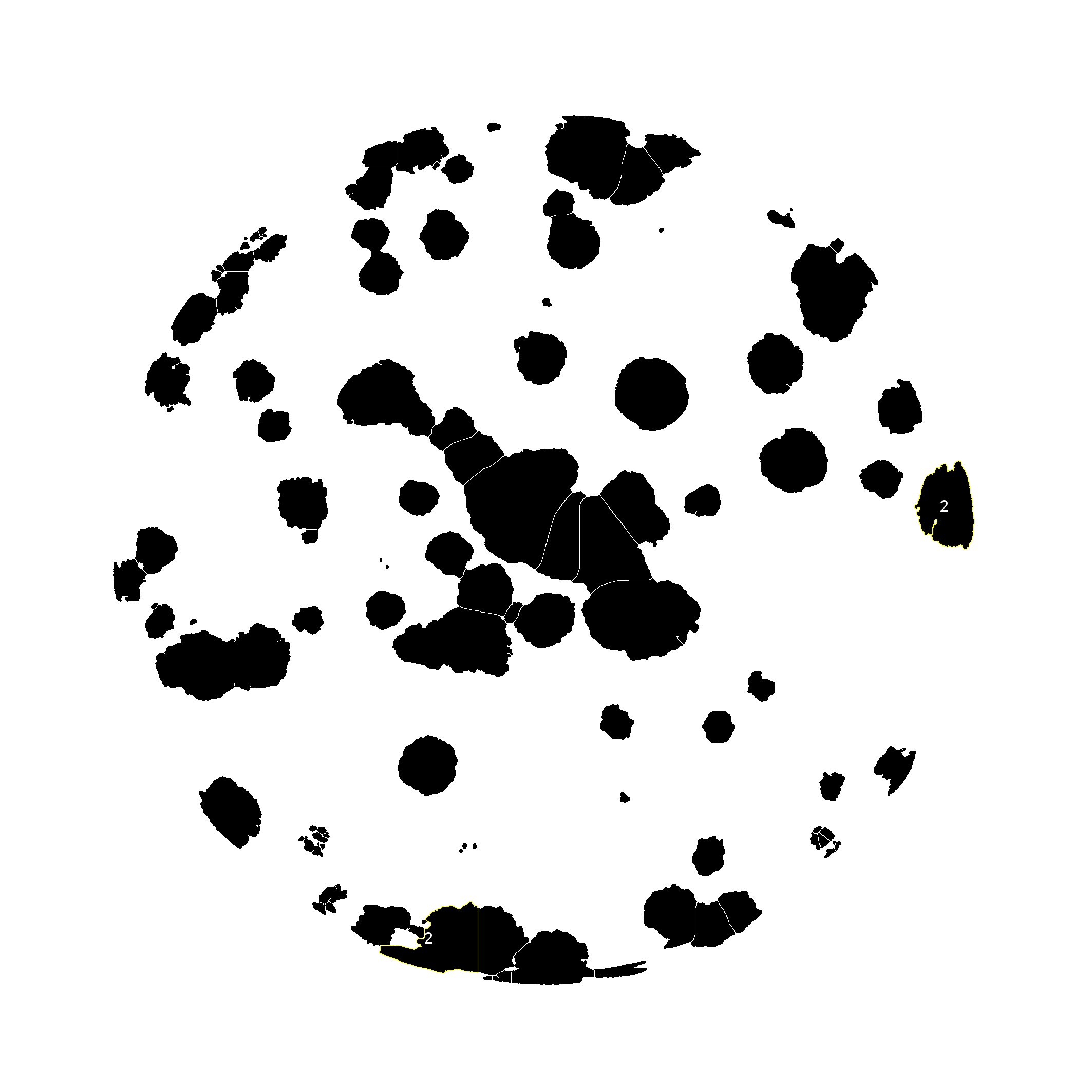

Supplement: S3 Datasets — It also contains a text file where results achieved by automated (CoCoNut, CAI, AutoCellSeg, and OpenCFU) and manual methods are summarized. (ZIP) [file pone.0205823.s004.zip › 180501 HeLa Dish/11 Second counting.jpg]

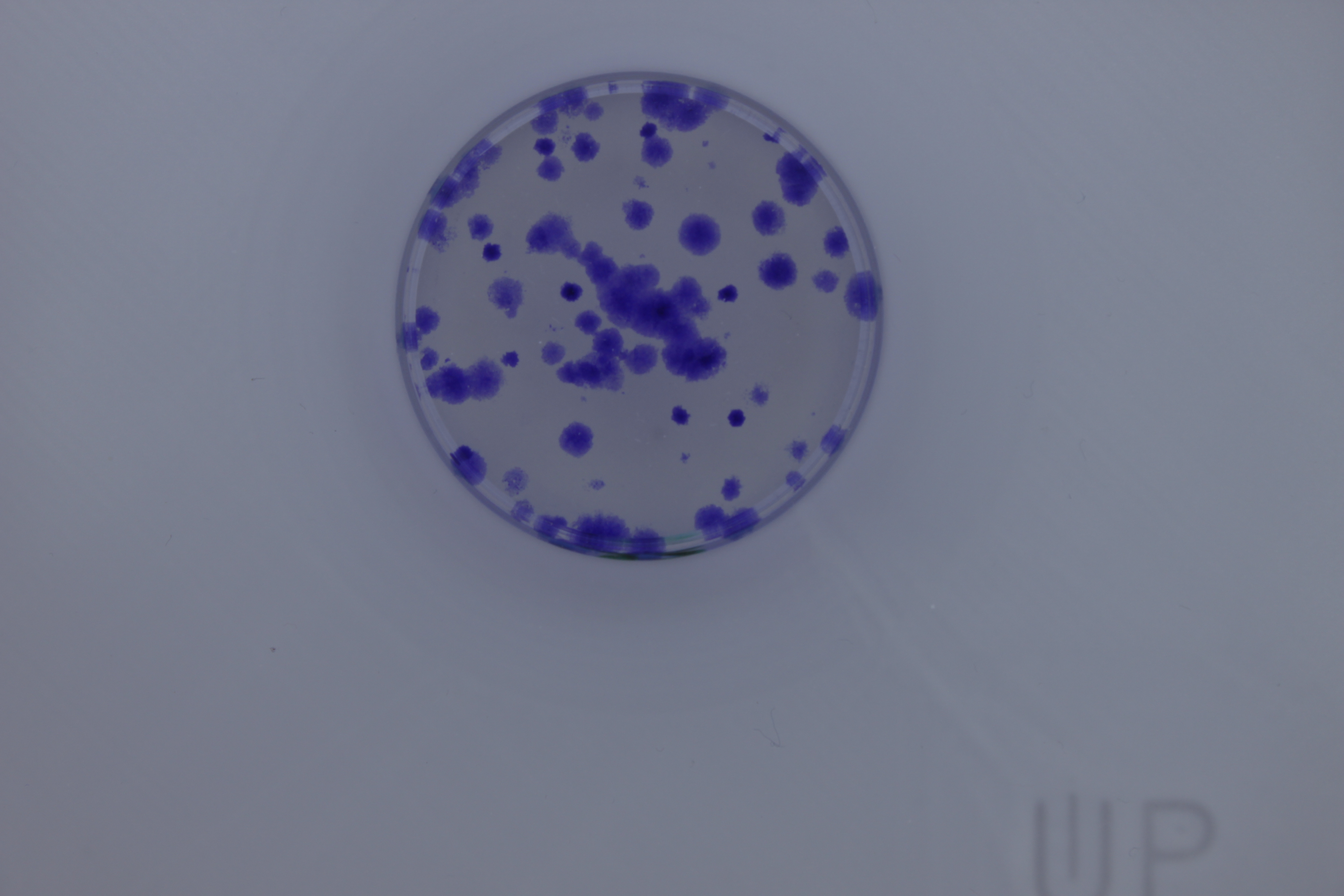

Supplement: S3 Datasets — It also contains a text file where results achieved by automated (CoCoNut, CAI, AutoCellSeg, and OpenCFU) and manual methods are summarized. (ZIP) [file pone.0205823.s004.zip › 180501 HeLa Dish/11.JPG]

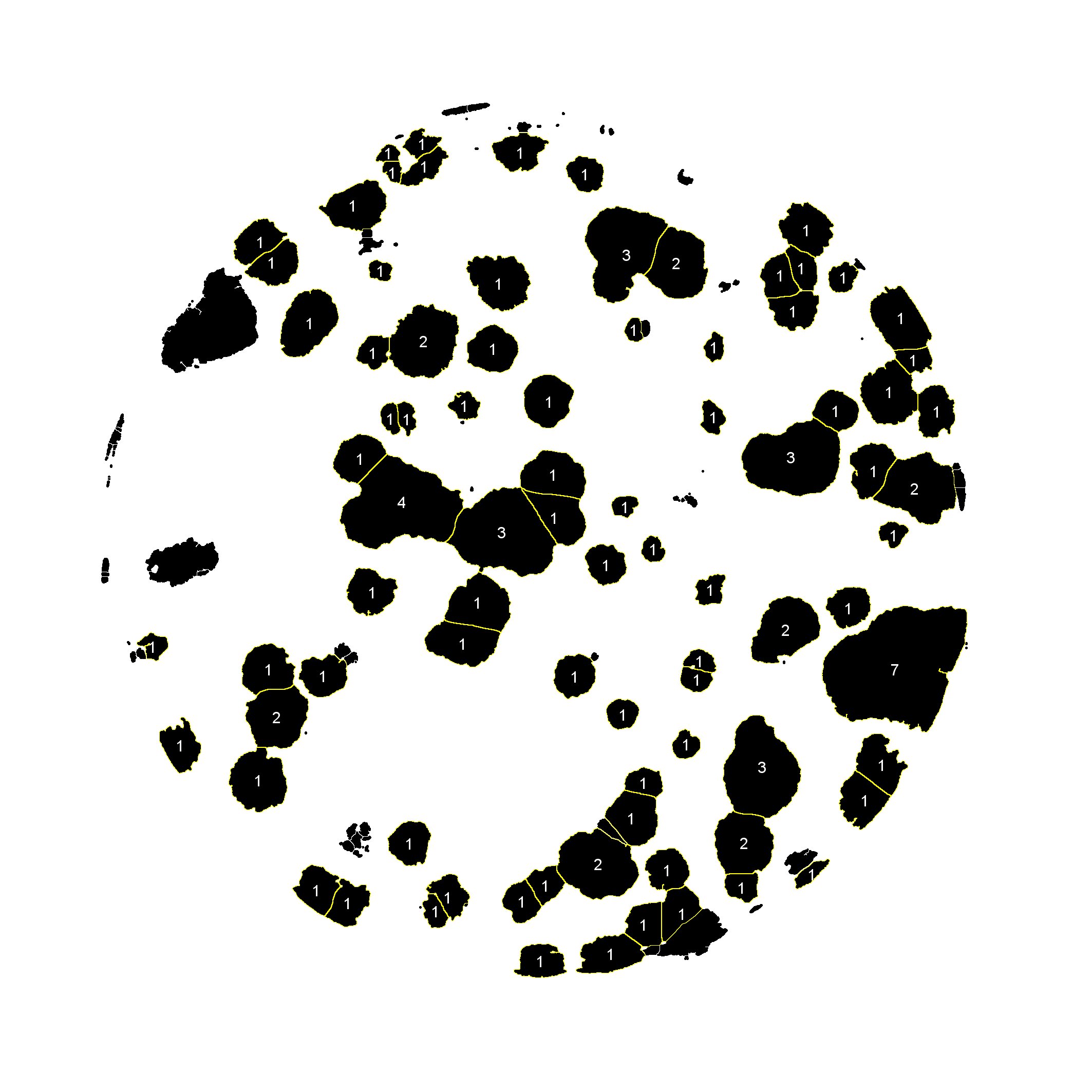

Supplement: S3 Datasets — It also contains a text file where results achieved by automated (CoCoNut, CAI, AutoCellSeg, and OpenCFU) and manual methods are summarized. (ZIP) [file pone.0205823.s004.zip › 180501 HeLa Dish/12 First counting.jpg]

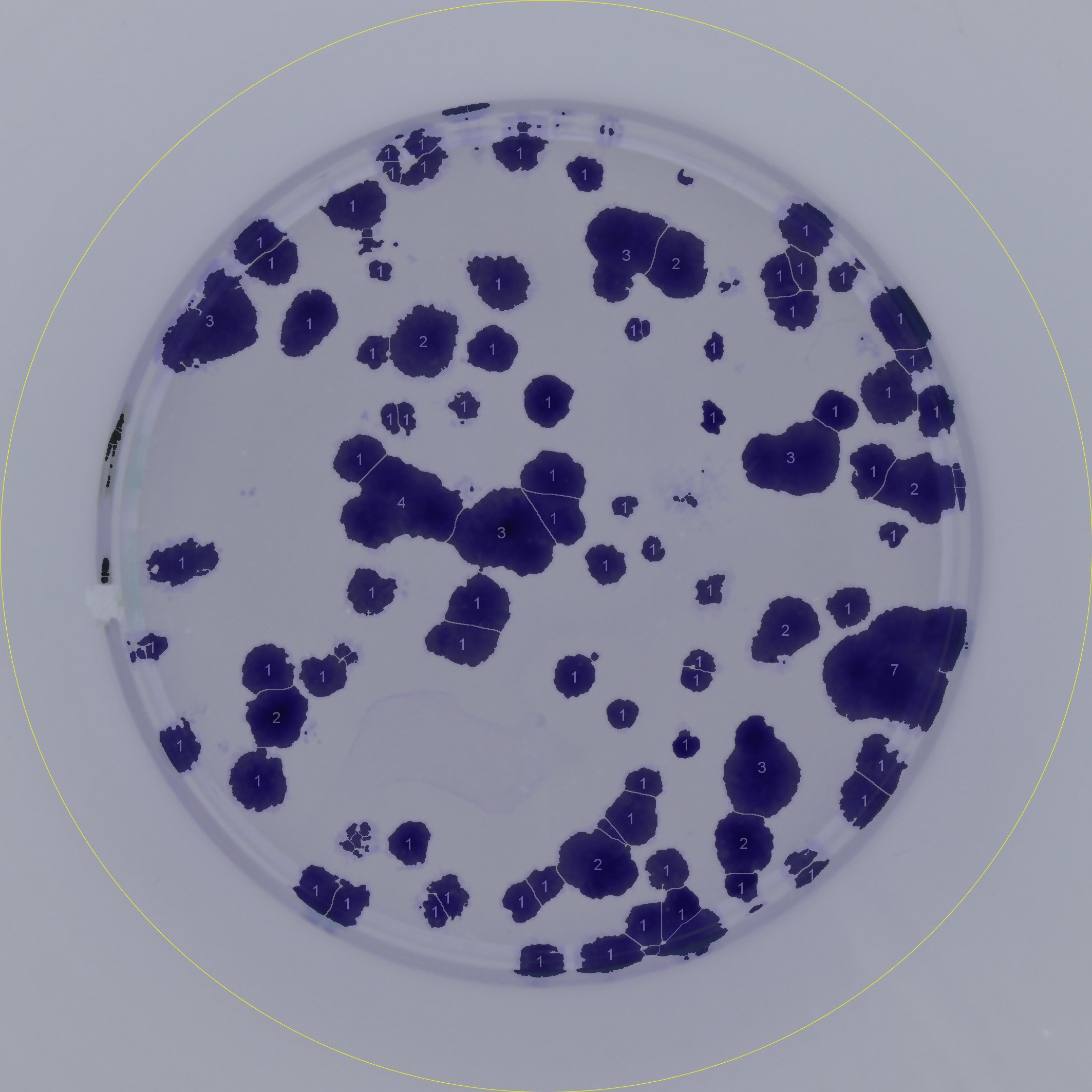

Supplement: S3 Datasets — It also contains a text file where results achieved by automated (CoCoNut, CAI, AutoCellSeg, and OpenCFU) and manual methods are summarized. (ZIP) [file pone.0205823.s004.zip › 180501 HeLa Dish/12 Results.jpg]

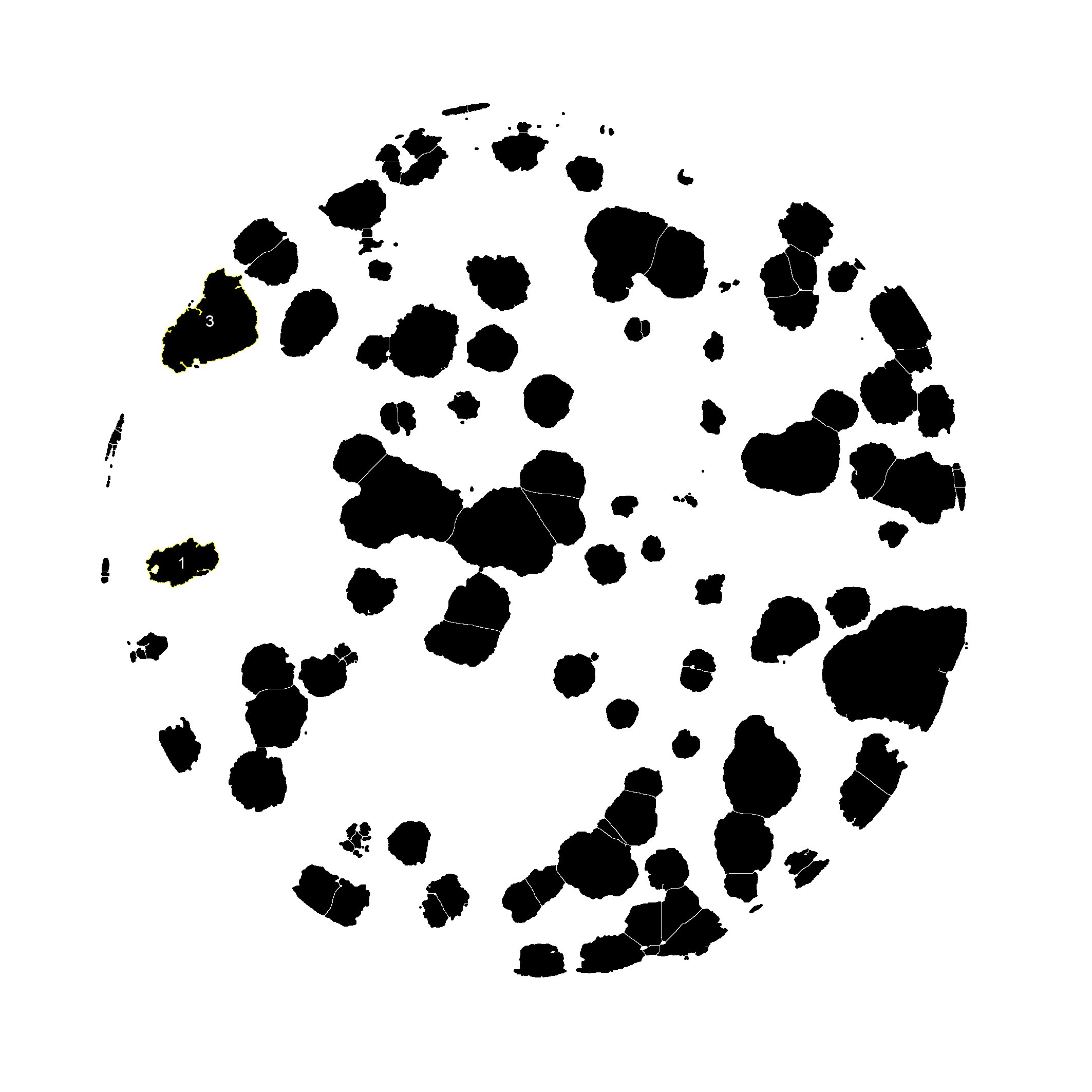

Supplement: S3 Datasets — It also contains a text file where results achieved by automated (CoCoNut, CAI, AutoCellSeg, and OpenCFU) and manual methods are summarized. (ZIP) [file pone.0205823.s004.zip › 180501 HeLa Dish/12 Second counting.jpg]

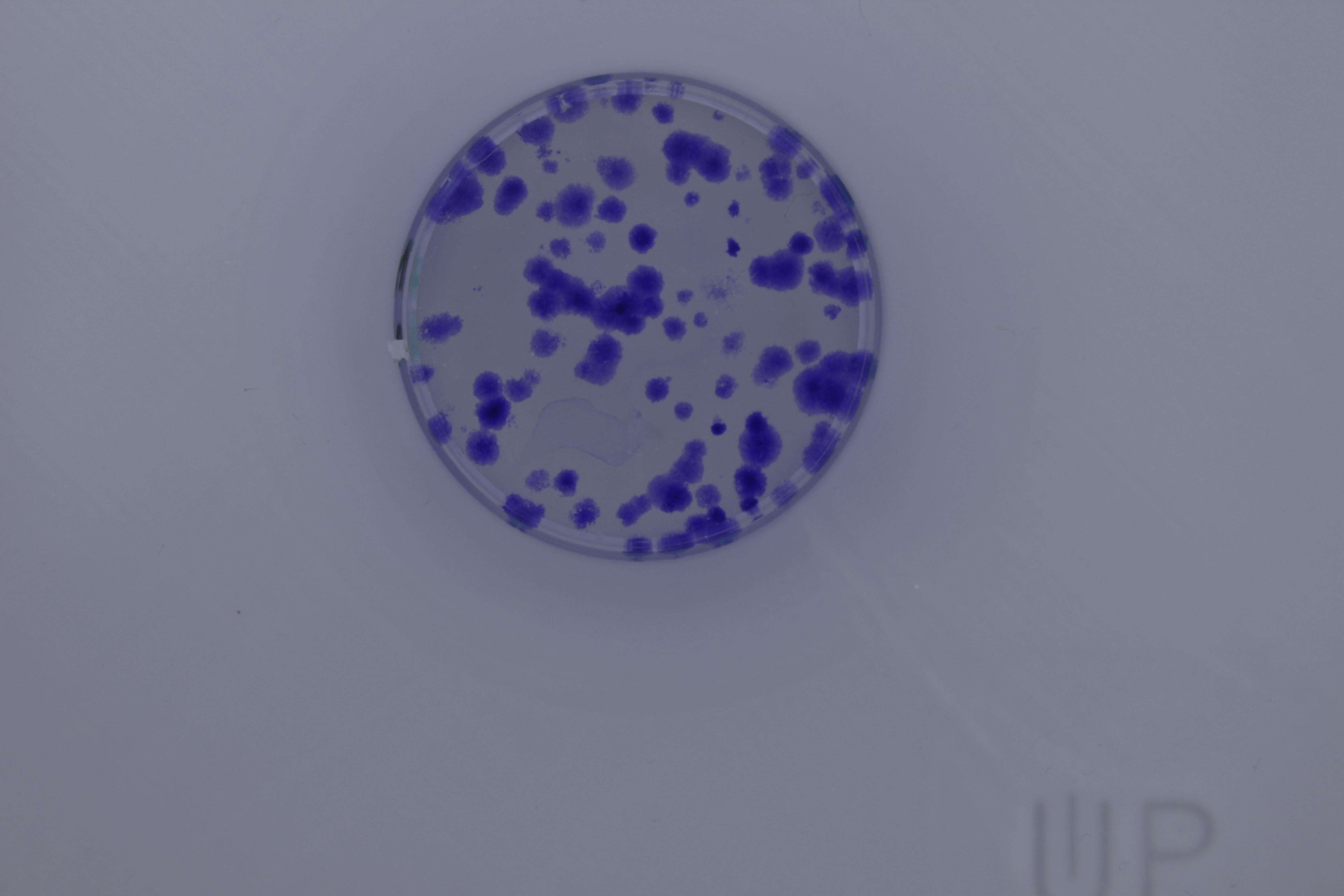

Supplement: S3 Datasets — It also contains a text file where results achieved by automated (CoCoNut, CAI, AutoCellSeg, and OpenCFU) and manual methods are summarized. (ZIP) [file pone.0205823.s004.zip › 180501 HeLa Dish/12.JPG]

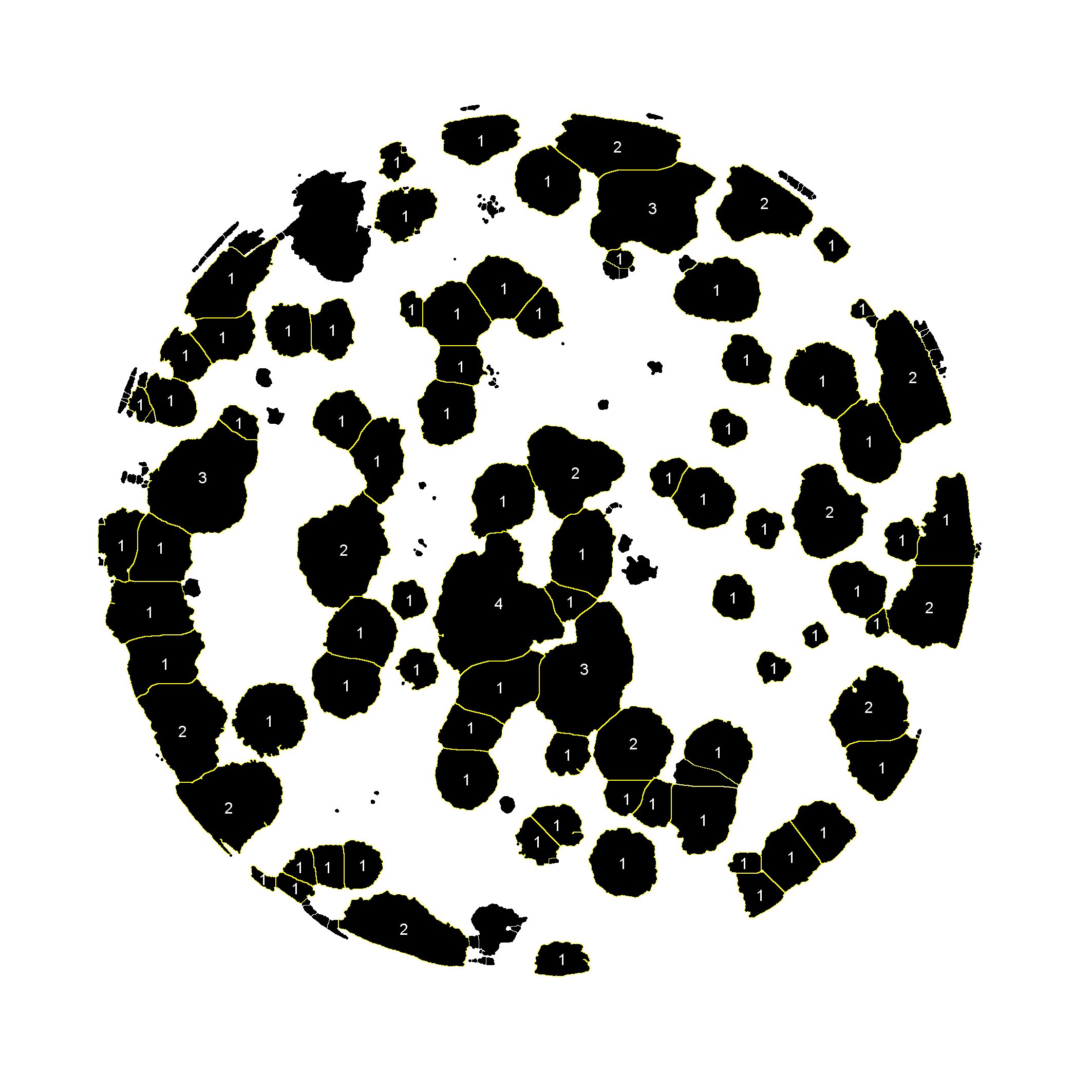

Supplement: S3 Datasets — It also contains a text file where results achieved by automated (CoCoNut, CAI, AutoCellSeg, and OpenCFU) and manual methods are summarized. (ZIP) [file pone.0205823.s004.zip › 180501 HeLa Dish/13 First counting.jpg]

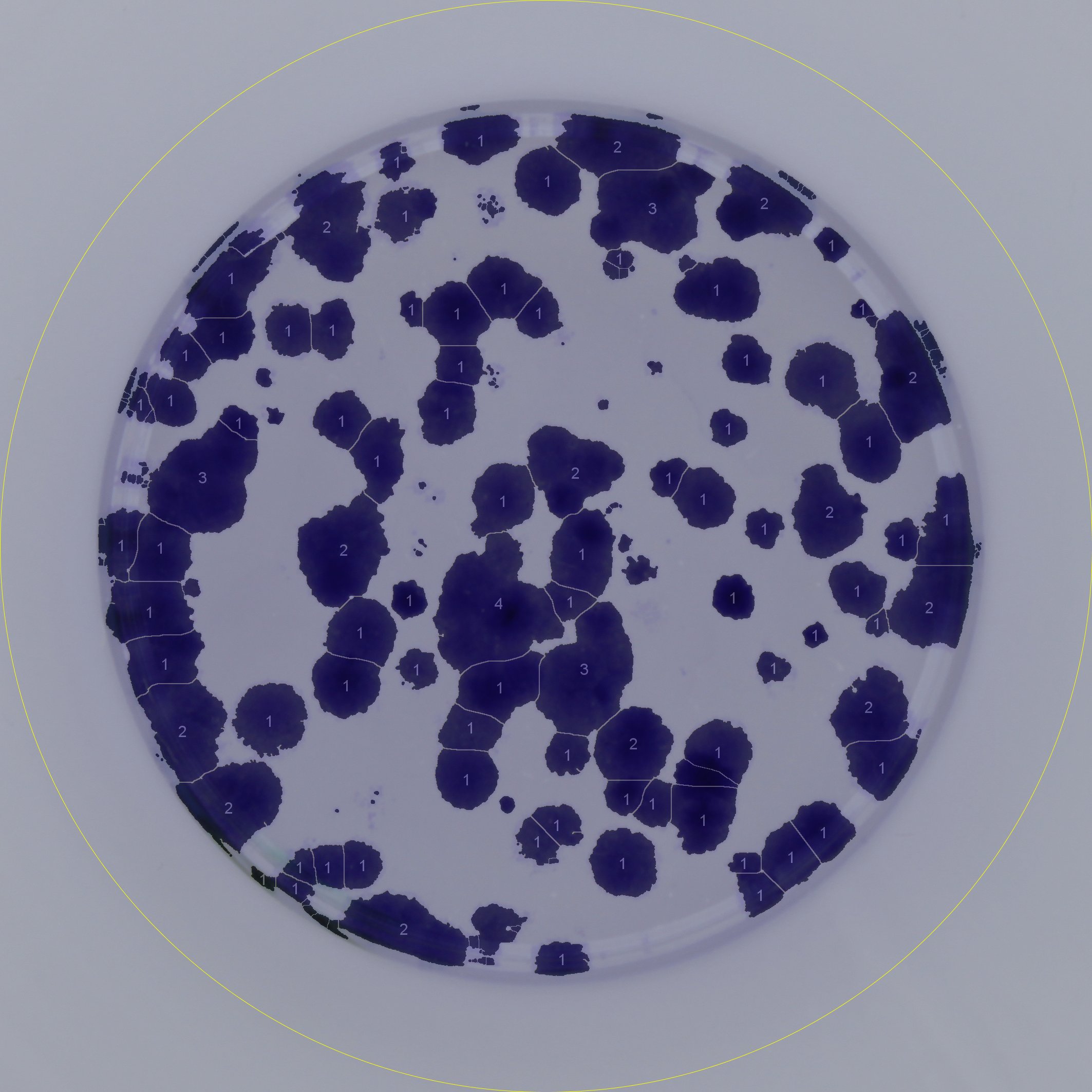

Supplement: S3 Datasets — It also contains a text file where results achieved by automated (CoCoNut, CAI, AutoCellSeg, and OpenCFU) and manual methods are summarized. (ZIP) [file pone.0205823.s004.zip › 180501 HeLa Dish/13 Results.jpg]

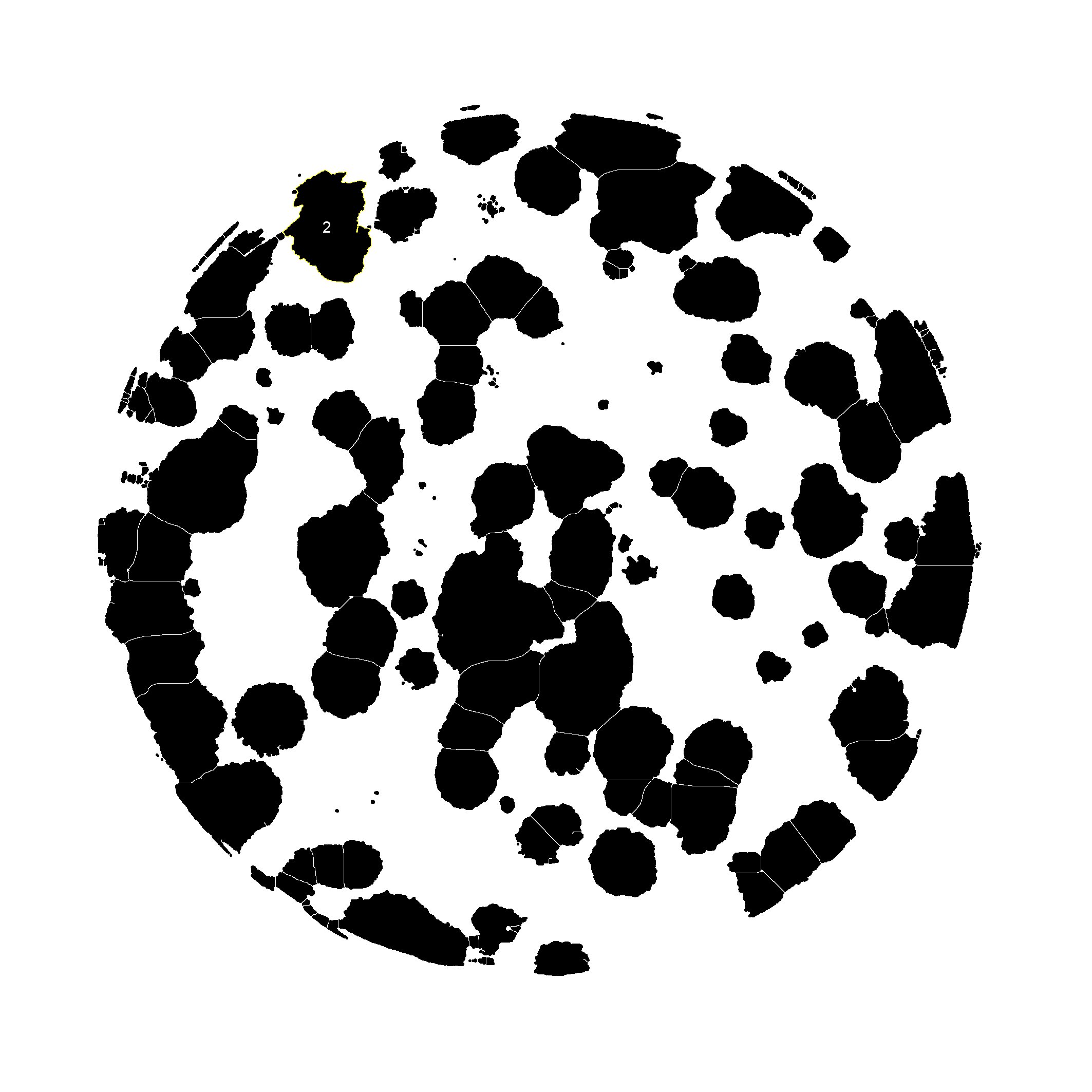

Supplement: S3 Datasets — It also contains a text file where results achieved by automated (CoCoNut, CAI, AutoCellSeg, and OpenCFU) and manual methods are summarized. (ZIP) [file pone.0205823.s004.zip › 180501 HeLa Dish/13 Second counting.jpg]

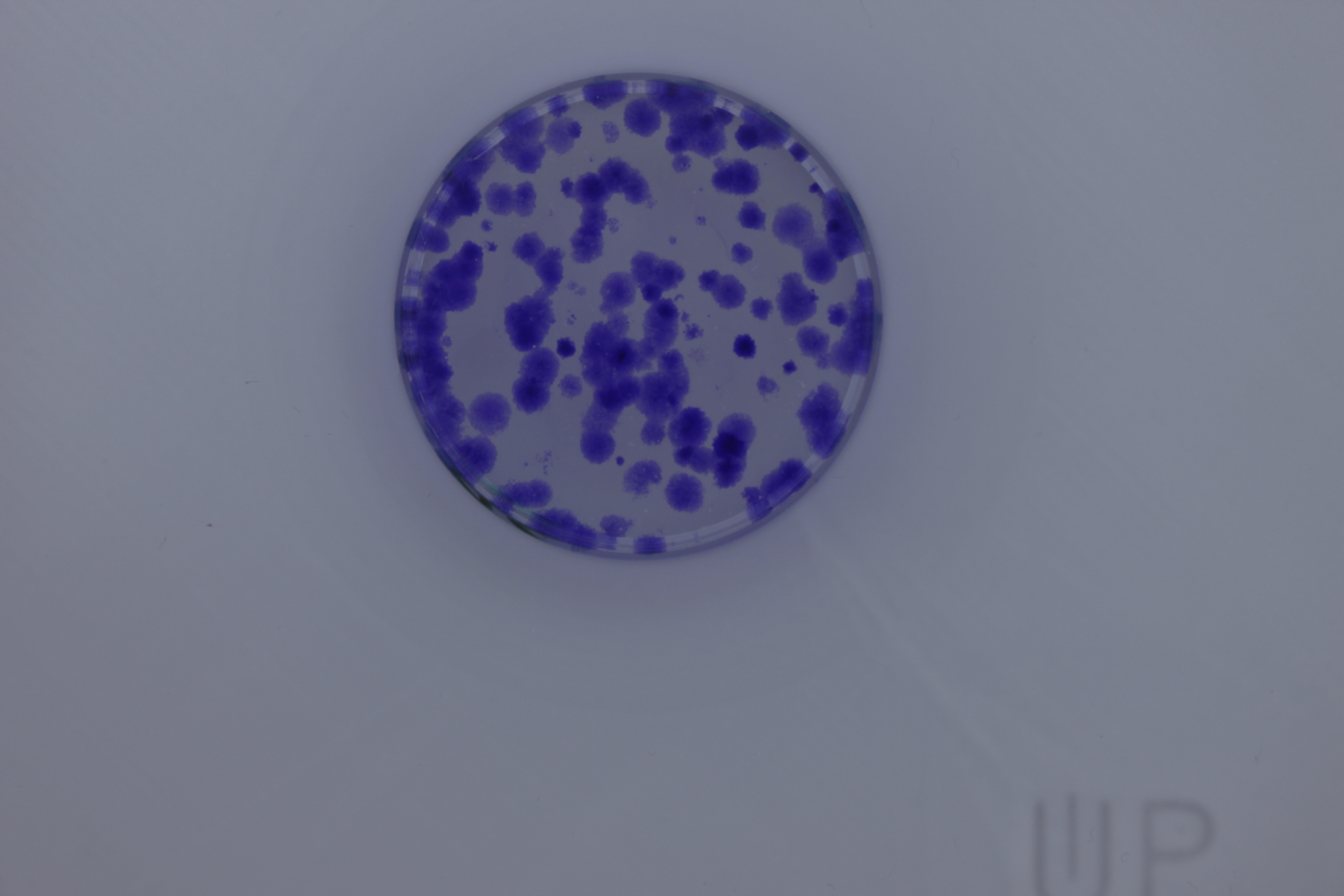

Supplement: S3 Datasets — It also contains a text file where results achieved by automated (CoCoNut, CAI, AutoCellSeg, and OpenCFU) and manual methods are summarized. (ZIP) [file pone.0205823.s004.zip › 180501 HeLa Dish/13.JPG]

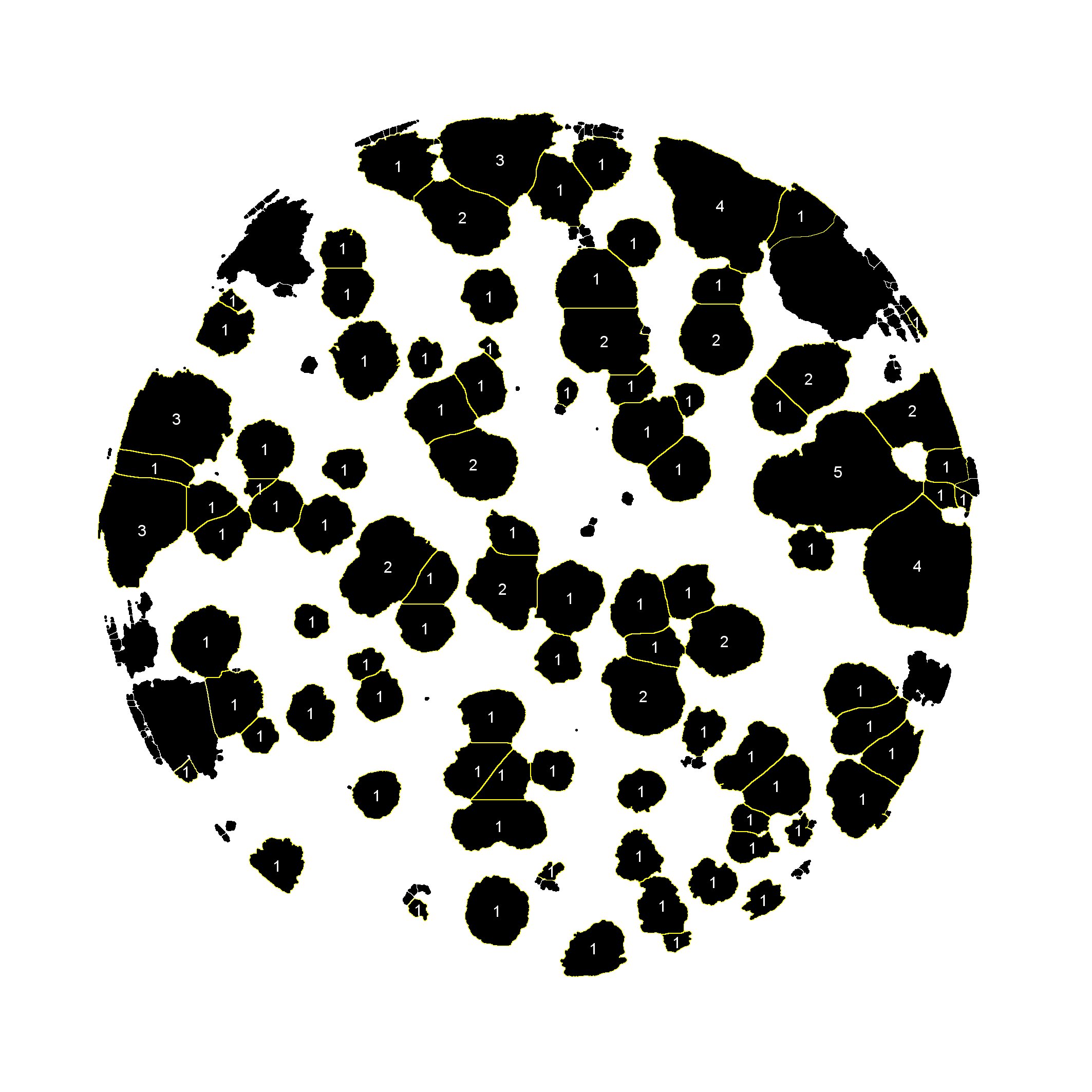

Supplement: S3 Datasets — It also contains a text file where results achieved by automated (CoCoNut, CAI, AutoCellSeg, and OpenCFU) and manual methods are summarized. (ZIP) [file pone.0205823.s004.zip › 180501 HeLa Dish/14 First counting.jpg]

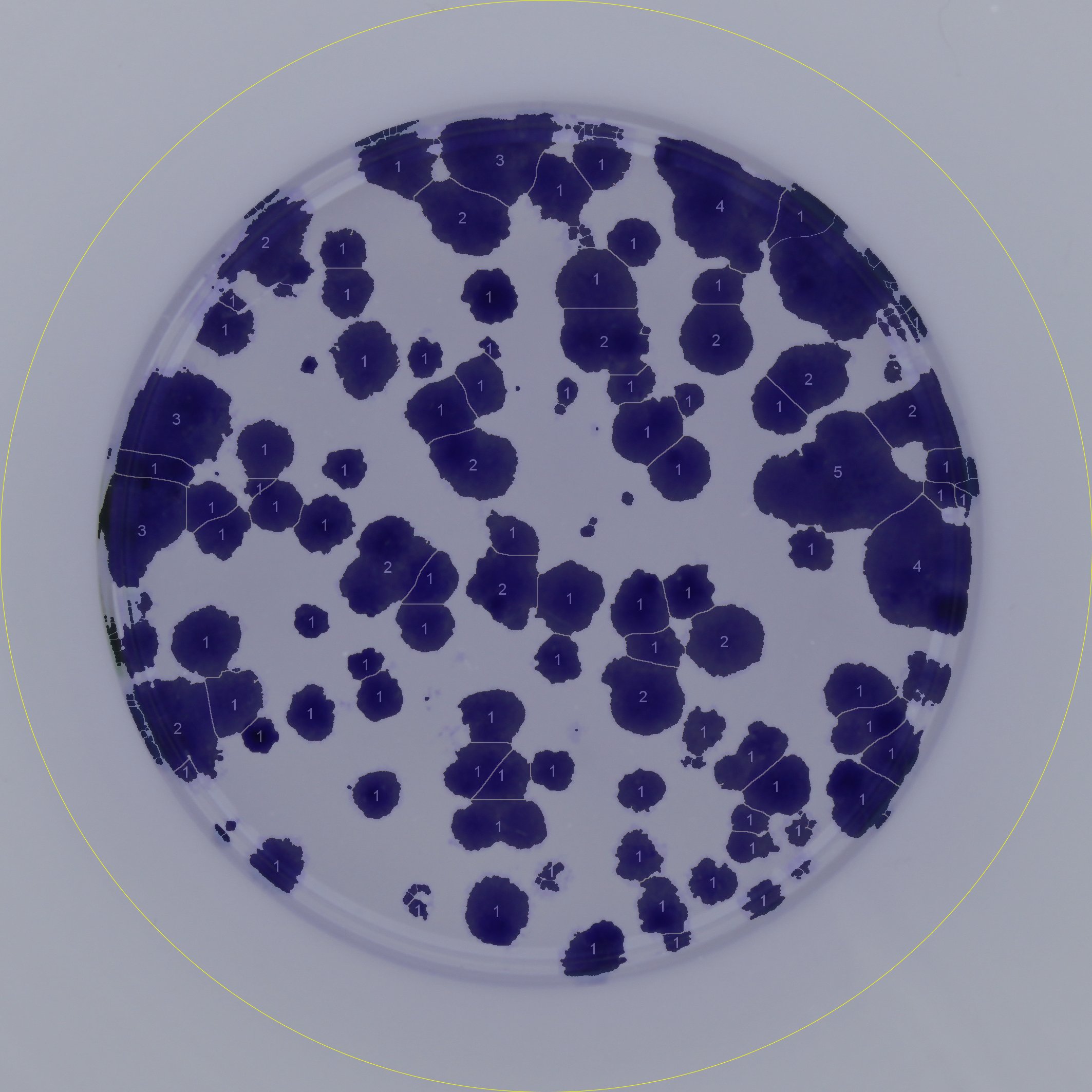

Supplement: S3 Datasets — It also contains a text file where results achieved by automated (CoCoNut, CAI, AutoCellSeg, and OpenCFU) and manual methods are summarized. (ZIP) [file pone.0205823.s004.zip › 180501 HeLa Dish/14 Results.jpg]

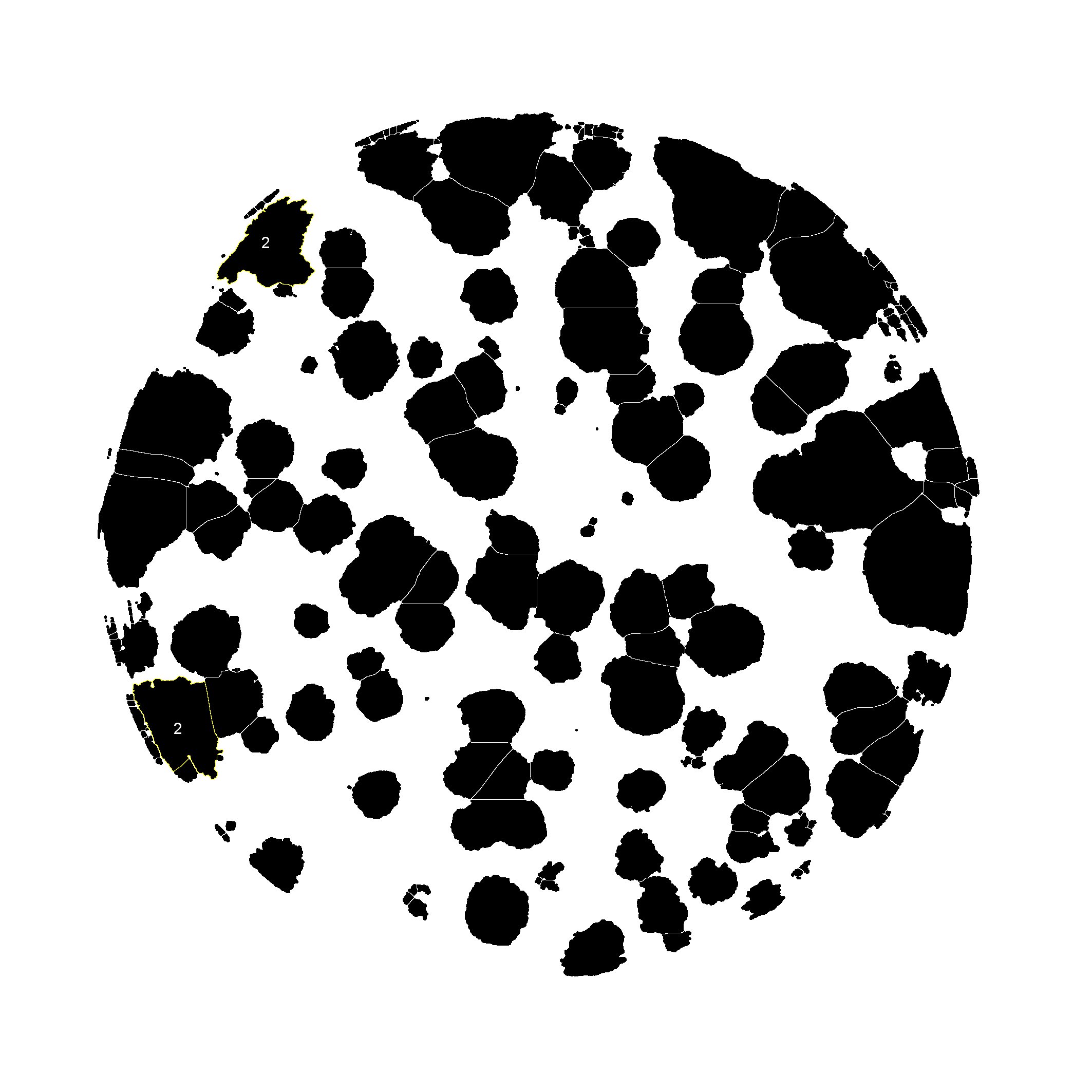

Supplement: S3 Datasets — It also contains a text file where results achieved by automated (CoCoNut, CAI, AutoCellSeg, and OpenCFU) and manual methods are summarized. (ZIP) [file pone.0205823.s004.zip › 180501 HeLa Dish/14 Second counting.jpg]

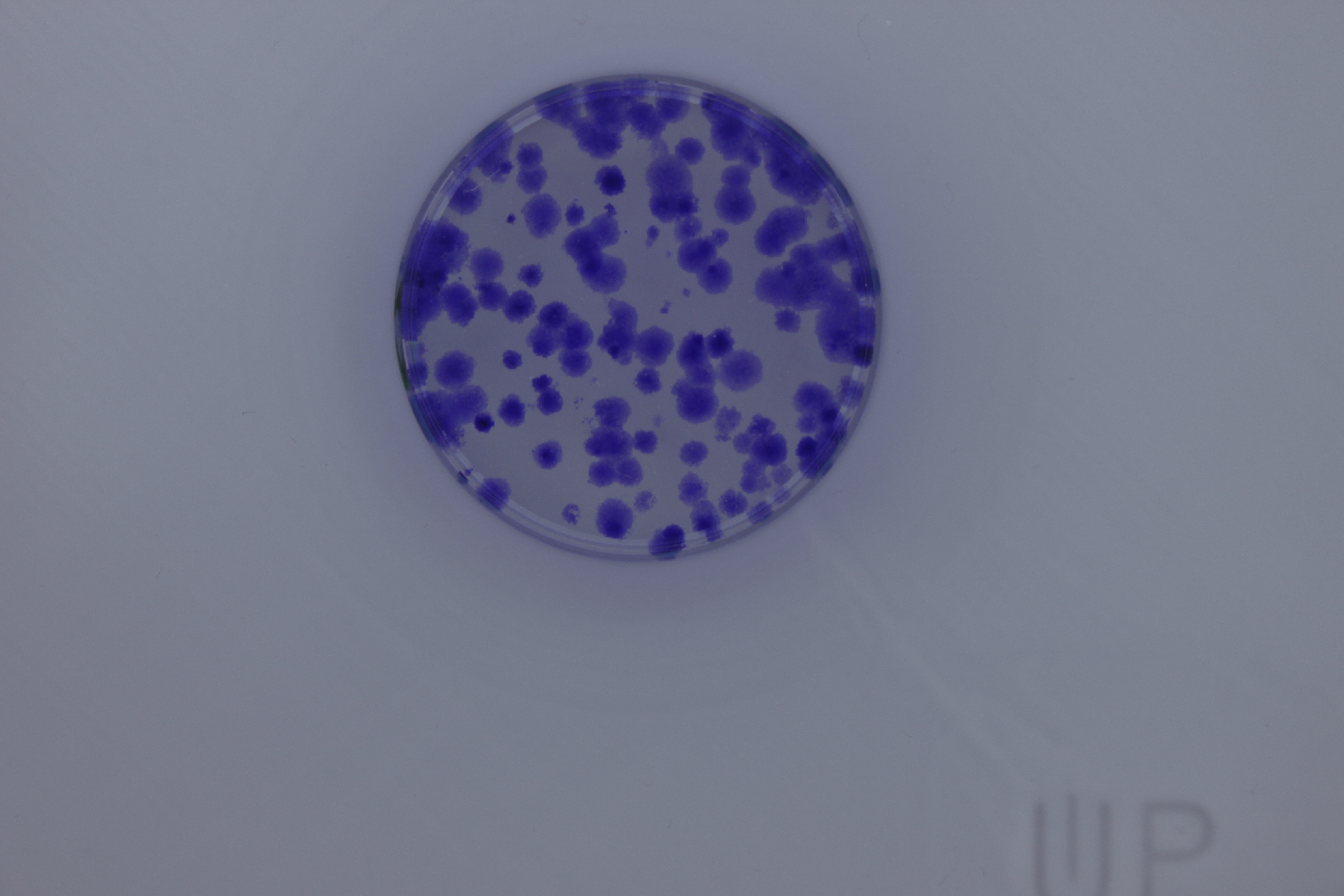

Supplement: S3 Datasets — It also contains a text file where results achieved by automated (CoCoNut, CAI, AutoCellSeg, and OpenCFU) and manual methods are summarized. (ZIP) [file pone.0205823.s004.zip › 180501 HeLa Dish/14.JPG]

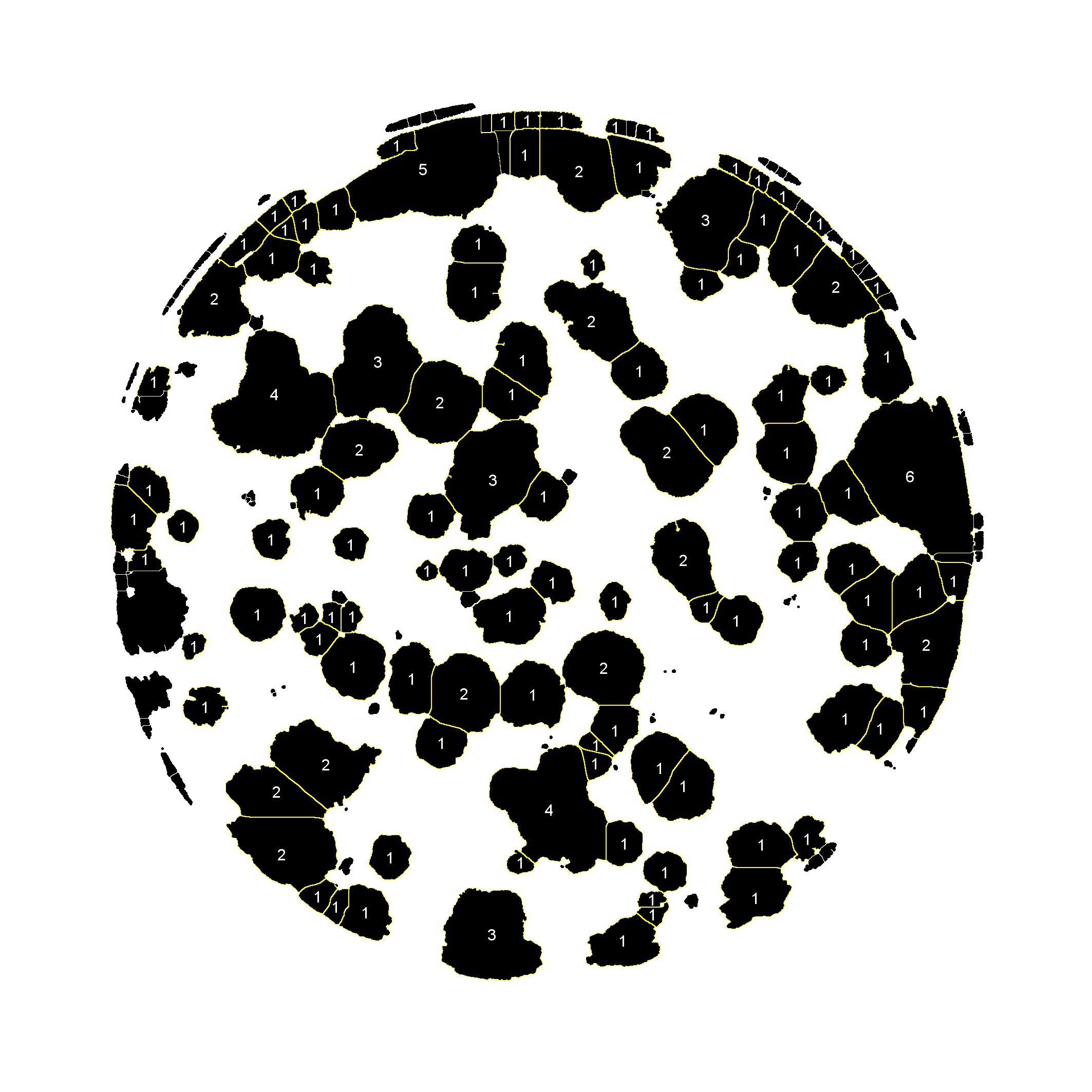

Supplement: S3 Datasets — It also contains a text file where results achieved by automated (CoCoNut, CAI, AutoCellSeg, and OpenCFU) and manual methods are summarized. (ZIP) [file pone.0205823.s004.zip › 180501 HeLa Dish/15 First counting.jpg]

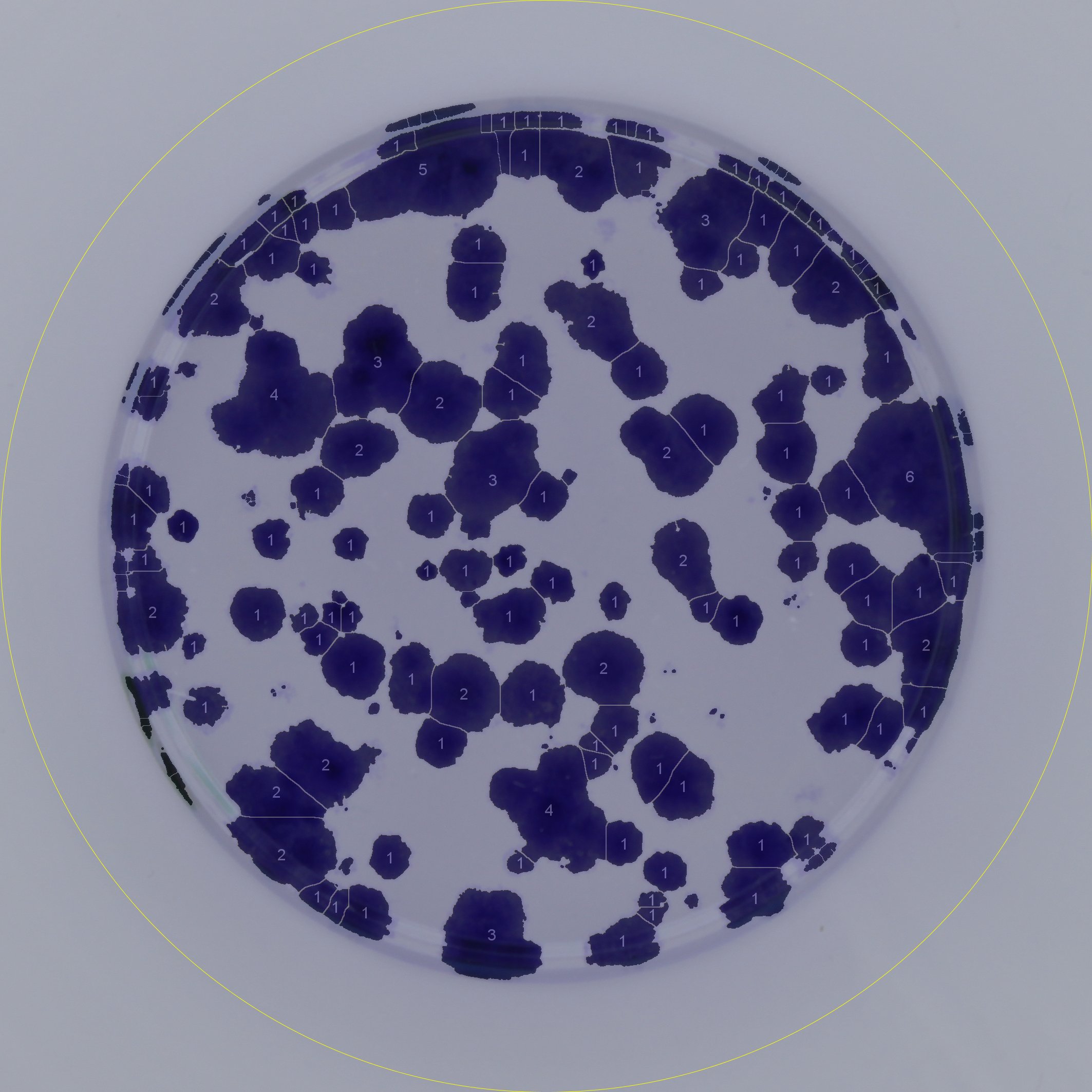

Supplement: S3 Datasets — It also contains a text file where results achieved by automated (CoCoNut, CAI, AutoCellSeg, and OpenCFU) and manual methods are summarized. (ZIP) [file pone.0205823.s004.zip › 180501 HeLa Dish/15 Results.jpg]

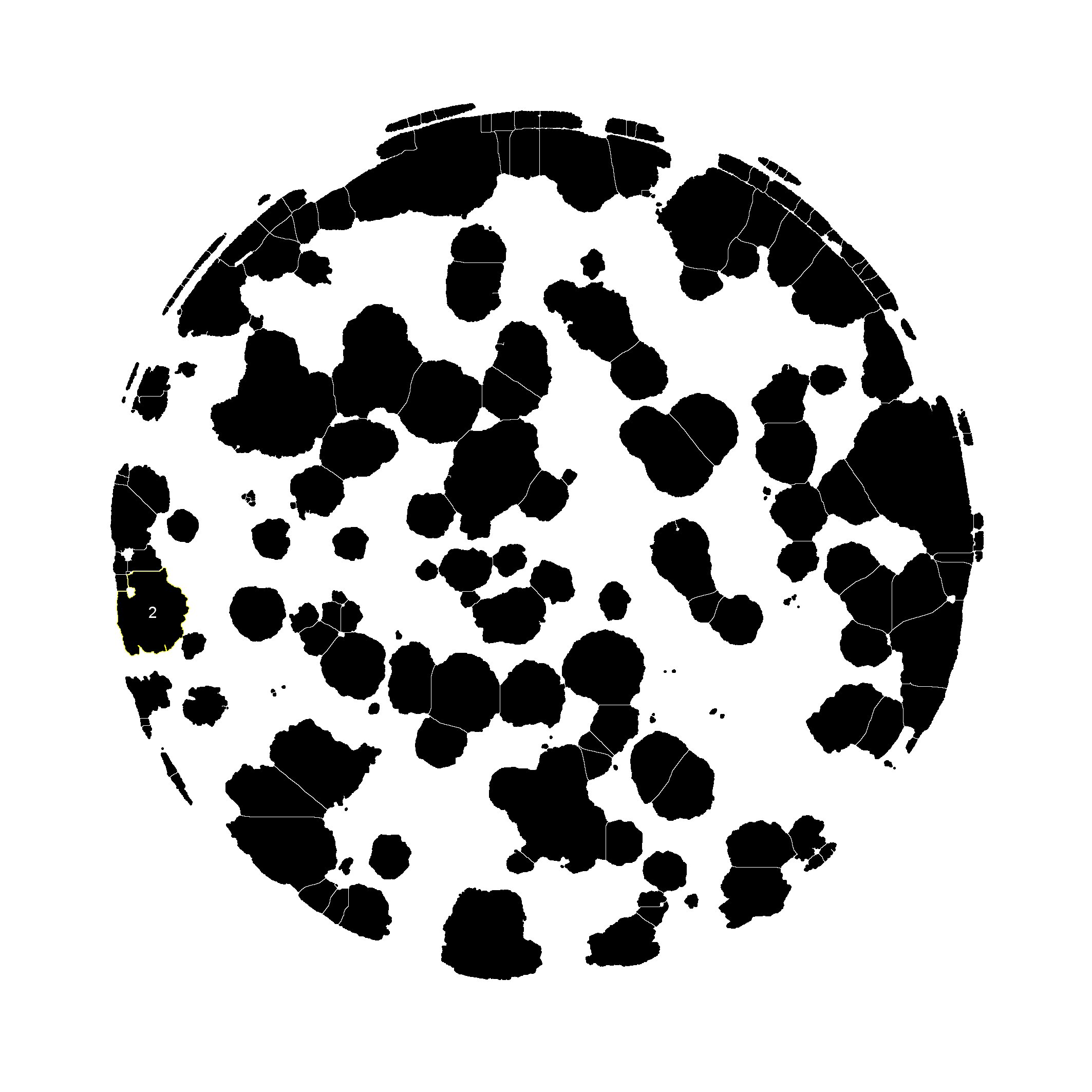

Supplement: S3 Datasets — It also contains a text file where results achieved by automated (CoCoNut, CAI, AutoCellSeg, and OpenCFU) and manual methods are summarized. (ZIP) [file pone.0205823.s004.zip › 180501 HeLa Dish/15 Second counting.jpg]

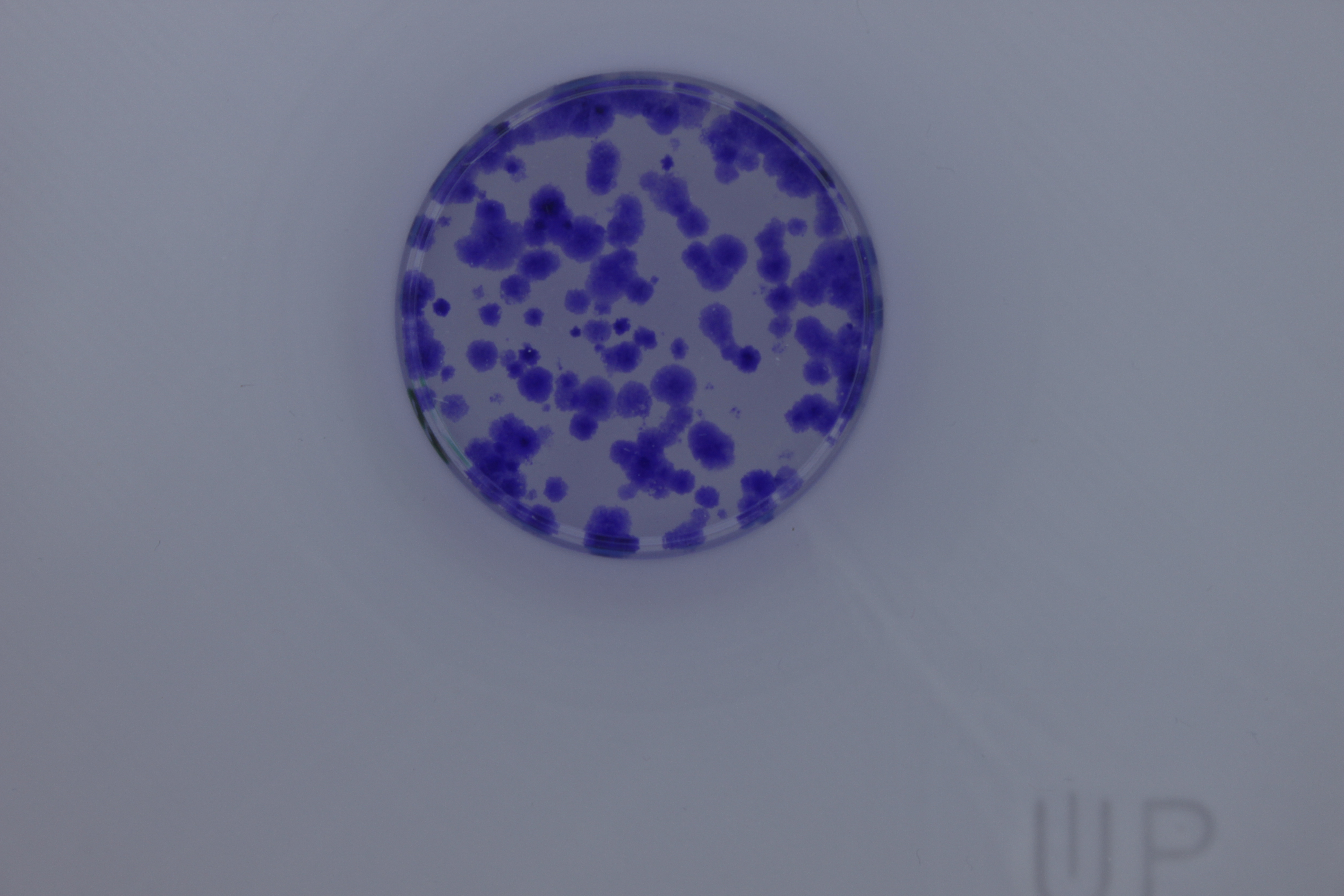

Supplement: S3 Datasets — It also contains a text file where results achieved by automated (CoCoNut, CAI, AutoCellSeg, and OpenCFU) and manual methods are summarized. (ZIP) [file pone.0205823.s004.zip › 180501 HeLa Dish/15.JPG]

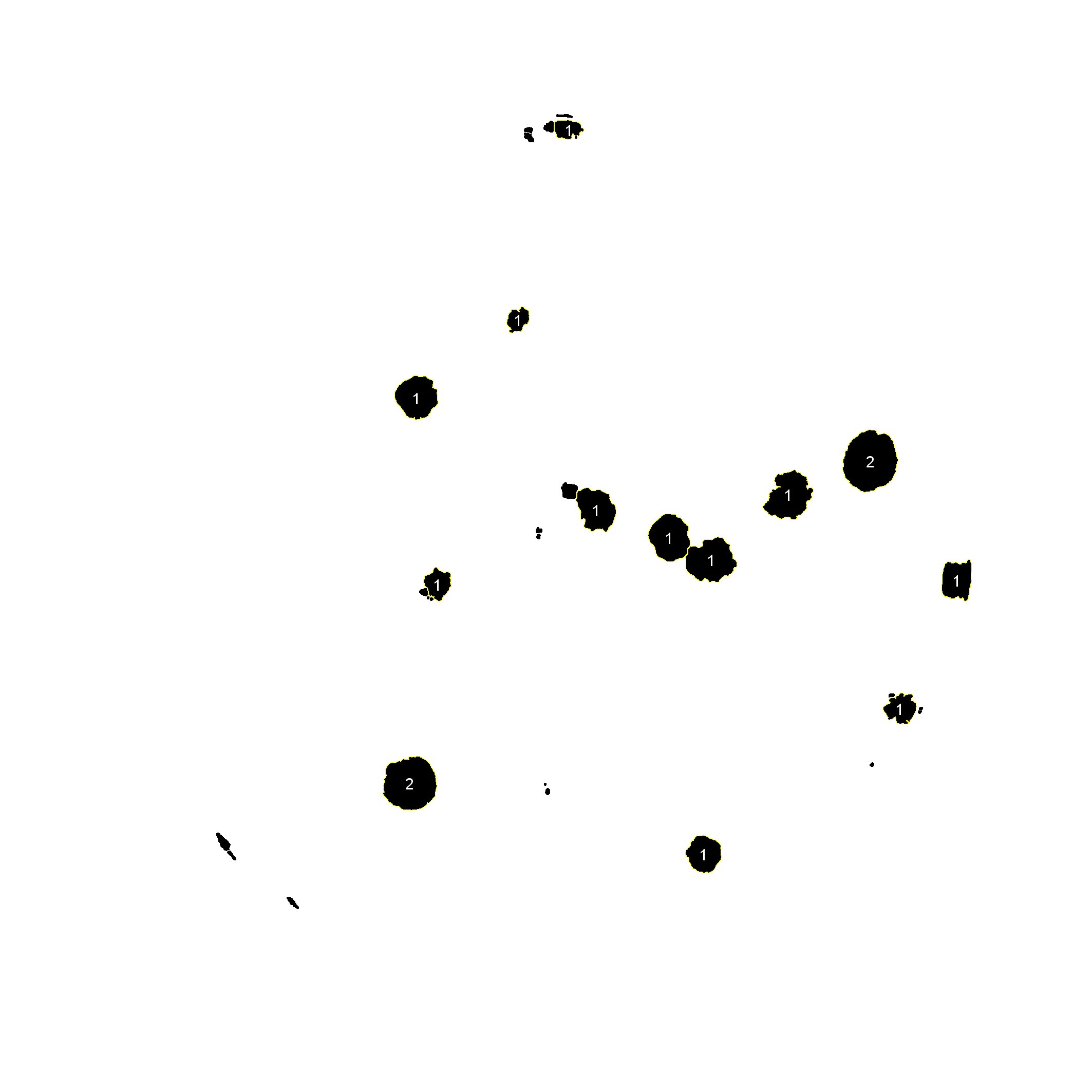

Supplement: S3 Datasets — It also contains a text file where results achieved by automated (CoCoNut, CAI, AutoCellSeg, and OpenCFU) and manual methods are summarized. (ZIP) [file pone.0205823.s004.zip › 180501 HeLa Dish/2 First counting.jpg]

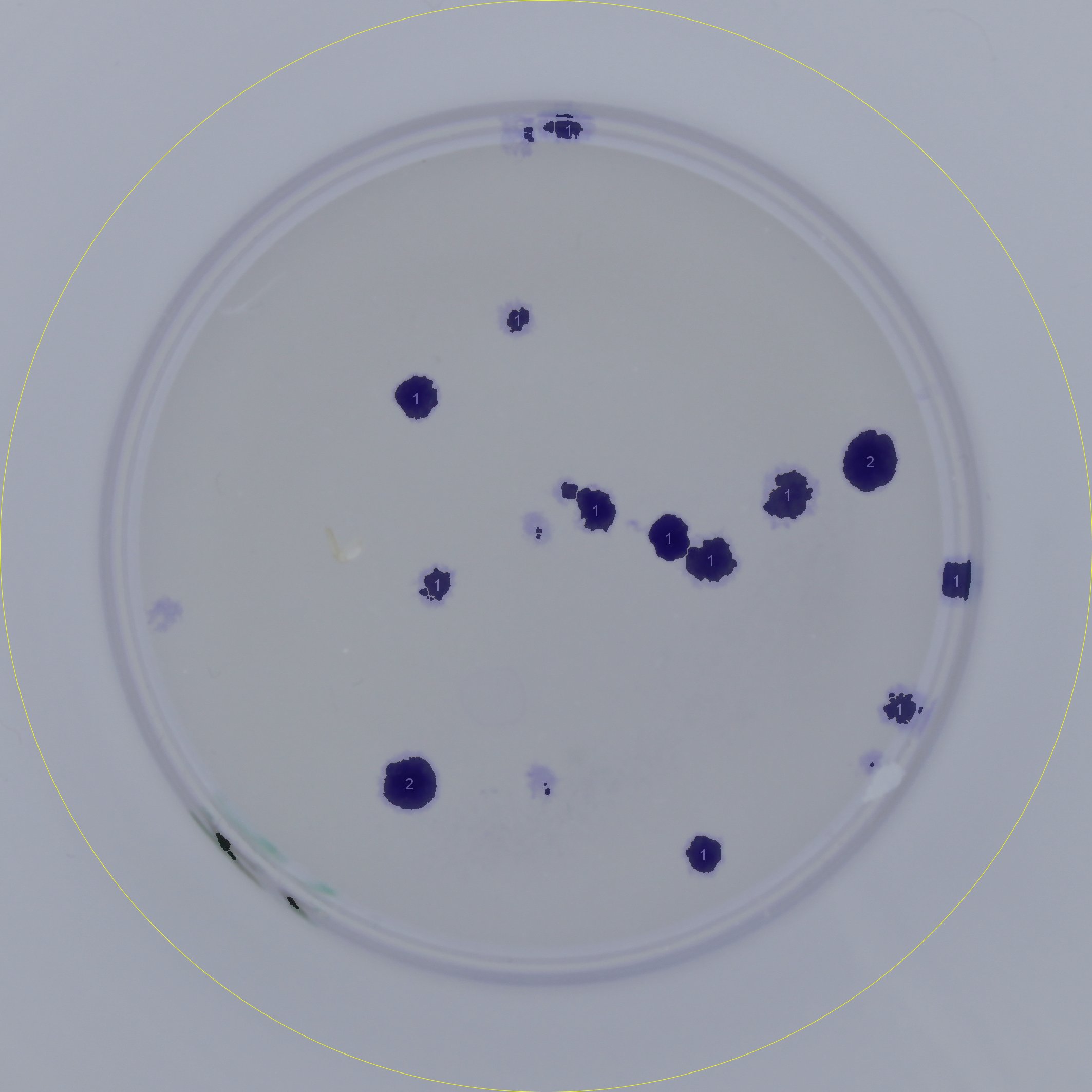

Supplement: S3 Datasets — It also contains a text file where results achieved by automated (CoCoNut, CAI, AutoCellSeg, and OpenCFU) and manual methods are summarized. (ZIP) [file pone.0205823.s004.zip › 180501 HeLa Dish/2 Results.jpg]

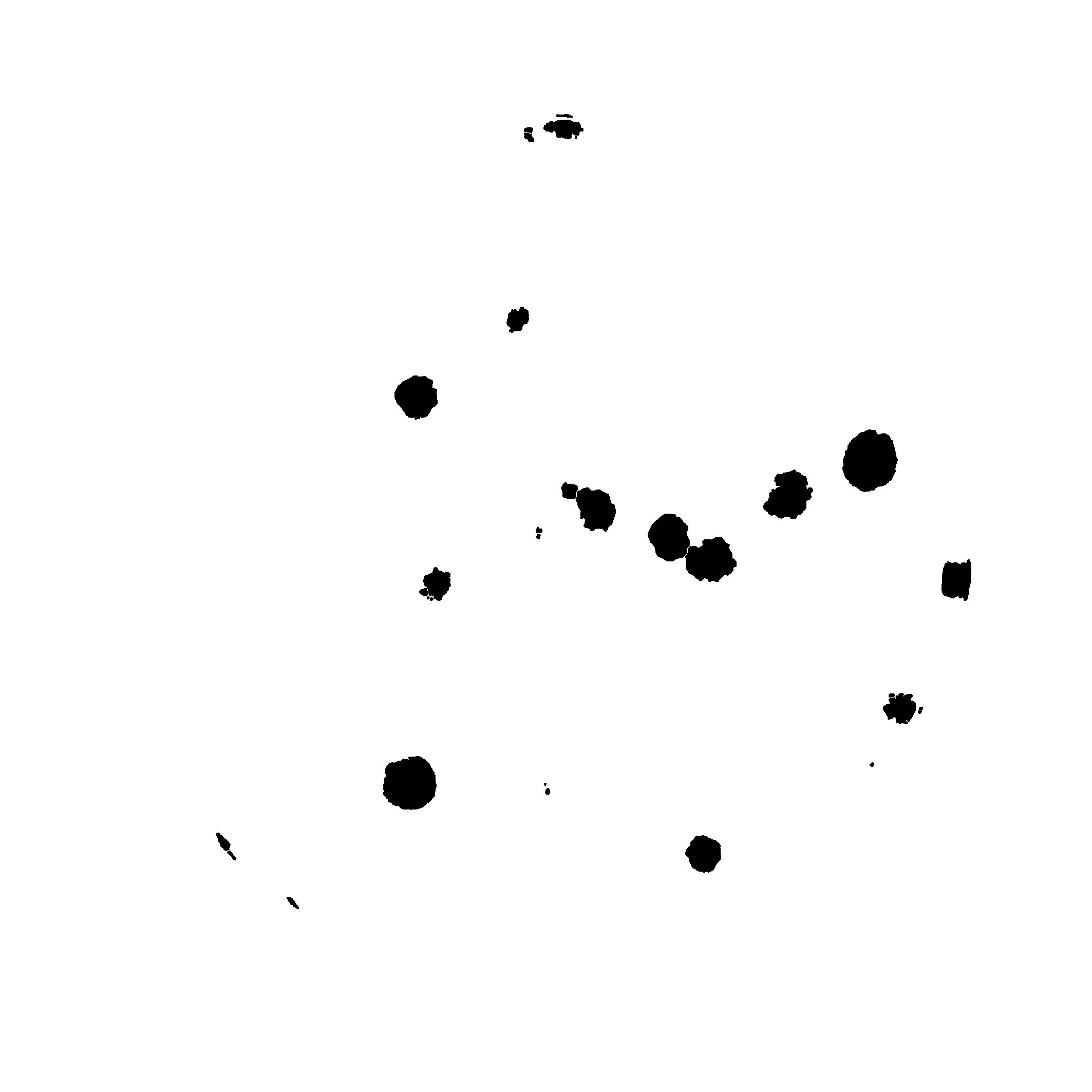

Supplement: S3 Datasets — It also contains a text file where results achieved by automated (CoCoNut, CAI, AutoCellSeg, and OpenCFU) and manual methods are summarized. (ZIP) [file pone.0205823.s004.zip › 180501 HeLa Dish/2 Second counting.jpg]

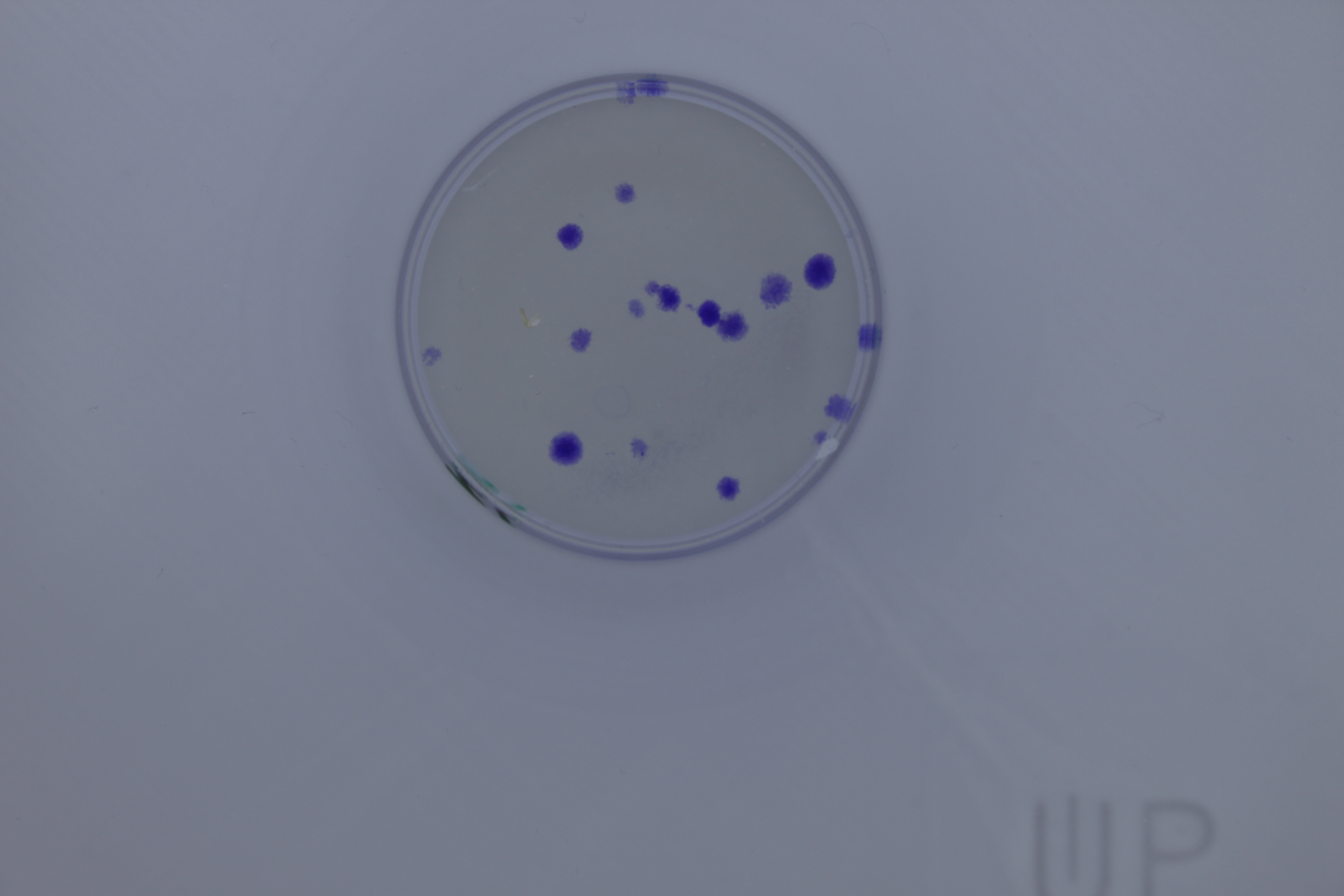

Supplement: S3 Datasets — It also contains a text file where results achieved by automated (CoCoNut, CAI, AutoCellSeg, and OpenCFU) and manual methods are summarized. (ZIP) [file pone.0205823.s004.zip › 180501 HeLa Dish/2.JPG]

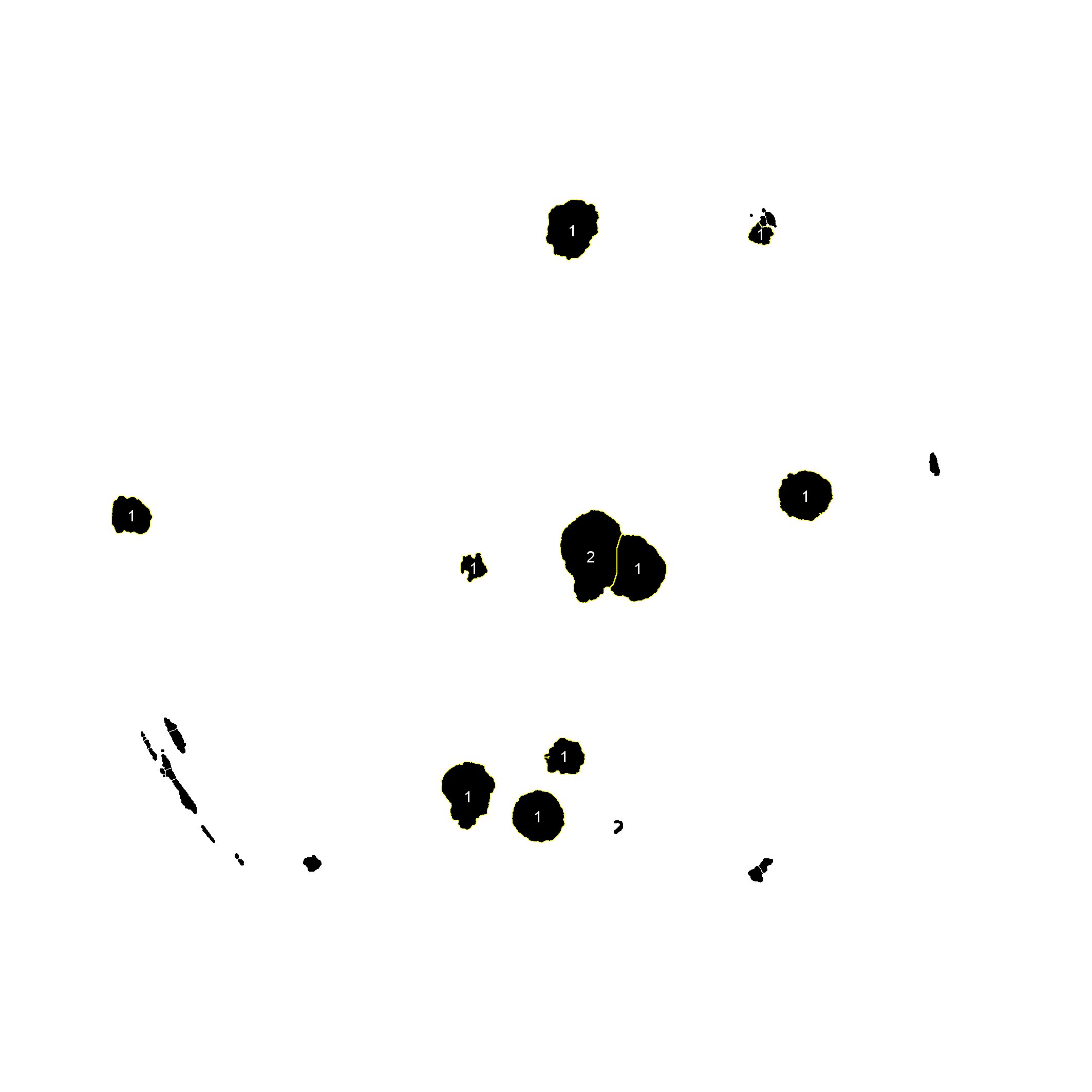

Supplement: S3 Datasets — It also contains a text file where results achieved by automated (CoCoNut, CAI, AutoCellSeg, and OpenCFU) and manual methods are summarized. (ZIP) [file pone.0205823.s004.zip › 180501 HeLa Dish/3 First counting.jpg]

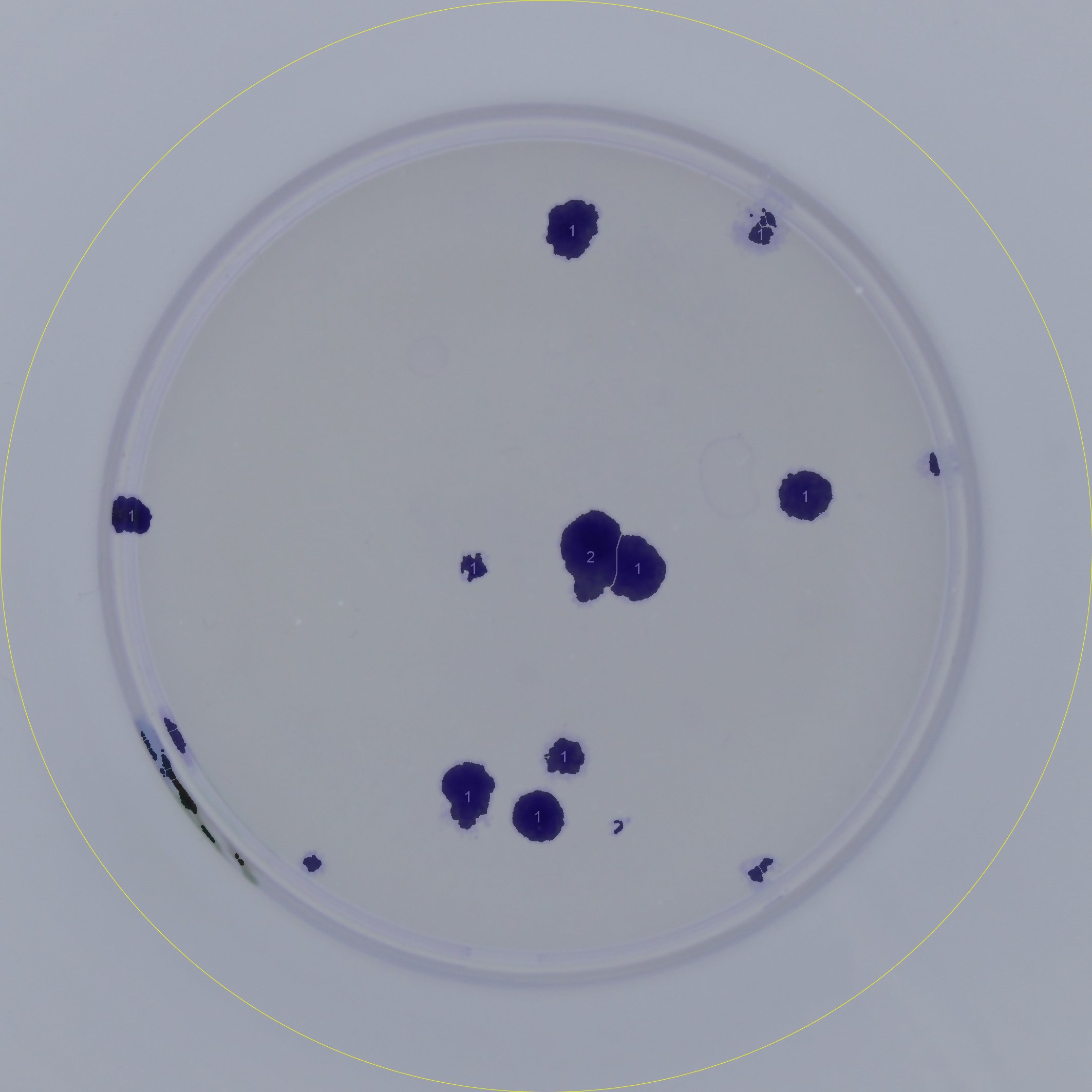

Supplement: S3 Datasets — It also contains a text file where results achieved by automated (CoCoNut, CAI, AutoCellSeg, and OpenCFU) and manual methods are summarized. (ZIP) [file pone.0205823.s004.zip › 180501 HeLa Dish/3 Results.jpg]

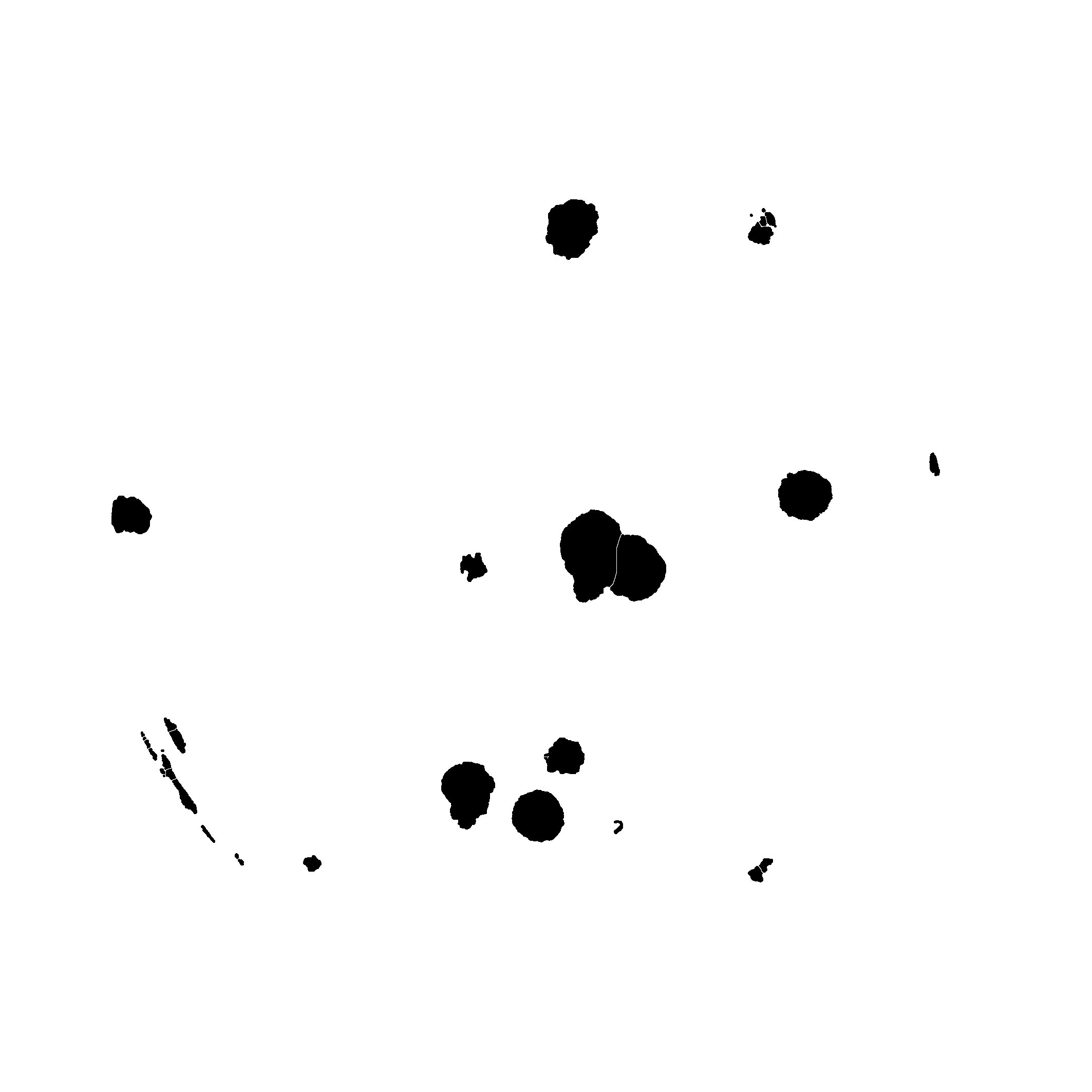

Supplement: S3 Datasets — It also contains a text file where results achieved by automated (CoCoNut, CAI, AutoCellSeg, and OpenCFU) and manual methods are summarized. (ZIP) [file pone.0205823.s004.zip › 180501 HeLa Dish/3 Second counting.jpg]

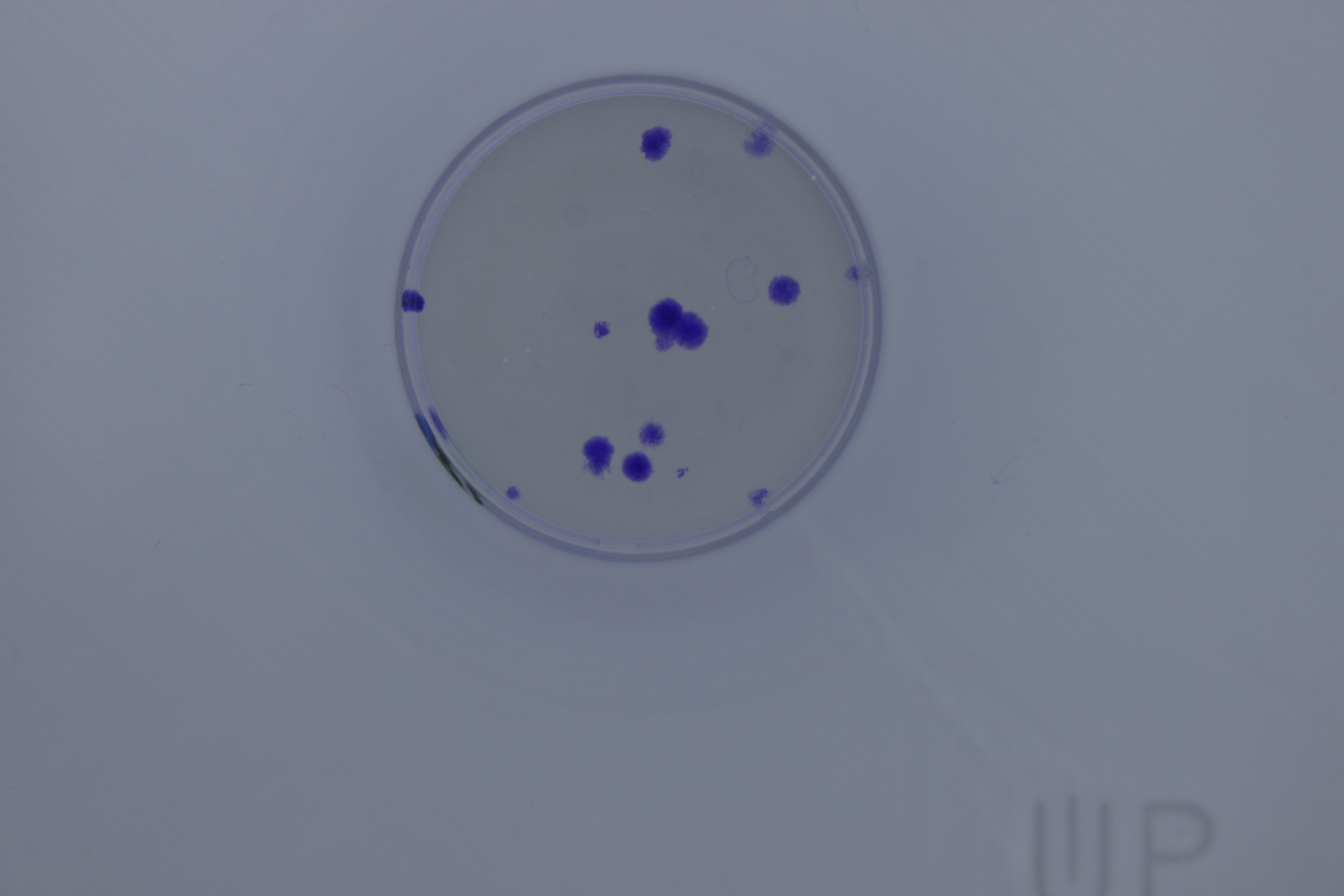

Supplement: S3 Datasets — It also contains a text file where results achieved by automated (CoCoNut, CAI, AutoCellSeg, and OpenCFU) and manual methods are summarized. (ZIP) [file pone.0205823.s004.zip › 180501 HeLa Dish/3.JPG]

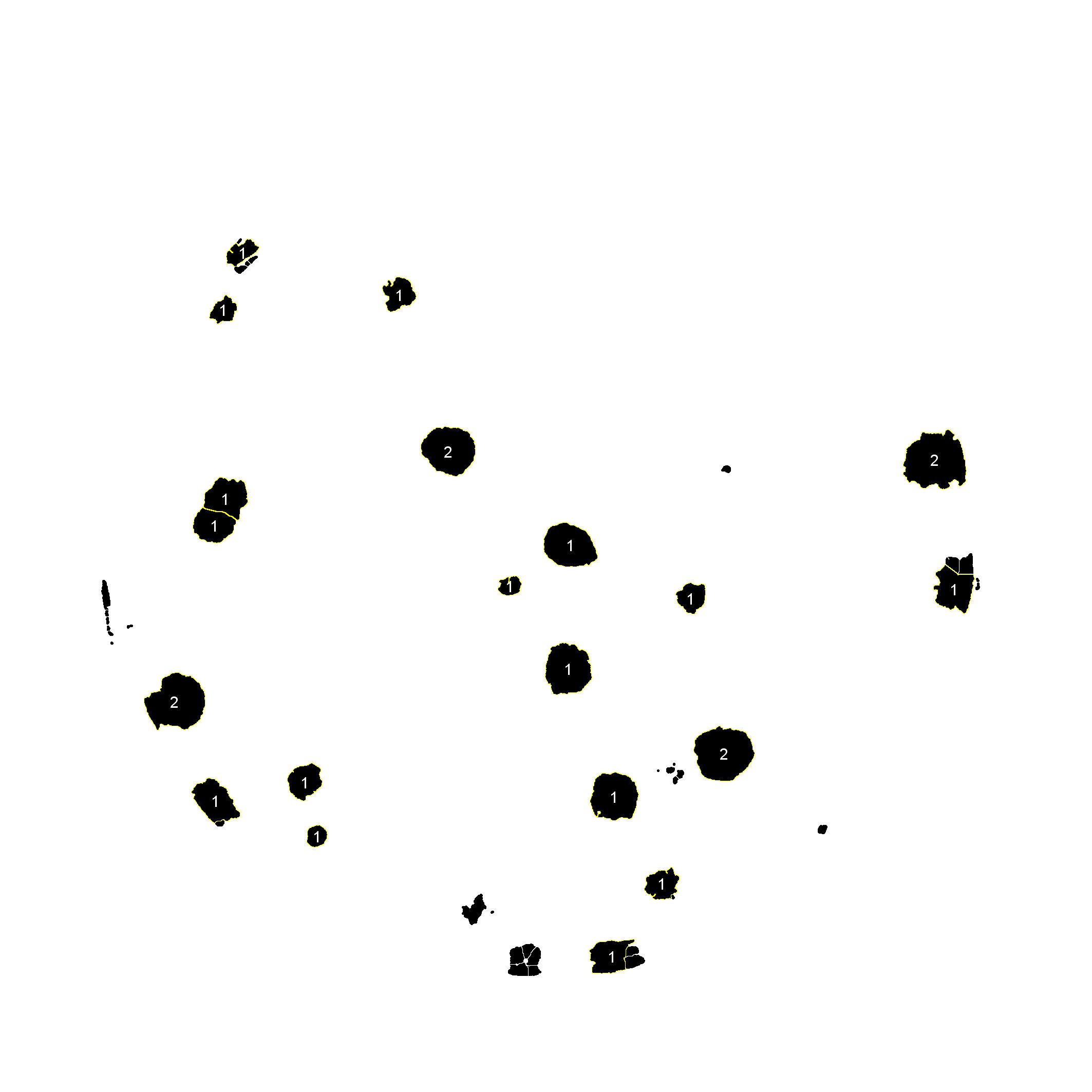

Supplement: S3 Datasets — It also contains a text file where results achieved by automated (CoCoNut, CAI, AutoCellSeg, and OpenCFU) and manual methods are summarized. (ZIP) [file pone.0205823.s004.zip › 180501 HeLa Dish/4 First counting.jpg]

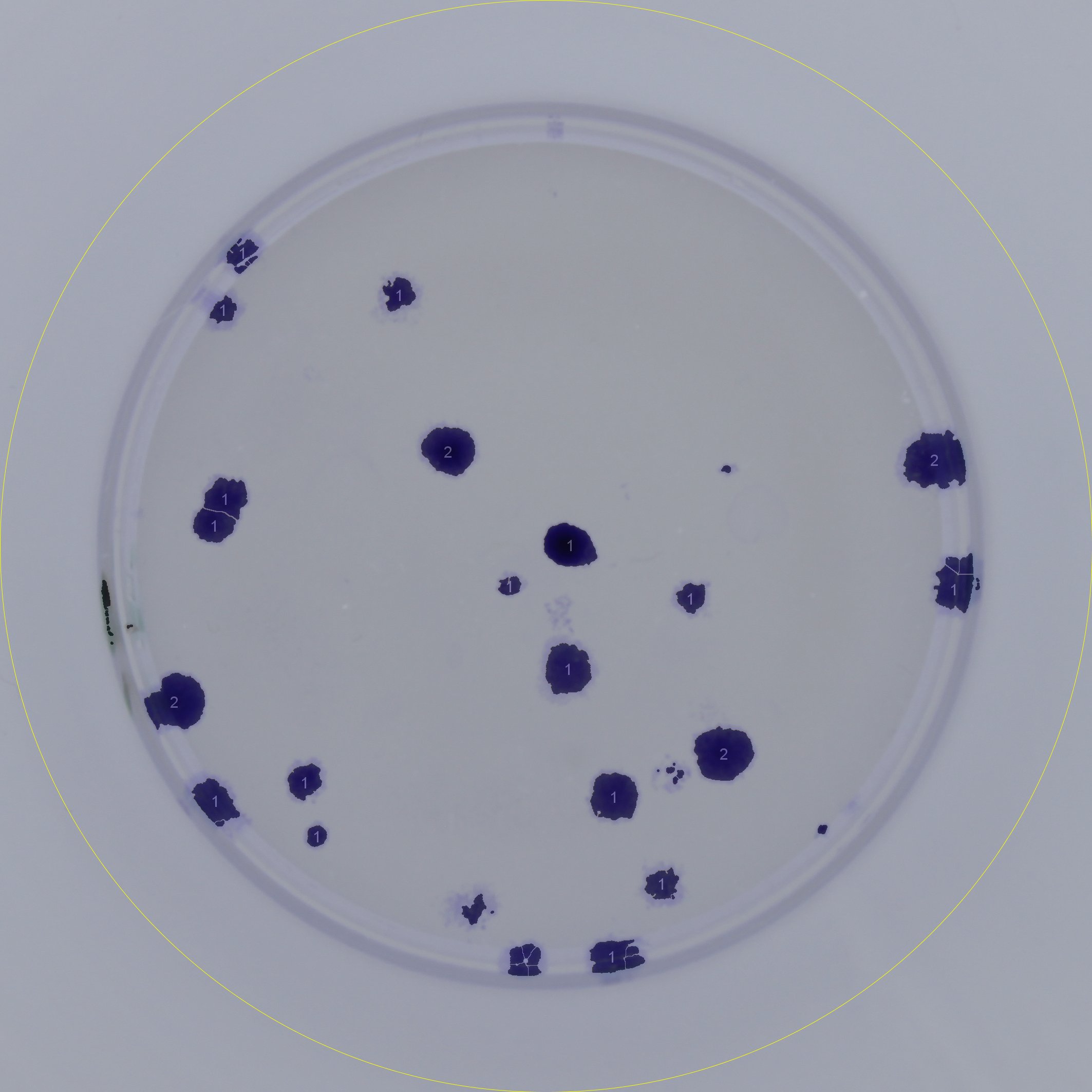

Supplement: S3 Datasets — It also contains a text file where results achieved by automated (CoCoNut, CAI, AutoCellSeg, and OpenCFU) and manual methods are summarized. (ZIP) [file pone.0205823.s004.zip › 180501 HeLa Dish/4 Results.jpg]

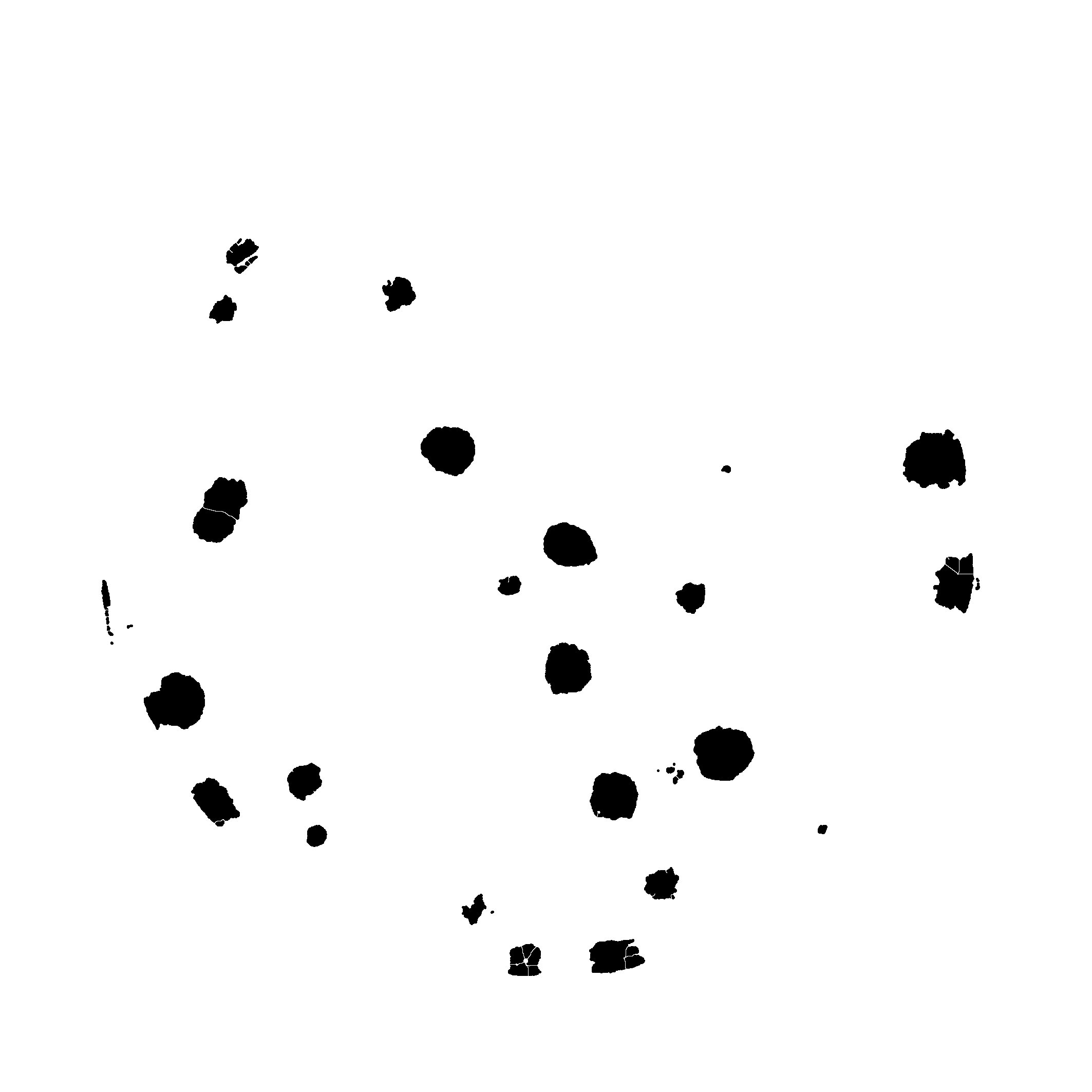

Supplement: S3 Datasets — It also contains a text file where results achieved by automated (CoCoNut, CAI, AutoCellSeg, and OpenCFU) and manual methods are summarized. (ZIP) [file pone.0205823.s004.zip › 180501 HeLa Dish/4 Second counting.jpg]

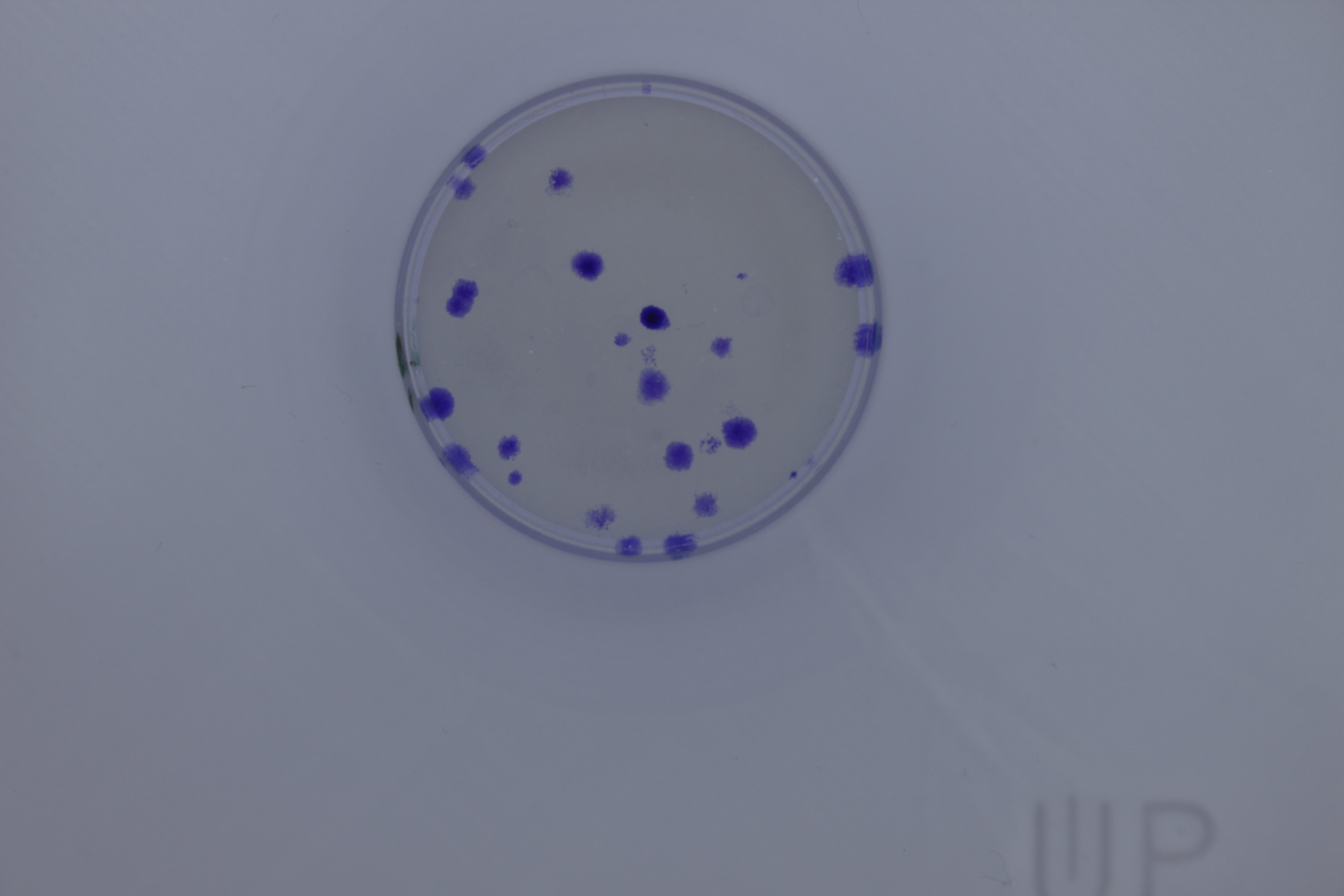

Supplement: S3 Datasets — It also contains a text file where results achieved by automated (CoCoNut, CAI, AutoCellSeg, and OpenCFU) and manual methods are summarized. (ZIP) [file pone.0205823.s004.zip › 180501 HeLa Dish/4.JPG]

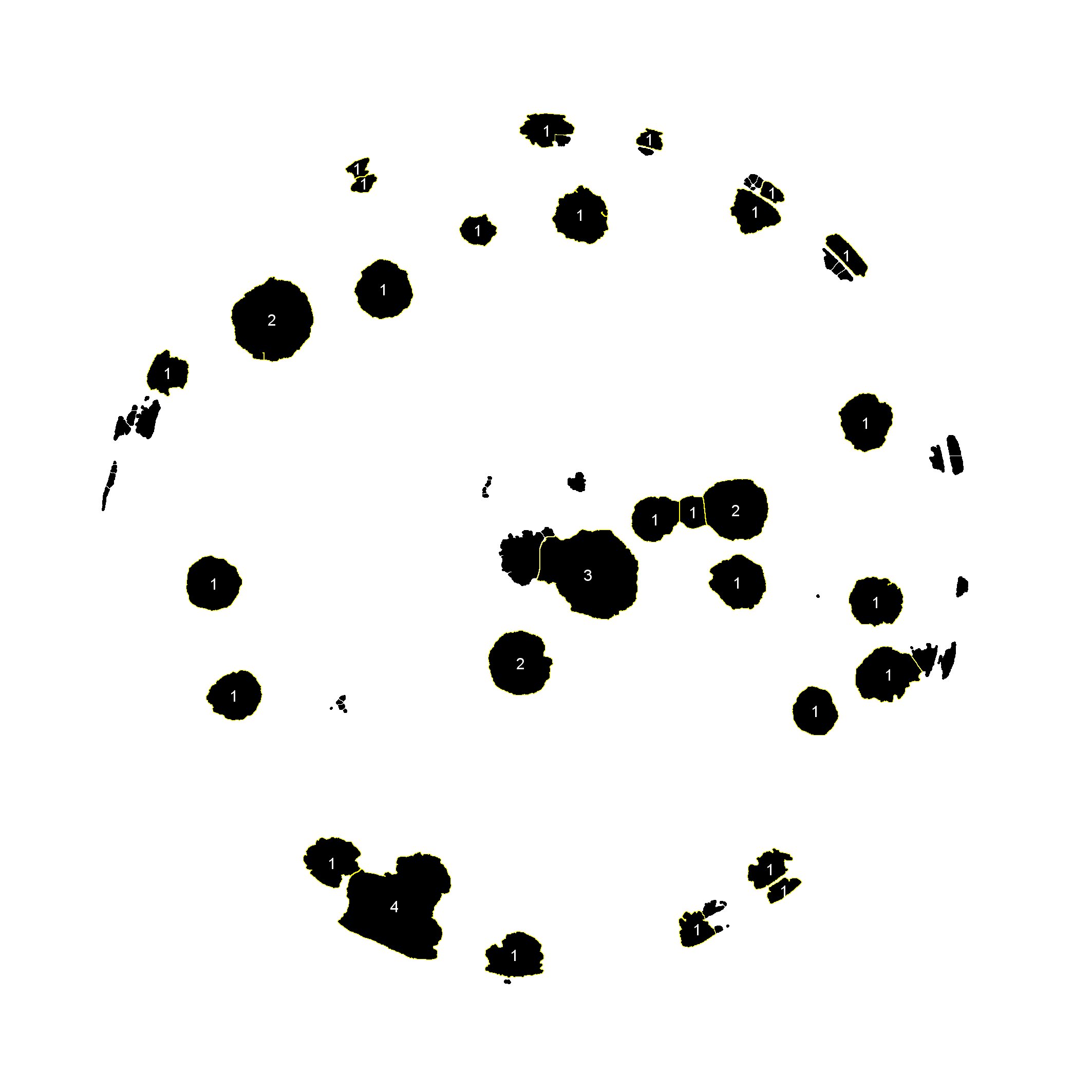

Supplement: S3 Datasets — It also contains a text file where results achieved by automated (CoCoNut, CAI, AutoCellSeg, and OpenCFU) and manual methods are summarized. (ZIP) [file pone.0205823.s004.zip › 180501 HeLa Dish/5 First counting.jpg]

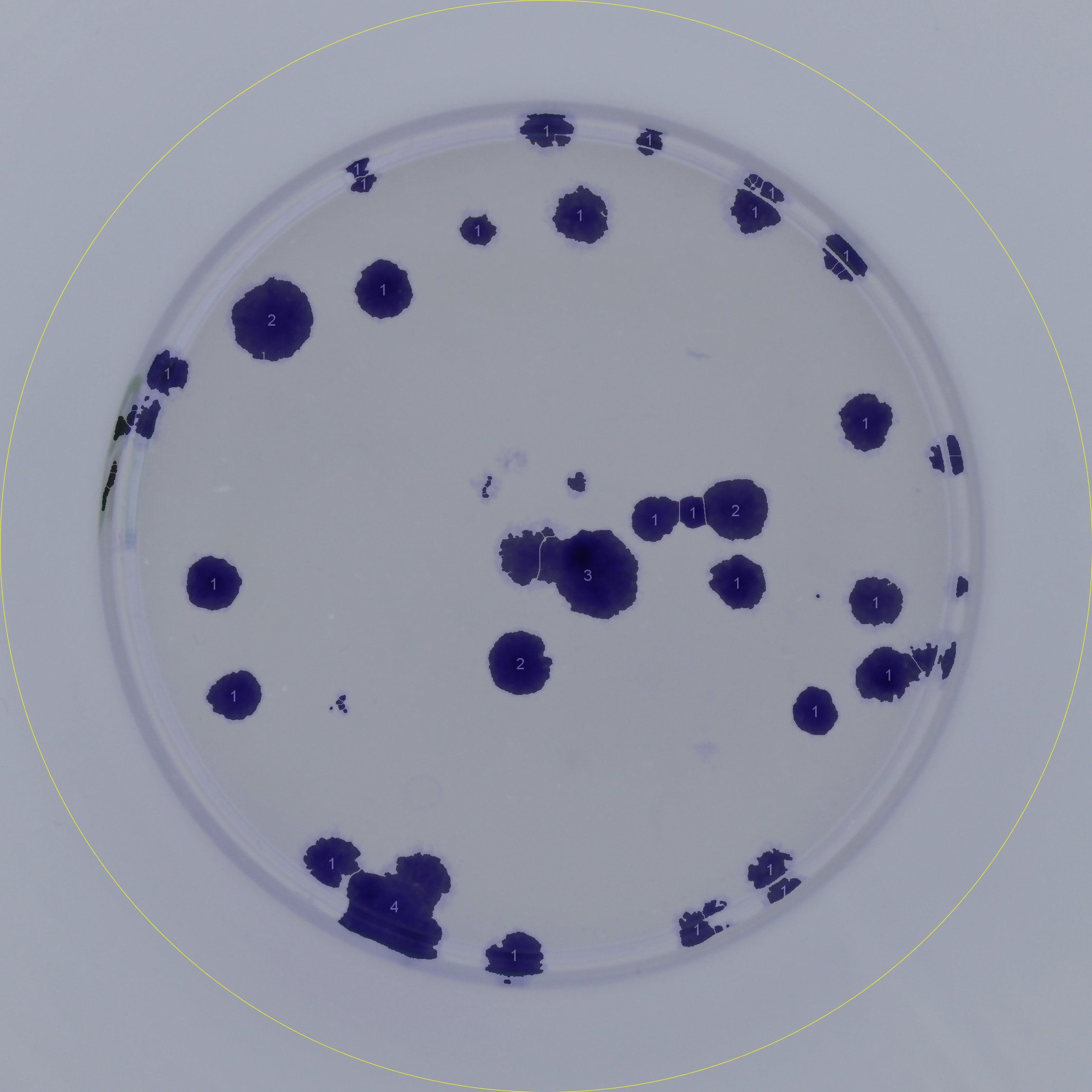

Supplement: S3 Datasets — It also contains a text file where results achieved by automated (CoCoNut, CAI, AutoCellSeg, and OpenCFU) and manual methods are summarized. (ZIP) [file pone.0205823.s004.zip › 180501 HeLa Dish/5 Results.jpg]

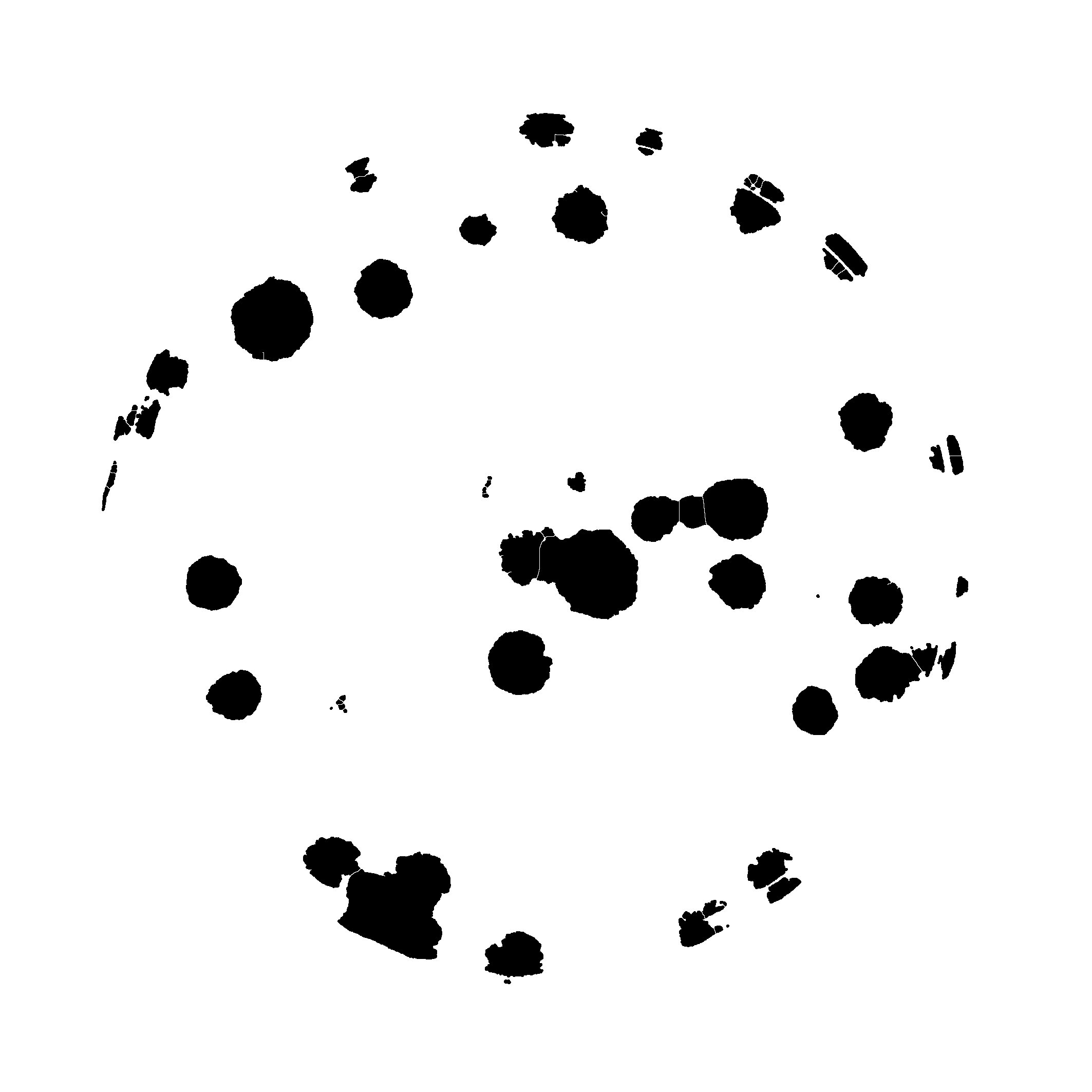

Supplement: S3 Datasets — It also contains a text file where results achieved by automated (CoCoNut, CAI, AutoCellSeg, and OpenCFU) and manual methods are summarized. (ZIP) [file pone.0205823.s004.zip › 180501 HeLa Dish/5 Second counting.jpg]

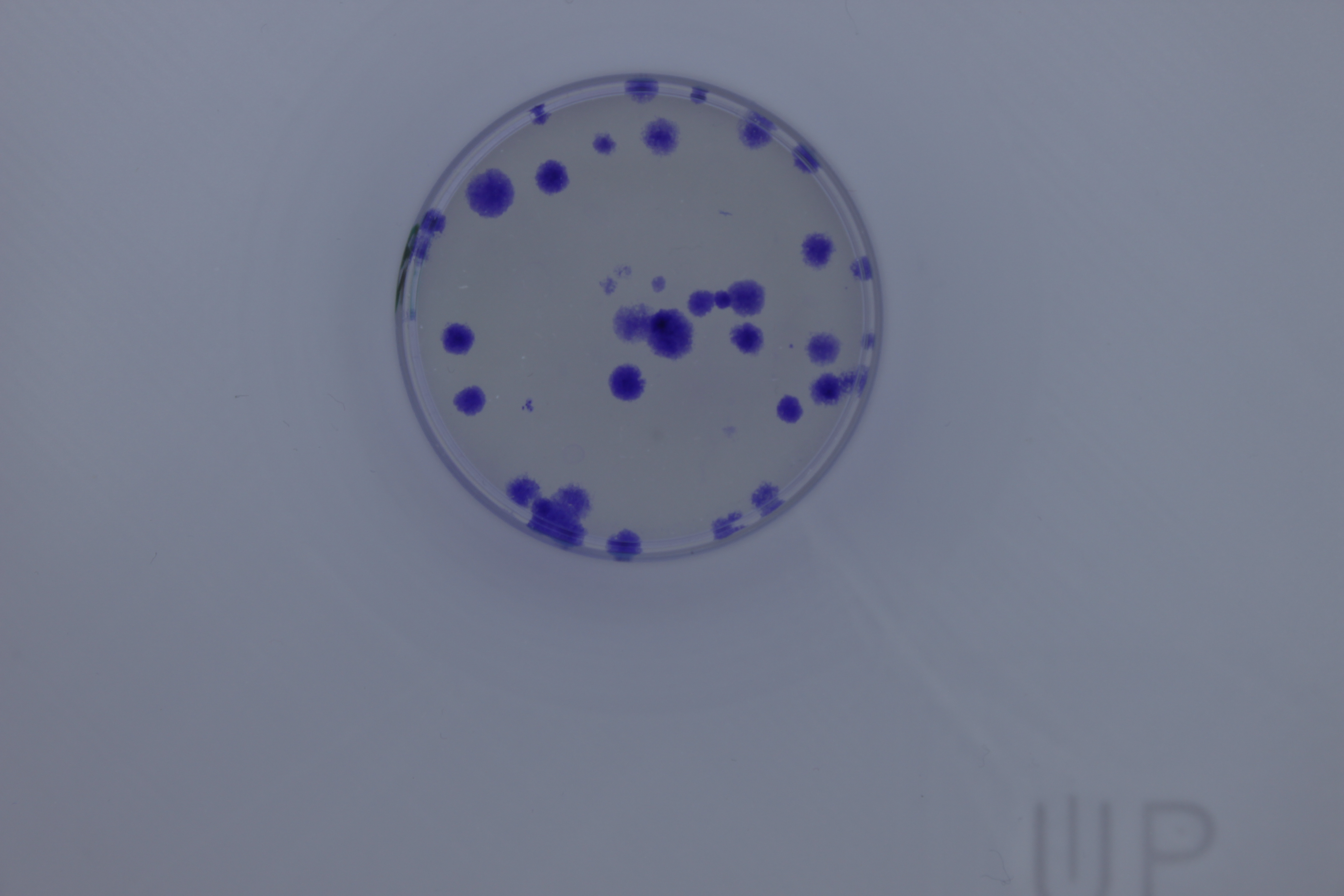

Supplement: S3 Datasets — It also contains a text file where results achieved by automated (CoCoNut, CAI, AutoCellSeg, and OpenCFU) and manual methods are summarized. (ZIP) [file pone.0205823.s004.zip › 180501 HeLa Dish/5.JPG]

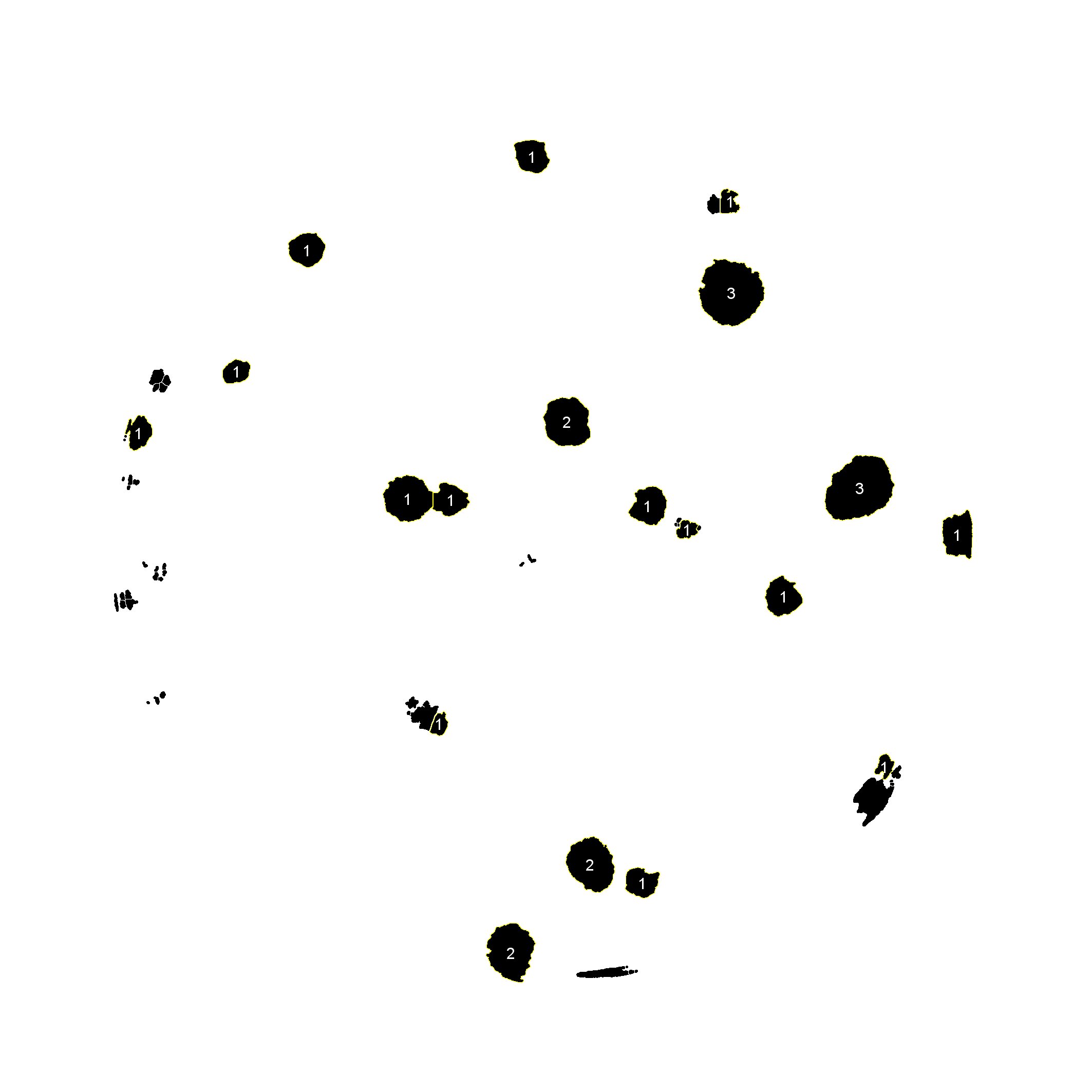

Supplement: S3 Datasets — It also contains a text file where results achieved by automated (CoCoNut, CAI, AutoCellSeg, and OpenCFU) and manual methods are summarized. (ZIP) [file pone.0205823.s004.zip › 180501 HeLa Dish/6 First counting.jpg]

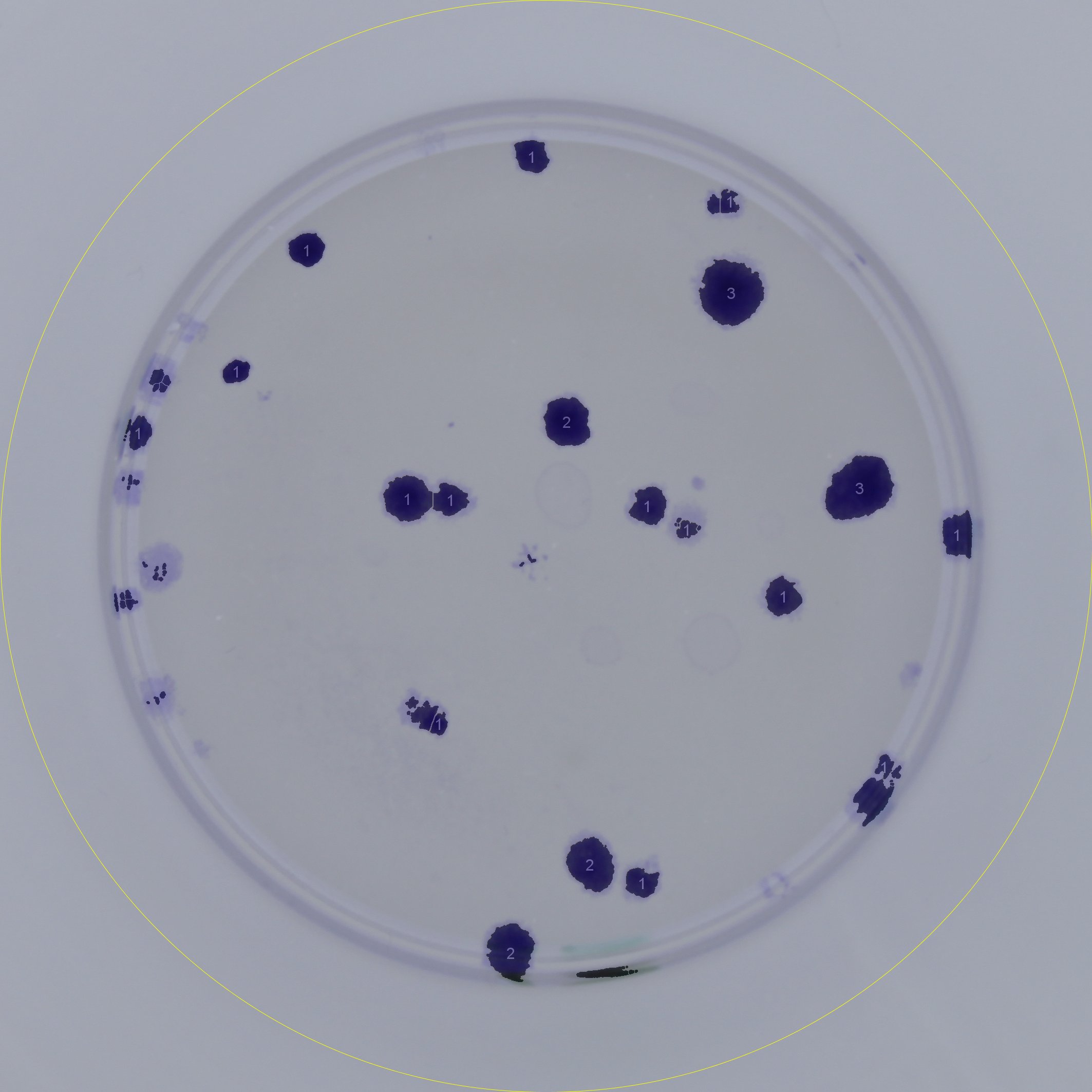

Supplement: S3 Datasets — It also contains a text file where results achieved by automated (CoCoNut, CAI, AutoCellSeg, and OpenCFU) and manual methods are summarized. (ZIP) [file pone.0205823.s004.zip › 180501 HeLa Dish/6 Results.jpg]

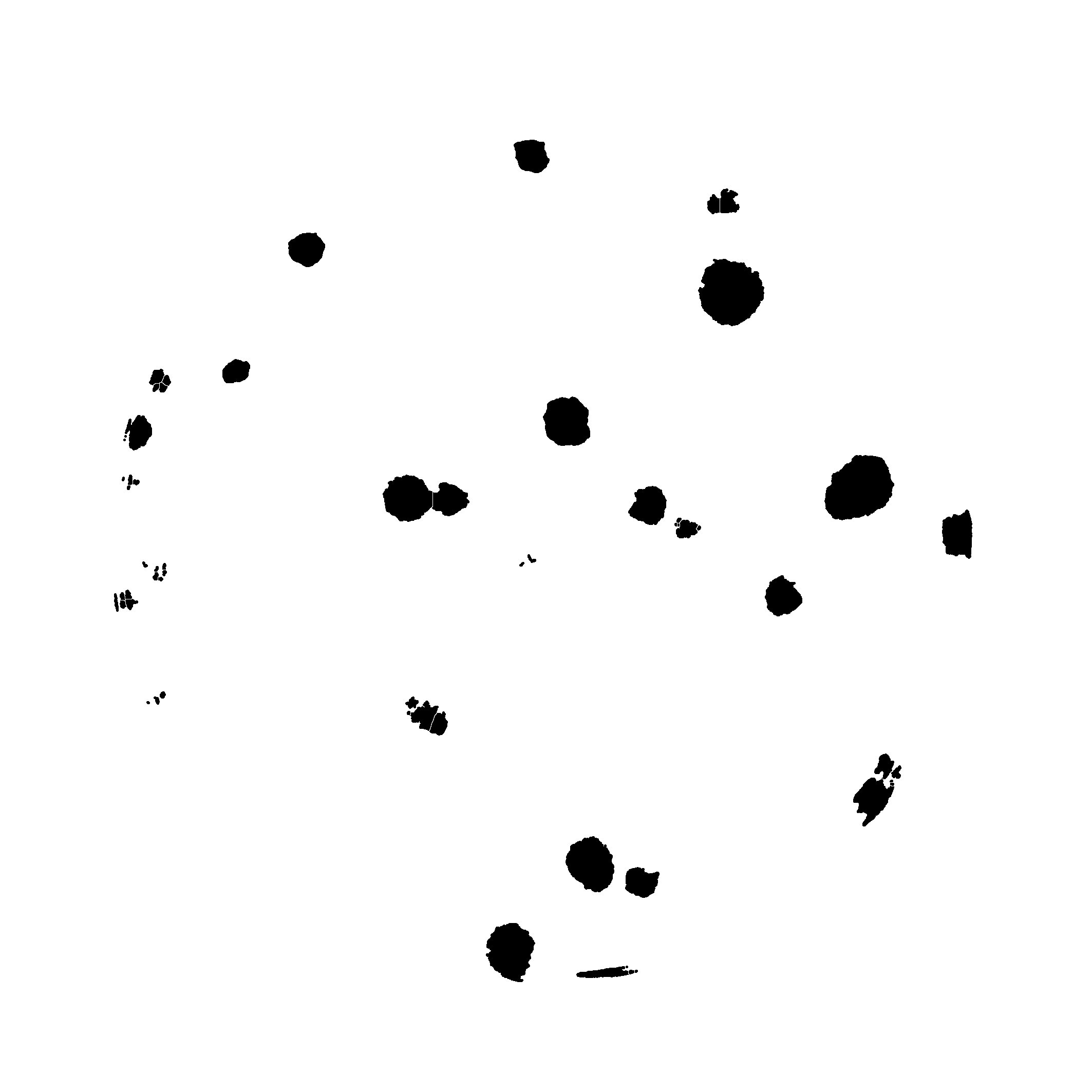

Supplement: S3 Datasets — It also contains a text file where results achieved by automated (CoCoNut, CAI, AutoCellSeg, and OpenCFU) and manual methods are summarized. (ZIP) [file pone.0205823.s004.zip › 180501 HeLa Dish/6 Second counting.jpg]

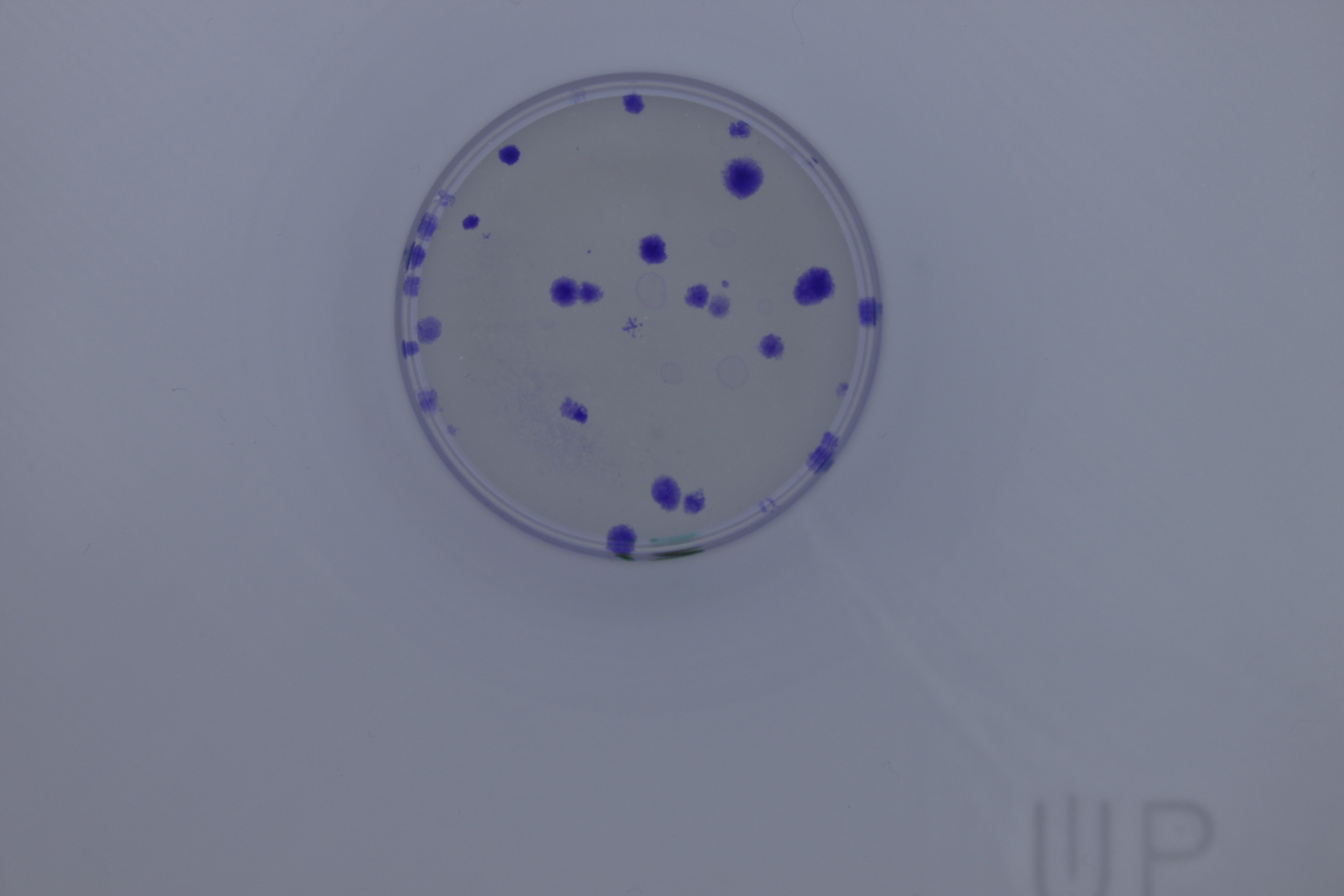

Supplement: S3 Datasets — It also contains a text file where results achieved by automated (CoCoNut, CAI, AutoCellSeg, and OpenCFU) and manual methods are summarized. (ZIP) [file pone.0205823.s004.zip › 180501 HeLa Dish/6.JPG]

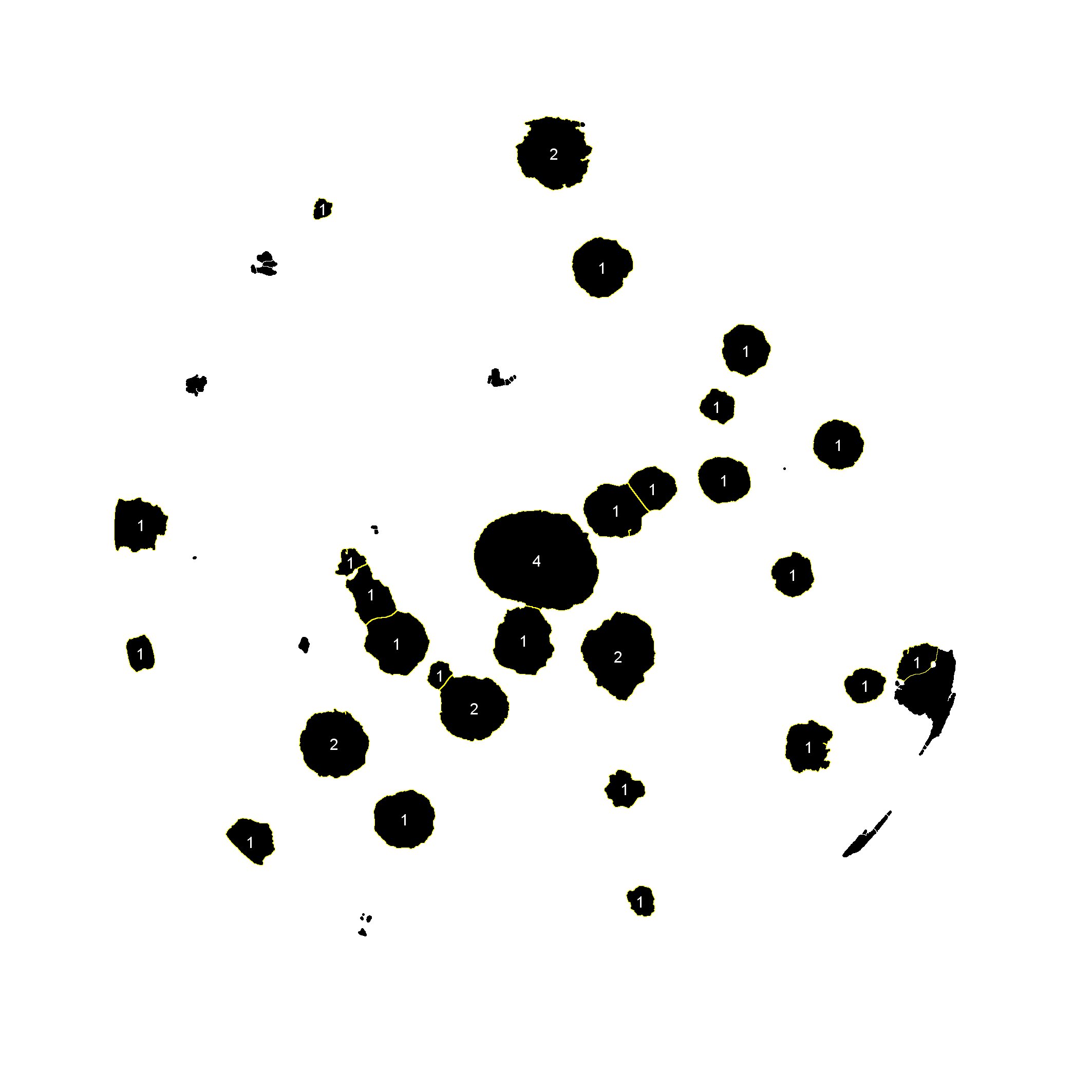

Supplement: S3 Datasets — It also contains a text file where results achieved by automated (CoCoNut, CAI, AutoCellSeg, and OpenCFU) and manual methods are summarized. (ZIP) [file pone.0205823.s004.zip › 180501 HeLa Dish/7 First counting.jpg]

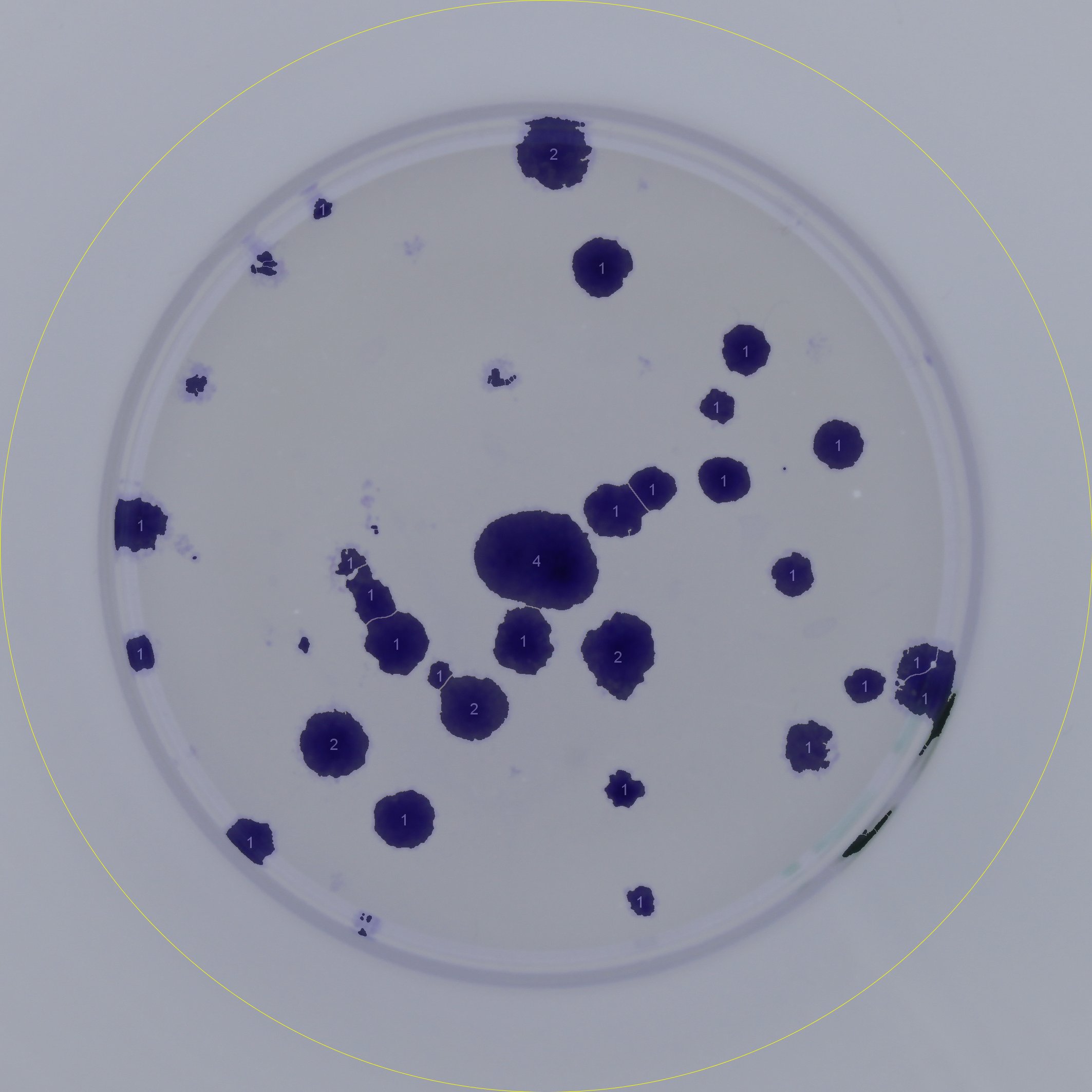

Supplement: S3 Datasets — It also contains a text file where results achieved by automated (CoCoNut, CAI, AutoCellSeg, and OpenCFU) and manual methods are summarized. (ZIP) [file pone.0205823.s004.zip › 180501 HeLa Dish/7 Results.jpg]

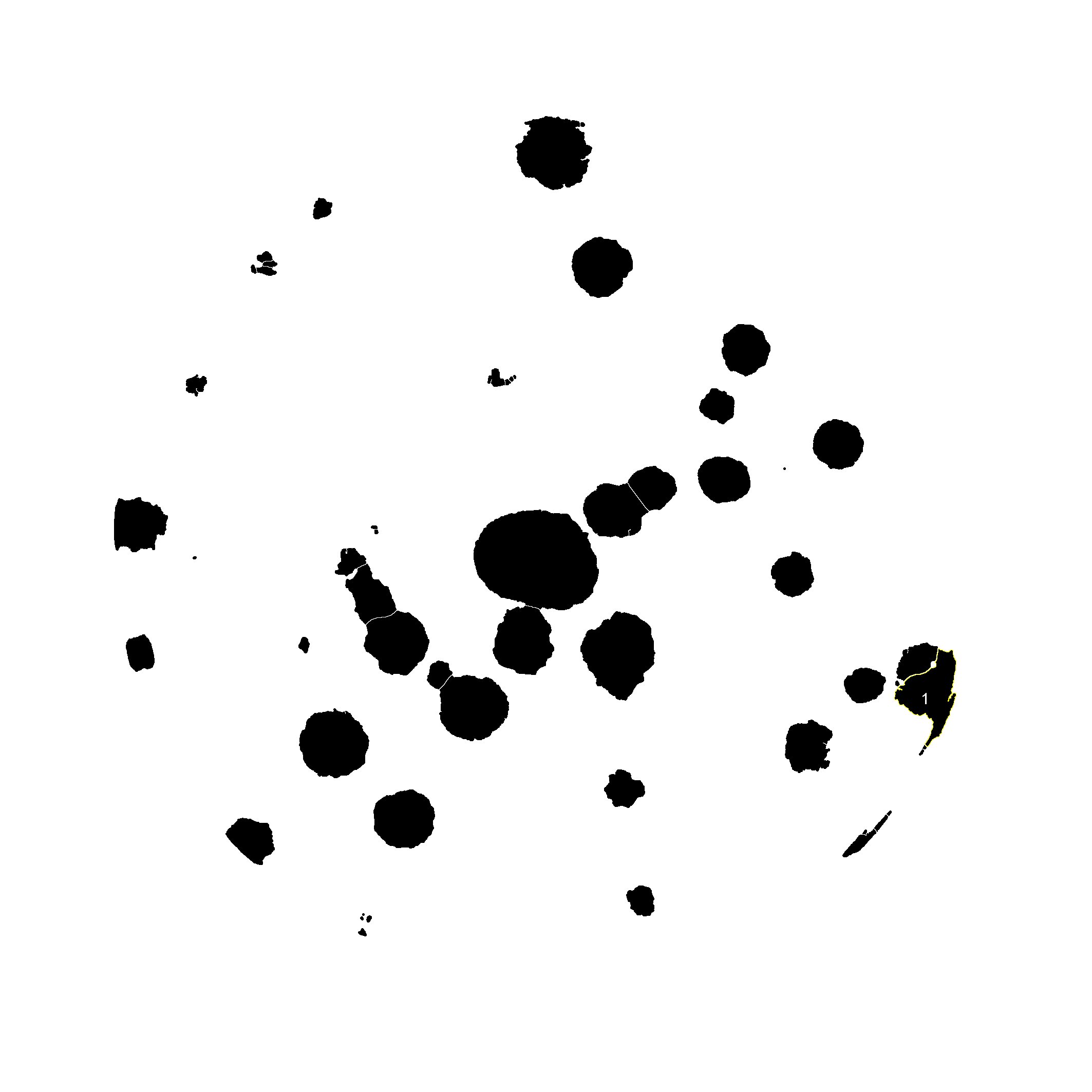

Supplement: S3 Datasets — It also contains a text file where results achieved by automated (CoCoNut, CAI, AutoCellSeg, and OpenCFU) and manual methods are summarized. (ZIP) [file pone.0205823.s004.zip › 180501 HeLa Dish/7 Second counting.jpg]

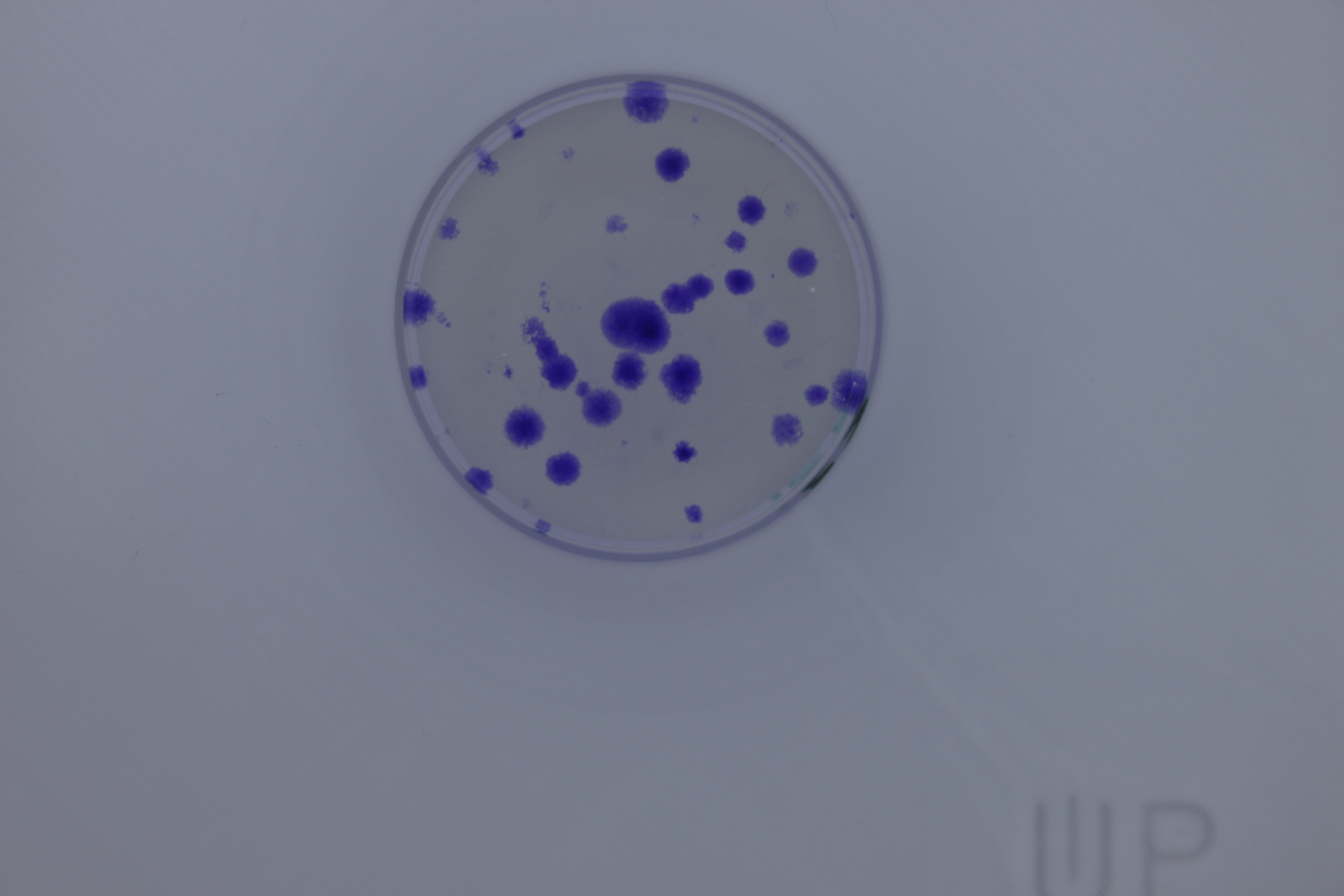

Supplement: S3 Datasets — It also contains a text file where results achieved by automated (CoCoNut, CAI, AutoCellSeg, and OpenCFU) and manual methods are summarized. (ZIP) [file pone.0205823.s004.zip › 180501 HeLa Dish/7.JPG]

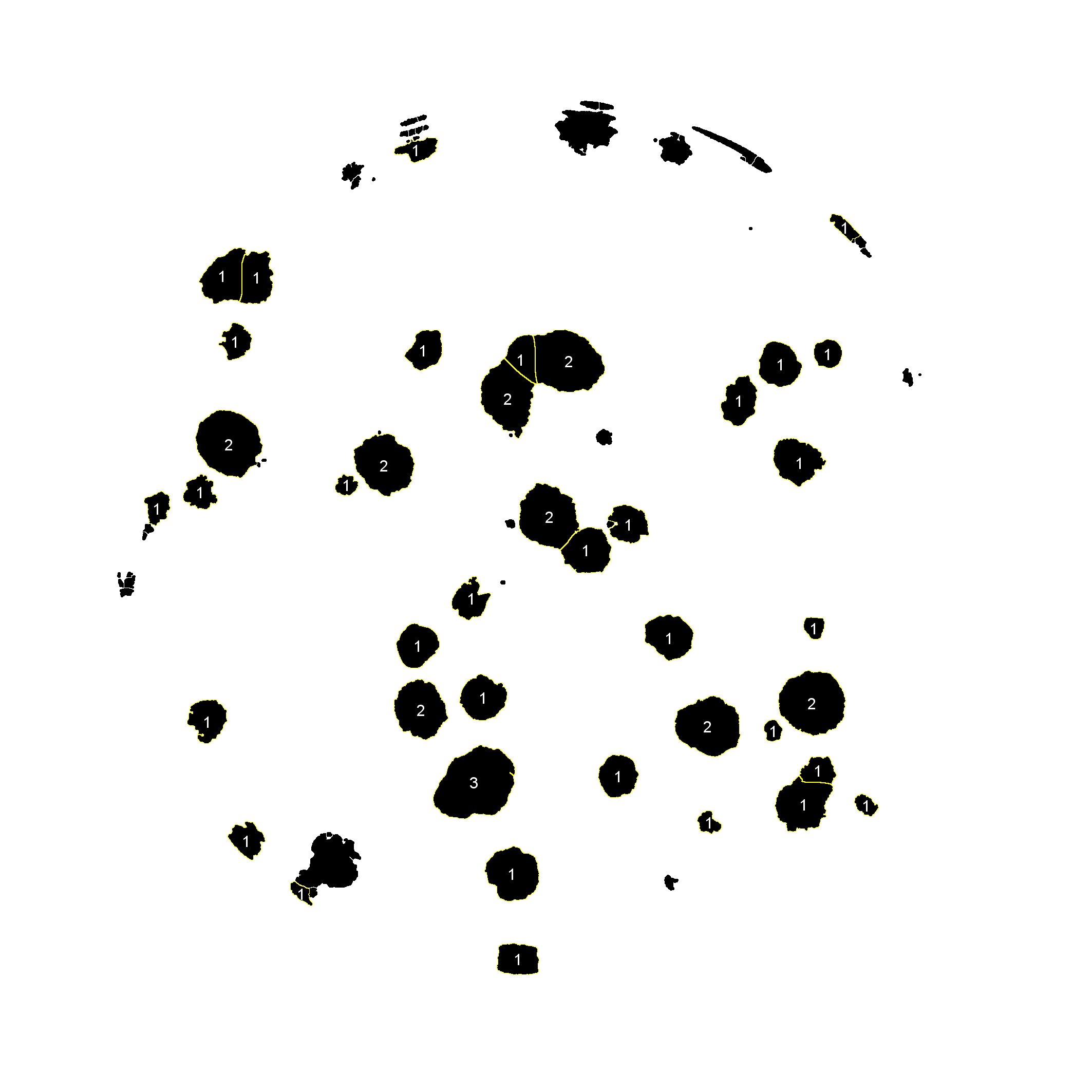

Supplement: S3 Datasets — It also contains a text file where results achieved by automated (CoCoNut, CAI, AutoCellSeg, and OpenCFU) and manual methods are summarized. (ZIP) [file pone.0205823.s004.zip › 180501 HeLa Dish/8 First counting.jpg]

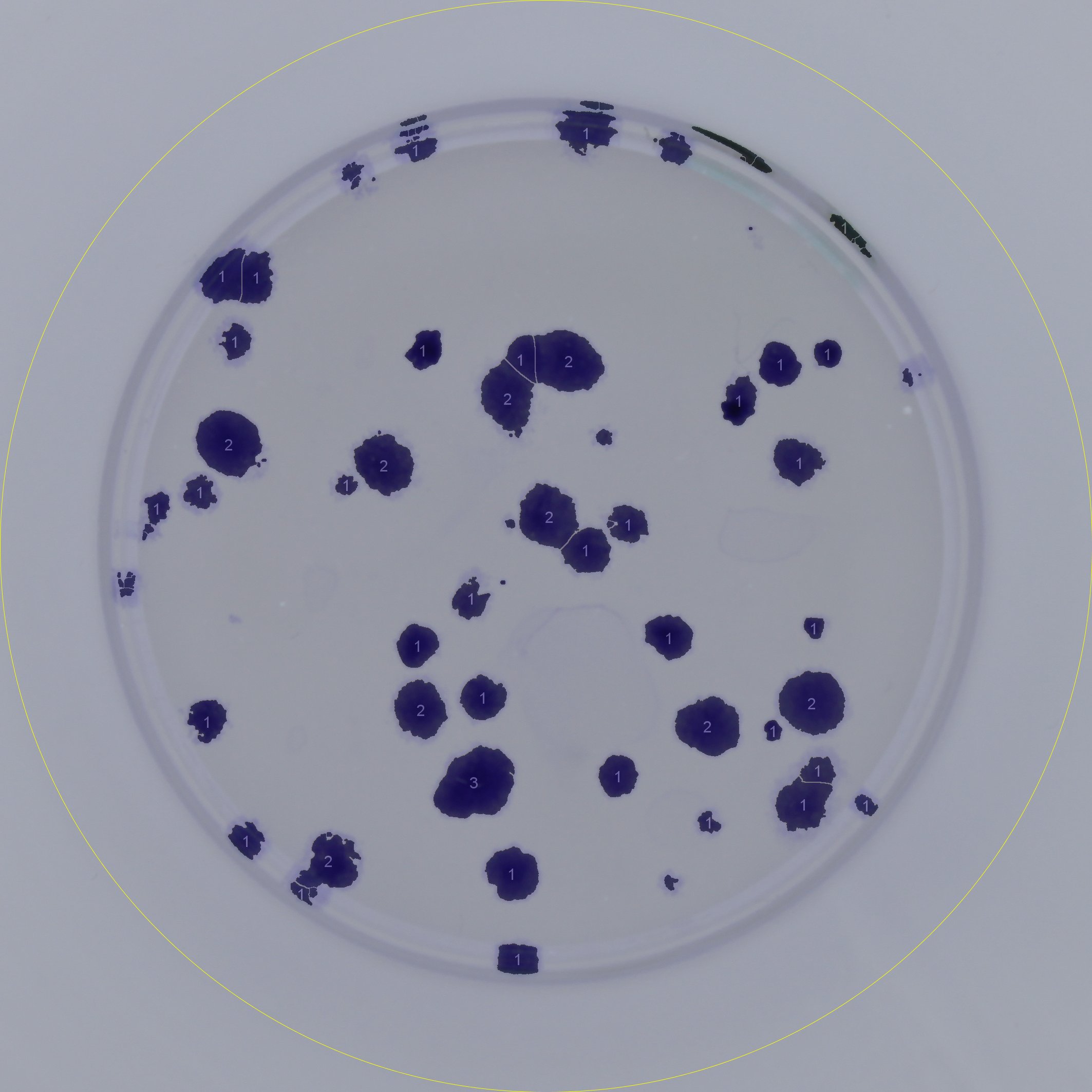

Supplement: S3 Datasets — It also contains a text file where results achieved by automated (CoCoNut, CAI, AutoCellSeg, and OpenCFU) and manual methods are summarized. (ZIP) [file pone.0205823.s004.zip › 180501 HeLa Dish/8 Results.jpg]

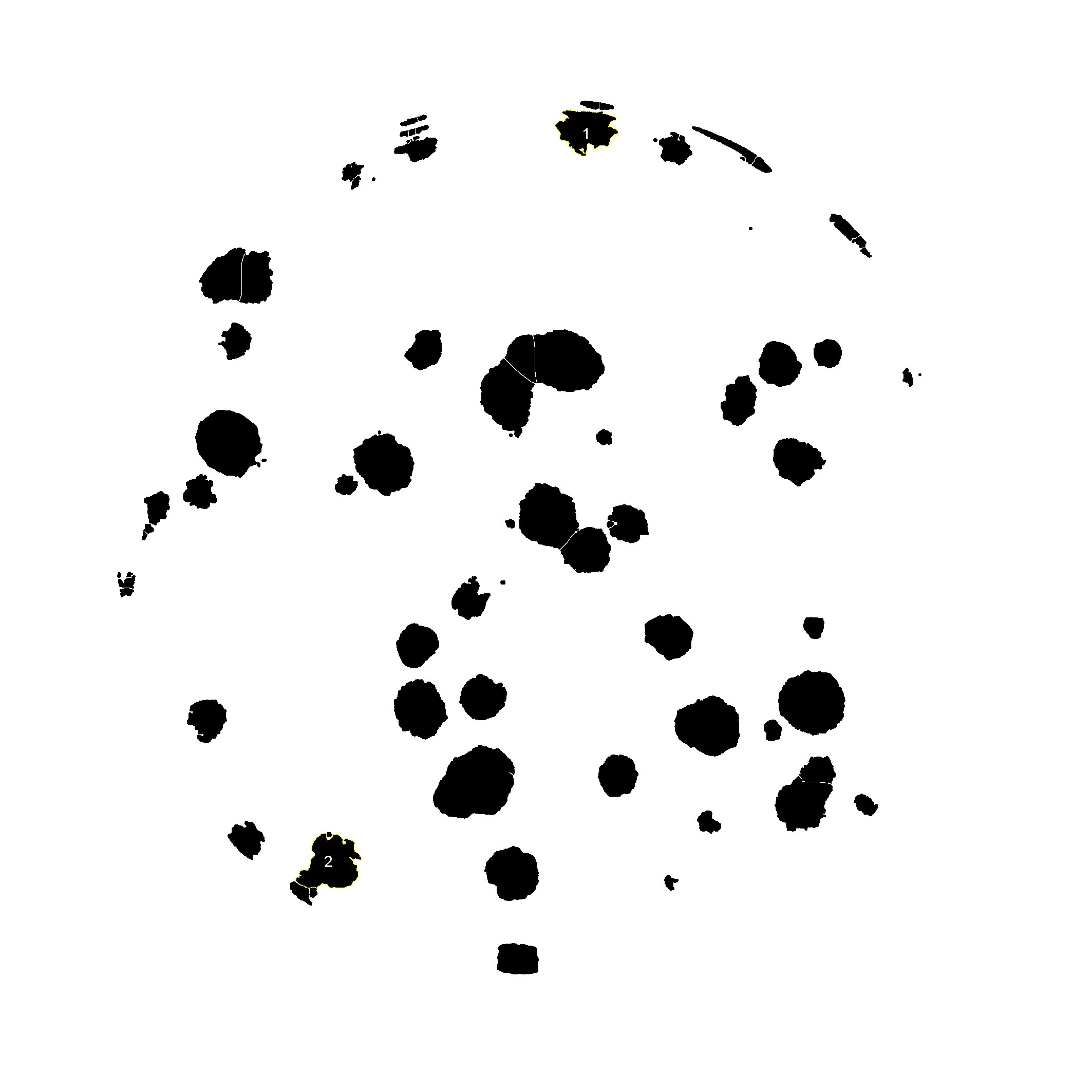

Supplement: S3 Datasets — It also contains a text file where results achieved by automated (CoCoNut, CAI, AutoCellSeg, and OpenCFU) and manual methods are summarized. (ZIP) [file pone.0205823.s004.zip › 180501 HeLa Dish/8 Second counting.jpg]

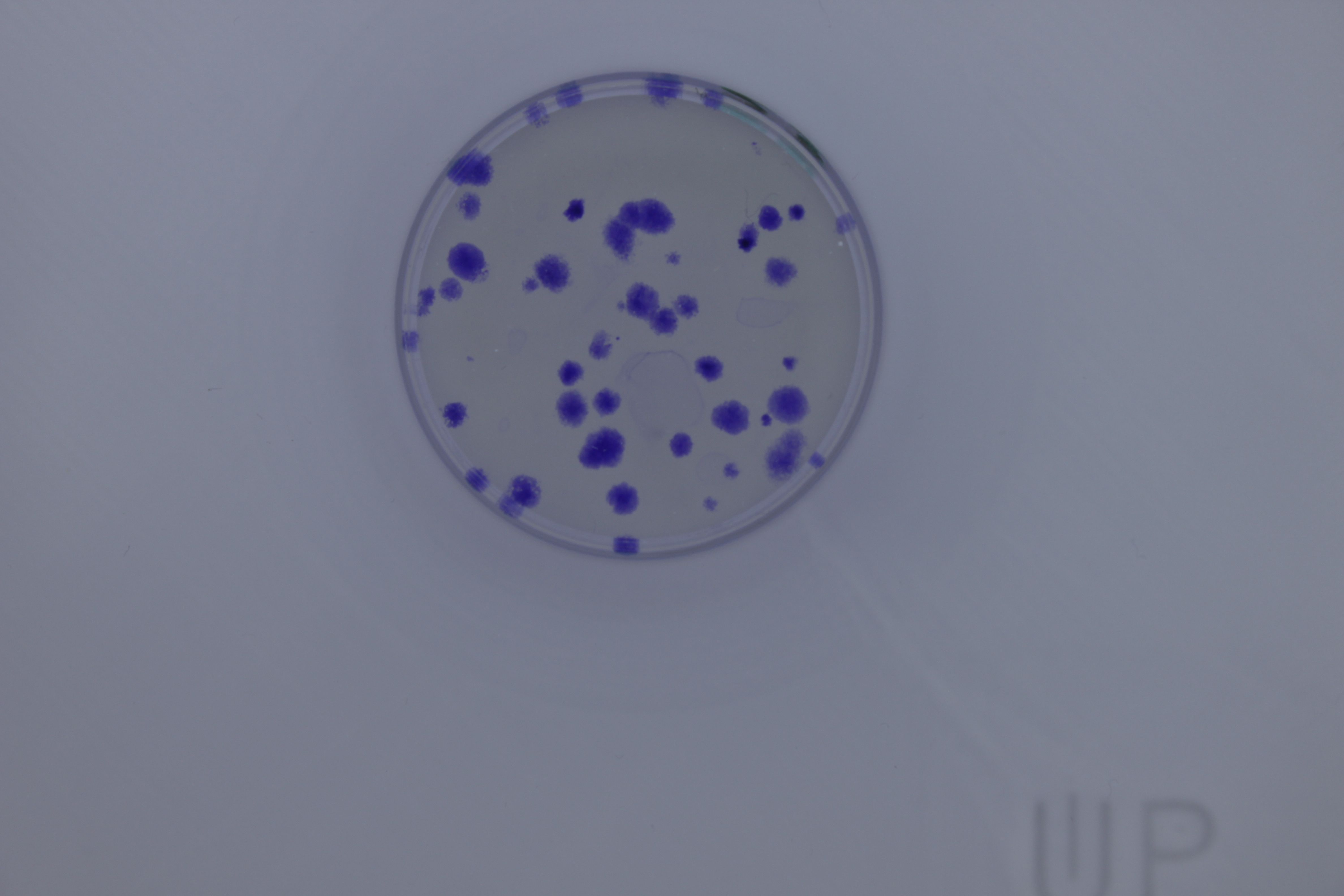

Supplement: S3 Datasets — It also contains a text file where results achieved by automated (CoCoNut, CAI, AutoCellSeg, and OpenCFU) and manual methods are summarized. (ZIP) [file pone.0205823.s004.zip › 180501 HeLa Dish/8.JPG]

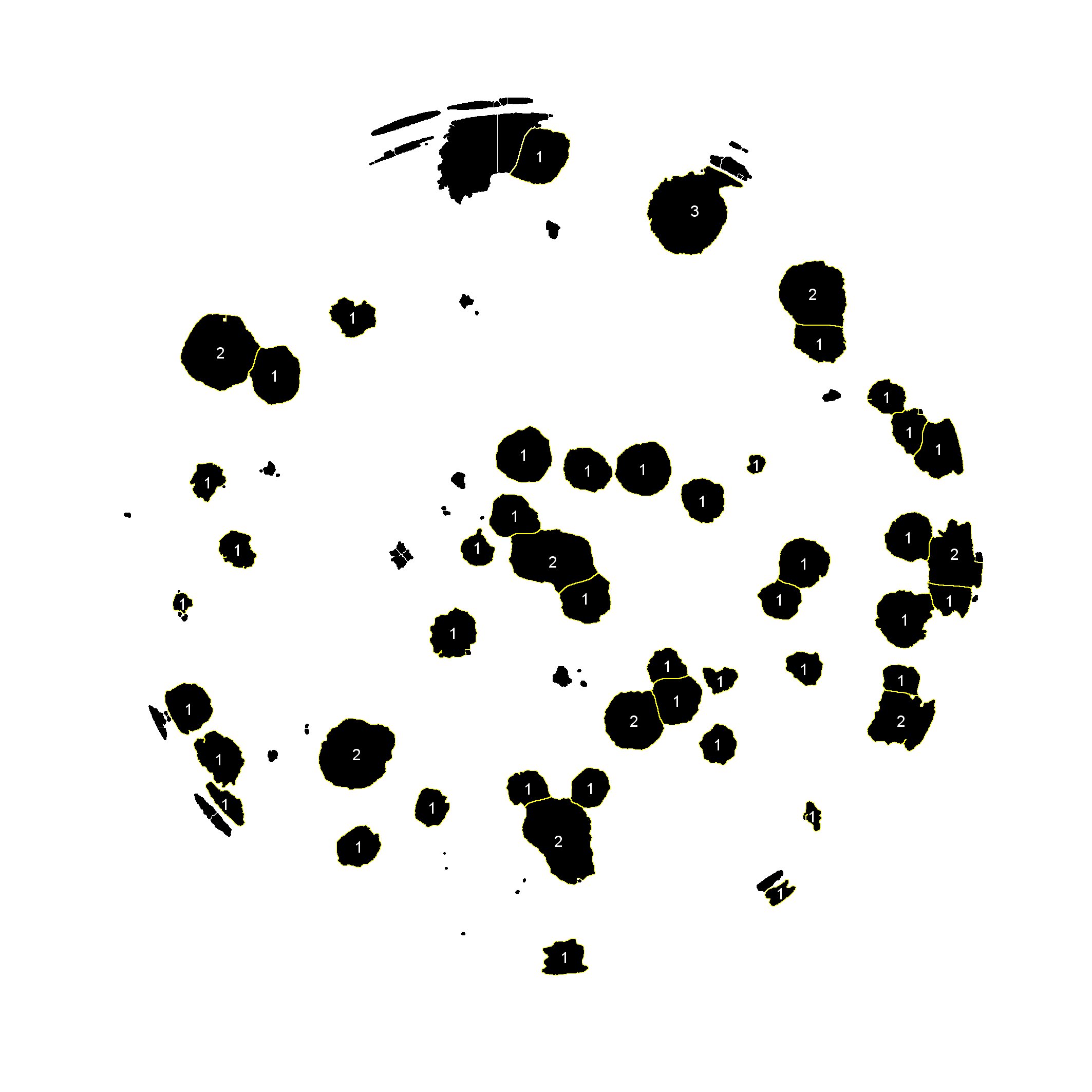

Supplement: S3 Datasets — It also contains a text file where results achieved by automated (CoCoNut, CAI, AutoCellSeg, and OpenCFU) and manual methods are summarized. (ZIP) [file pone.0205823.s004.zip › 180501 HeLa Dish/9 First counting.jpg]

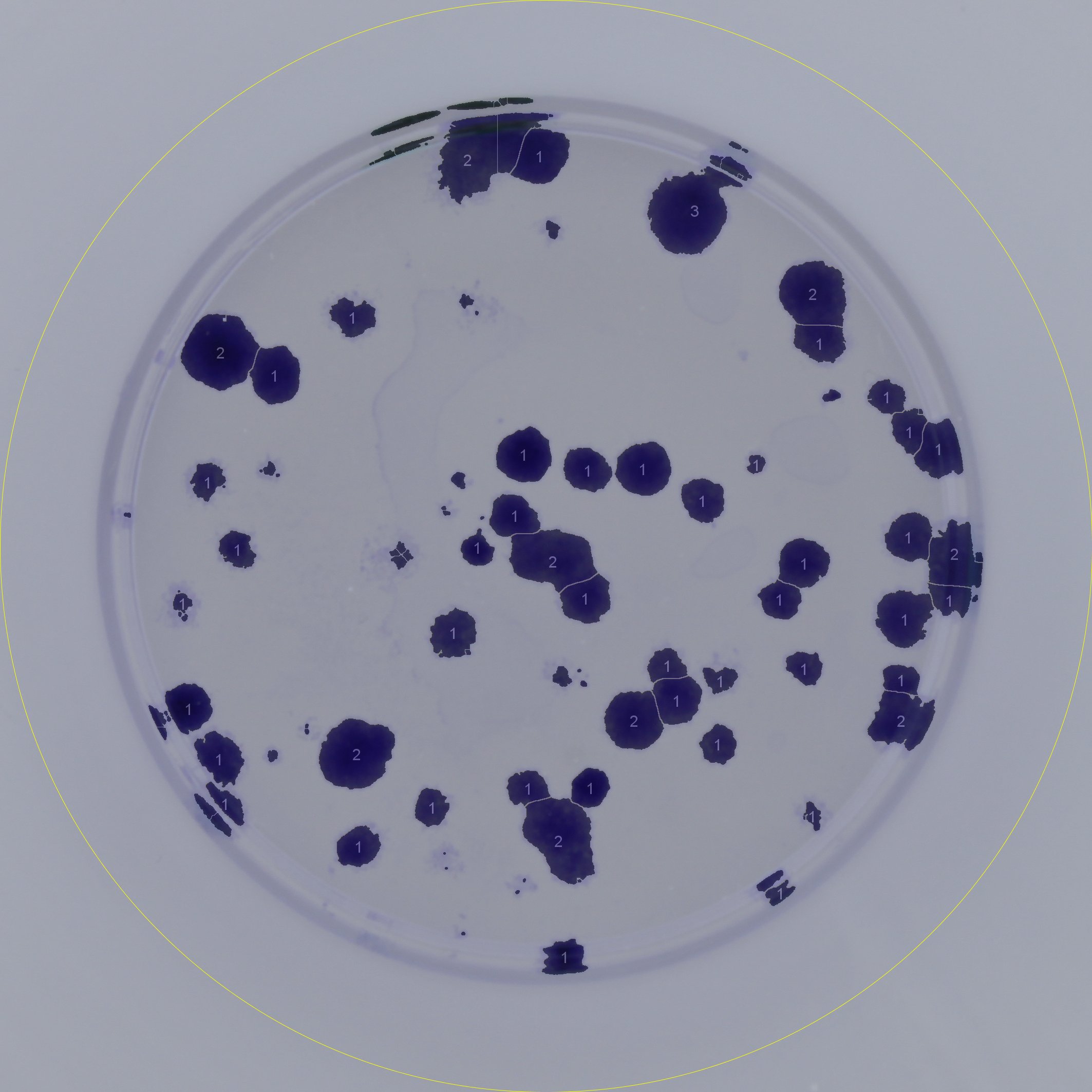

Supplement: S3 Datasets — It also contains a text file where results achieved by automated (CoCoNut, CAI, AutoCellSeg, and OpenCFU) and manual methods are summarized. (ZIP) [file pone.0205823.s004.zip › 180501 HeLa Dish/9 Results.jpg]

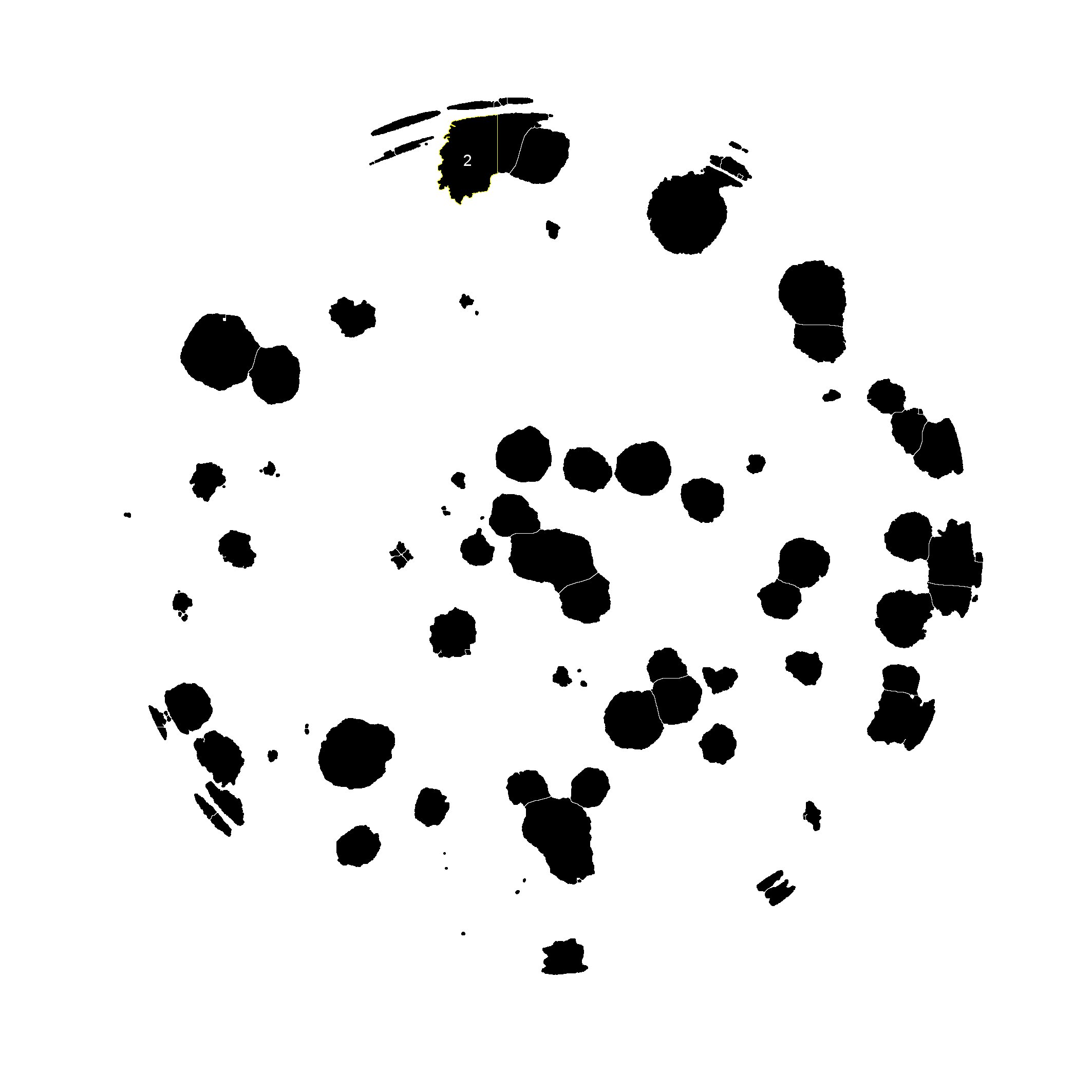

Supplement: S3 Datasets — It also contains a text file where results achieved by automated (CoCoNut, CAI, AutoCellSeg, and OpenCFU) and manual methods are summarized. (ZIP) [file pone.0205823.s004.zip › 180501 HeLa Dish/9 Second counting.jpg]

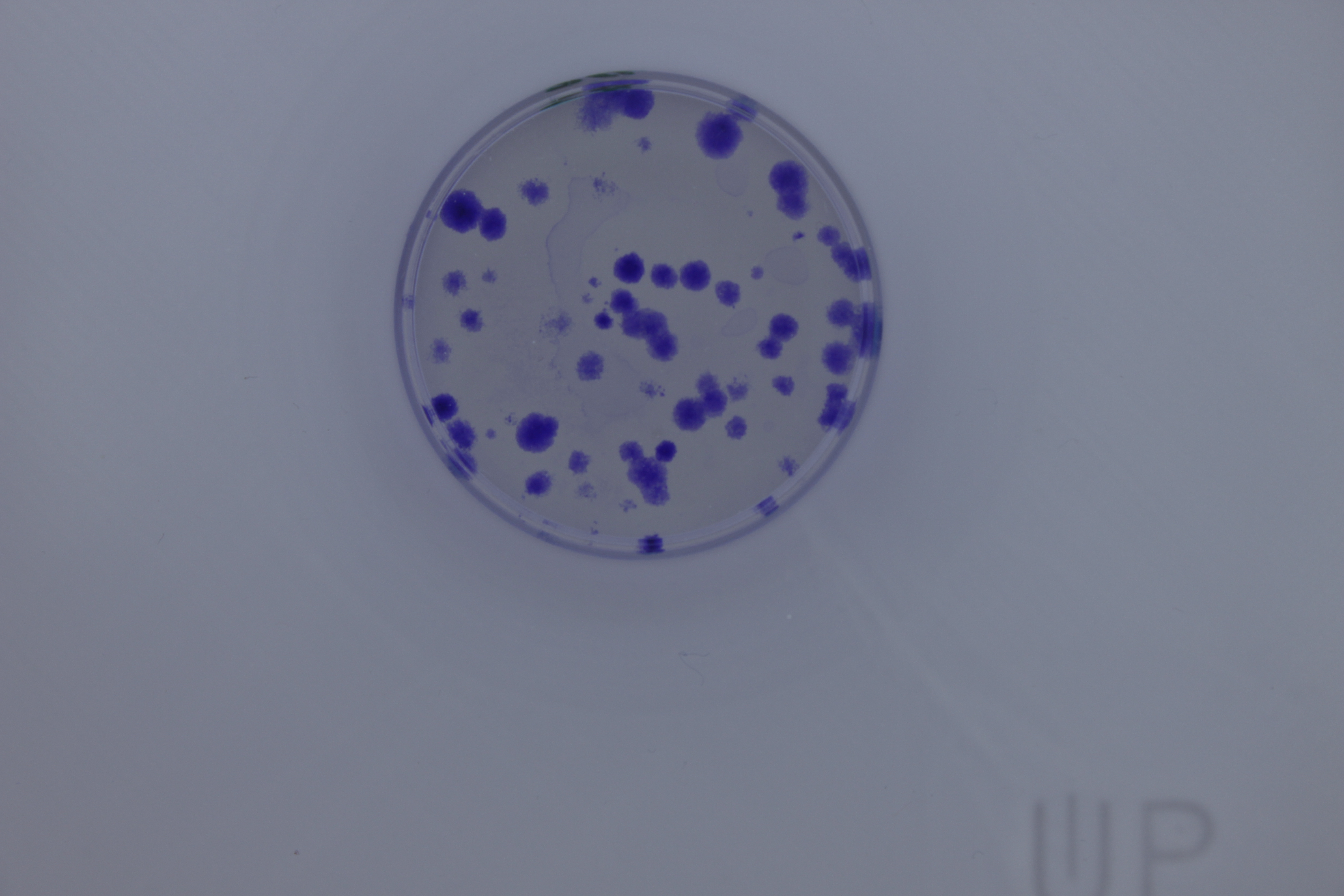

Supplement: S3 Datasets — It also contains a text file where results achieved by automated (CoCoNut, CAI, AutoCellSeg, and OpenCFU) and manual methods are summarized. (ZIP) [file pone.0205823.s004.zip › 180501 HeLa Dish/9.JPG]

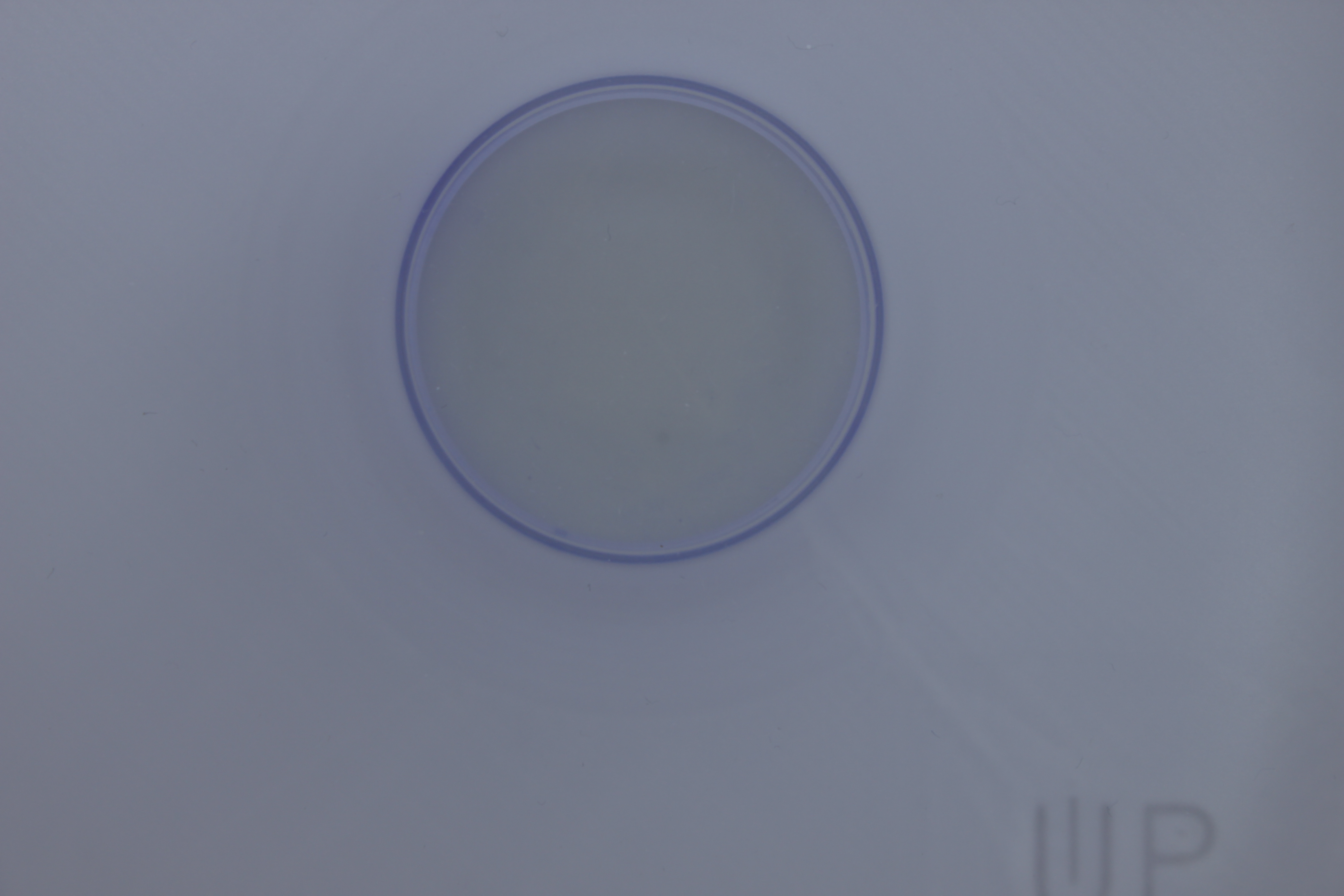

Supplement: S3 Datasets — It also contains a text file where results achieved by automated (CoCoNut, CAI, AutoCellSeg, and OpenCFU) and manual methods are summarized. (ZIP) [file pone.0205823.s004.zip › 180501 HeLa Dish/background.JPG]

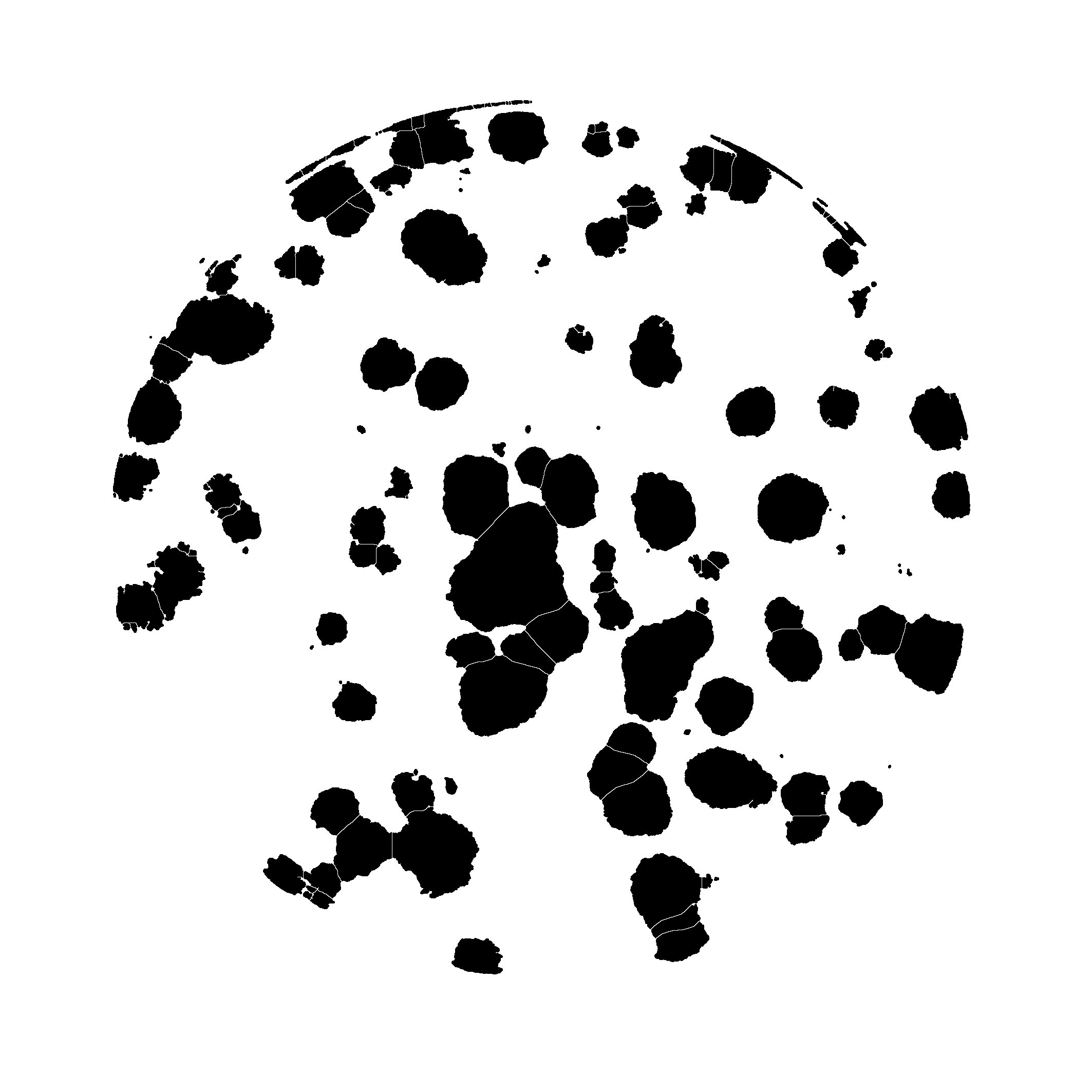

Supplement: S3 Datasets — It also contains a text file where results achieved by automated (CoCoNut, CAI, AutoCellSeg, and OpenCFU) and manual methods are summarized. (ZIP) [file pone.0205823.s004.zip › 180501 HeLa Dish/binary10.jpg]

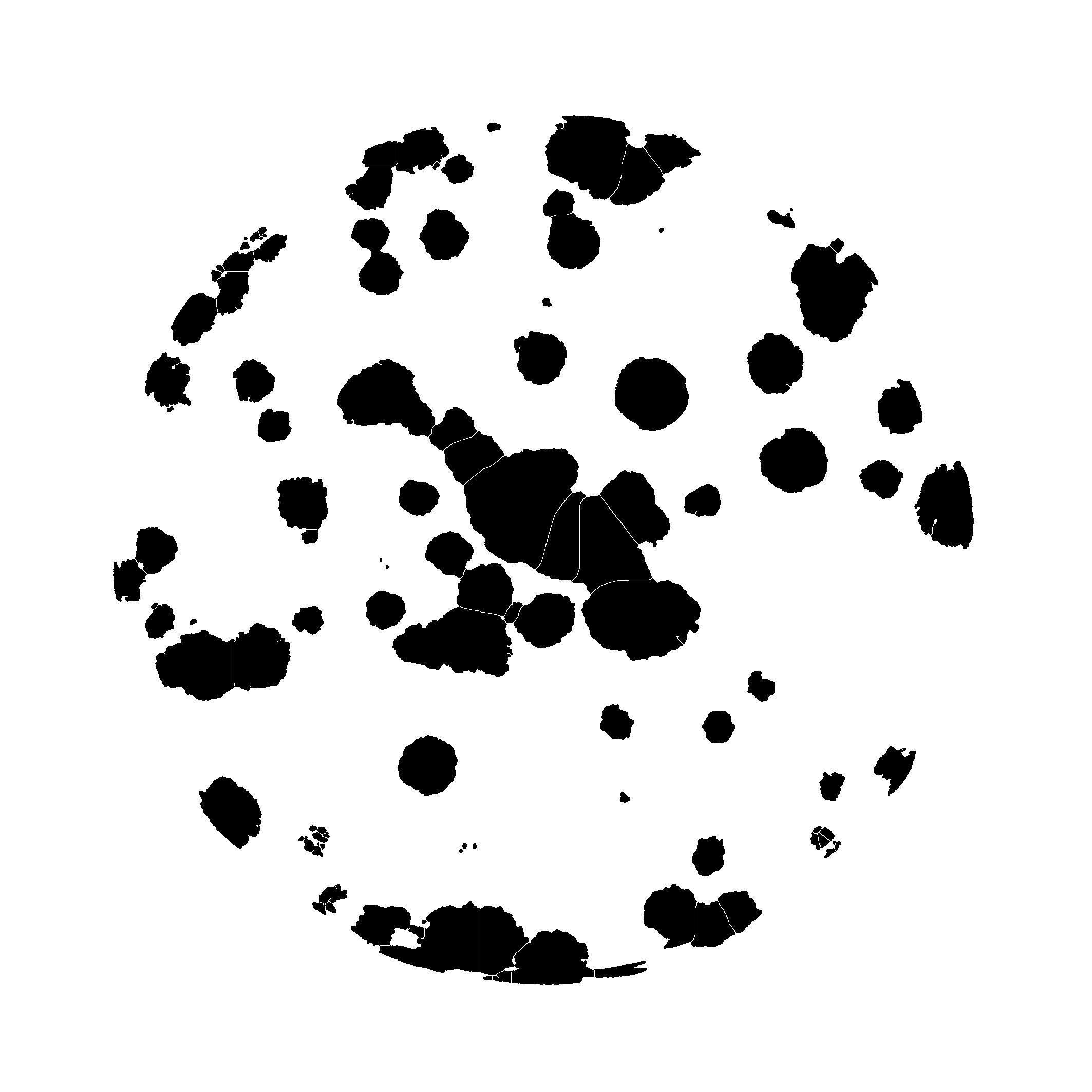

Supplement: S3 Datasets — It also contains a text file where results achieved by automated (CoCoNut, CAI, AutoCellSeg, and OpenCFU) and manual methods are summarized. (ZIP) [file pone.0205823.s004.zip › 180501 HeLa Dish/binary11.jpg]

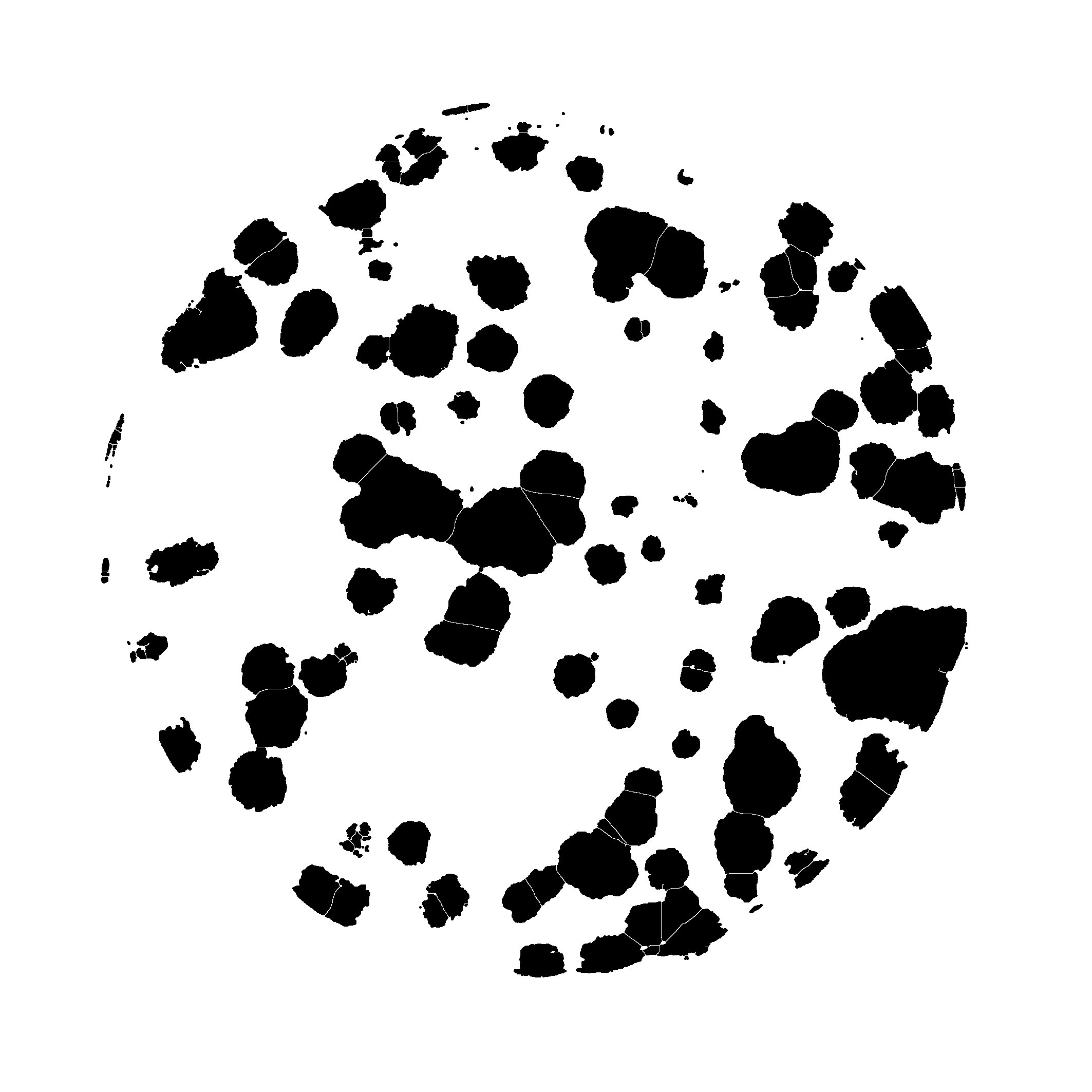

Supplement: S3 Datasets — It also contains a text file where results achieved by automated (CoCoNut, CAI, AutoCellSeg, and OpenCFU) and manual methods are summarized. (ZIP) [file pone.0205823.s004.zip › 180501 HeLa Dish/binary12.jpg]

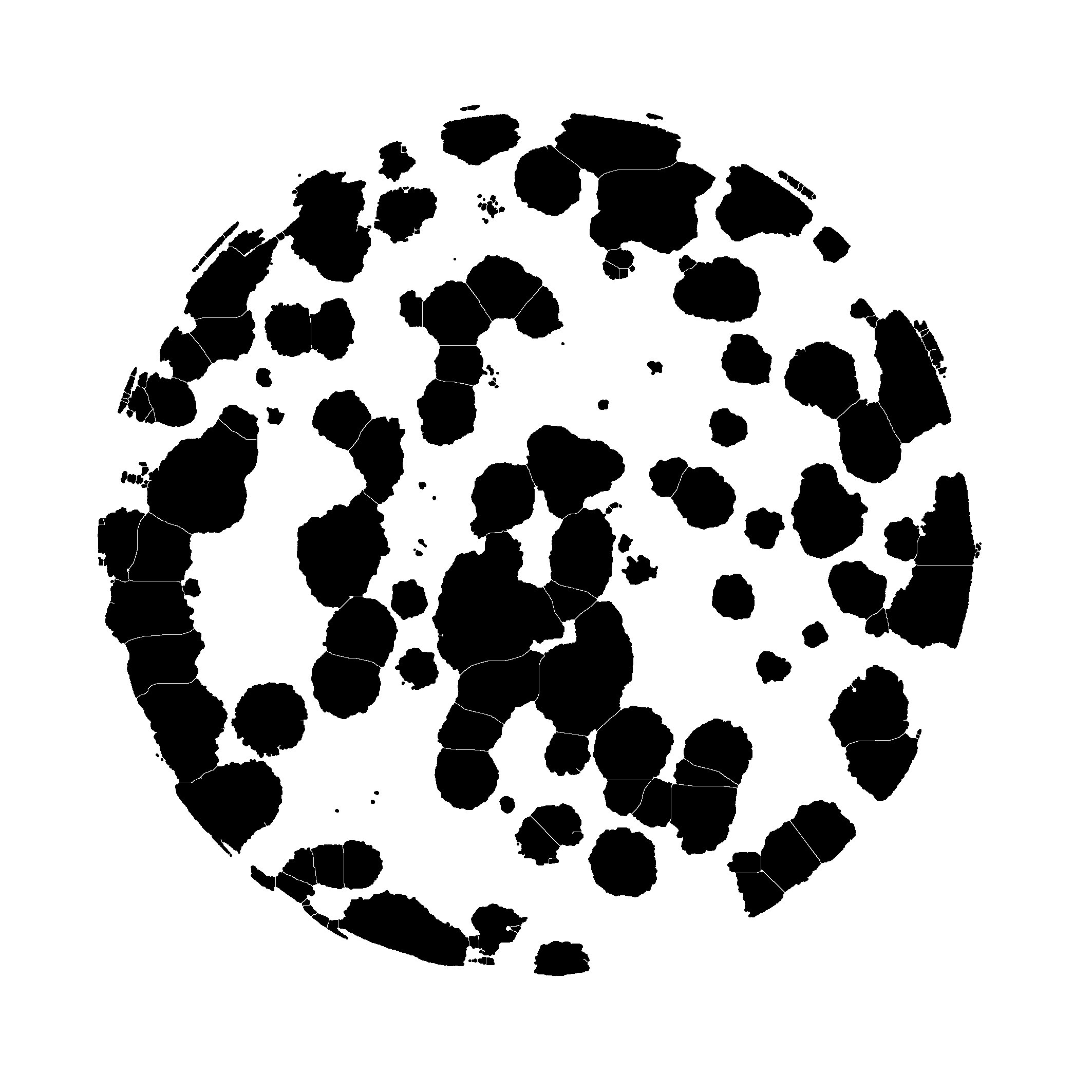

Supplement: S3 Datasets — It also contains a text file where results achieved by automated (CoCoNut, CAI, AutoCellSeg, and OpenCFU) and manual methods are summarized. (ZIP) [file pone.0205823.s004.zip › 180501 HeLa Dish/binary13.jpg]

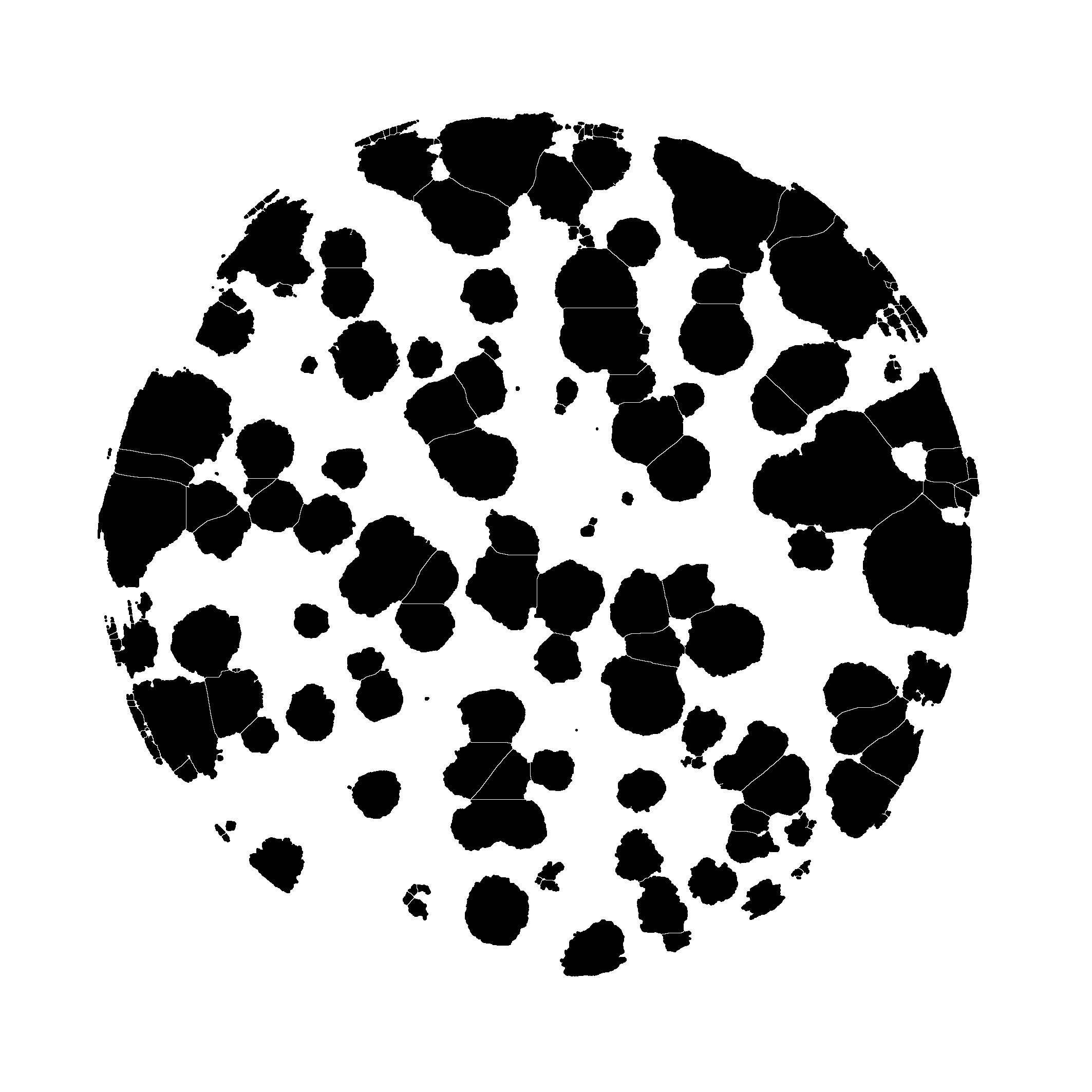

Supplement: S3 Datasets — It also contains a text file where results achieved by automated (CoCoNut, CAI, AutoCellSeg, and OpenCFU) and manual methods are summarized. (ZIP) [file pone.0205823.s004.zip › 180501 HeLa Dish/binary14.jpg]

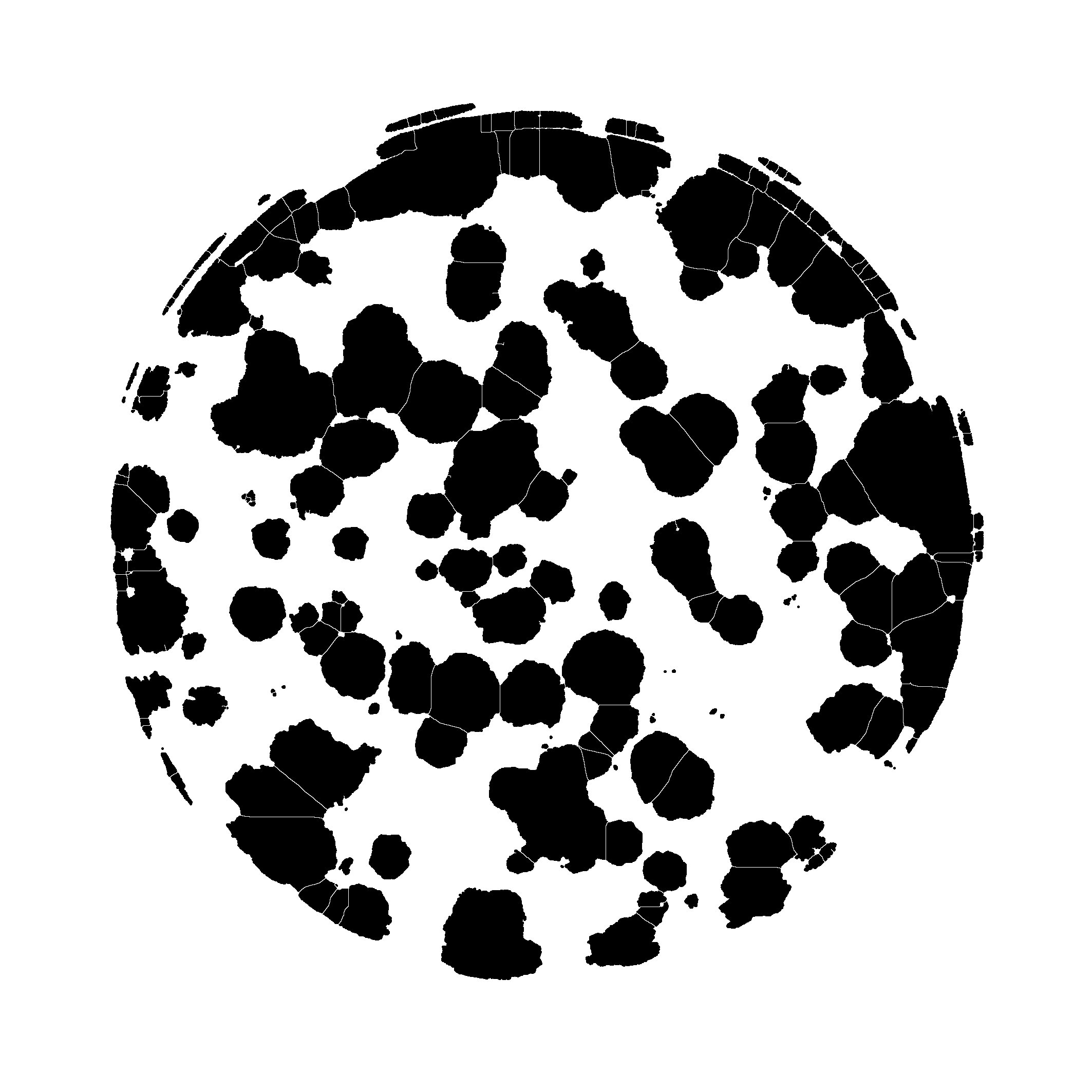

Supplement: S3 Datasets — It also contains a text file where results achieved by automated (CoCoNut, CAI, AutoCellSeg, and OpenCFU) and manual methods are summarized. (ZIP) [file pone.0205823.s004.zip › 180501 HeLa Dish/binary15.jpg]

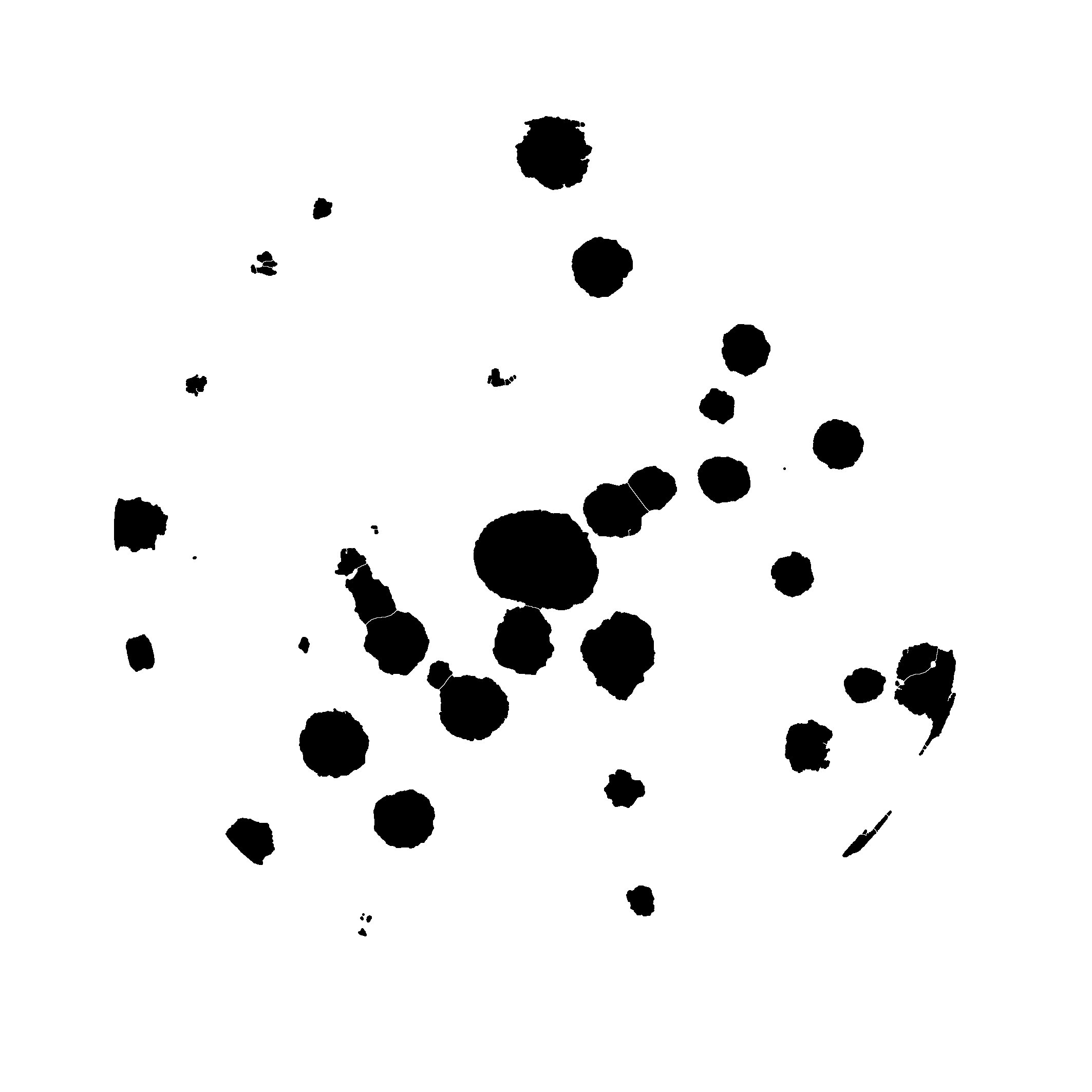

Supplement: S3 Datasets — It also contains a text file where results achieved by automated (CoCoNut, CAI, AutoCellSeg, and OpenCFU) and manual methods are summarized. (ZIP) [file pone.0205823.s004.zip › 180501 HeLa Dish/binary7.jpg]

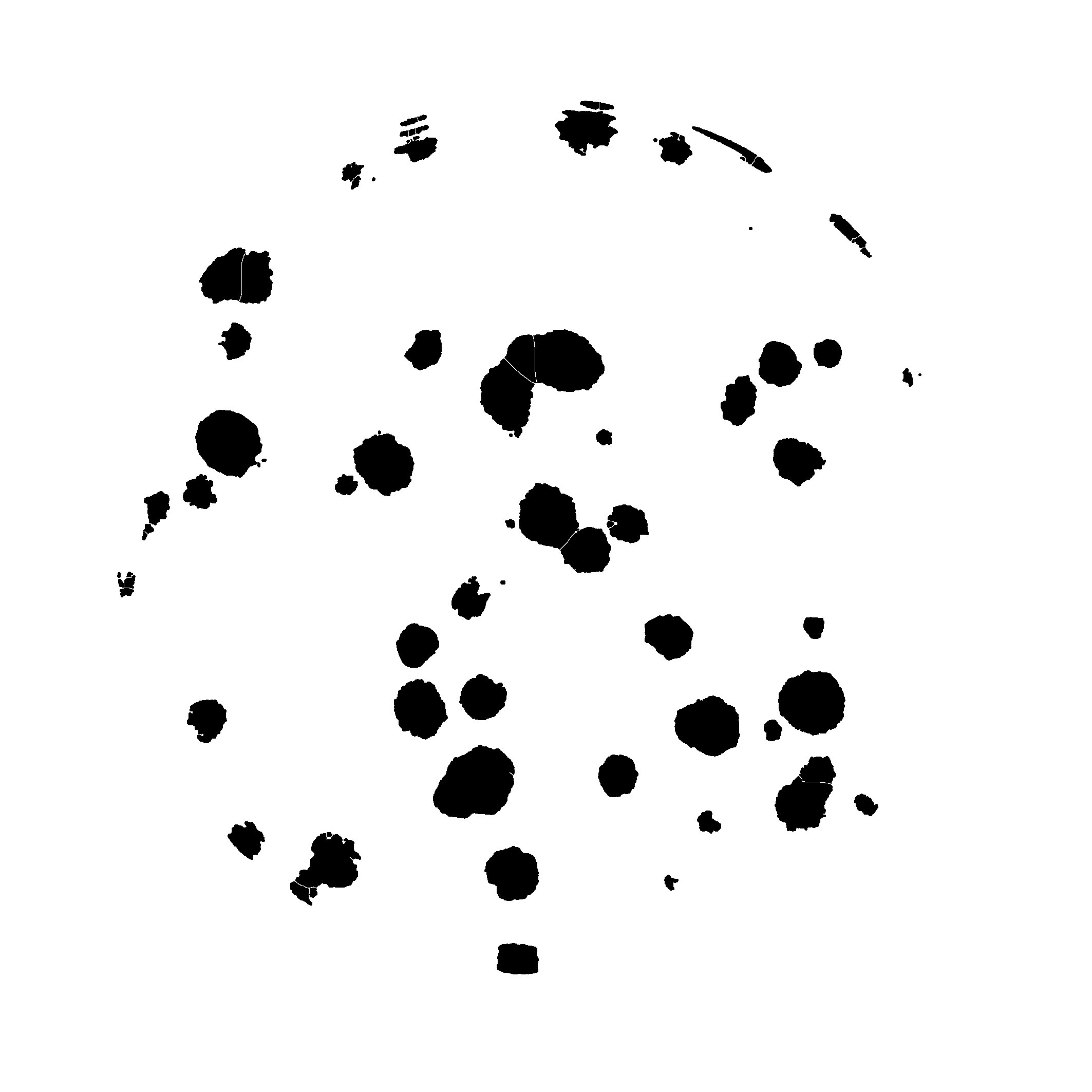

Supplement: S3 Datasets — It also contains a text file where results achieved by automated (CoCoNut, CAI, AutoCellSeg, and OpenCFU) and manual methods are summarized. (ZIP) [file pone.0205823.s004.zip › 180501 HeLa Dish/binary8.jpg]

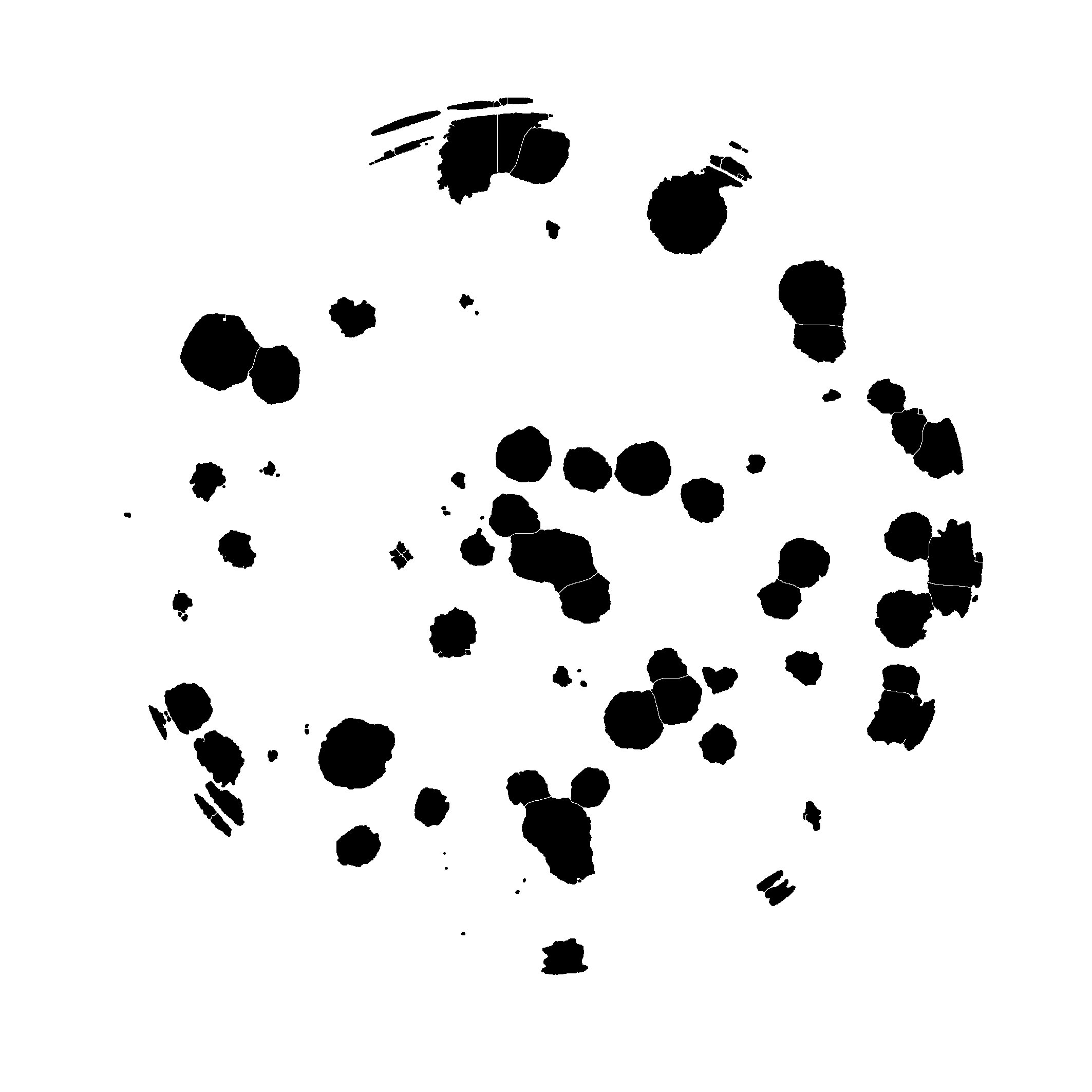

Supplement: S3 Datasets — It also contains a text file where results achieved by automated (CoCoNut, CAI, AutoCellSeg, and OpenCFU) and manual methods are summarized. (ZIP) [file pone.0205823.s004.zip › 180501 HeLa Dish/binary9.jpg]

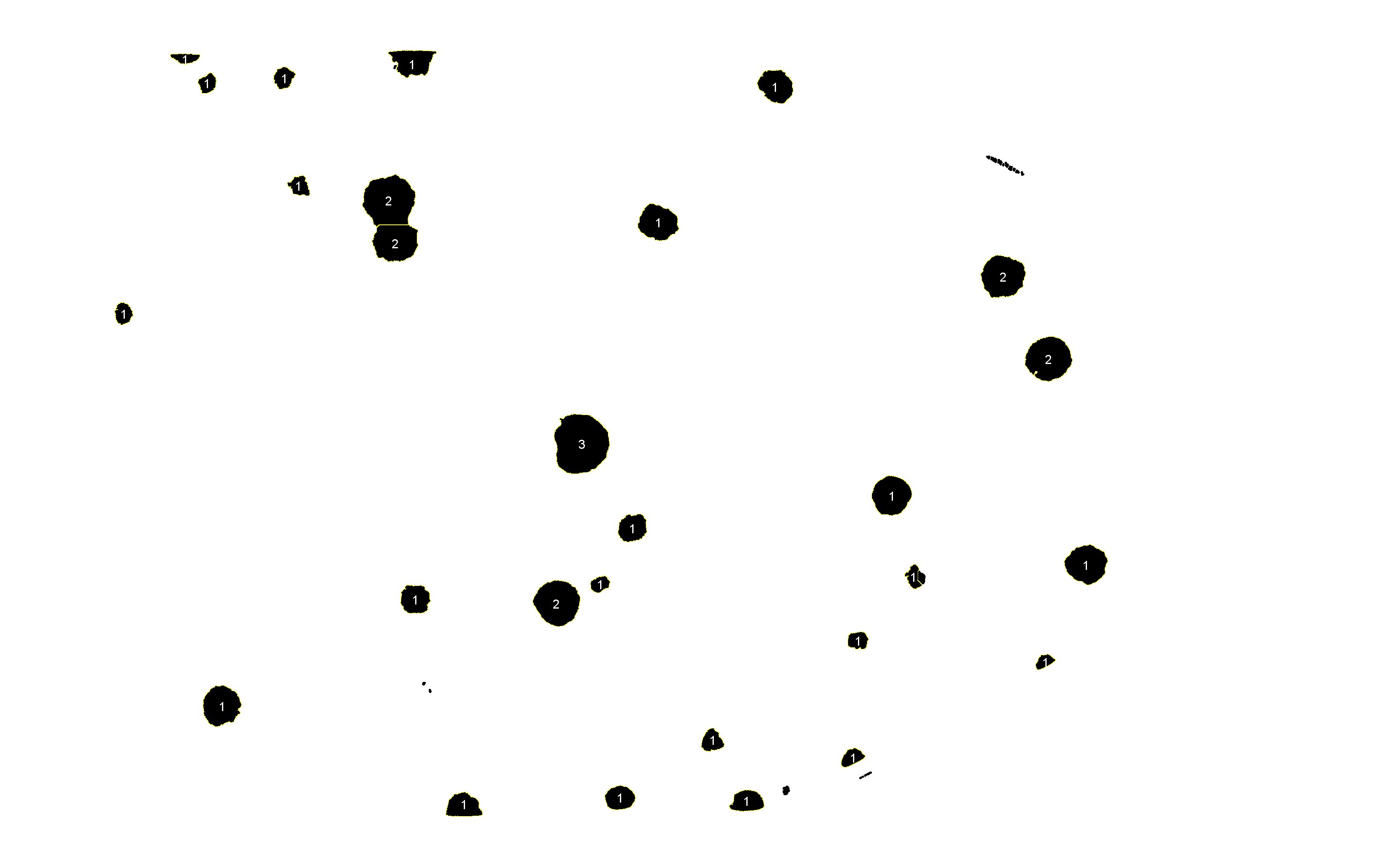

Supplement: S4 Datasets — It also contains a text file where results achieved by automated (CoCoNut, CAI, AutoCellSeg, and OpenCFU) and manual methods are summarized. (ZIP) [file pone.0205823.s005.zip › 180501 HeLa Flask/1 First counting.jpg]

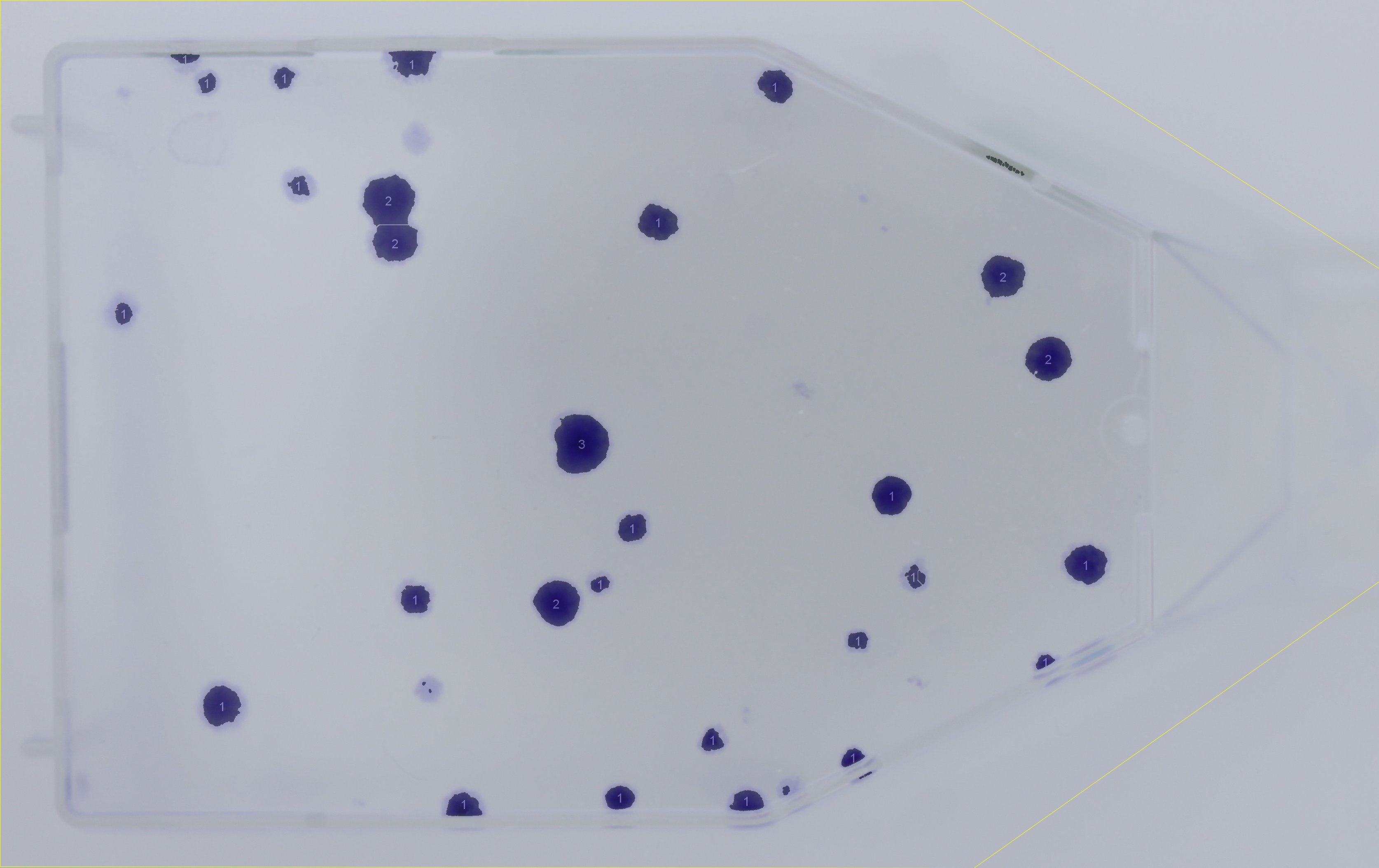

Supplement: S4 Datasets — It also contains a text file where results achieved by automated (CoCoNut, CAI, AutoCellSeg, and OpenCFU) and manual methods are summarized. (ZIP) [file pone.0205823.s005.zip › 180501 HeLa Flask/1 Results.jpg]

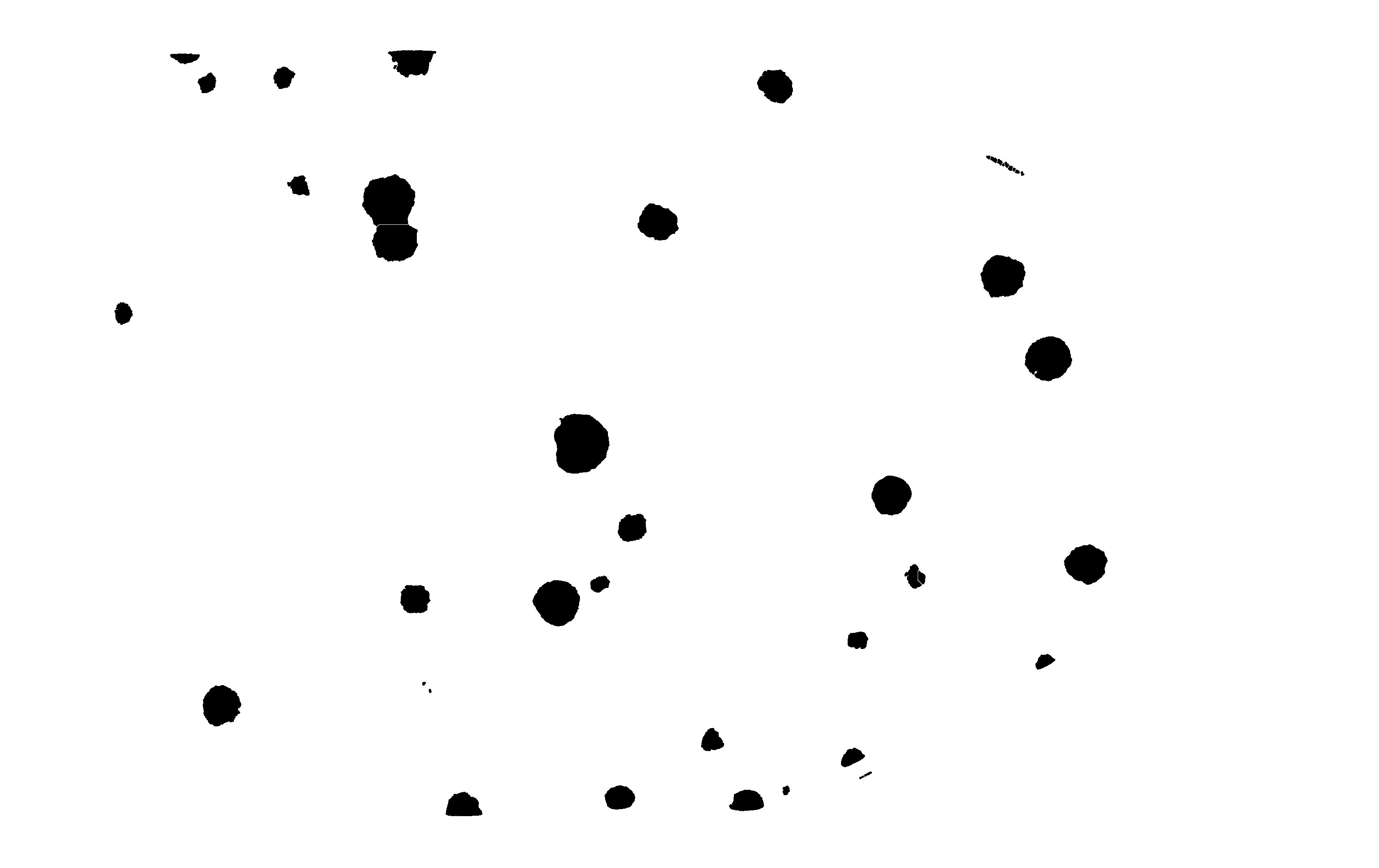

Supplement: S4 Datasets — It also contains a text file where results achieved by automated (CoCoNut, CAI, AutoCellSeg, and OpenCFU) and manual methods are summarized. (ZIP) [file pone.0205823.s005.zip › 180501 HeLa Flask/1 Second counting.jpg]

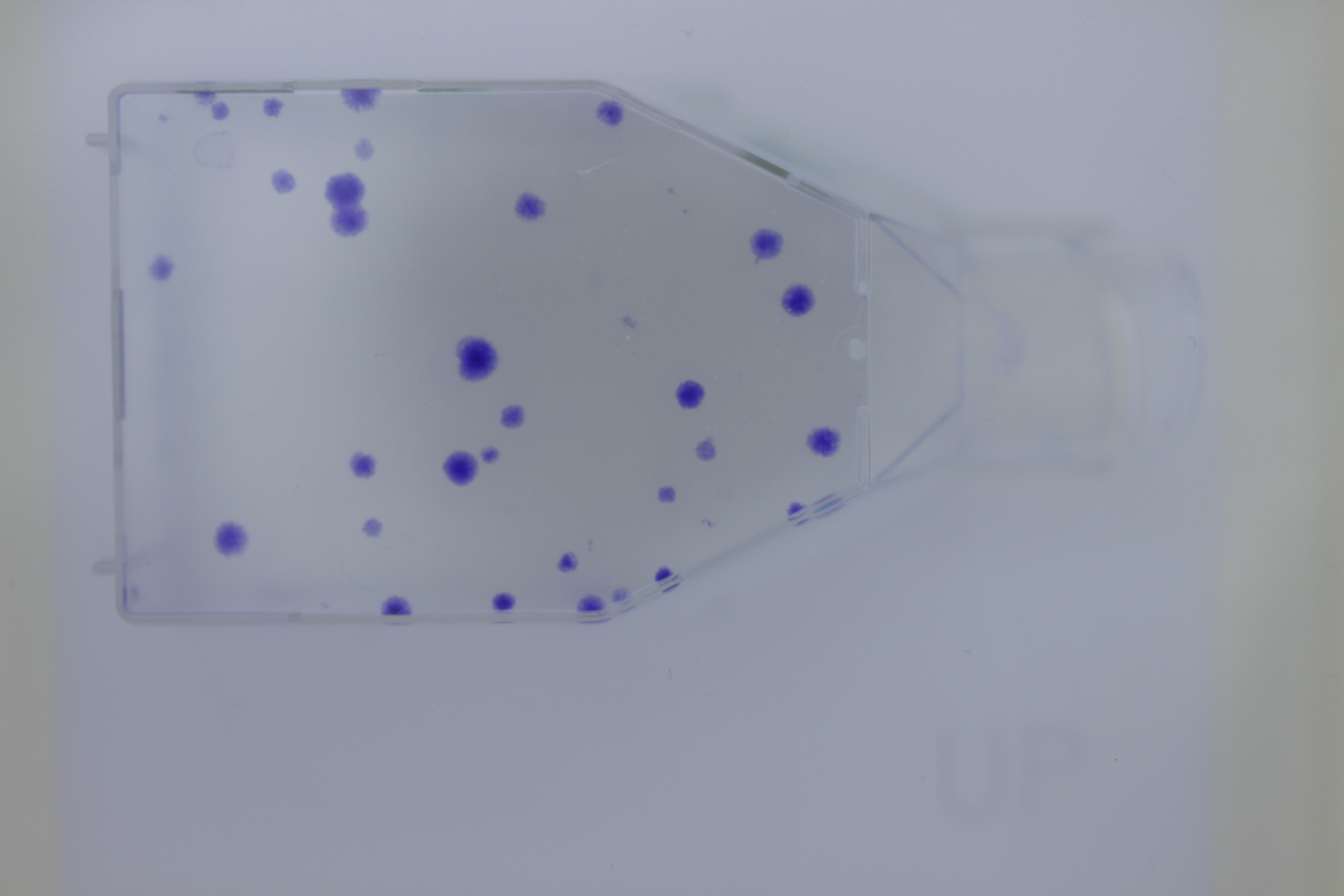

Supplement: S4 Datasets — It also contains a text file where results achieved by automated (CoCoNut, CAI, AutoCellSeg, and OpenCFU) and manual methods are summarized. (ZIP) [file pone.0205823.s005.zip › 180501 HeLa Flask/1.JPG]

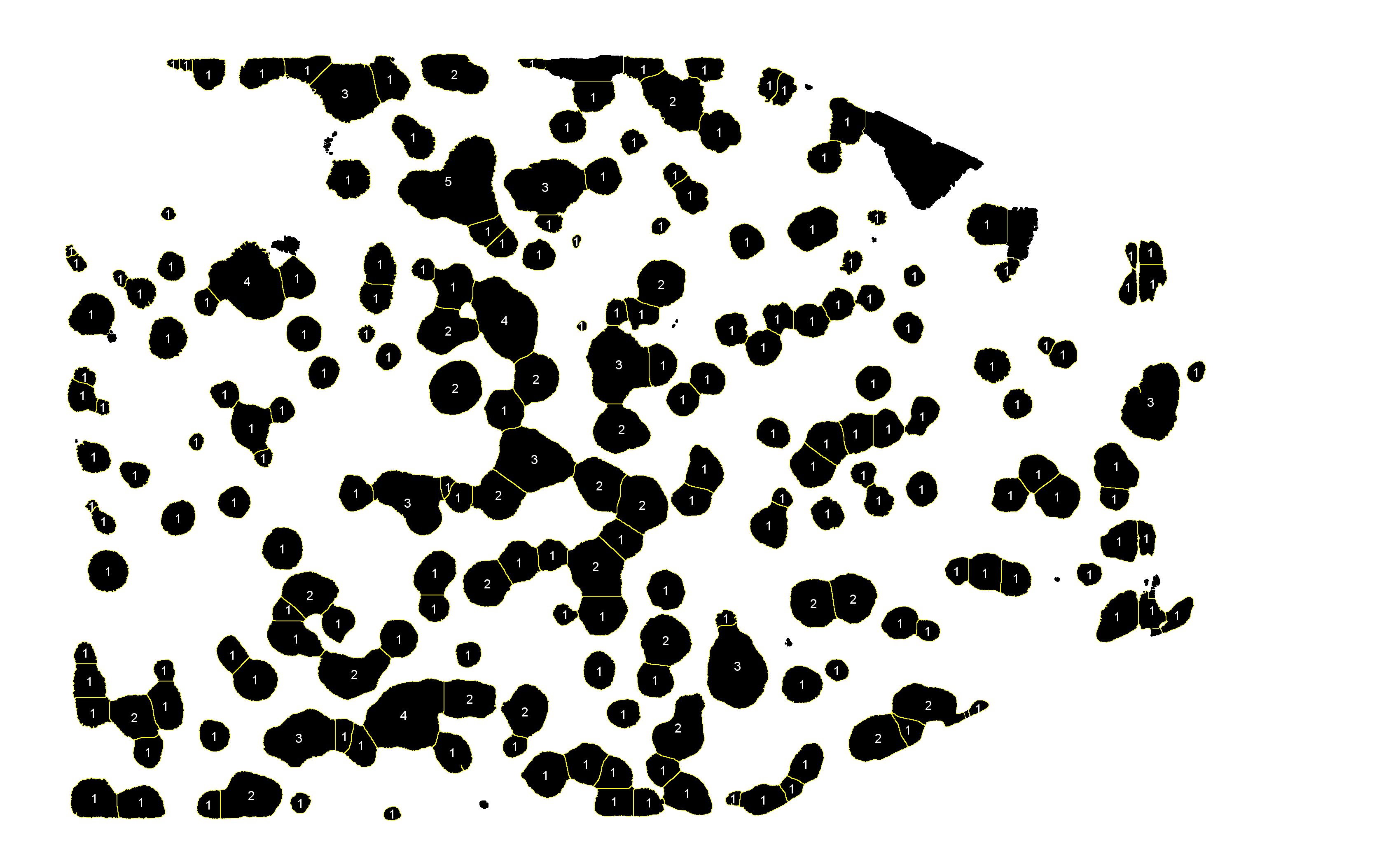

Supplement: S4 Datasets — It also contains a text file where results achieved by automated (CoCoNut, CAI, AutoCellSeg, and OpenCFU) and manual methods are summarized. (ZIP) [file pone.0205823.s005.zip › 180501 HeLa Flask/10 First counting.jpg]

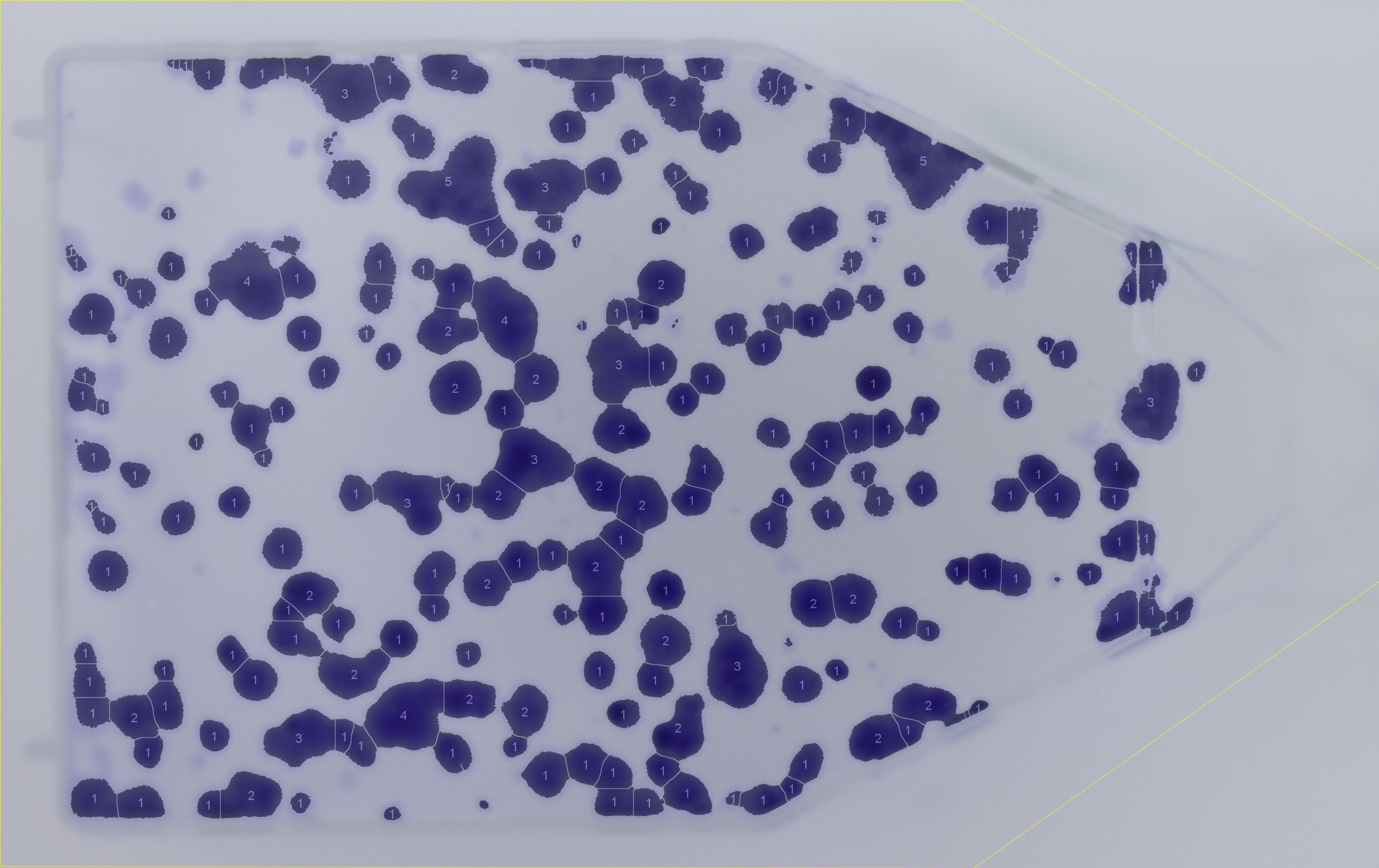

Supplement: S4 Datasets — It also contains a text file where results achieved by automated (CoCoNut, CAI, AutoCellSeg, and OpenCFU) and manual methods are summarized. (ZIP) [file pone.0205823.s005.zip › 180501 HeLa Flask/10 Results.jpg]
