# Supplementary figures and images for: Cell colony counter called CoCoNut (part 3 of 5)
Source: PLoS One. 2018 Nov 7;13(11):e0205823. doi: 10.1371/journal.pone.0205823 (PMC6221277; doi:10.1371/journal.pone.0205823)

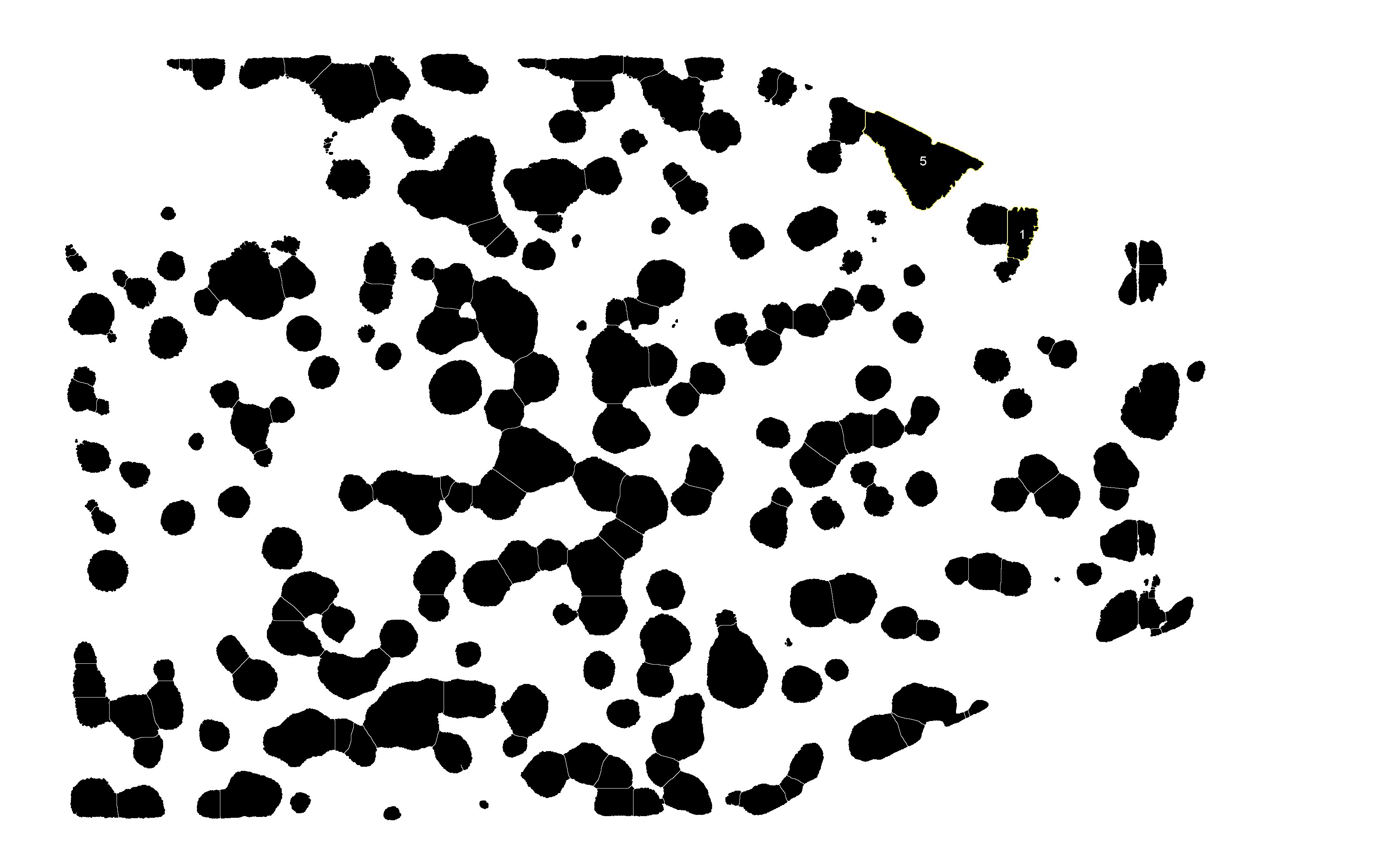

Supplement: S4 Datasets — It also contains a text file where results achieved by automated (CoCoNut, CAI, AutoCellSeg, and OpenCFU) and manual methods are summarized. (ZIP) [file pone.0205823.s005.zip › 180501 HeLa Flask/10 Second counting.jpg]

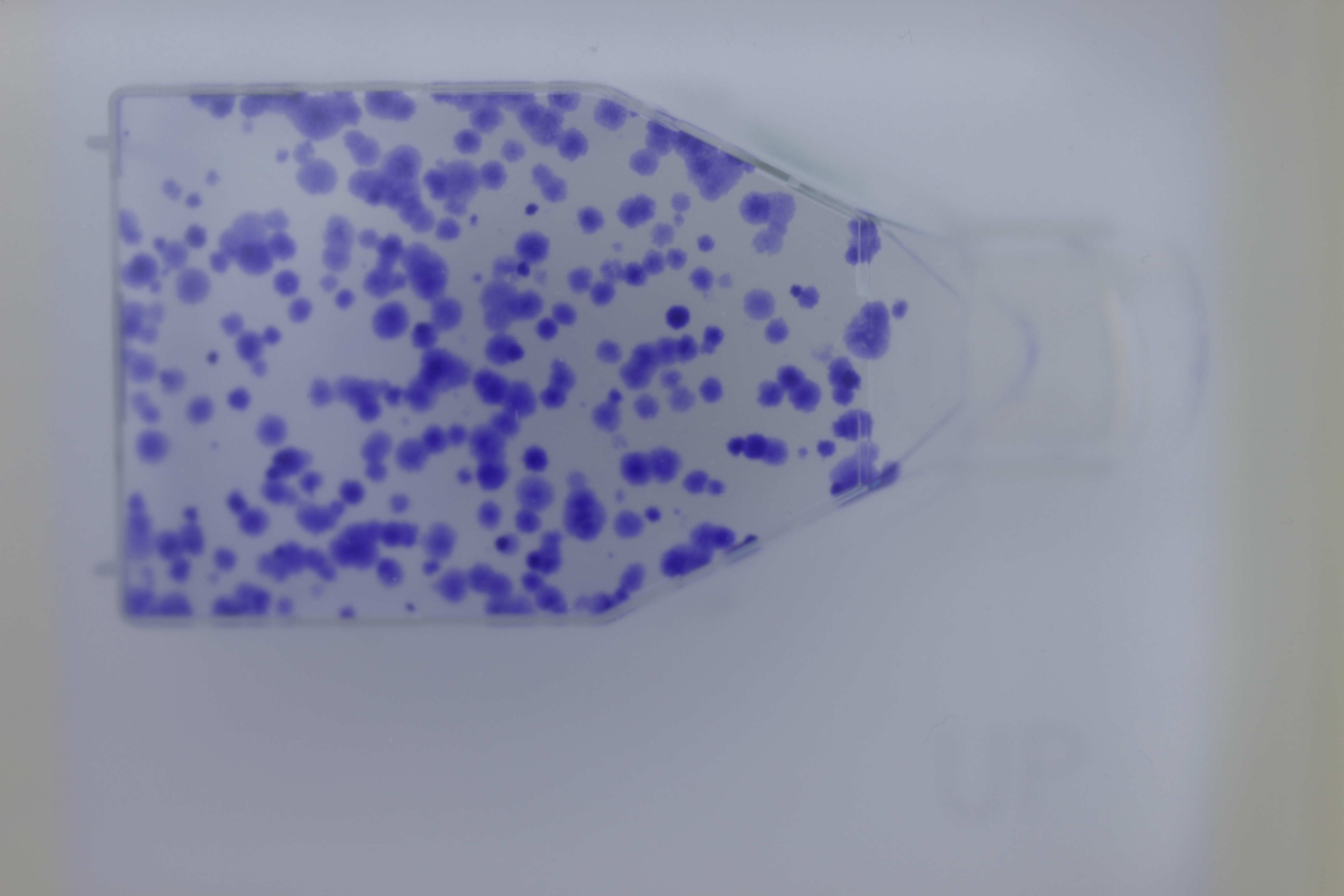

Supplement: S4 Datasets — It also contains a text file where results achieved by automated (CoCoNut, CAI, AutoCellSeg, and OpenCFU) and manual methods are summarized. (ZIP) [file pone.0205823.s005.zip › 180501 HeLa Flask/10.JPG]

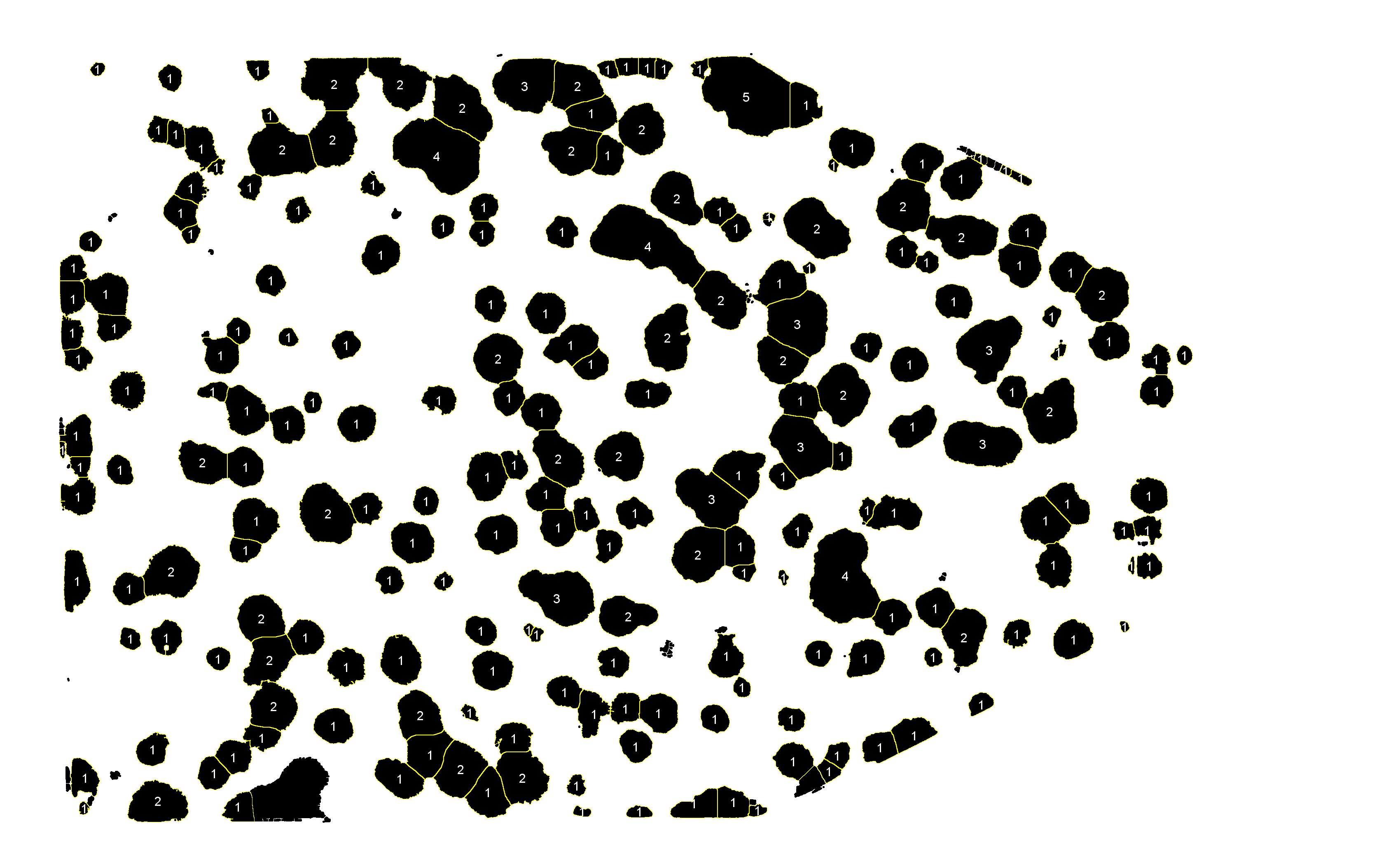

Supplement: S4 Datasets — It also contains a text file where results achieved by automated (CoCoNut, CAI, AutoCellSeg, and OpenCFU) and manual methods are summarized. (ZIP) [file pone.0205823.s005.zip › 180501 HeLa Flask/11 First counting.jpg]

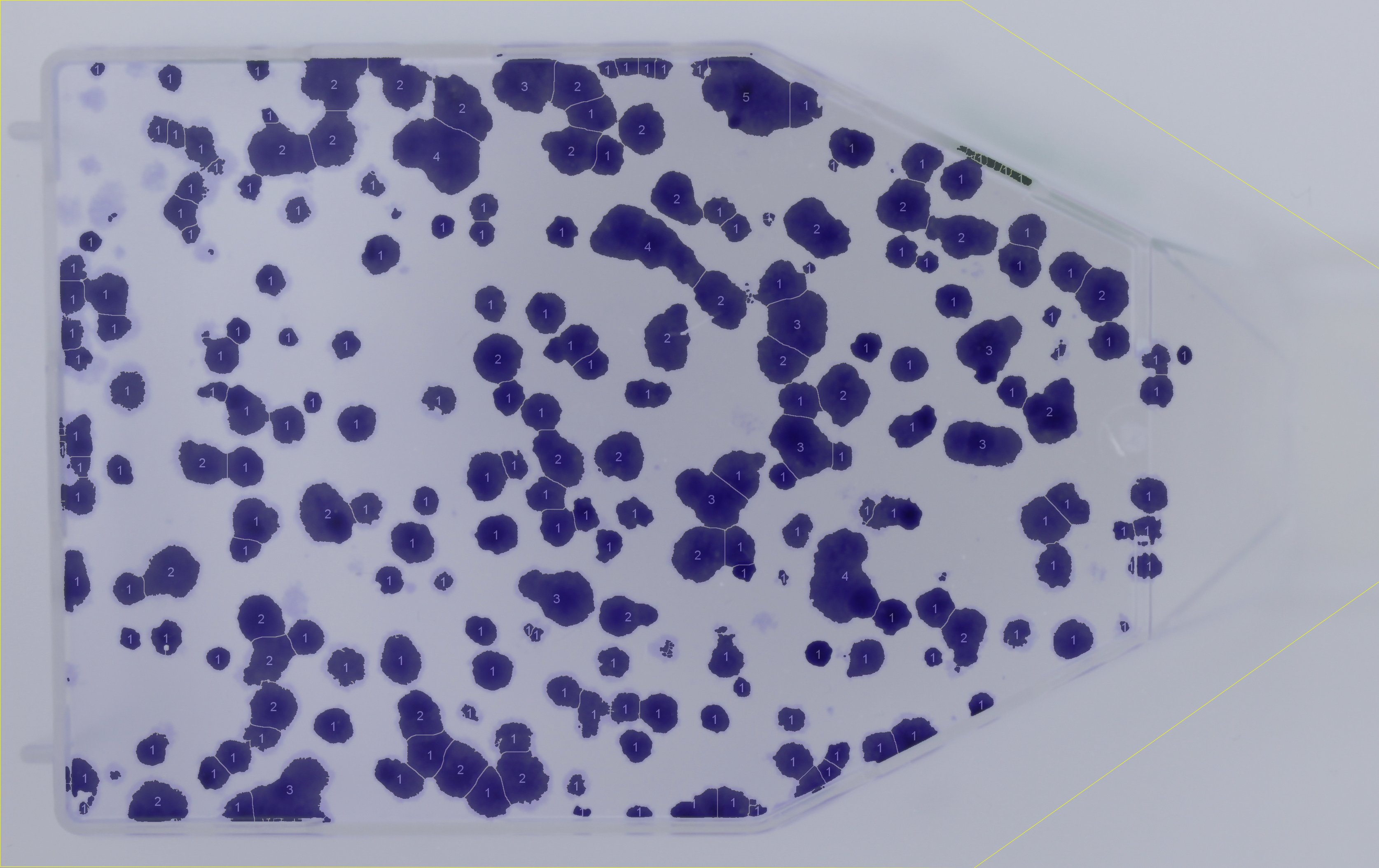

Supplement: S4 Datasets — It also contains a text file where results achieved by automated (CoCoNut, CAI, AutoCellSeg, and OpenCFU) and manual methods are summarized. (ZIP) [file pone.0205823.s005.zip › 180501 HeLa Flask/11 Results.jpg]

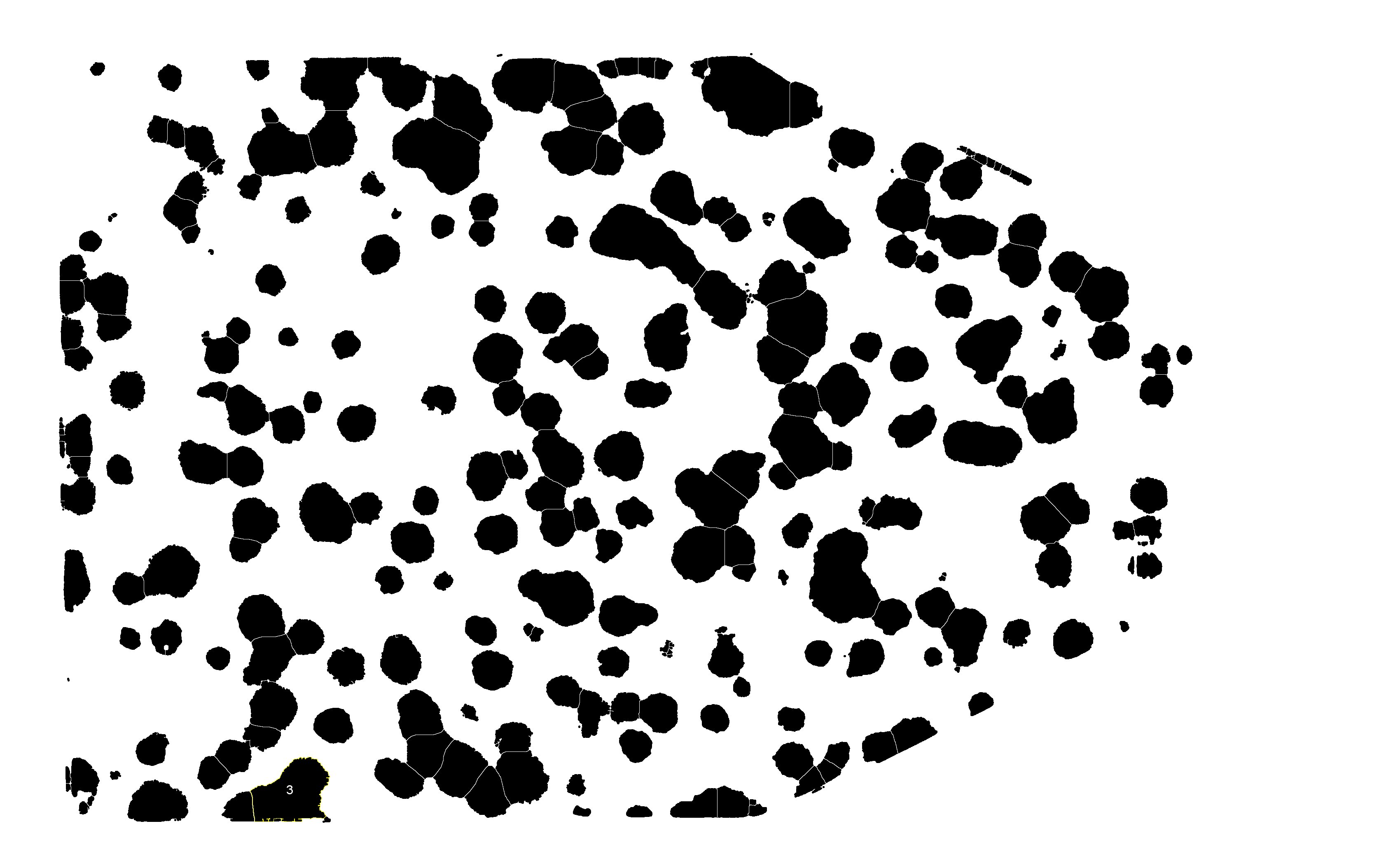

Supplement: S4 Datasets — It also contains a text file where results achieved by automated (CoCoNut, CAI, AutoCellSeg, and OpenCFU) and manual methods are summarized. (ZIP) [file pone.0205823.s005.zip › 180501 HeLa Flask/11 Second counting.jpg]

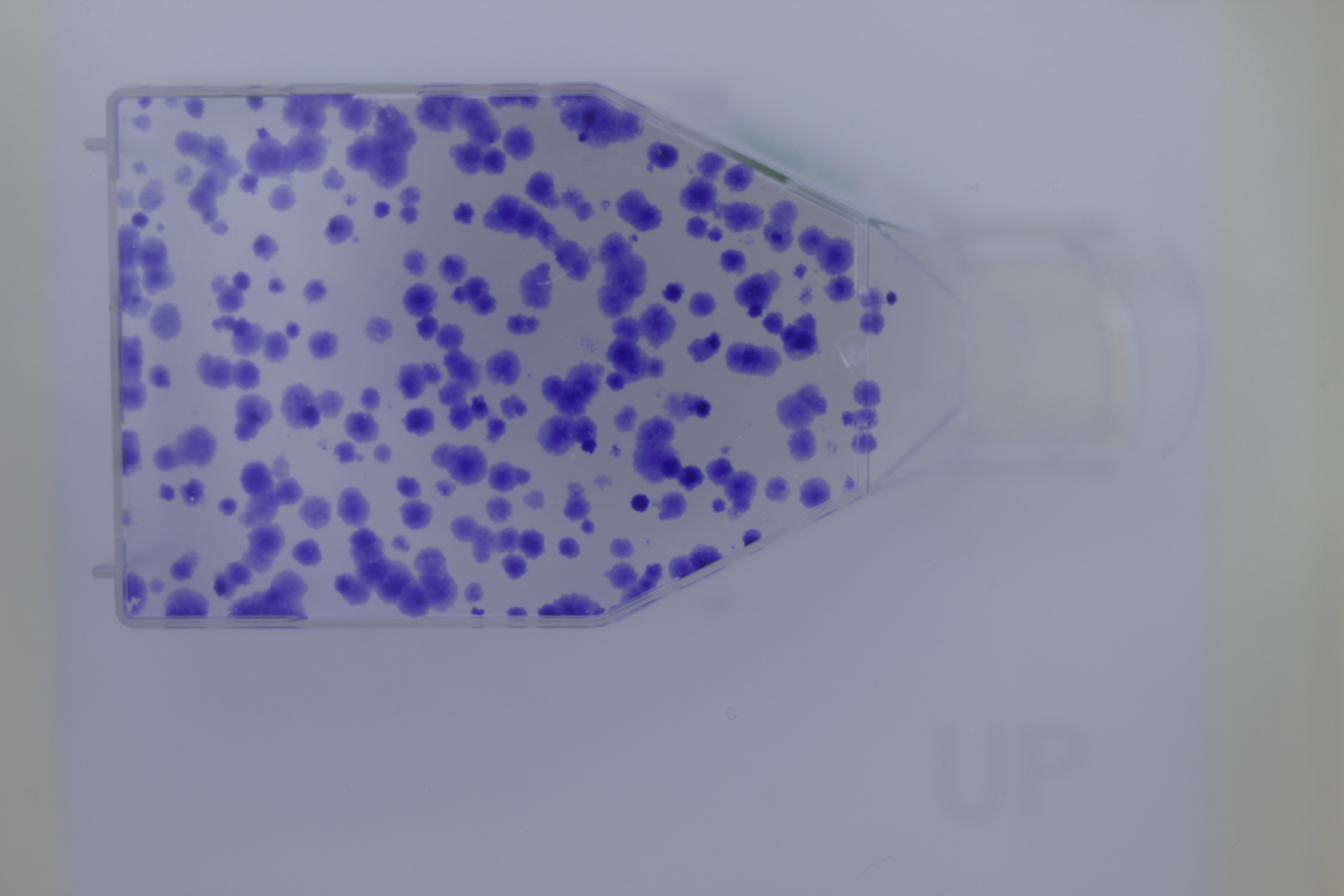

Supplement: S4 Datasets — It also contains a text file where results achieved by automated (CoCoNut, CAI, AutoCellSeg, and OpenCFU) and manual methods are summarized. (ZIP) [file pone.0205823.s005.zip › 180501 HeLa Flask/11.JPG]

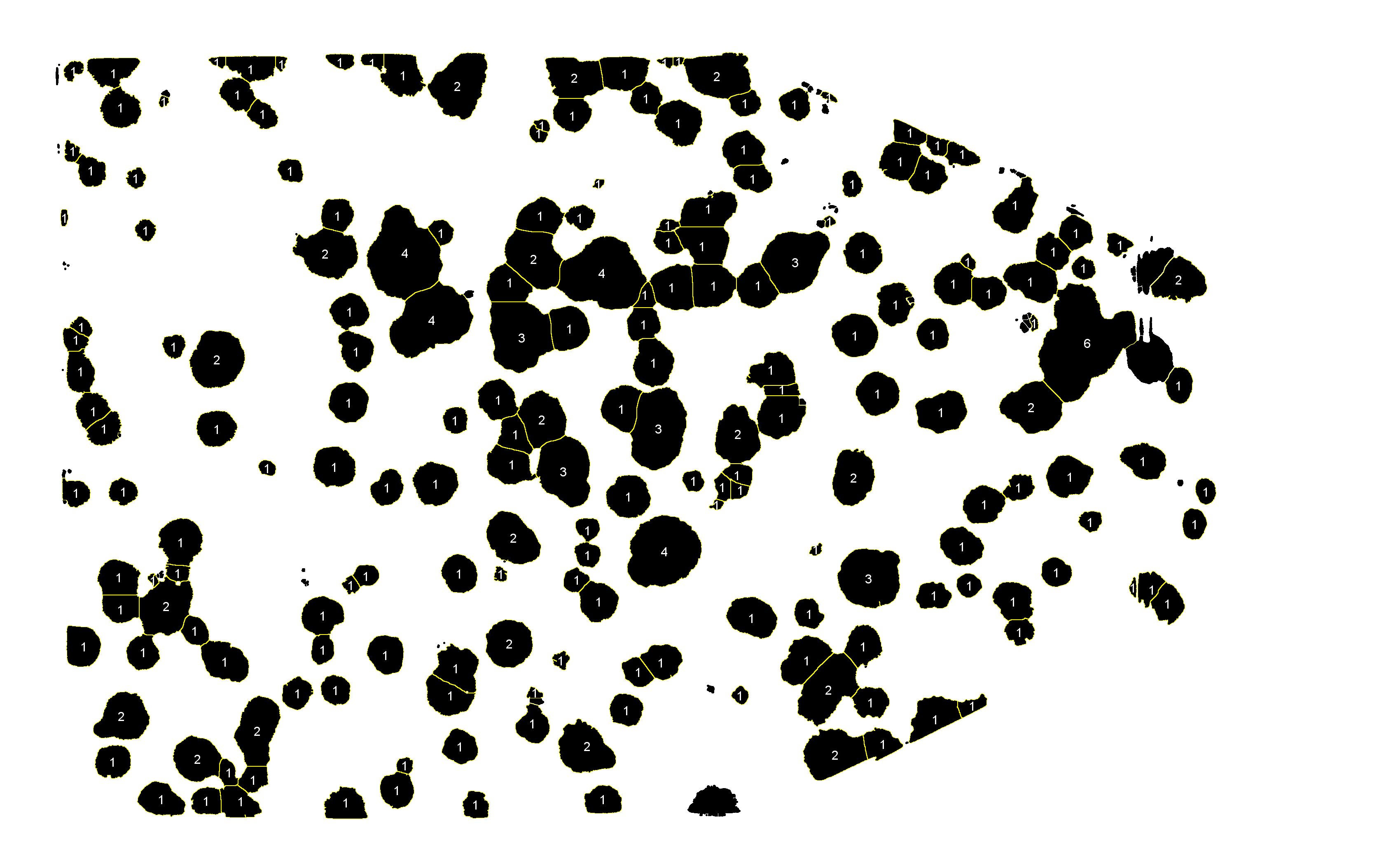

Supplement: S4 Datasets — It also contains a text file where results achieved by automated (CoCoNut, CAI, AutoCellSeg, and OpenCFU) and manual methods are summarized. (ZIP) [file pone.0205823.s005.zip › 180501 HeLa Flask/12 First counting.jpg]

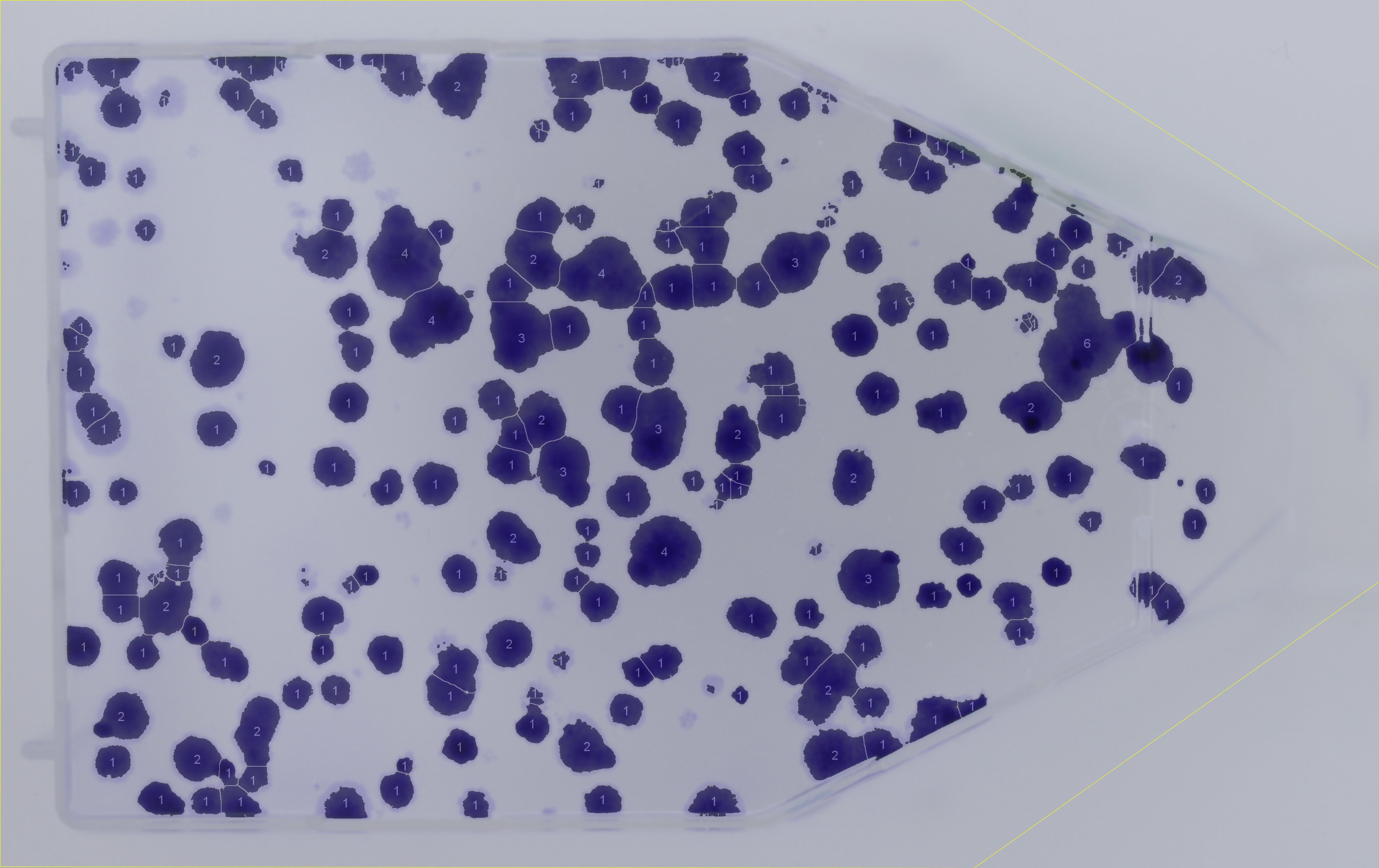

Supplement: S4 Datasets — It also contains a text file where results achieved by automated (CoCoNut, CAI, AutoCellSeg, and OpenCFU) and manual methods are summarized. (ZIP) [file pone.0205823.s005.zip › 180501 HeLa Flask/12 Results.jpg]

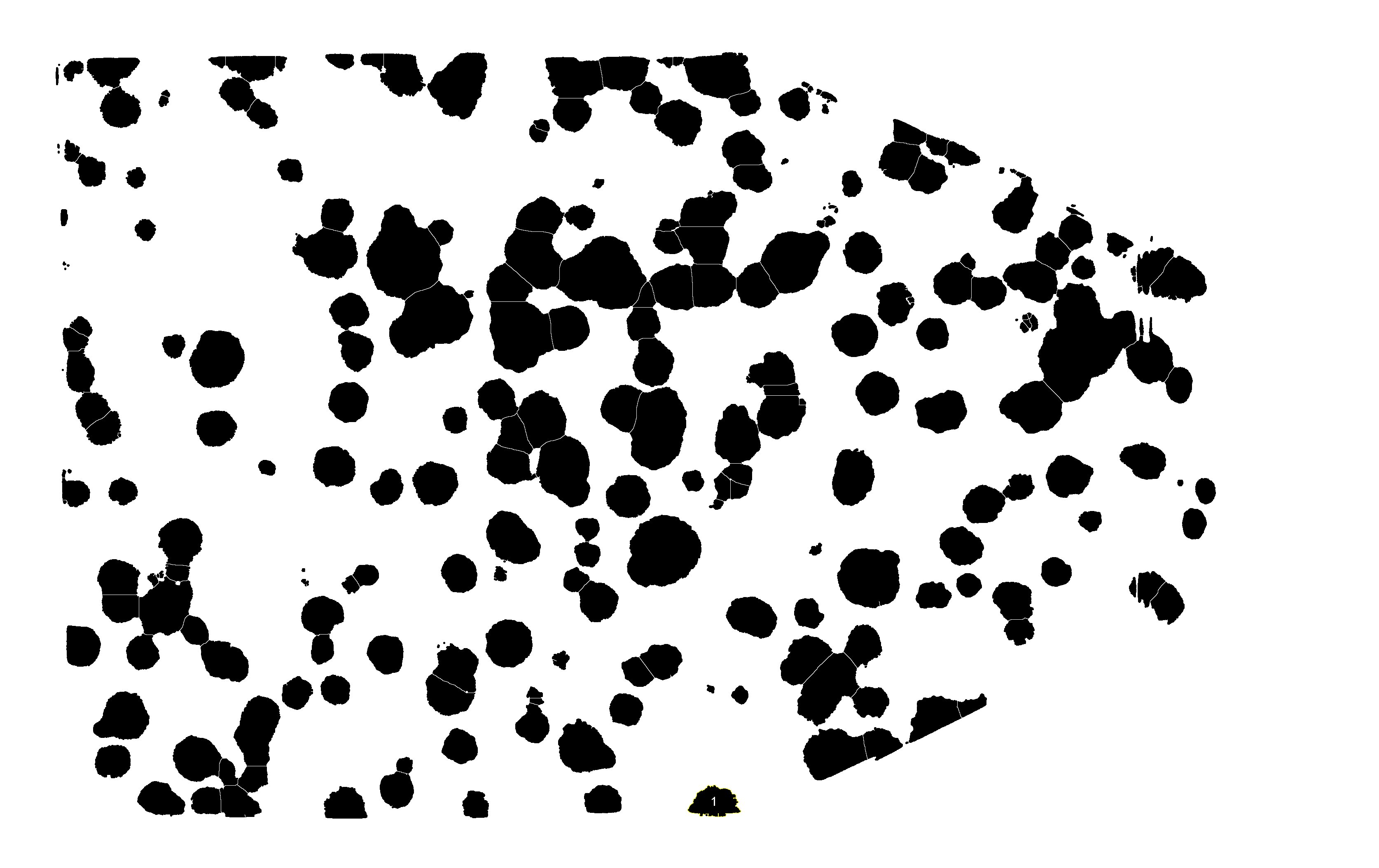

Supplement: S4 Datasets — It also contains a text file where results achieved by automated (CoCoNut, CAI, AutoCellSeg, and OpenCFU) and manual methods are summarized. (ZIP) [file pone.0205823.s005.zip › 180501 HeLa Flask/12 Second counting.jpg]

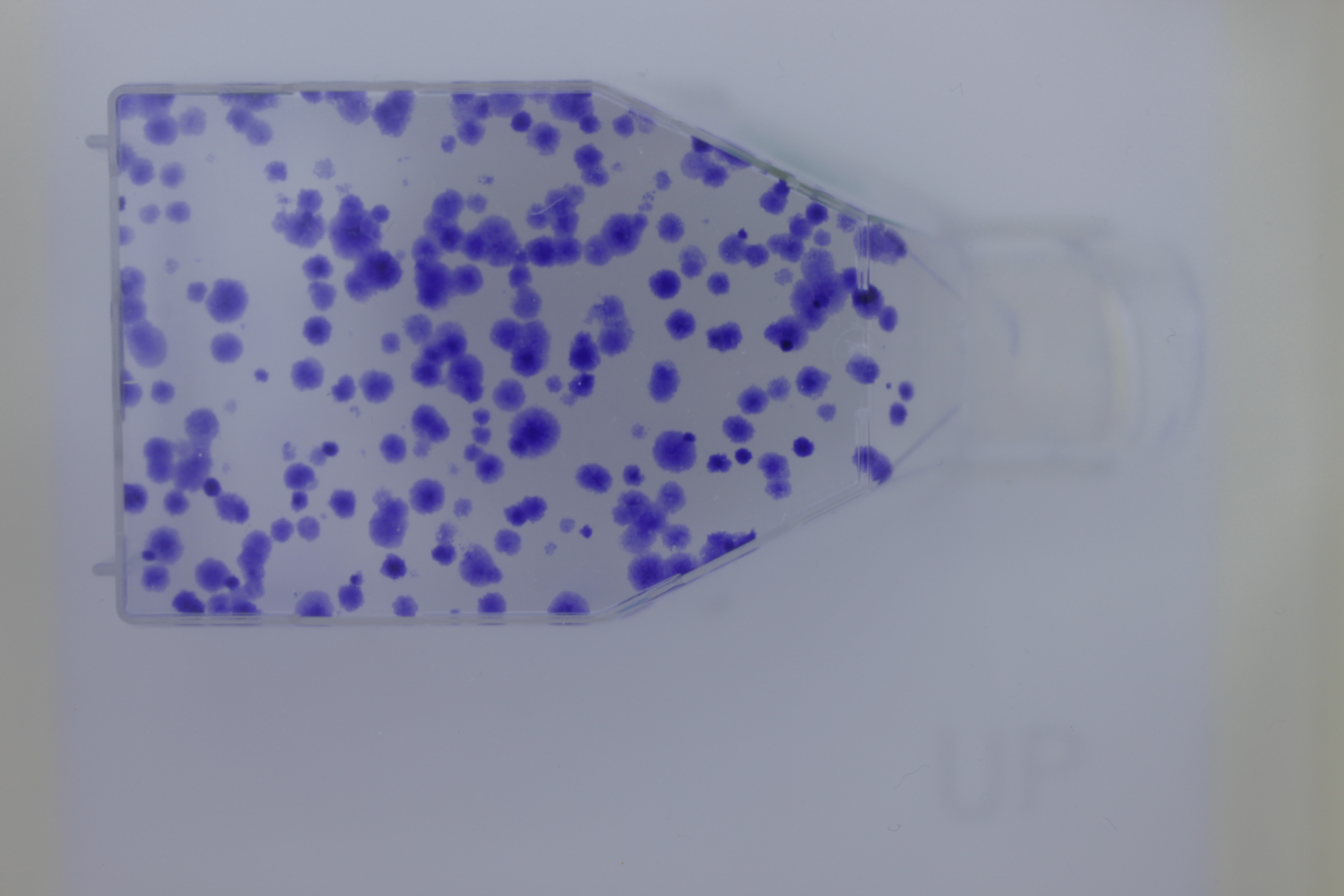

Supplement: S4 Datasets — It also contains a text file where results achieved by automated (CoCoNut, CAI, AutoCellSeg, and OpenCFU) and manual methods are summarized. (ZIP) [file pone.0205823.s005.zip › 180501 HeLa Flask/12.JPG]

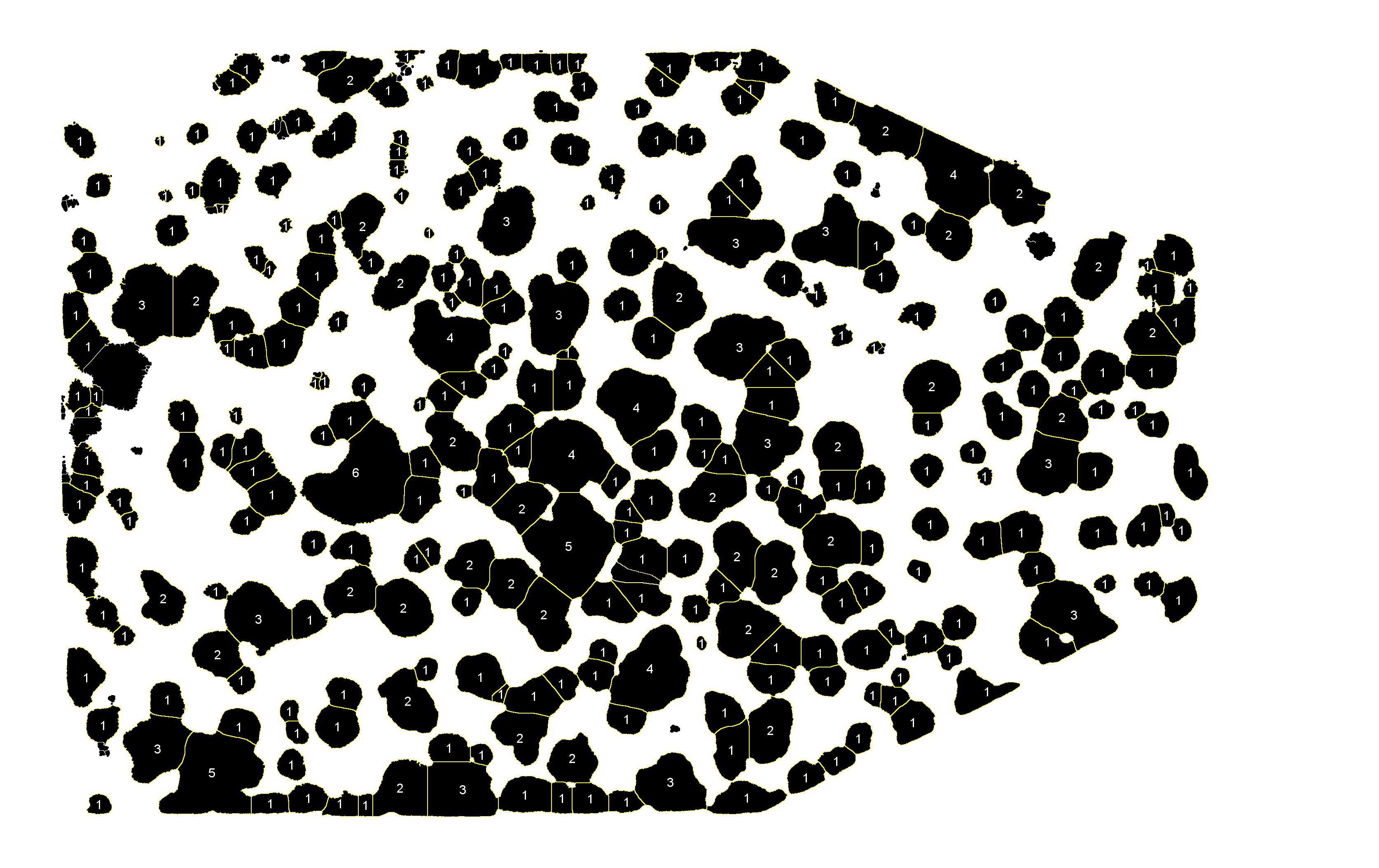

Supplement: S4 Datasets — It also contains a text file where results achieved by automated (CoCoNut, CAI, AutoCellSeg, and OpenCFU) and manual methods are summarized. (ZIP) [file pone.0205823.s005.zip › 180501 HeLa Flask/13 First counting.jpg]

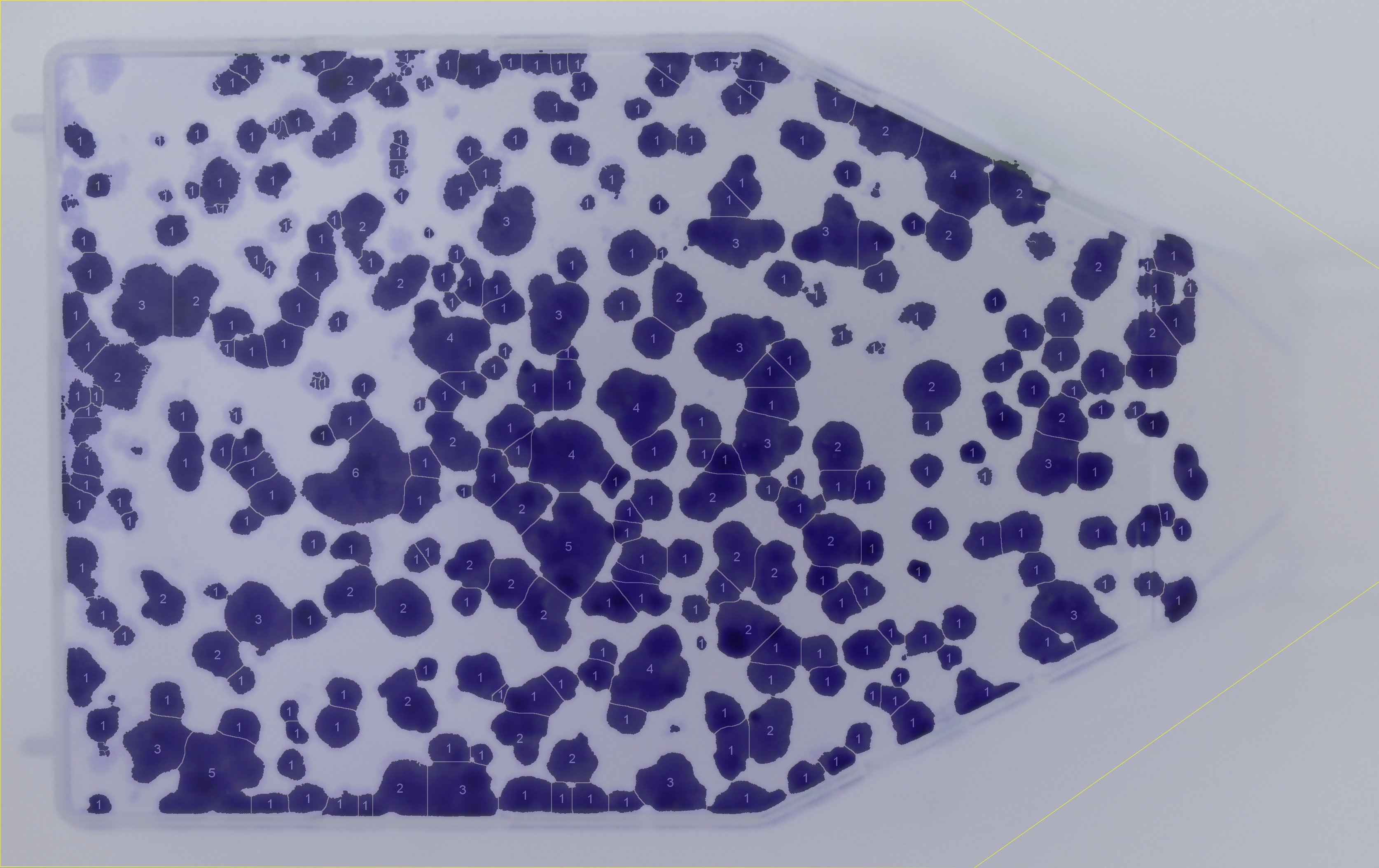

Supplement: S4 Datasets — It also contains a text file where results achieved by automated (CoCoNut, CAI, AutoCellSeg, and OpenCFU) and manual methods are summarized. (ZIP) [file pone.0205823.s005.zip › 180501 HeLa Flask/13 Results.jpg]

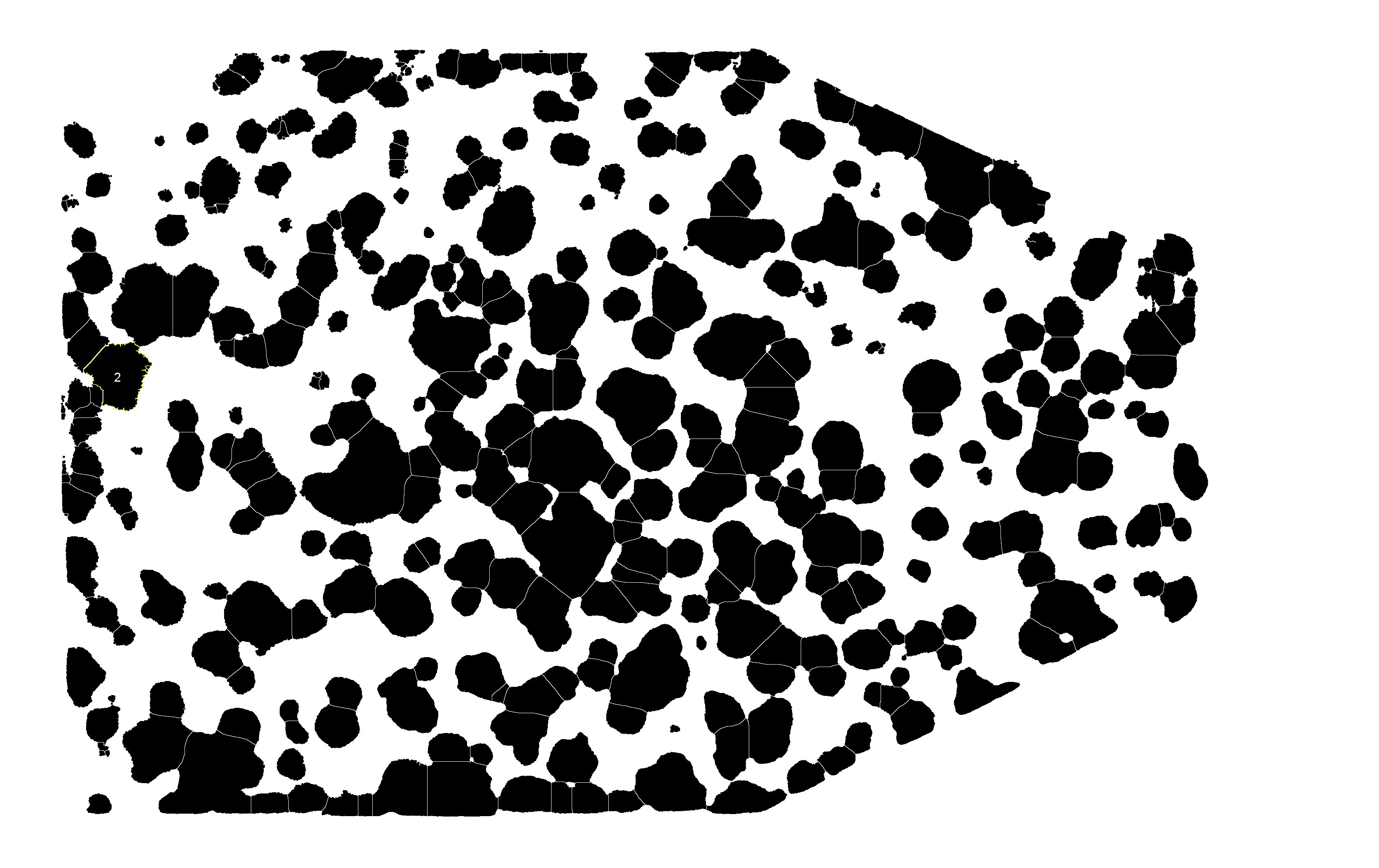

Supplement: S4 Datasets — It also contains a text file where results achieved by automated (CoCoNut, CAI, AutoCellSeg, and OpenCFU) and manual methods are summarized. (ZIP) [file pone.0205823.s005.zip › 180501 HeLa Flask/13 Second counting.jpg]

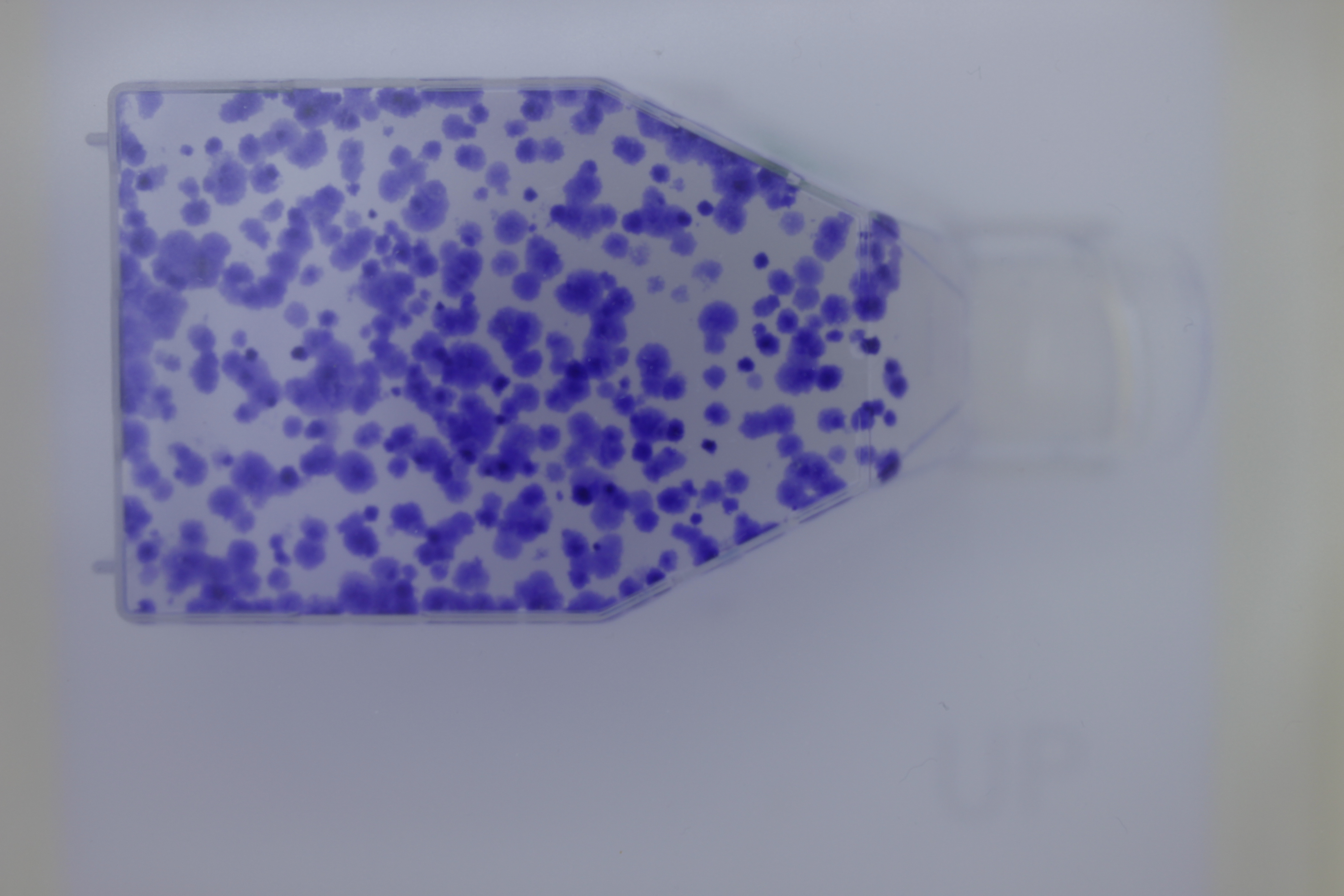

Supplement: S4 Datasets — It also contains a text file where results achieved by automated (CoCoNut, CAI, AutoCellSeg, and OpenCFU) and manual methods are summarized. (ZIP) [file pone.0205823.s005.zip › 180501 HeLa Flask/13.JPG]

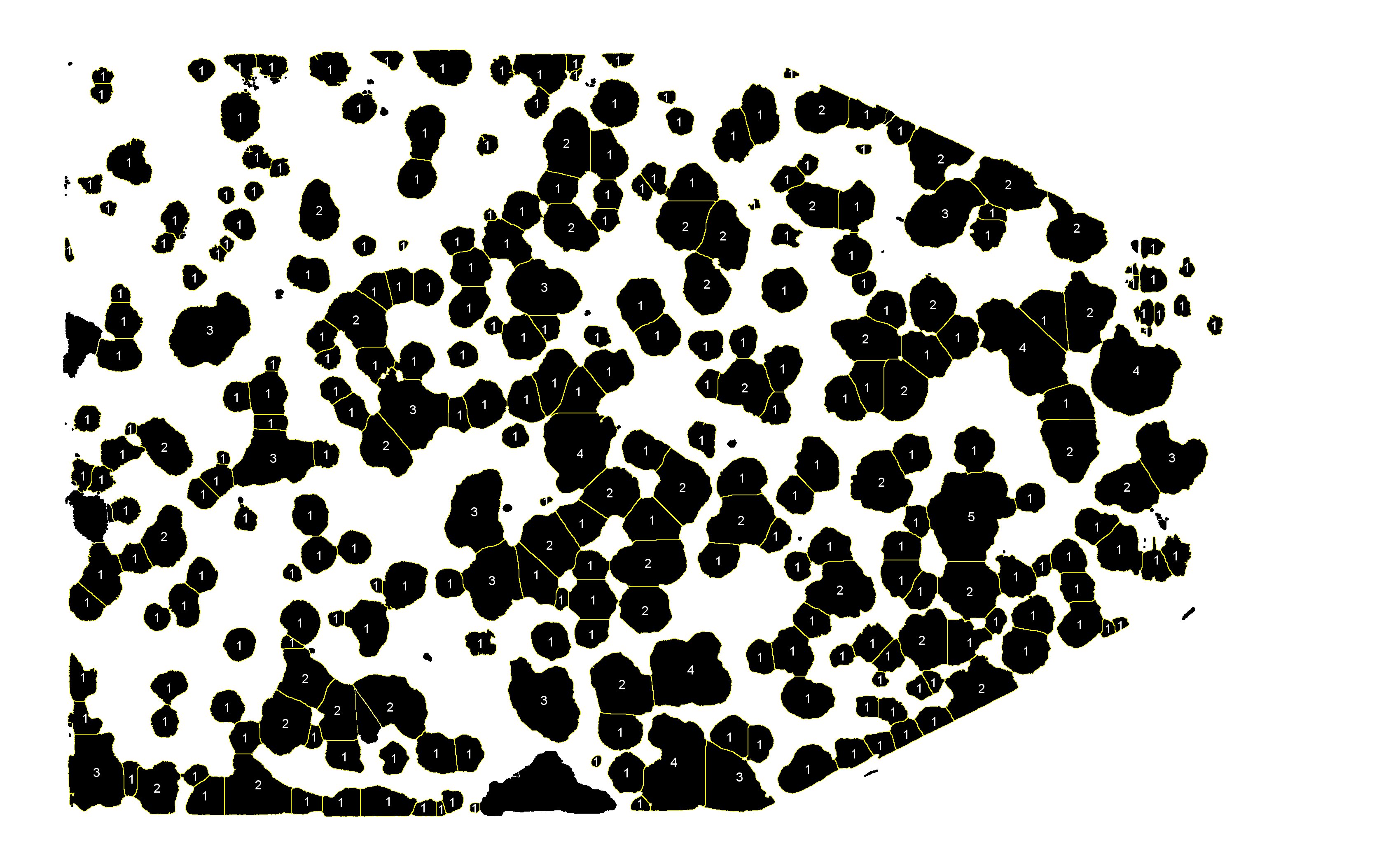

Supplement: S4 Datasets — It also contains a text file where results achieved by automated (CoCoNut, CAI, AutoCellSeg, and OpenCFU) and manual methods are summarized. (ZIP) [file pone.0205823.s005.zip › 180501 HeLa Flask/14 First counting.jpg]

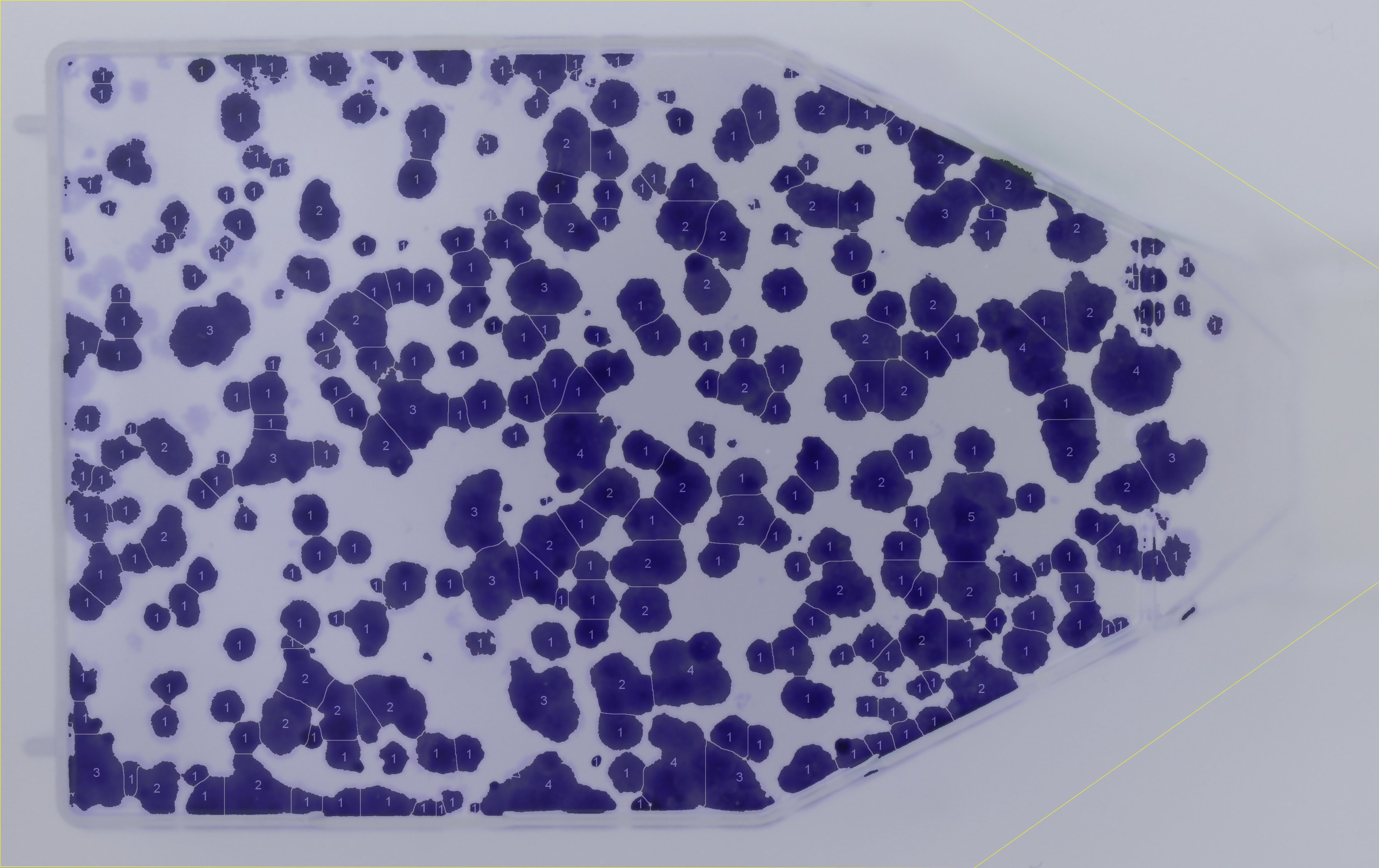

Supplement: S4 Datasets — It also contains a text file where results achieved by automated (CoCoNut, CAI, AutoCellSeg, and OpenCFU) and manual methods are summarized. (ZIP) [file pone.0205823.s005.zip › 180501 HeLa Flask/14 Results.jpg]

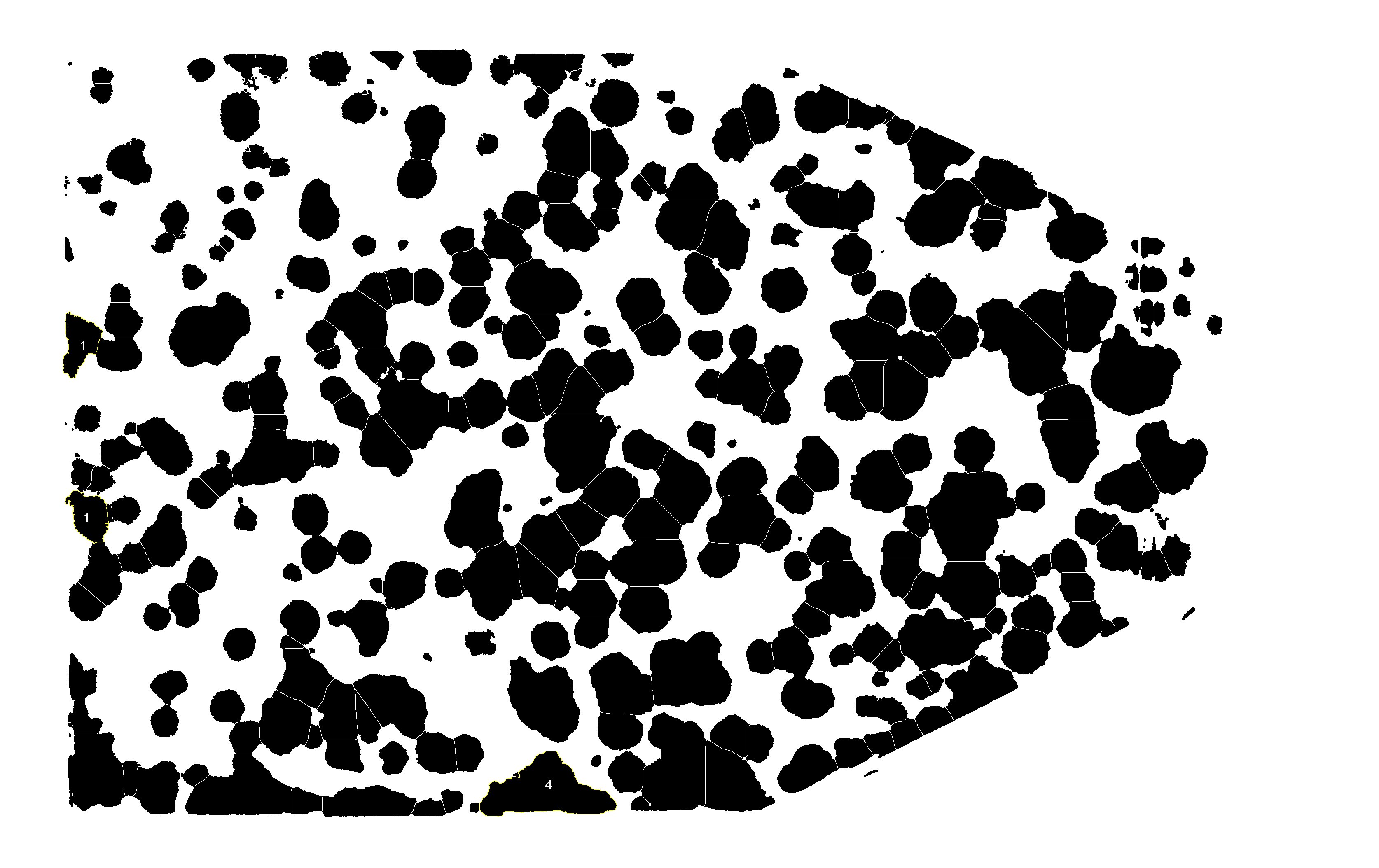

Supplement: S4 Datasets — It also contains a text file where results achieved by automated (CoCoNut, CAI, AutoCellSeg, and OpenCFU) and manual methods are summarized. (ZIP) [file pone.0205823.s005.zip › 180501 HeLa Flask/14 Second counting.jpg]

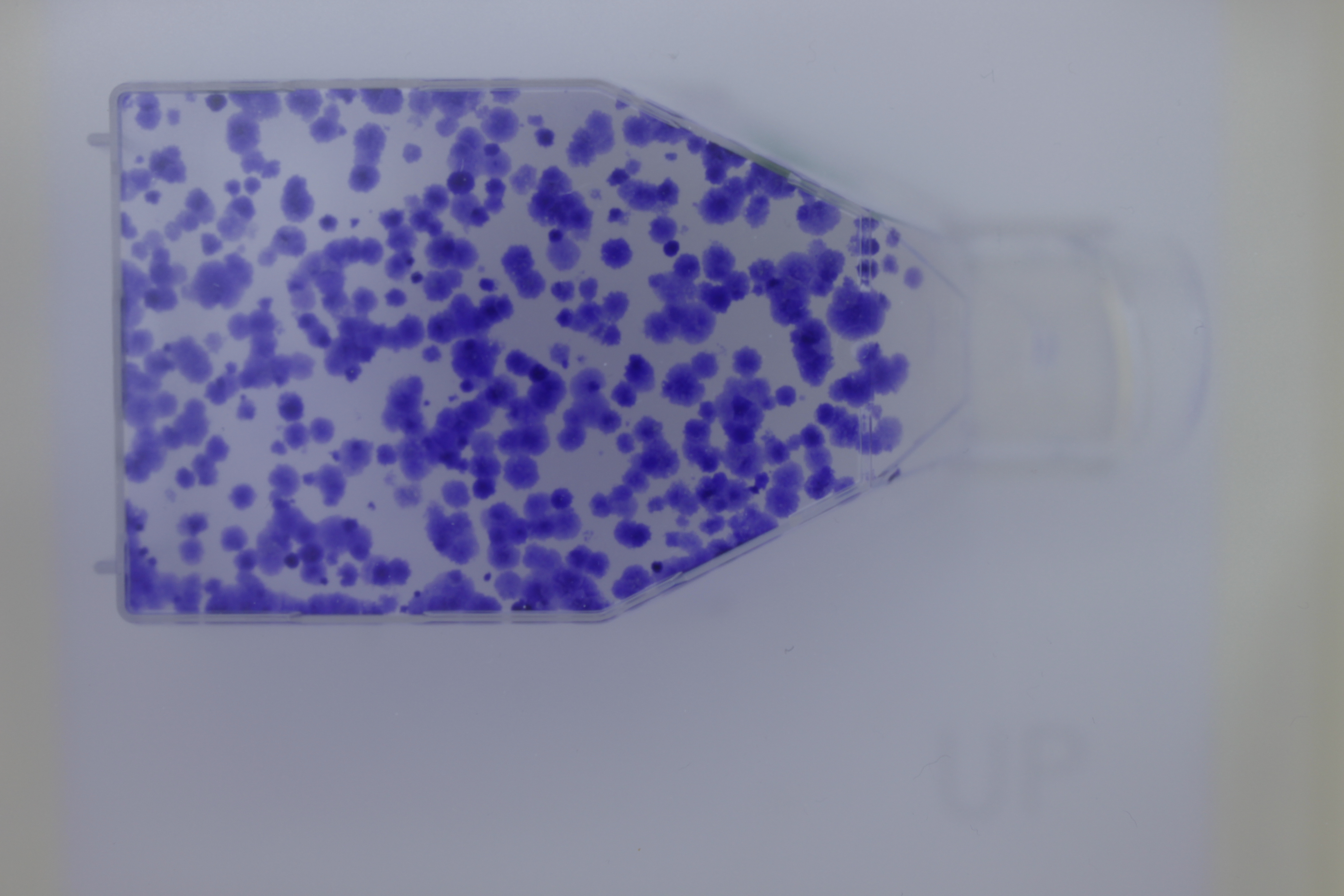

Supplement: S4 Datasets — It also contains a text file where results achieved by automated (CoCoNut, CAI, AutoCellSeg, and OpenCFU) and manual methods are summarized. (ZIP) [file pone.0205823.s005.zip › 180501 HeLa Flask/14.JPG]

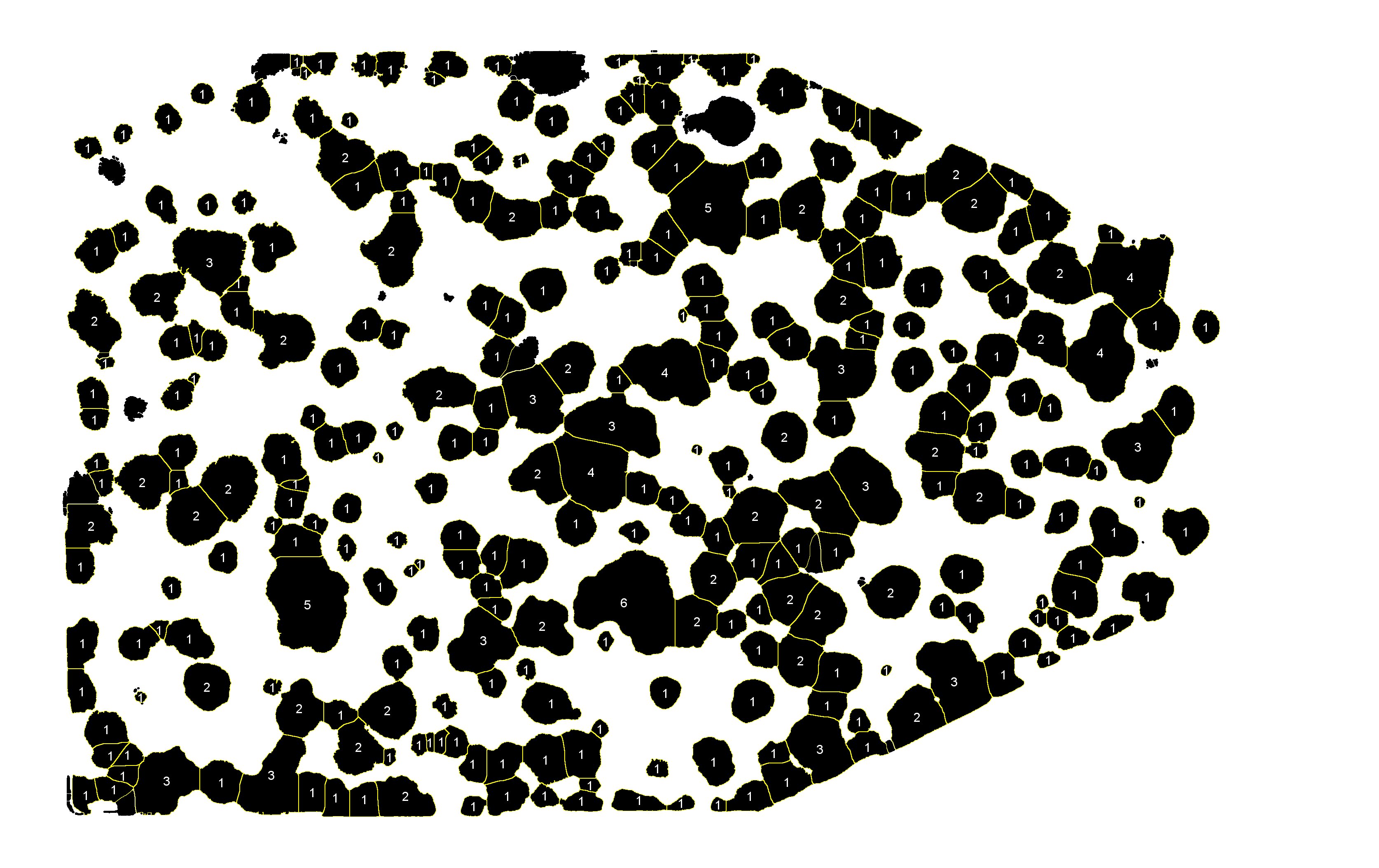

Supplement: S4 Datasets — It also contains a text file where results achieved by automated (CoCoNut, CAI, AutoCellSeg, and OpenCFU) and manual methods are summarized. (ZIP) [file pone.0205823.s005.zip › 180501 HeLa Flask/15 First counting.jpg]

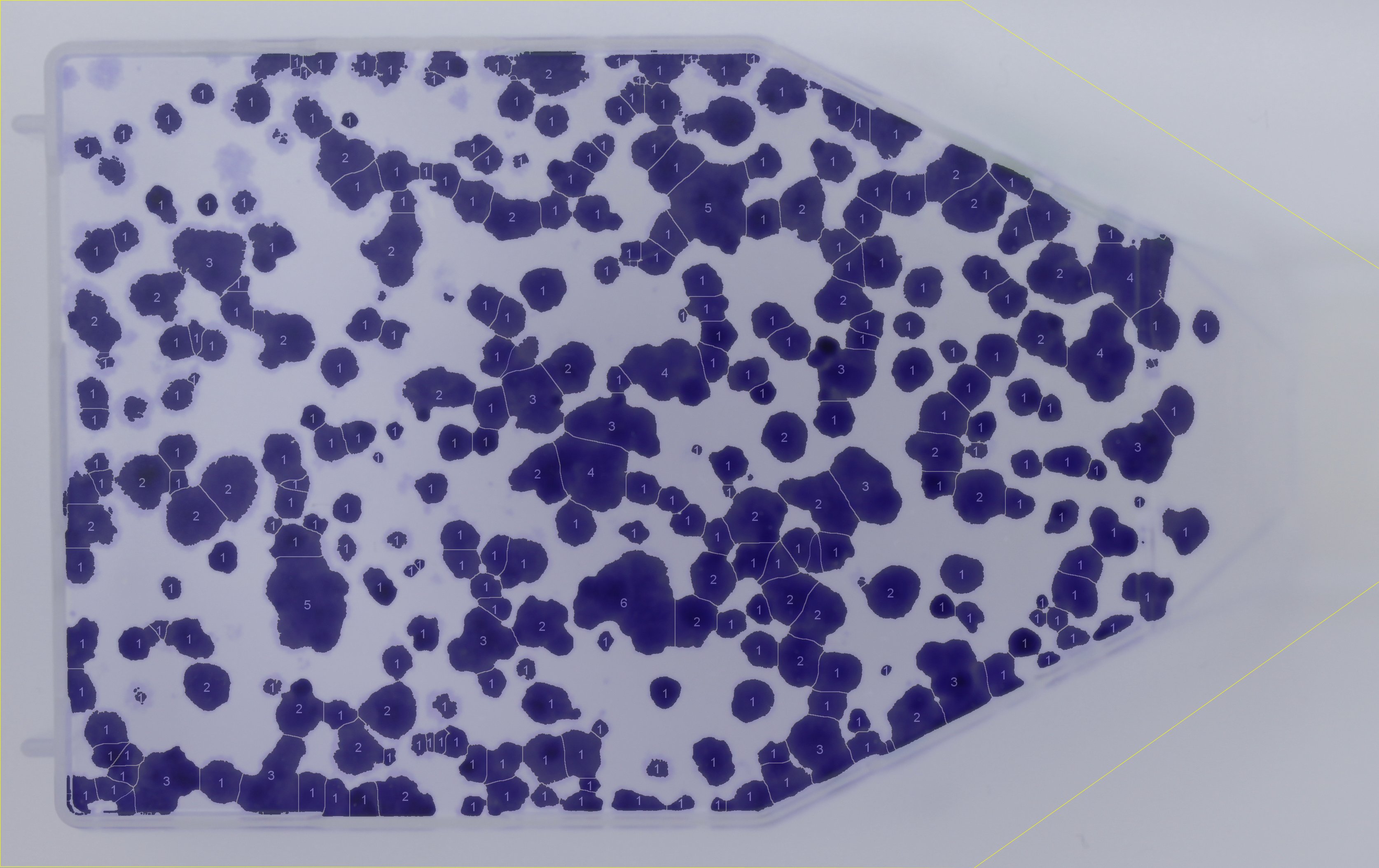

Supplement: S4 Datasets — It also contains a text file where results achieved by automated (CoCoNut, CAI, AutoCellSeg, and OpenCFU) and manual methods are summarized. (ZIP) [file pone.0205823.s005.zip › 180501 HeLa Flask/15 Results.jpg]

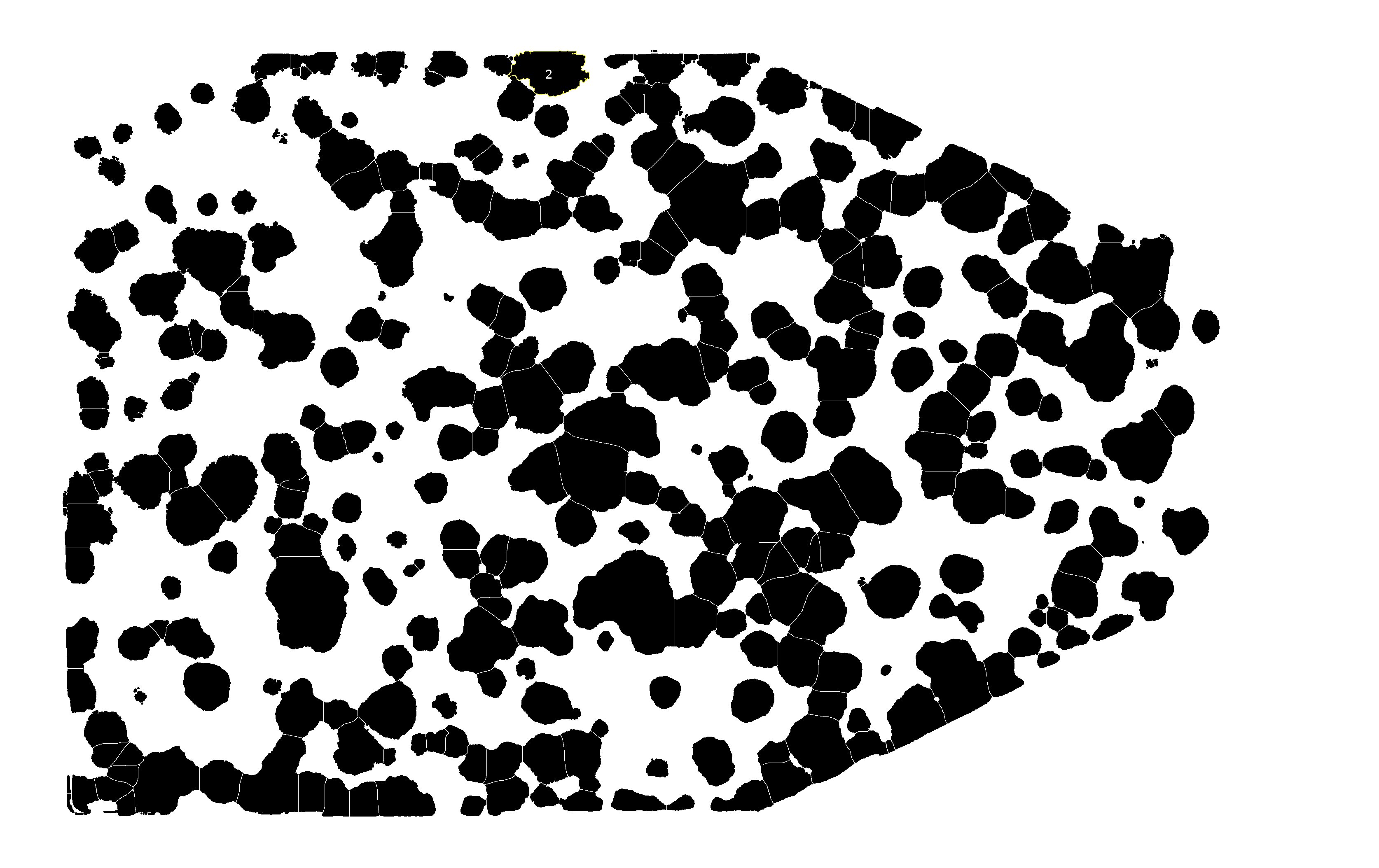

Supplement: S4 Datasets — It also contains a text file where results achieved by automated (CoCoNut, CAI, AutoCellSeg, and OpenCFU) and manual methods are summarized. (ZIP) [file pone.0205823.s005.zip › 180501 HeLa Flask/15 Second counting.jpg]

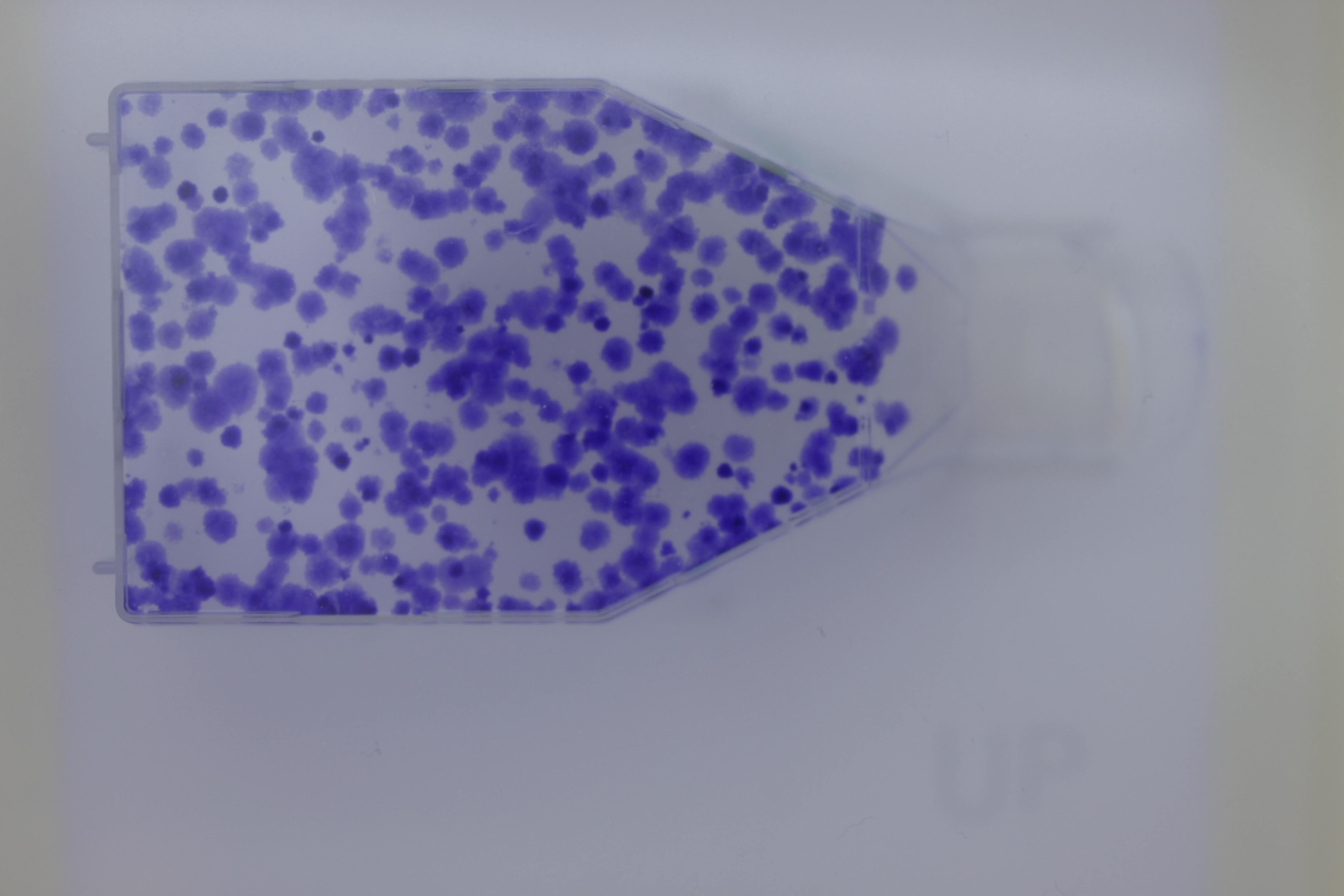

Supplement: S4 Datasets — It also contains a text file where results achieved by automated (CoCoNut, CAI, AutoCellSeg, and OpenCFU) and manual methods are summarized. (ZIP) [file pone.0205823.s005.zip › 180501 HeLa Flask/15.JPG]

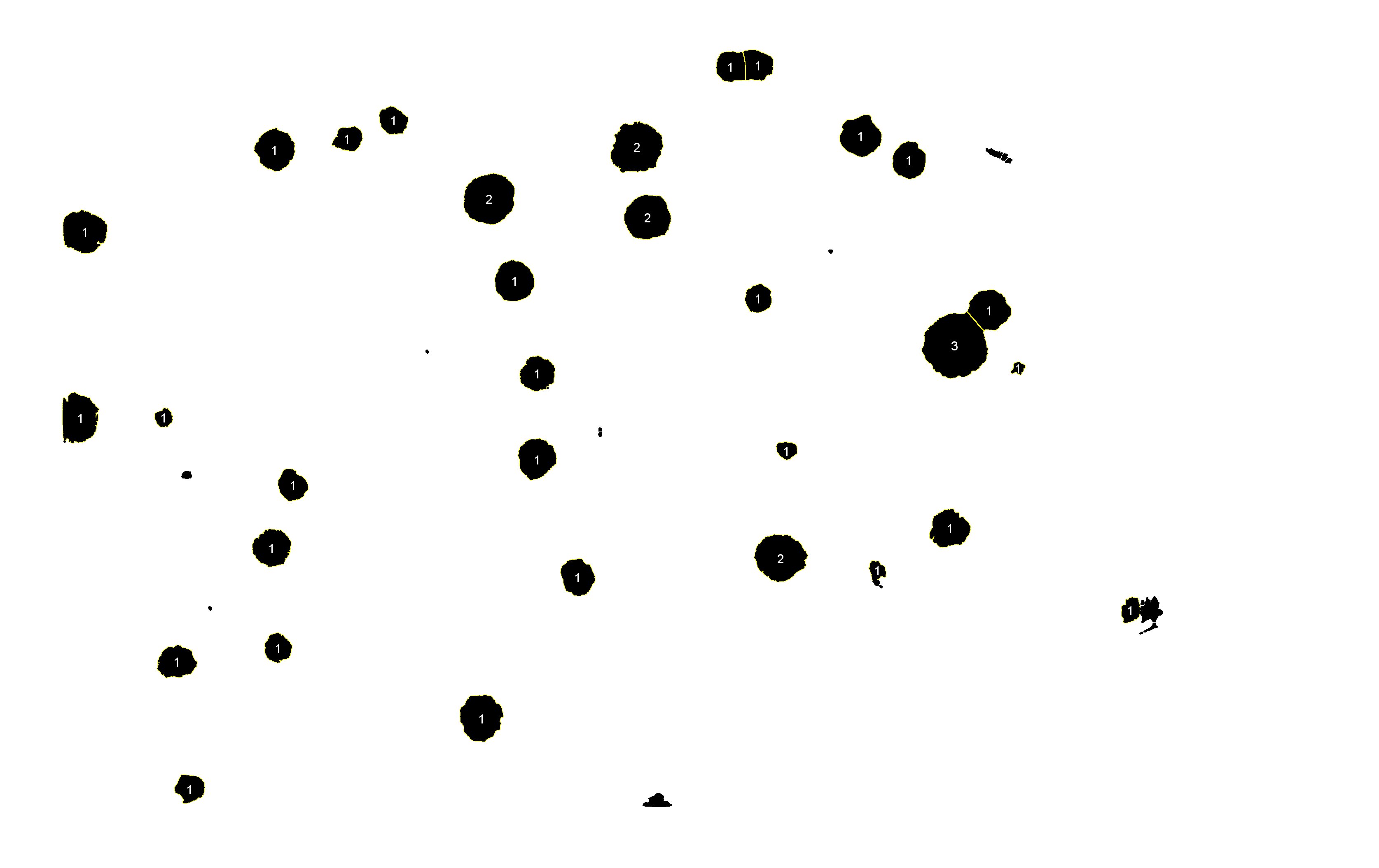

Supplement: S4 Datasets — It also contains a text file where results achieved by automated (CoCoNut, CAI, AutoCellSeg, and OpenCFU) and manual methods are summarized. (ZIP) [file pone.0205823.s005.zip › 180501 HeLa Flask/2 First counting.jpg]

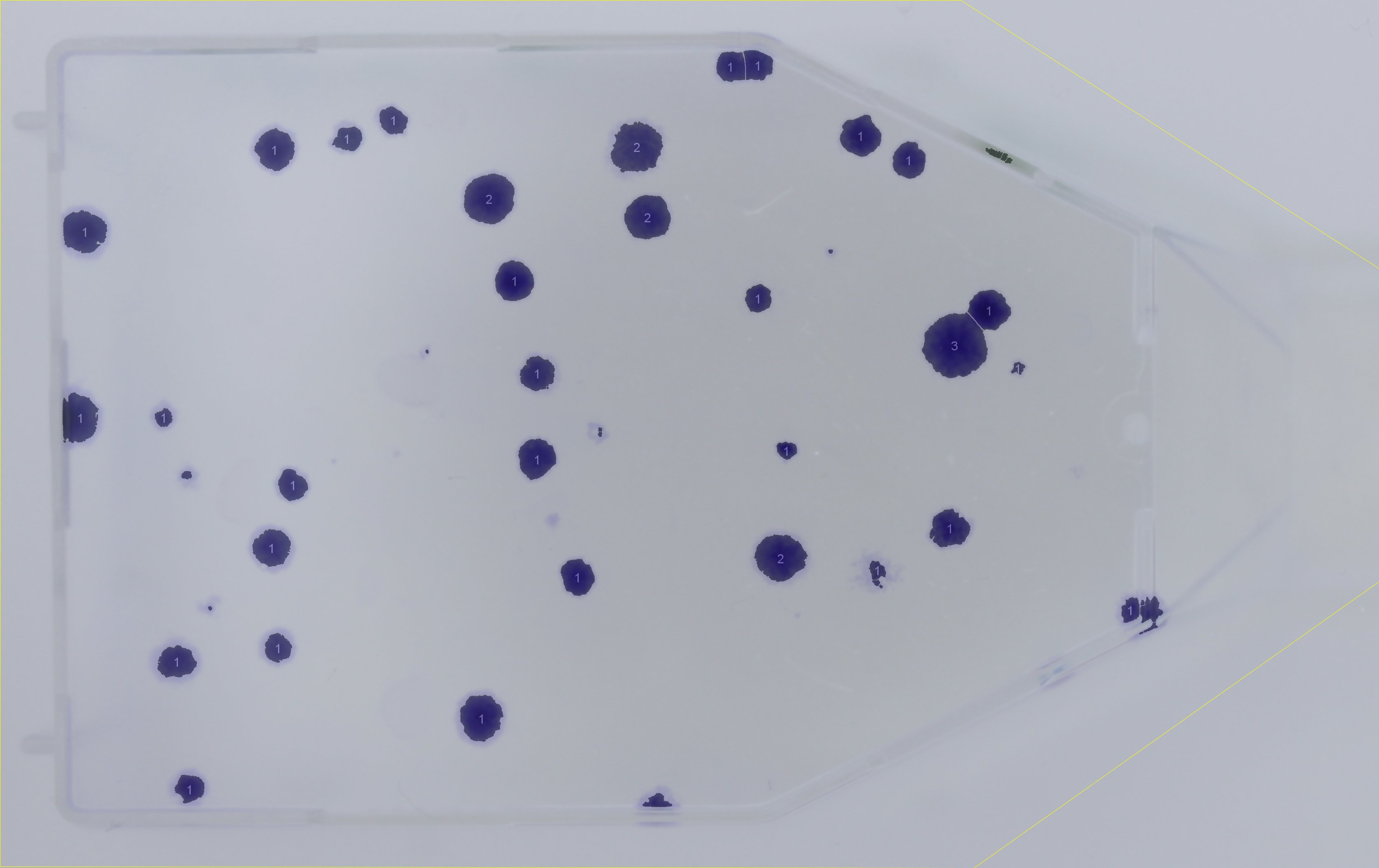

Supplement: S4 Datasets — It also contains a text file where results achieved by automated (CoCoNut, CAI, AutoCellSeg, and OpenCFU) and manual methods are summarized. (ZIP) [file pone.0205823.s005.zip › 180501 HeLa Flask/2 Results.jpg]

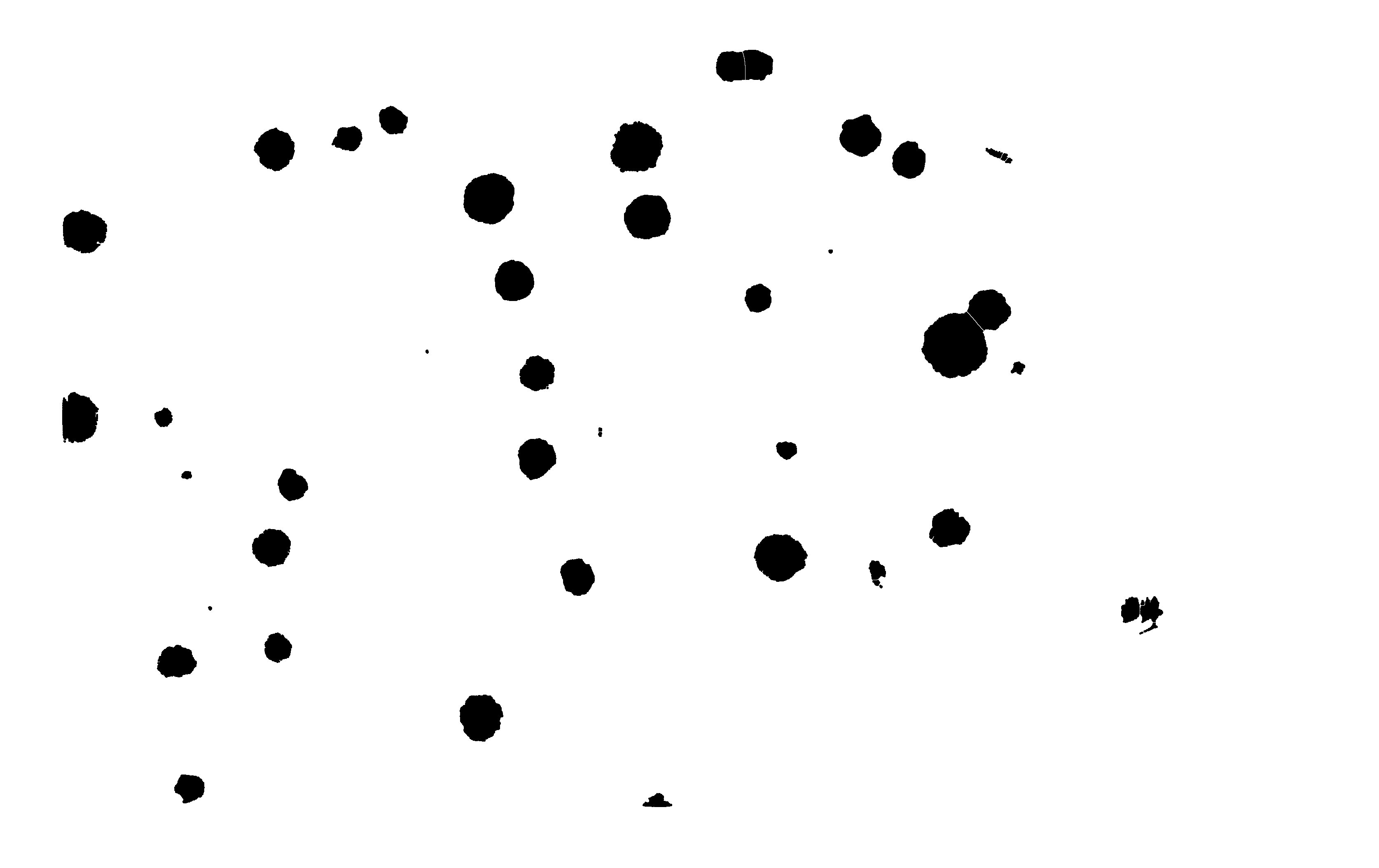

Supplement: S4 Datasets — It also contains a text file where results achieved by automated (CoCoNut, CAI, AutoCellSeg, and OpenCFU) and manual methods are summarized. (ZIP) [file pone.0205823.s005.zip › 180501 HeLa Flask/2 Second counting.jpg]

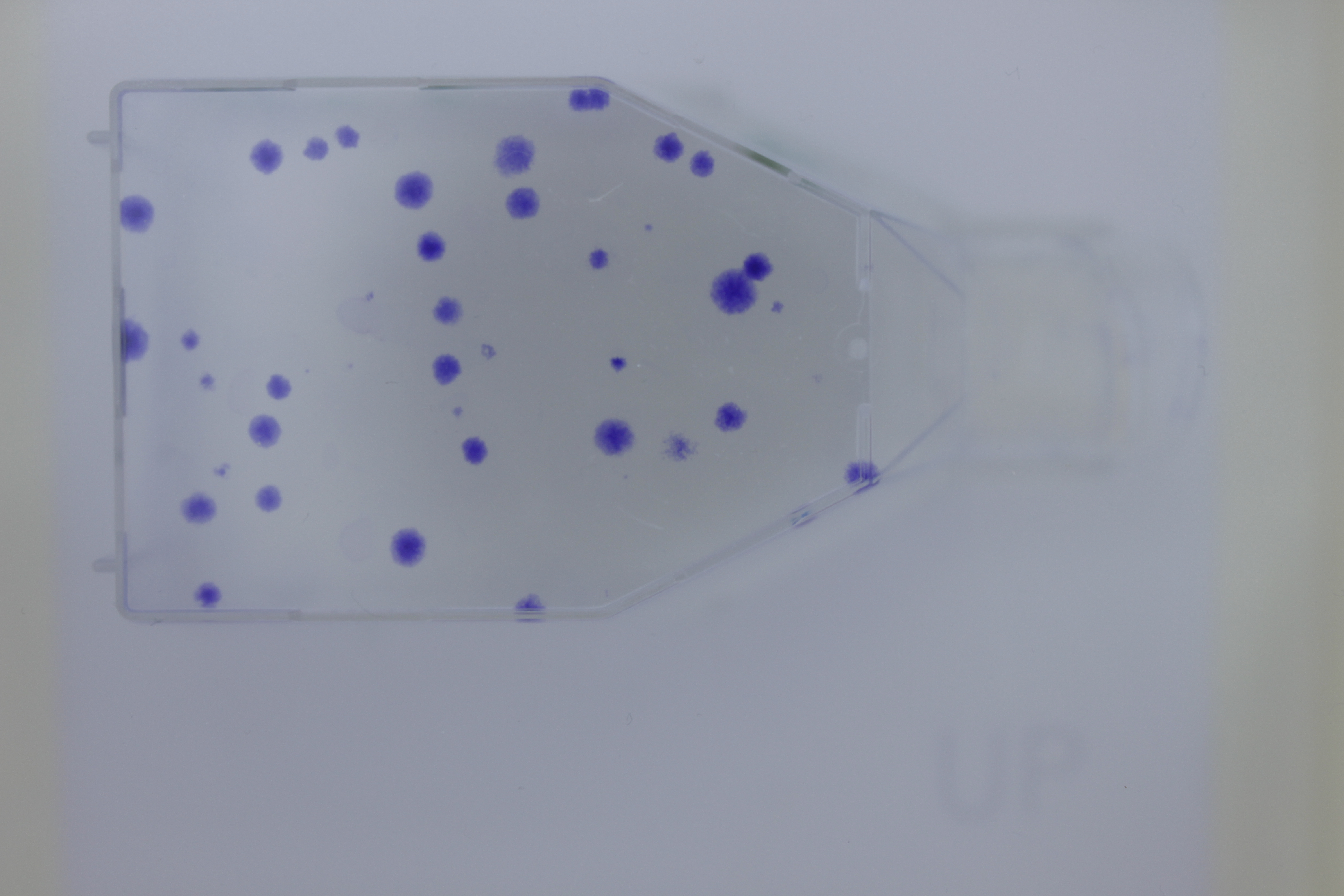

Supplement: S4 Datasets — It also contains a text file where results achieved by automated (CoCoNut, CAI, AutoCellSeg, and OpenCFU) and manual methods are summarized. (ZIP) [file pone.0205823.s005.zip › 180501 HeLa Flask/2.JPG]

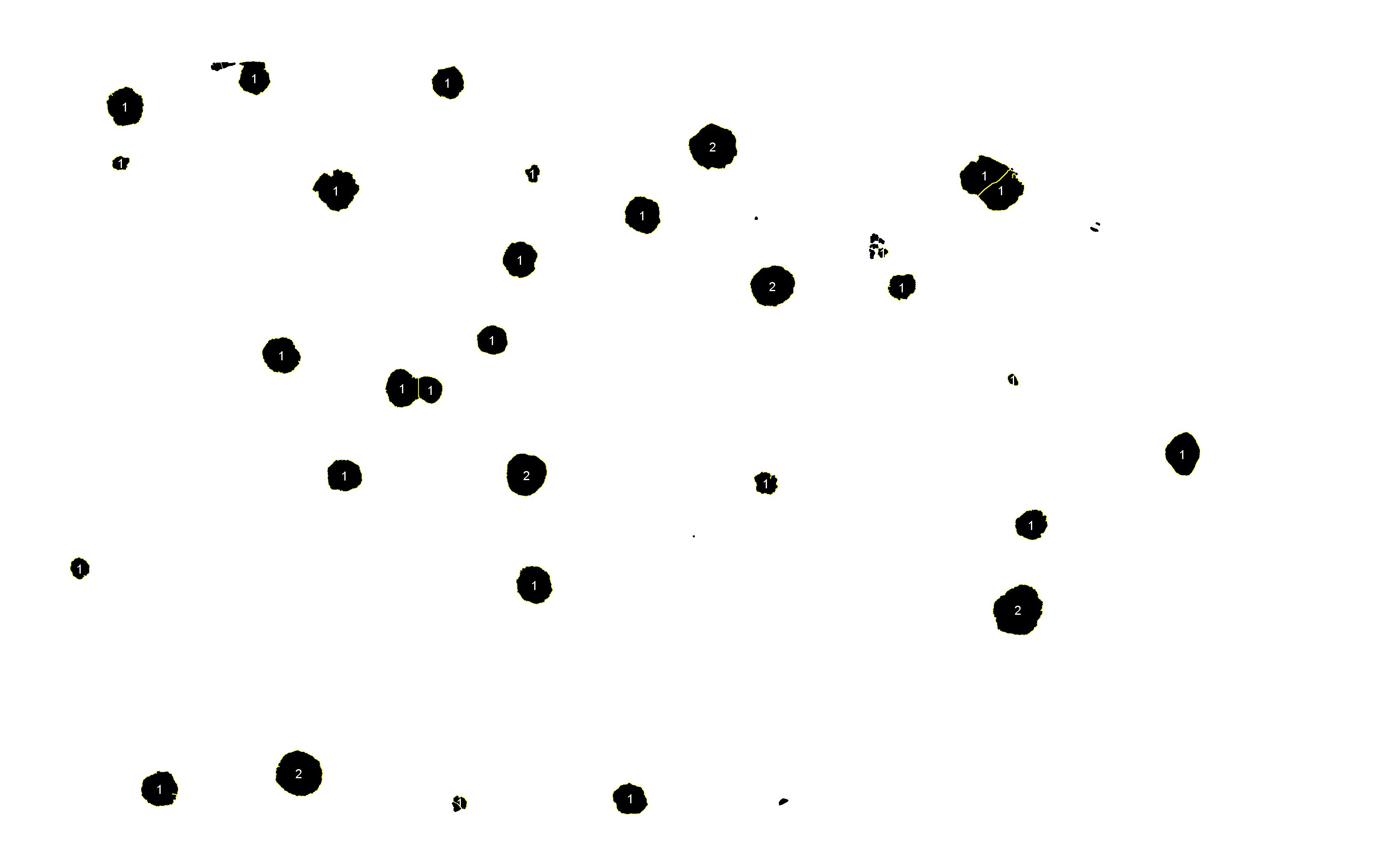

Supplement: S4 Datasets — It also contains a text file where results achieved by automated (CoCoNut, CAI, AutoCellSeg, and OpenCFU) and manual methods are summarized. (ZIP) [file pone.0205823.s005.zip › 180501 HeLa Flask/3 First counting.jpg]

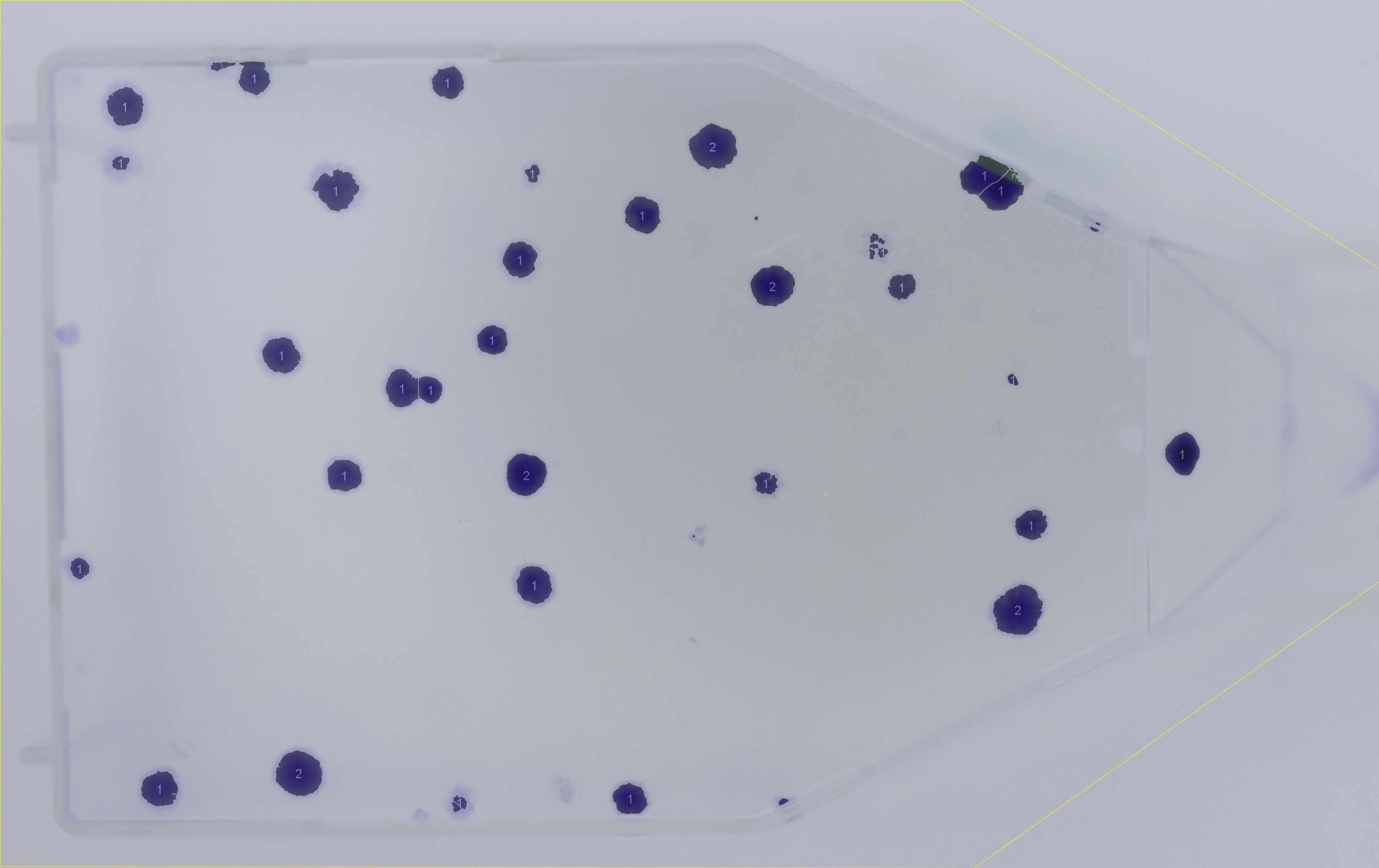

Supplement: S4 Datasets — It also contains a text file where results achieved by automated (CoCoNut, CAI, AutoCellSeg, and OpenCFU) and manual methods are summarized. (ZIP) [file pone.0205823.s005.zip › 180501 HeLa Flask/3 Results.jpg]

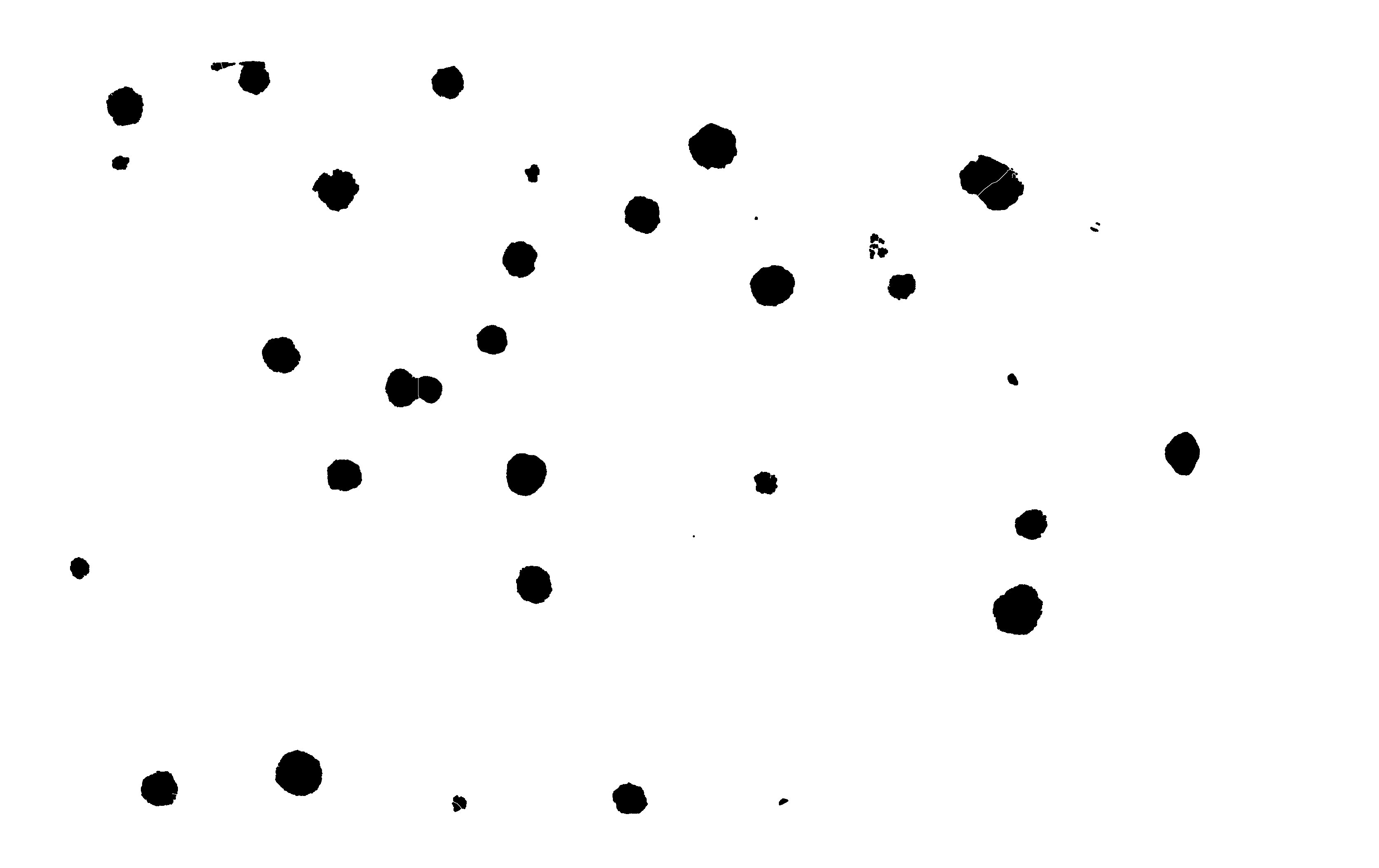

Supplement: S4 Datasets — It also contains a text file where results achieved by automated (CoCoNut, CAI, AutoCellSeg, and OpenCFU) and manual methods are summarized. (ZIP) [file pone.0205823.s005.zip › 180501 HeLa Flask/3 Second counting.jpg]

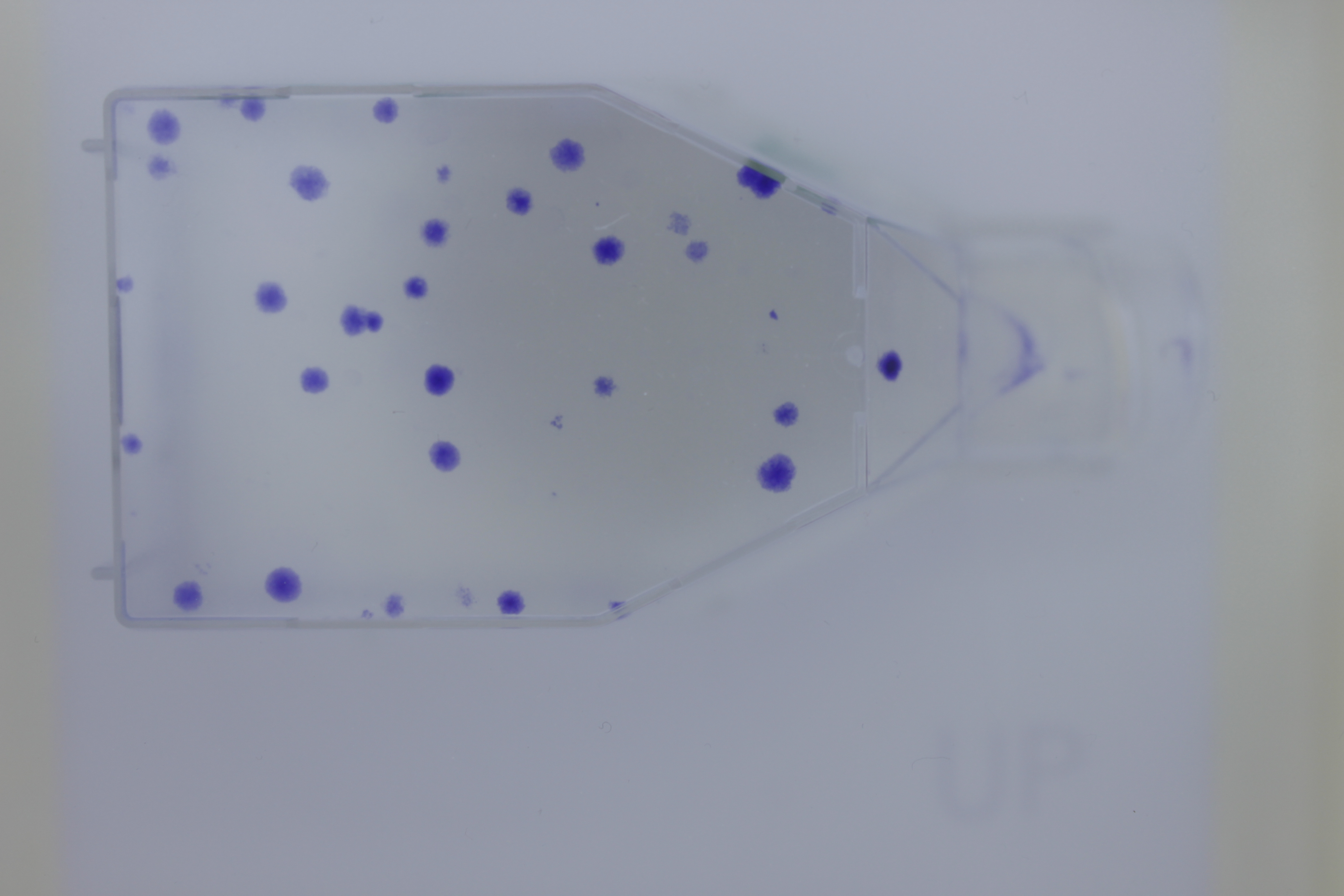

Supplement: S4 Datasets — It also contains a text file where results achieved by automated (CoCoNut, CAI, AutoCellSeg, and OpenCFU) and manual methods are summarized. (ZIP) [file pone.0205823.s005.zip › 180501 HeLa Flask/3.JPG]

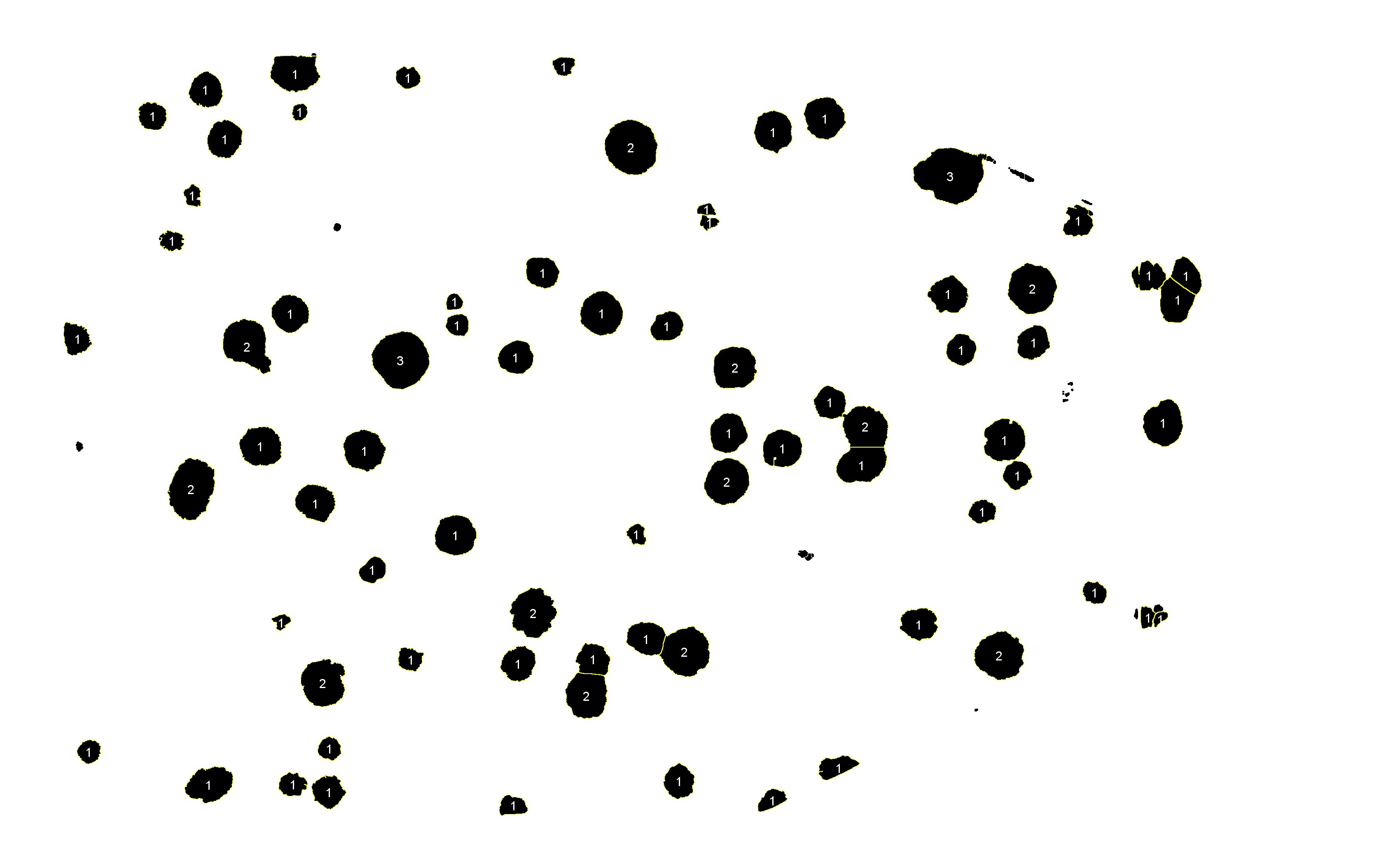

Supplement: S4 Datasets — It also contains a text file where results achieved by automated (CoCoNut, CAI, AutoCellSeg, and OpenCFU) and manual methods are summarized. (ZIP) [file pone.0205823.s005.zip › 180501 HeLa Flask/4 First counting.jpg]

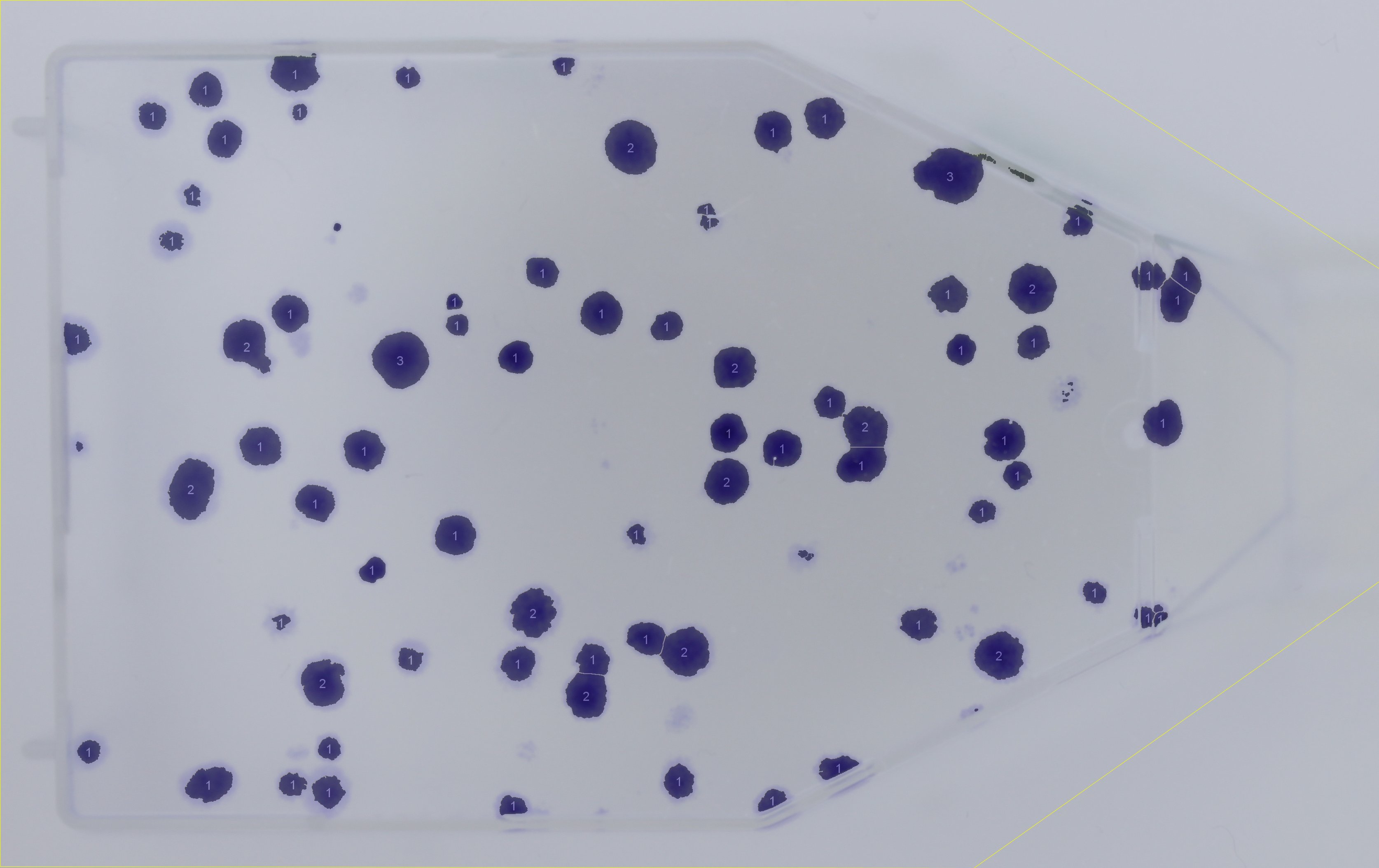

Supplement: S4 Datasets — It also contains a text file where results achieved by automated (CoCoNut, CAI, AutoCellSeg, and OpenCFU) and manual methods are summarized. (ZIP) [file pone.0205823.s005.zip › 180501 HeLa Flask/4 Results.jpg]

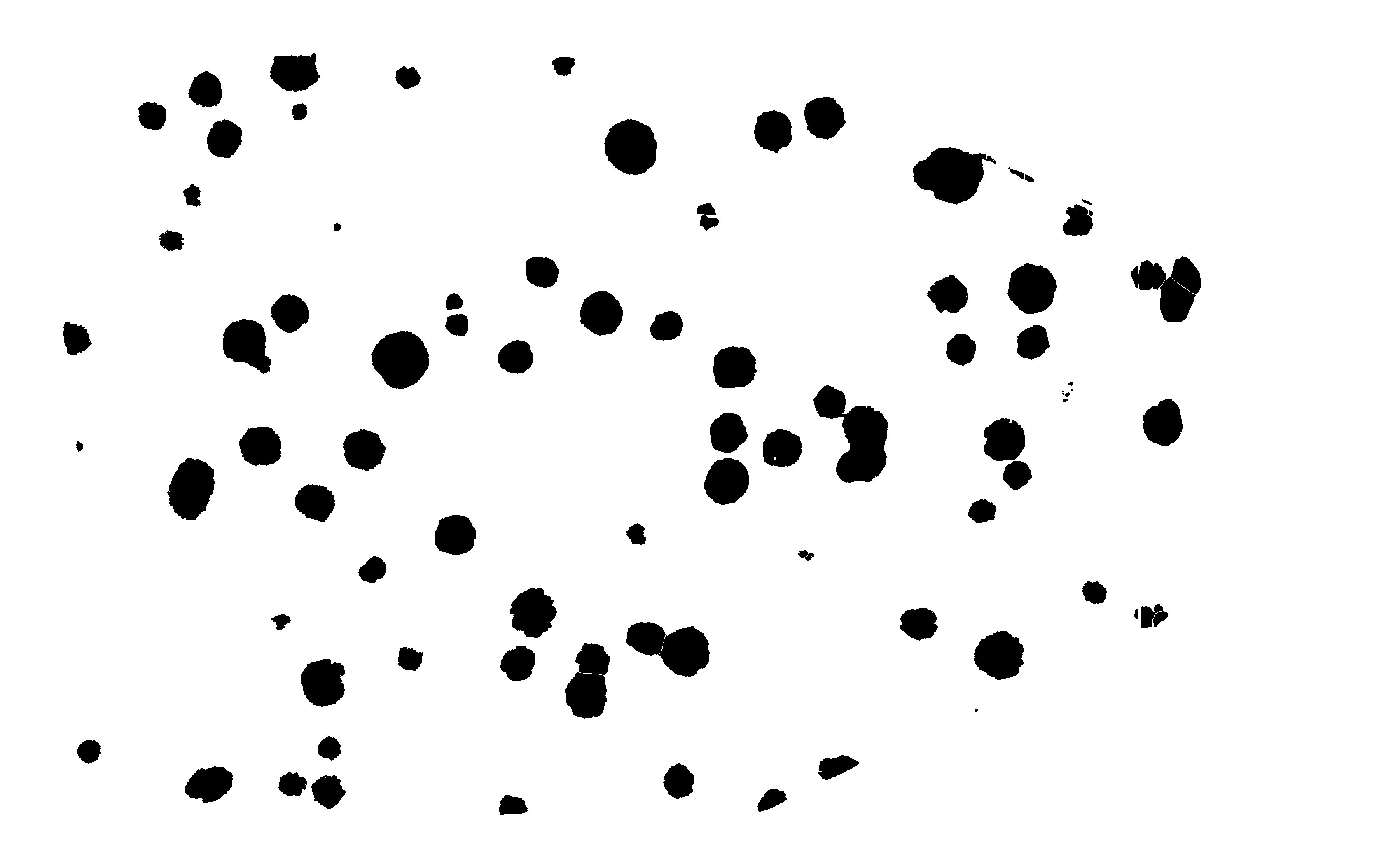

Supplement: S4 Datasets — It also contains a text file where results achieved by automated (CoCoNut, CAI, AutoCellSeg, and OpenCFU) and manual methods are summarized. (ZIP) [file pone.0205823.s005.zip › 180501 HeLa Flask/4 Second counting.jpg]

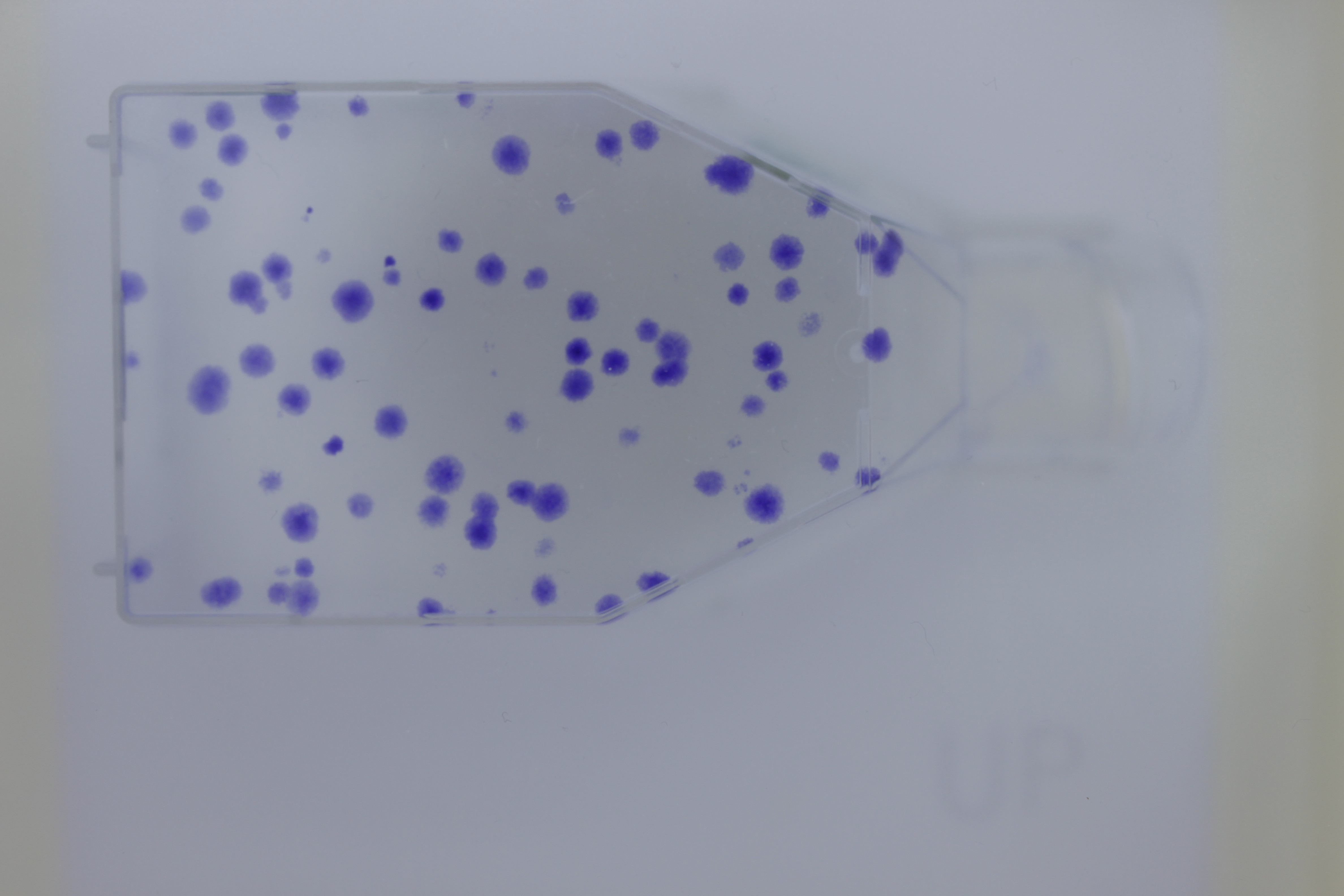

Supplement: S4 Datasets — It also contains a text file where results achieved by automated (CoCoNut, CAI, AutoCellSeg, and OpenCFU) and manual methods are summarized. (ZIP) [file pone.0205823.s005.zip › 180501 HeLa Flask/4.JPG]

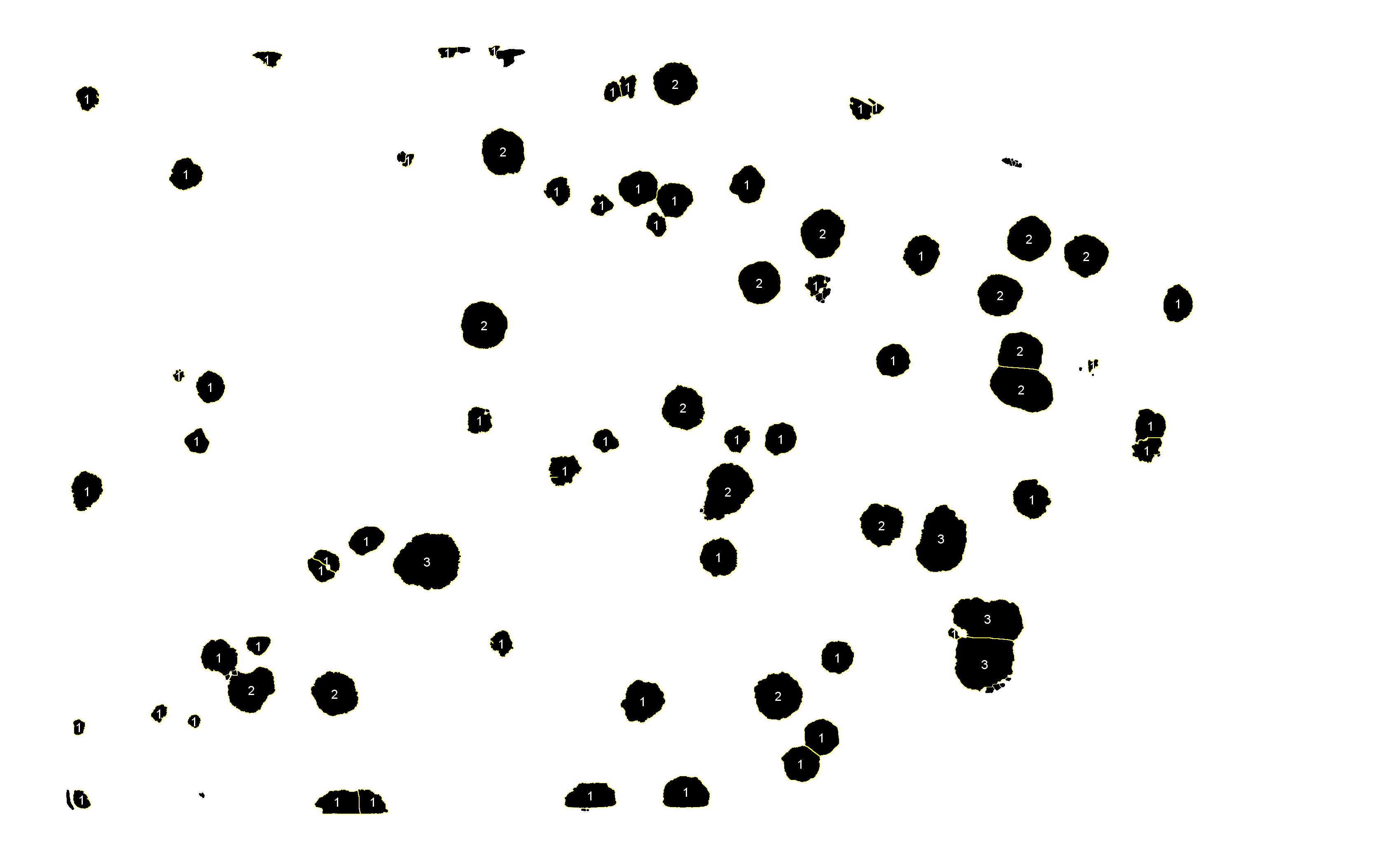

Supplement: S4 Datasets — It also contains a text file where results achieved by automated (CoCoNut, CAI, AutoCellSeg, and OpenCFU) and manual methods are summarized. (ZIP) [file pone.0205823.s005.zip › 180501 HeLa Flask/5 First counting.jpg]

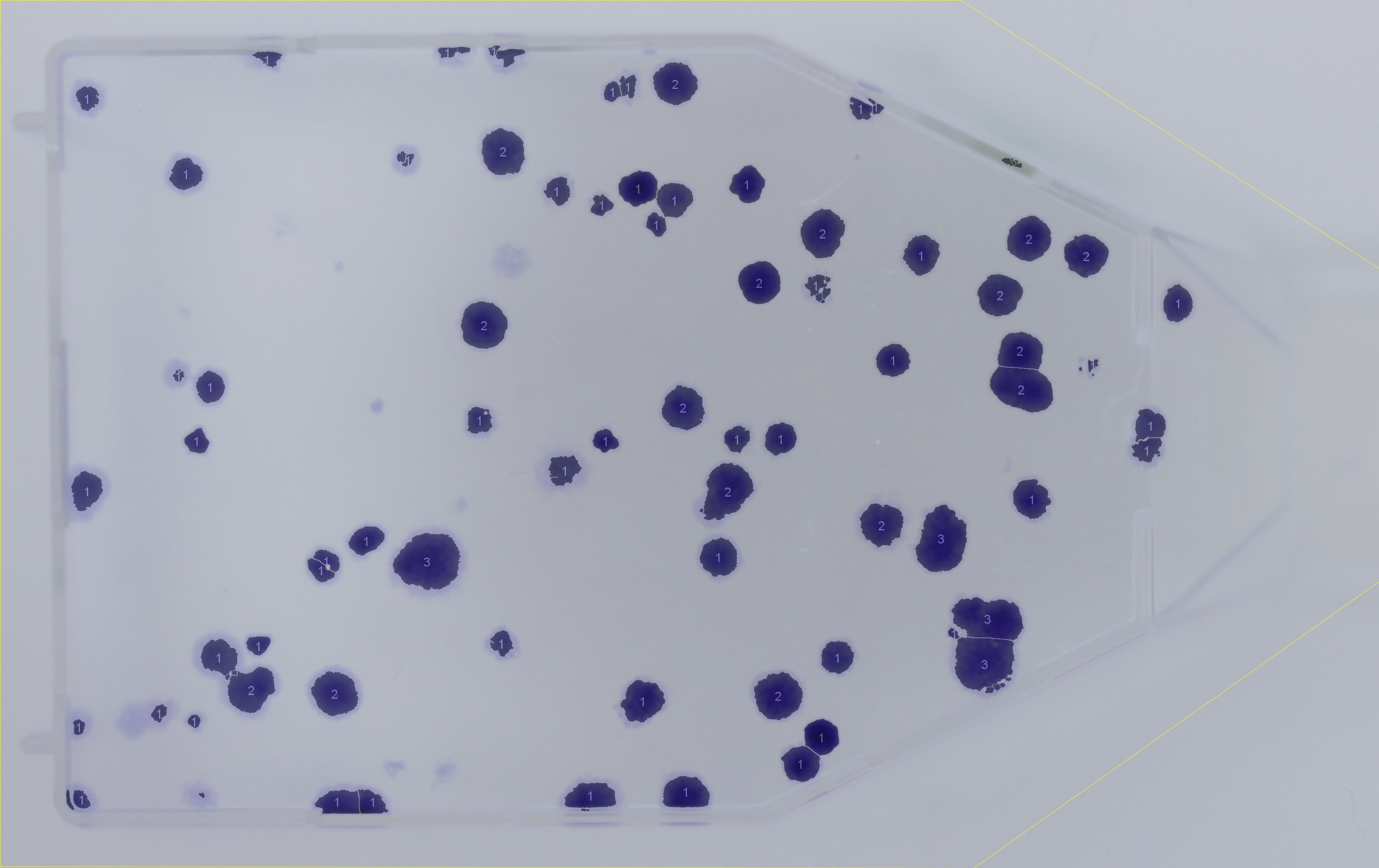

Supplement: S4 Datasets — It also contains a text file where results achieved by automated (CoCoNut, CAI, AutoCellSeg, and OpenCFU) and manual methods are summarized. (ZIP) [file pone.0205823.s005.zip › 180501 HeLa Flask/5 Results.jpg]

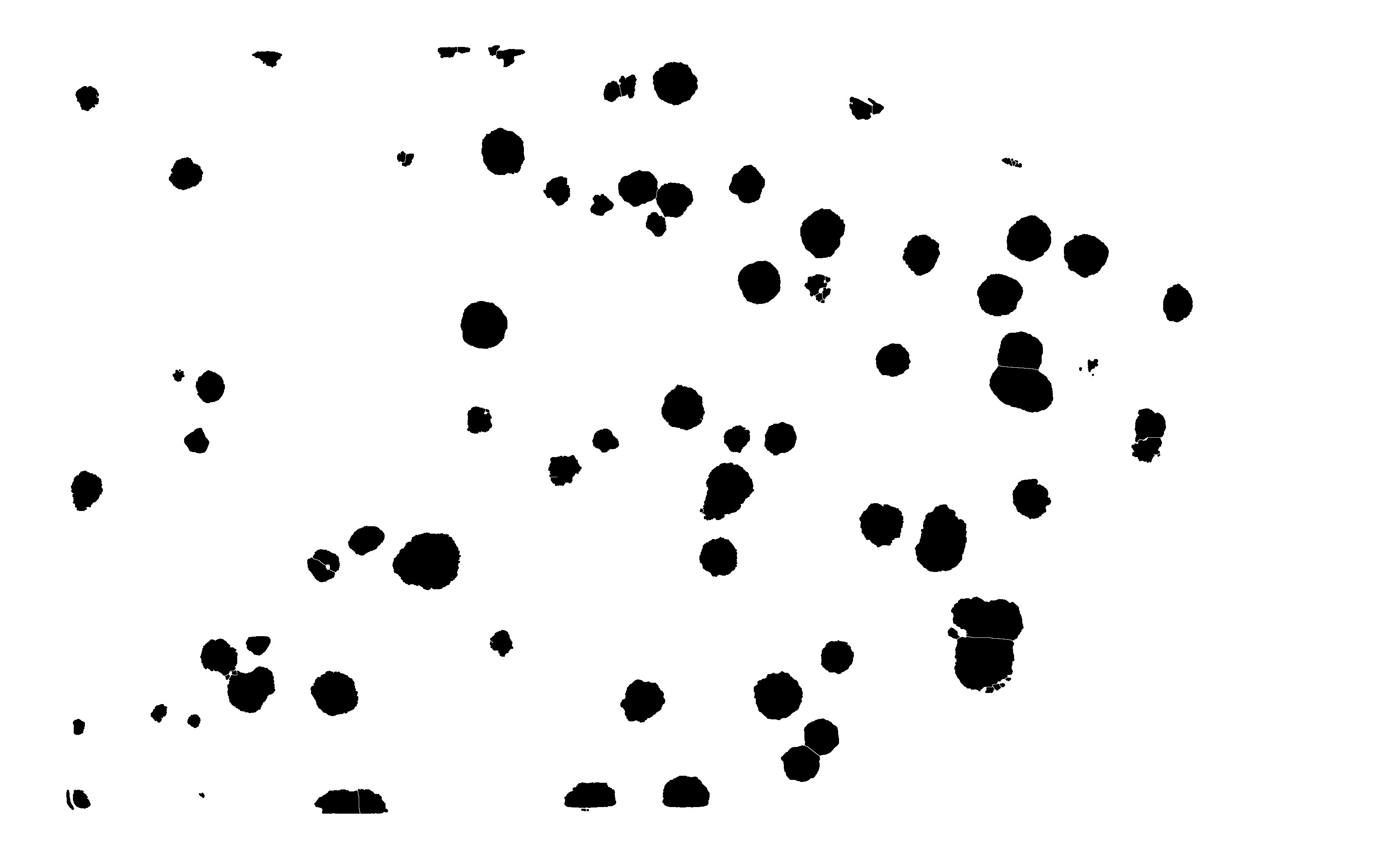

Supplement: S4 Datasets — It also contains a text file where results achieved by automated (CoCoNut, CAI, AutoCellSeg, and OpenCFU) and manual methods are summarized. (ZIP) [file pone.0205823.s005.zip › 180501 HeLa Flask/5 Second counting.jpg]

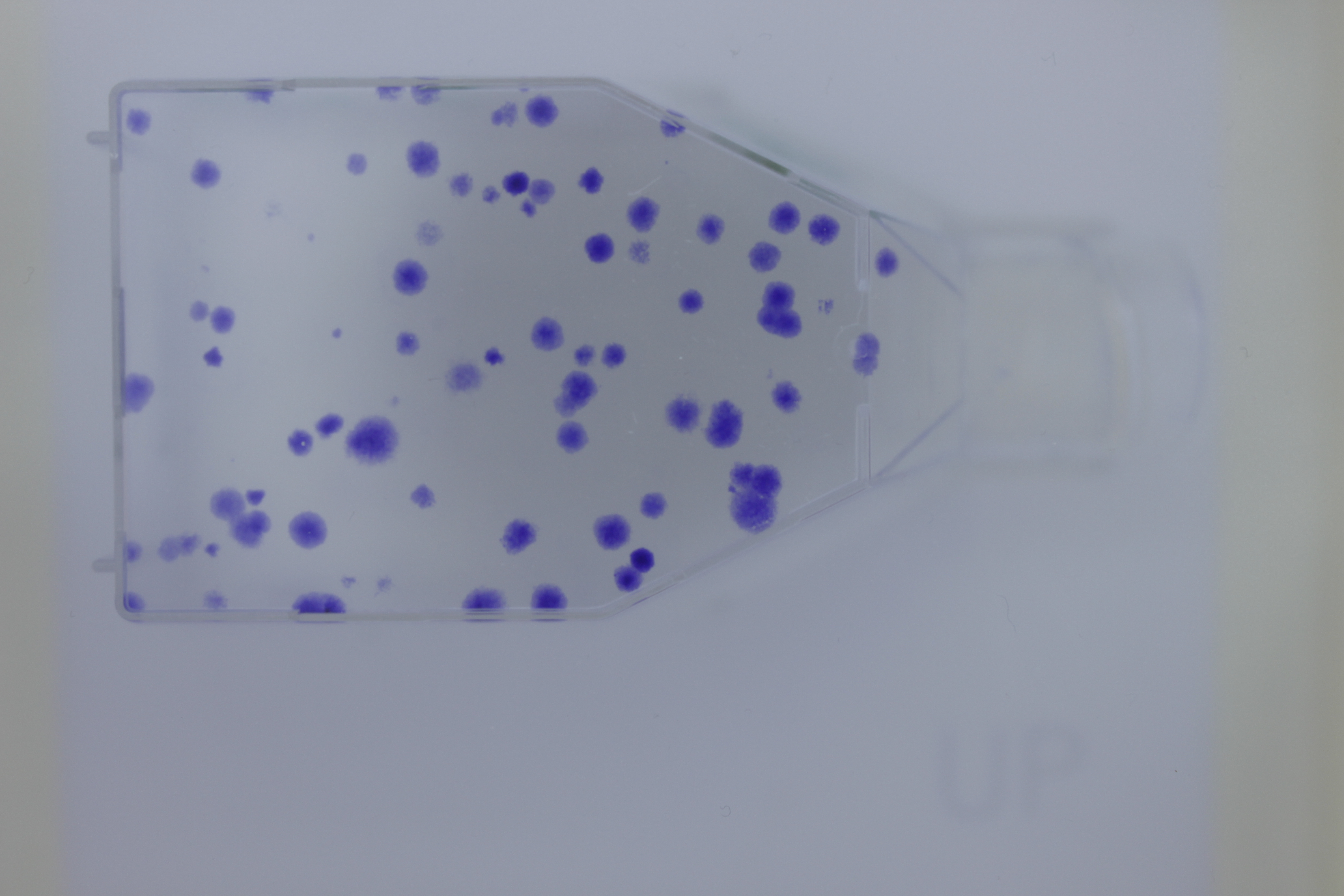

Supplement: S4 Datasets — It also contains a text file where results achieved by automated (CoCoNut, CAI, AutoCellSeg, and OpenCFU) and manual methods are summarized. (ZIP) [file pone.0205823.s005.zip › 180501 HeLa Flask/5.JPG]

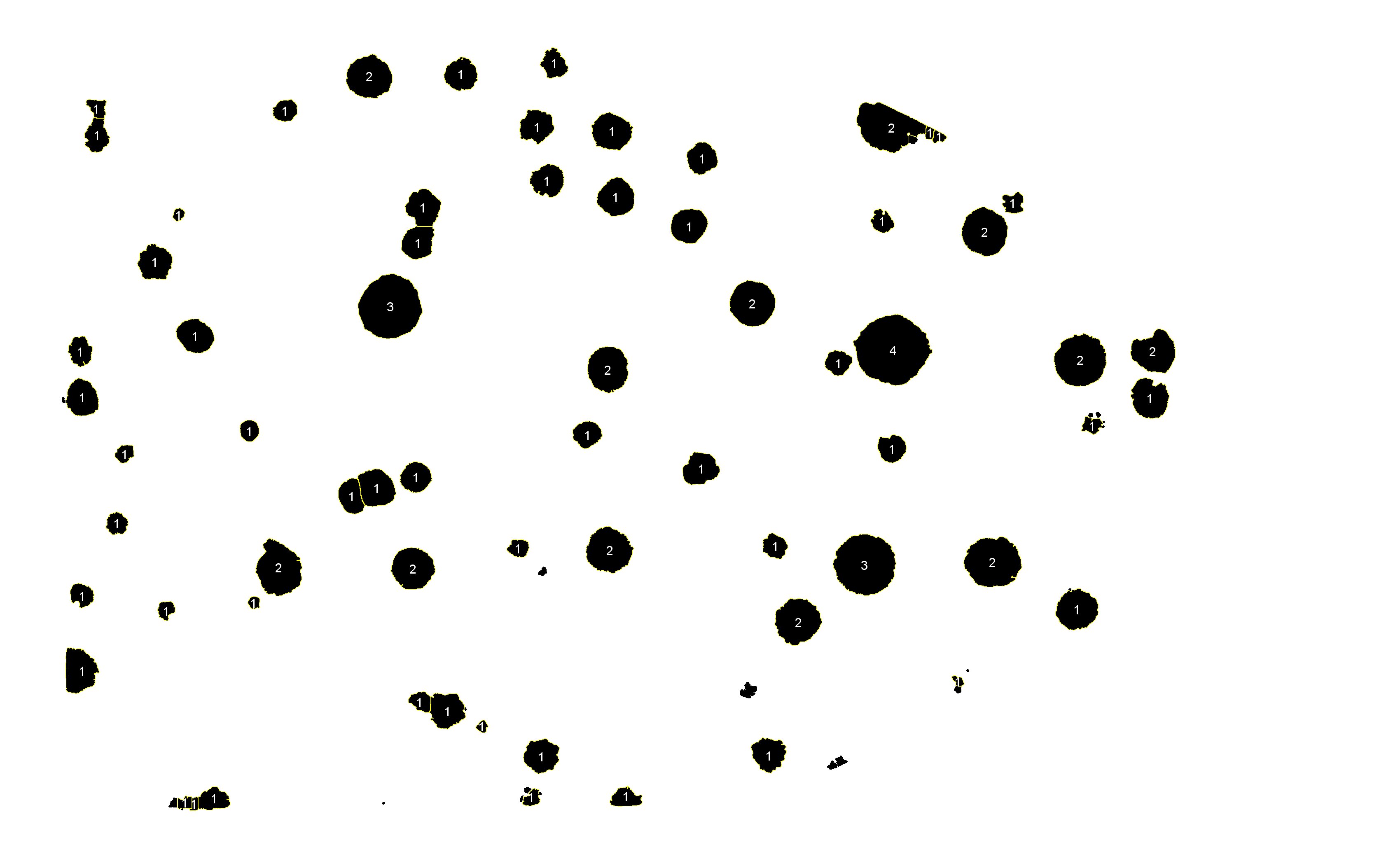

Supplement: S4 Datasets — It also contains a text file where results achieved by automated (CoCoNut, CAI, AutoCellSeg, and OpenCFU) and manual methods are summarized. (ZIP) [file pone.0205823.s005.zip › 180501 HeLa Flask/6 First counting.jpg]

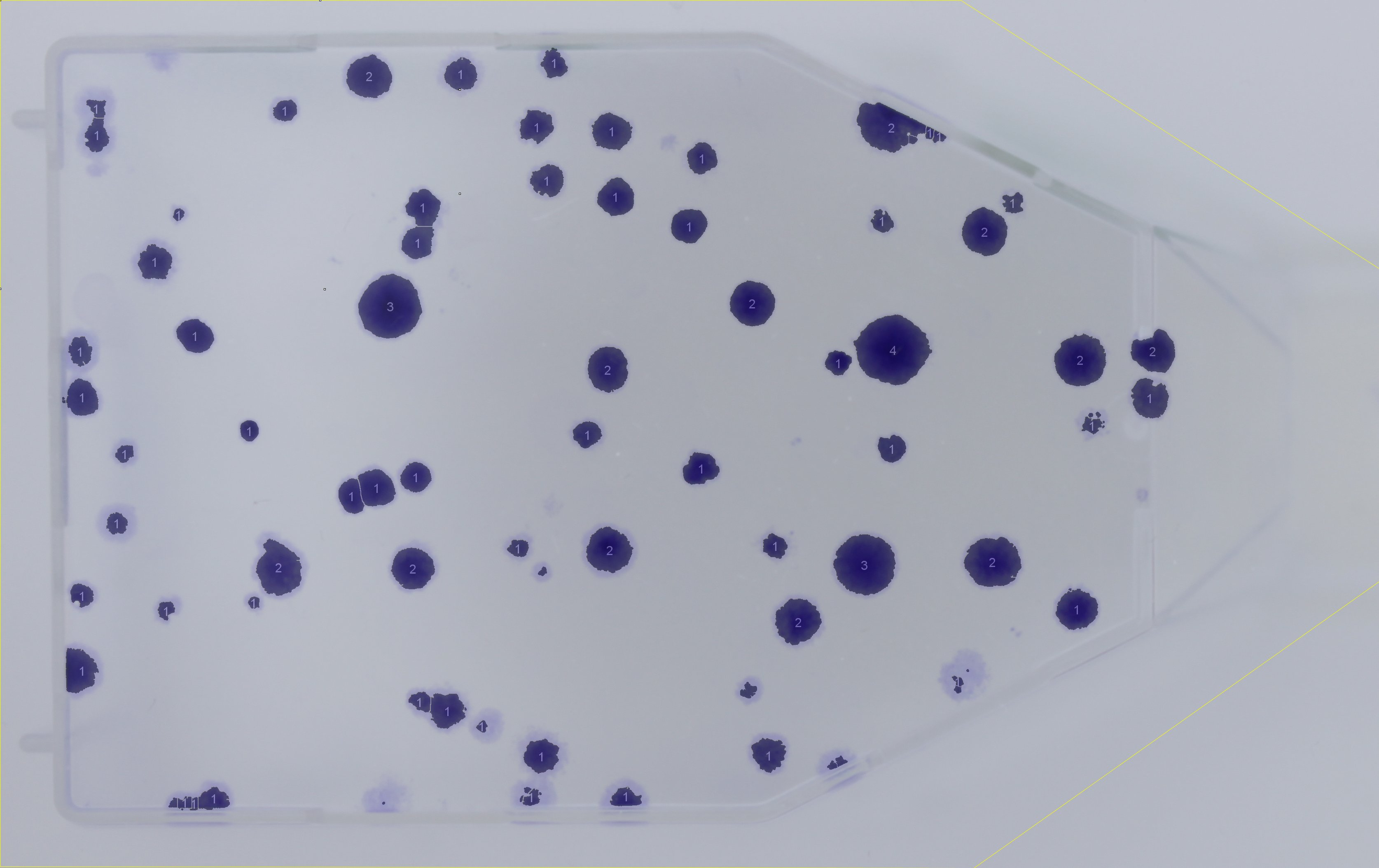

Supplement: S4 Datasets — It also contains a text file where results achieved by automated (CoCoNut, CAI, AutoCellSeg, and OpenCFU) and manual methods are summarized. (ZIP) [file pone.0205823.s005.zip › 180501 HeLa Flask/6 Results.jpg]

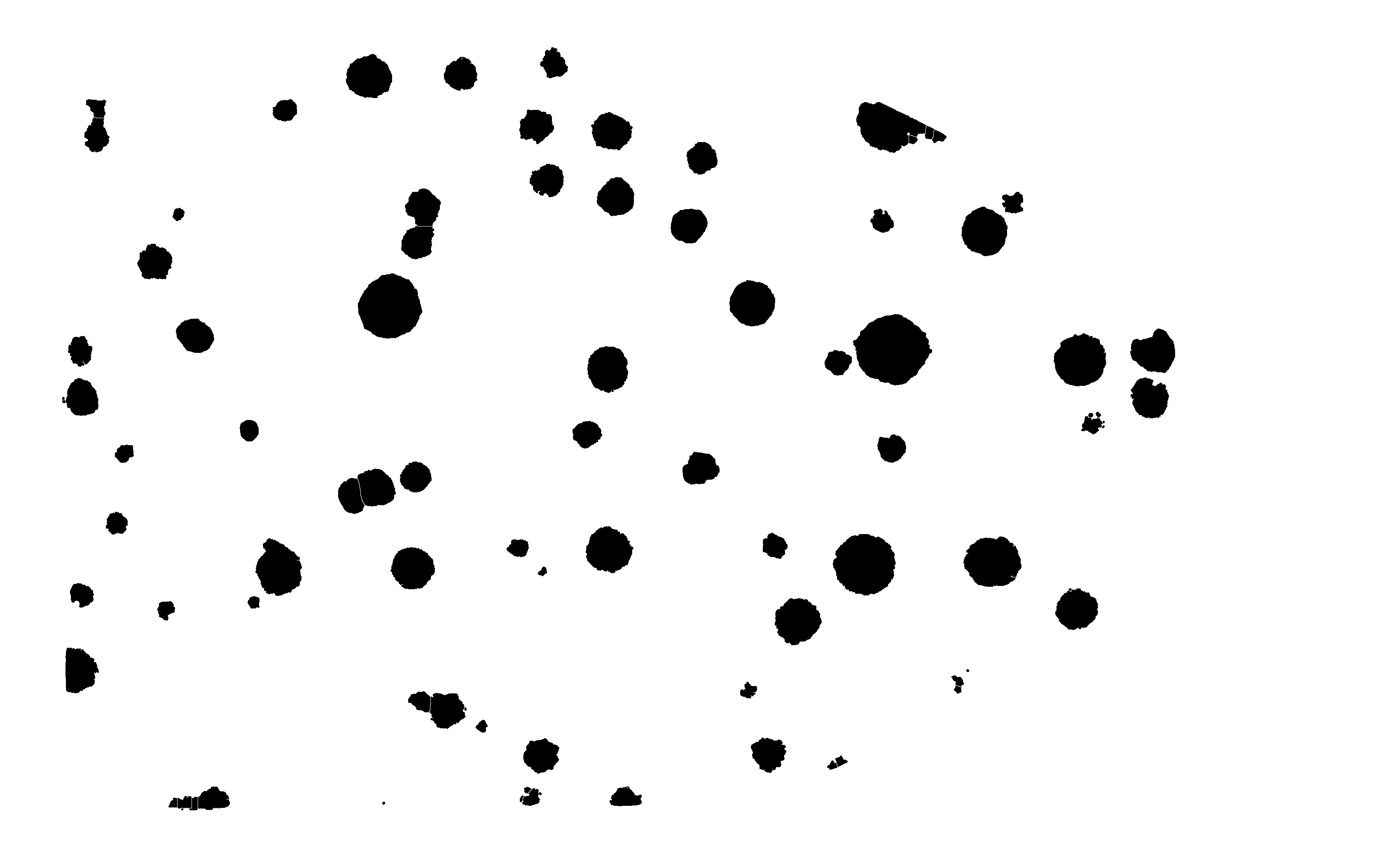

Supplement: S4 Datasets — It also contains a text file where results achieved by automated (CoCoNut, CAI, AutoCellSeg, and OpenCFU) and manual methods are summarized. (ZIP) [file pone.0205823.s005.zip › 180501 HeLa Flask/6 Second counting.jpg]

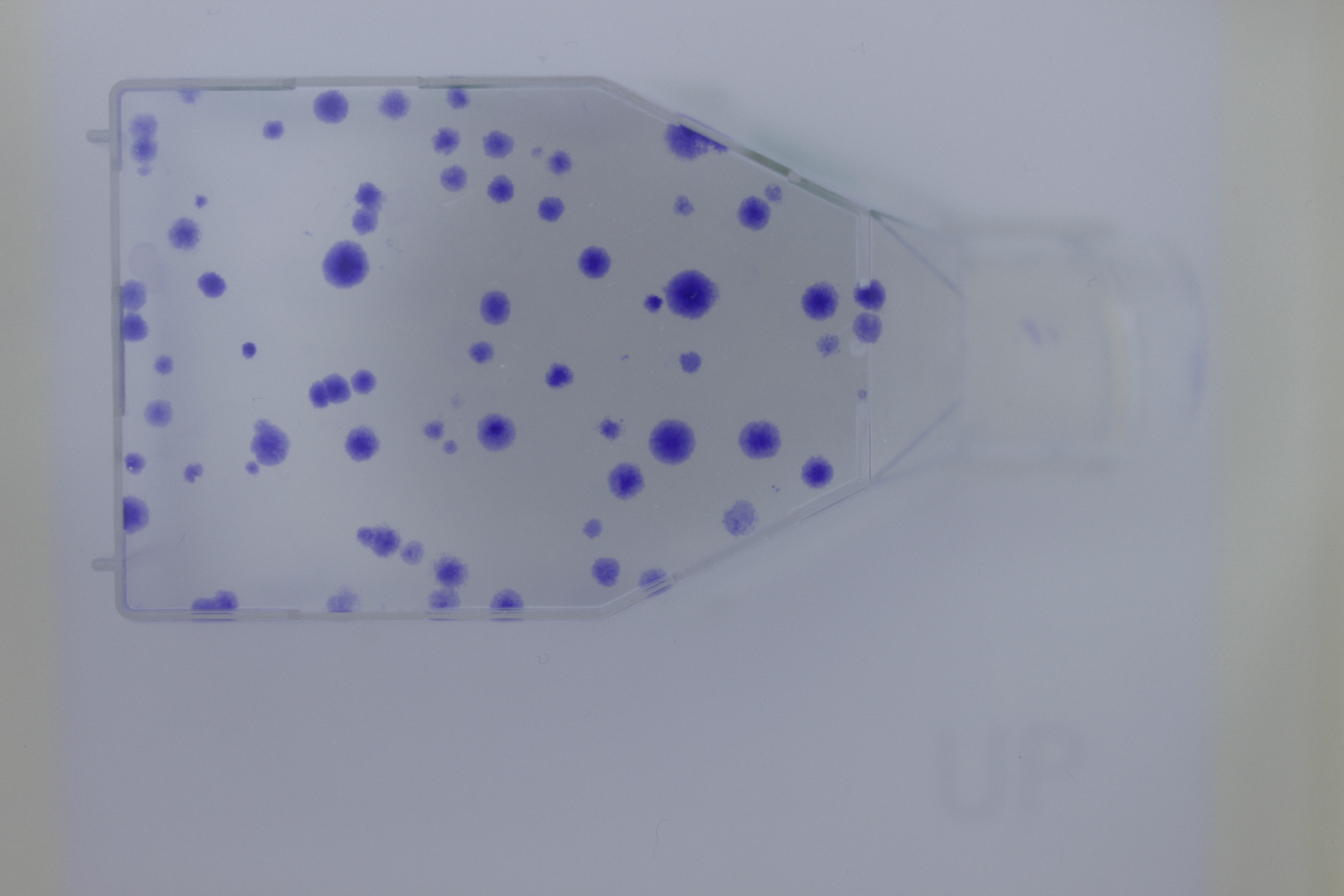

Supplement: S4 Datasets — It also contains a text file where results achieved by automated (CoCoNut, CAI, AutoCellSeg, and OpenCFU) and manual methods are summarized. (ZIP) [file pone.0205823.s005.zip › 180501 HeLa Flask/6.JPG]

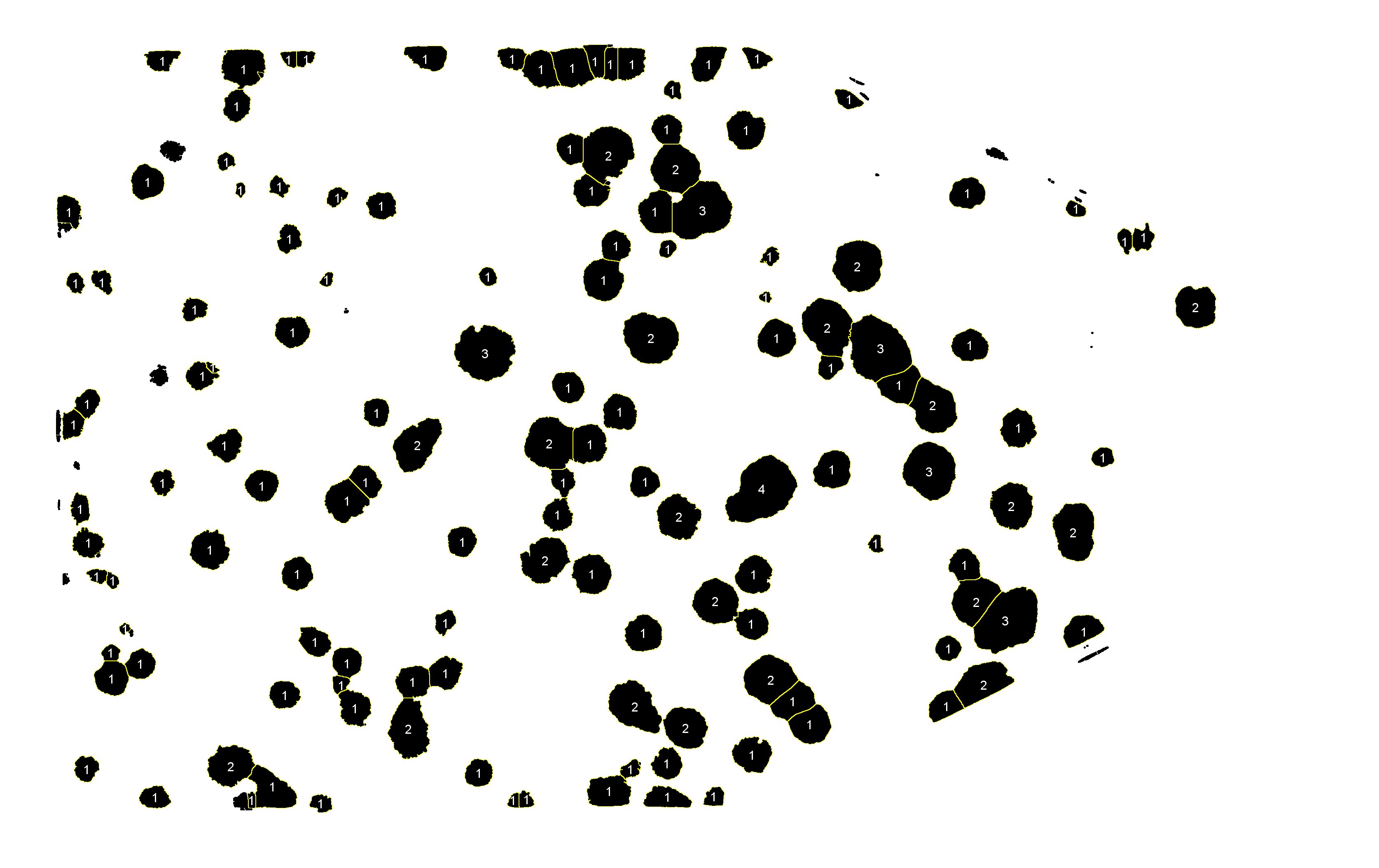

Supplement: S4 Datasets — It also contains a text file where results achieved by automated (CoCoNut, CAI, AutoCellSeg, and OpenCFU) and manual methods are summarized. (ZIP) [file pone.0205823.s005.zip › 180501 HeLa Flask/7 First counting.jpg]

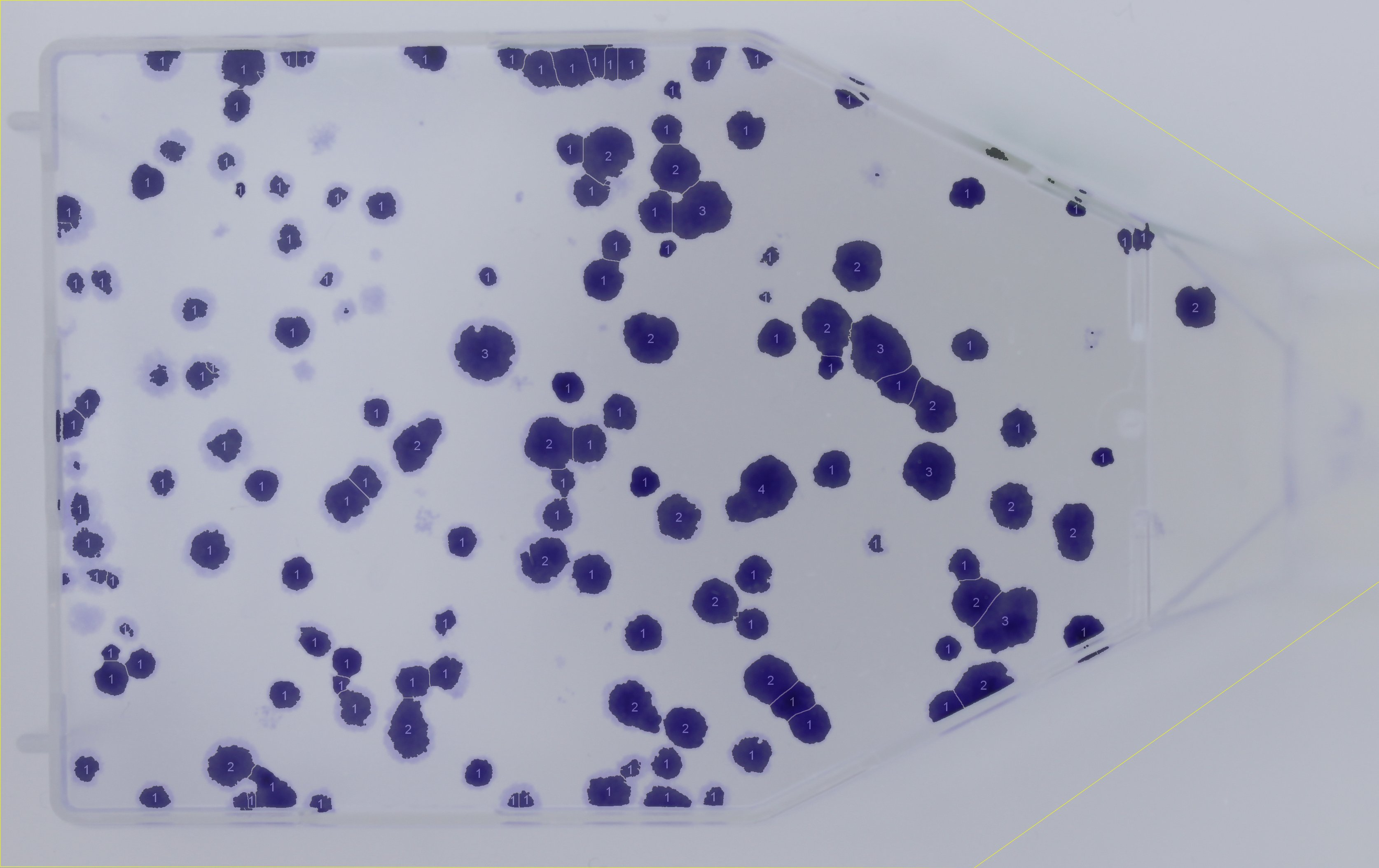

Supplement: S4 Datasets — It also contains a text file where results achieved by automated (CoCoNut, CAI, AutoCellSeg, and OpenCFU) and manual methods are summarized. (ZIP) [file pone.0205823.s005.zip › 180501 HeLa Flask/7 Results.jpg]

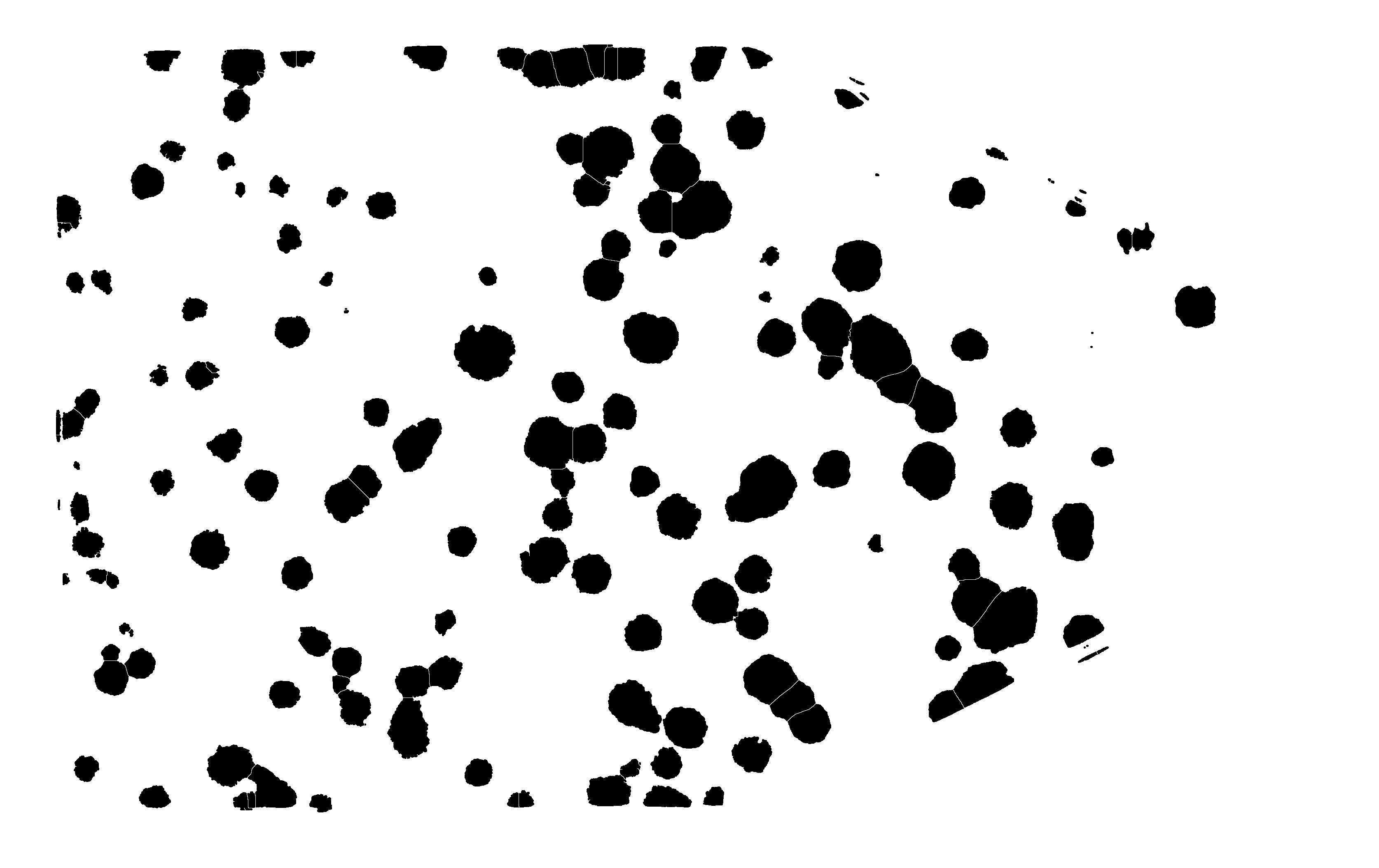

Supplement: S4 Datasets — It also contains a text file where results achieved by automated (CoCoNut, CAI, AutoCellSeg, and OpenCFU) and manual methods are summarized. (ZIP) [file pone.0205823.s005.zip › 180501 HeLa Flask/7 Second counting.jpg]

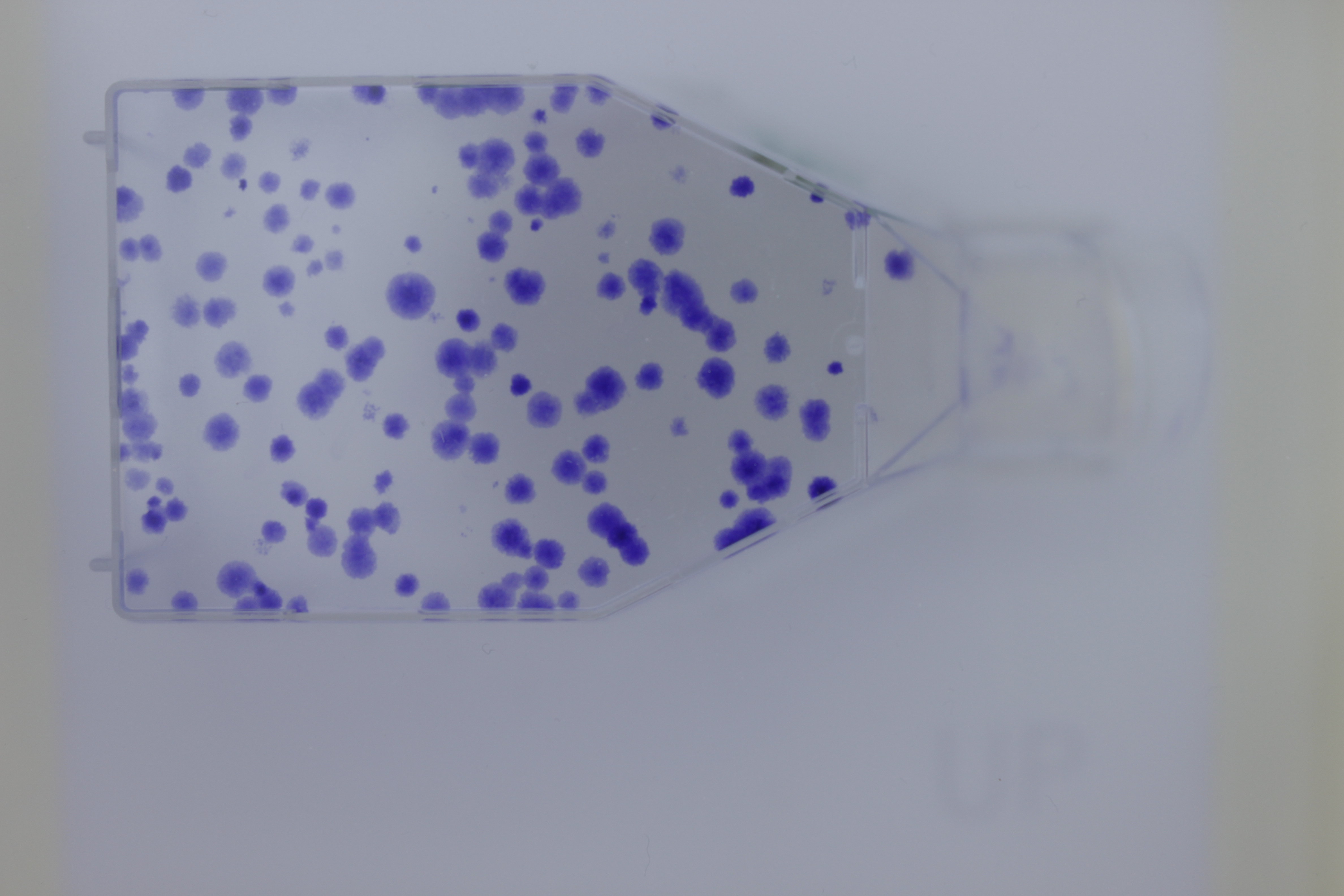

Supplement: S4 Datasets — It also contains a text file where results achieved by automated (CoCoNut, CAI, AutoCellSeg, and OpenCFU) and manual methods are summarized. (ZIP) [file pone.0205823.s005.zip › 180501 HeLa Flask/7.JPG]

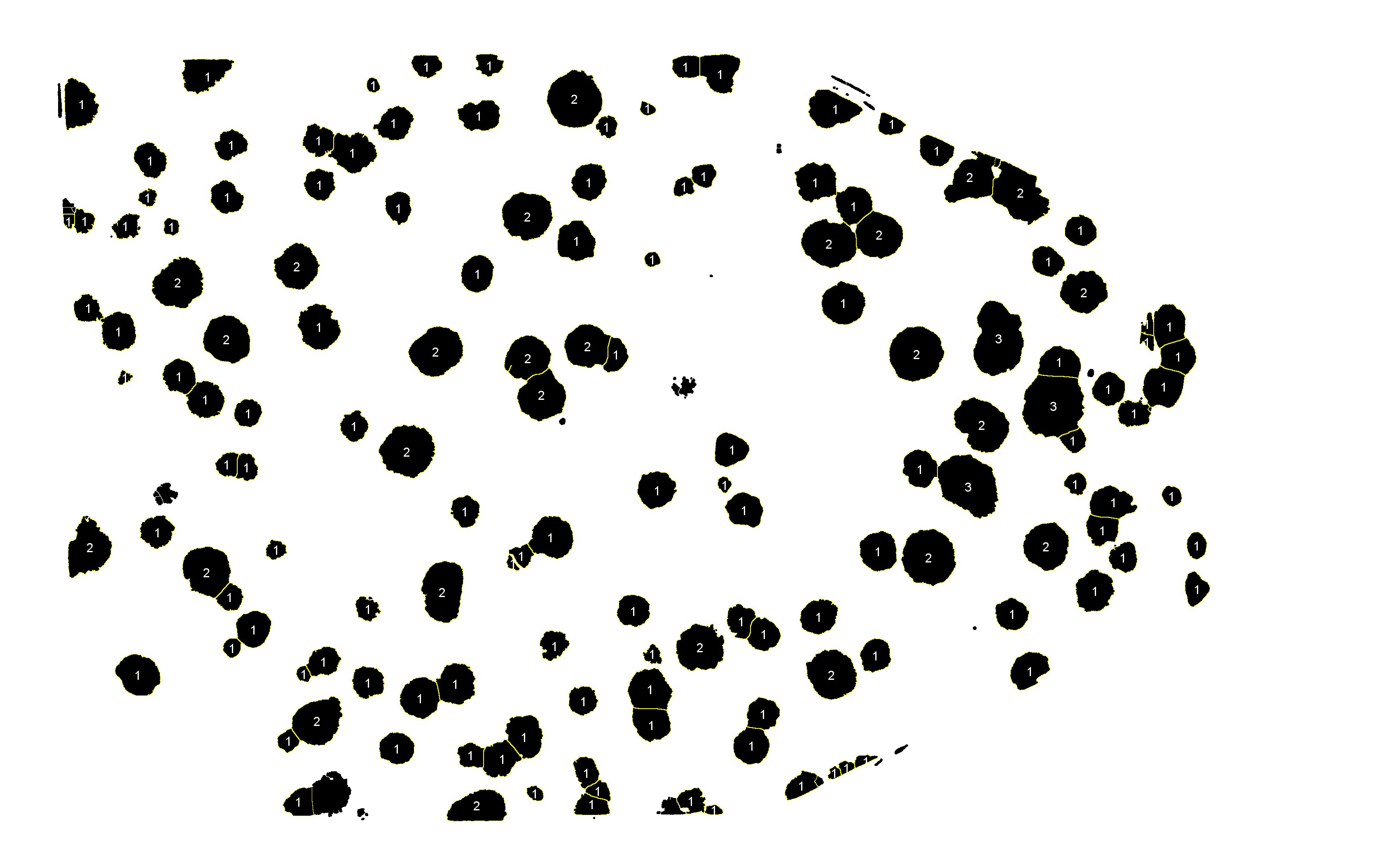

Supplement: S4 Datasets — It also contains a text file where results achieved by automated (CoCoNut, CAI, AutoCellSeg, and OpenCFU) and manual methods are summarized. (ZIP) [file pone.0205823.s005.zip › 180501 HeLa Flask/8 First counting.jpg]

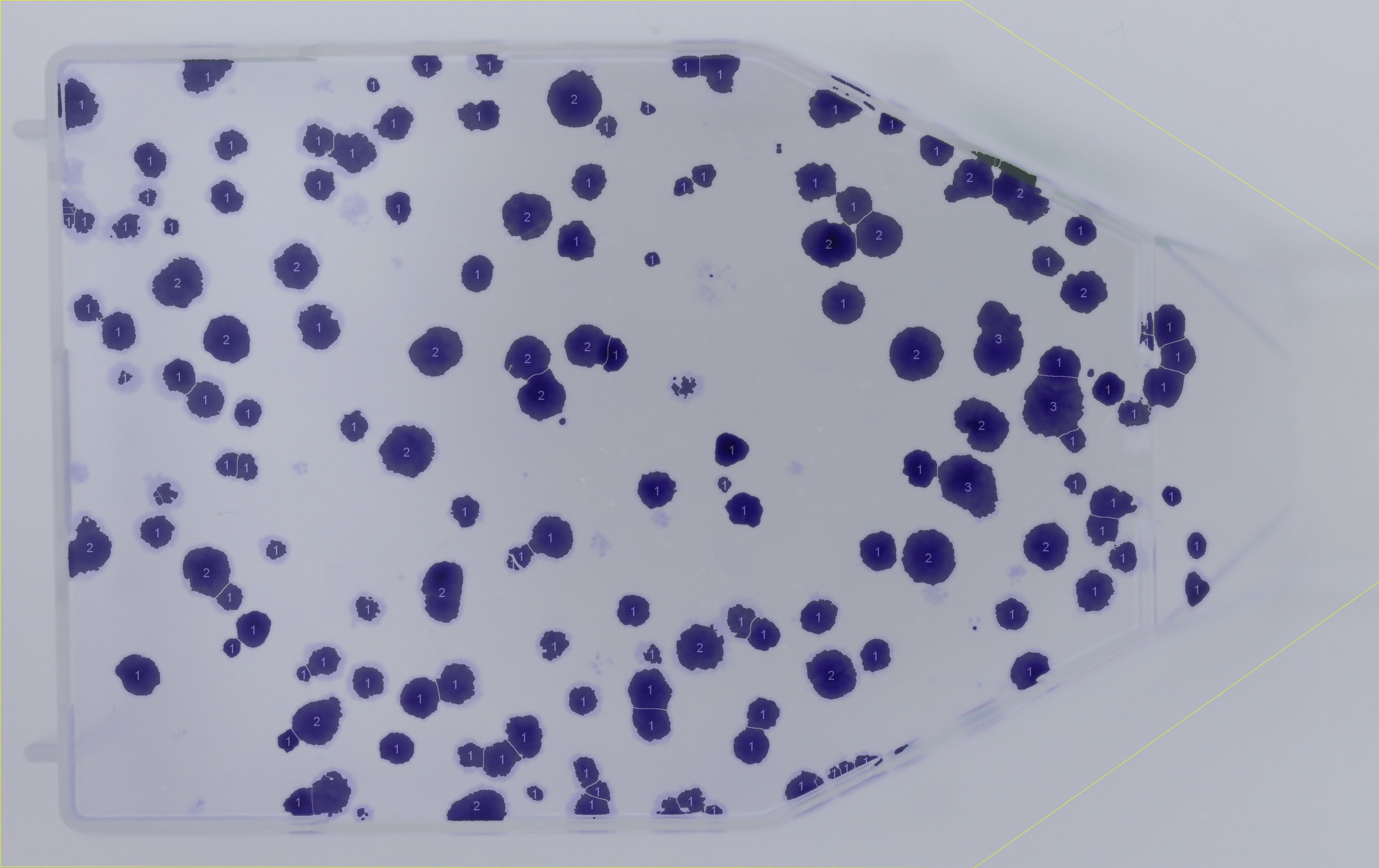

Supplement: S4 Datasets — It also contains a text file where results achieved by automated (CoCoNut, CAI, AutoCellSeg, and OpenCFU) and manual methods are summarized. (ZIP) [file pone.0205823.s005.zip › 180501 HeLa Flask/8 Results.jpg]

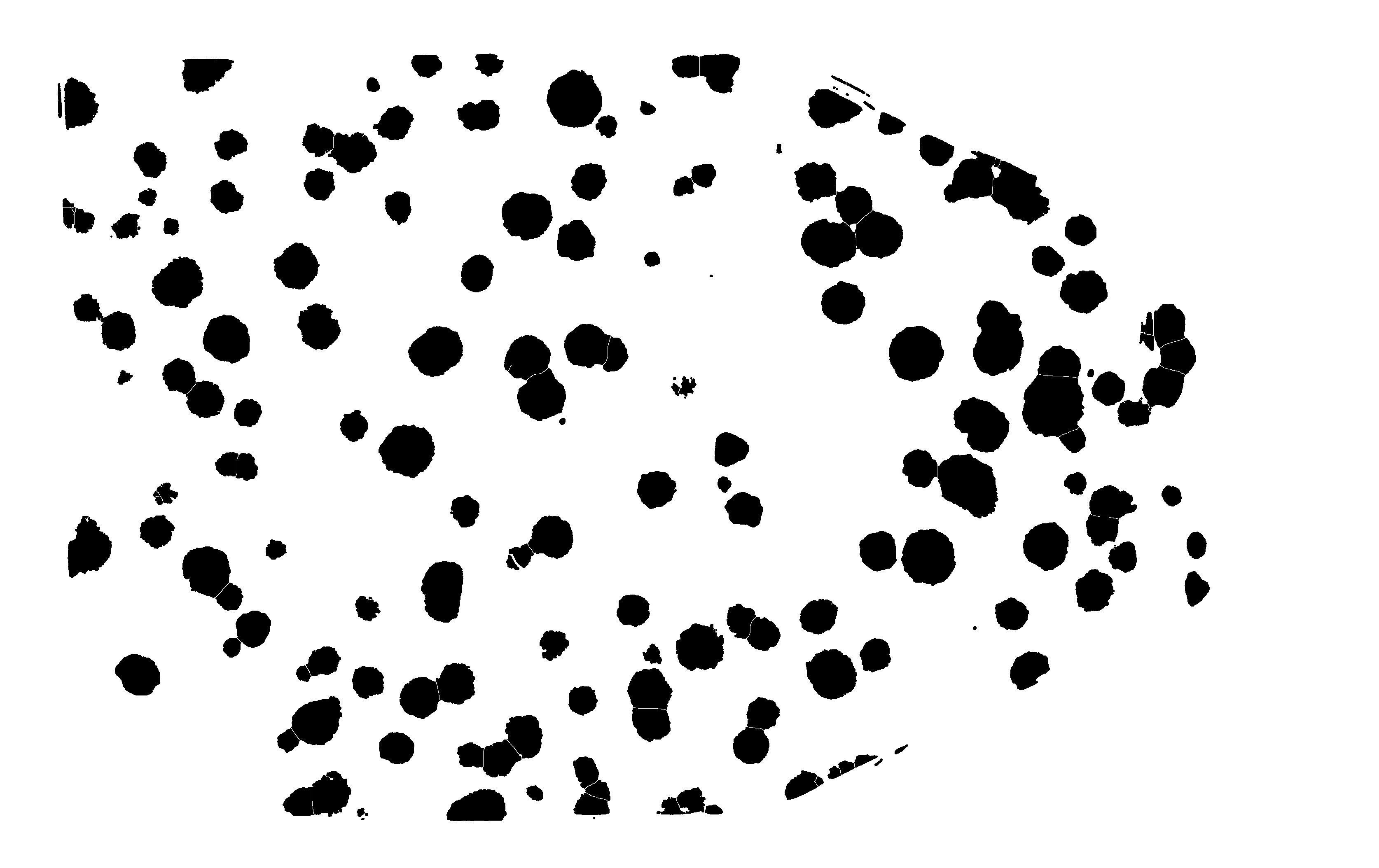

Supplement: S4 Datasets — It also contains a text file where results achieved by automated (CoCoNut, CAI, AutoCellSeg, and OpenCFU) and manual methods are summarized. (ZIP) [file pone.0205823.s005.zip › 180501 HeLa Flask/8 Second counting.jpg]

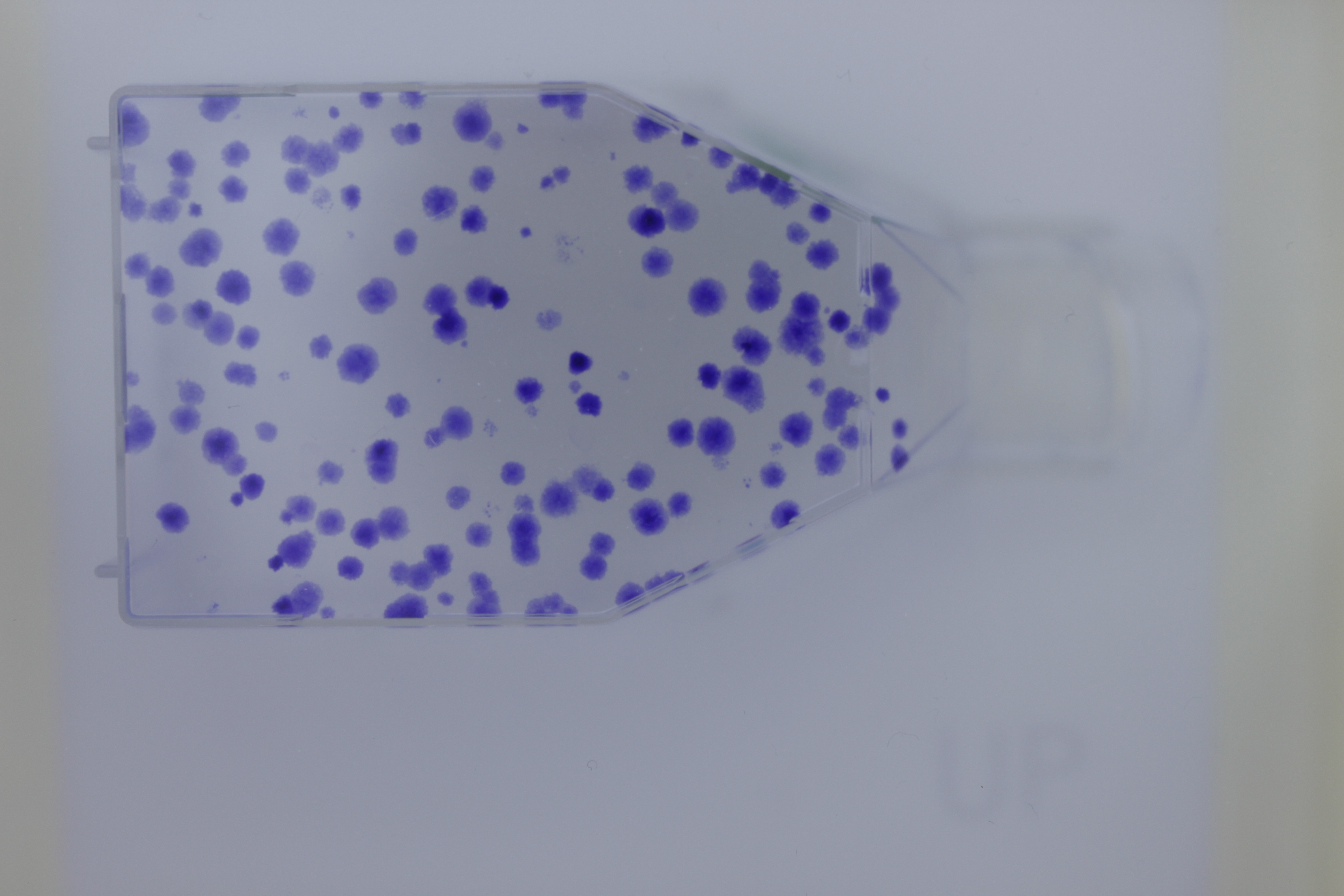

Supplement: S4 Datasets — It also contains a text file where results achieved by automated (CoCoNut, CAI, AutoCellSeg, and OpenCFU) and manual methods are summarized. (ZIP) [file pone.0205823.s005.zip › 180501 HeLa Flask/8.JPG]

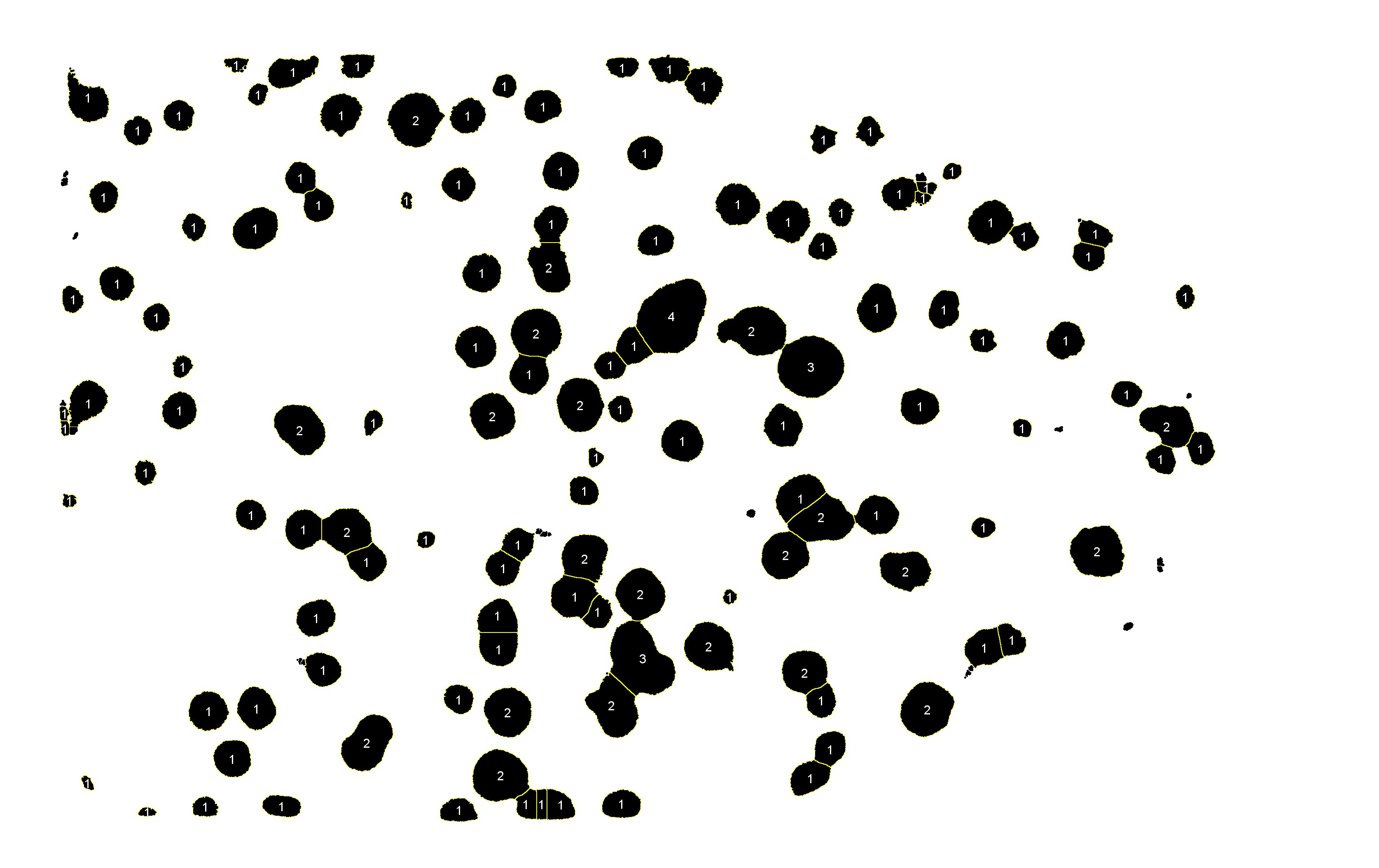

Supplement: S4 Datasets — It also contains a text file where results achieved by automated (CoCoNut, CAI, AutoCellSeg, and OpenCFU) and manual methods are summarized. (ZIP) [file pone.0205823.s005.zip › 180501 HeLa Flask/9 First counting.jpg]

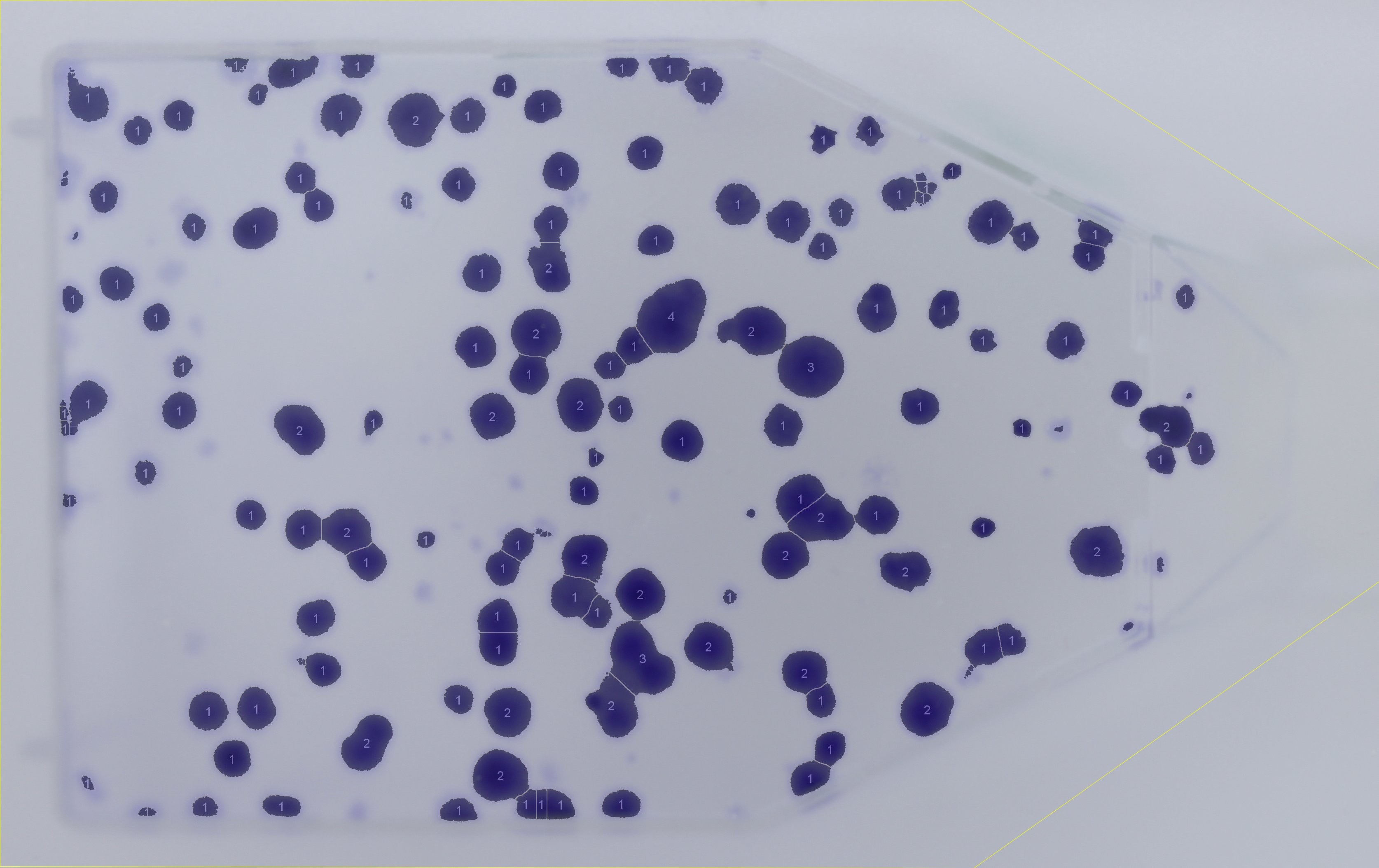

Supplement: S4 Datasets — It also contains a text file where results achieved by automated (CoCoNut, CAI, AutoCellSeg, and OpenCFU) and manual methods are summarized. (ZIP) [file pone.0205823.s005.zip › 180501 HeLa Flask/9 Results.jpg]

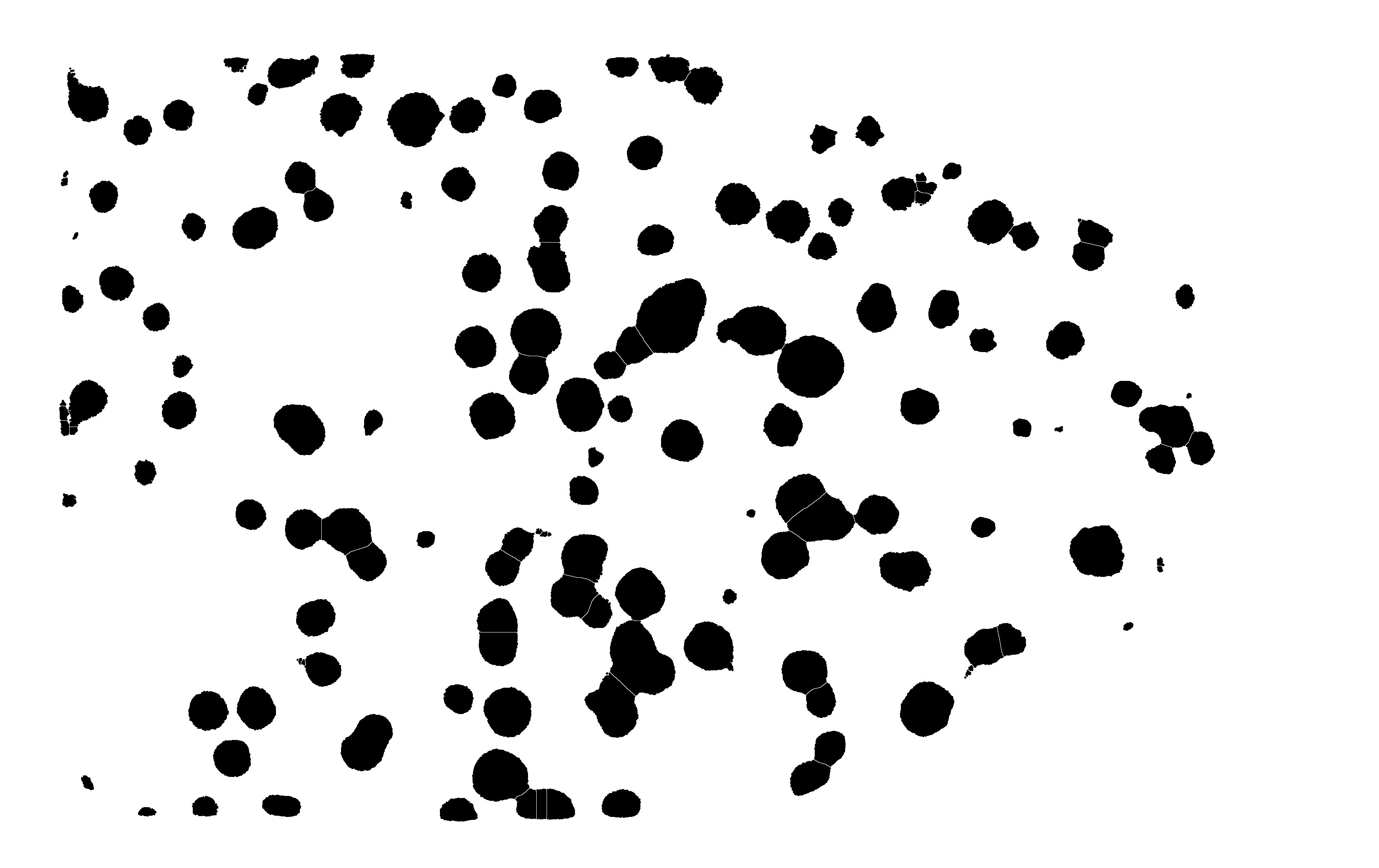

Supplement: S4 Datasets — It also contains a text file where results achieved by automated (CoCoNut, CAI, AutoCellSeg, and OpenCFU) and manual methods are summarized. (ZIP) [file pone.0205823.s005.zip › 180501 HeLa Flask/9 Second counting.jpg]

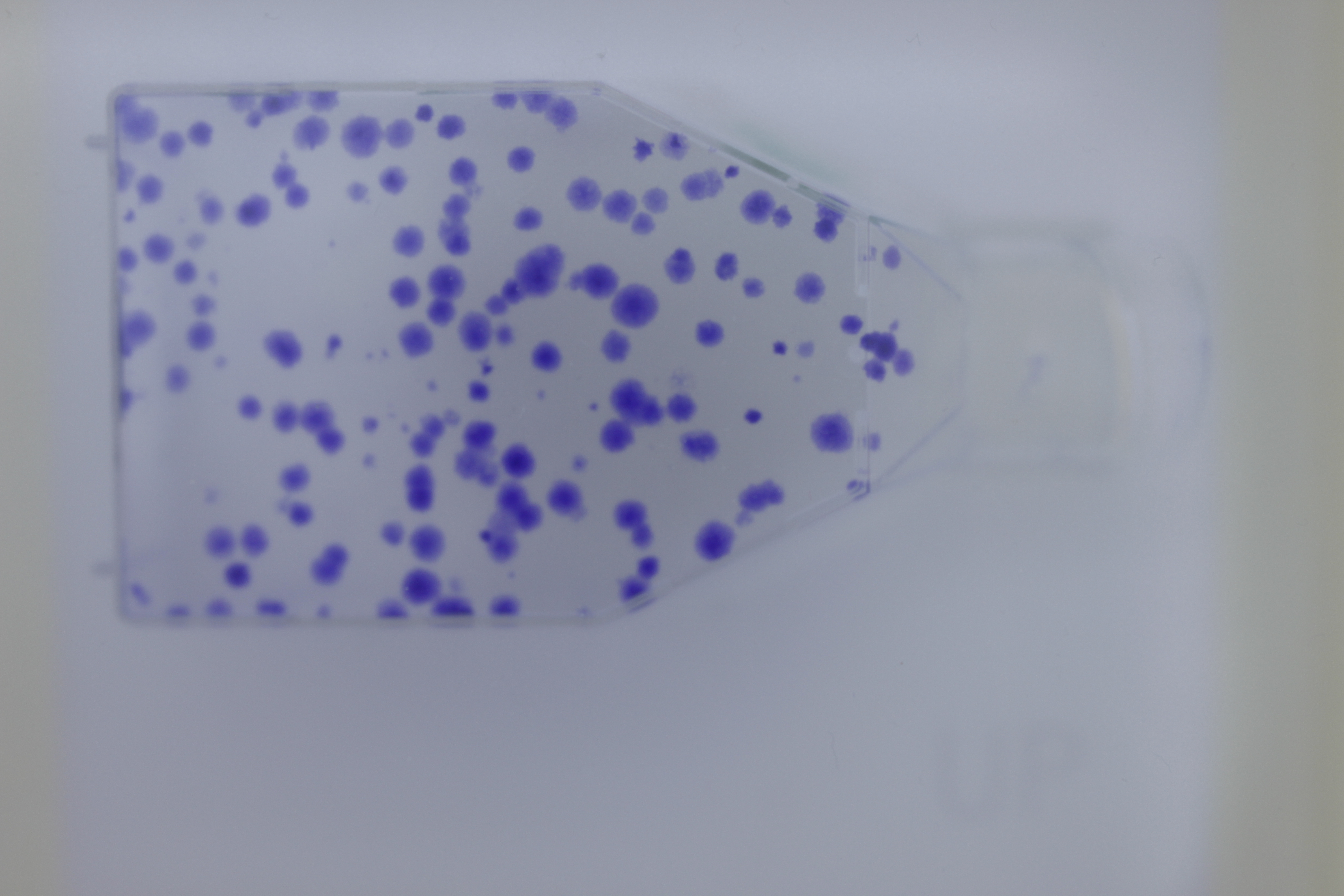

Supplement: S4 Datasets — It also contains a text file where results achieved by automated (CoCoNut, CAI, AutoCellSeg, and OpenCFU) and manual methods are summarized. (ZIP) [file pone.0205823.s005.zip › 180501 HeLa Flask/9.JPG]

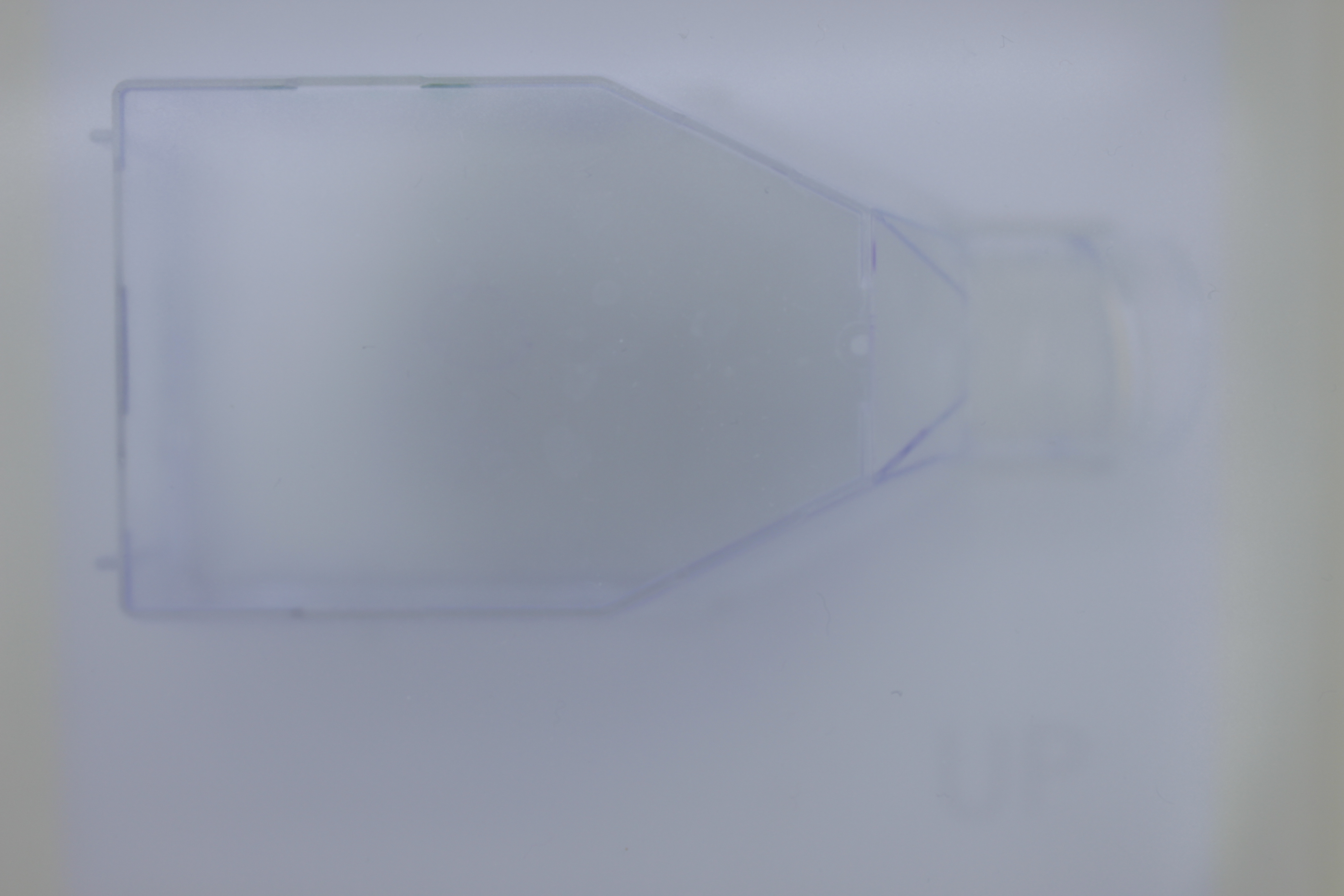

Supplement: S4 Datasets — It also contains a text file where results achieved by automated (CoCoNut, CAI, AutoCellSeg, and OpenCFU) and manual methods are summarized. (ZIP) [file pone.0205823.s005.zip › 180501 HeLa Flask/background.JPG]

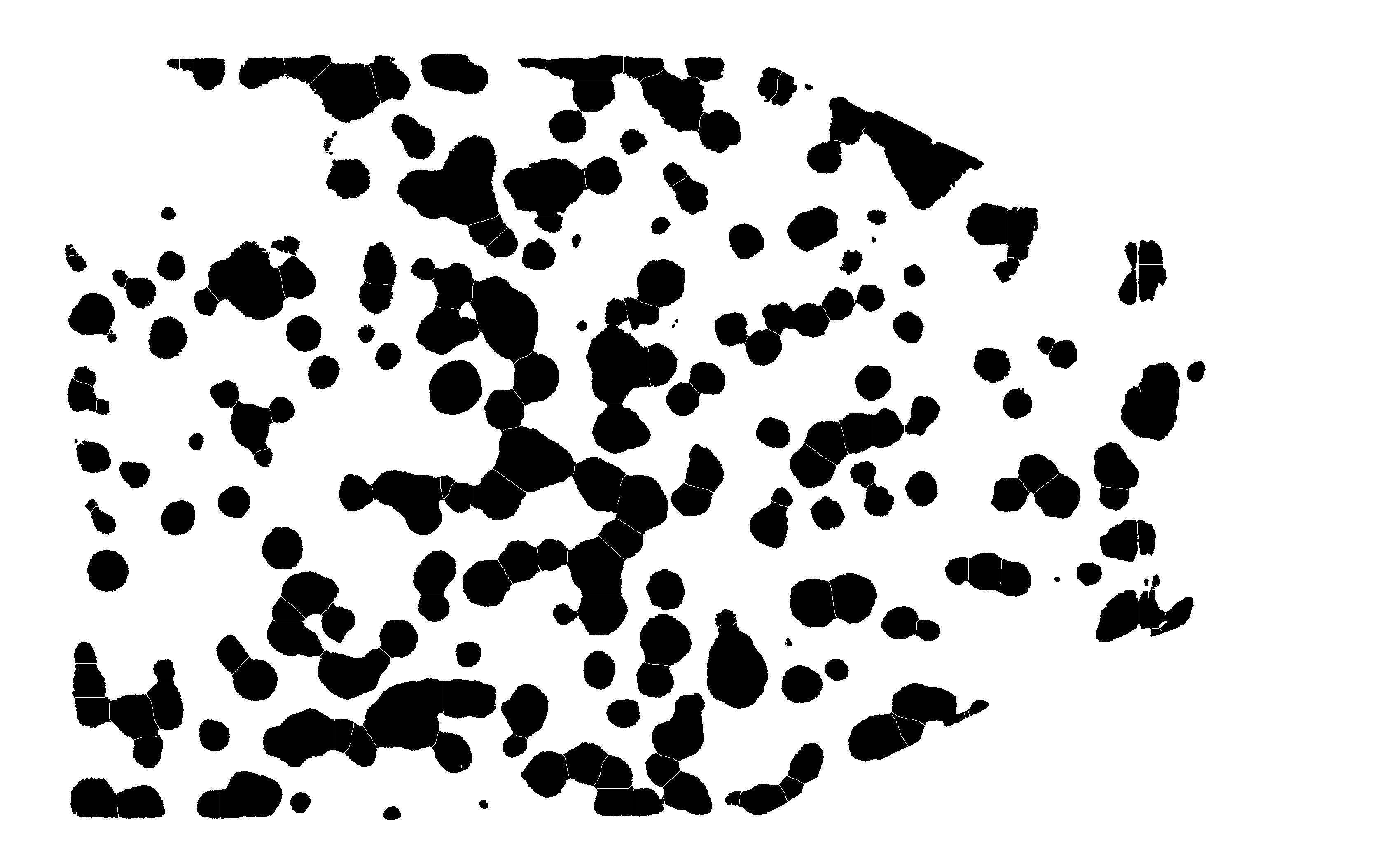

Supplement: S4 Datasets — It also contains a text file where results achieved by automated (CoCoNut, CAI, AutoCellSeg, and OpenCFU) and manual methods are summarized. (ZIP) [file pone.0205823.s005.zip › 180501 HeLa Flask/binary10.jpg]

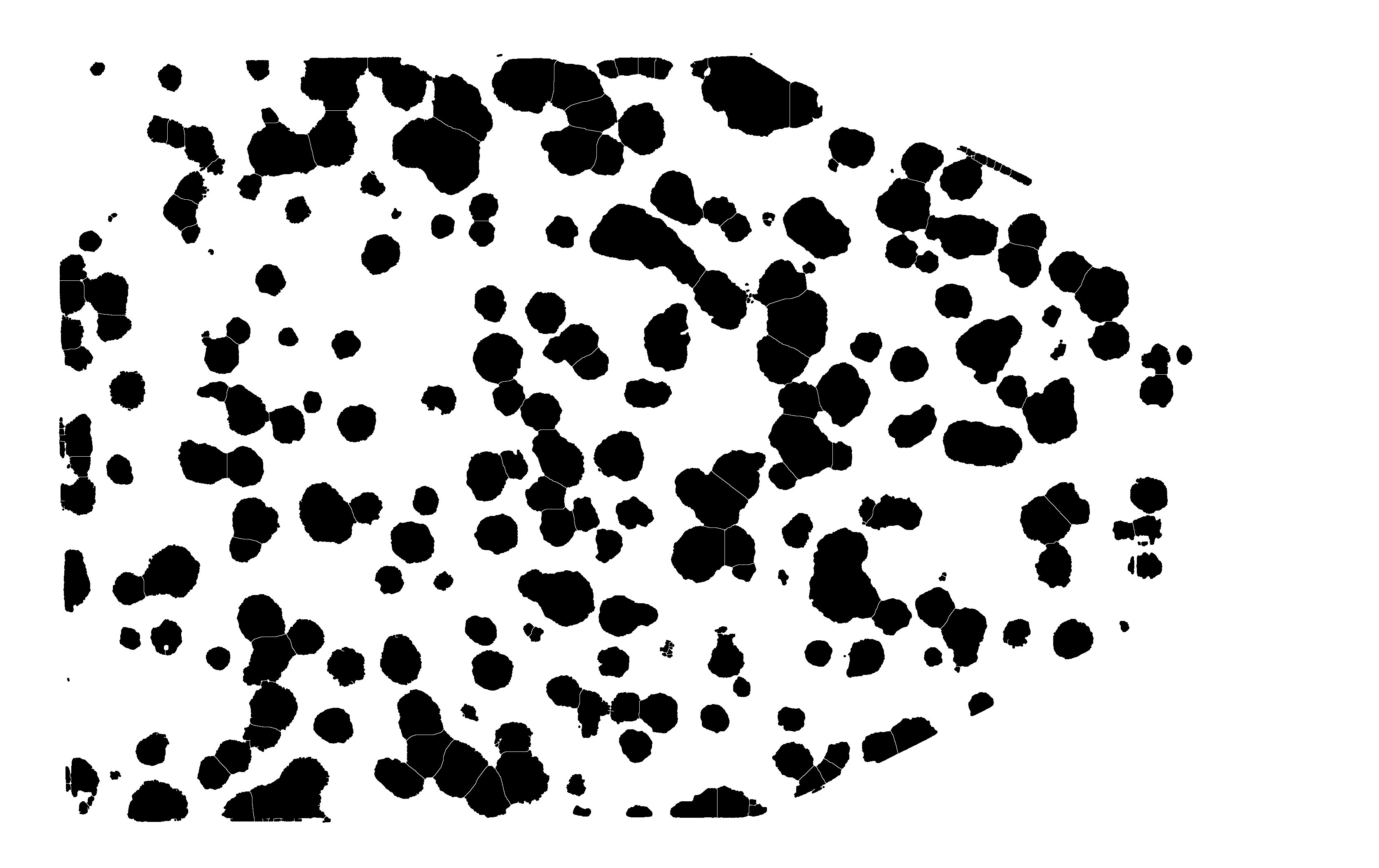

Supplement: S4 Datasets — It also contains a text file where results achieved by automated (CoCoNut, CAI, AutoCellSeg, and OpenCFU) and manual methods are summarized. (ZIP) [file pone.0205823.s005.zip › 180501 HeLa Flask/binary11.jpg]

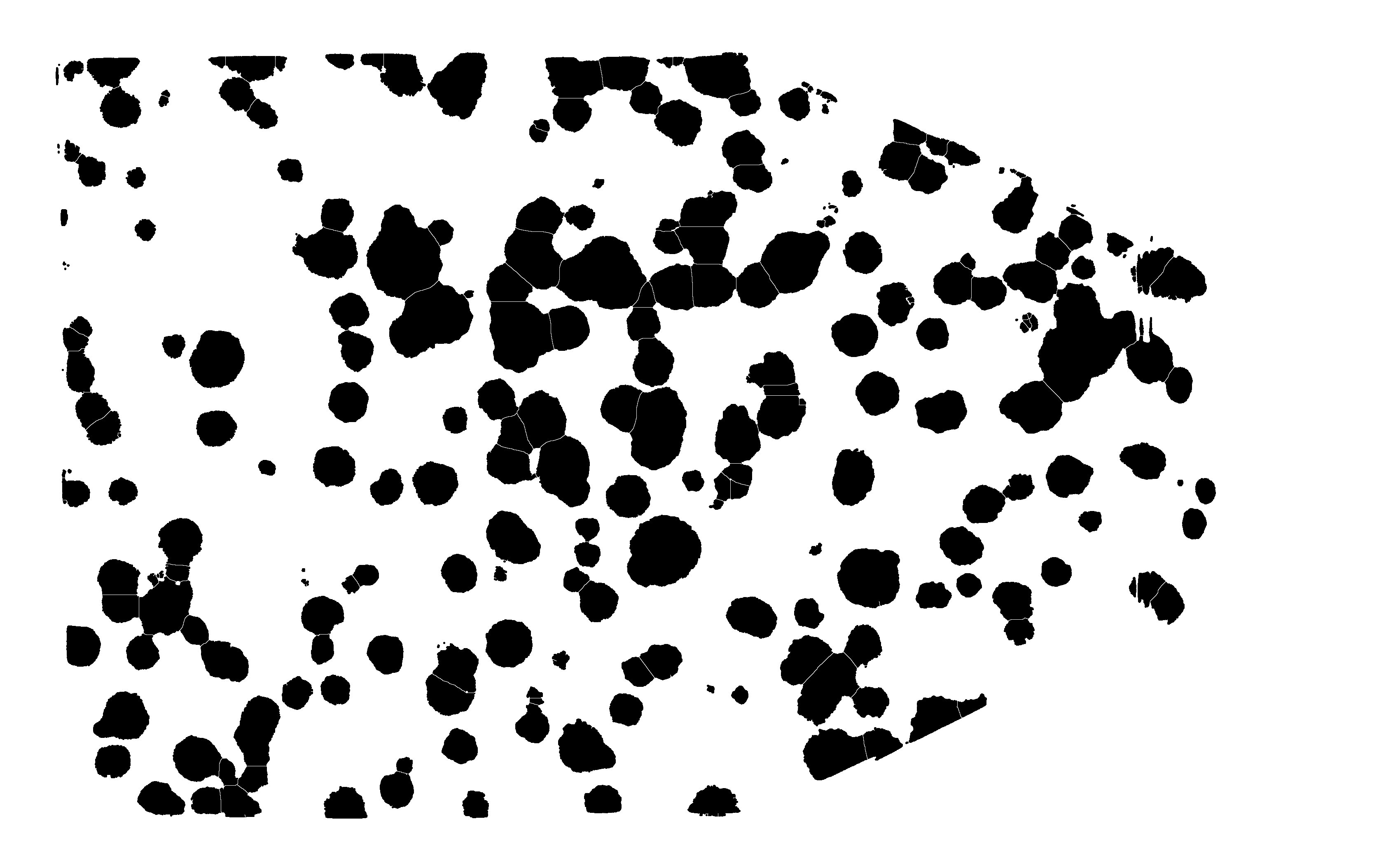

Supplement: S4 Datasets — It also contains a text file where results achieved by automated (CoCoNut, CAI, AutoCellSeg, and OpenCFU) and manual methods are summarized. (ZIP) [file pone.0205823.s005.zip › 180501 HeLa Flask/binary12.jpg]

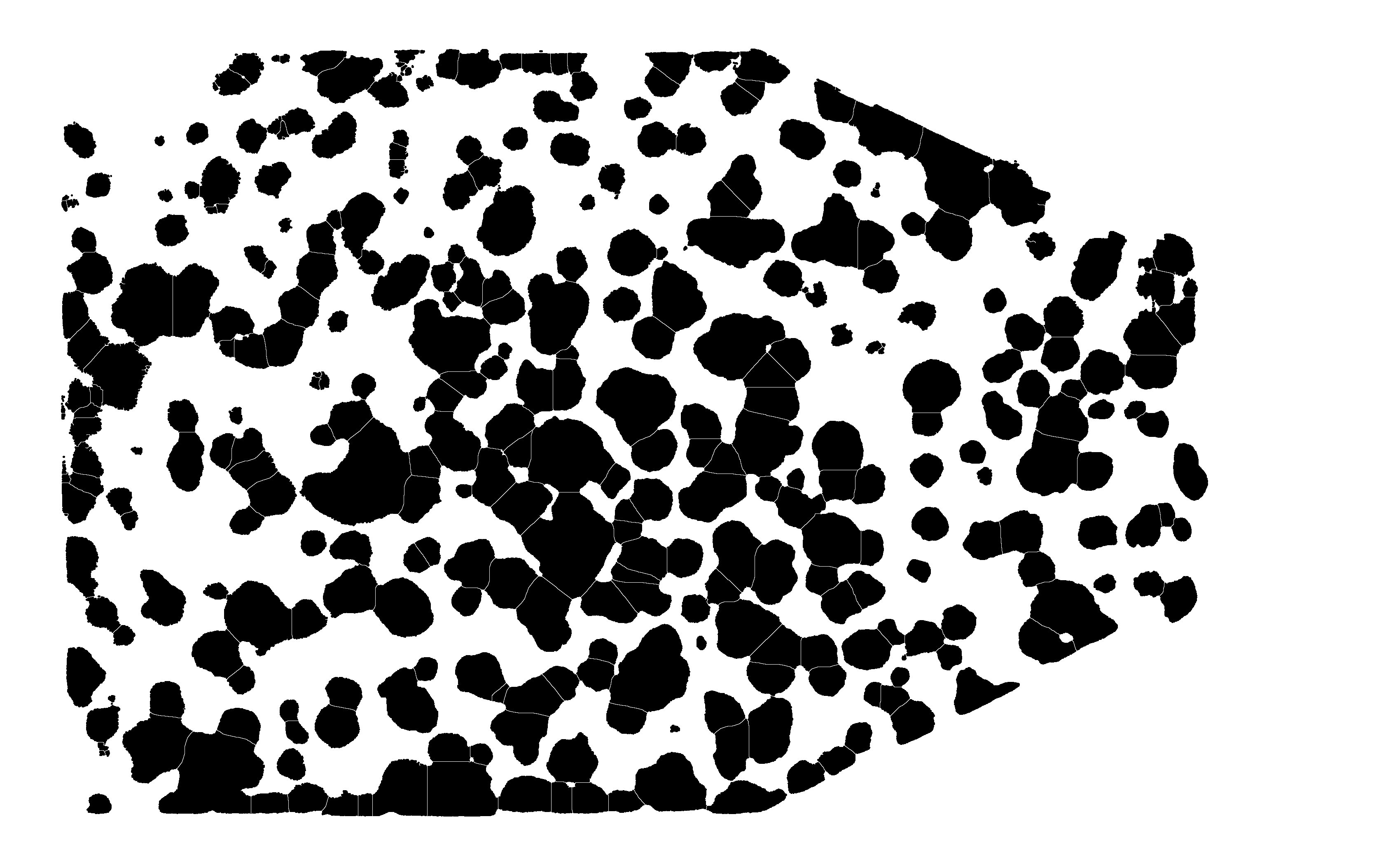

Supplement: S4 Datasets — It also contains a text file where results achieved by automated (CoCoNut, CAI, AutoCellSeg, and OpenCFU) and manual methods are summarized. (ZIP) [file pone.0205823.s005.zip › 180501 HeLa Flask/binary13.jpg]

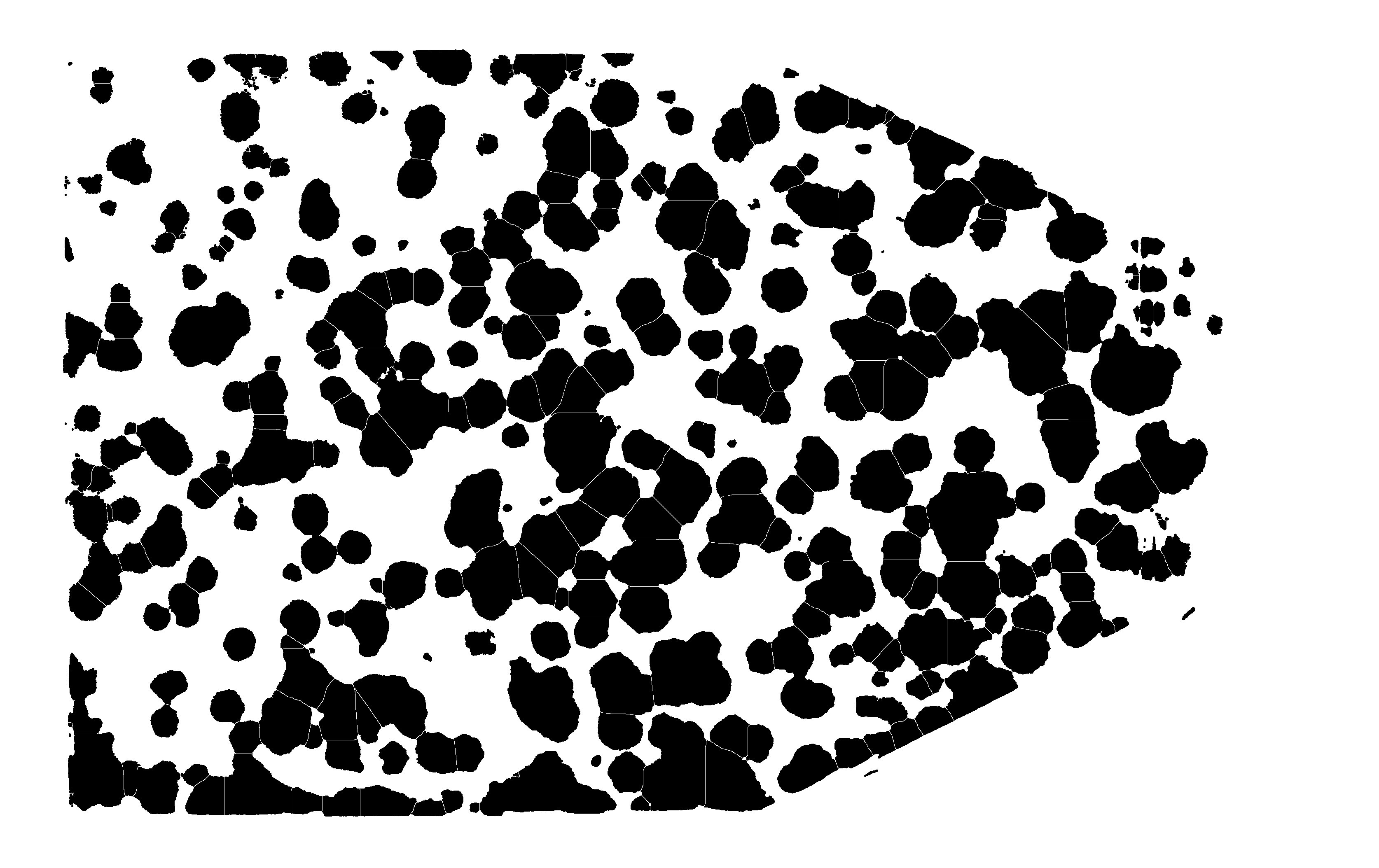

Supplement: S4 Datasets — It also contains a text file where results achieved by automated (CoCoNut, CAI, AutoCellSeg, and OpenCFU) and manual methods are summarized. (ZIP) [file pone.0205823.s005.zip › 180501 HeLa Flask/binary14.jpg]

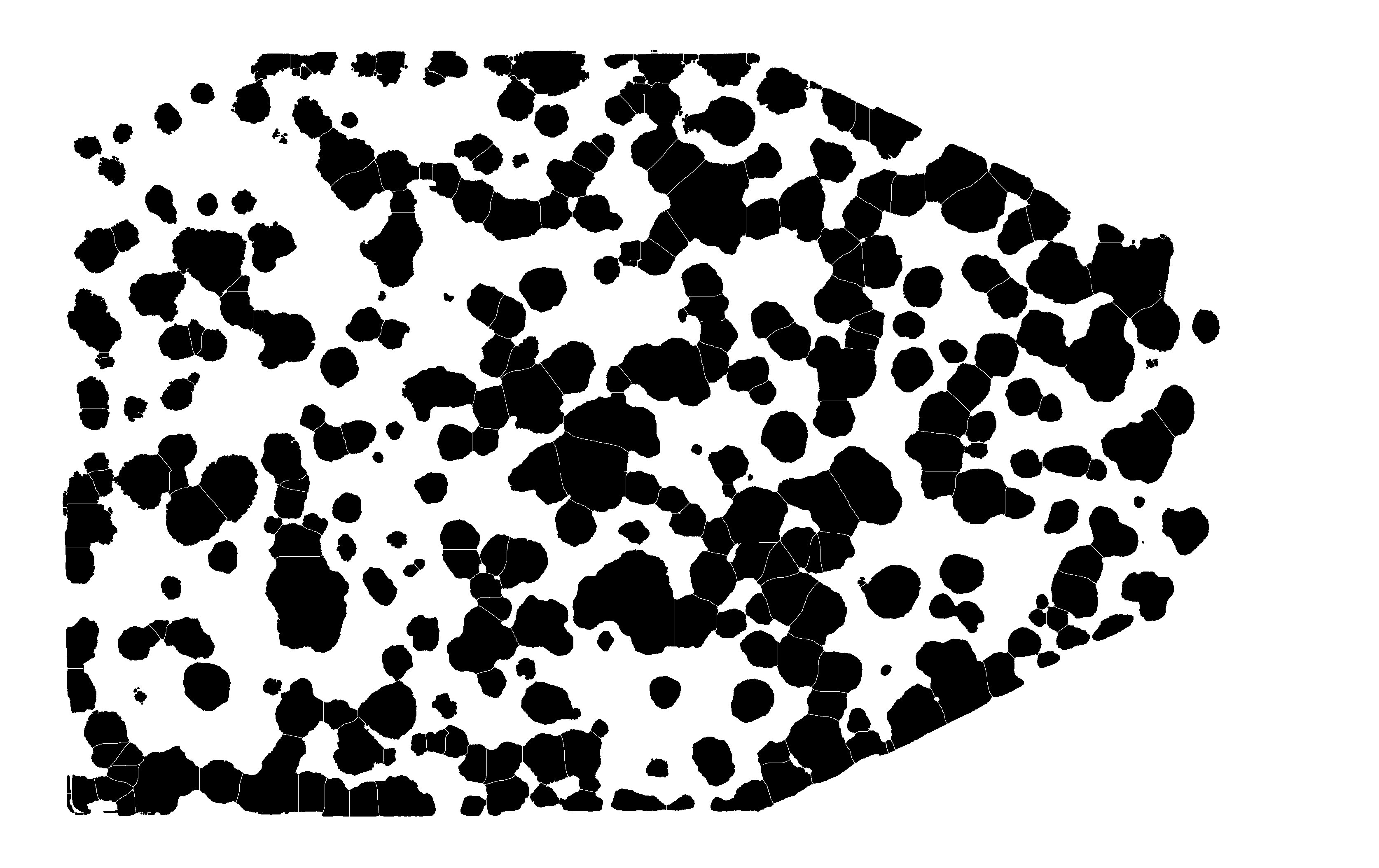

Supplement: S4 Datasets — It also contains a text file where results achieved by automated (CoCoNut, CAI, AutoCellSeg, and OpenCFU) and manual methods are summarized. (ZIP) [file pone.0205823.s005.zip › 180501 HeLa Flask/binary15.jpg]

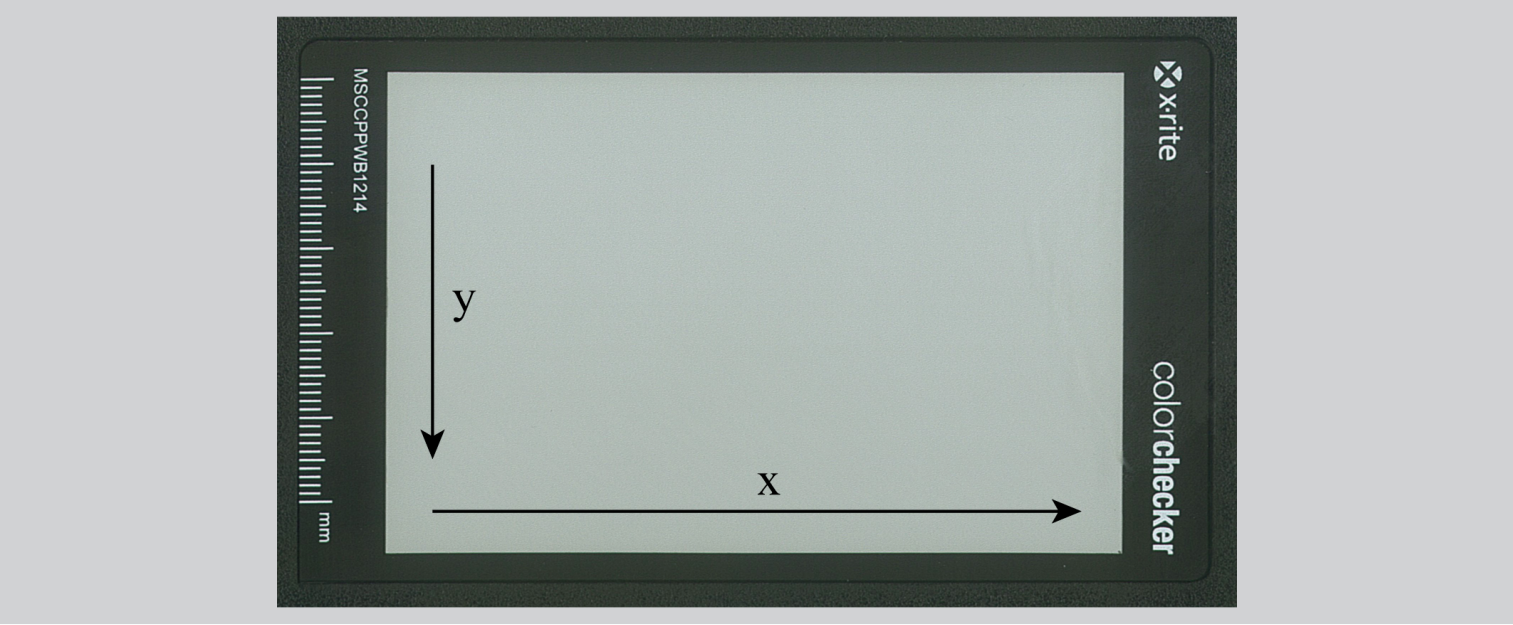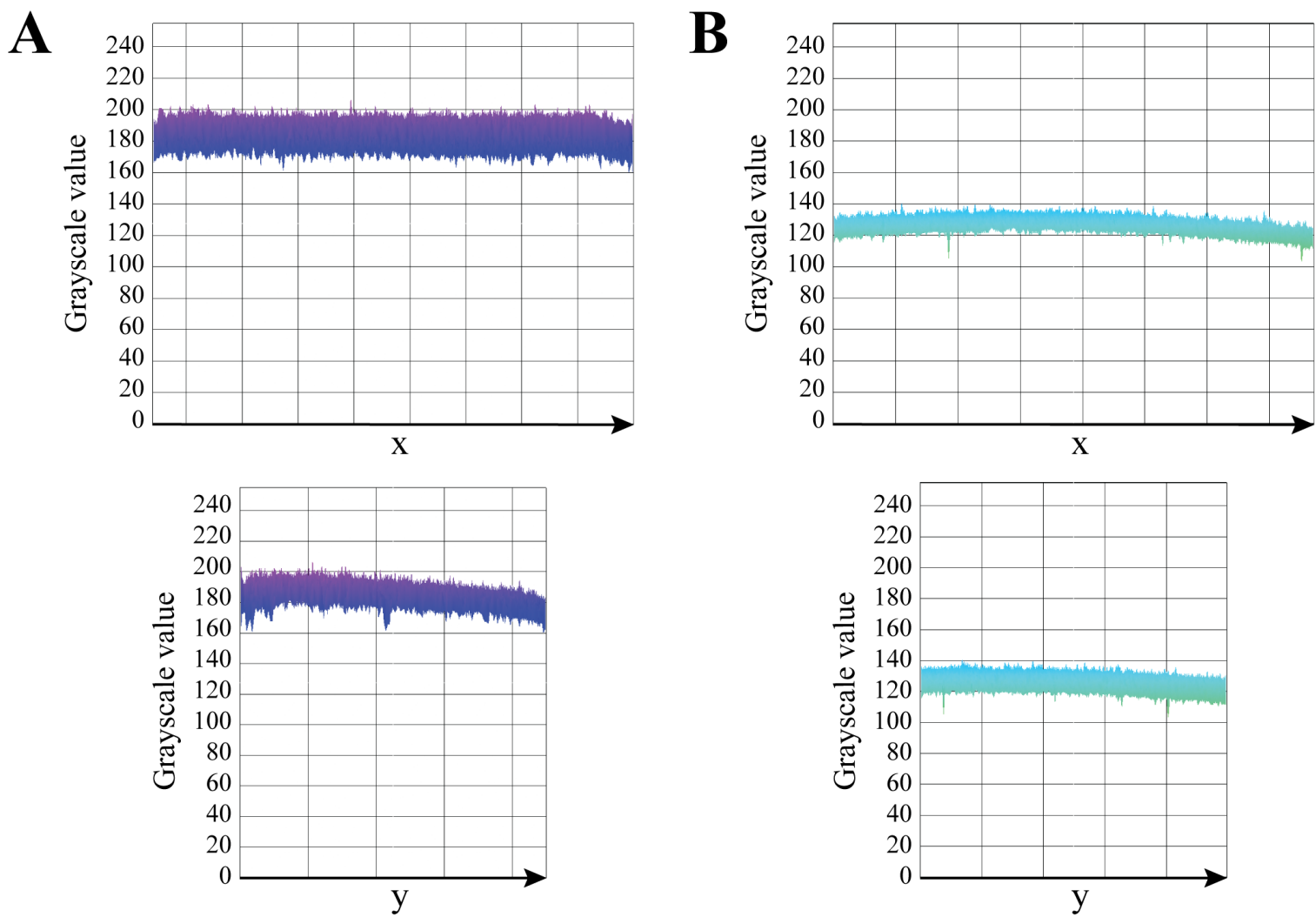

Fig A

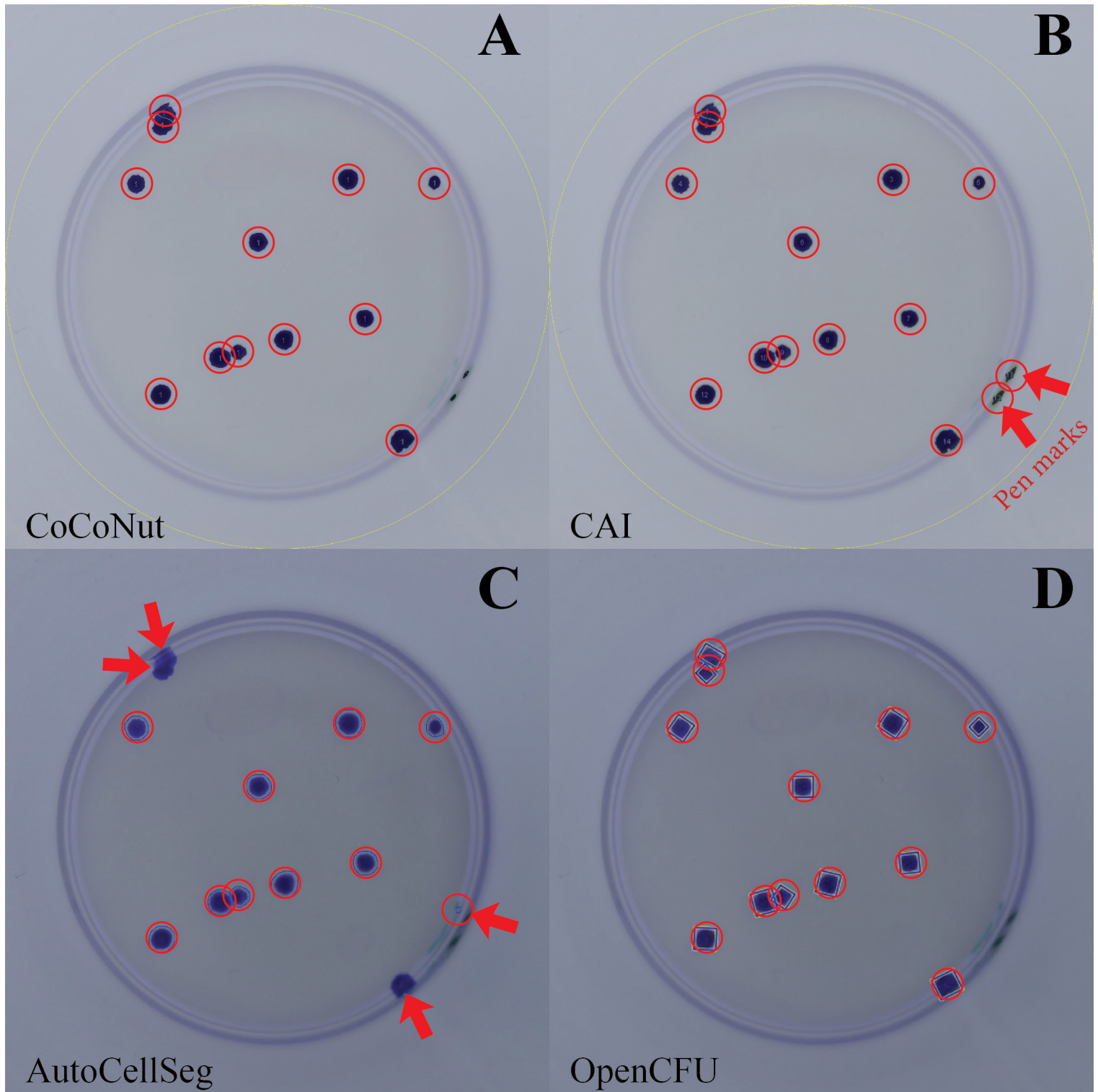

Fig B

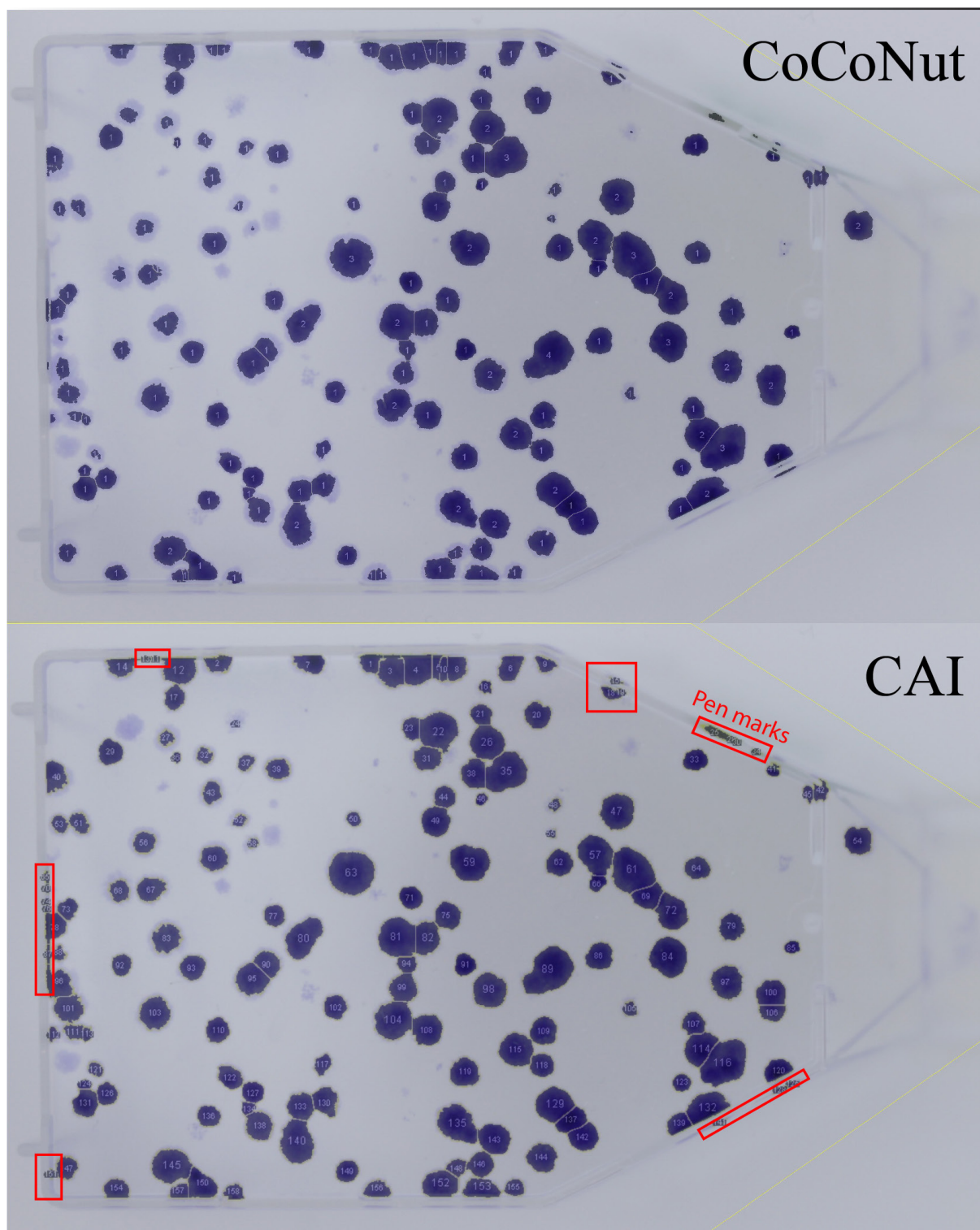

Fig C

Supplement: S1 Fig — The supporting information file contains three figures, labeled A, B, and C. Fig A. In the figure, we compare our own office flatbed scanner (A, on the left, WorkCentre 7775, Xerox, Ballerup, Denmark) with the CoCoNut light-box (B, on the right), which was coupled to the Canon camera described in the paper. We have tested uniform lighting using the X-Rite ColorChecker White Balance target (on the top) as described in the article. Fig B. 171214 V79 Dish/1.jpg is used as an example to show how scoring decisions change among different tools. Red circles were added in post-production in correspondence to the counted cell clones in order to simplify the comparison. The yellow circle in A shows the region of interest selected in CoCoNut for the analysis. The same region was used in B and D, while AutoCellSeg does not provide such feature but it always analyzes the whole picture. Fig C. This example (180501 HeLa Flask/7.jpg) illustrates how false positives by CAI (bottom) can counterbalance underestimated colony counts. In CoCoNut (top), numbers indicate the number of colonies contained within each scoring region, while they have just an ordering function in CAI, where regions can contain only 1 colony (ZIP) [file pone.0205823.s006.zip › S1 Figures.pdf]

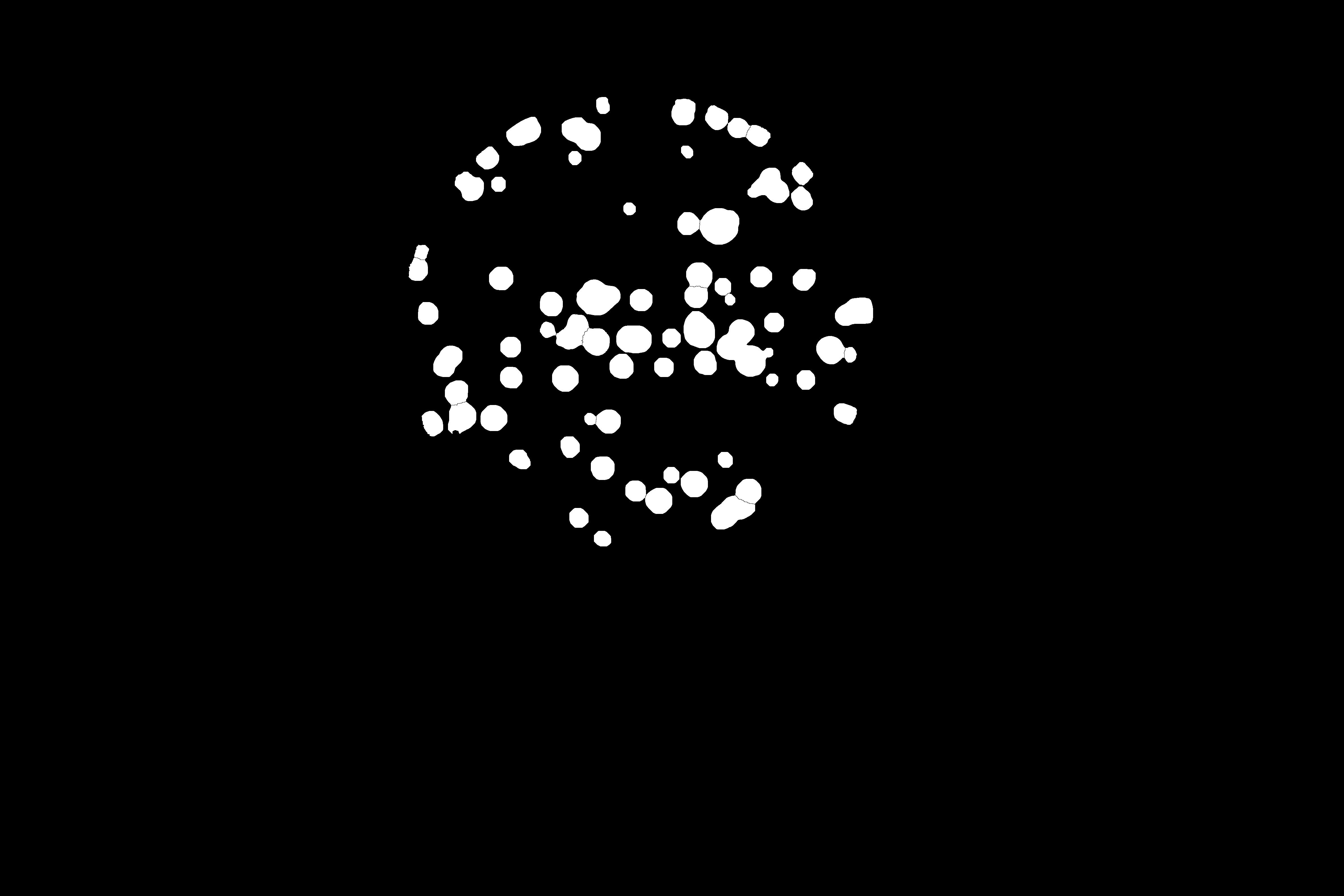

Supplement: S1 Comparison to others — (ZIP) [file pone.0205823.s007.zip › S1 Comparison to others/AutoCellSeg/171214 V79 Dish/10_mask.jpg]

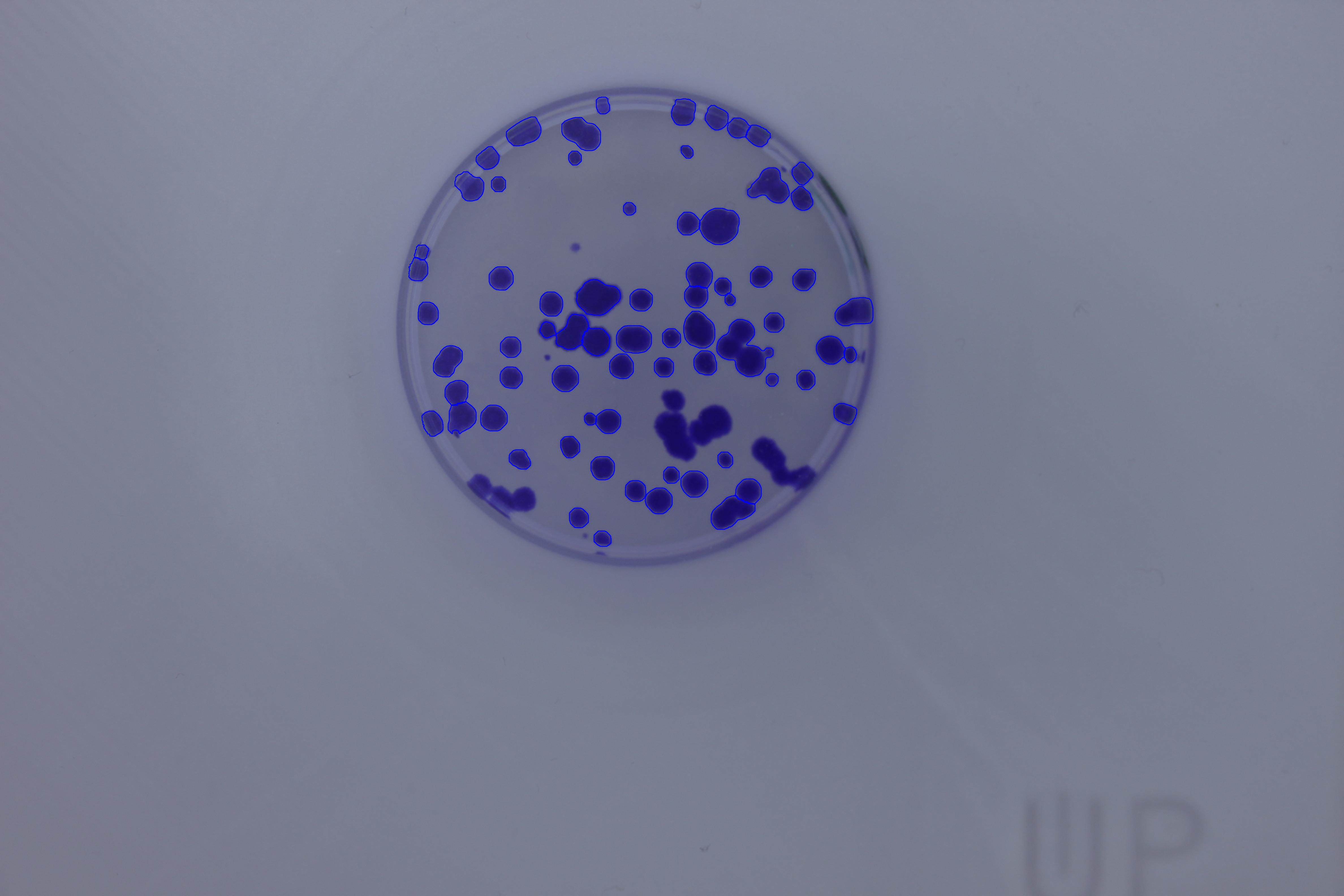

Supplement: S1 Comparison to others — (ZIP) [file pone.0205823.s007.zip › S1 Comparison to others/AutoCellSeg/171214 V79 Dish/10_seg.jpg]

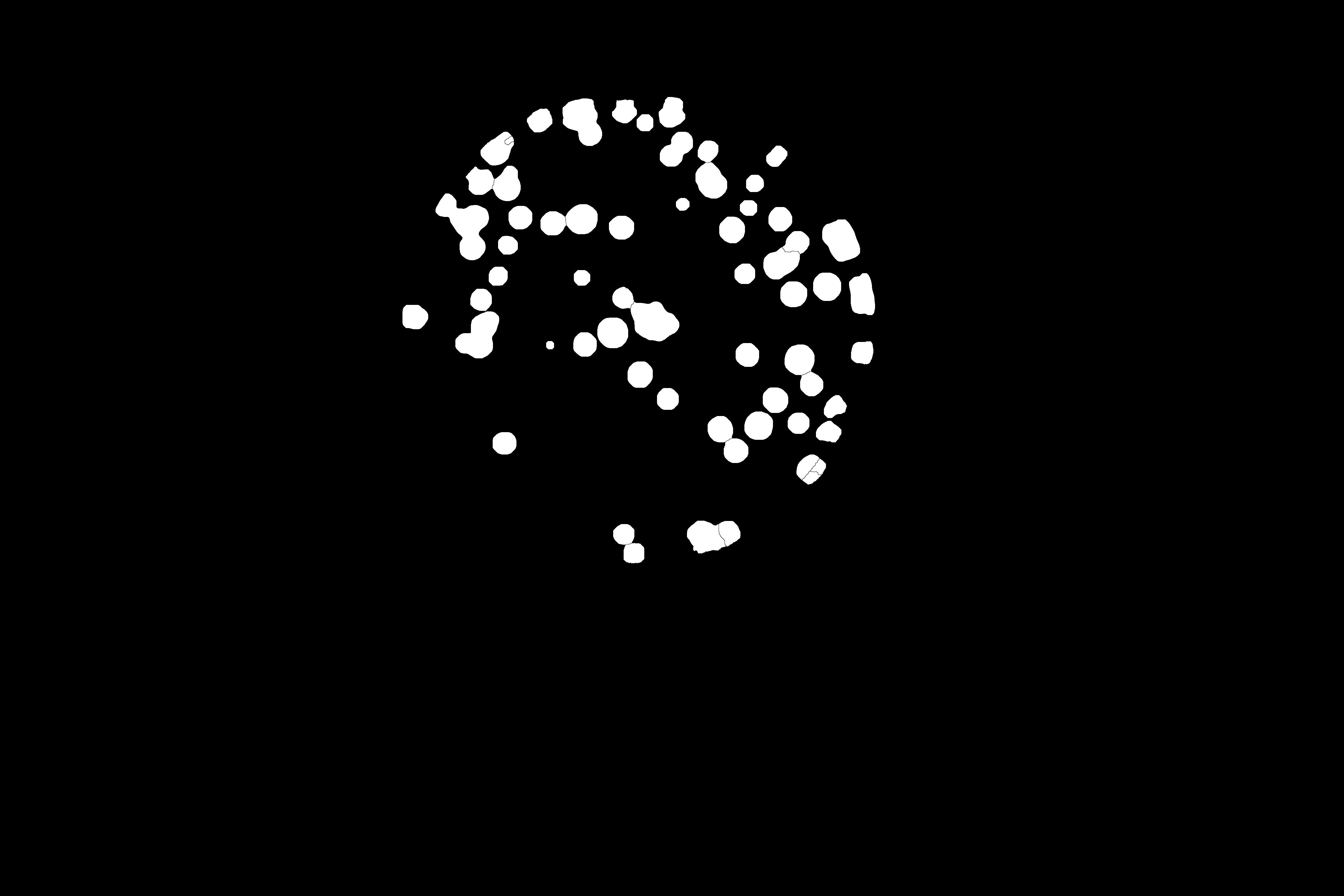

Supplement: S1 Comparison to others — (ZIP) [file pone.0205823.s007.zip › S1 Comparison to others/AutoCellSeg/171214 V79 Dish/11_mask.jpg]

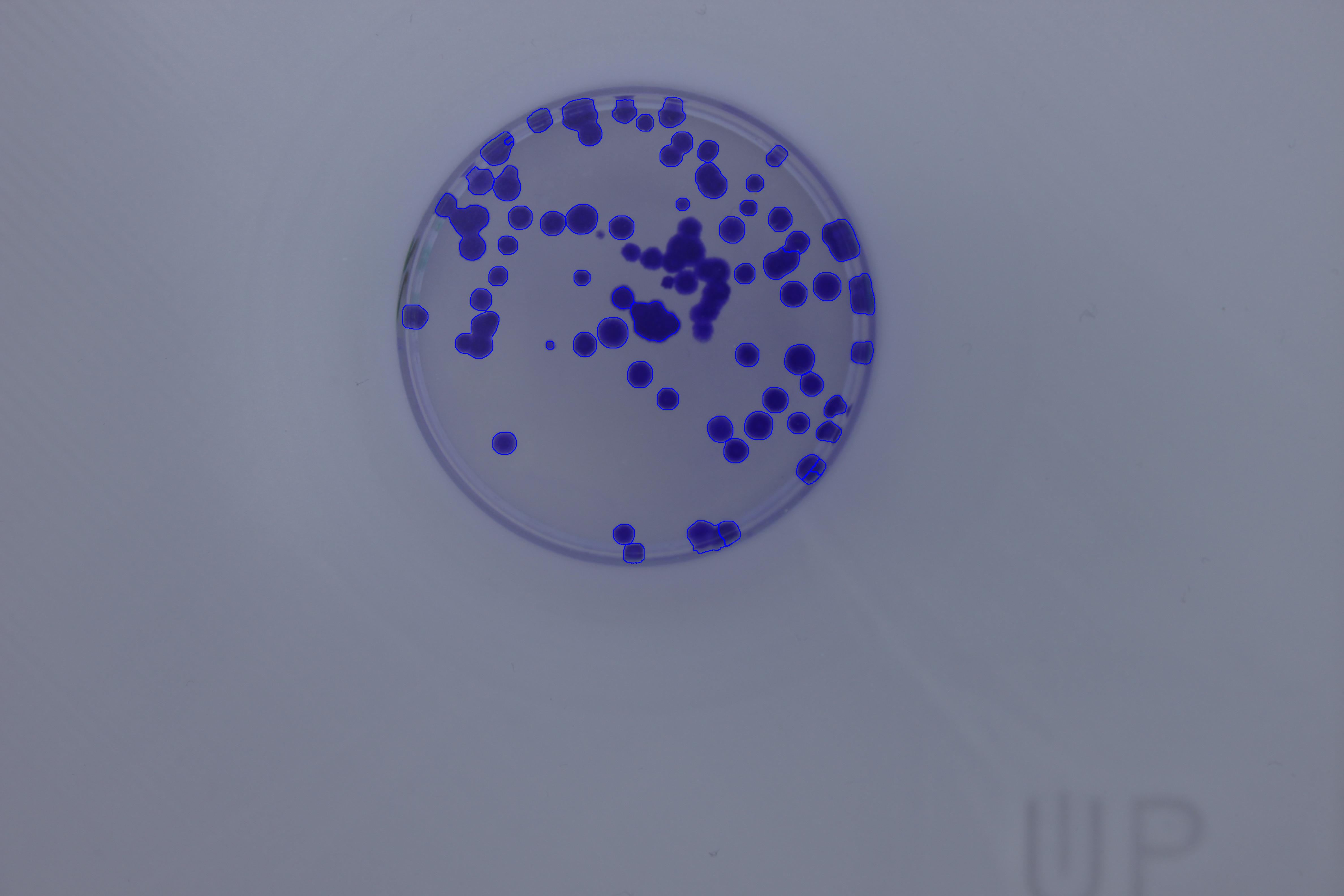

Supplement: S1 Comparison to others — (ZIP) [file pone.0205823.s007.zip › S1 Comparison to others/AutoCellSeg/171214 V79 Dish/11_seg.jpg]

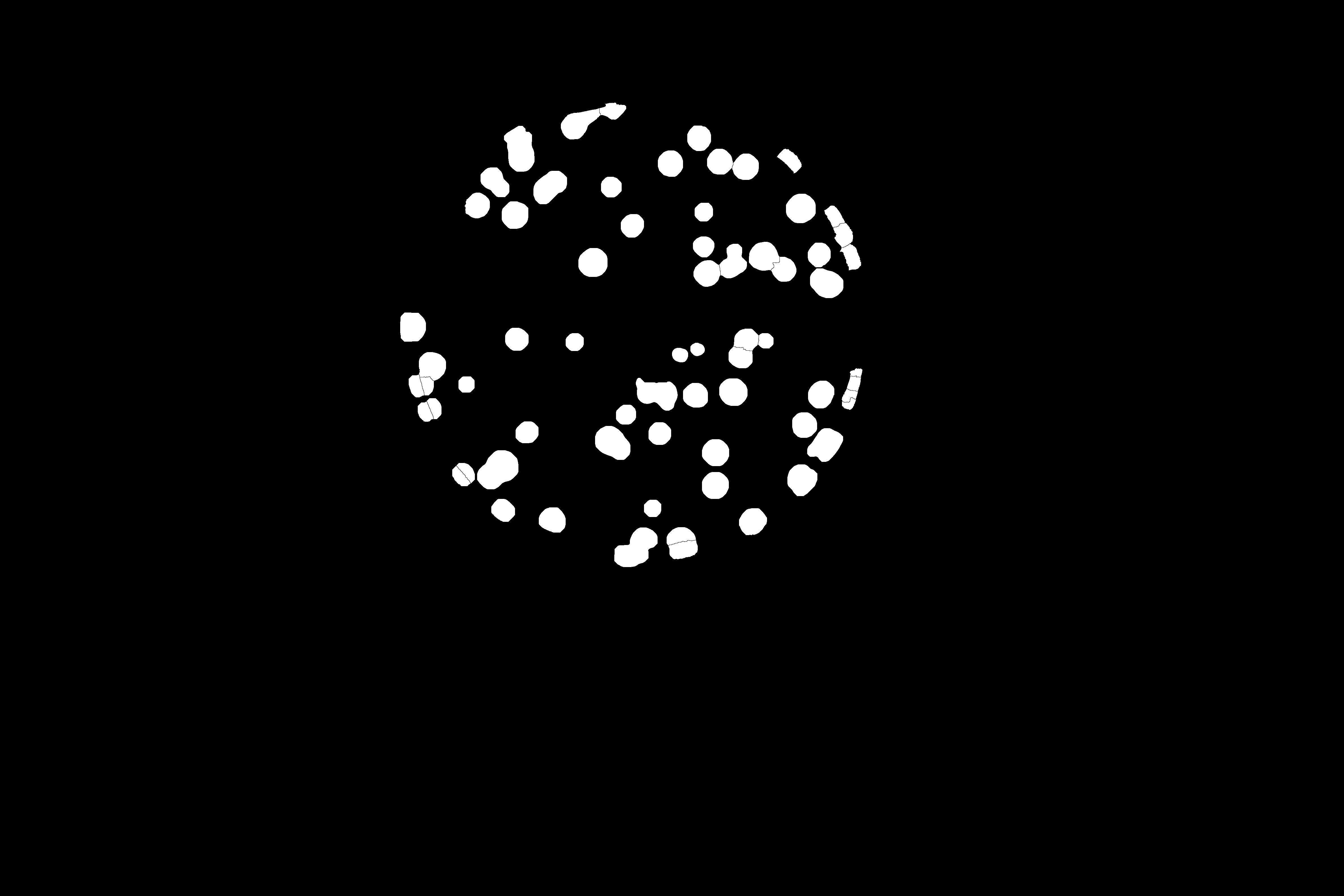

Supplement: S1 Comparison to others — (ZIP) [file pone.0205823.s007.zip › S1 Comparison to others/AutoCellSeg/171214 V79 Dish/12_mask.jpg]

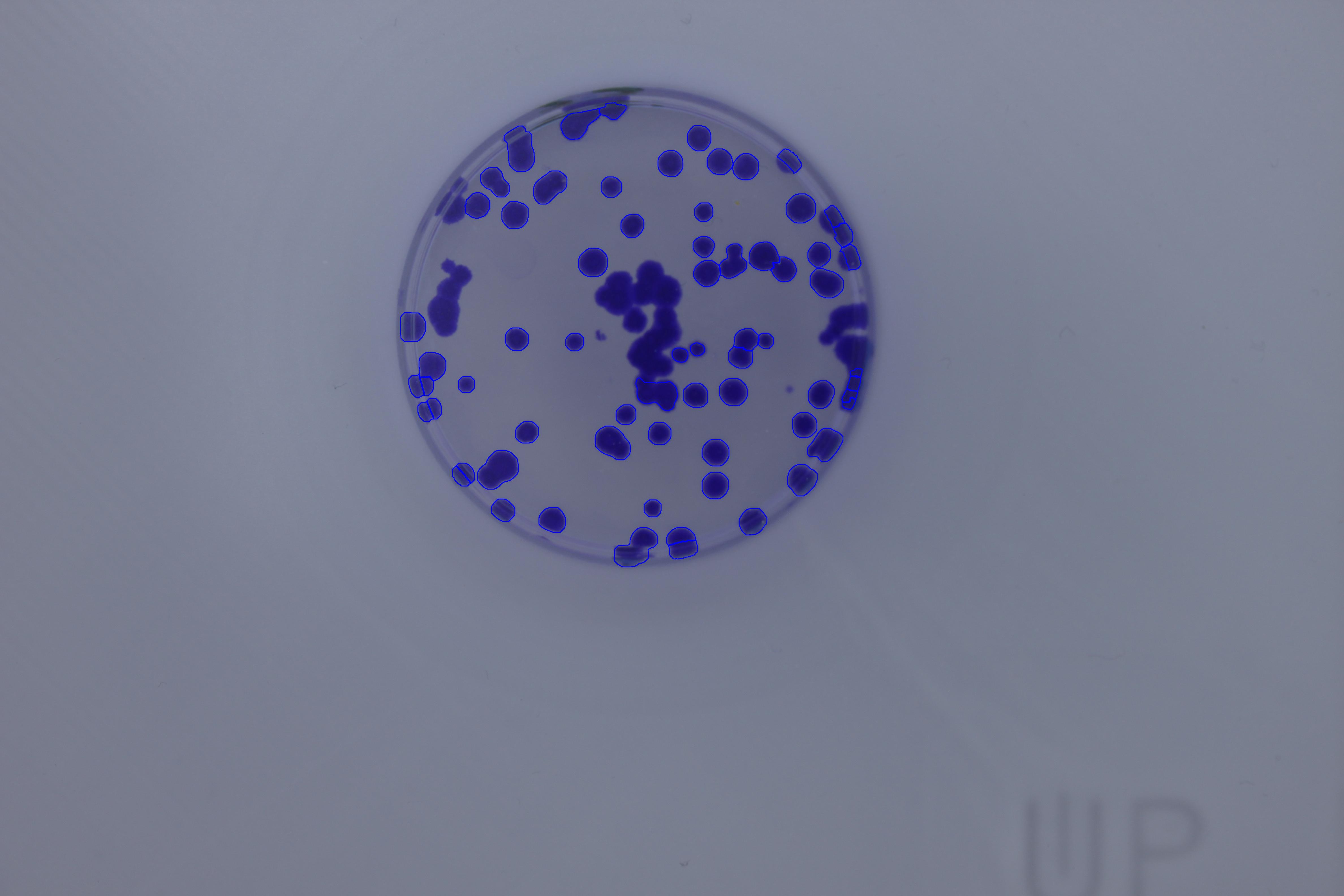

Supplement: S1 Comparison to others — (ZIP) [file pone.0205823.s007.zip › S1 Comparison to others/AutoCellSeg/171214 V79 Dish/12_seg.jpg]

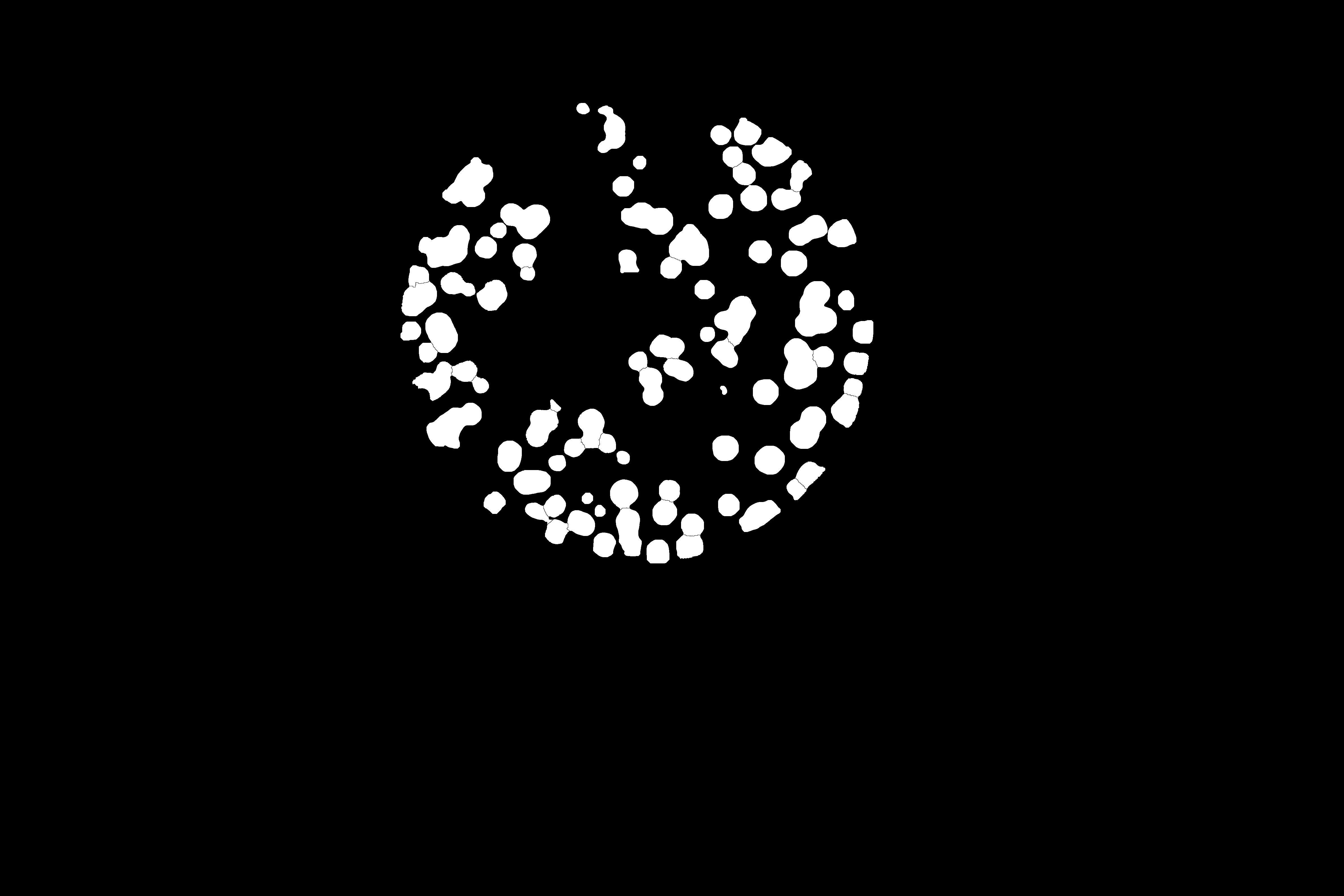

Supplement: S1 Comparison to others — (ZIP) [file pone.0205823.s007.zip › S1 Comparison to others/AutoCellSeg/171214 V79 Dish/13_mask.jpg]

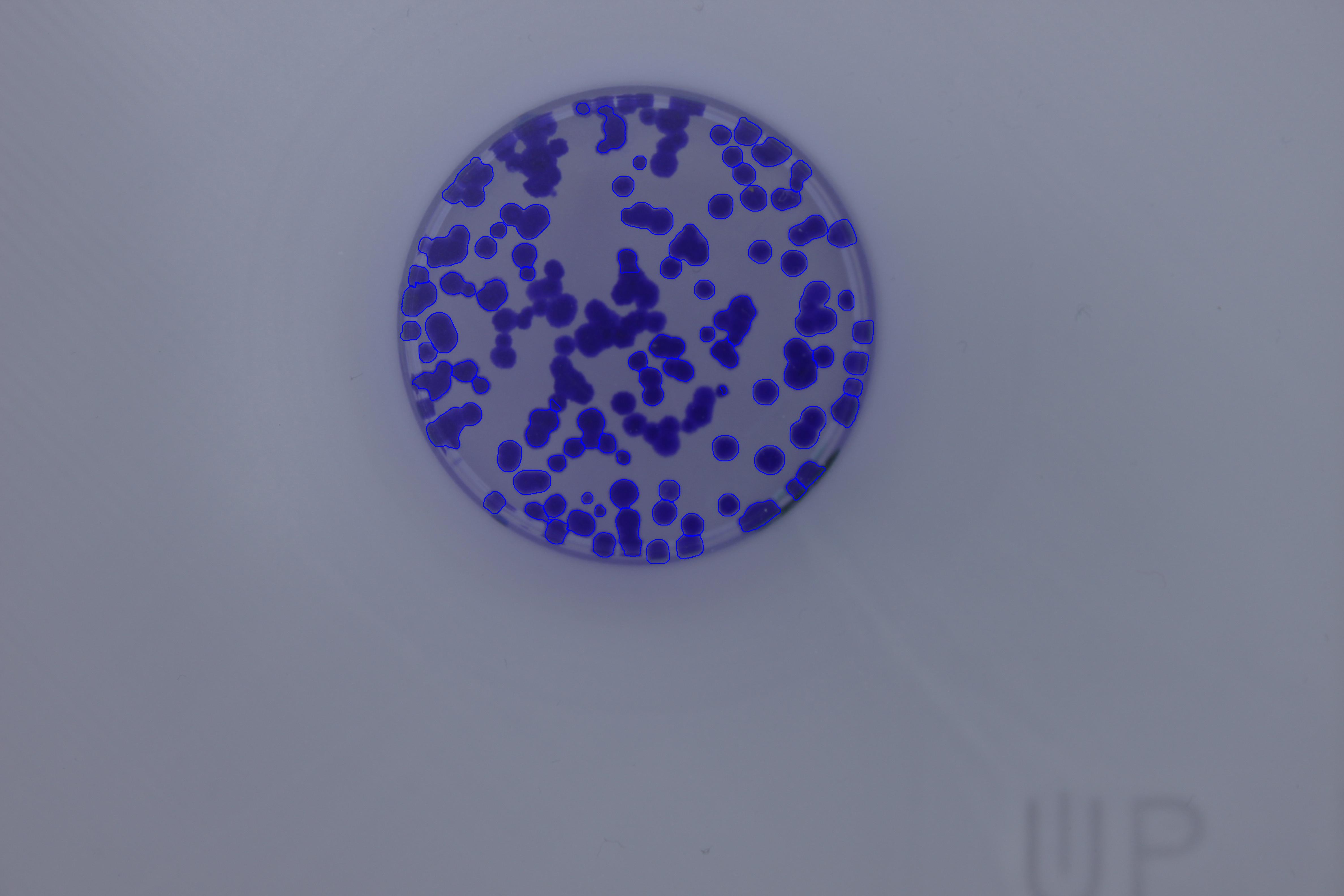

Supplement: S1 Comparison to others — (ZIP) [file pone.0205823.s007.zip › S1 Comparison to others/AutoCellSeg/171214 V79 Dish/13_seg.jpg]

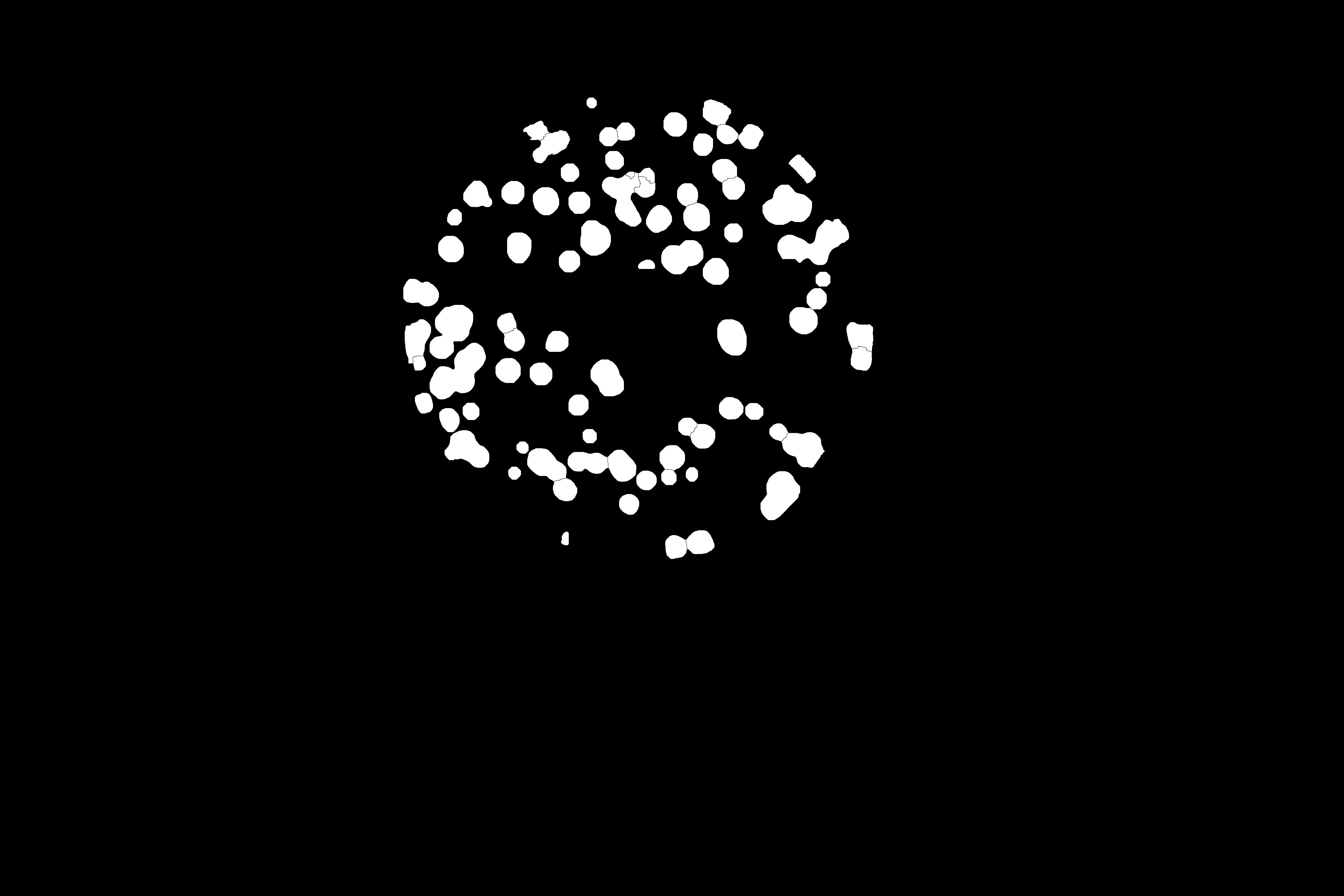

Supplement: S1 Comparison to others — (ZIP) [file pone.0205823.s007.zip › S1 Comparison to others/AutoCellSeg/171214 V79 Dish/14_mask.jpg]

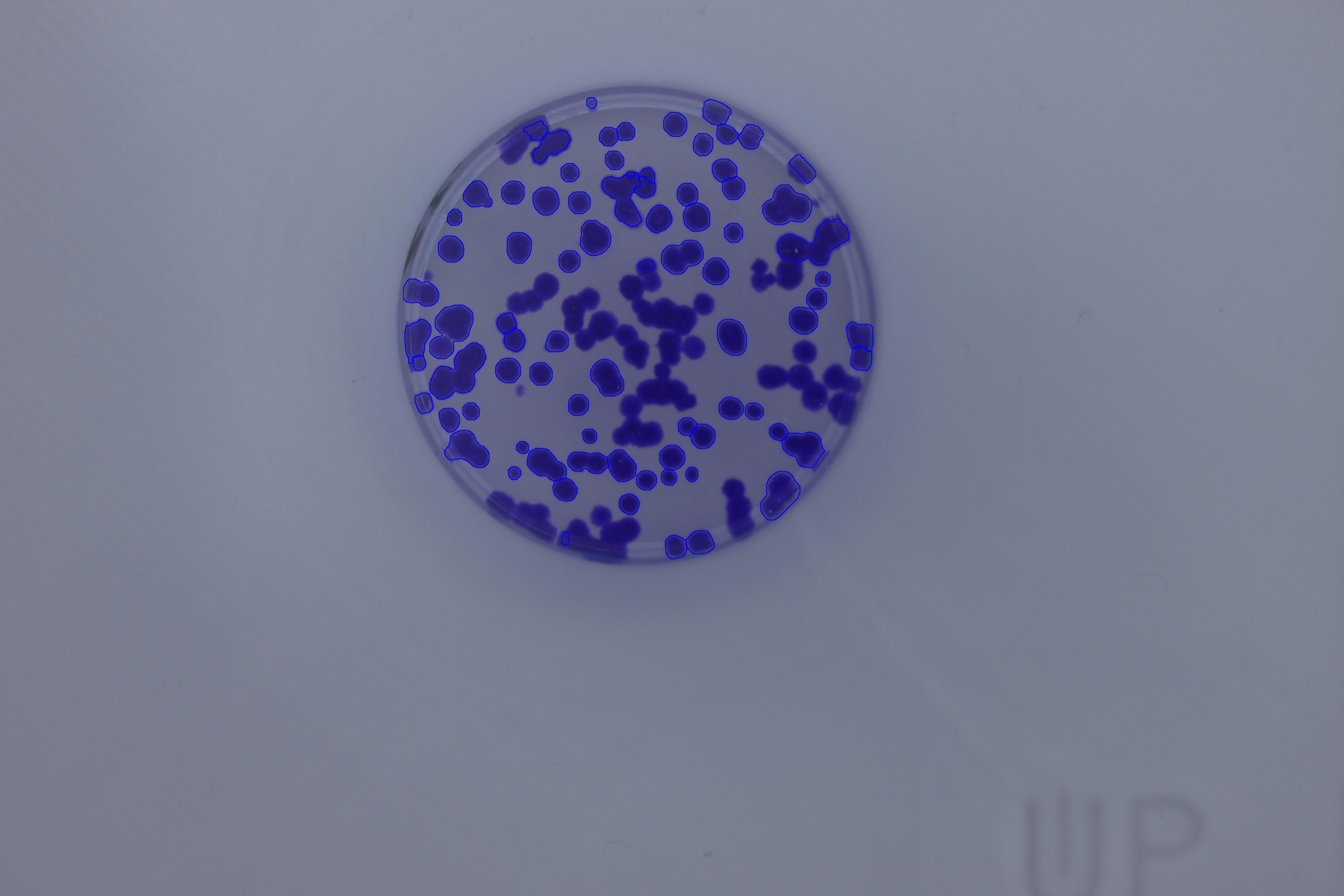

Supplement: S1 Comparison to others — (ZIP) [file pone.0205823.s007.zip › S1 Comparison to others/AutoCellSeg/171214 V79 Dish/14_seg.jpg]

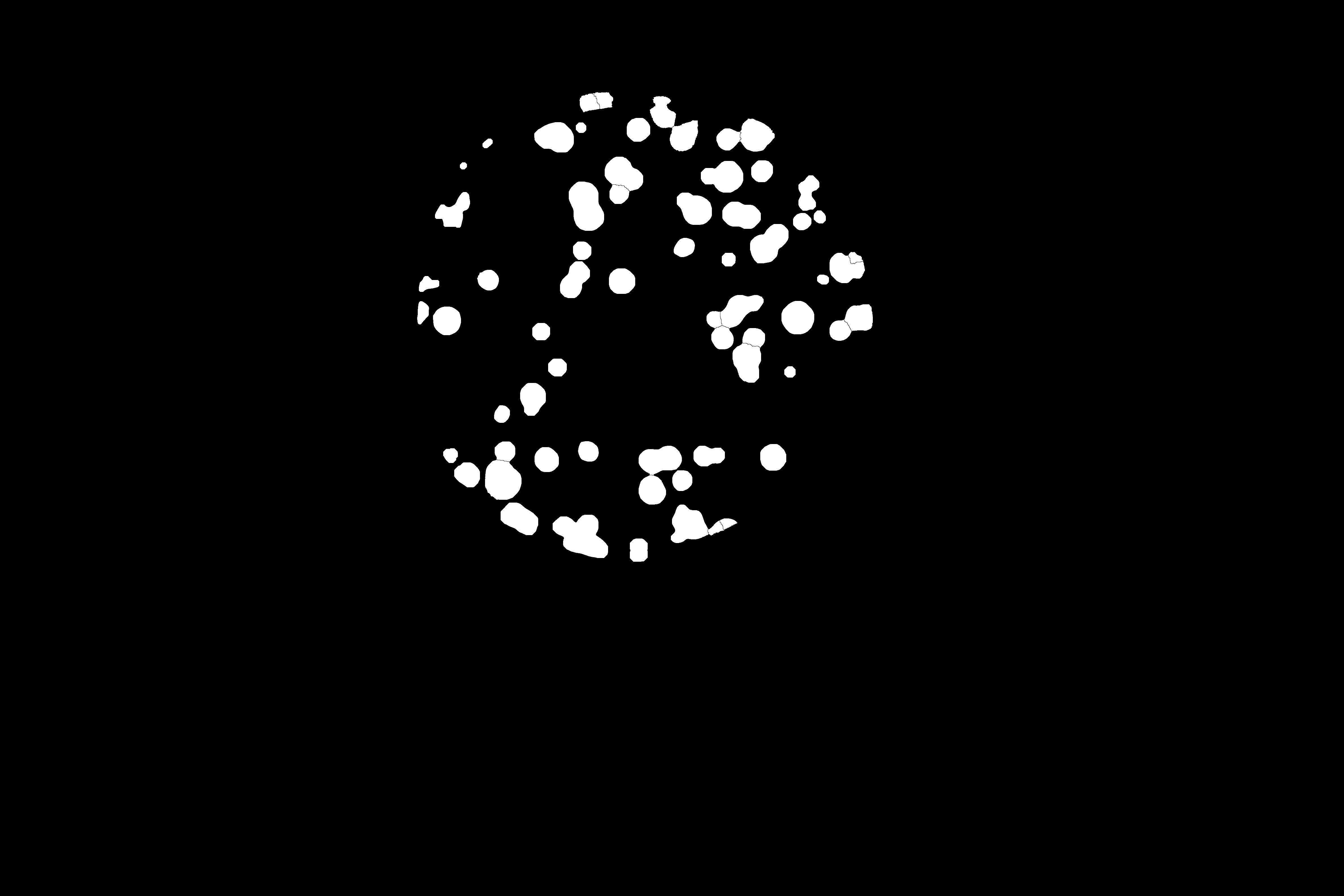

Supplement: S1 Comparison to others — (ZIP) [file pone.0205823.s007.zip › S1 Comparison to others/AutoCellSeg/171214 V79 Dish/15_mask.jpg]

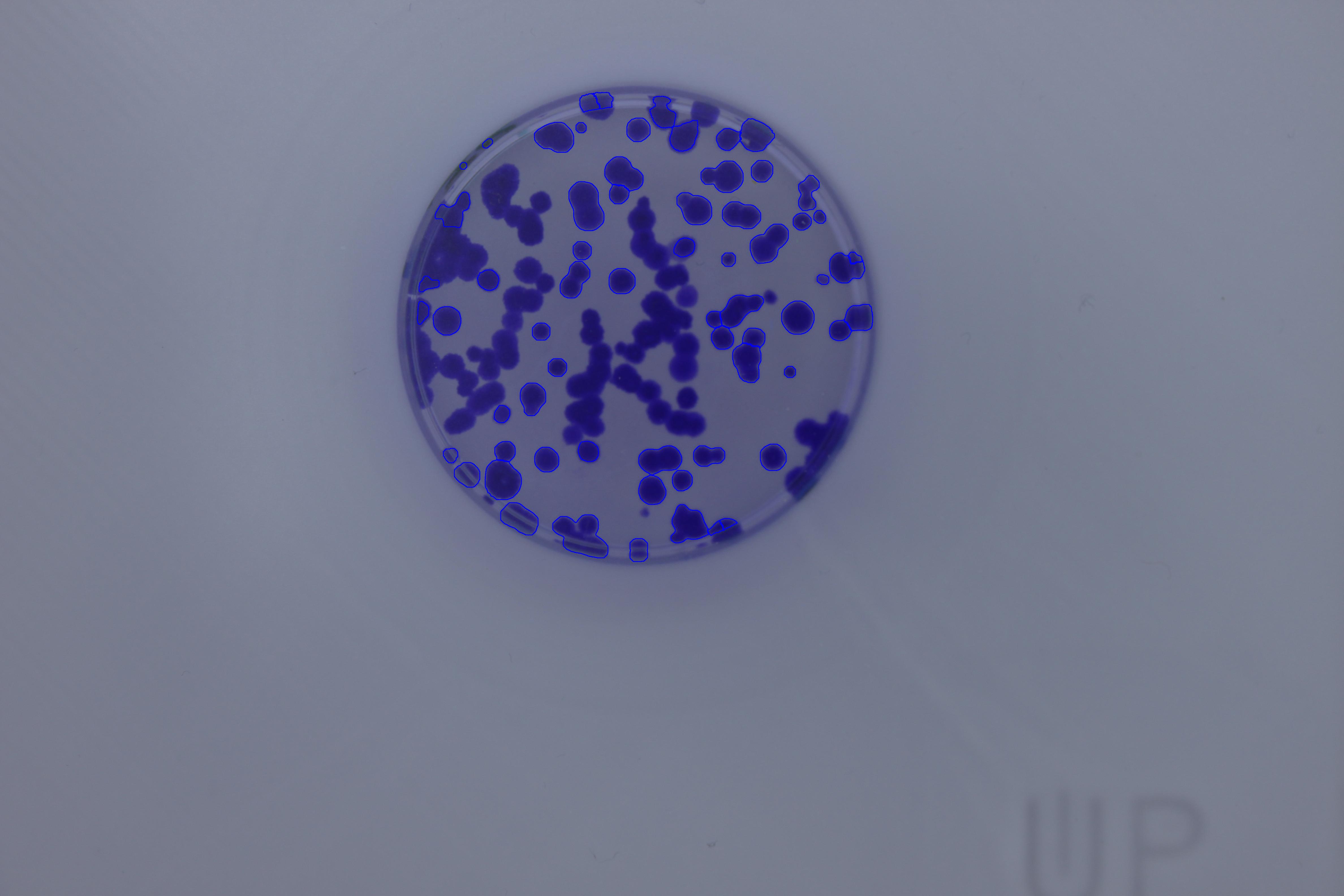

Supplement: S1 Comparison to others — (ZIP) [file pone.0205823.s007.zip › S1 Comparison to others/AutoCellSeg/171214 V79 Dish/15_seg.jpg]

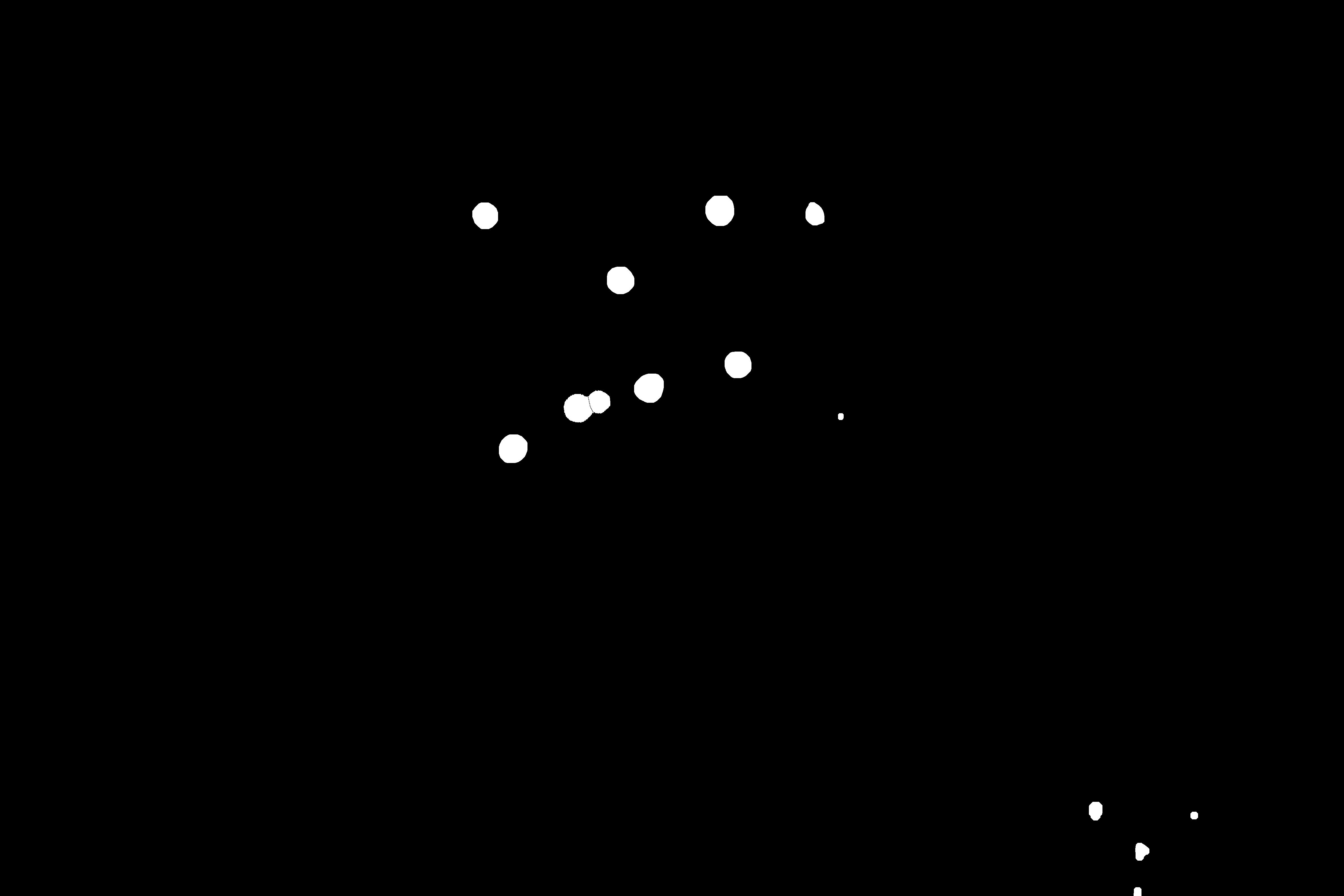

Supplement: S1 Comparison to others — (ZIP) [file pone.0205823.s007.zip › S1 Comparison to others/AutoCellSeg/171214 V79 Dish/1_mask.jpg]

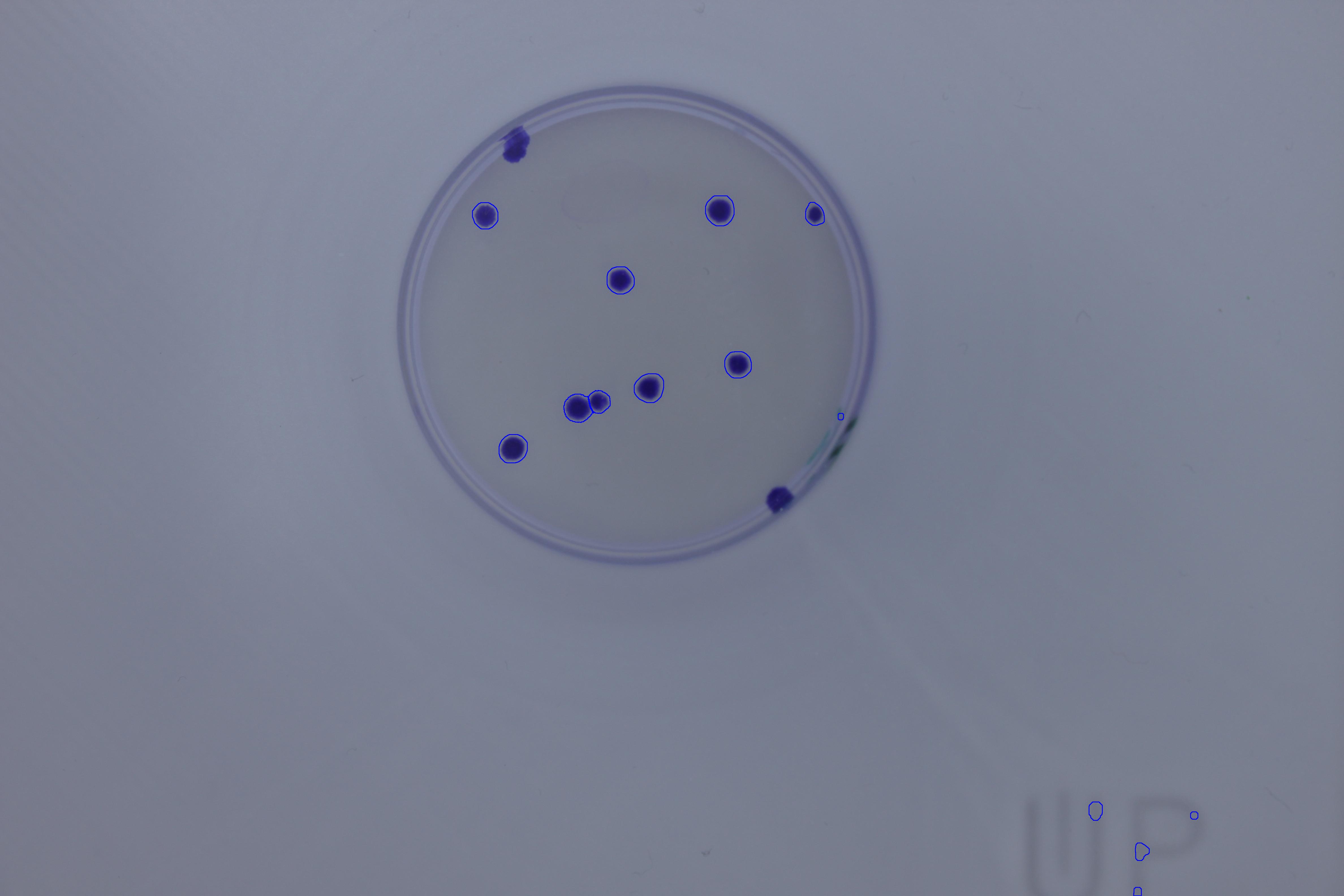

Supplement: S1 Comparison to others — (ZIP) [file pone.0205823.s007.zip › S1 Comparison to others/AutoCellSeg/171214 V79 Dish/1_seg.jpg]

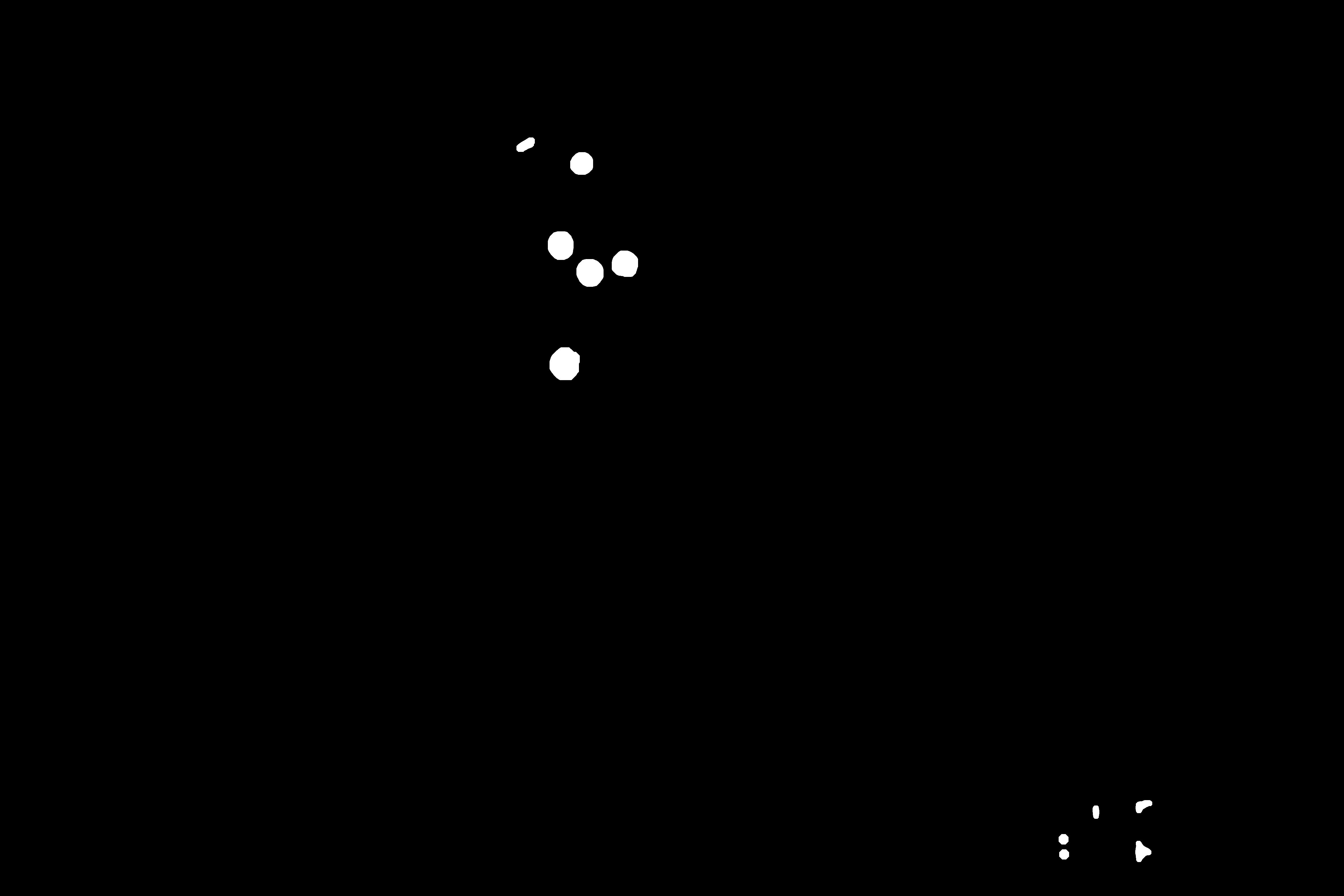

Supplement: S1 Comparison to others — (ZIP) [file pone.0205823.s007.zip › S1 Comparison to others/AutoCellSeg/171214 V79 Dish/2_mask.jpg]

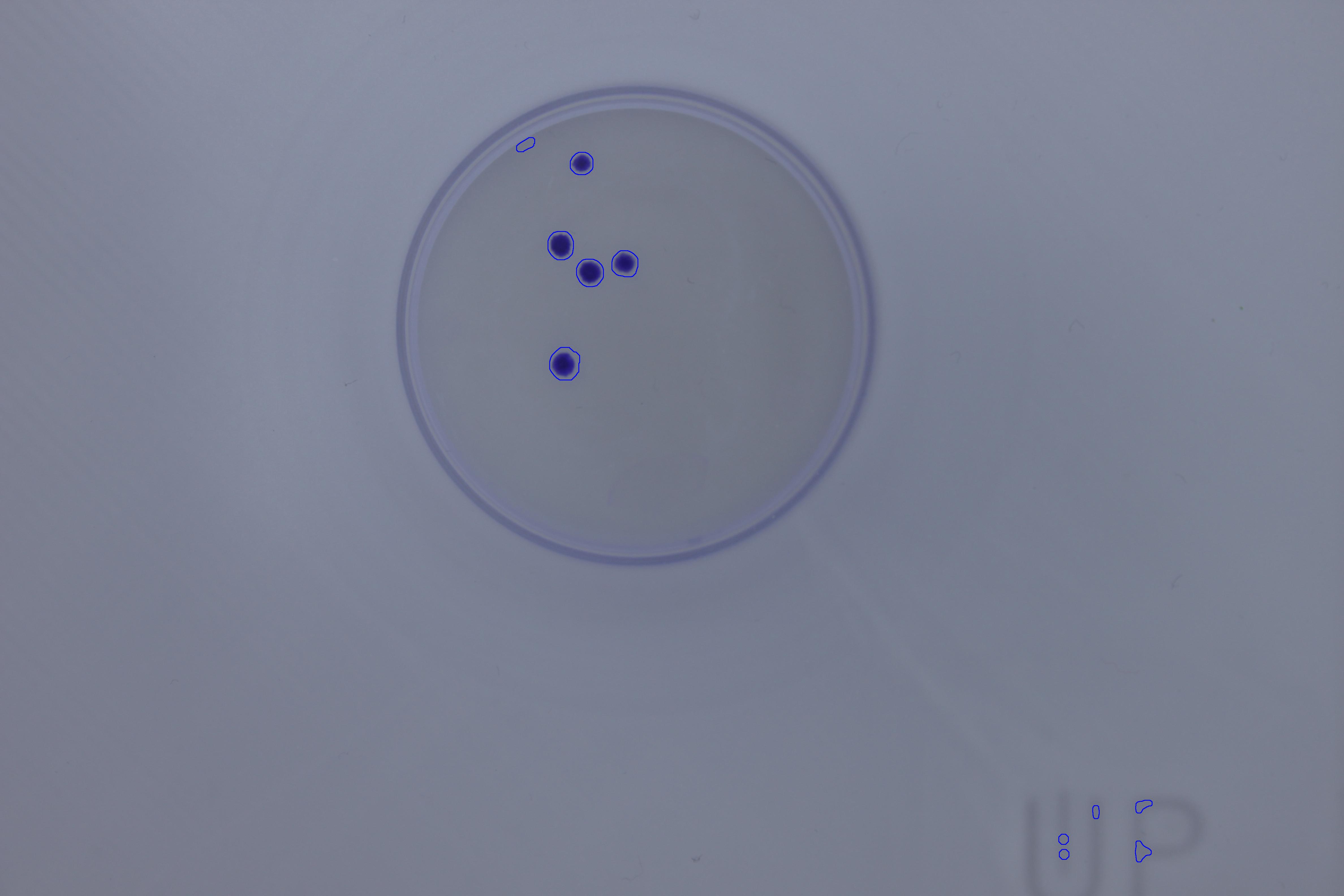

Supplement: S1 Comparison to others — (ZIP) [file pone.0205823.s007.zip › S1 Comparison to others/AutoCellSeg/171214 V79 Dish/2_seg.jpg]

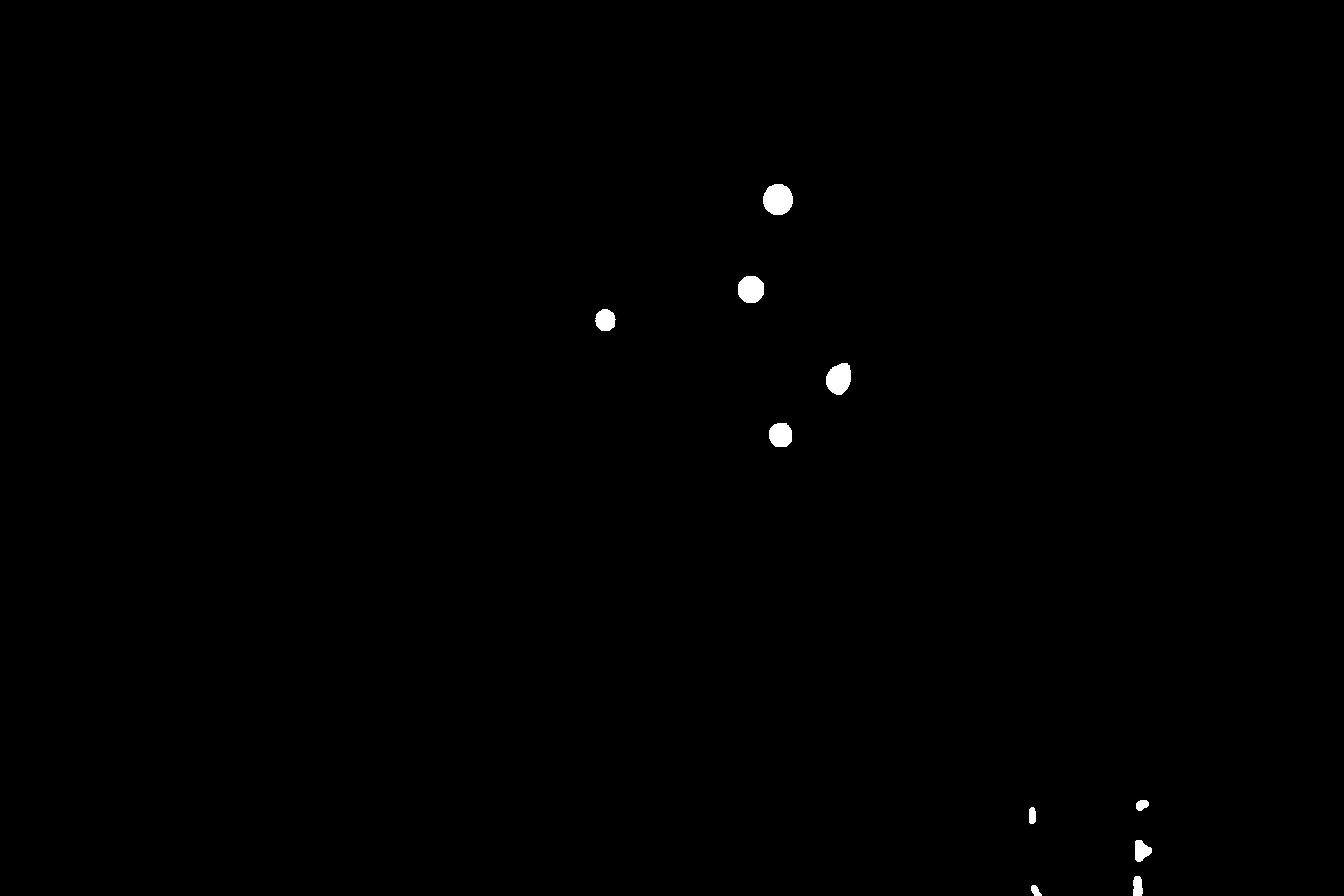

Supplement: S1 Comparison to others — (ZIP) [file pone.0205823.s007.zip › S1 Comparison to others/AutoCellSeg/171214 V79 Dish/3_mask.jpg]

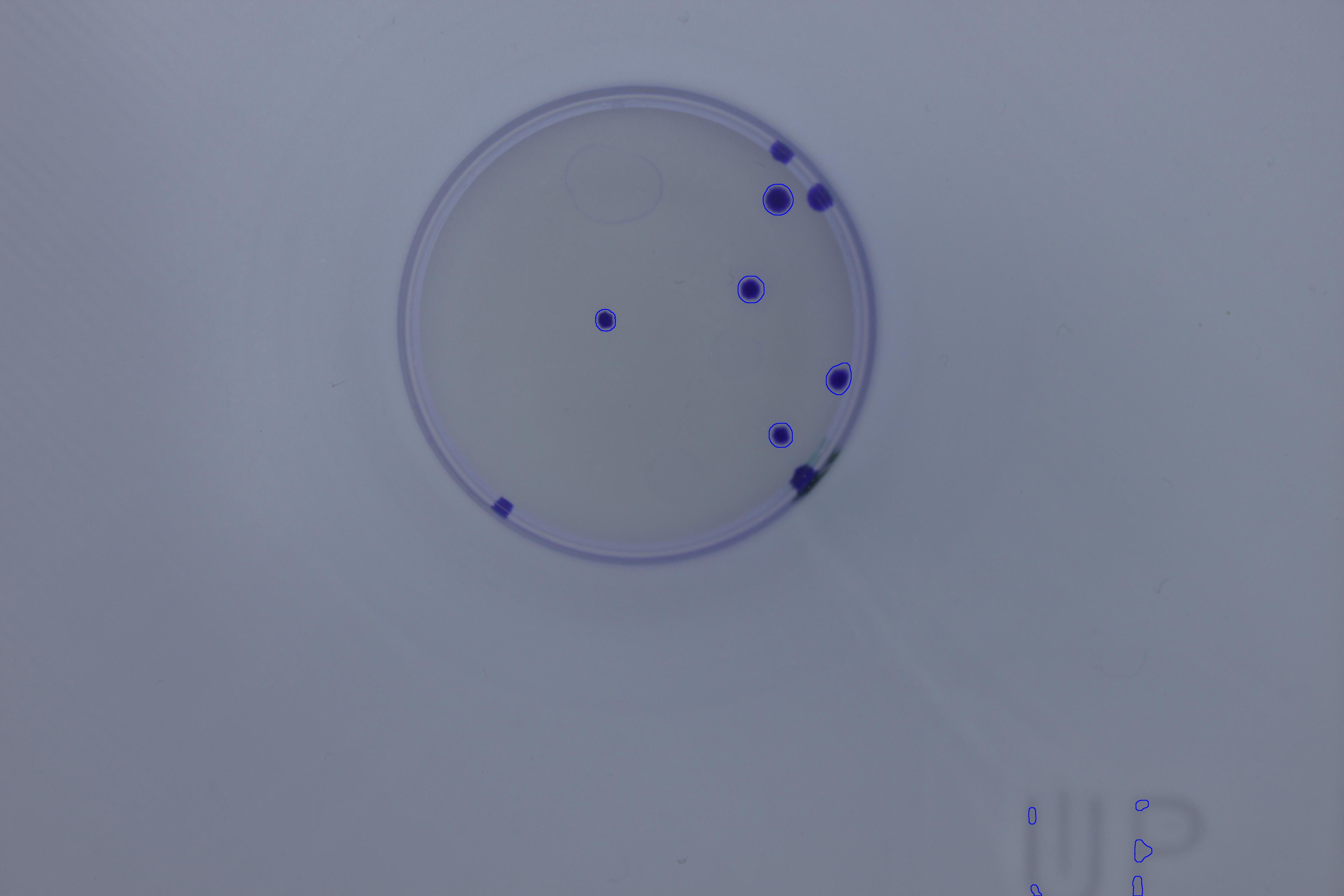

Supplement: S1 Comparison to others — (ZIP) [file pone.0205823.s007.zip › S1 Comparison to others/AutoCellSeg/171214 V79 Dish/3_seg.jpg]

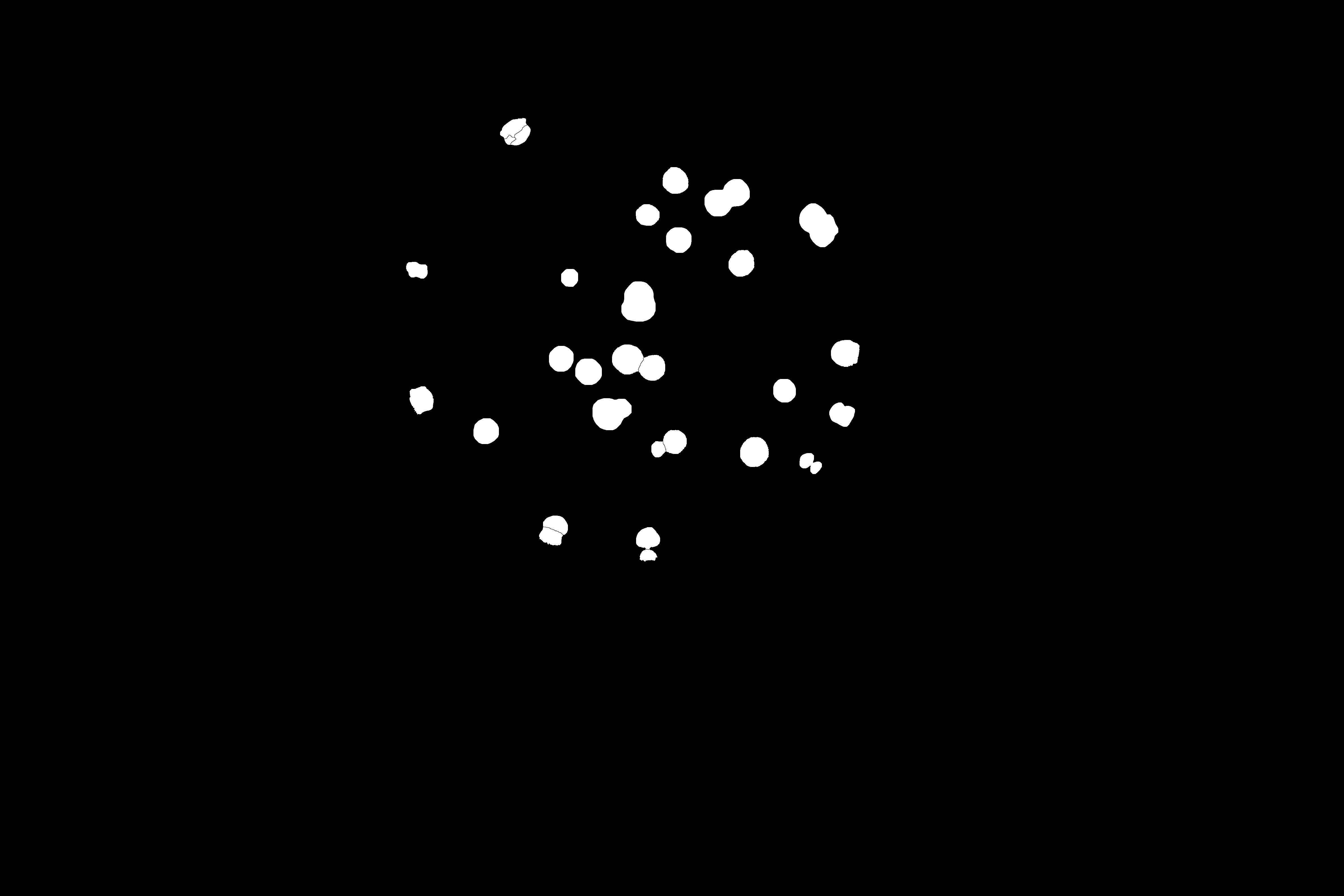

Supplement: S1 Comparison to others — (ZIP) [file pone.0205823.s007.zip › S1 Comparison to others/AutoCellSeg/171214 V79 Dish/4_mask.jpg]

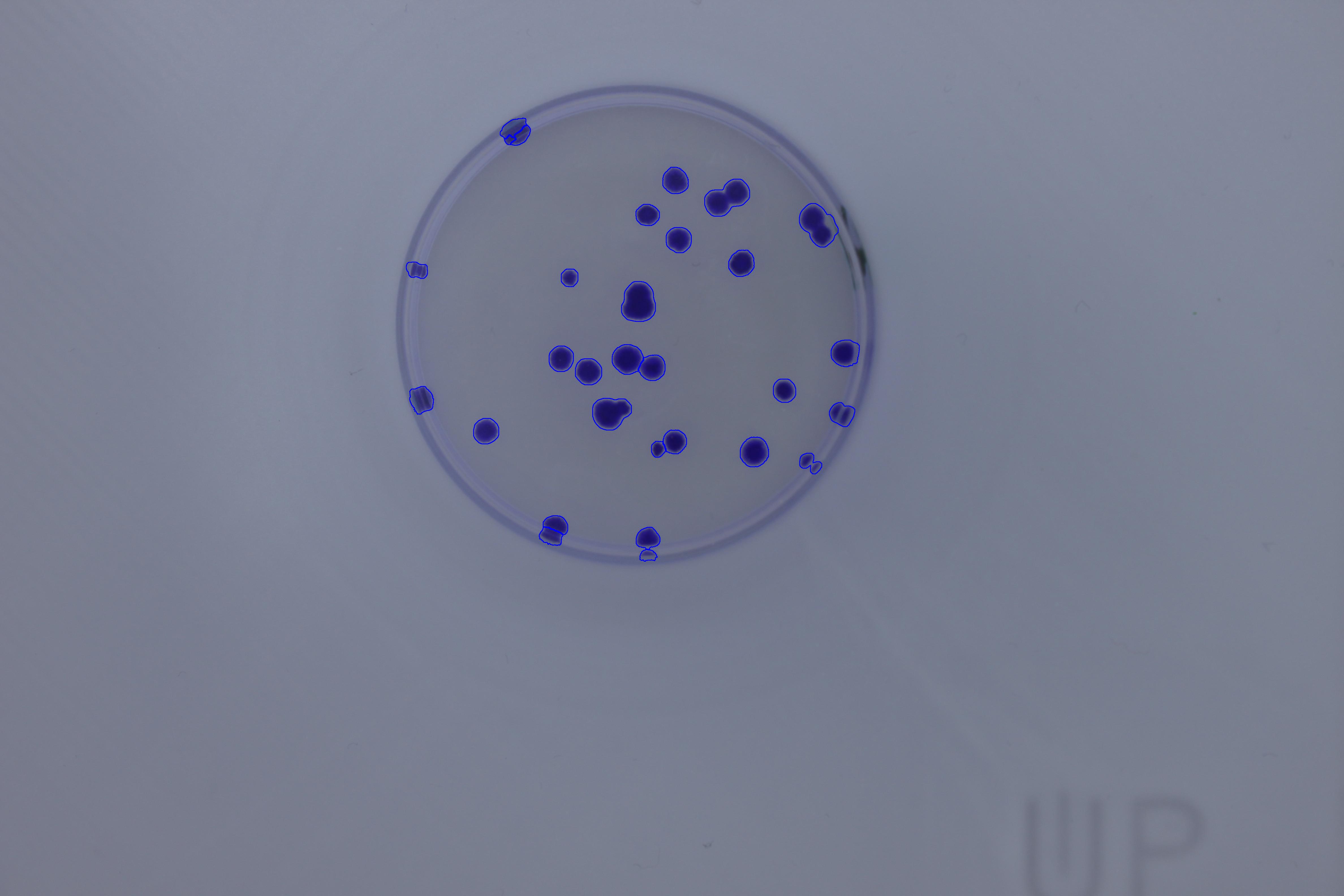

Supplement: S1 Comparison to others — (ZIP) [file pone.0205823.s007.zip › S1 Comparison to others/AutoCellSeg/171214 V79 Dish/4_seg.jpg]

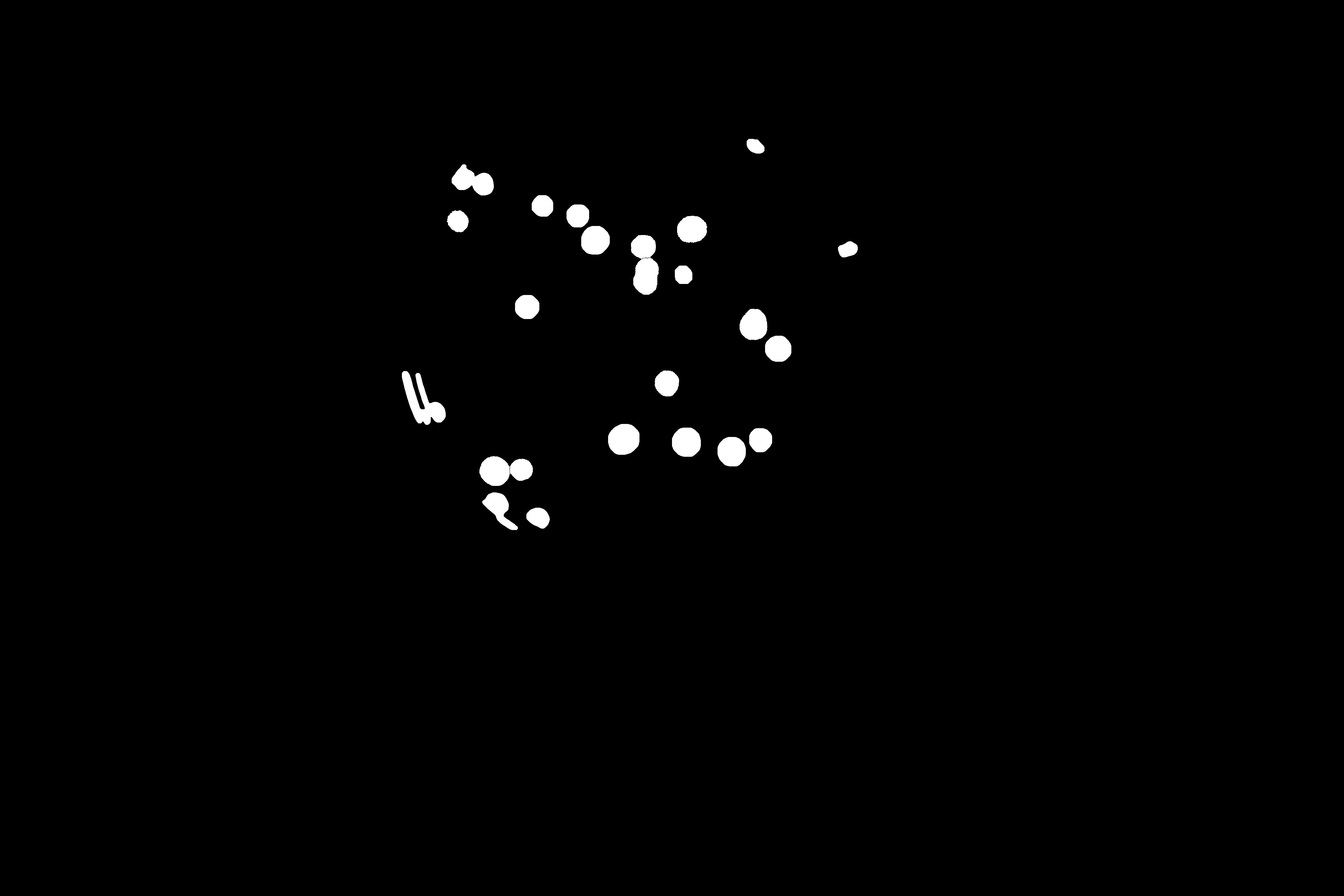

Supplement: S1 Comparison to others — (ZIP) [file pone.0205823.s007.zip › S1 Comparison to others/AutoCellSeg/171214 V79 Dish/5_mask.jpg]

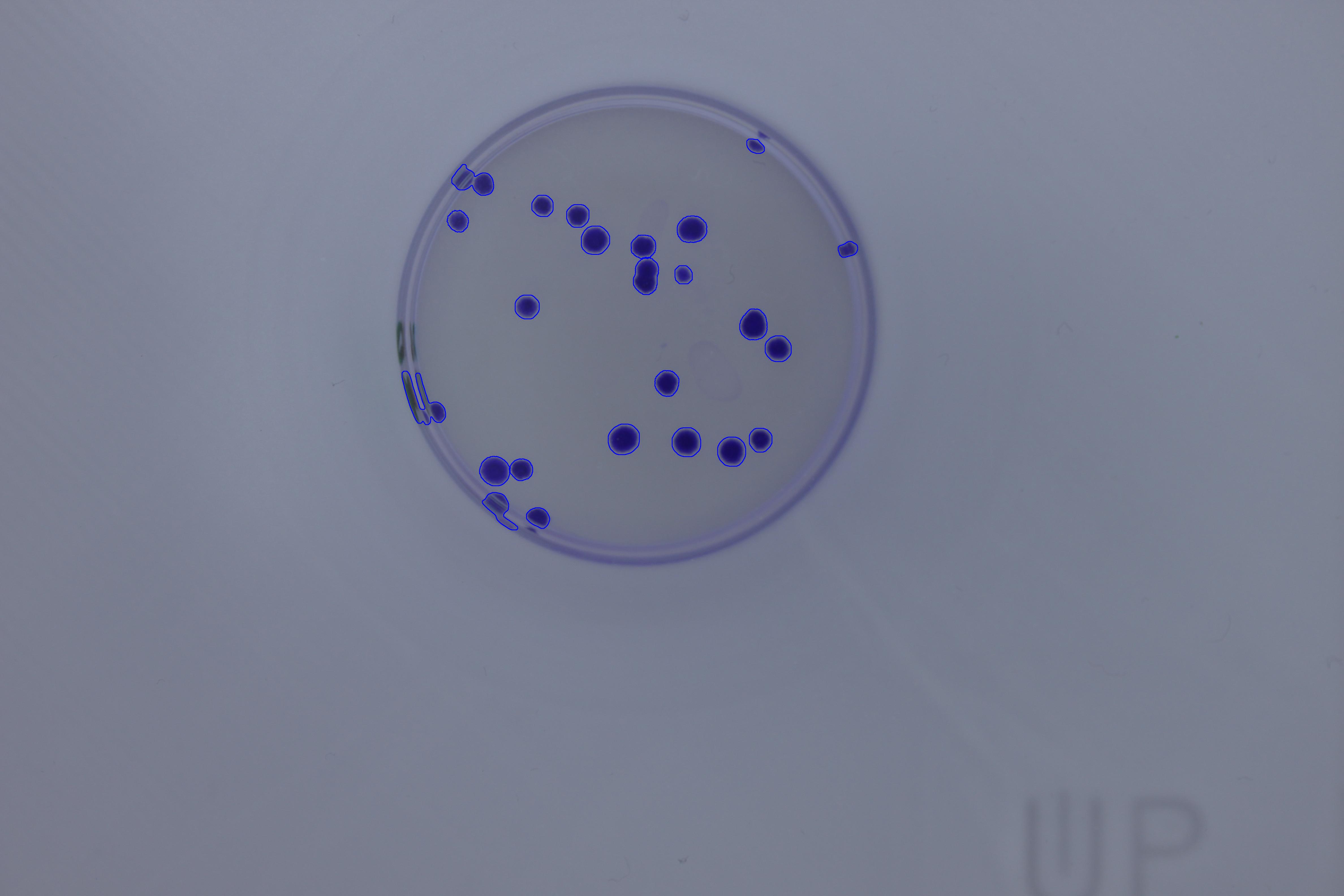

Supplement: S1 Comparison to others — (ZIP) [file pone.0205823.s007.zip › S1 Comparison to others/AutoCellSeg/171214 V79 Dish/5_seg.jpg]

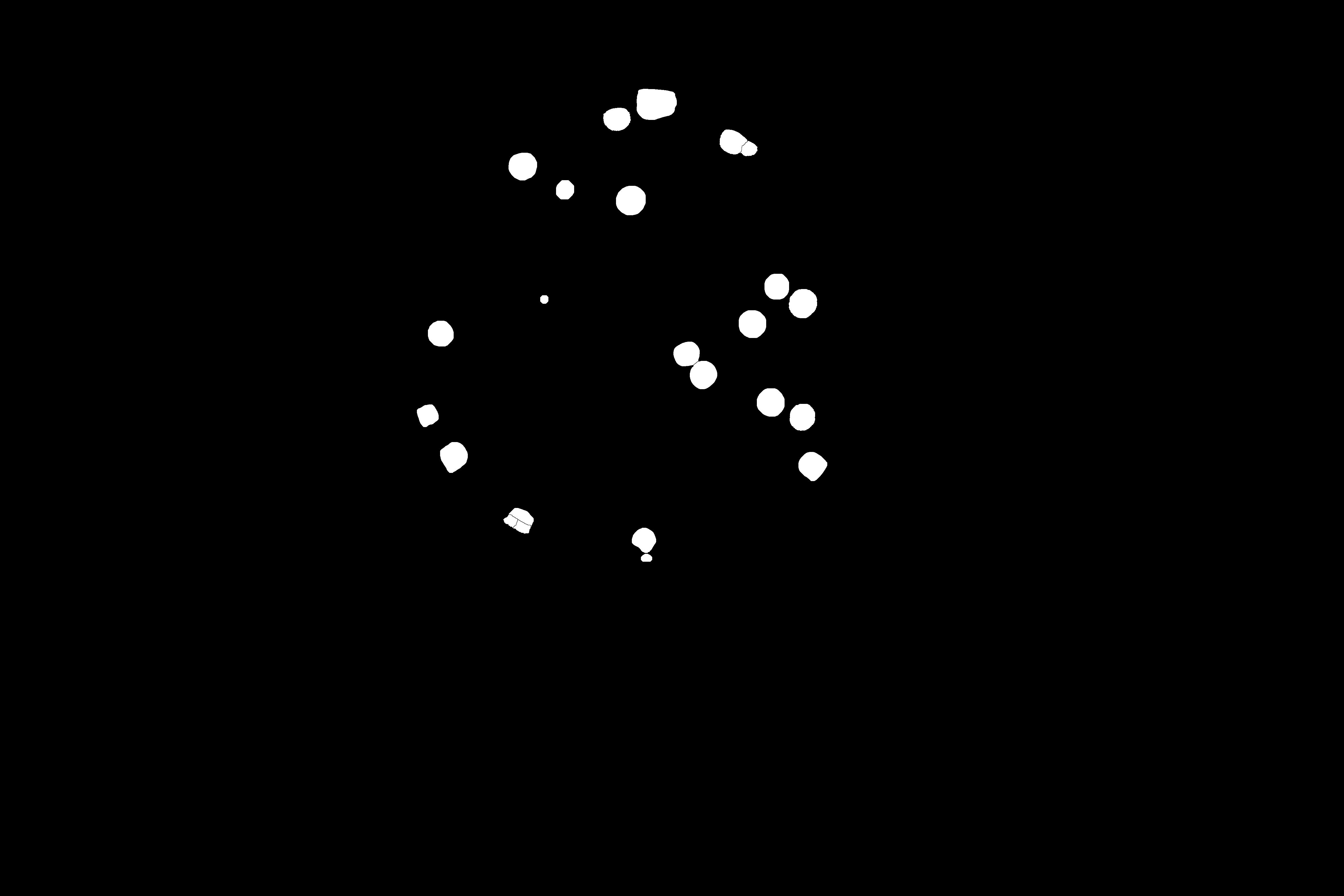

Supplement: S1 Comparison to others — (ZIP) [file pone.0205823.s007.zip › S1 Comparison to others/AutoCellSeg/171214 V79 Dish/6_mask.jpg]

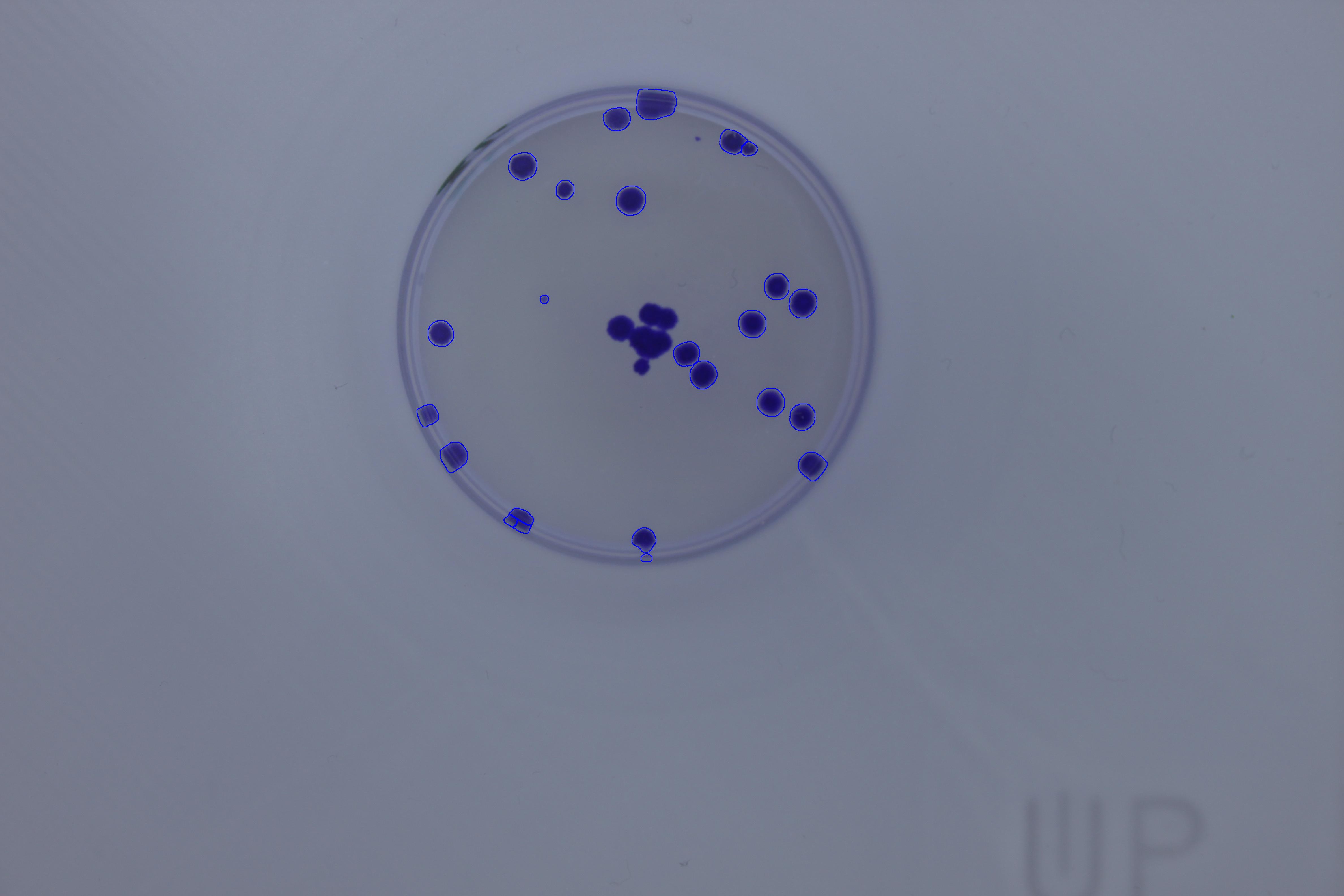

Supplement: S1 Comparison to others — (ZIP) [file pone.0205823.s007.zip › S1 Comparison to others/AutoCellSeg/171214 V79 Dish/6_seg.jpg]

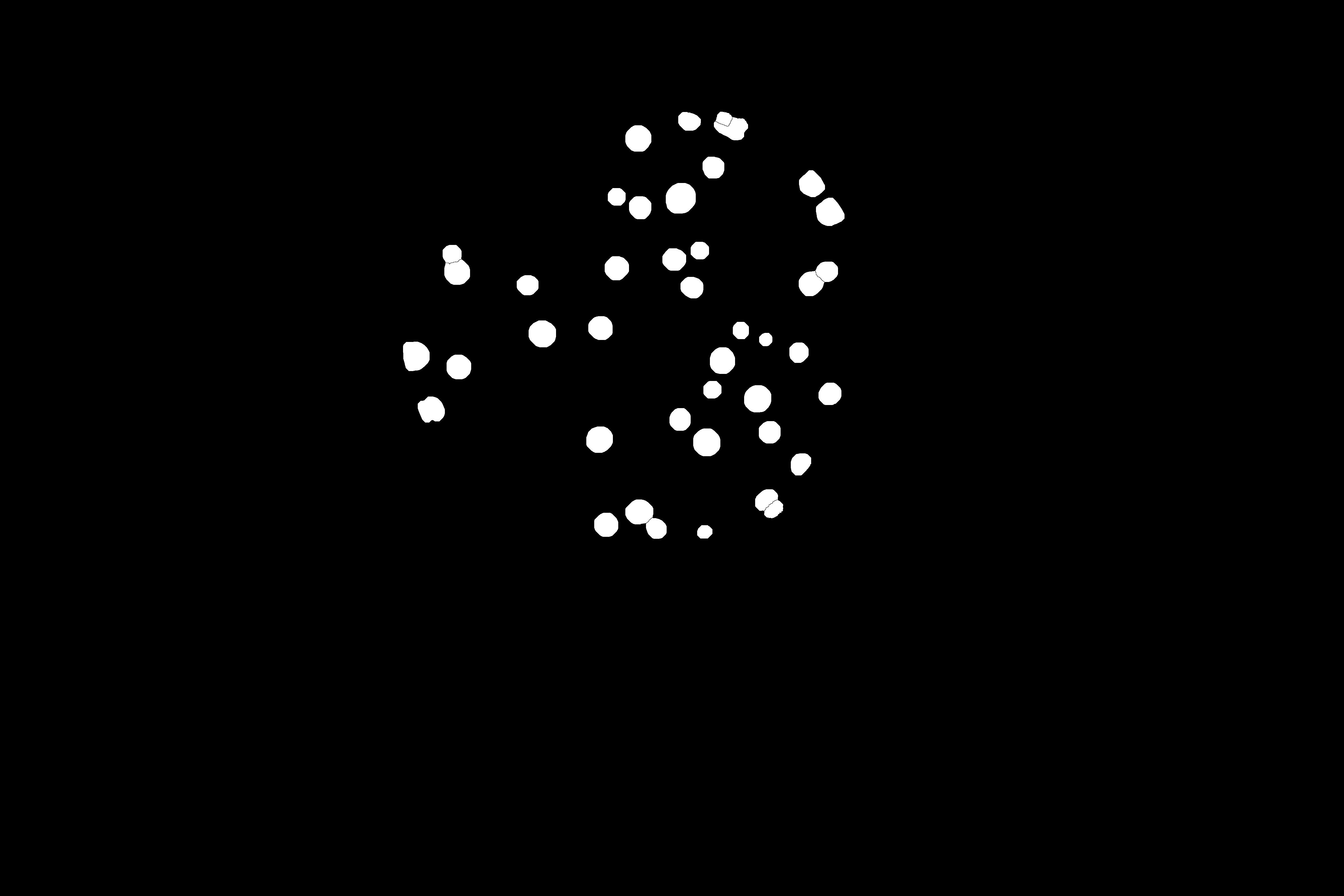

Supplement: S1 Comparison to others — (ZIP) [file pone.0205823.s007.zip › S1 Comparison to others/AutoCellSeg/171214 V79 Dish/7_mask.jpg]

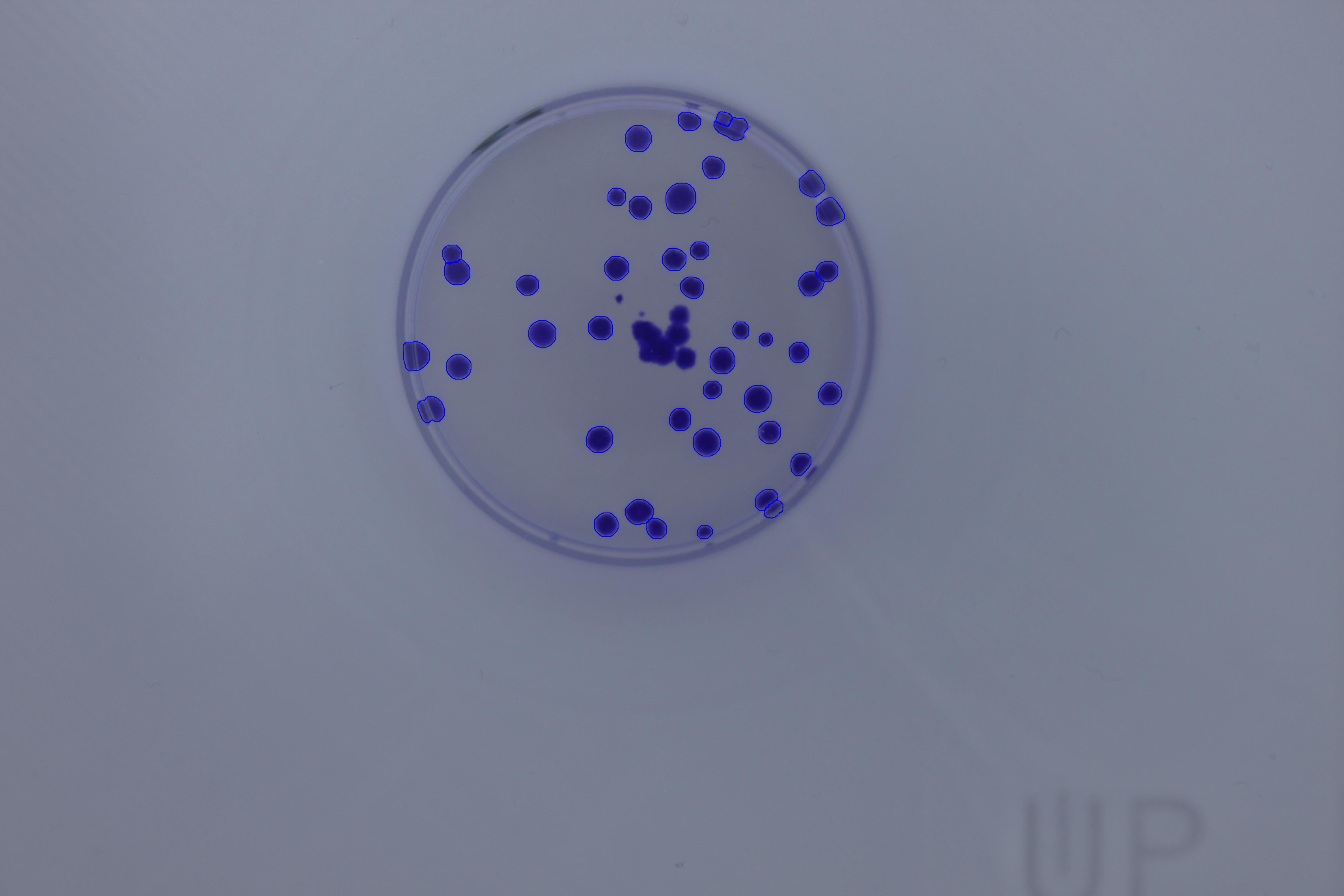

Supplement: S1 Comparison to others — (ZIP) [file pone.0205823.s007.zip › S1 Comparison to others/AutoCellSeg/171214 V79 Dish/7_seg.jpg]

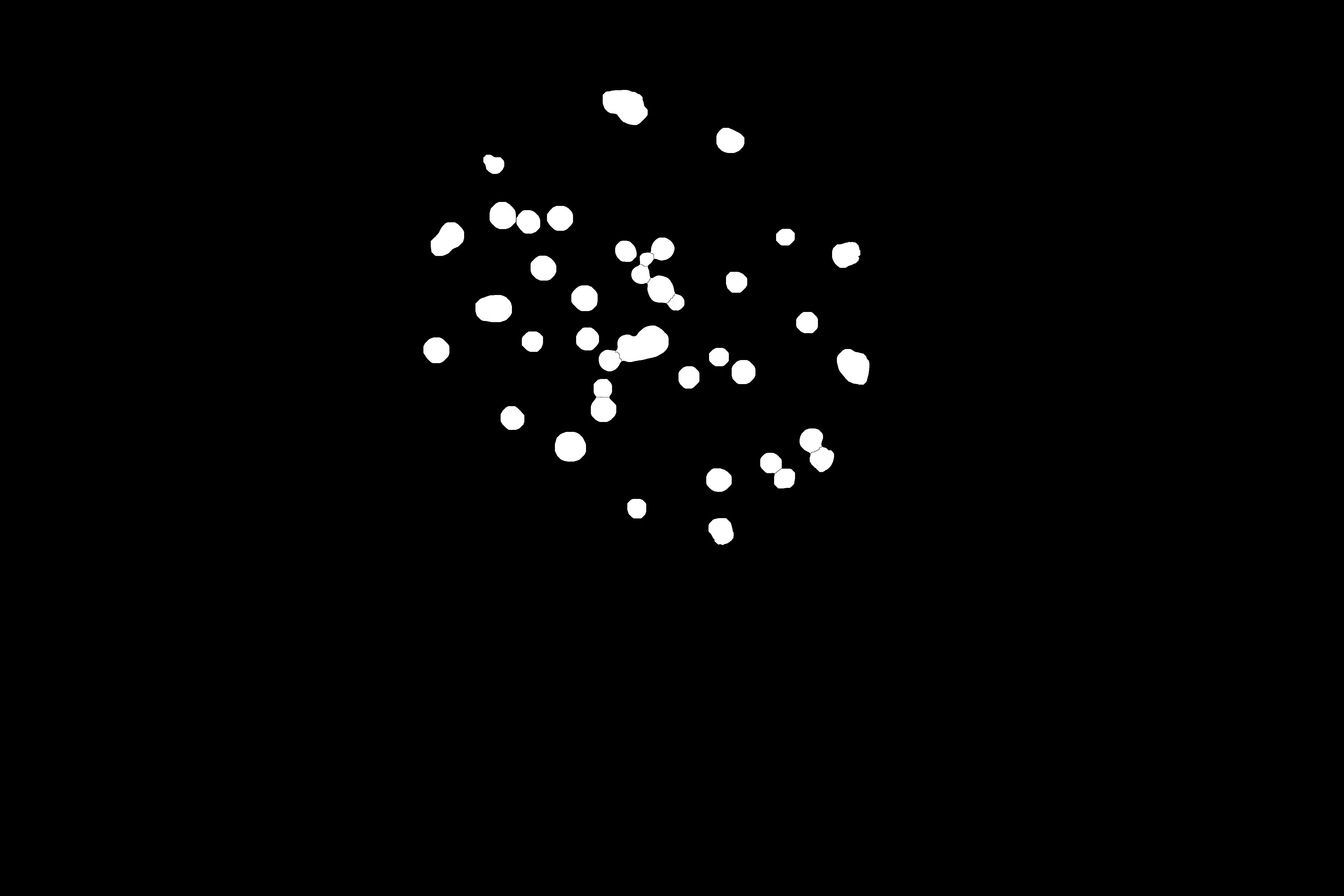

Supplement: S1 Comparison to others — (ZIP) [file pone.0205823.s007.zip › S1 Comparison to others/AutoCellSeg/171214 V79 Dish/8_mask.jpg]

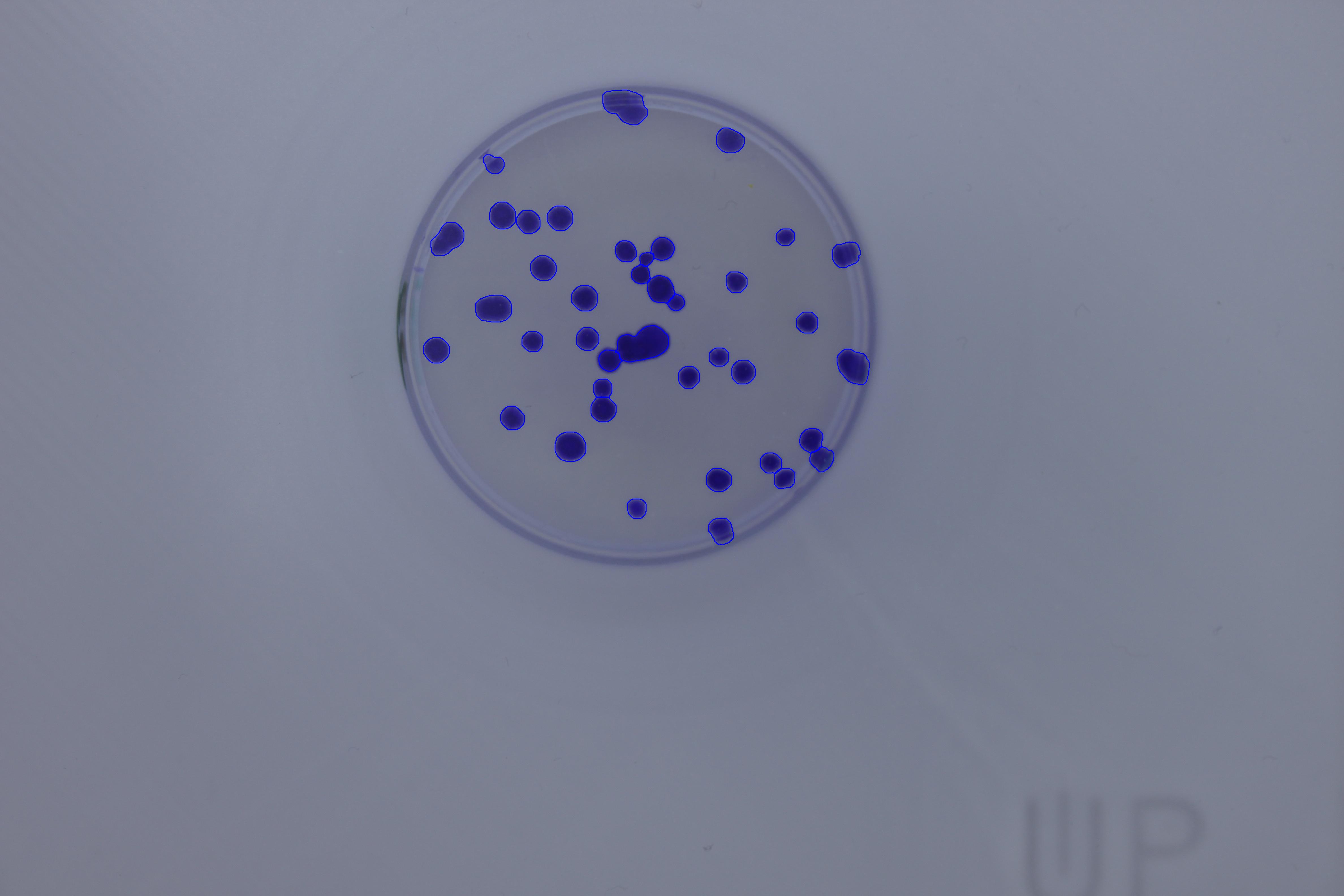

Supplement: S1 Comparison to others — (ZIP) [file pone.0205823.s007.zip › S1 Comparison to others/AutoCellSeg/171214 V79 Dish/8_seg.jpg]

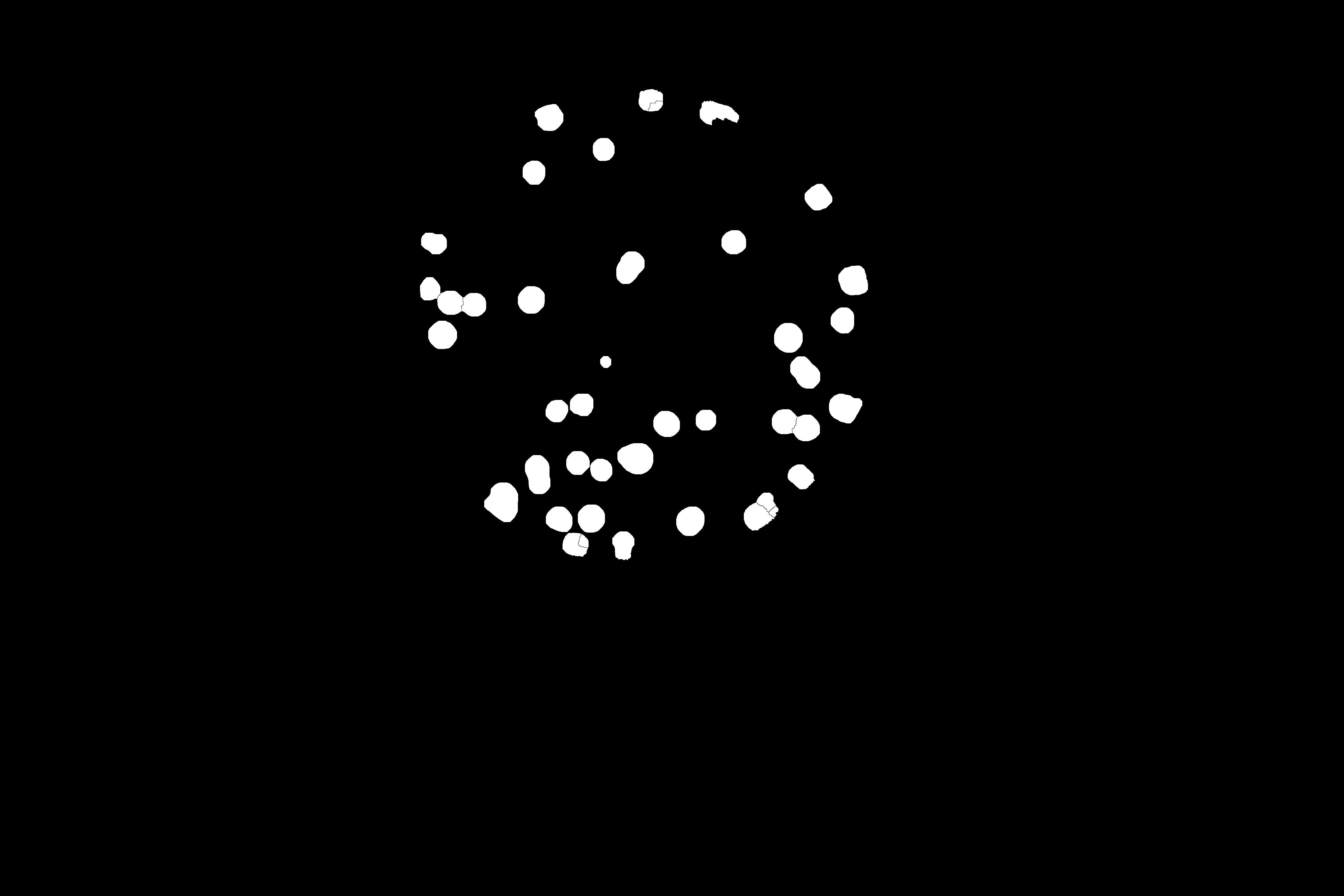

Supplement: S1 Comparison to others — (ZIP) [file pone.0205823.s007.zip › S1 Comparison to others/AutoCellSeg/171214 V79 Dish/9_mask.jpg]

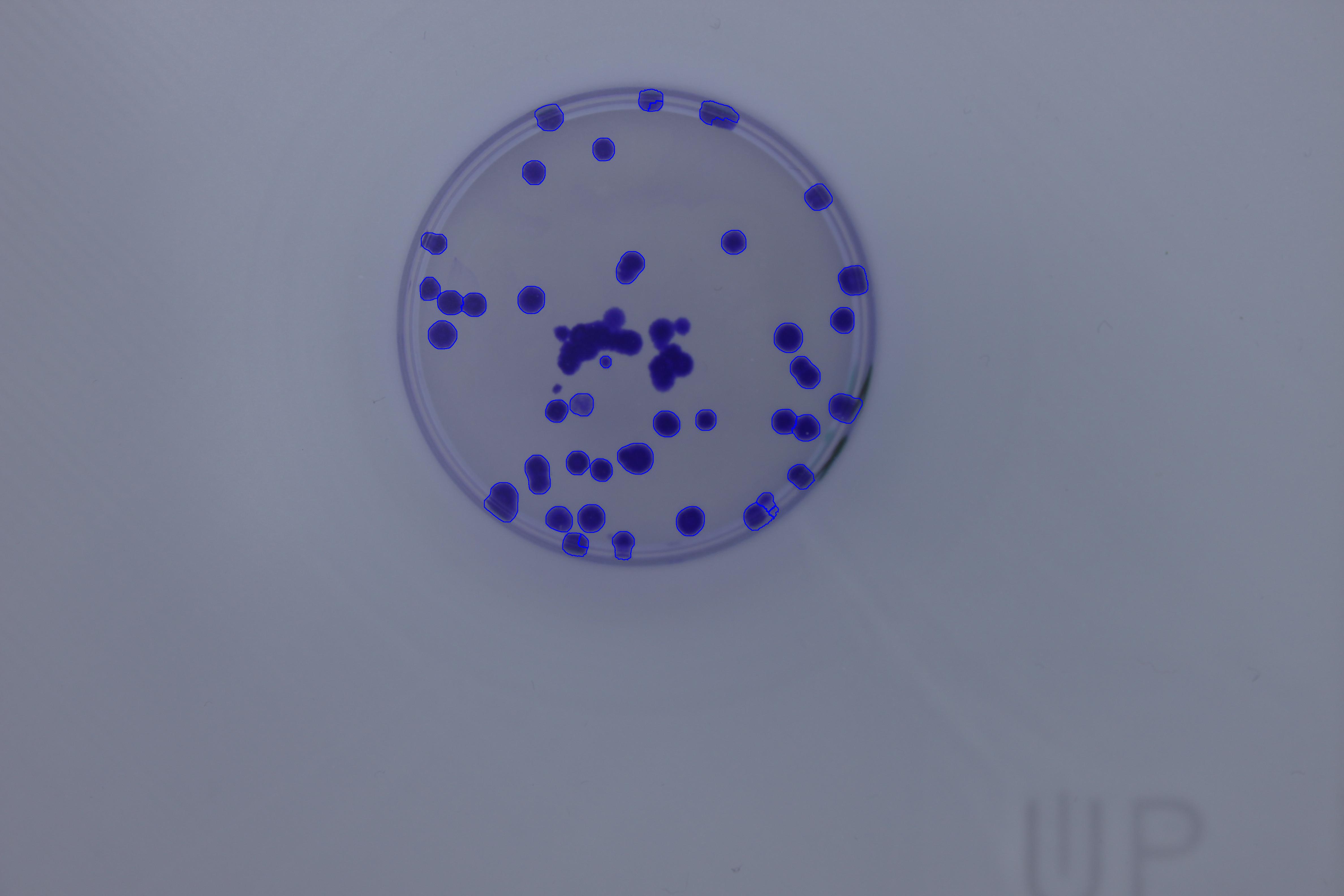

Supplement: S1 Comparison to others — (ZIP) [file pone.0205823.s007.zip › S1 Comparison to others/AutoCellSeg/171214 V79 Dish/9_seg.jpg]

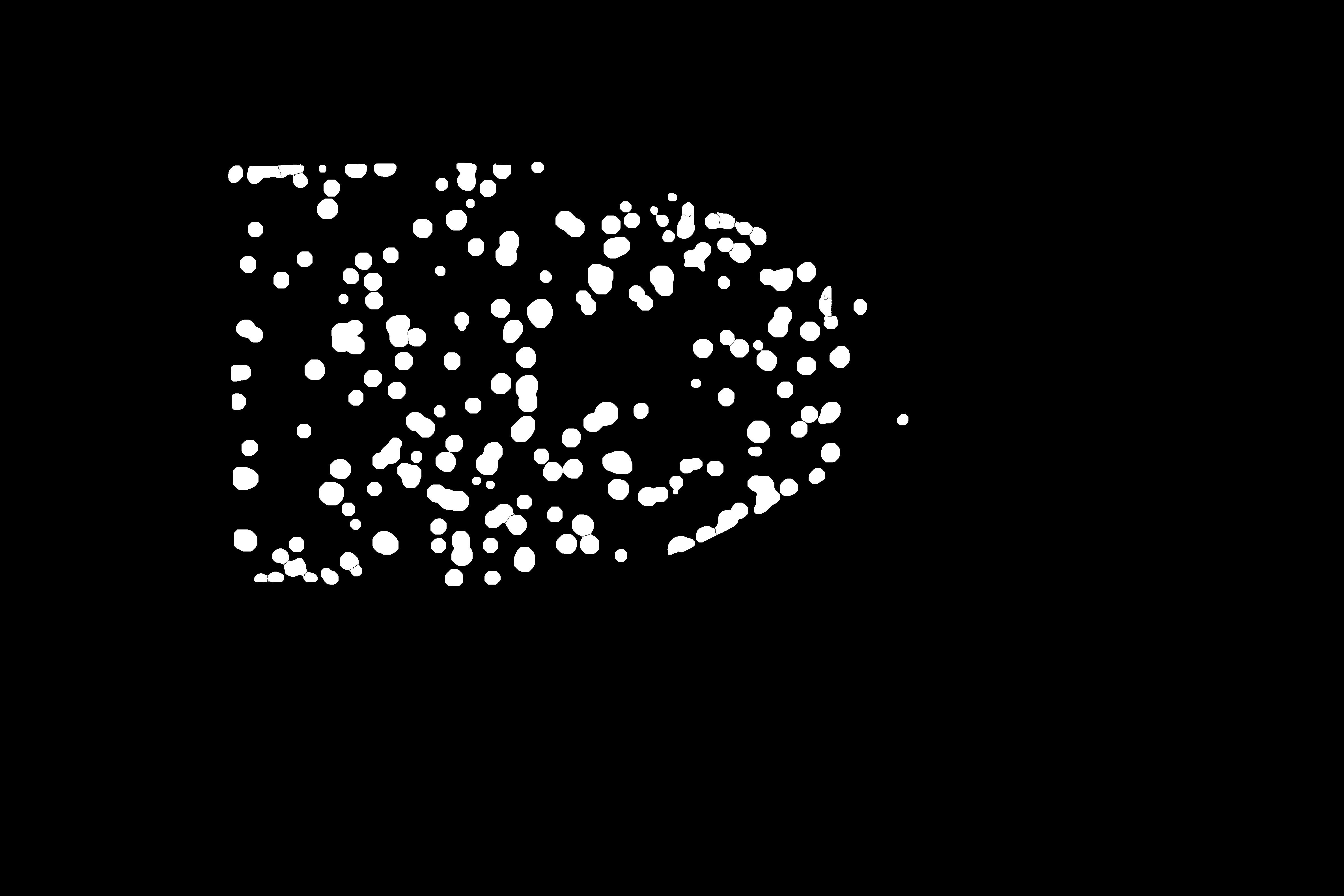

Supplement: S1 Comparison to others — (ZIP) [file pone.0205823.s007.zip › S1 Comparison to others/AutoCellSeg/171214 V79 Flask/10_mask.jpg]

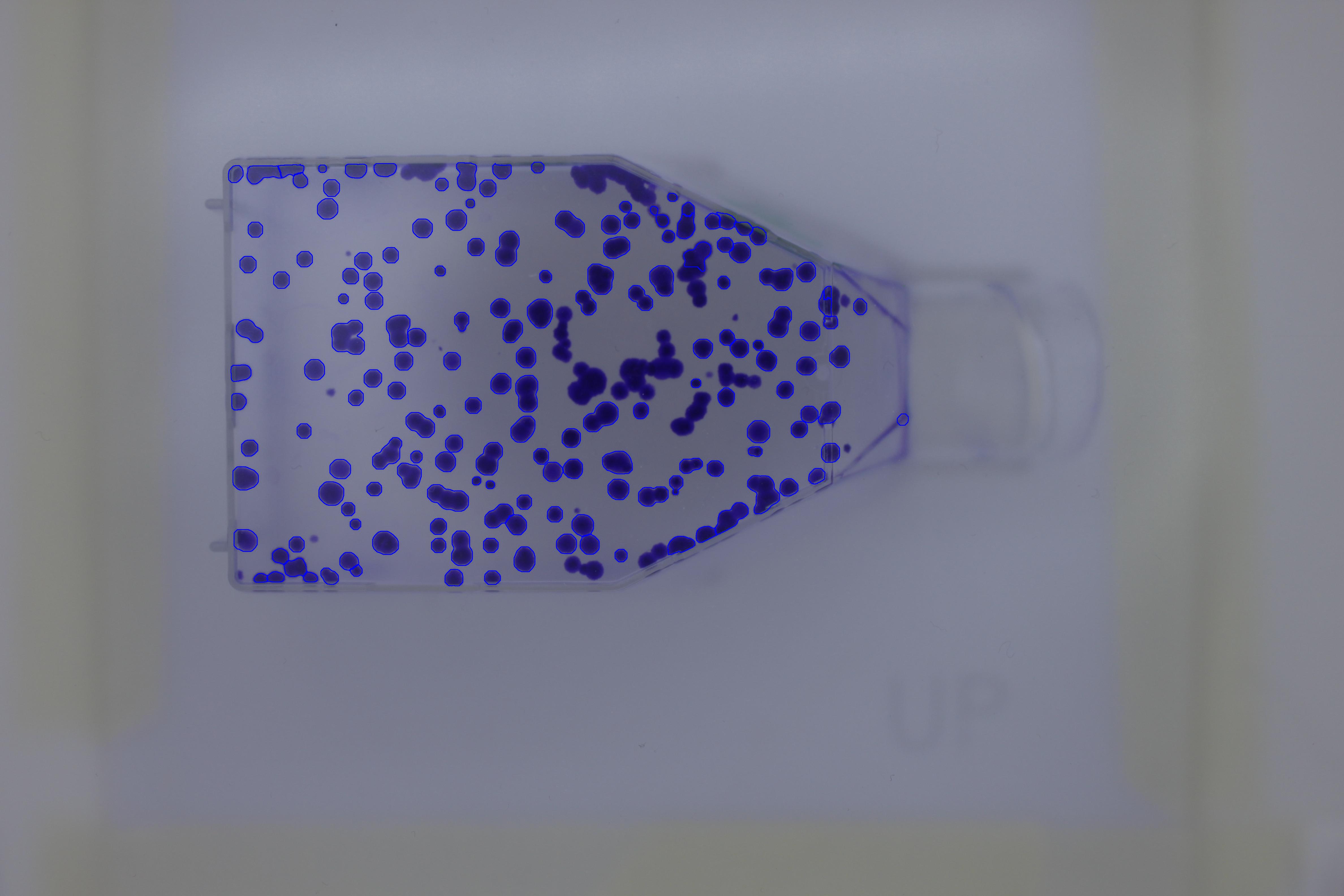

Supplement: S1 Comparison to others — (ZIP) [file pone.0205823.s007.zip › S1 Comparison to others/AutoCellSeg/171214 V79 Flask/10_seg.jpg]

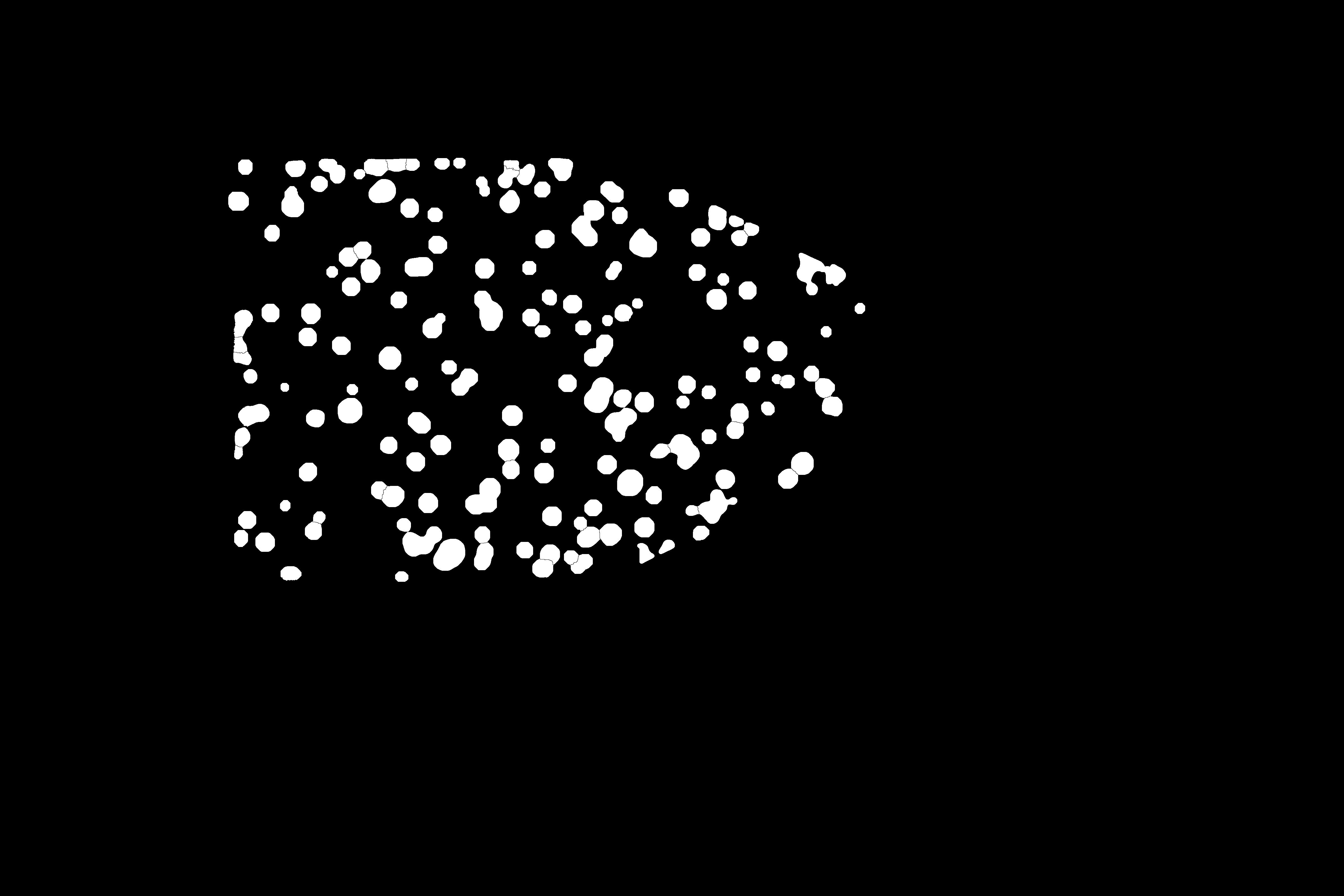

Supplement: S1 Comparison to others — (ZIP) [file pone.0205823.s007.zip › S1 Comparison to others/AutoCellSeg/171214 V79 Flask/11_mask.jpg]

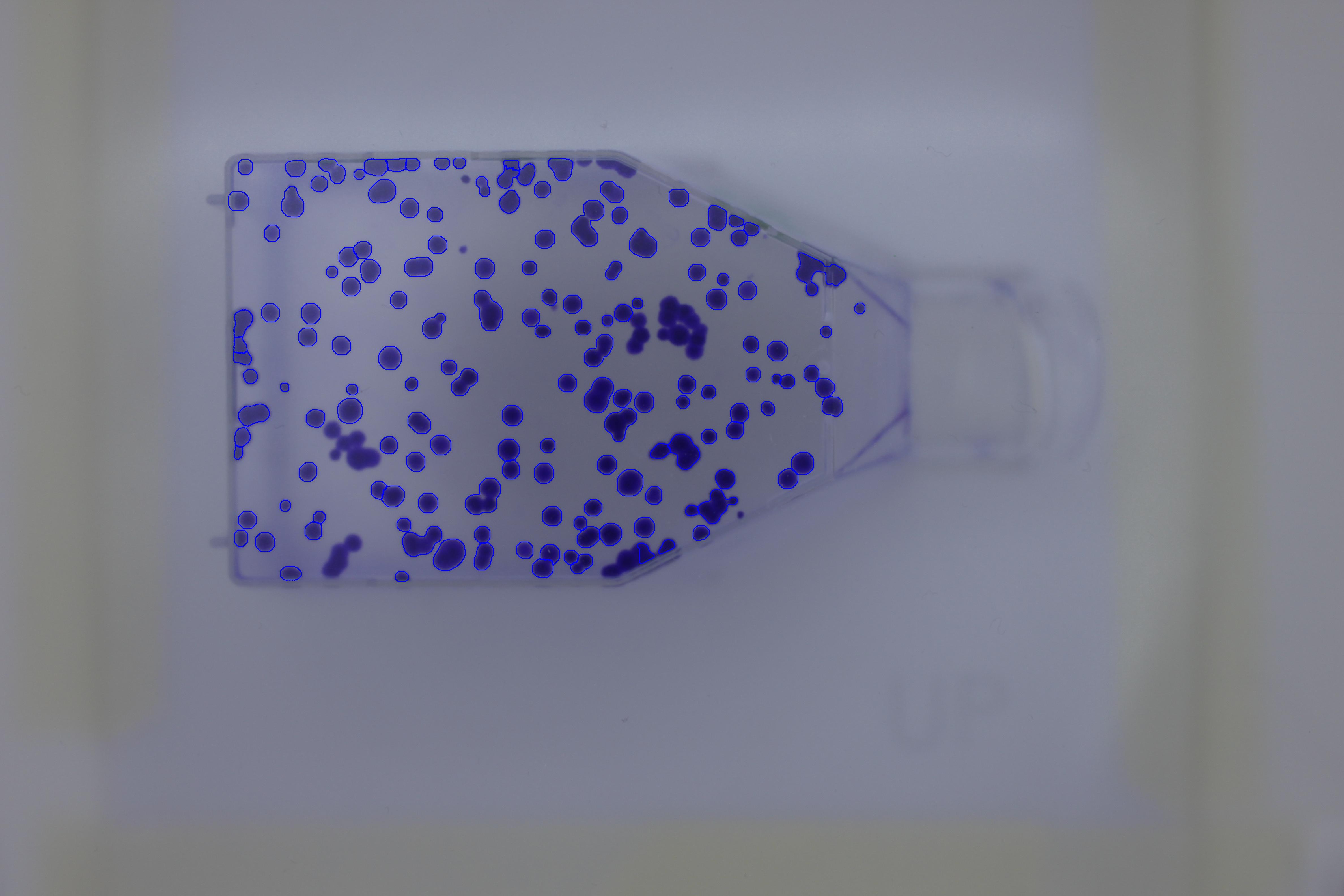

Supplement: S1 Comparison to others — (ZIP) [file pone.0205823.s007.zip › S1 Comparison to others/AutoCellSeg/171214 V79 Flask/11_seg.jpg]

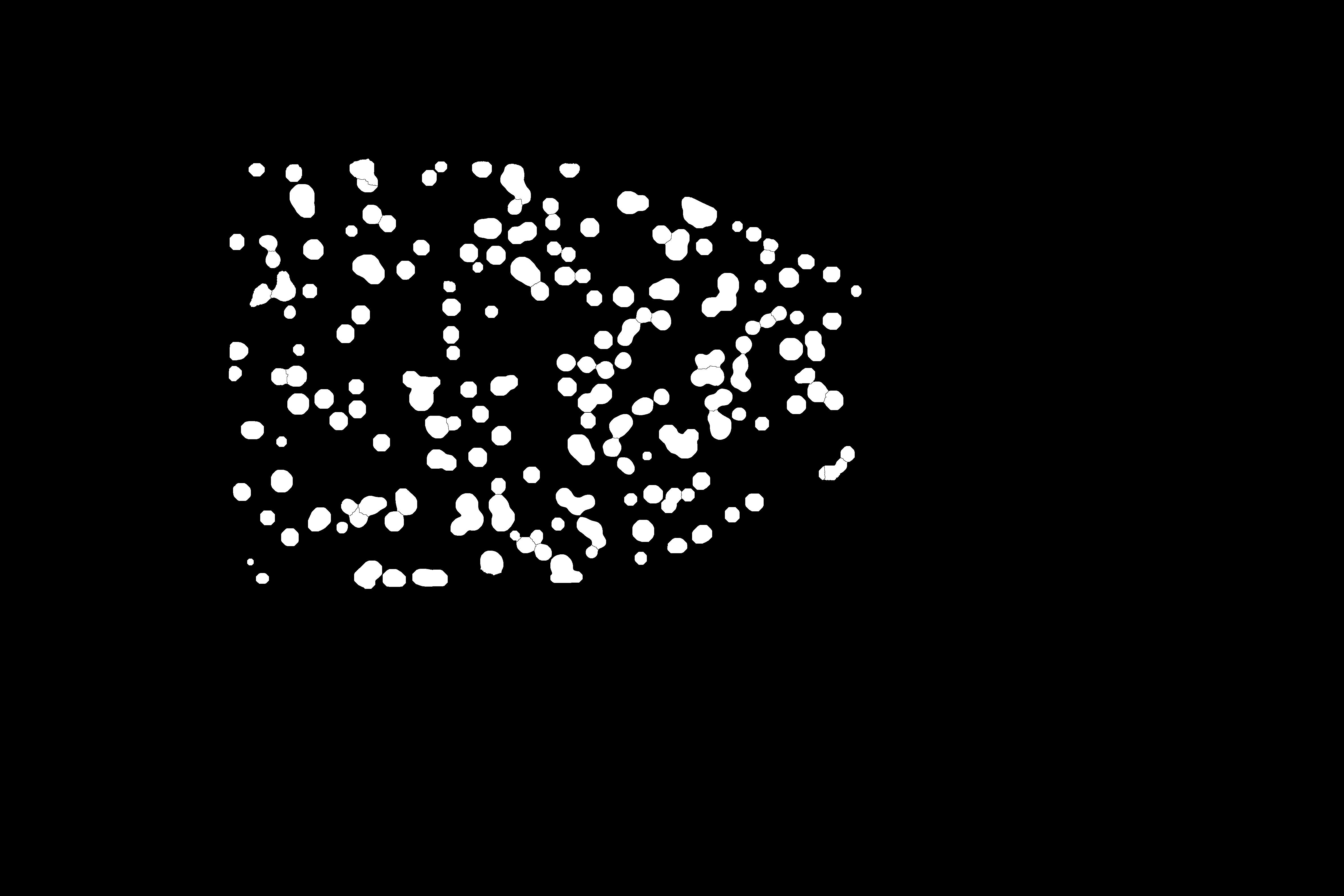

Supplement: S1 Comparison to others — (ZIP) [file pone.0205823.s007.zip › S1 Comparison to others/AutoCellSeg/171214 V79 Flask/12_mask.jpg]

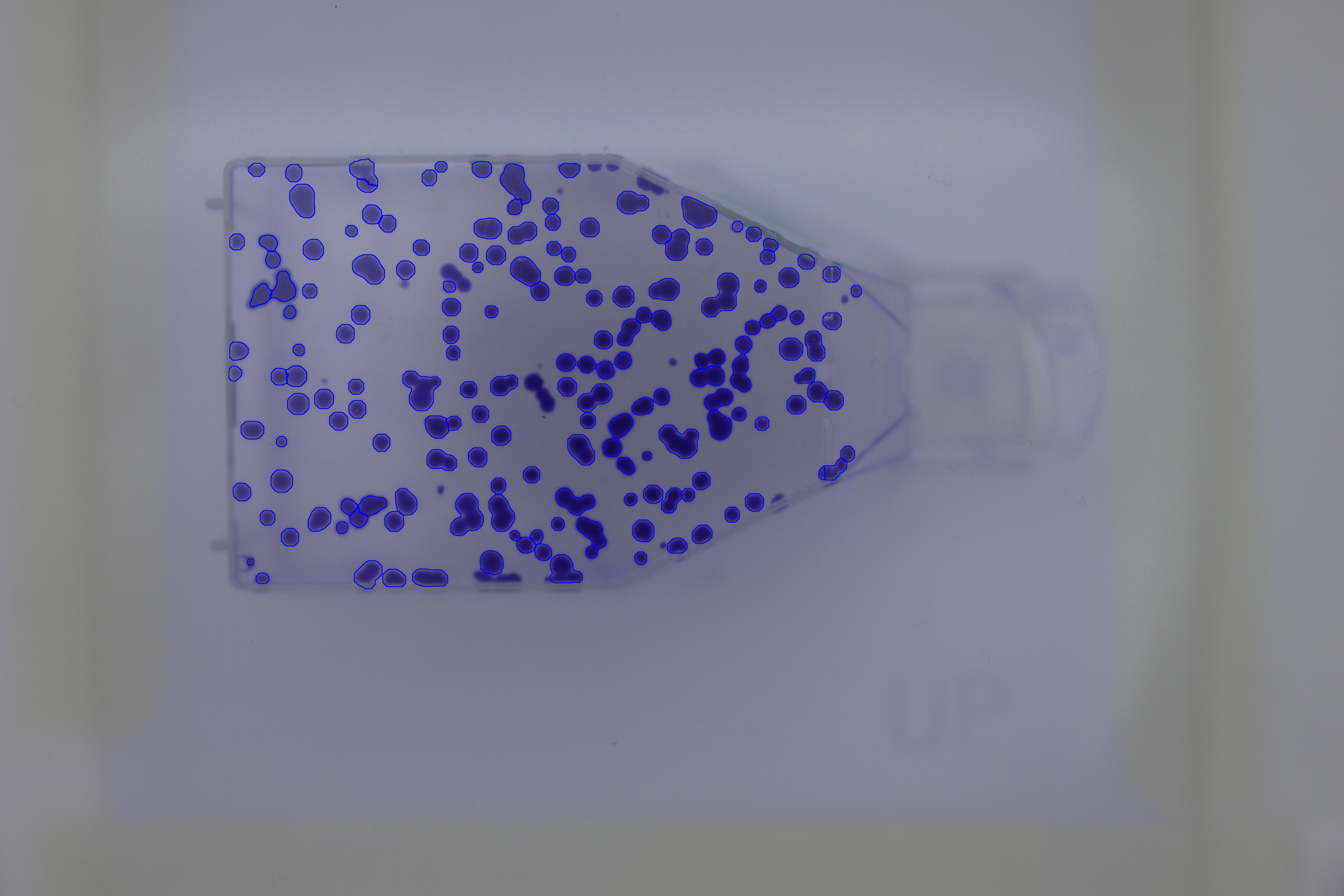

Supplement: S1 Comparison to others — (ZIP) [file pone.0205823.s007.zip › S1 Comparison to others/AutoCellSeg/171214 V79 Flask/12_seg.jpg]

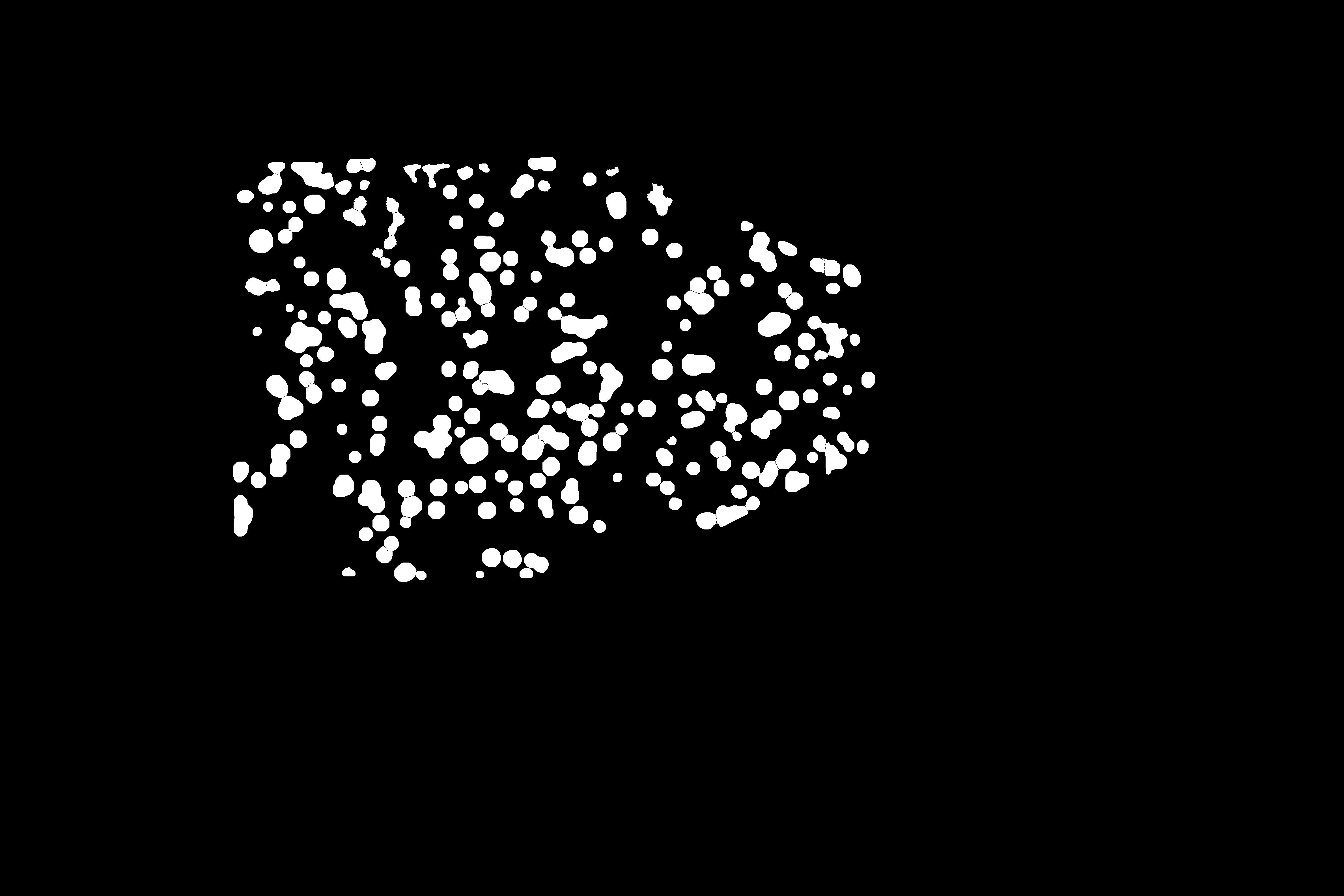

Supplement: S1 Comparison to others — (ZIP) [file pone.0205823.s007.zip › S1 Comparison to others/AutoCellSeg/171214 V79 Flask/13_mask.jpg]

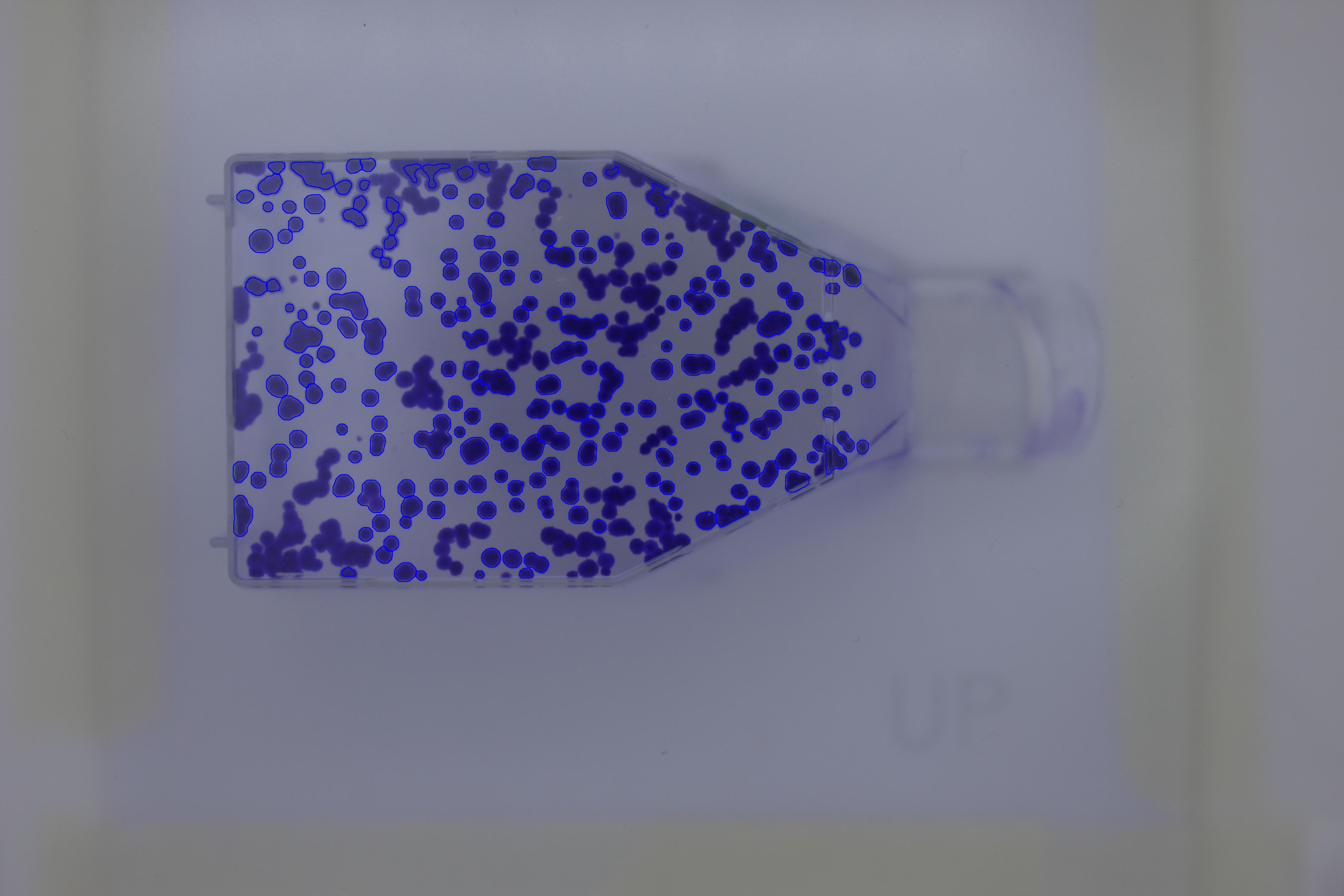

Supplement: S1 Comparison to others — (ZIP) [file pone.0205823.s007.zip › S1 Comparison to others/AutoCellSeg/171214 V79 Flask/13_seg.jpg]
