# Supplementary figures and images for: Cell colony counter called CoCoNut (part 5 of 5)
Source: PLoS One. 2018 Nov 7;13(11):e0205823. doi: 10.1371/journal.pone.0205823 (PMC6221277; doi:10.1371/journal.pone.0205823)

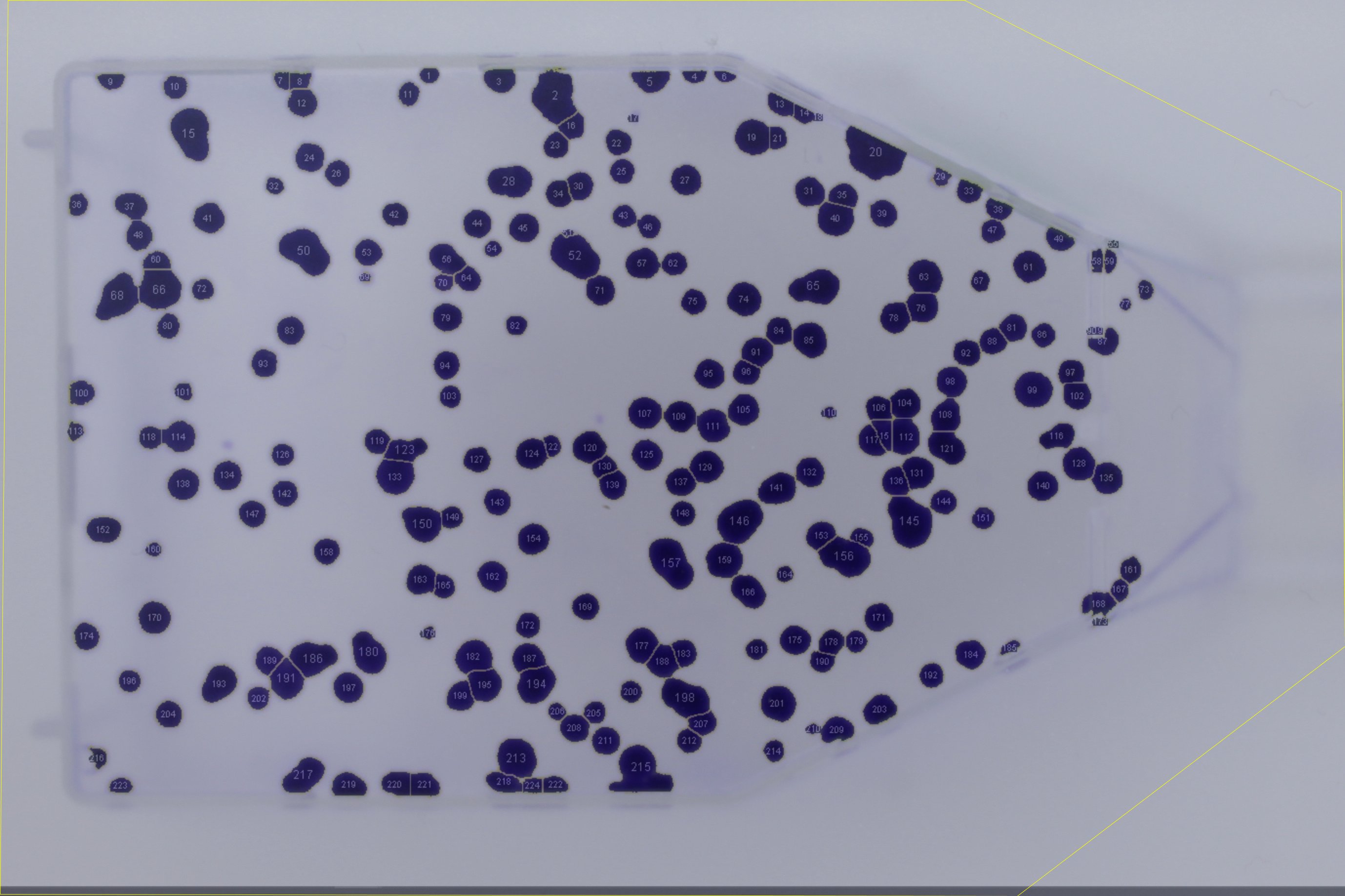

Supplement: S1 Comparison to others — (ZIP) [file pone.0205823.s007.zip › S1 Comparison to others/CAI/171214 V79 Flask/12 Results.jpg]

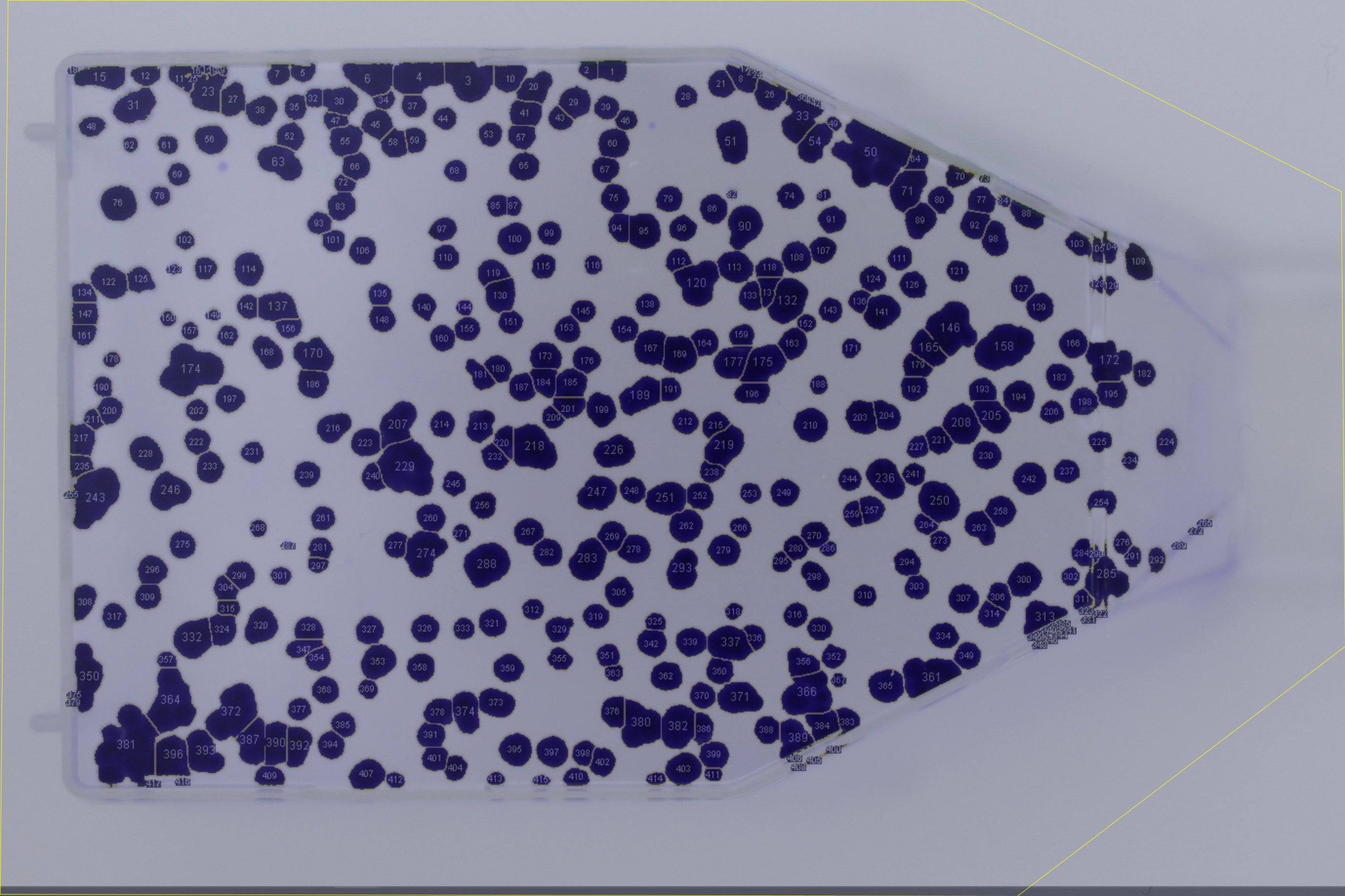

Supplement: S1 Comparison to others — (ZIP) [file pone.0205823.s007.zip › S1 Comparison to others/CAI/171214 V79 Flask/13 Results.jpg]

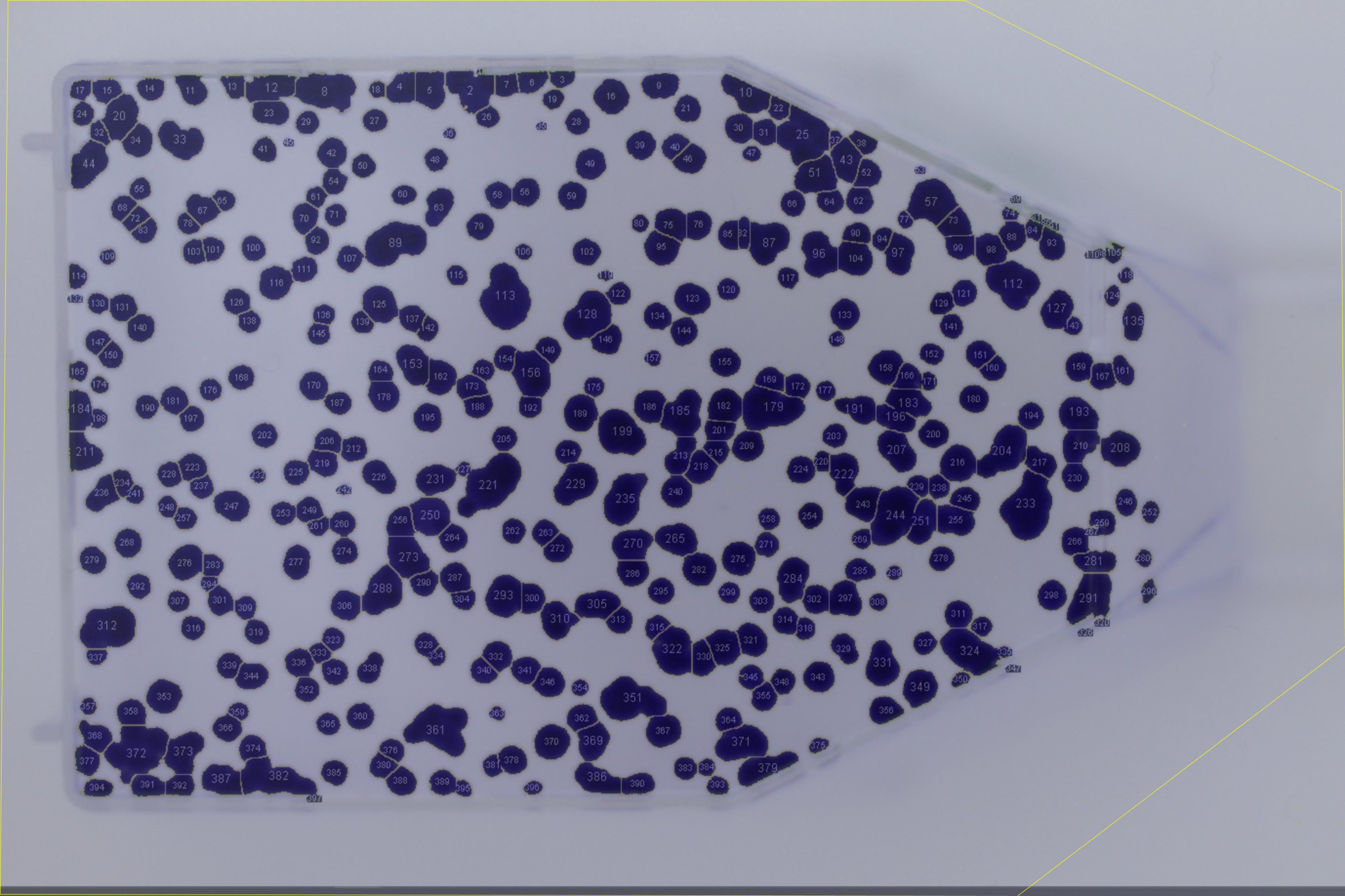

Supplement: S1 Comparison to others — (ZIP) [file pone.0205823.s007.zip › S1 Comparison to others/CAI/171214 V79 Flask/14 Results.jpg]

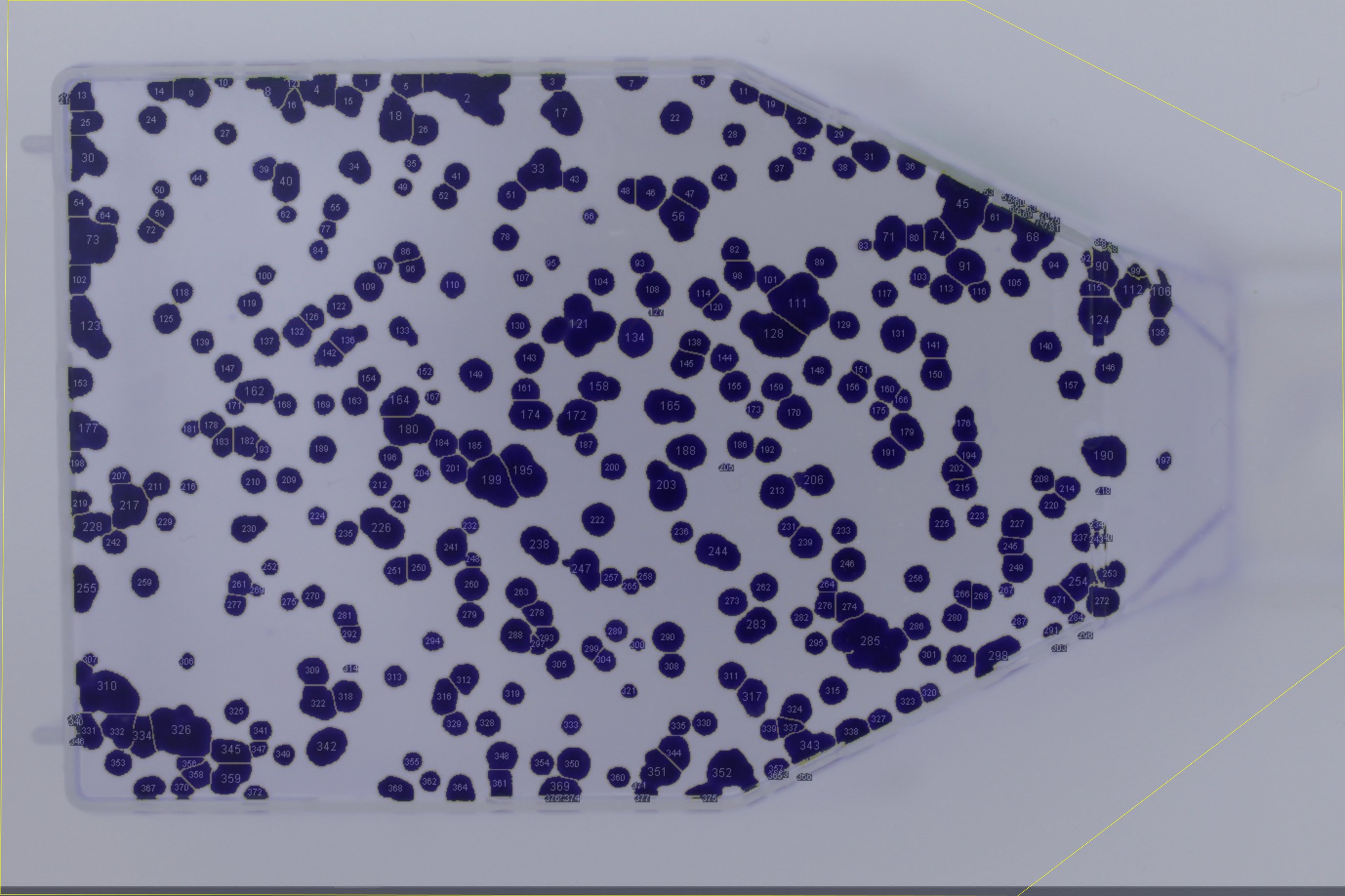

Supplement: S1 Comparison to others — (ZIP) [file pone.0205823.s007.zip › S1 Comparison to others/CAI/171214 V79 Flask/15 Results.jpg]

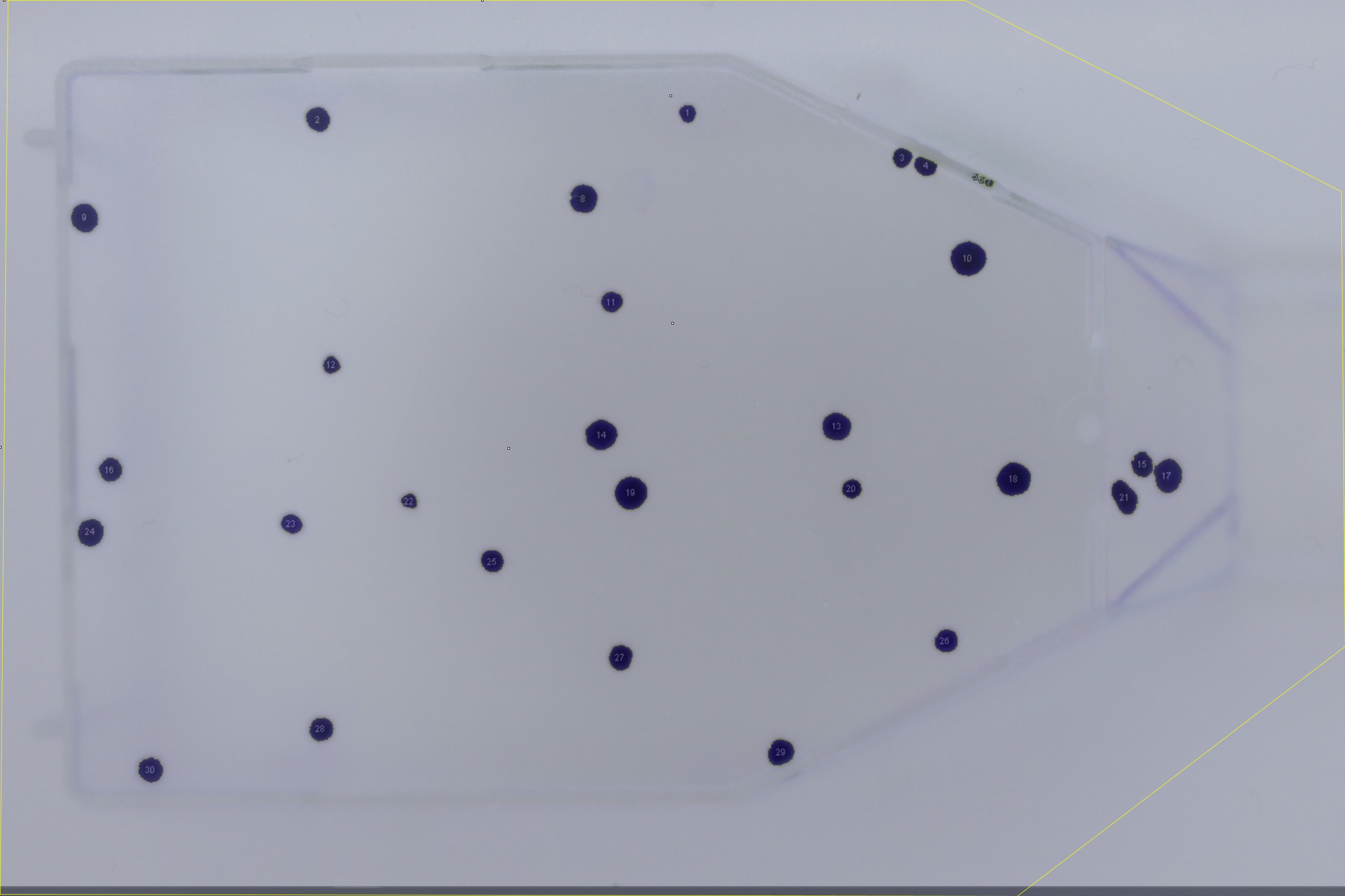

Supplement: S1 Comparison to others — (ZIP) [file pone.0205823.s007.zip › S1 Comparison to others/CAI/171214 V79 Flask/2 Results.jpg]

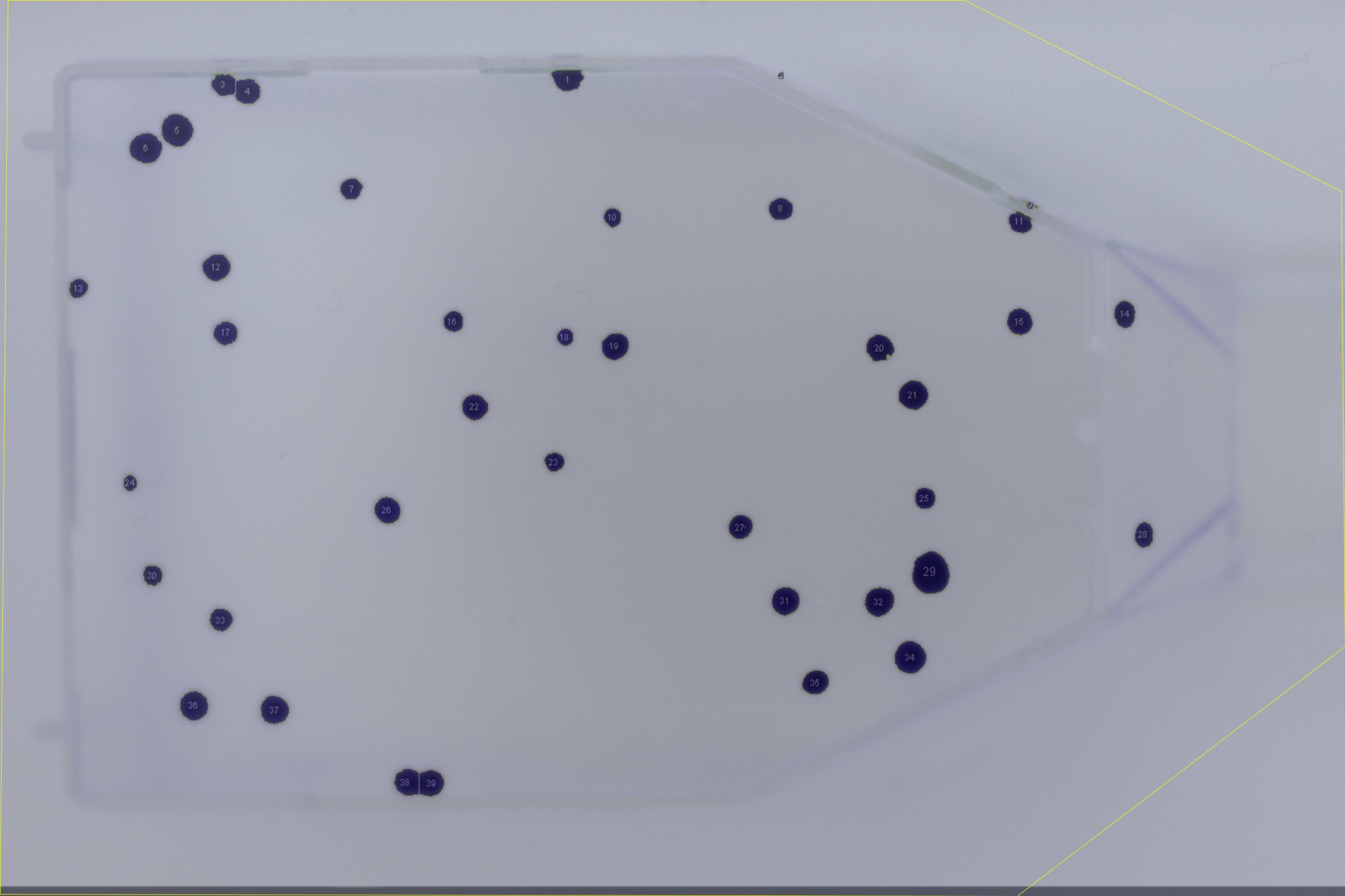

Supplement: S1 Comparison to others — (ZIP) [file pone.0205823.s007.zip › S1 Comparison to others/CAI/171214 V79 Flask/3 Results.jpg]

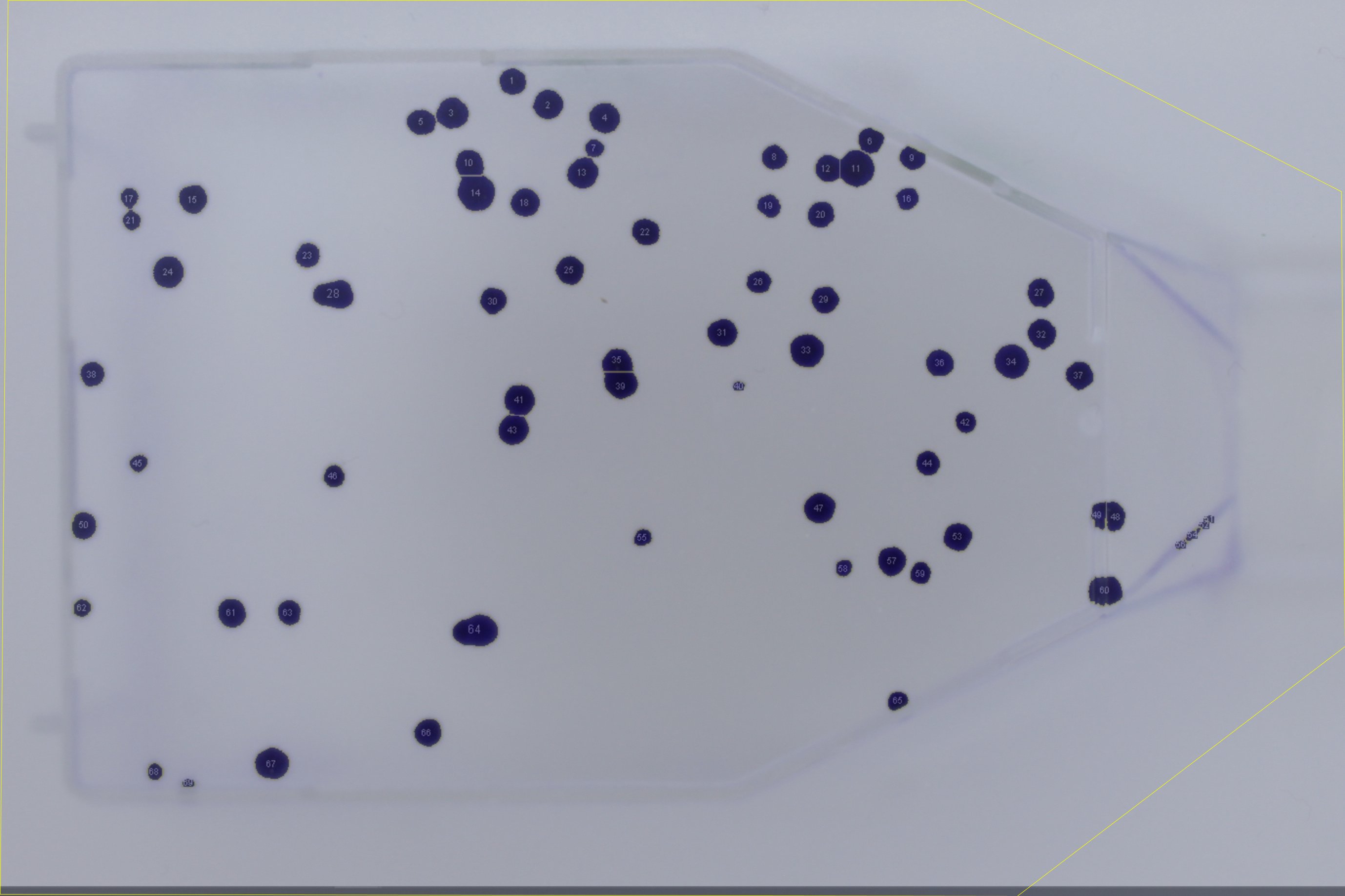

Supplement: S1 Comparison to others — (ZIP) [file pone.0205823.s007.zip › S1 Comparison to others/CAI/171214 V79 Flask/4 Results.jpg]

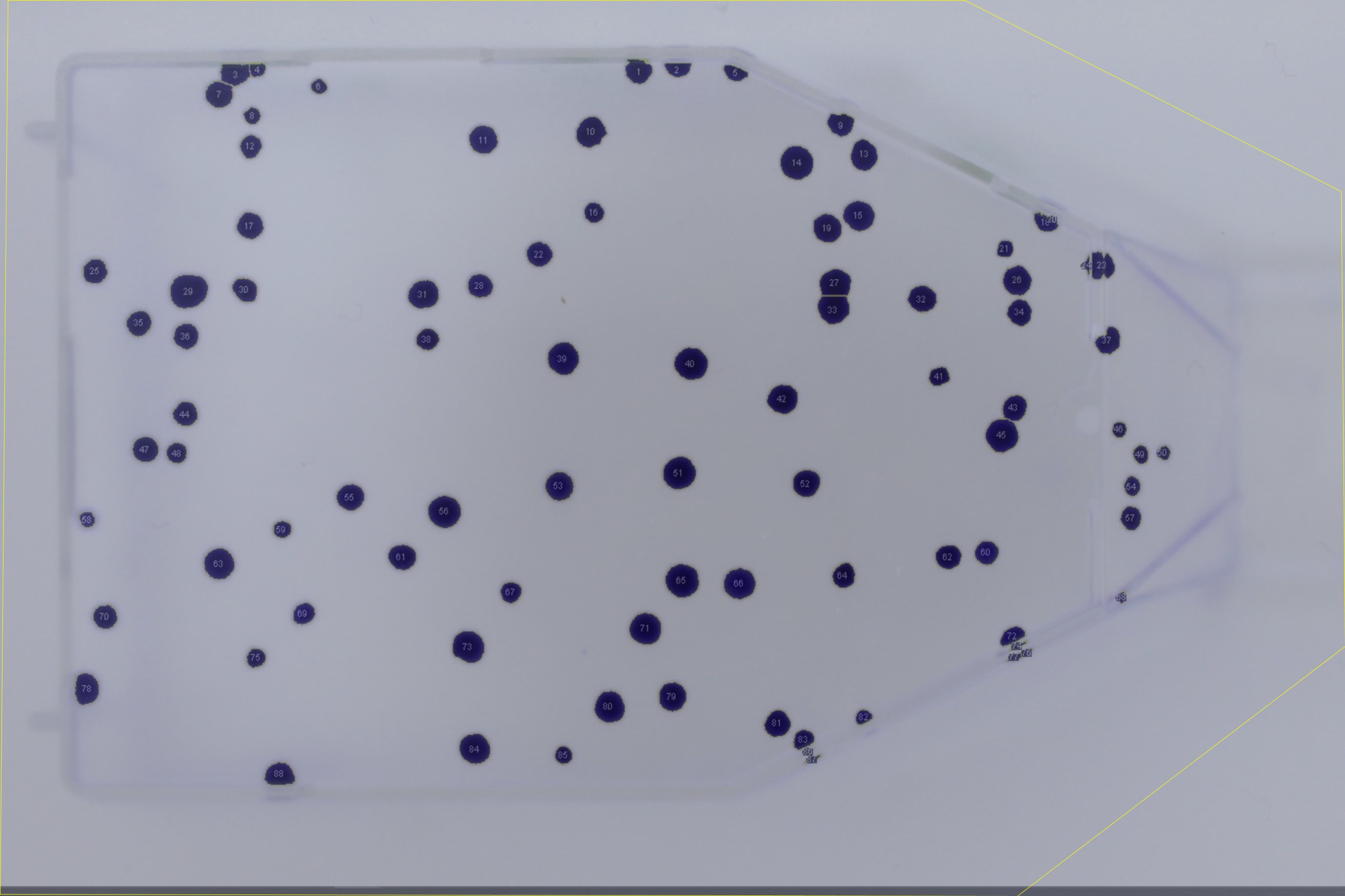

Supplement: S1 Comparison to others — (ZIP) [file pone.0205823.s007.zip › S1 Comparison to others/CAI/171214 V79 Flask/5 Results.jpg]

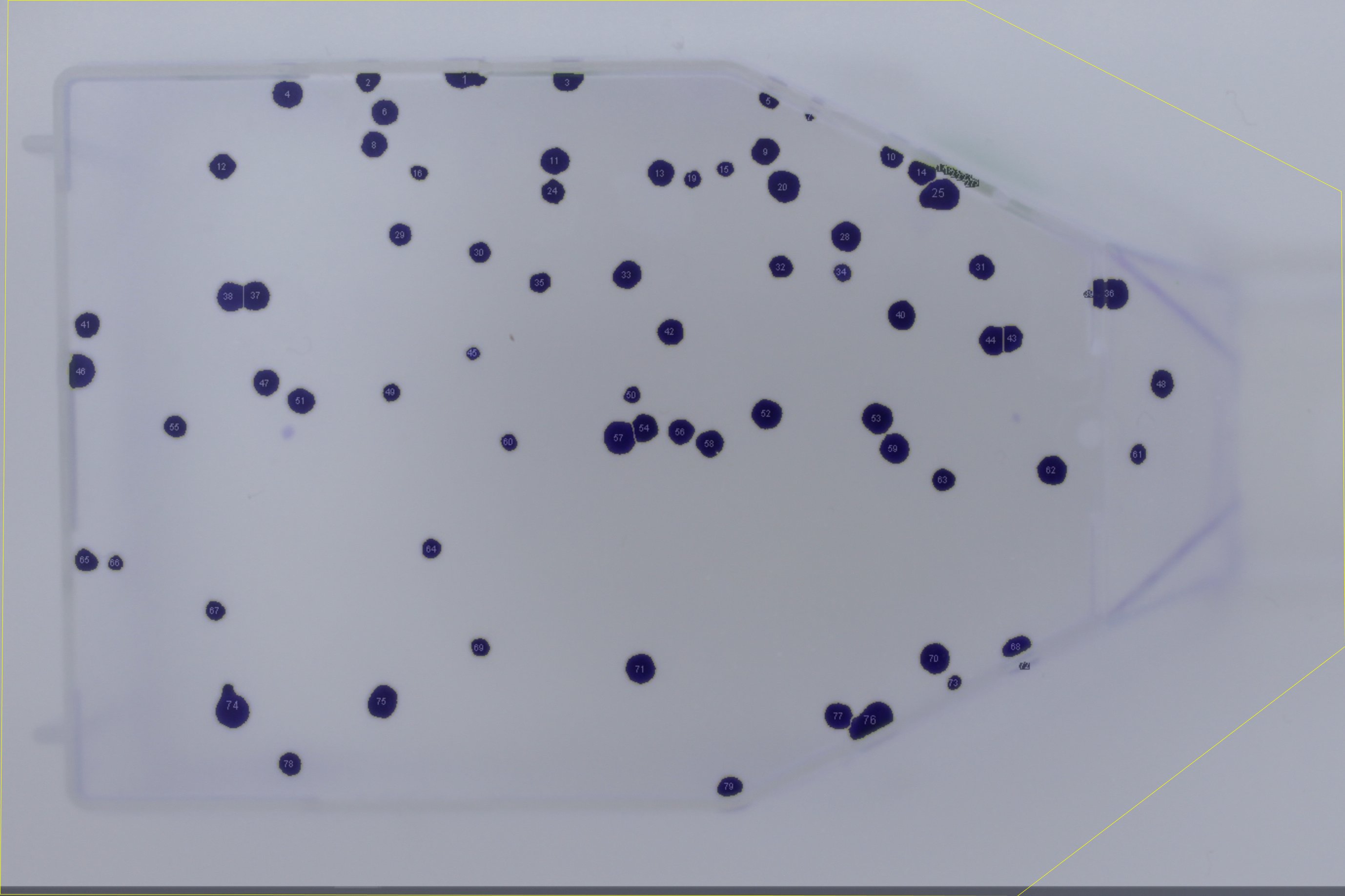

Supplement: S1 Comparison to others — (ZIP) [file pone.0205823.s007.zip › S1 Comparison to others/CAI/171214 V79 Flask/6 Results.jpg]

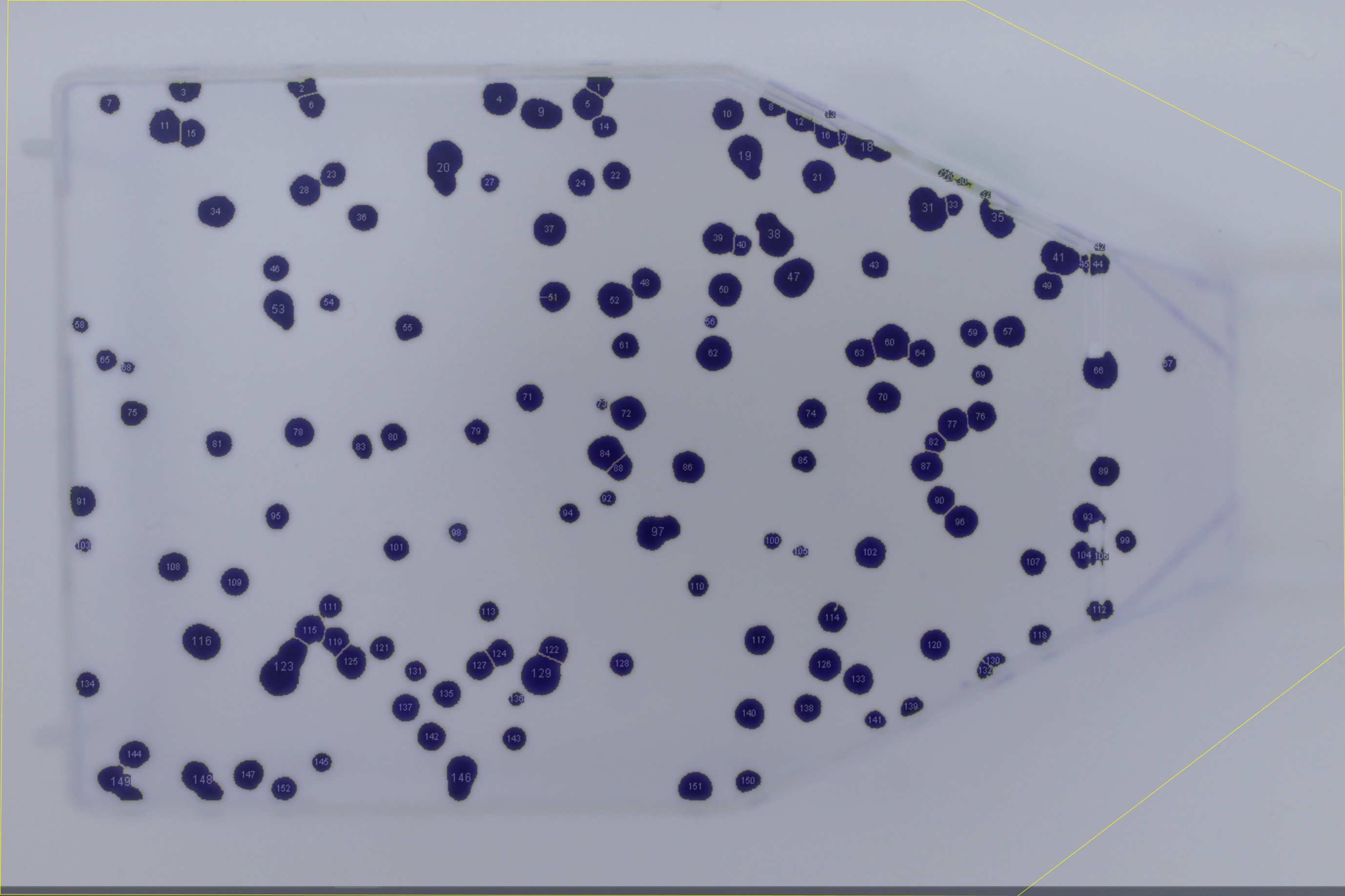

Supplement: S1 Comparison to others — (ZIP) [file pone.0205823.s007.zip › S1 Comparison to others/CAI/171214 V79 Flask/7 Results.jpg]

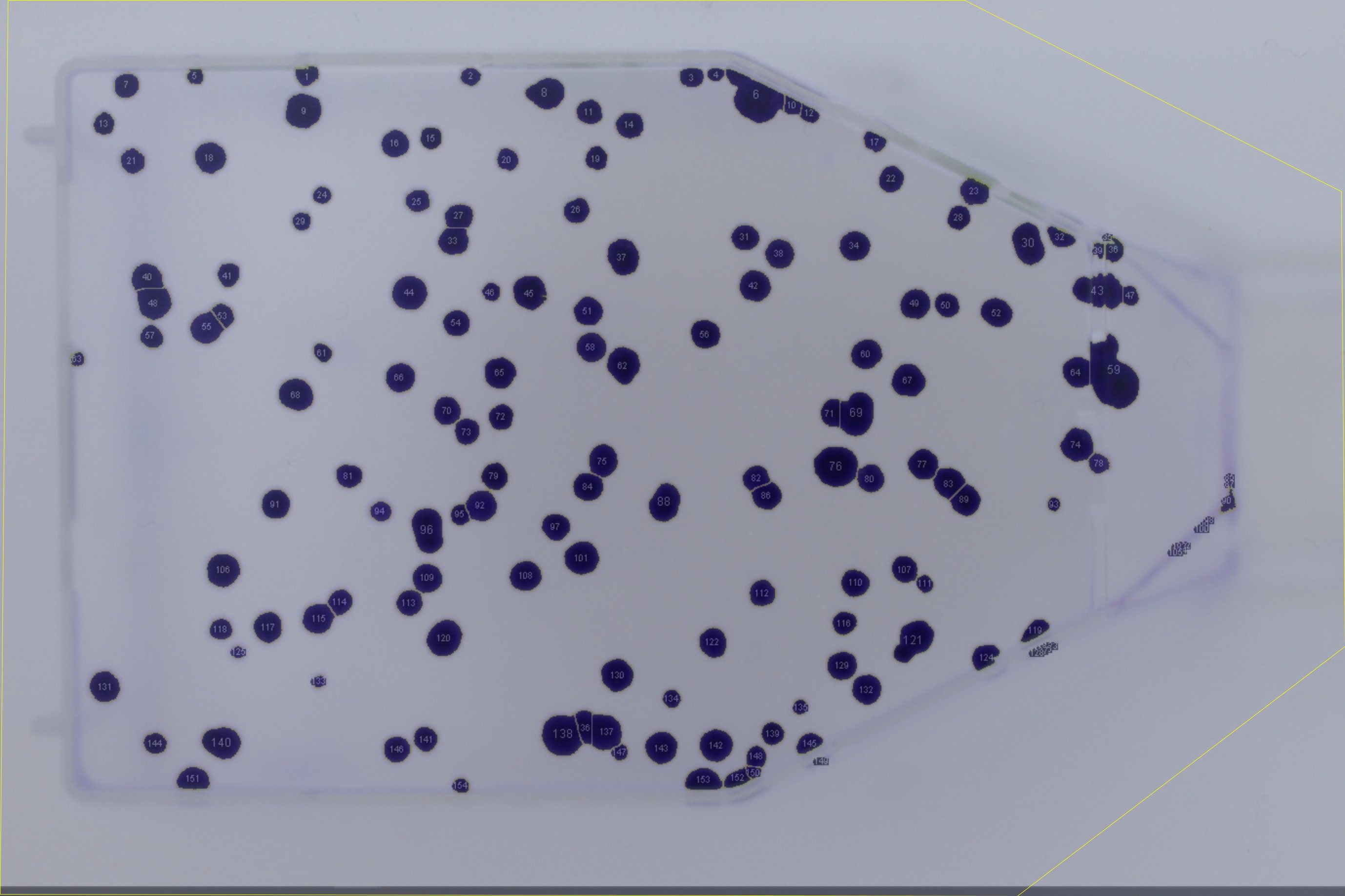

Supplement: S1 Comparison to others — (ZIP) [file pone.0205823.s007.zip › S1 Comparison to others/CAI/171214 V79 Flask/8 Results.jpg]

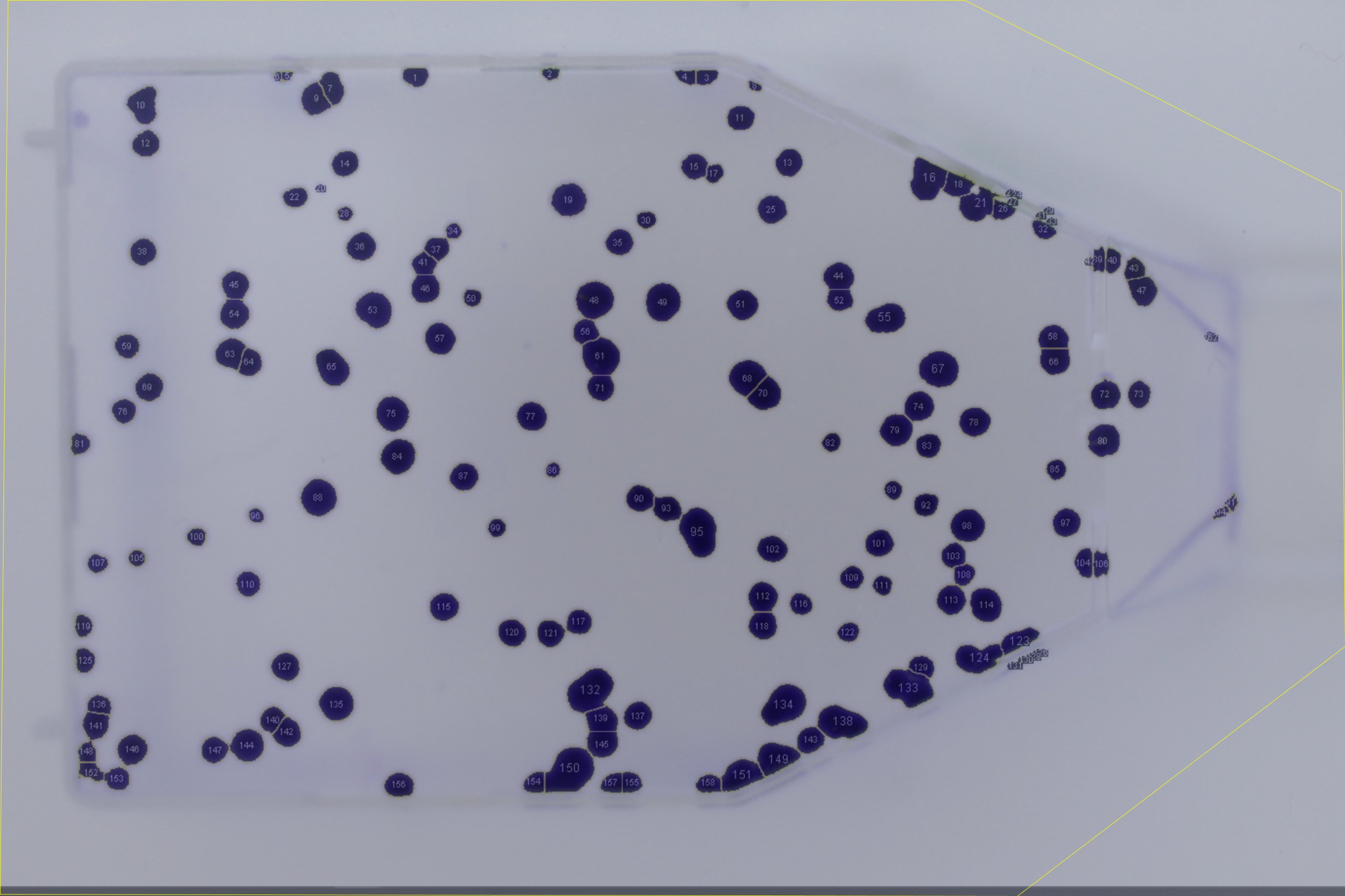

Supplement: S1 Comparison to others — (ZIP) [file pone.0205823.s007.zip › S1 Comparison to others/CAI/171214 V79 Flask/9 Results.jpg]

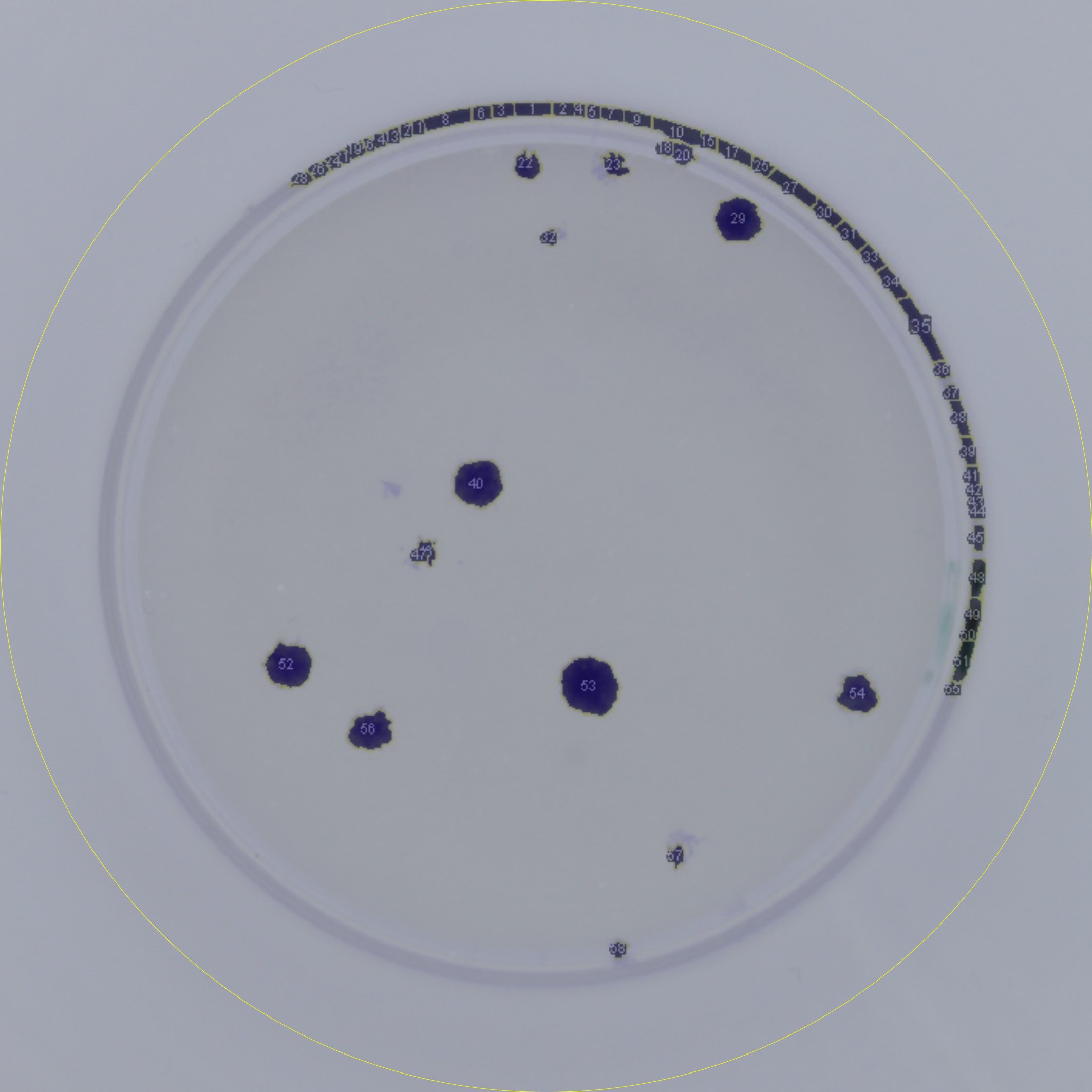

Supplement: S1 Comparison to others — (ZIP) [file pone.0205823.s007.zip › S1 Comparison to others/CAI/180501 HeLa Dish/1 Results.jpg]

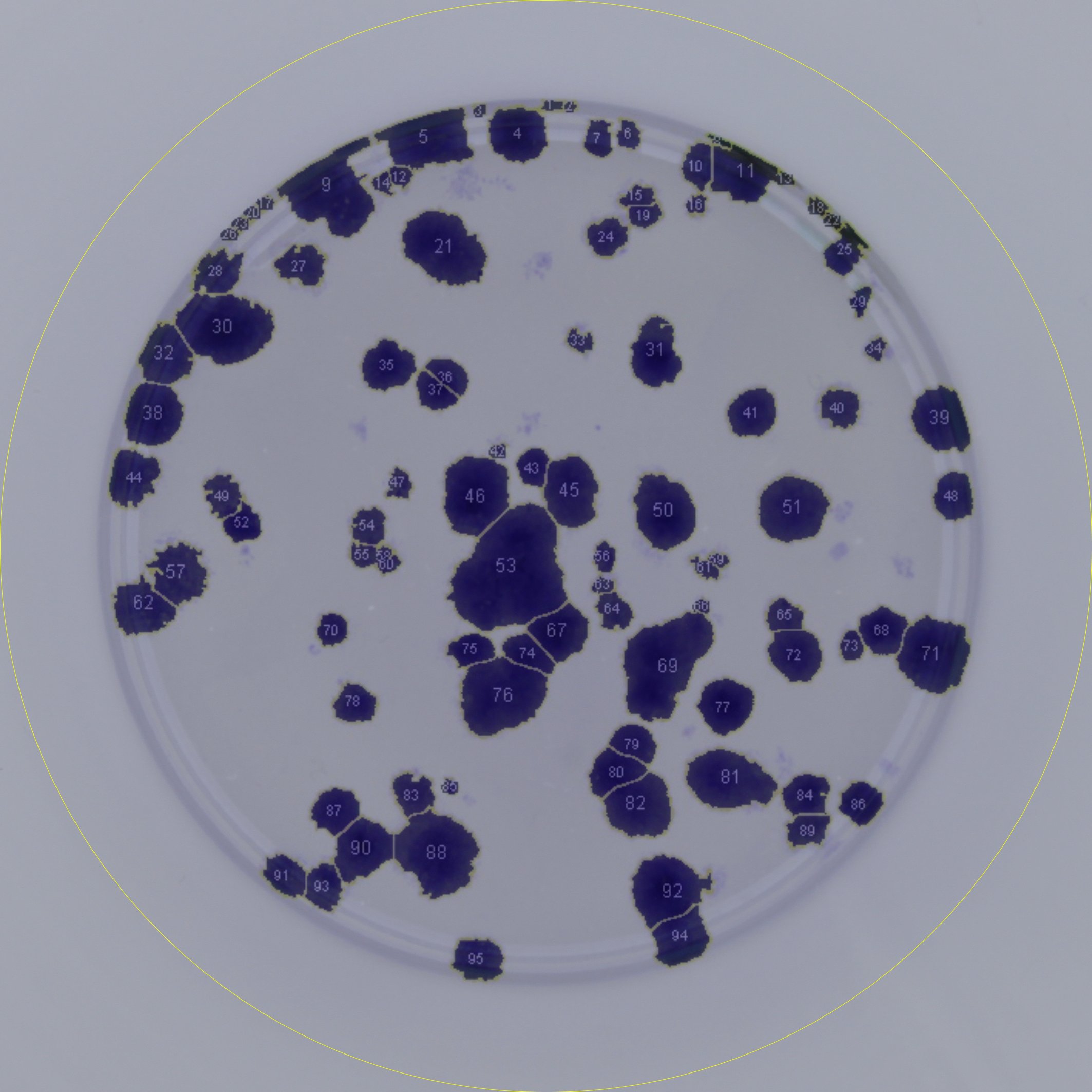

Supplement: S1 Comparison to others — (ZIP) [file pone.0205823.s007.zip › S1 Comparison to others/CAI/180501 HeLa Dish/10 Results.jpg]

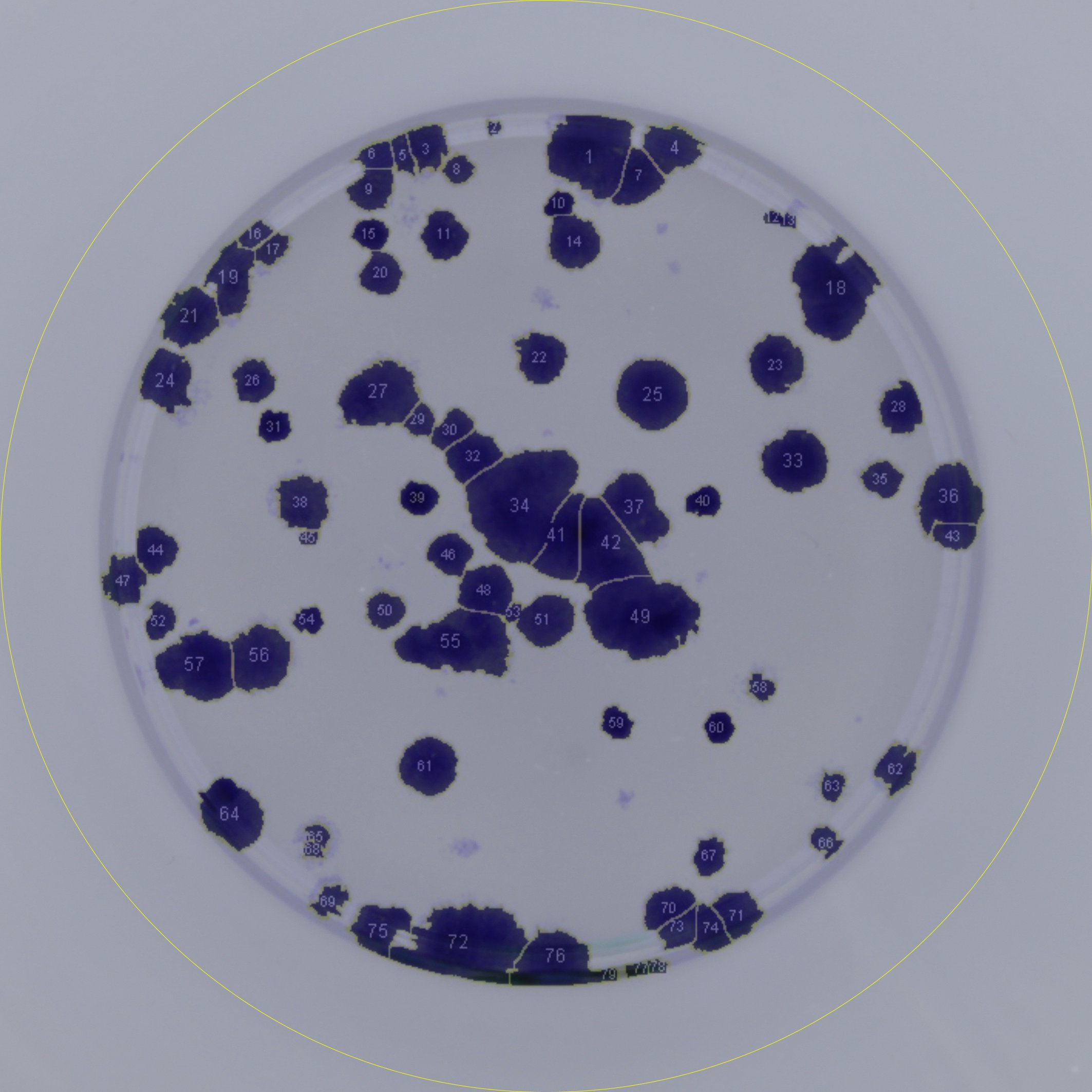

Supplement: S1 Comparison to others — (ZIP) [file pone.0205823.s007.zip › S1 Comparison to others/CAI/180501 HeLa Dish/11 Results.jpg]

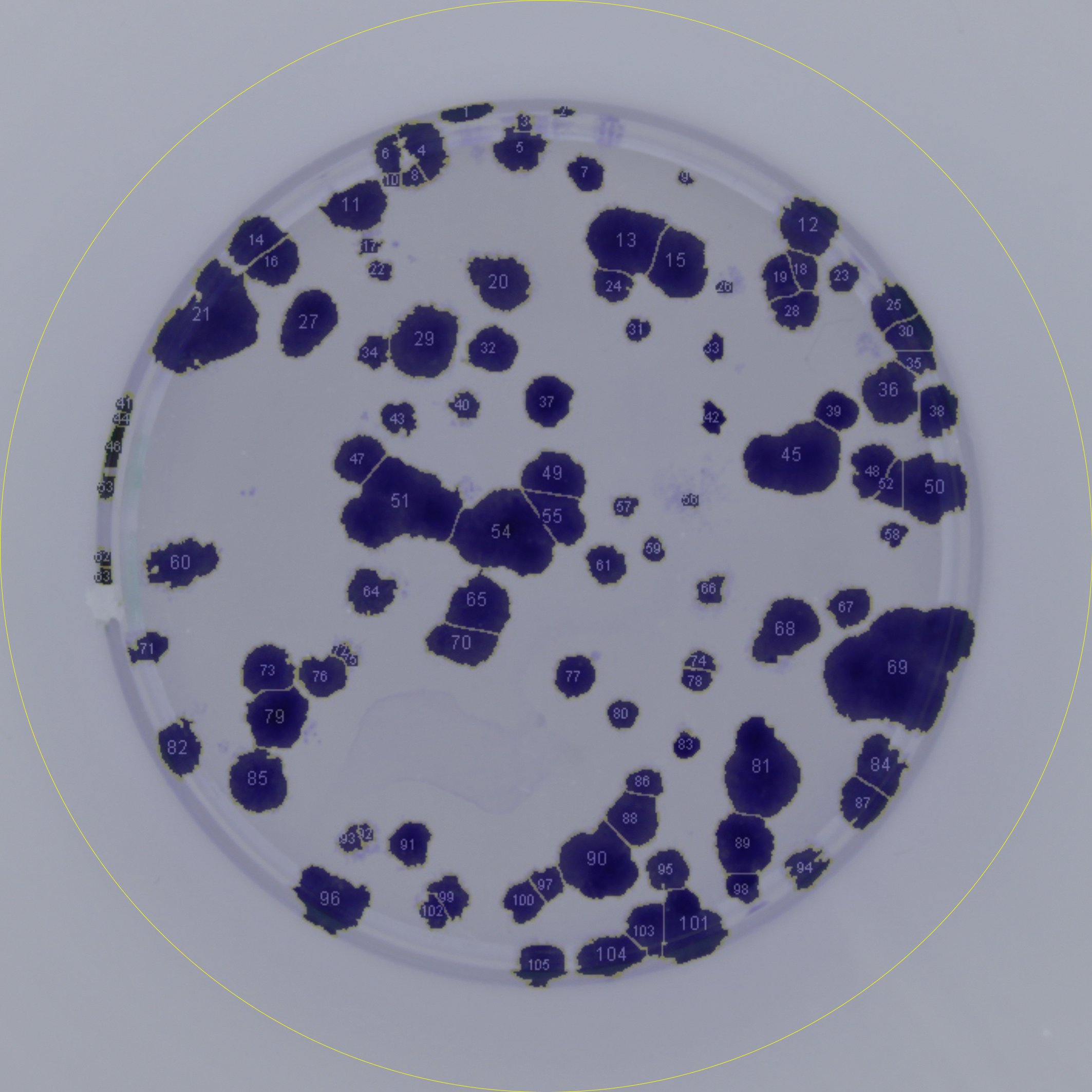

Supplement: S1 Comparison to others — (ZIP) [file pone.0205823.s007.zip › S1 Comparison to others/CAI/180501 HeLa Dish/12 Results.jpg]

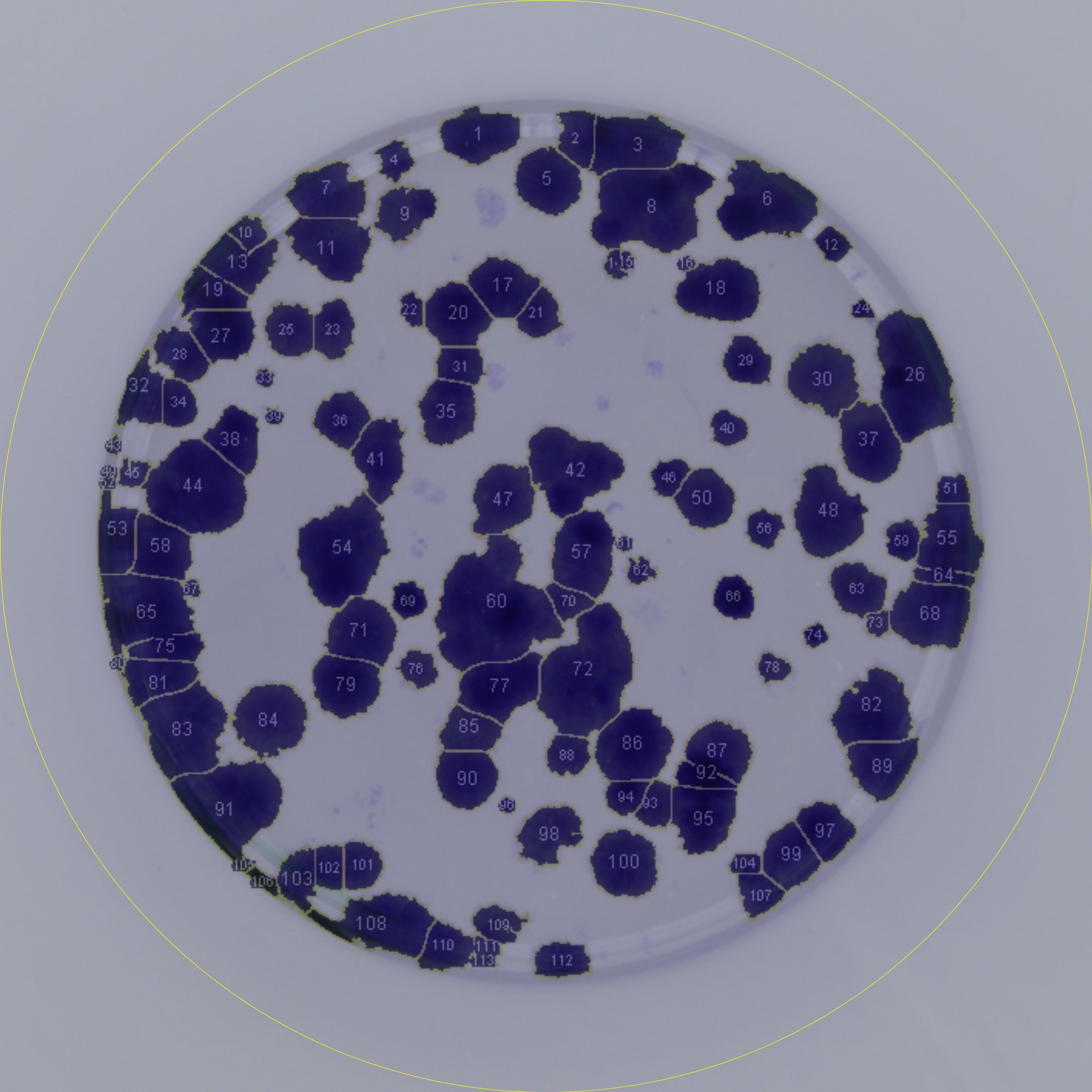

Supplement: S1 Comparison to others — (ZIP) [file pone.0205823.s007.zip › S1 Comparison to others/CAI/180501 HeLa Dish/13 Results.jpg]

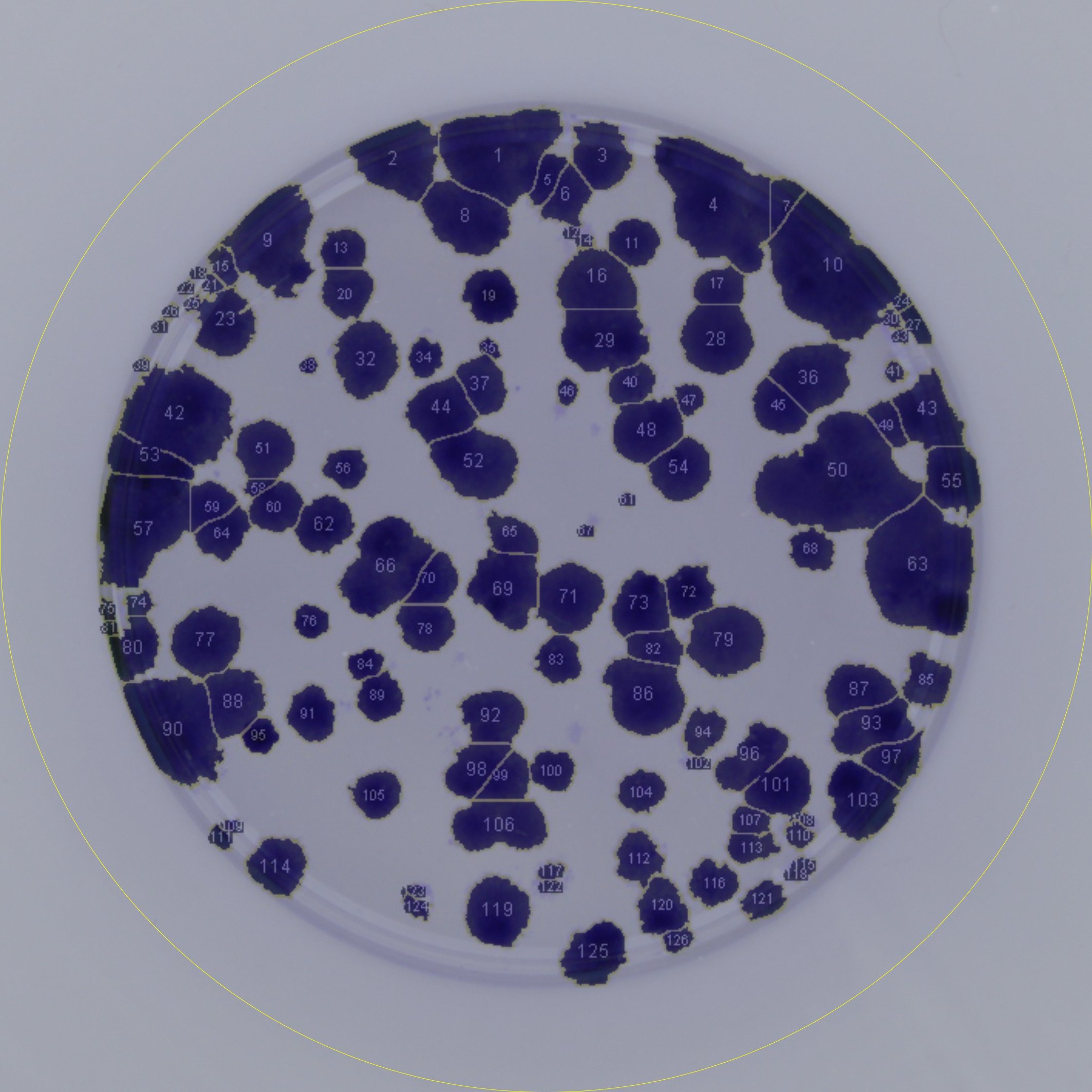

Supplement: S1 Comparison to others — (ZIP) [file pone.0205823.s007.zip › S1 Comparison to others/CAI/180501 HeLa Dish/14 Results.jpg]

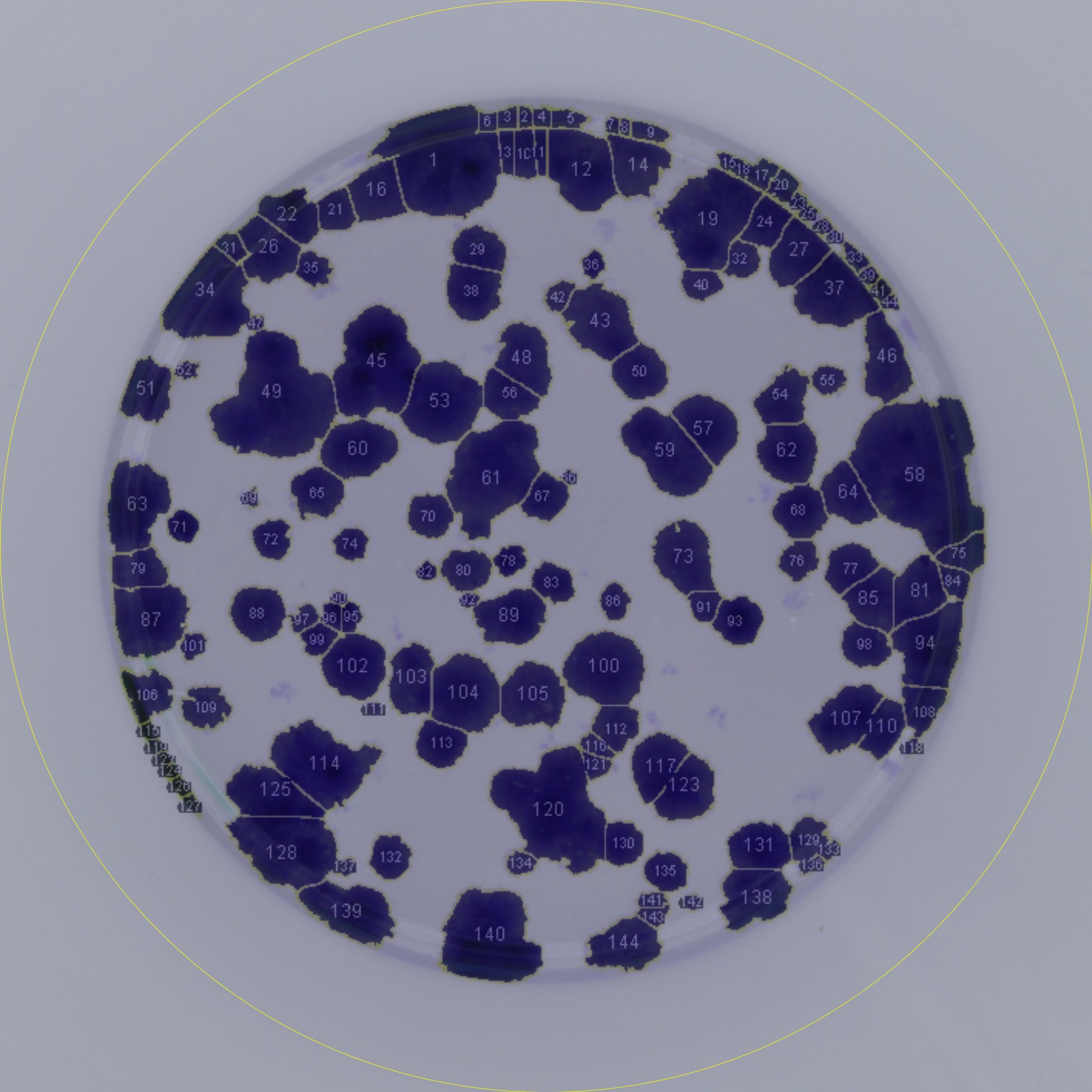

Supplement: S1 Comparison to others — (ZIP) [file pone.0205823.s007.zip › S1 Comparison to others/CAI/180501 HeLa Dish/15 Results.jpg]

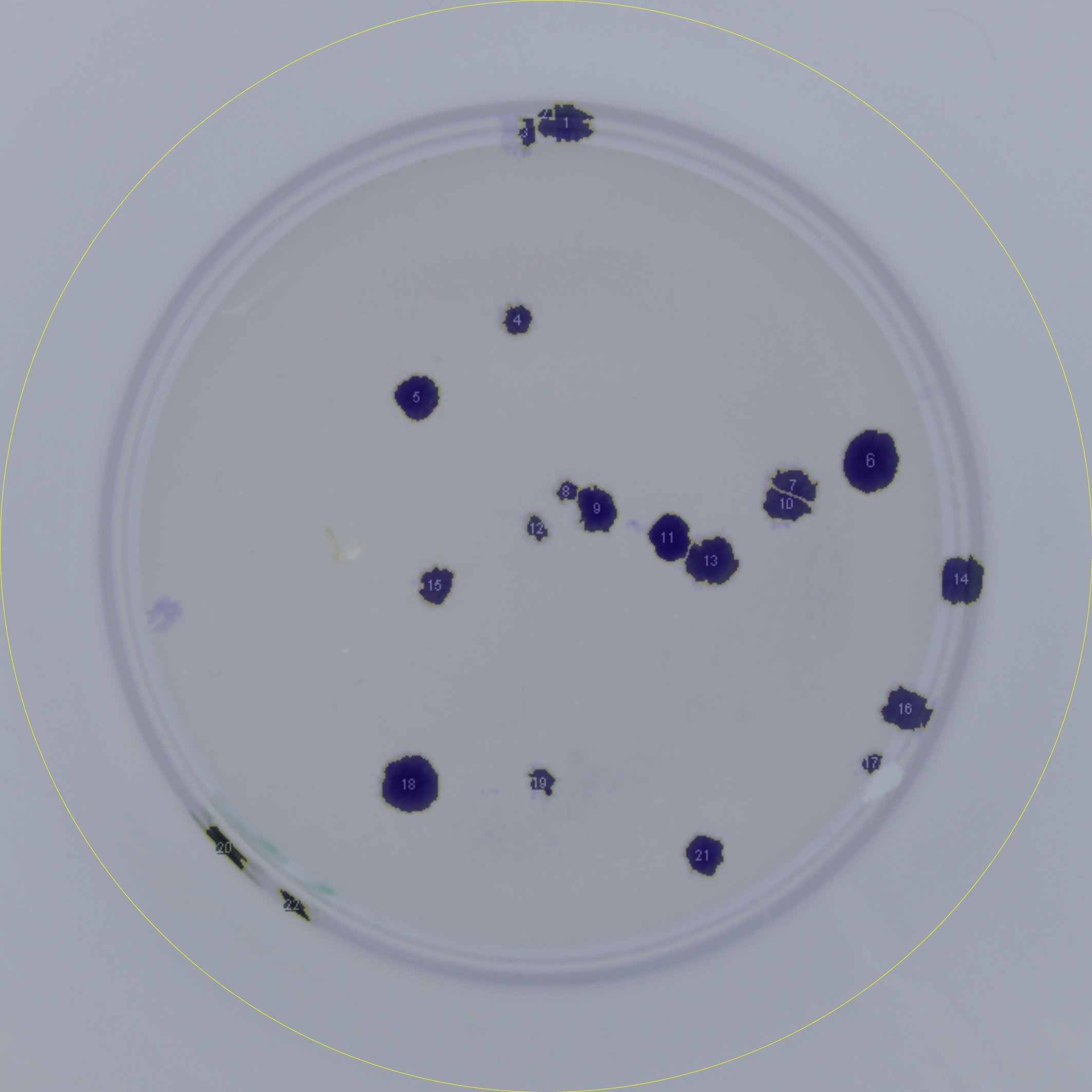

Supplement: S1 Comparison to others — (ZIP) [file pone.0205823.s007.zip › S1 Comparison to others/CAI/180501 HeLa Dish/2 Results.jpg]

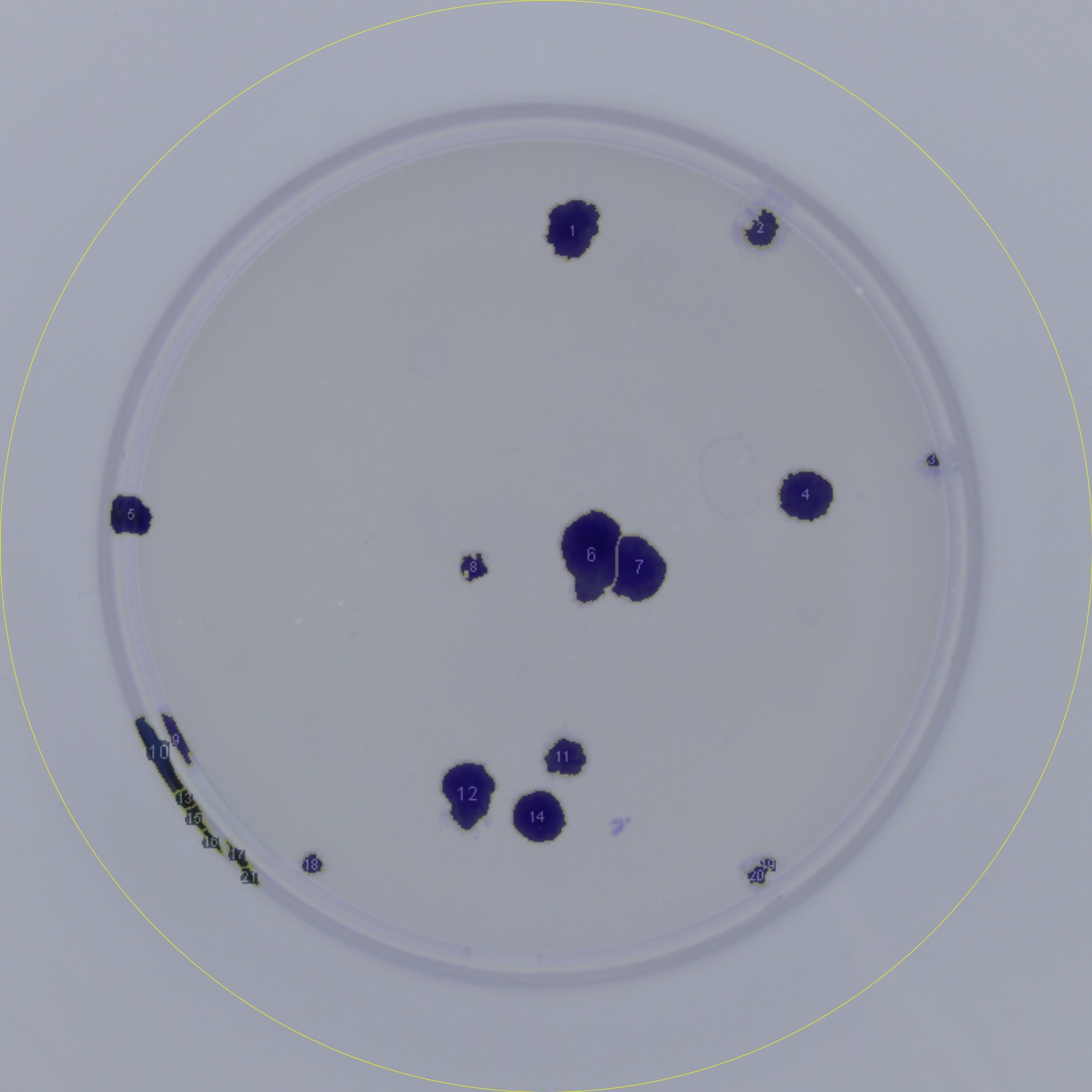

Supplement: S1 Comparison to others — (ZIP) [file pone.0205823.s007.zip › S1 Comparison to others/CAI/180501 HeLa Dish/3 Results.jpg]

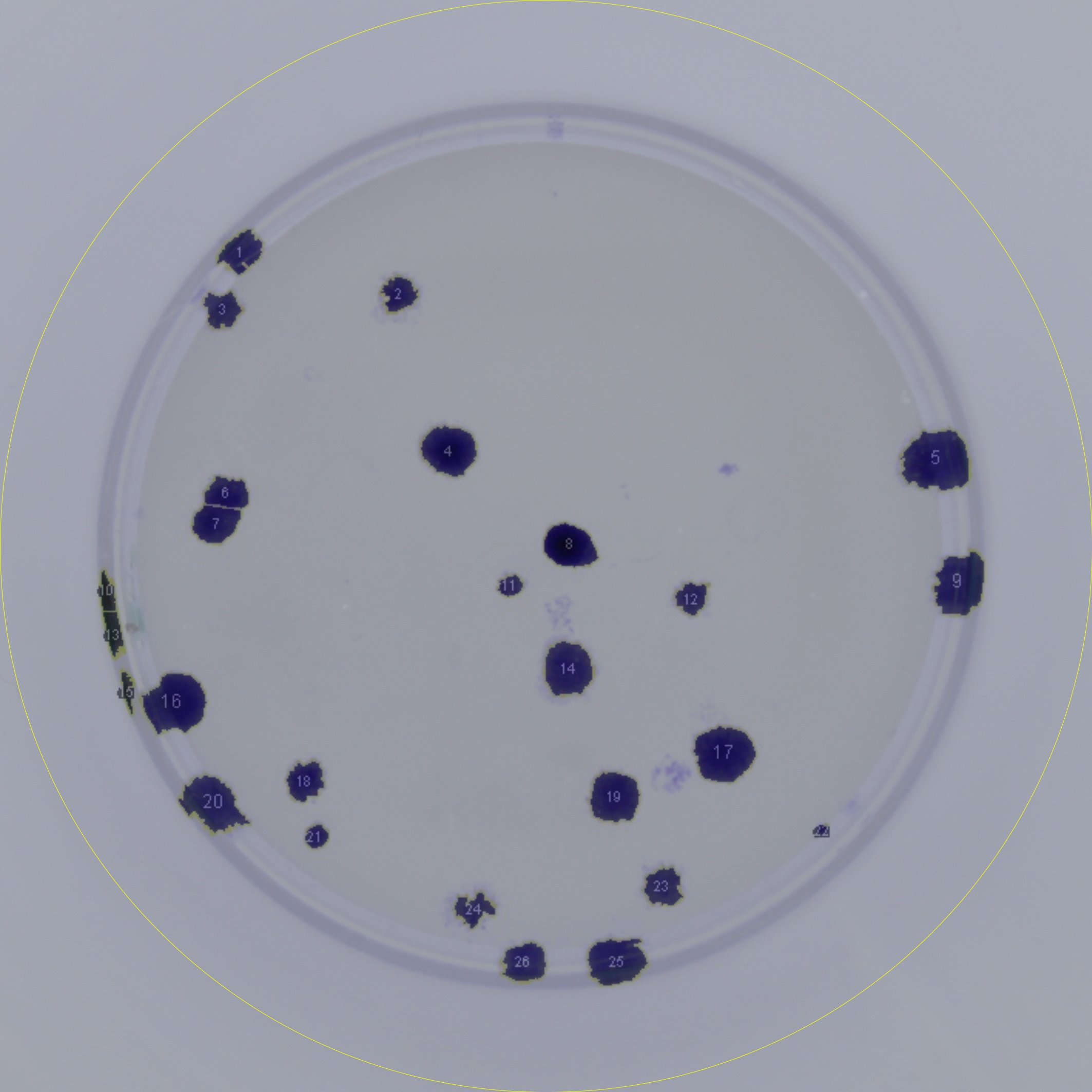

Supplement: S1 Comparison to others — (ZIP) [file pone.0205823.s007.zip › S1 Comparison to others/CAI/180501 HeLa Dish/4 Results.jpg]

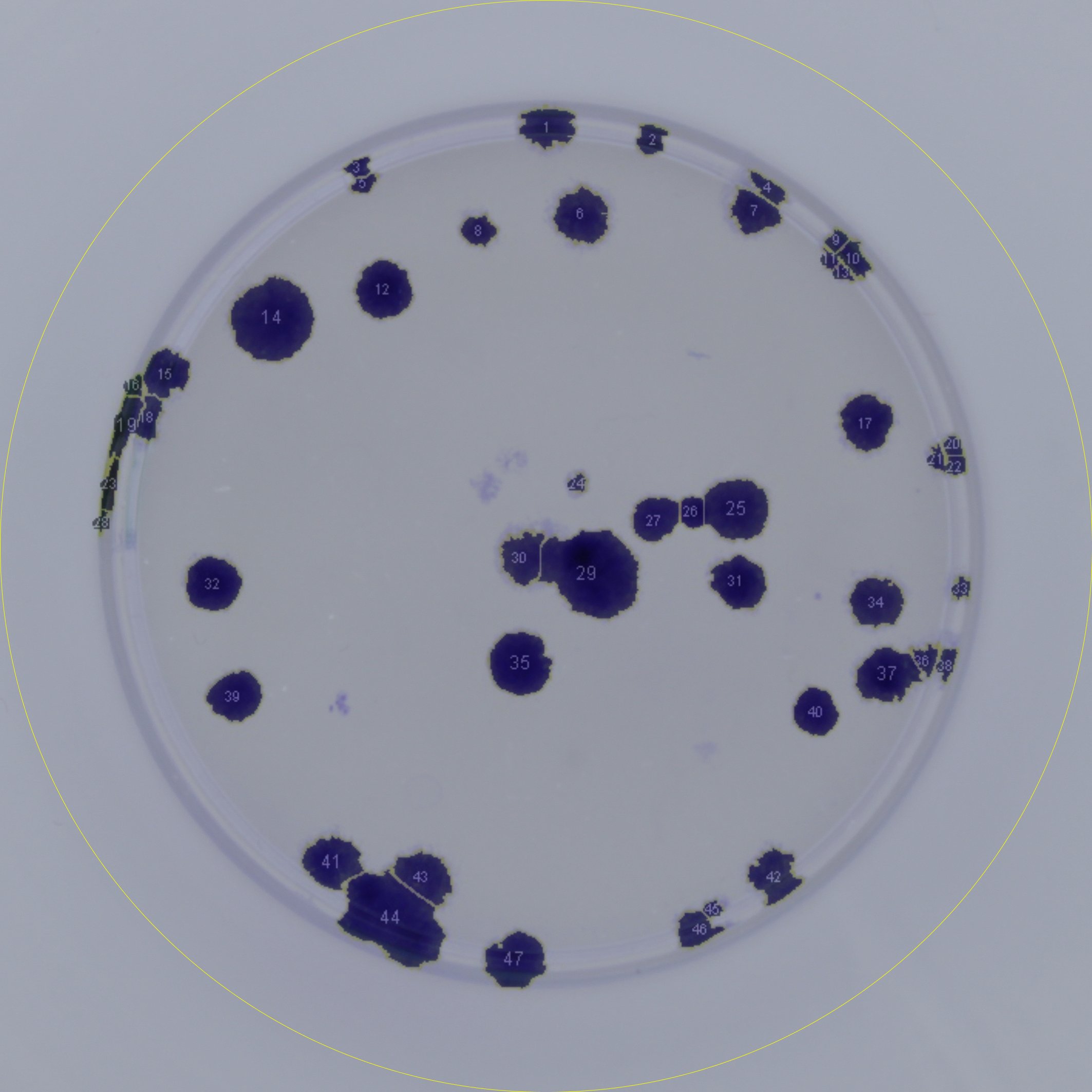

Supplement: S1 Comparison to others — (ZIP) [file pone.0205823.s007.zip › S1 Comparison to others/CAI/180501 HeLa Dish/5 Results.jpg]

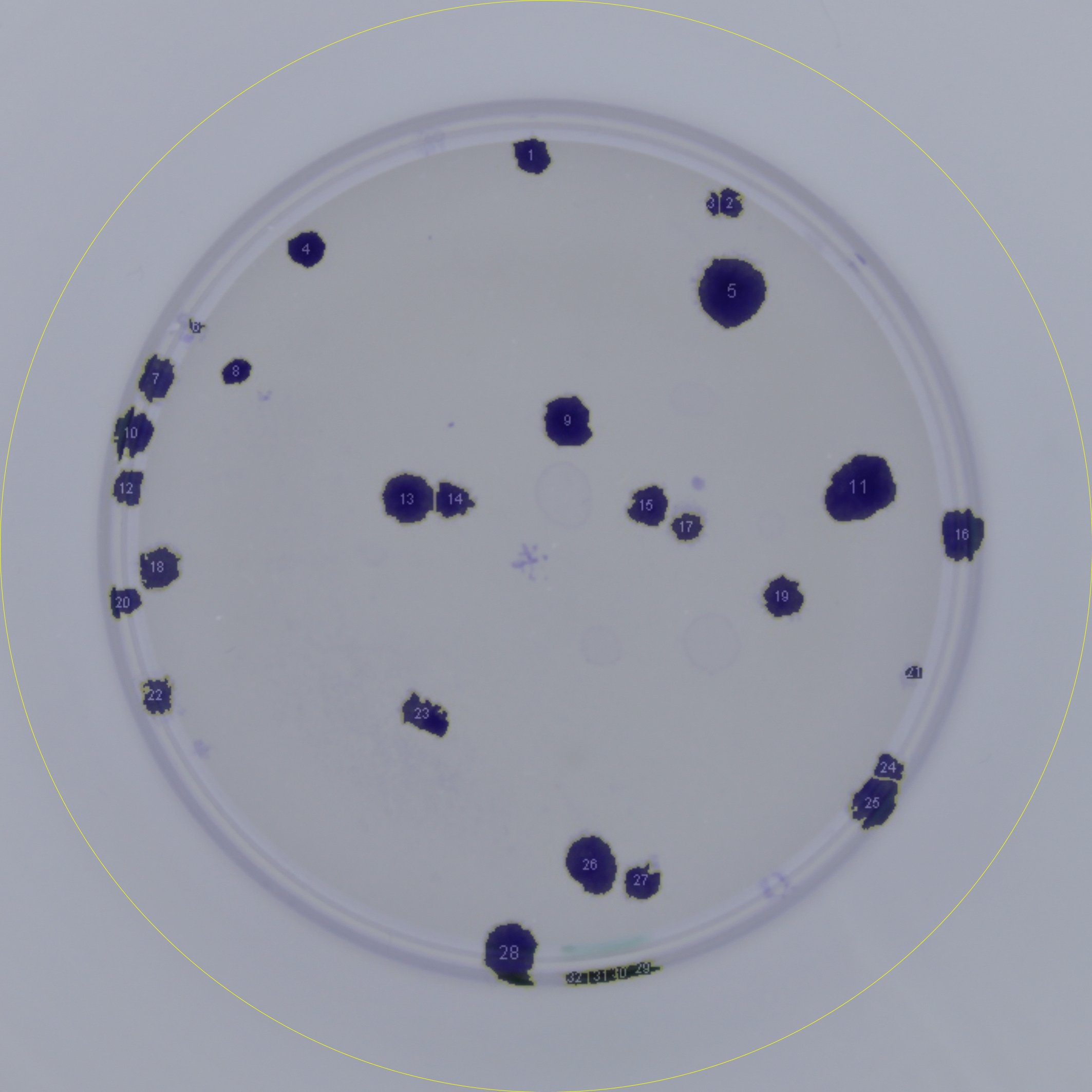

Supplement: S1 Comparison to others — (ZIP) [file pone.0205823.s007.zip › S1 Comparison to others/CAI/180501 HeLa Dish/6 Results.jpg]

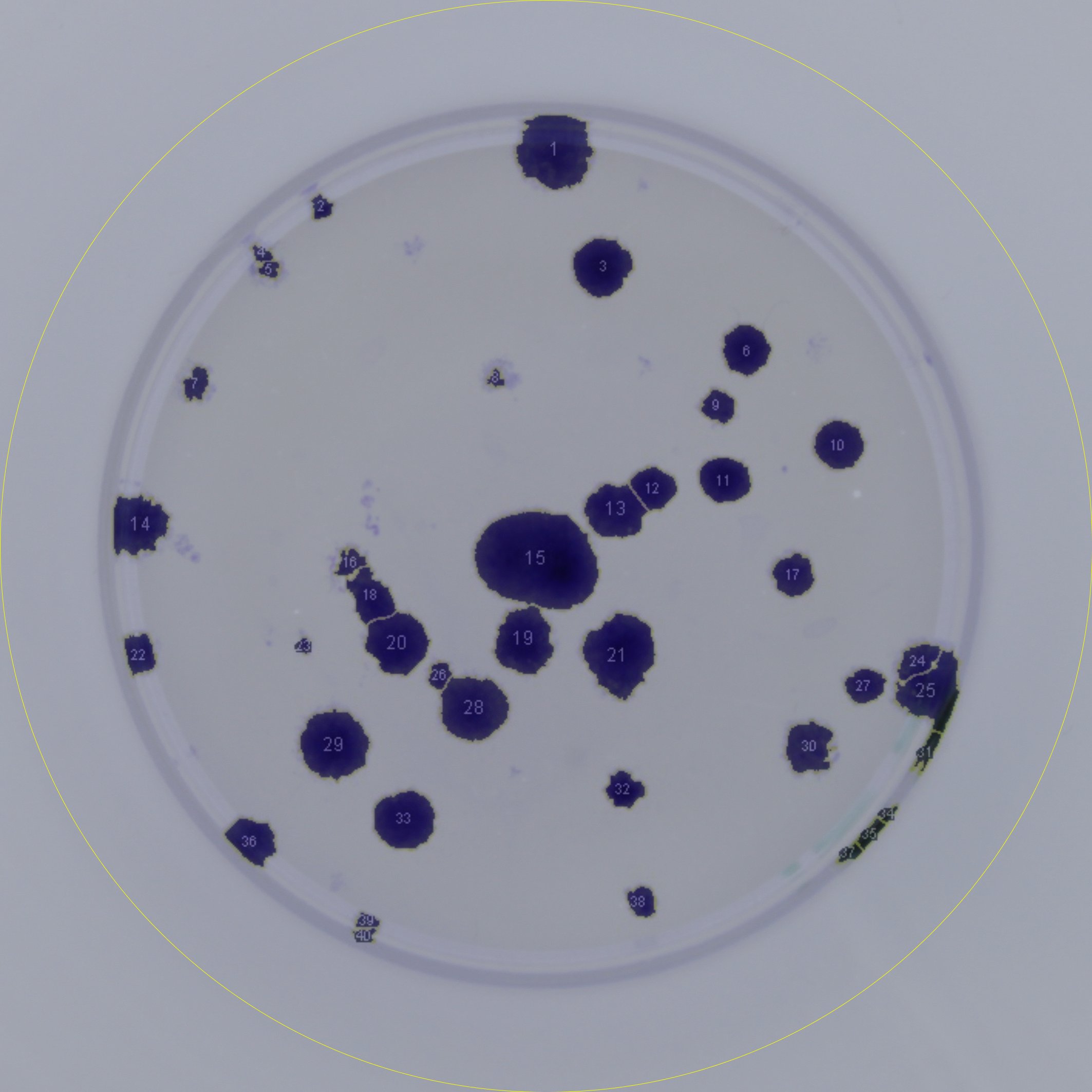

Supplement: S1 Comparison to others — (ZIP) [file pone.0205823.s007.zip › S1 Comparison to others/CAI/180501 HeLa Dish/7 Results.jpg]

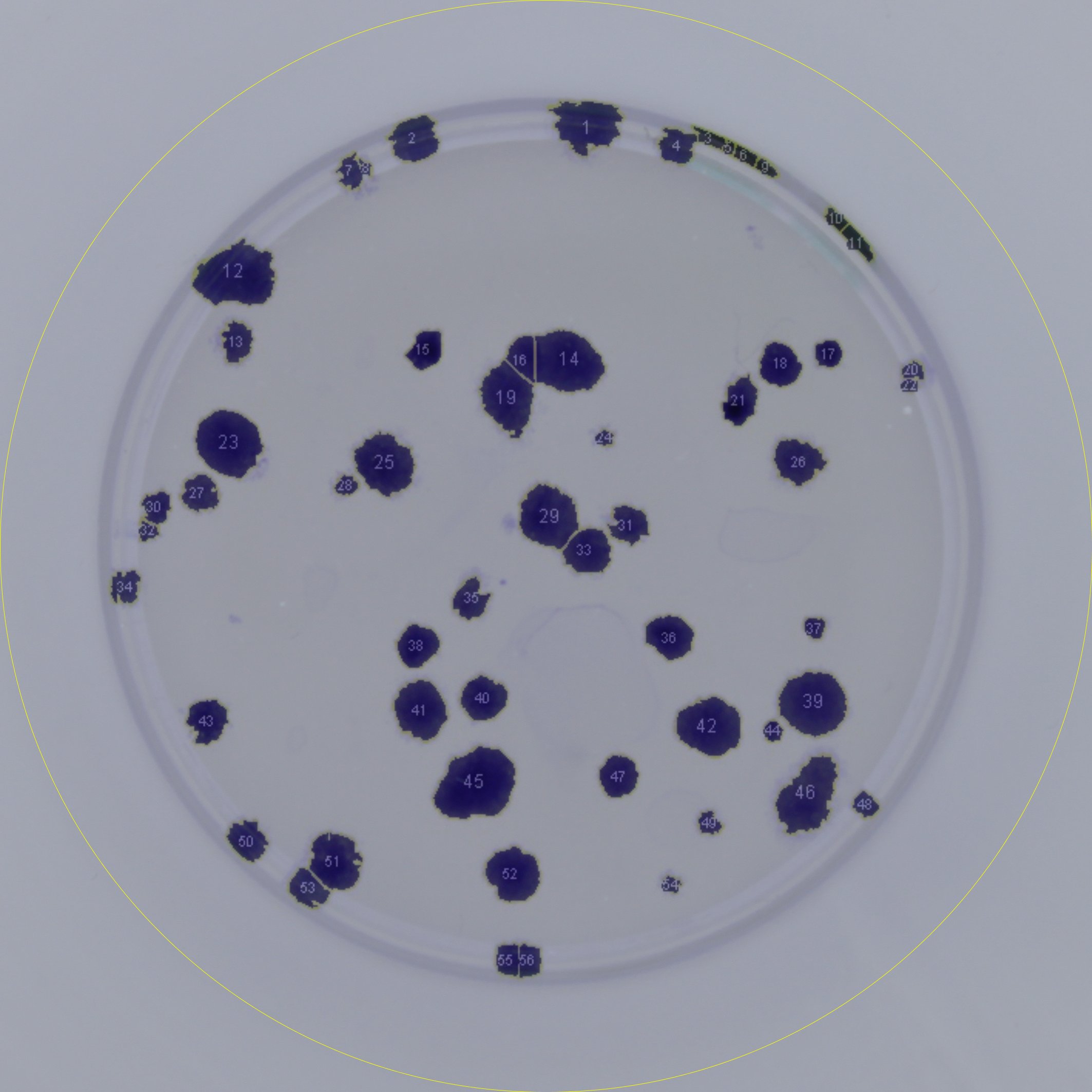

Supplement: S1 Comparison to others — (ZIP) [file pone.0205823.s007.zip › S1 Comparison to others/CAI/180501 HeLa Dish/8 Results.jpg]

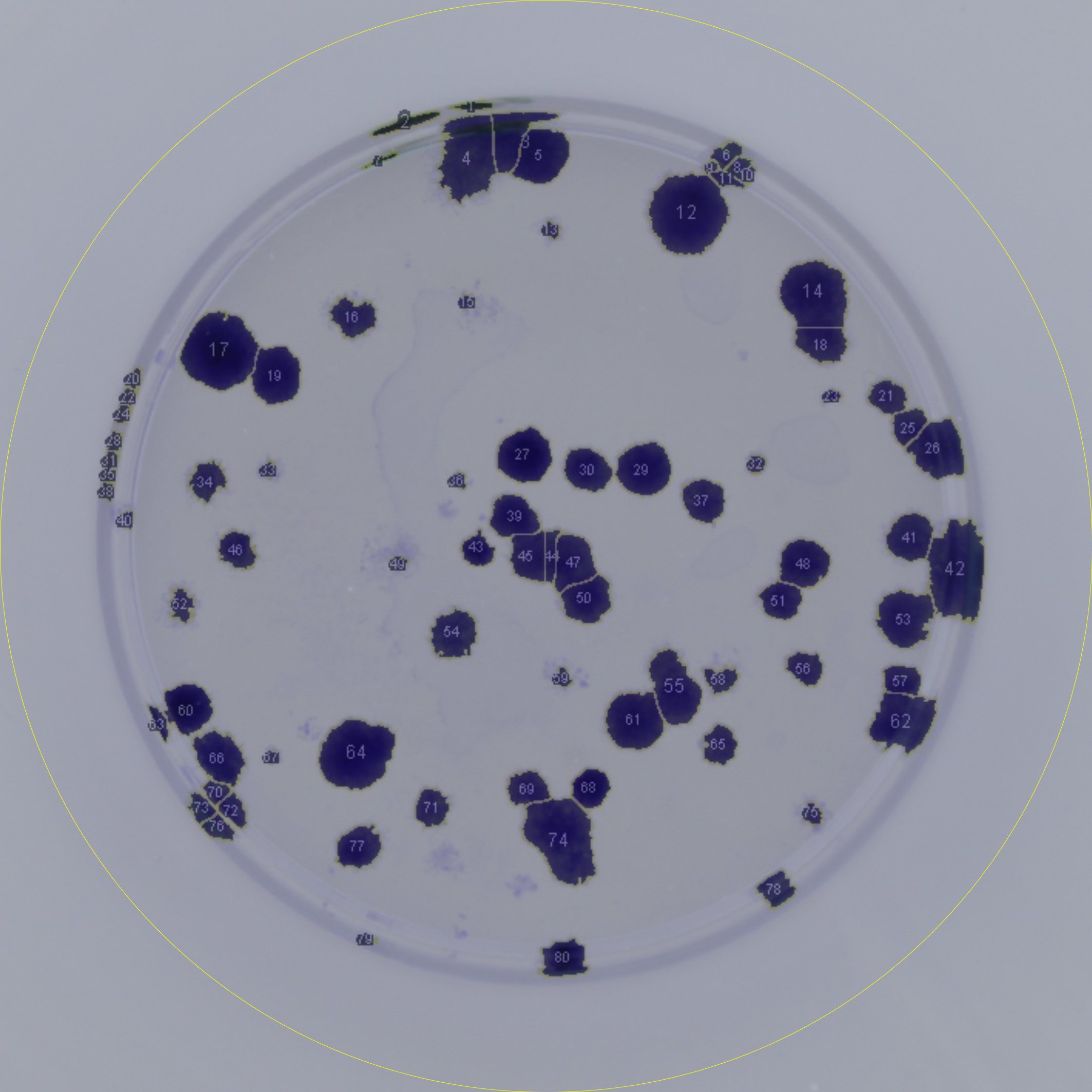

Supplement: S1 Comparison to others — (ZIP) [file pone.0205823.s007.zip › S1 Comparison to others/CAI/180501 HeLa Dish/9 Results.jpg]

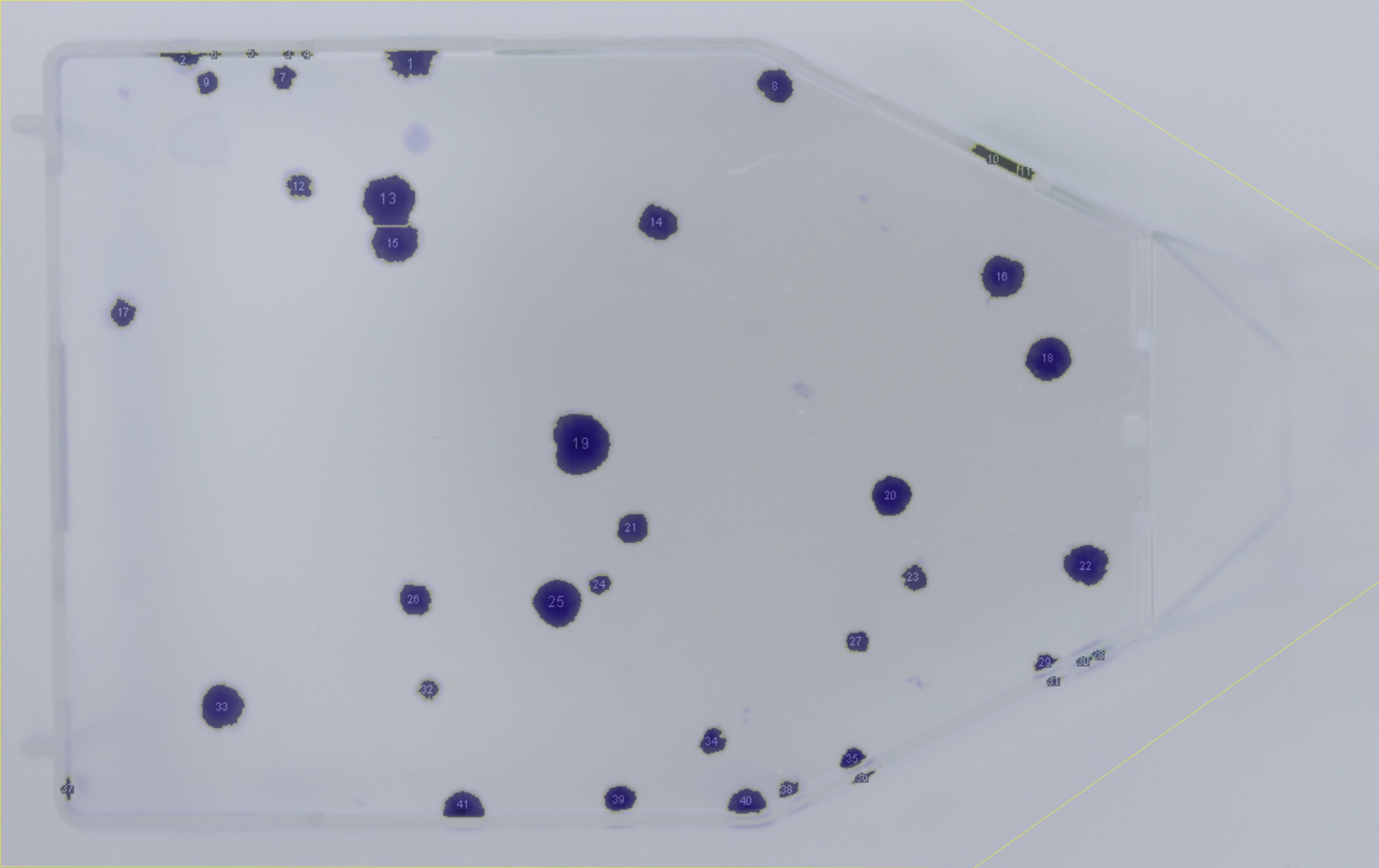

Supplement: S1 Comparison to others — (ZIP) [file pone.0205823.s007.zip › S1 Comparison to others/CAI/180501 HeLa Flask/1 Results.jpg]

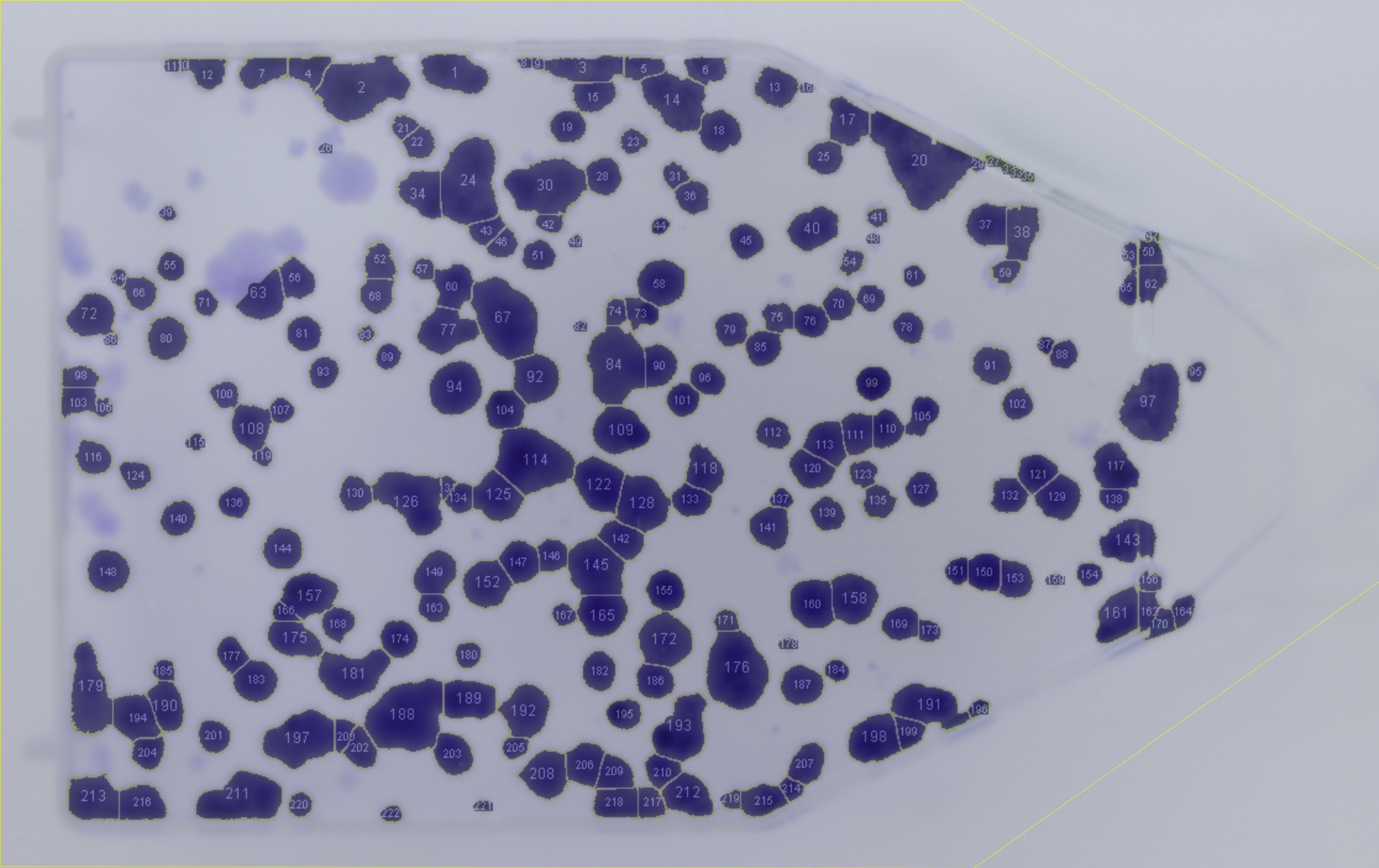

Supplement: S1 Comparison to others — (ZIP) [file pone.0205823.s007.zip › S1 Comparison to others/CAI/180501 HeLa Flask/10 Results.jpg]

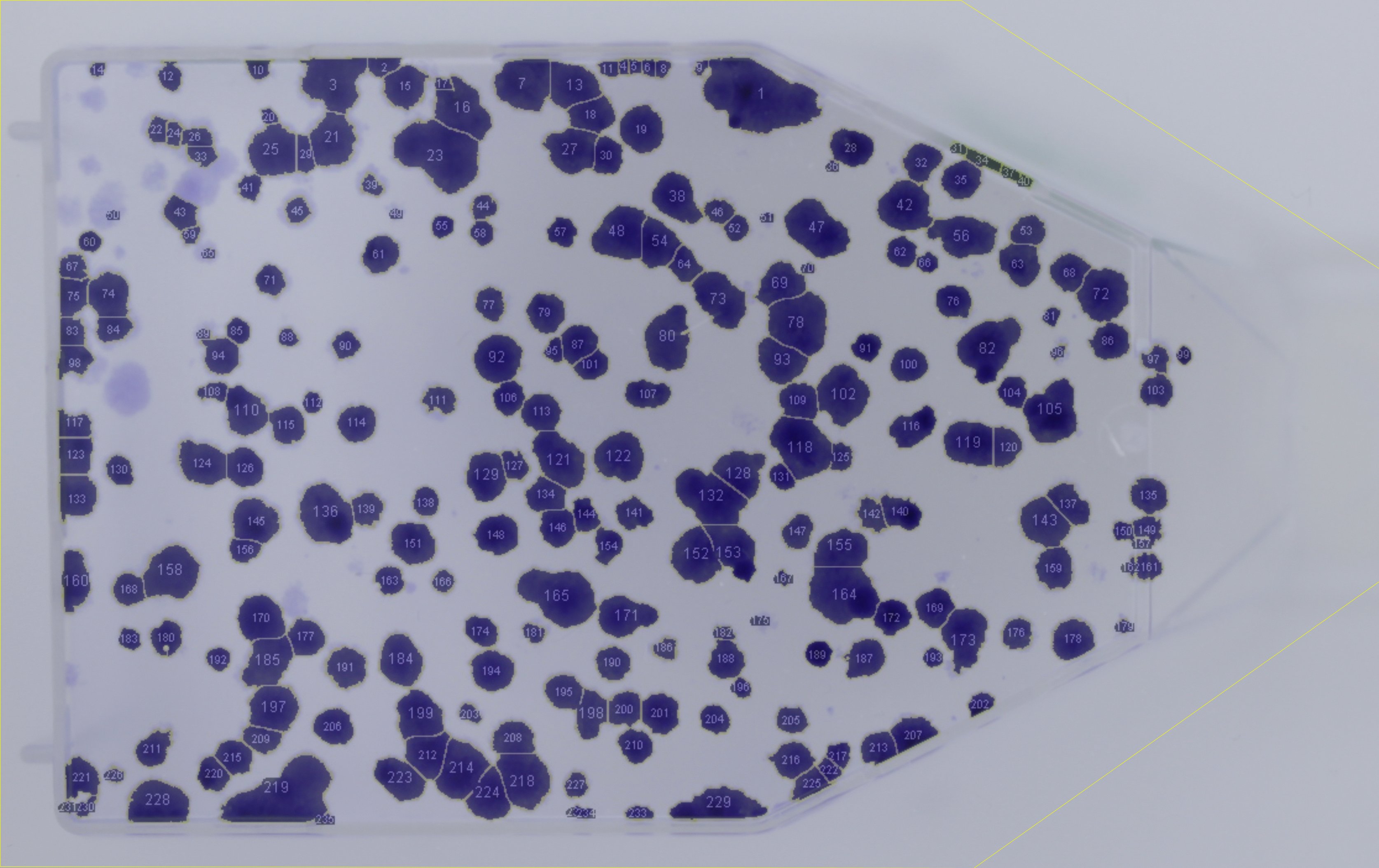

Supplement: S1 Comparison to others — (ZIP) [file pone.0205823.s007.zip › S1 Comparison to others/CAI/180501 HeLa Flask/11 Results.jpg]

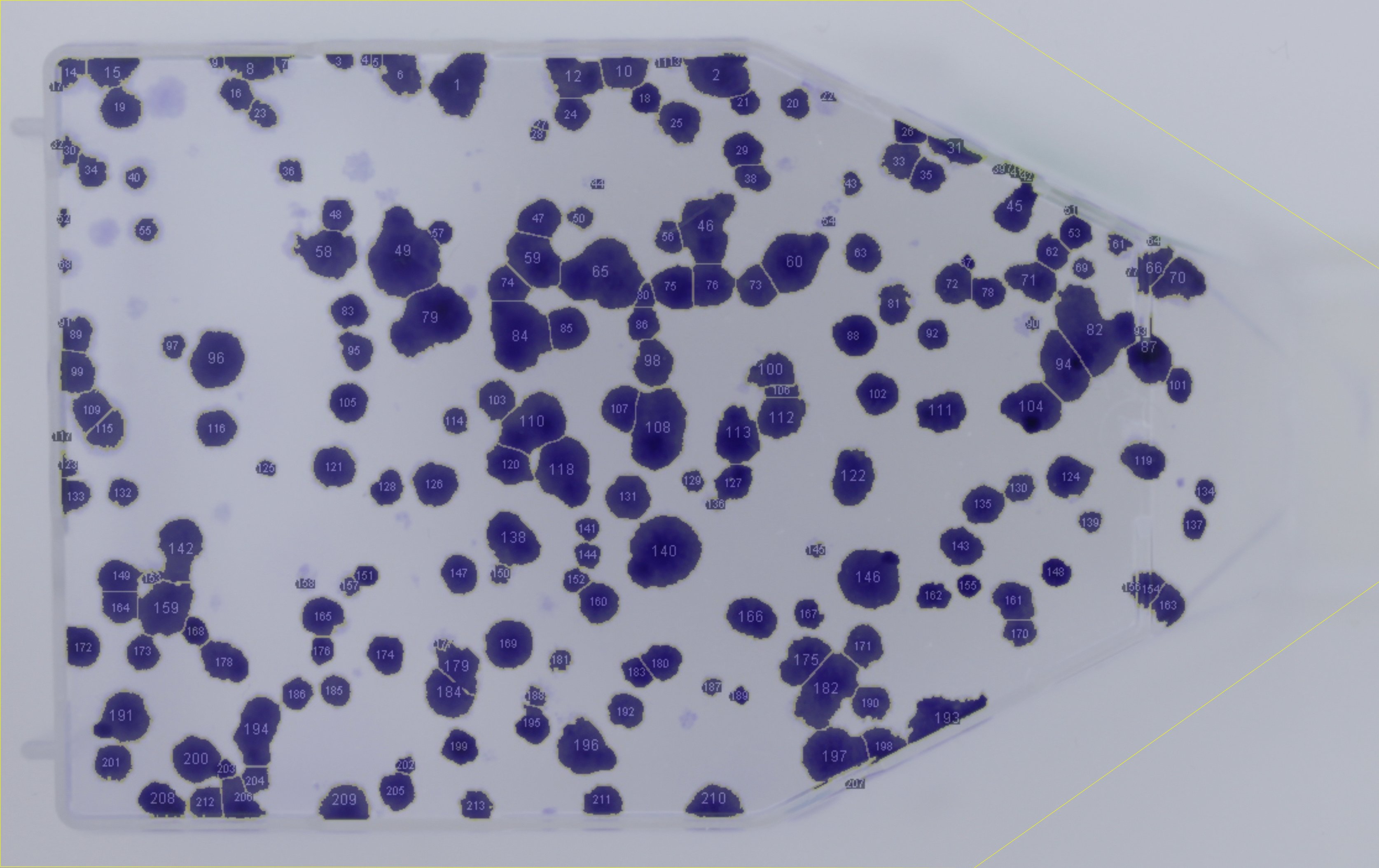

Supplement: S1 Comparison to others — (ZIP) [file pone.0205823.s007.zip › S1 Comparison to others/CAI/180501 HeLa Flask/12 Results.jpg]

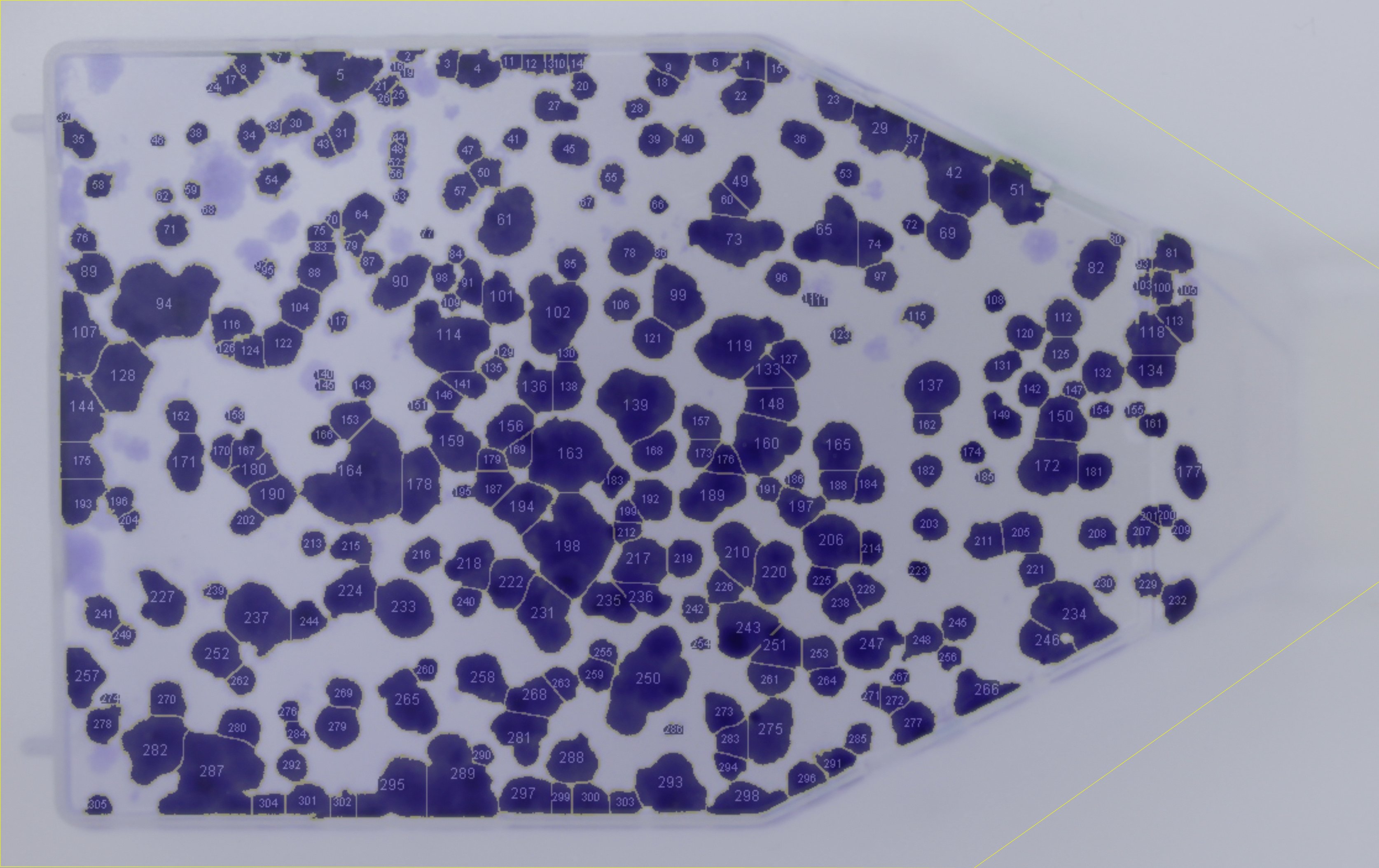

Supplement: S1 Comparison to others — (ZIP) [file pone.0205823.s007.zip › S1 Comparison to others/CAI/180501 HeLa Flask/13 Results.jpg]

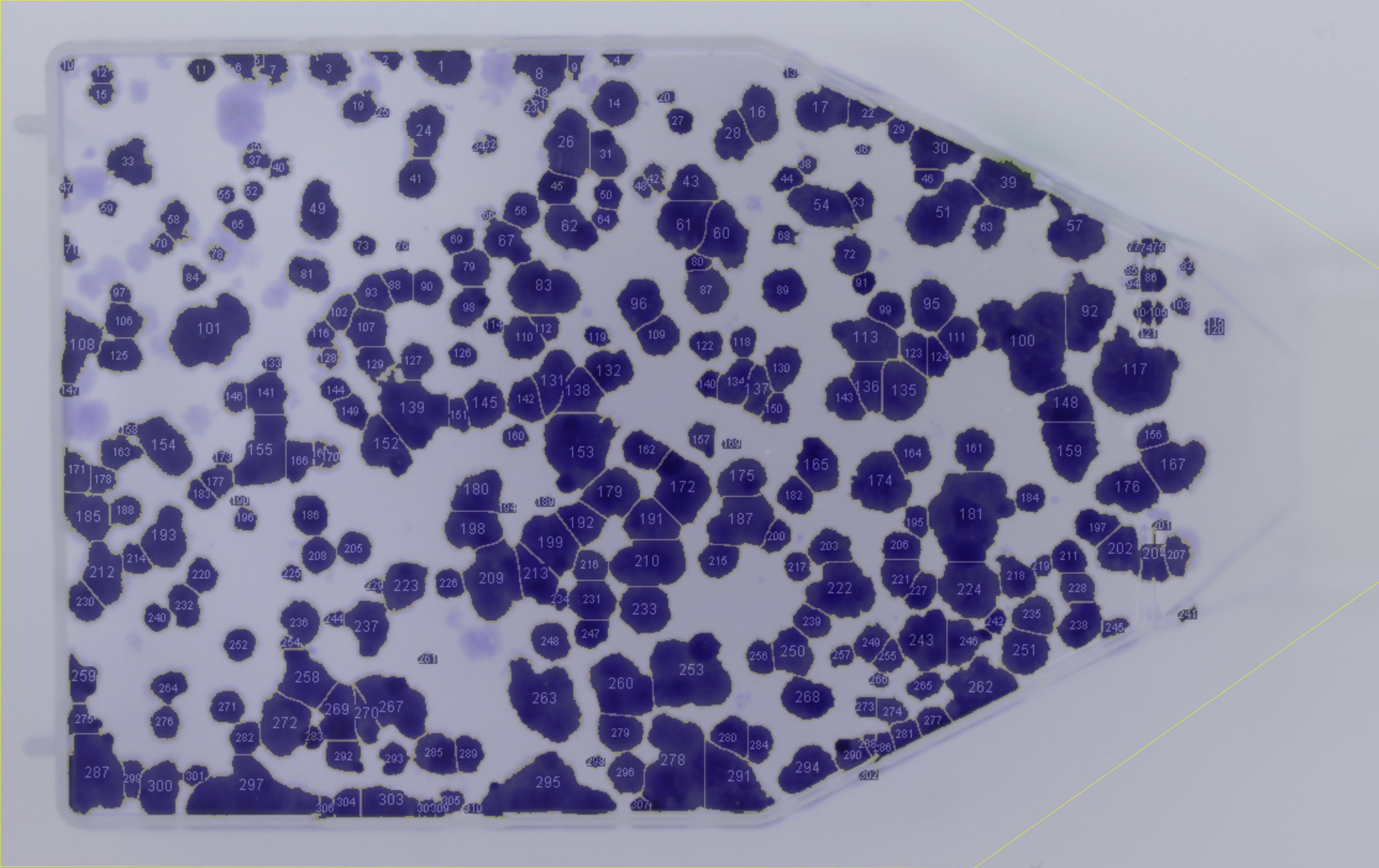

Supplement: S1 Comparison to others — (ZIP) [file pone.0205823.s007.zip › S1 Comparison to others/CAI/180501 HeLa Flask/14 Results.jpg]

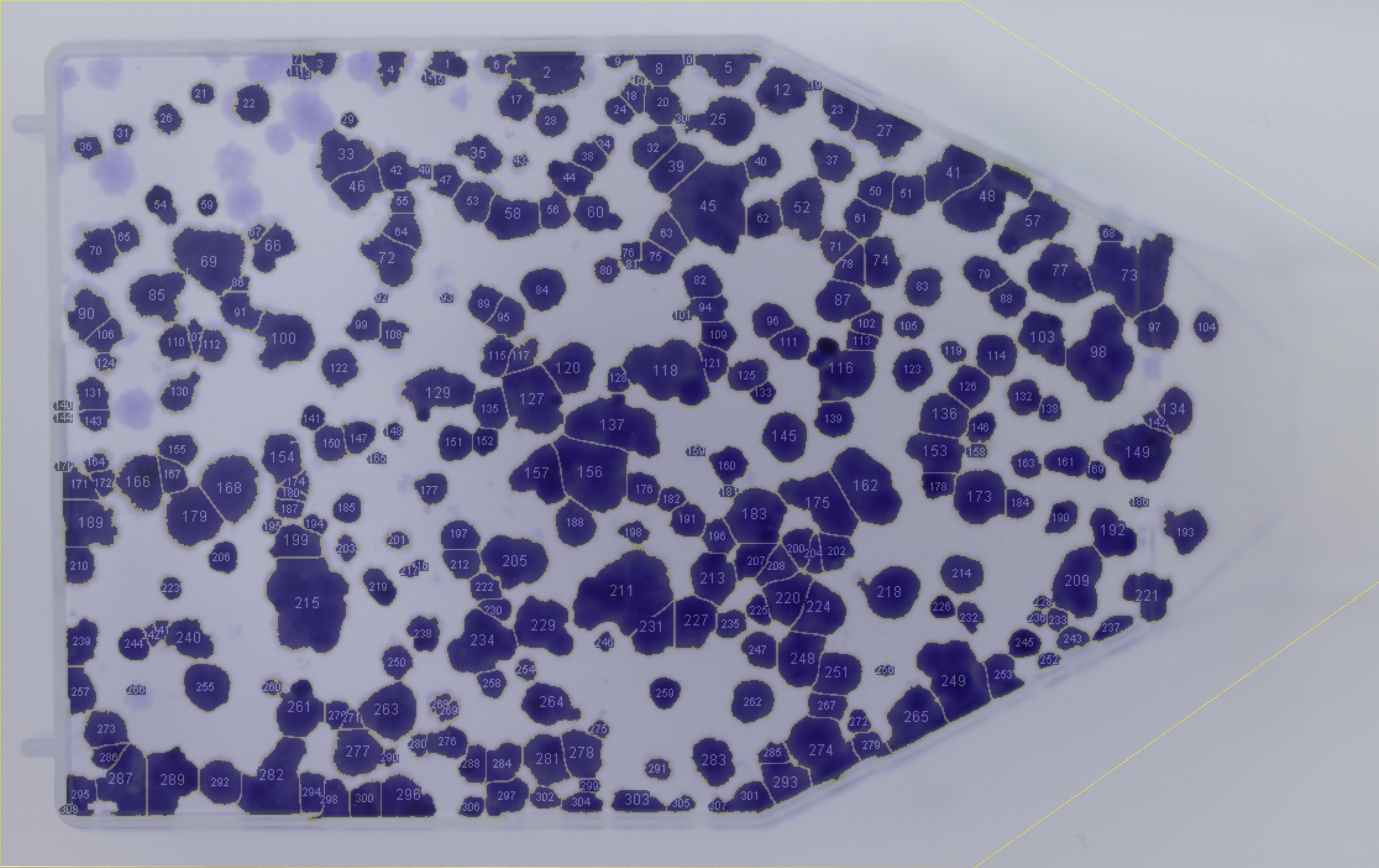

Supplement: S1 Comparison to others — (ZIP) [file pone.0205823.s007.zip › S1 Comparison to others/CAI/180501 HeLa Flask/15 Results.jpg]

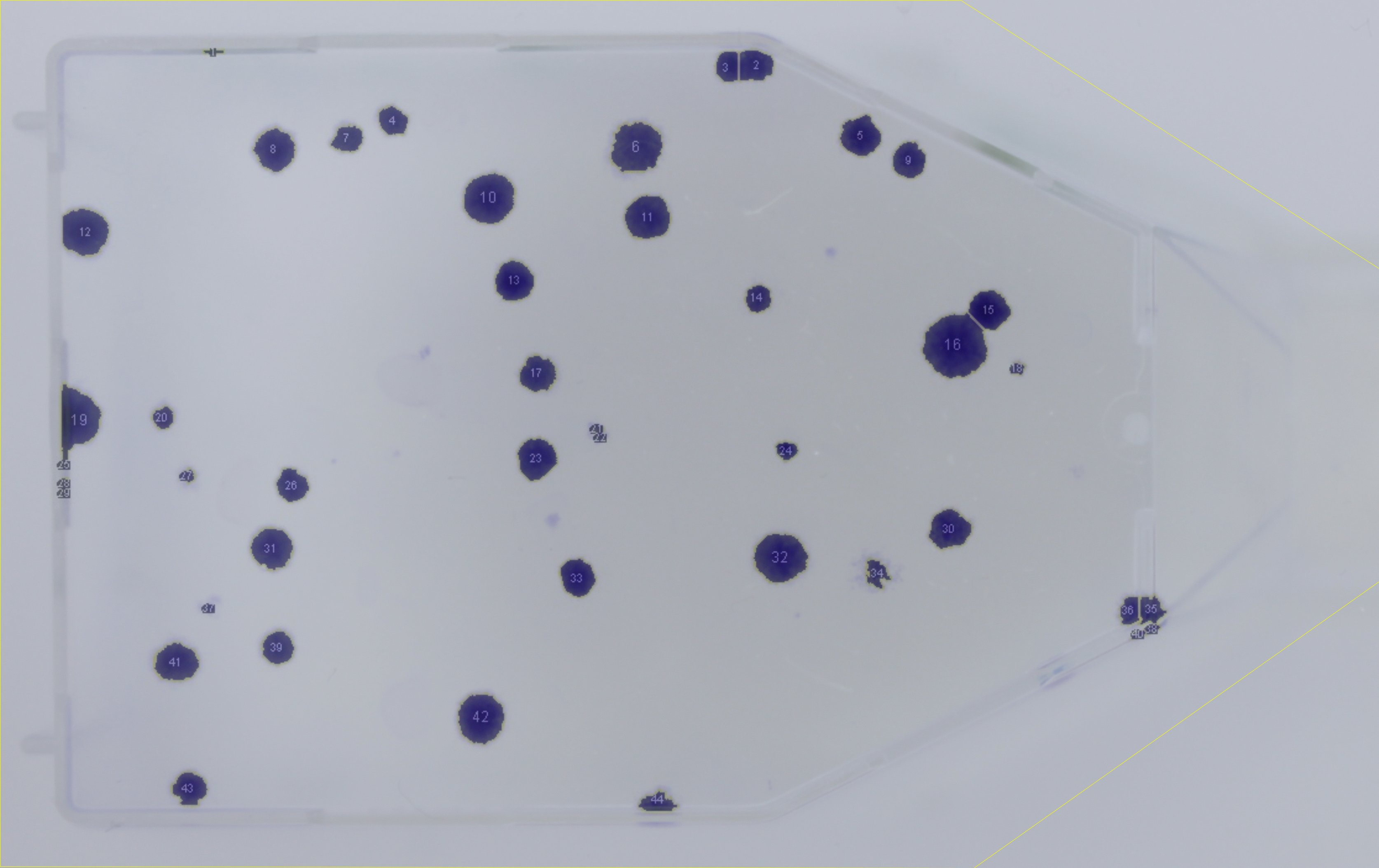

Supplement: S1 Comparison to others — (ZIP) [file pone.0205823.s007.zip › S1 Comparison to others/CAI/180501 HeLa Flask/2 Results.jpg]

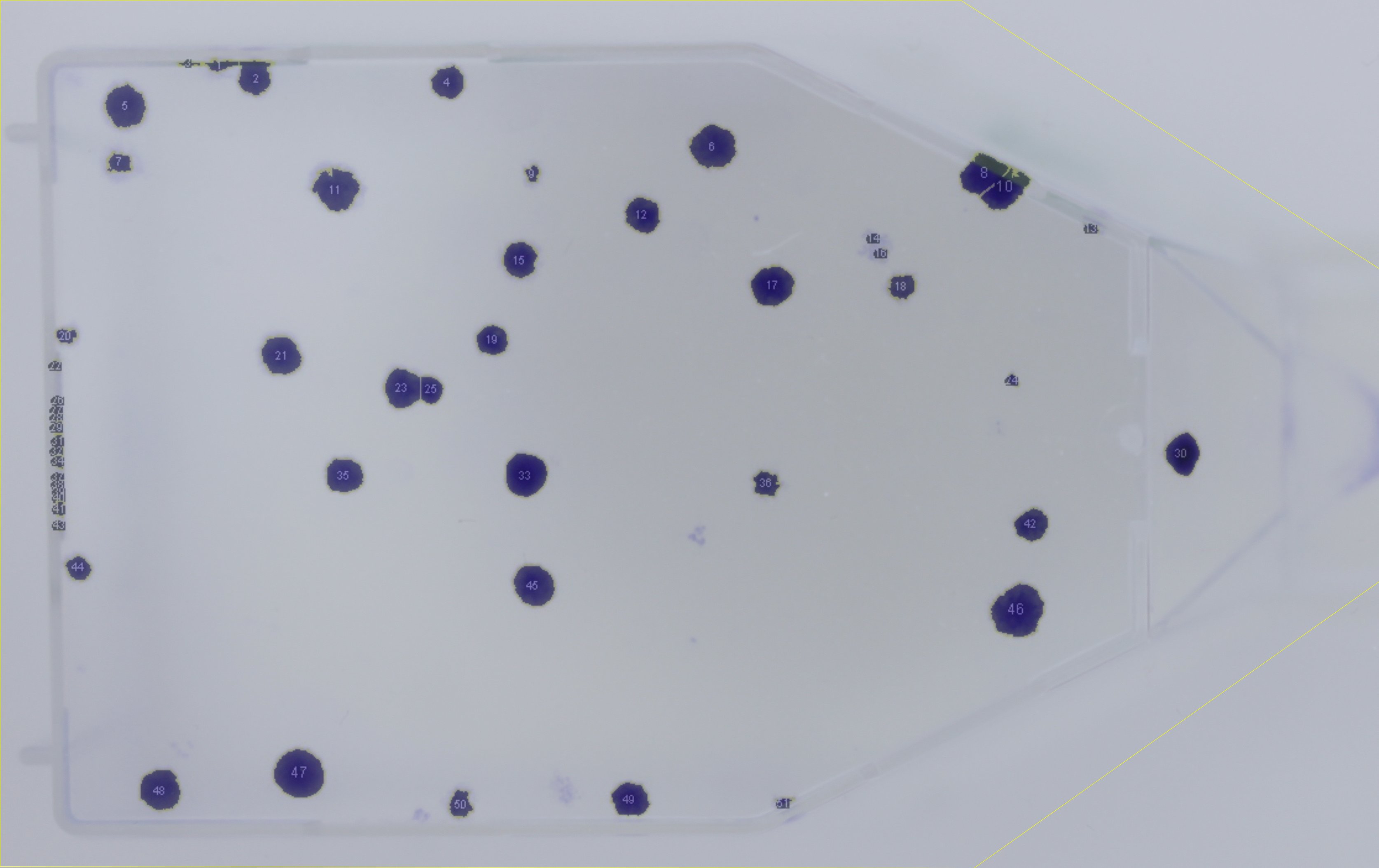

Supplement: S1 Comparison to others — (ZIP) [file pone.0205823.s007.zip › S1 Comparison to others/CAI/180501 HeLa Flask/3 Results.jpg]

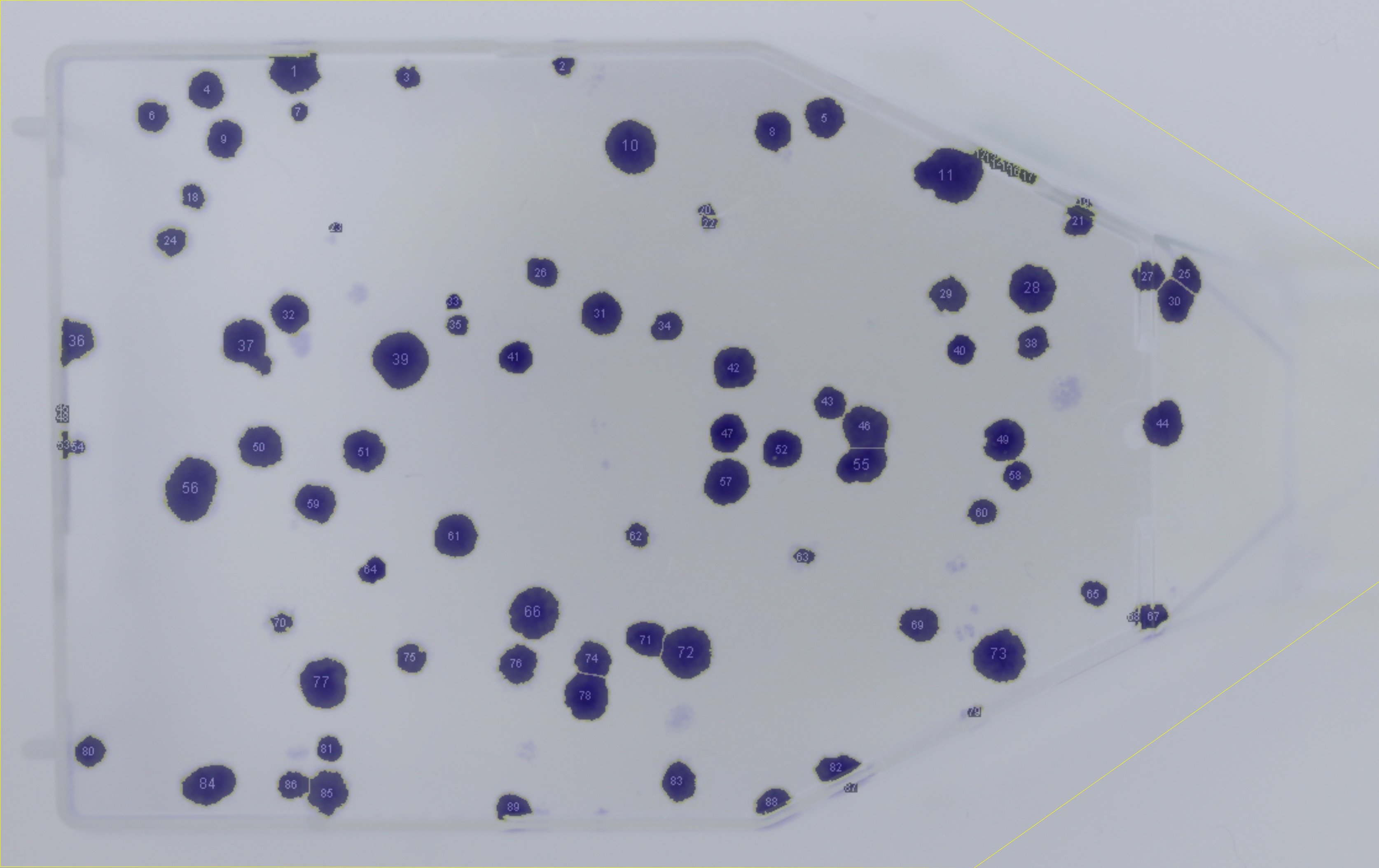

Supplement: S1 Comparison to others — (ZIP) [file pone.0205823.s007.zip › S1 Comparison to others/CAI/180501 HeLa Flask/4 Results.jpg]

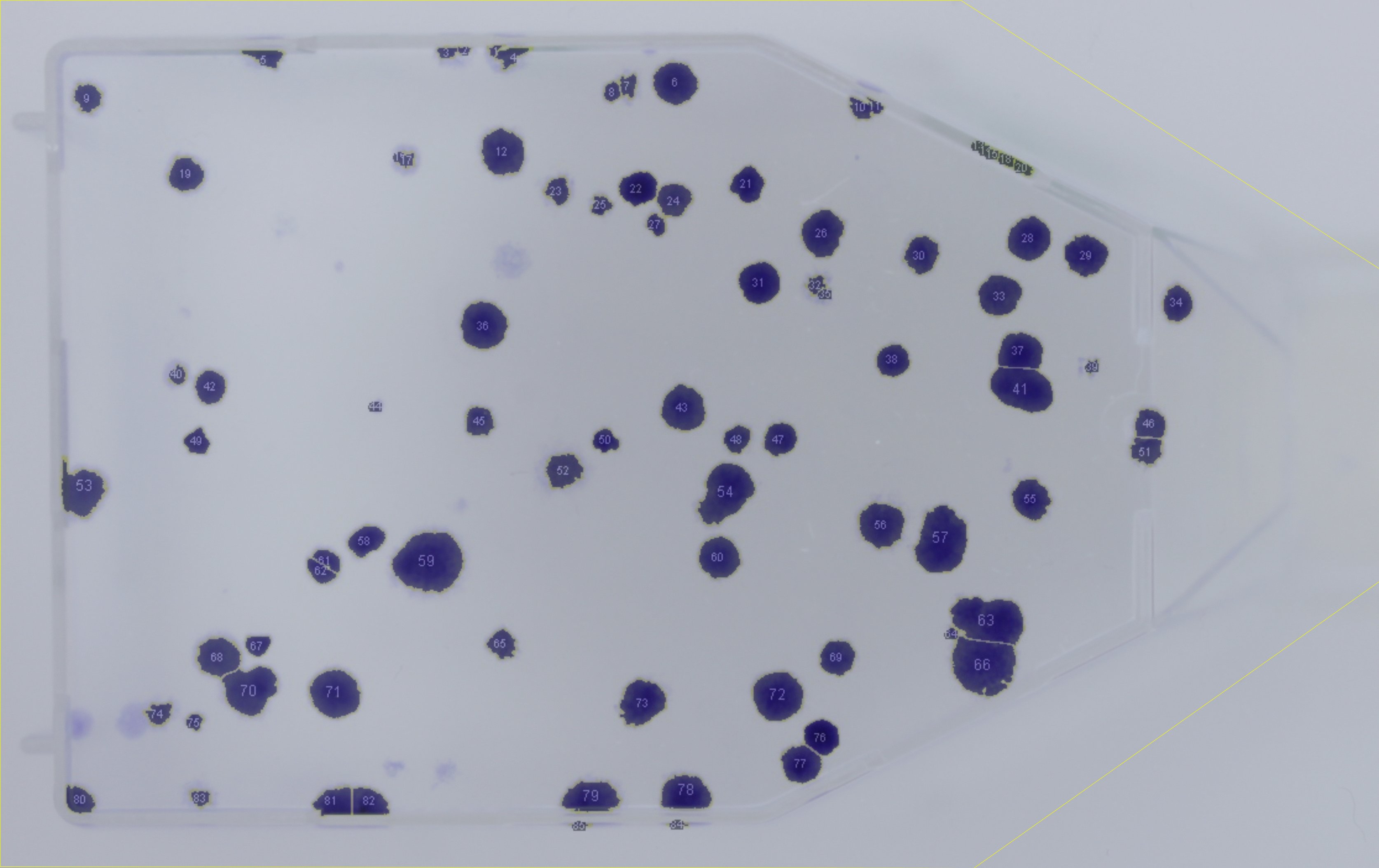

Supplement: S1 Comparison to others — (ZIP) [file pone.0205823.s007.zip › S1 Comparison to others/CAI/180501 HeLa Flask/5 Results.jpg]

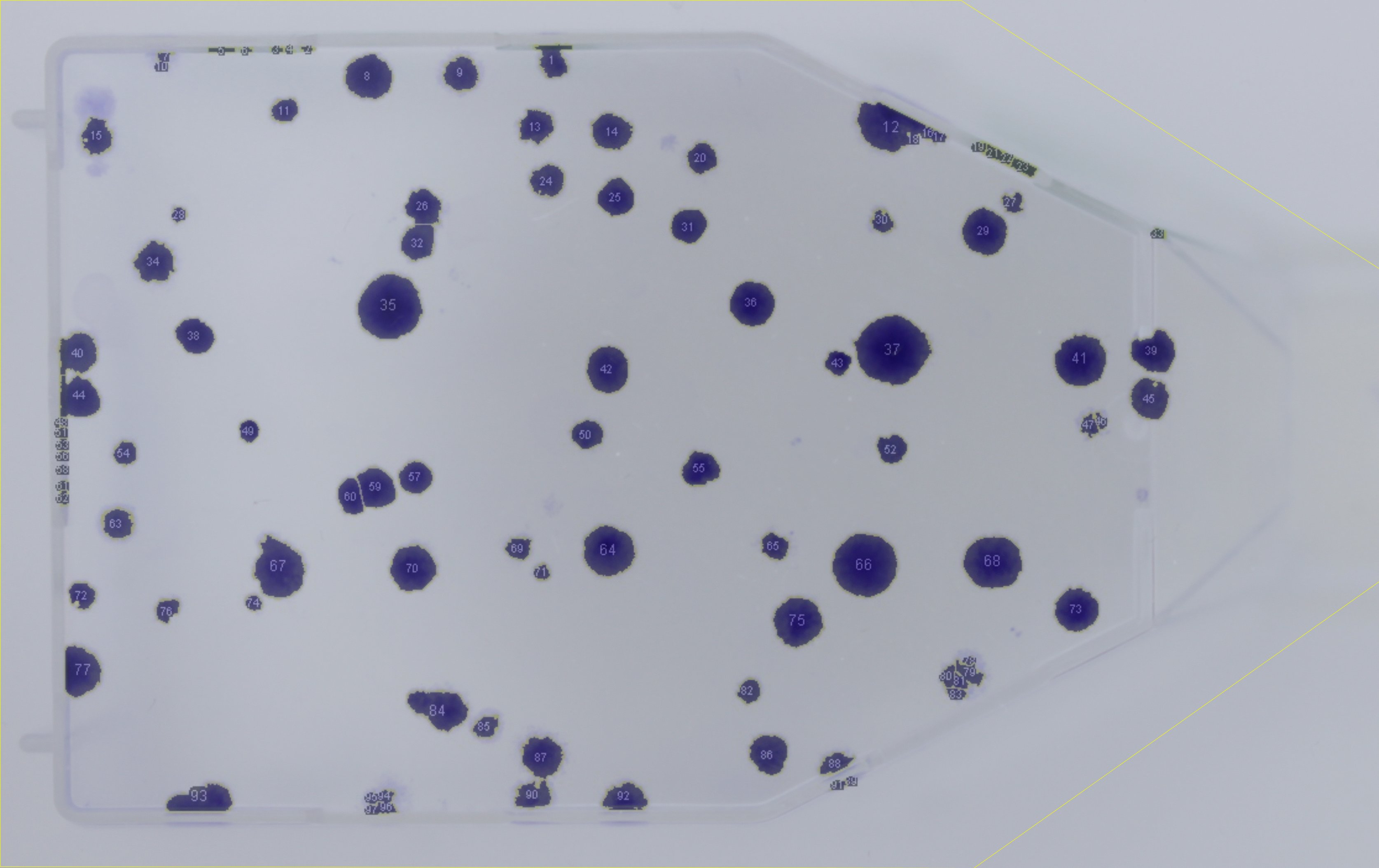

Supplement: S1 Comparison to others — (ZIP) [file pone.0205823.s007.zip › S1 Comparison to others/CAI/180501 HeLa Flask/6 Results.jpg]
