# Supplementary figures and images for: Cell colony counter called CoCoNut (part 4 of 5)
Source: PLoS One. 2018 Nov 7;13(11):e0205823. doi: 10.1371/journal.pone.0205823 (PMC6221277; doi:10.1371/journal.pone.0205823)

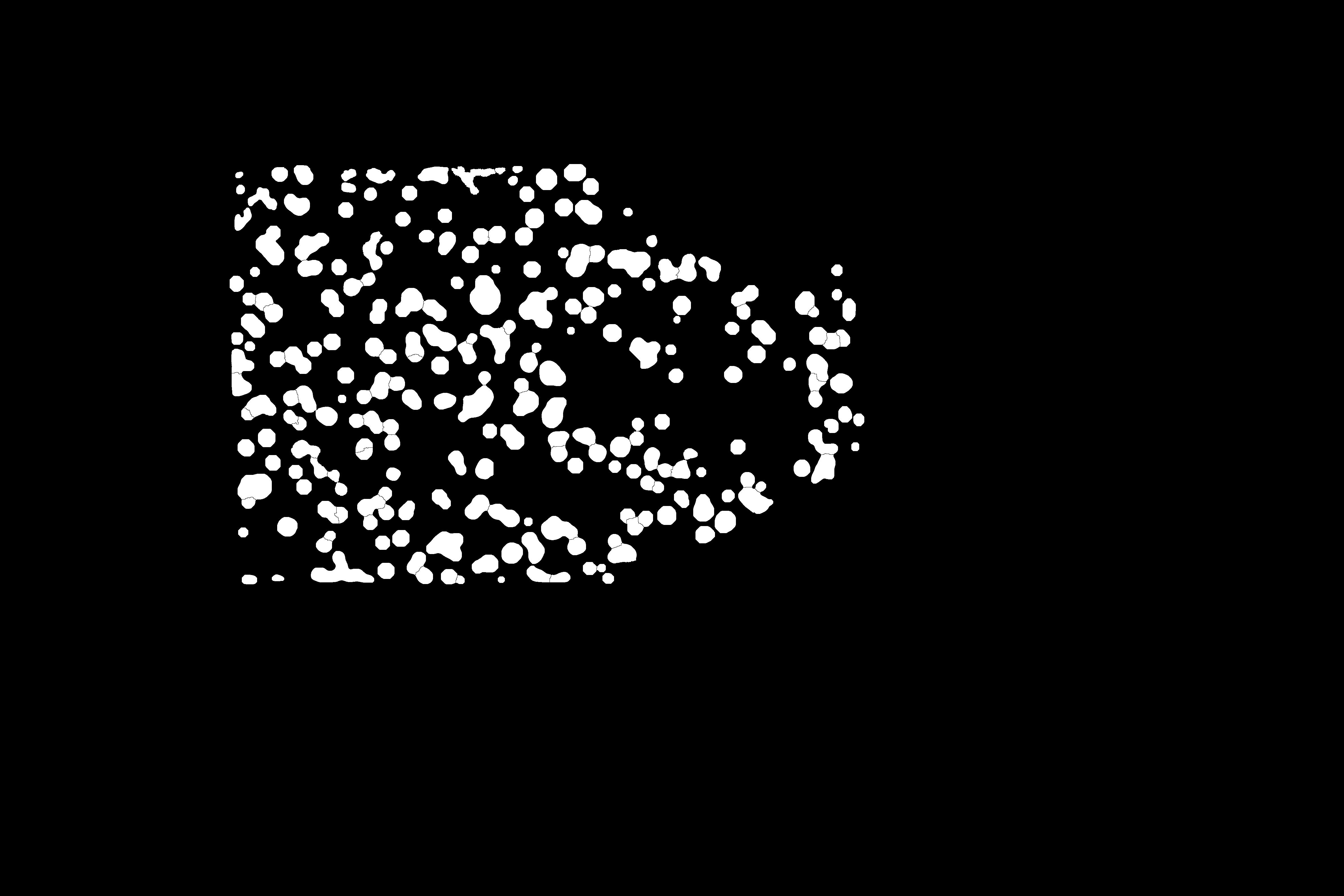

Supplement: S1 Comparison to others — (ZIP) [file pone.0205823.s007.zip › S1 Comparison to others/AutoCellSeg/171214 V79 Flask/14_mask.jpg]

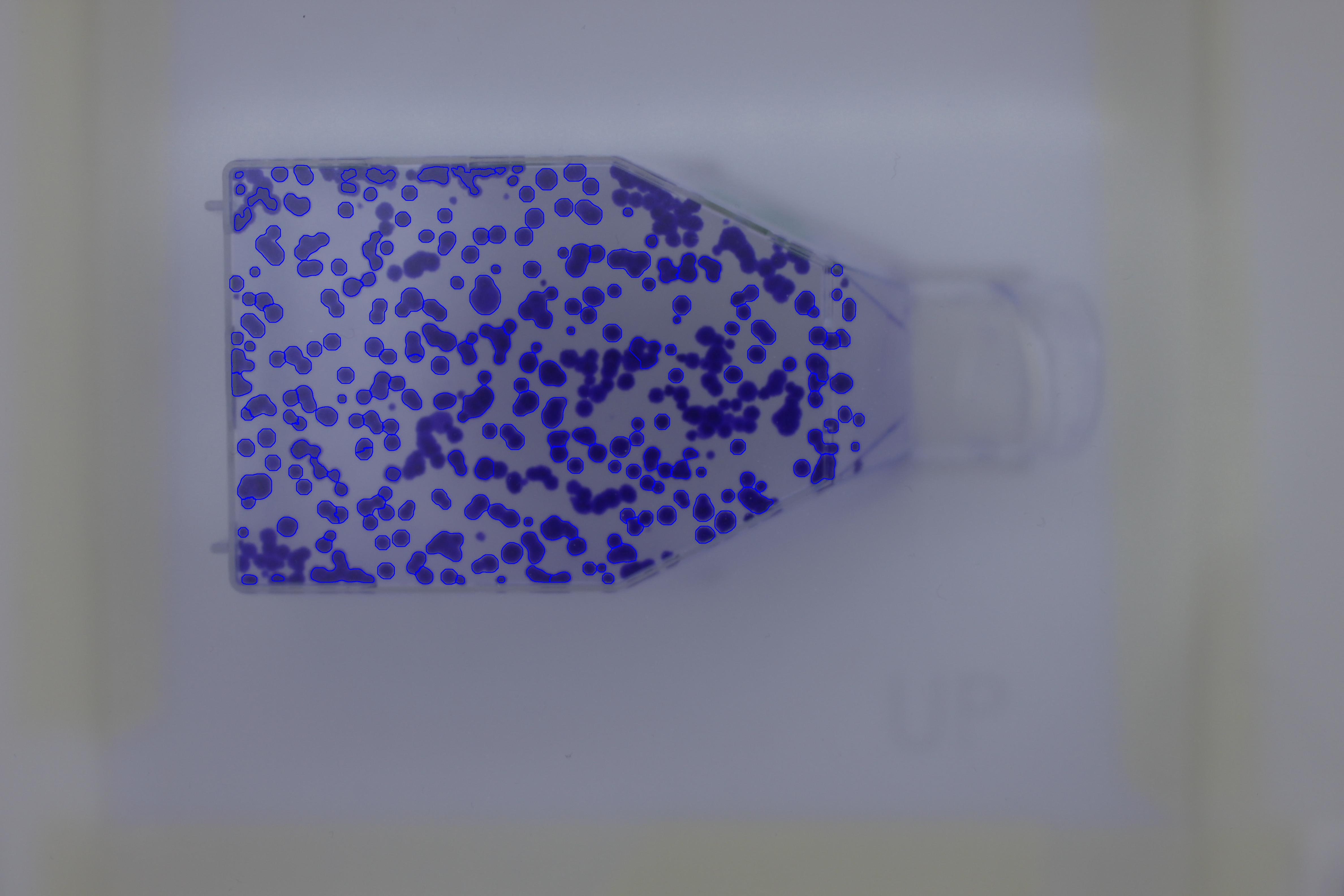

Supplement: S1 Comparison to others — (ZIP) [file pone.0205823.s007.zip › S1 Comparison to others/AutoCellSeg/171214 V79 Flask/14_seg.jpg]

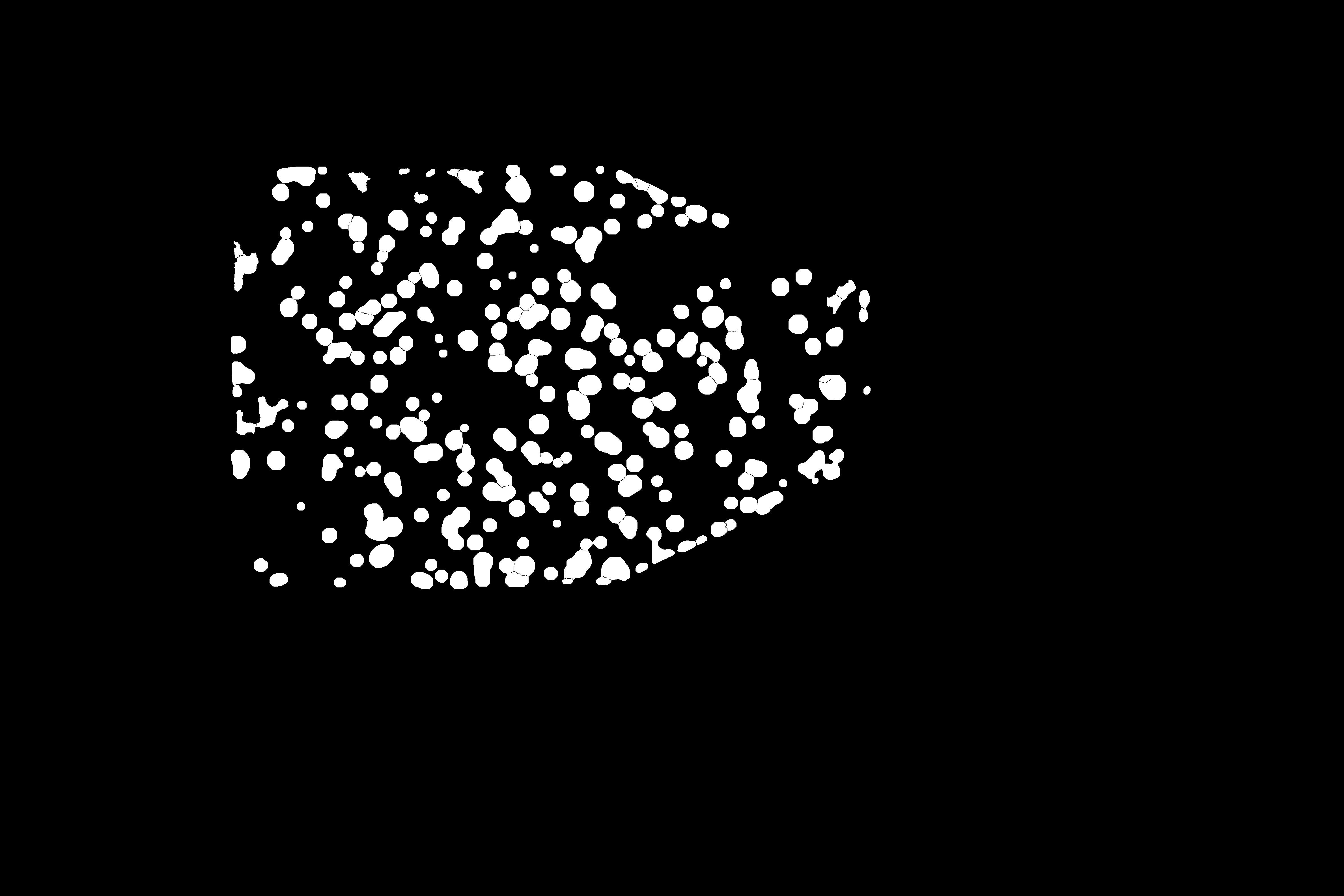

Supplement: S1 Comparison to others — (ZIP) [file pone.0205823.s007.zip › S1 Comparison to others/AutoCellSeg/171214 V79 Flask/15_mask.jpg]

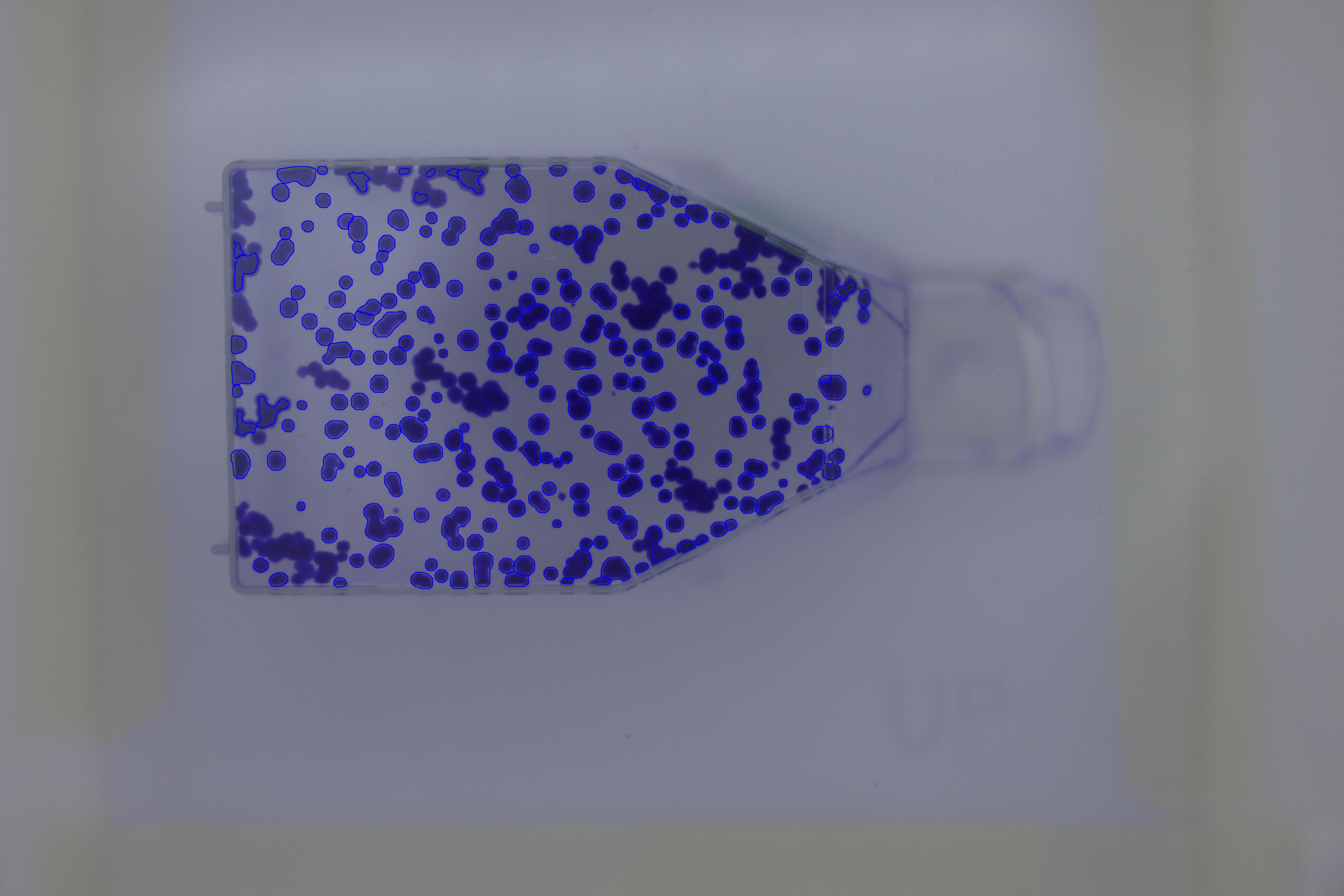

Supplement: S1 Comparison to others — (ZIP) [file pone.0205823.s007.zip › S1 Comparison to others/AutoCellSeg/171214 V79 Flask/15_seg.jpg]

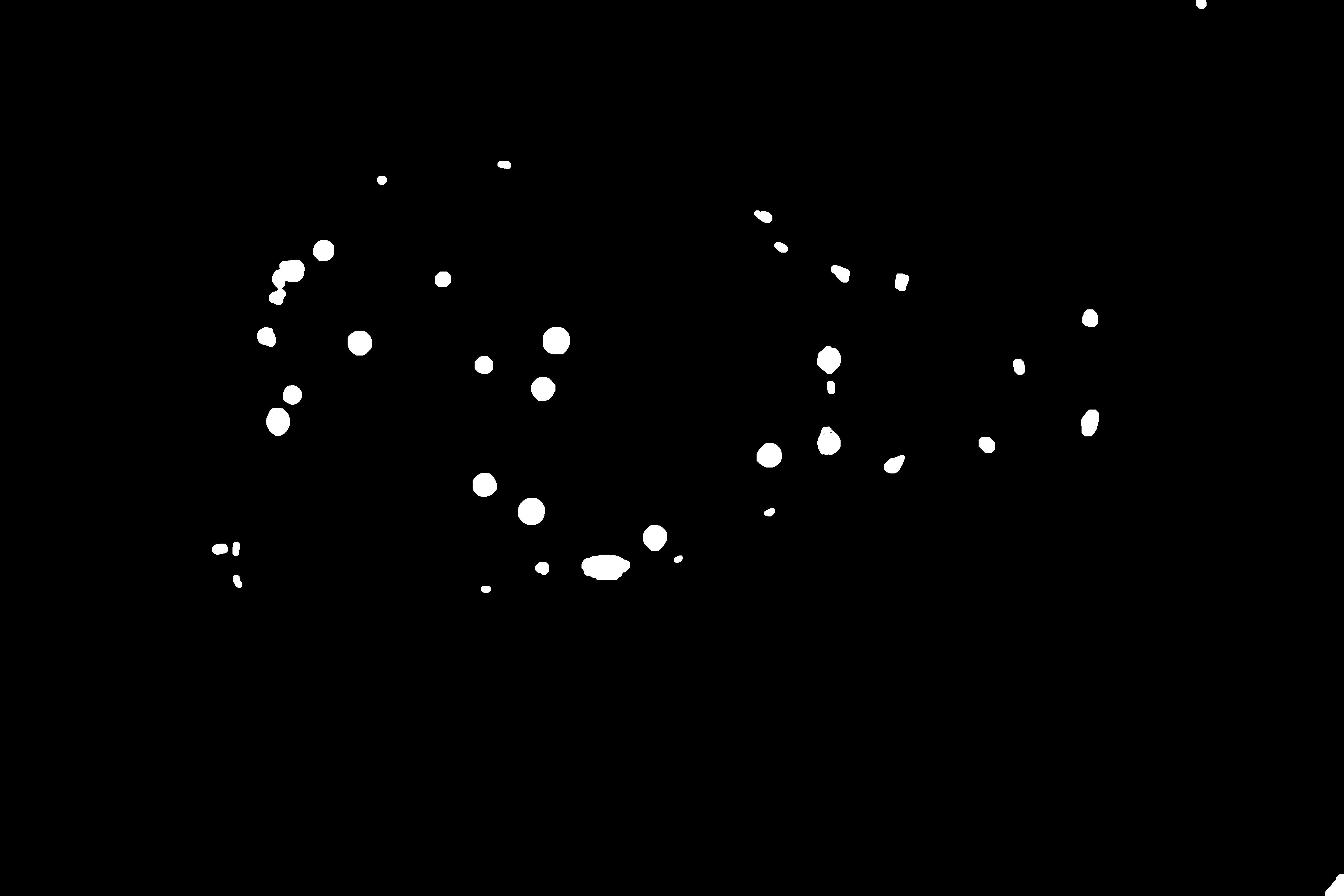

Supplement: S1 Comparison to others — (ZIP) [file pone.0205823.s007.zip › S1 Comparison to others/AutoCellSeg/171214 V79 Flask/1_mask.jpg]

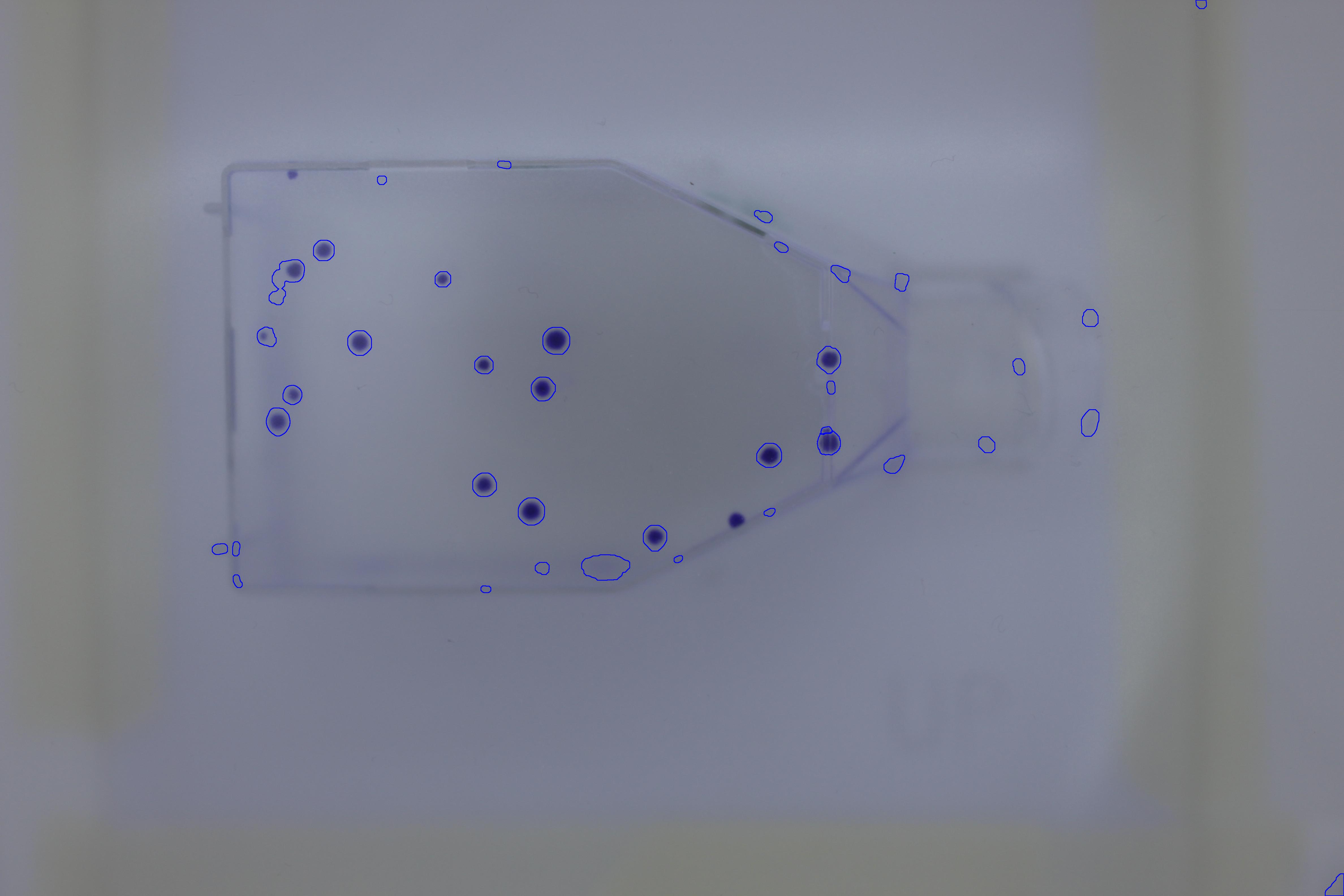

Supplement: S1 Comparison to others — (ZIP) [file pone.0205823.s007.zip › S1 Comparison to others/AutoCellSeg/171214 V79 Flask/1_seg.jpg]

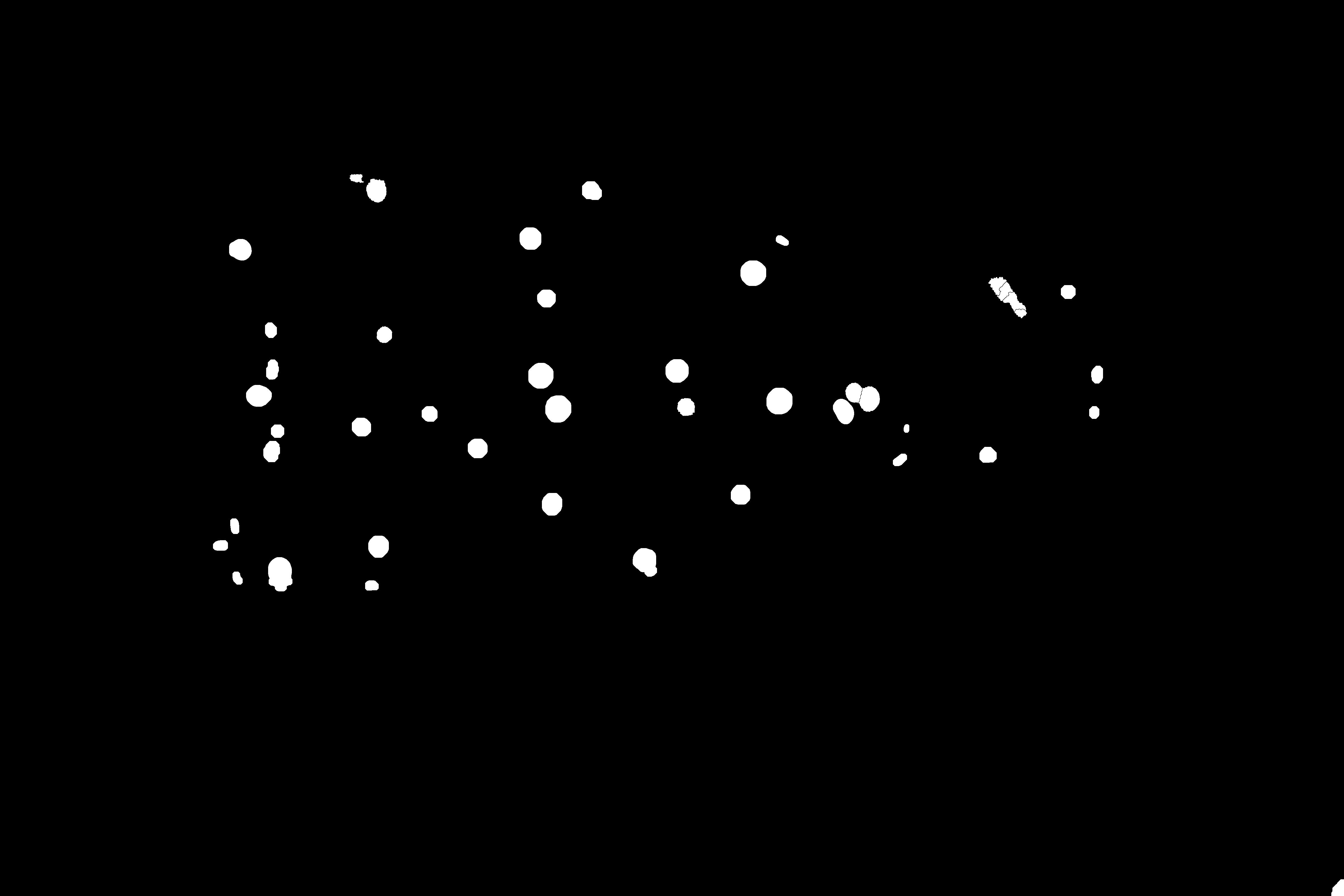

Supplement: S1 Comparison to others — (ZIP) [file pone.0205823.s007.zip › S1 Comparison to others/AutoCellSeg/171214 V79 Flask/2_mask.jpg]

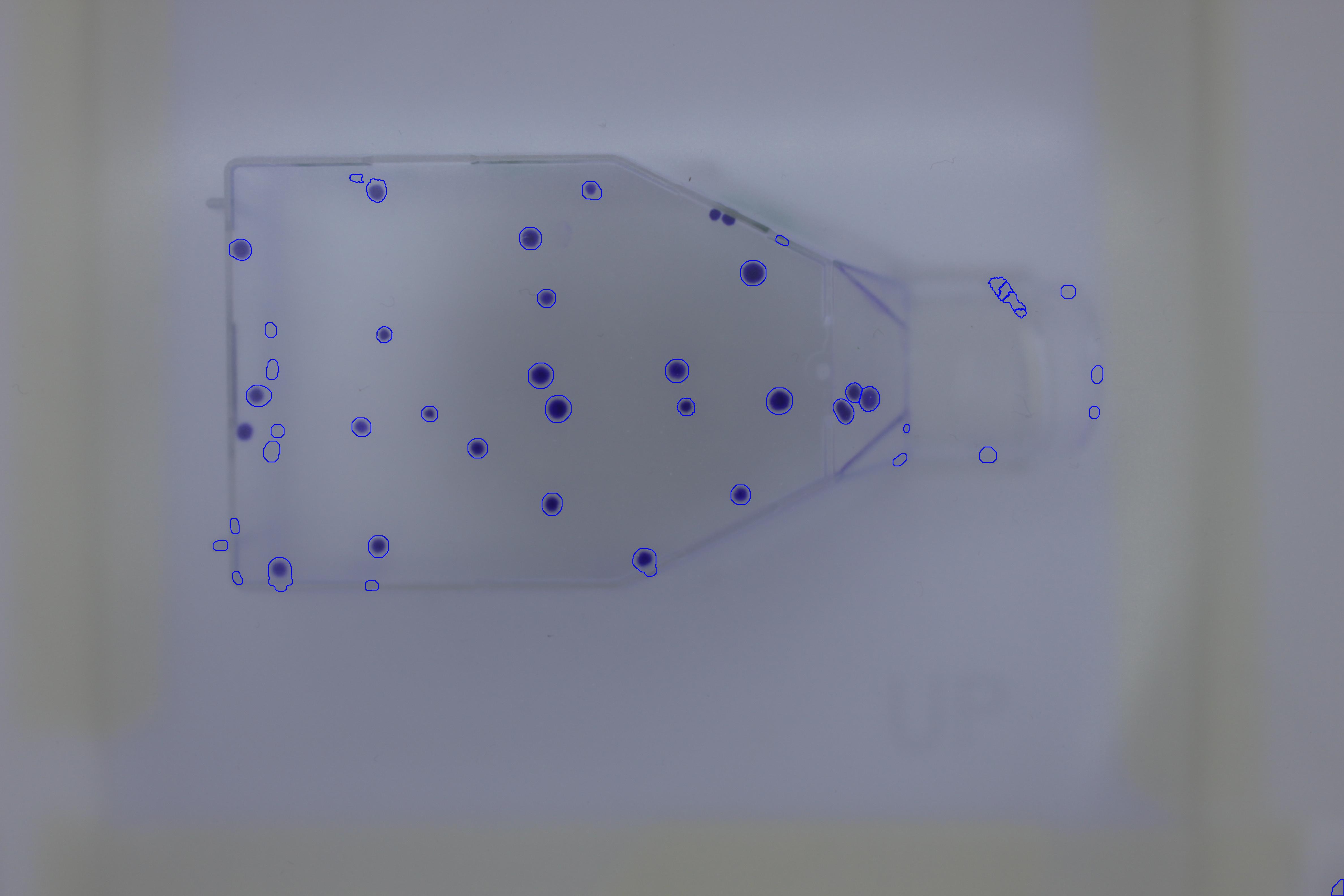

Supplement: S1 Comparison to others — (ZIP) [file pone.0205823.s007.zip › S1 Comparison to others/AutoCellSeg/171214 V79 Flask/2_seg.jpg]

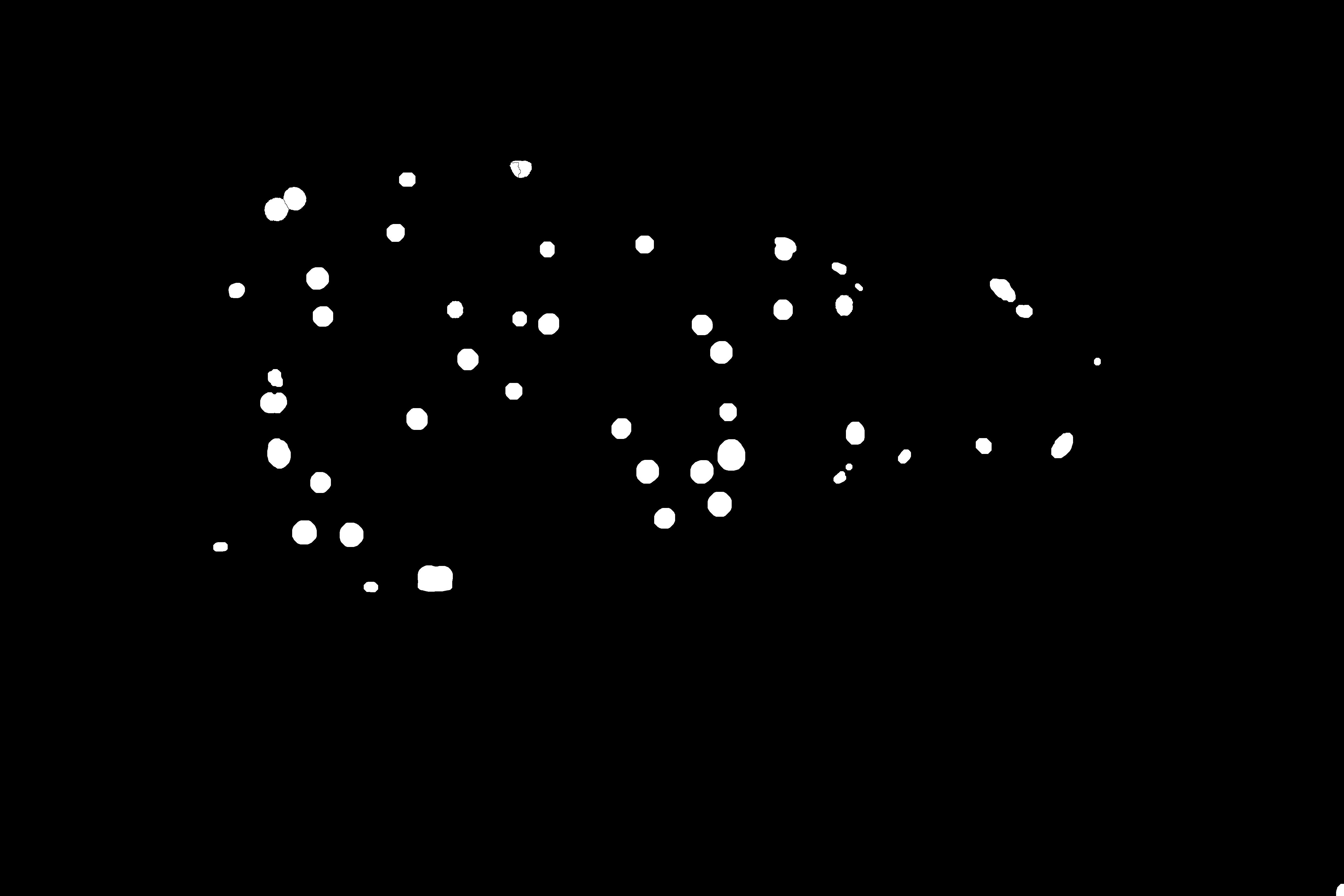

Supplement: S1 Comparison to others — (ZIP) [file pone.0205823.s007.zip › S1 Comparison to others/AutoCellSeg/171214 V79 Flask/3_mask.jpg]

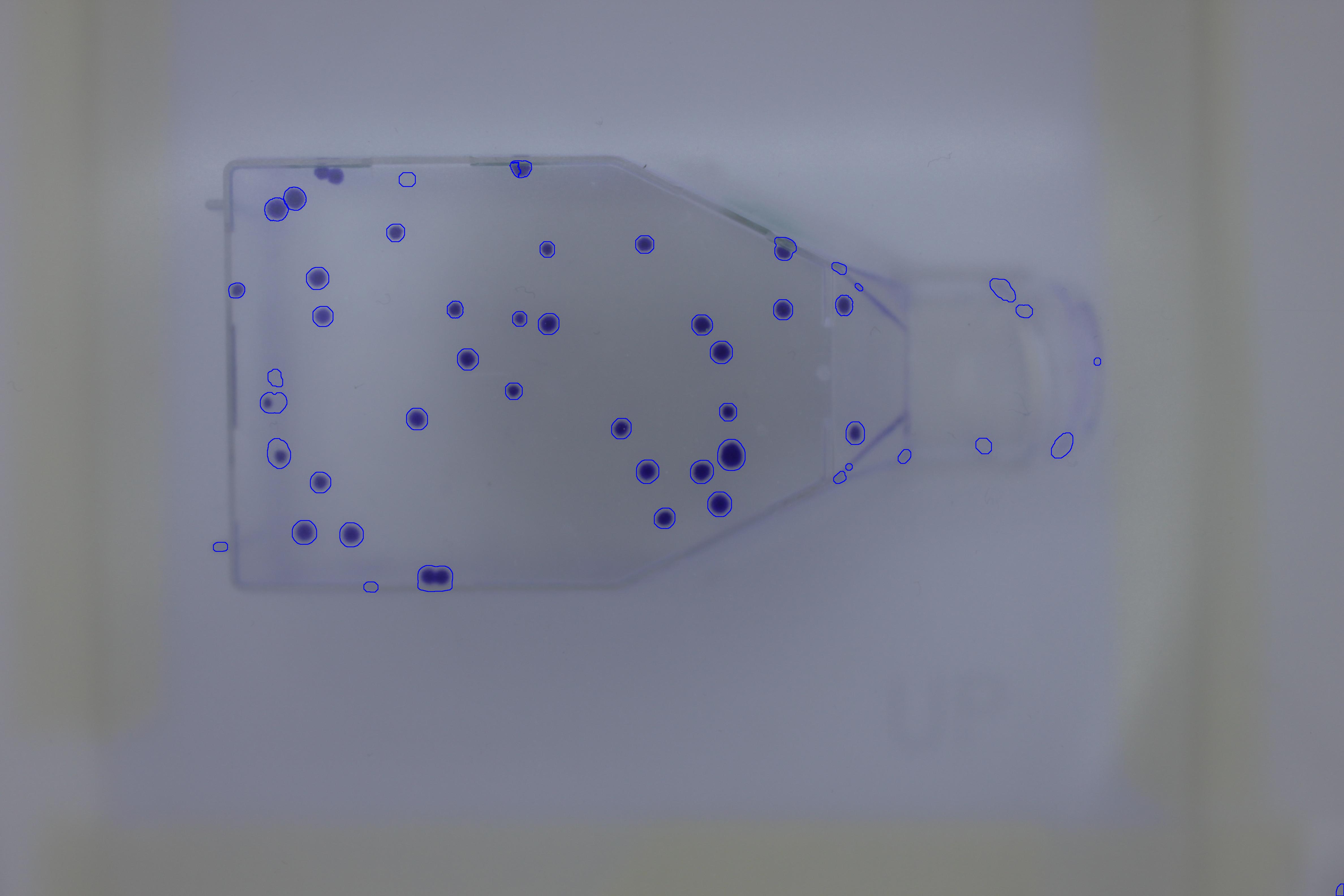

Supplement: S1 Comparison to others — (ZIP) [file pone.0205823.s007.zip › S1 Comparison to others/AutoCellSeg/171214 V79 Flask/3_seg.jpg]

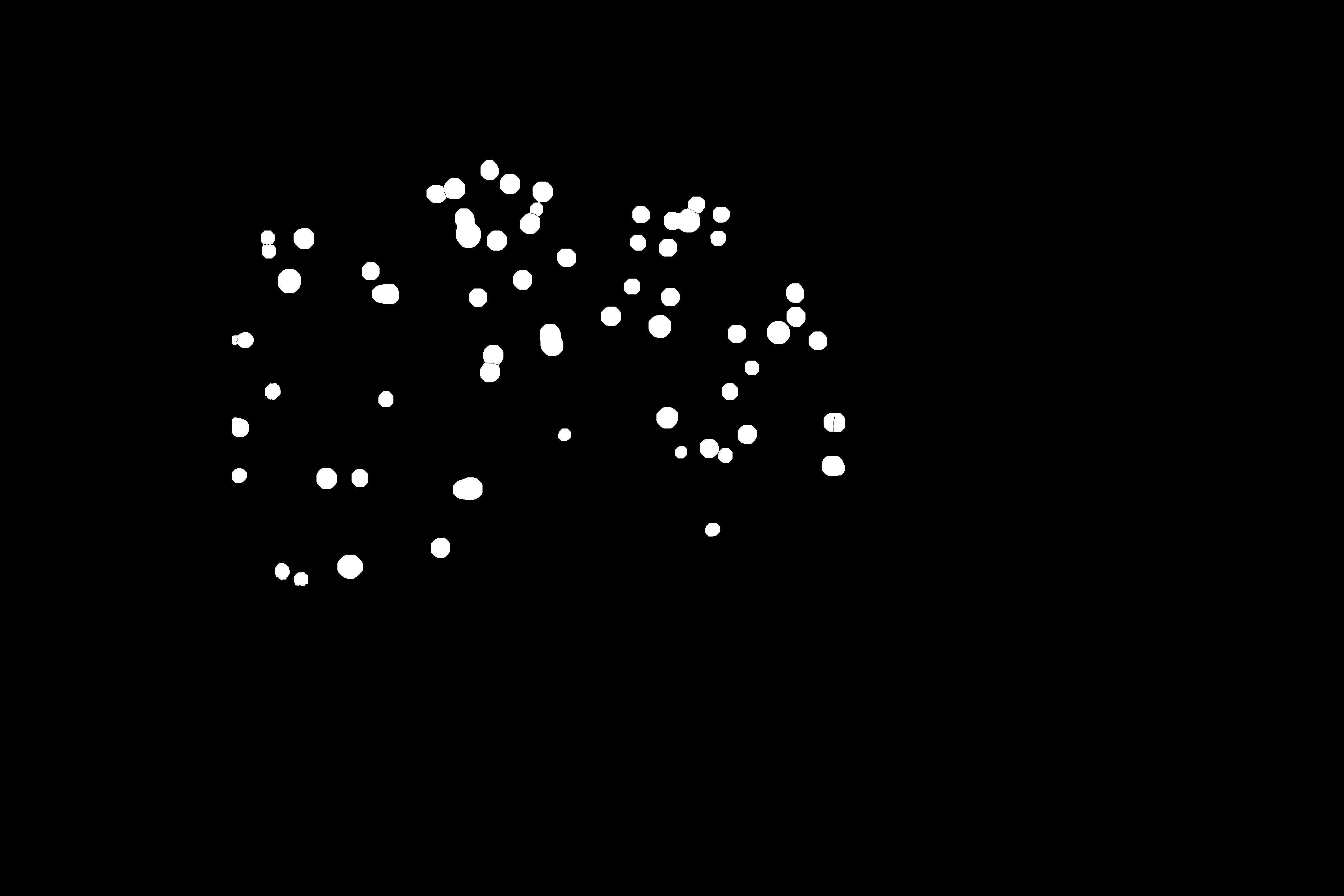

Supplement: S1 Comparison to others — (ZIP) [file pone.0205823.s007.zip › S1 Comparison to others/AutoCellSeg/171214 V79 Flask/4_mask.jpg]

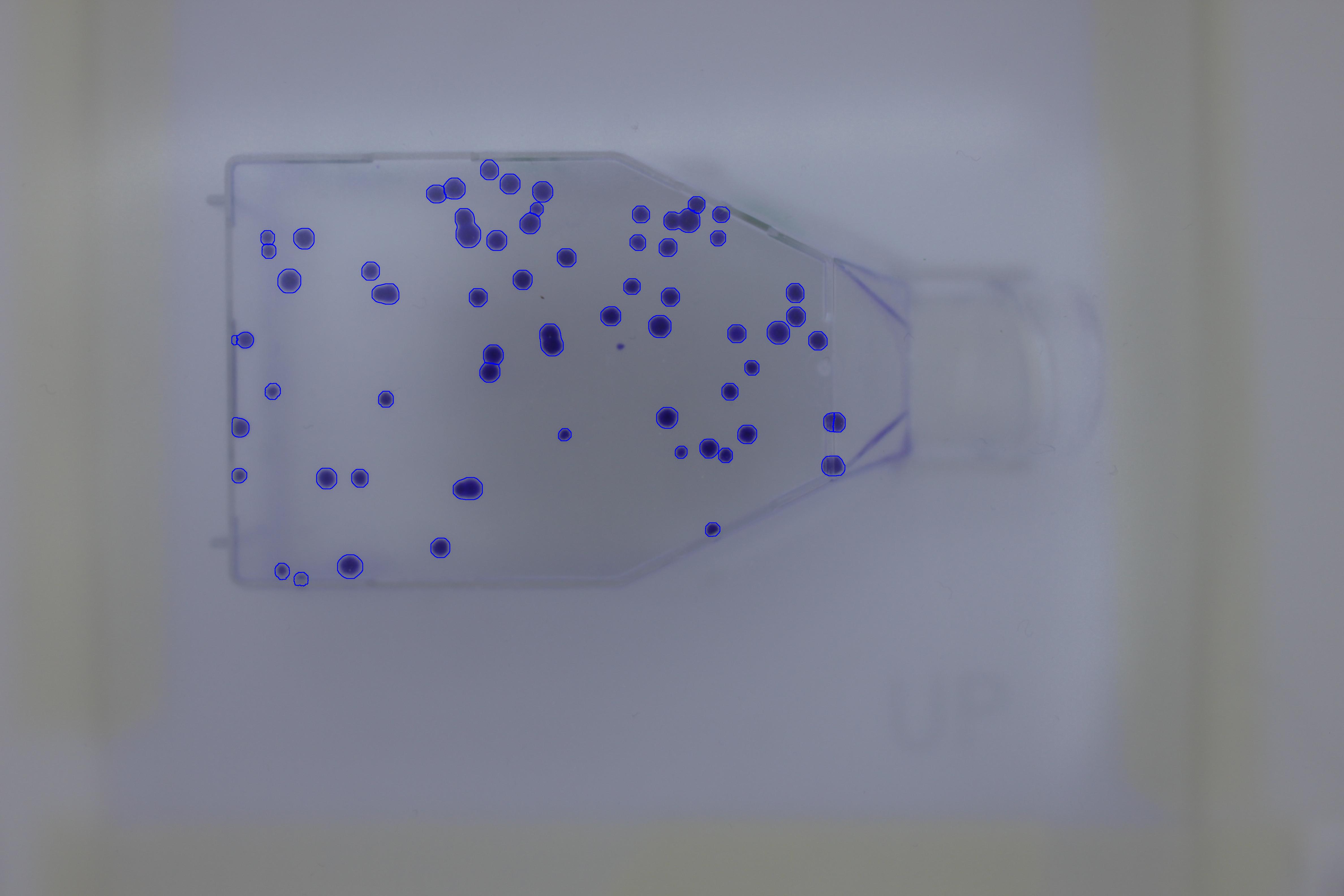

Supplement: S1 Comparison to others — (ZIP) [file pone.0205823.s007.zip › S1 Comparison to others/AutoCellSeg/171214 V79 Flask/4_seg.jpg]

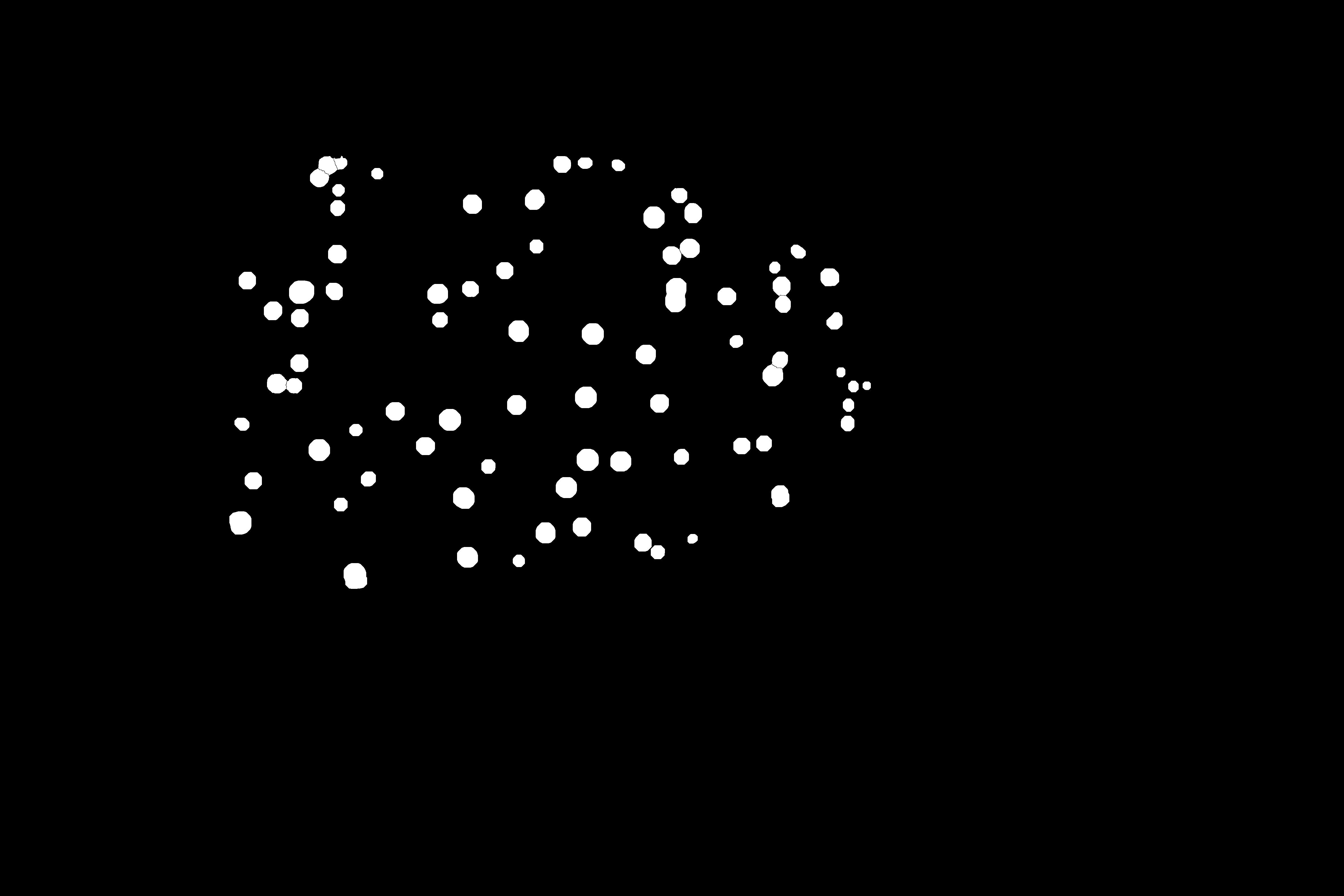

Supplement: S1 Comparison to others — (ZIP) [file pone.0205823.s007.zip › S1 Comparison to others/AutoCellSeg/171214 V79 Flask/5_mask.jpg]

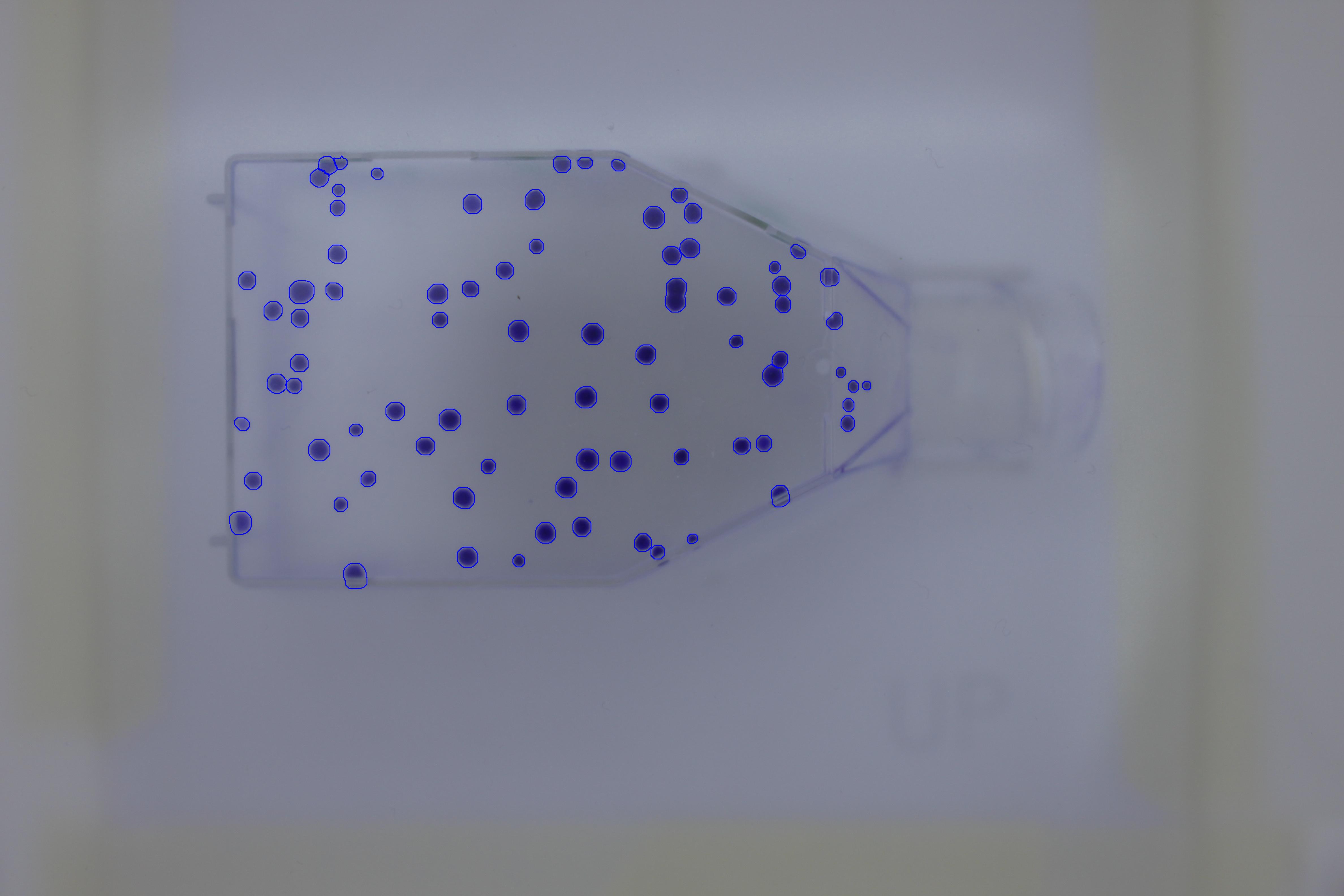

Supplement: S1 Comparison to others — (ZIP) [file pone.0205823.s007.zip › S1 Comparison to others/AutoCellSeg/171214 V79 Flask/5_seg.jpg]

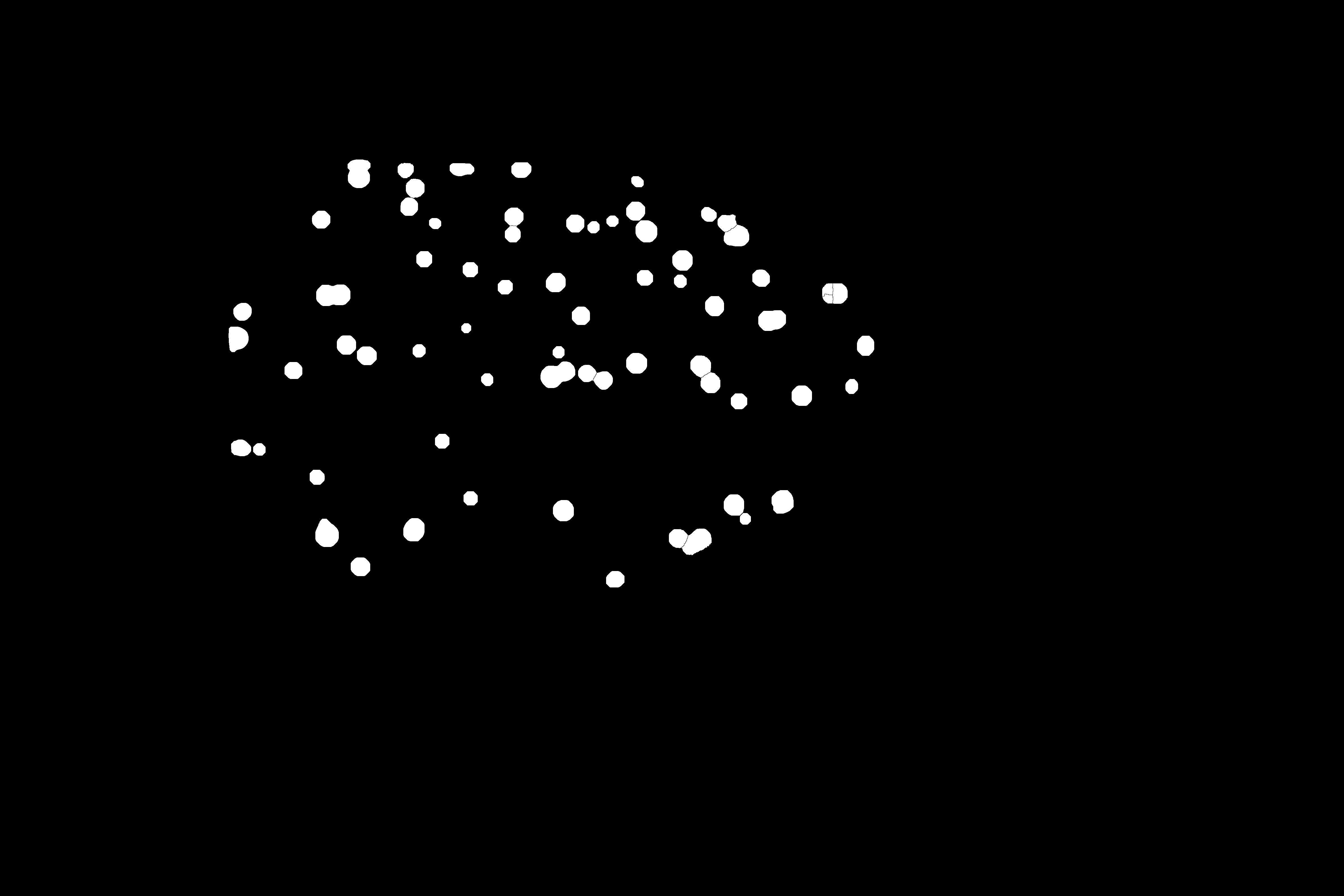

Supplement: S1 Comparison to others — (ZIP) [file pone.0205823.s007.zip › S1 Comparison to others/AutoCellSeg/171214 V79 Flask/6_mask.jpg]

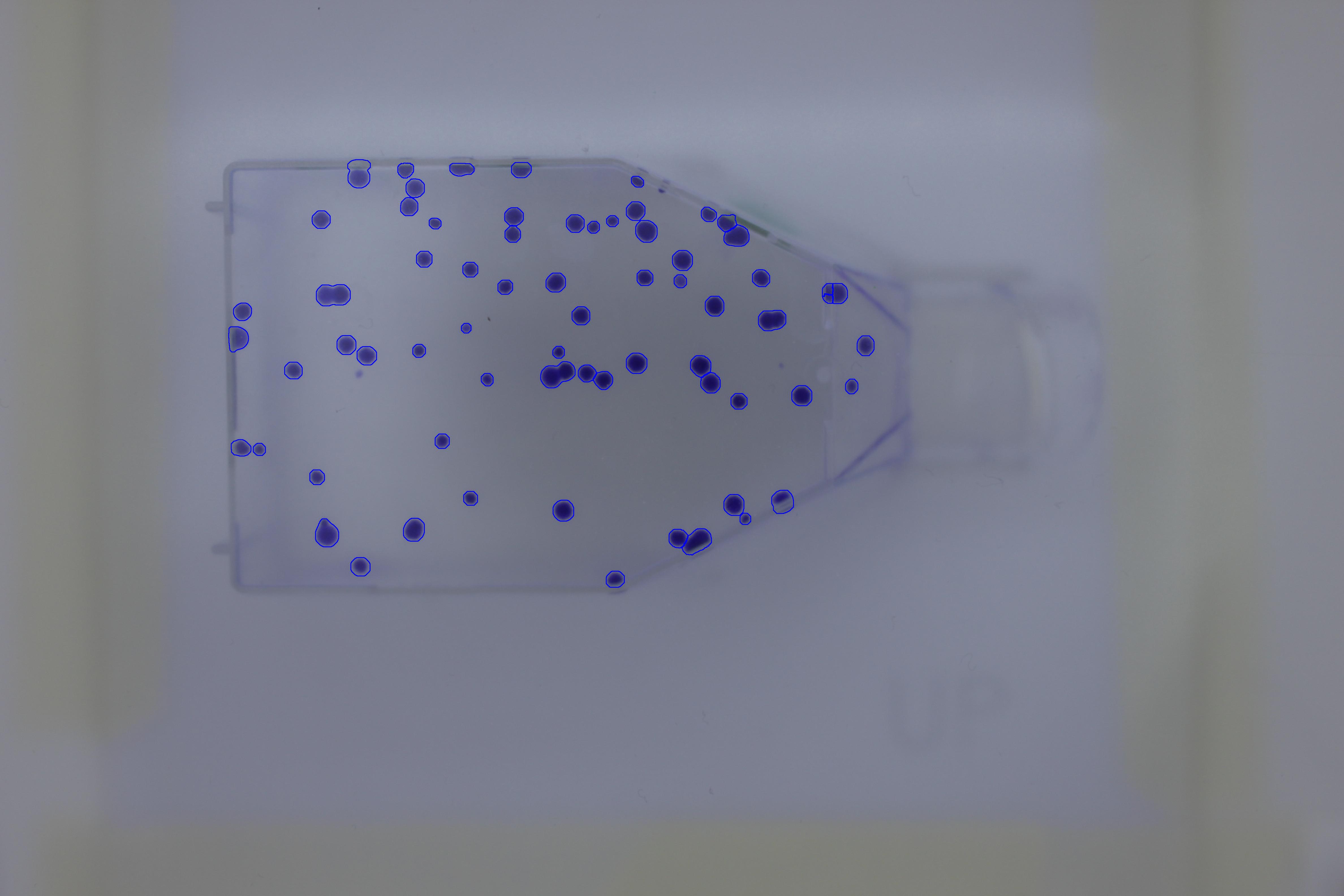

Supplement: S1 Comparison to others — (ZIP) [file pone.0205823.s007.zip › S1 Comparison to others/AutoCellSeg/171214 V79 Flask/6_seg.jpg]

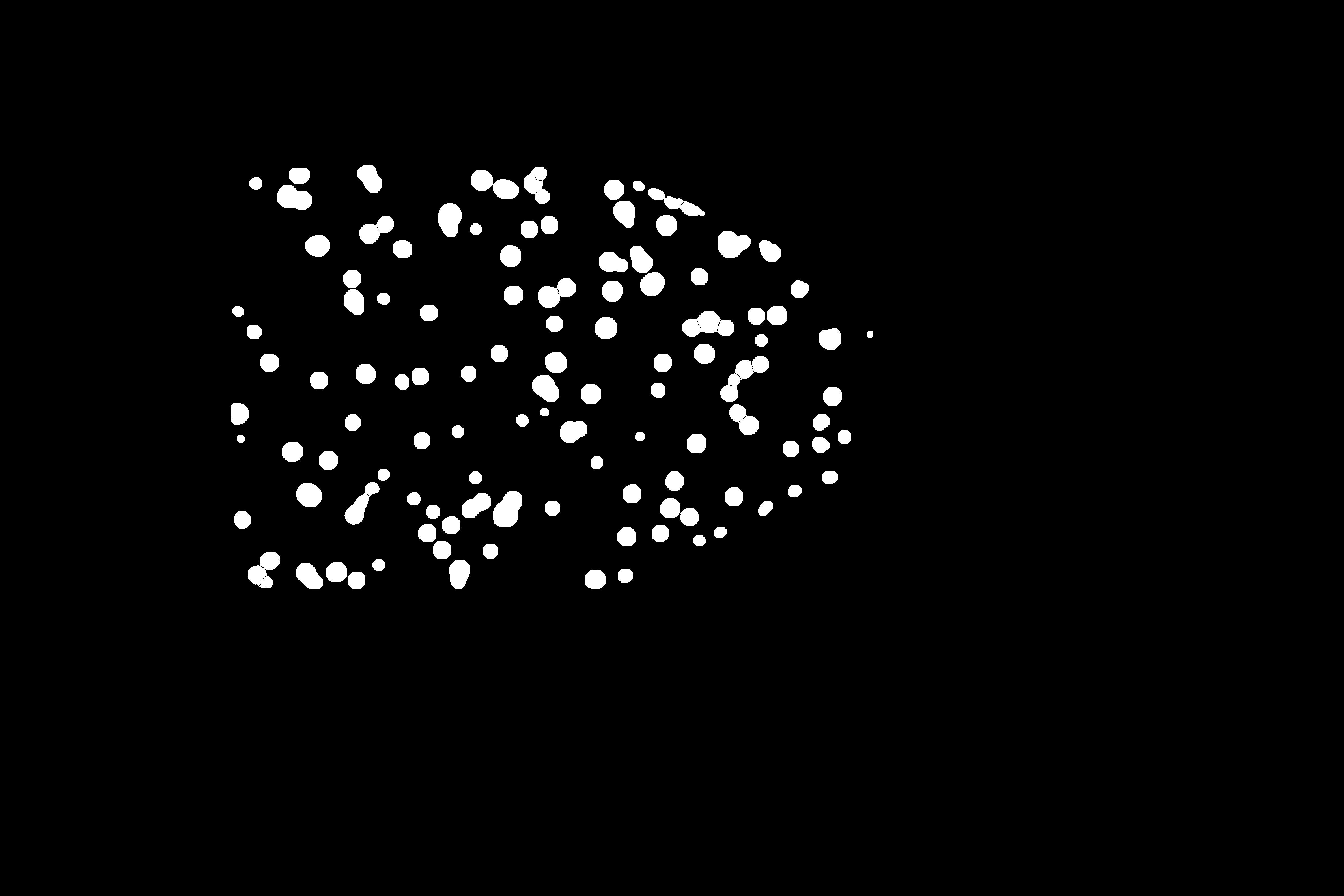

Supplement: S1 Comparison to others — (ZIP) [file pone.0205823.s007.zip › S1 Comparison to others/AutoCellSeg/171214 V79 Flask/7_mask.jpg]

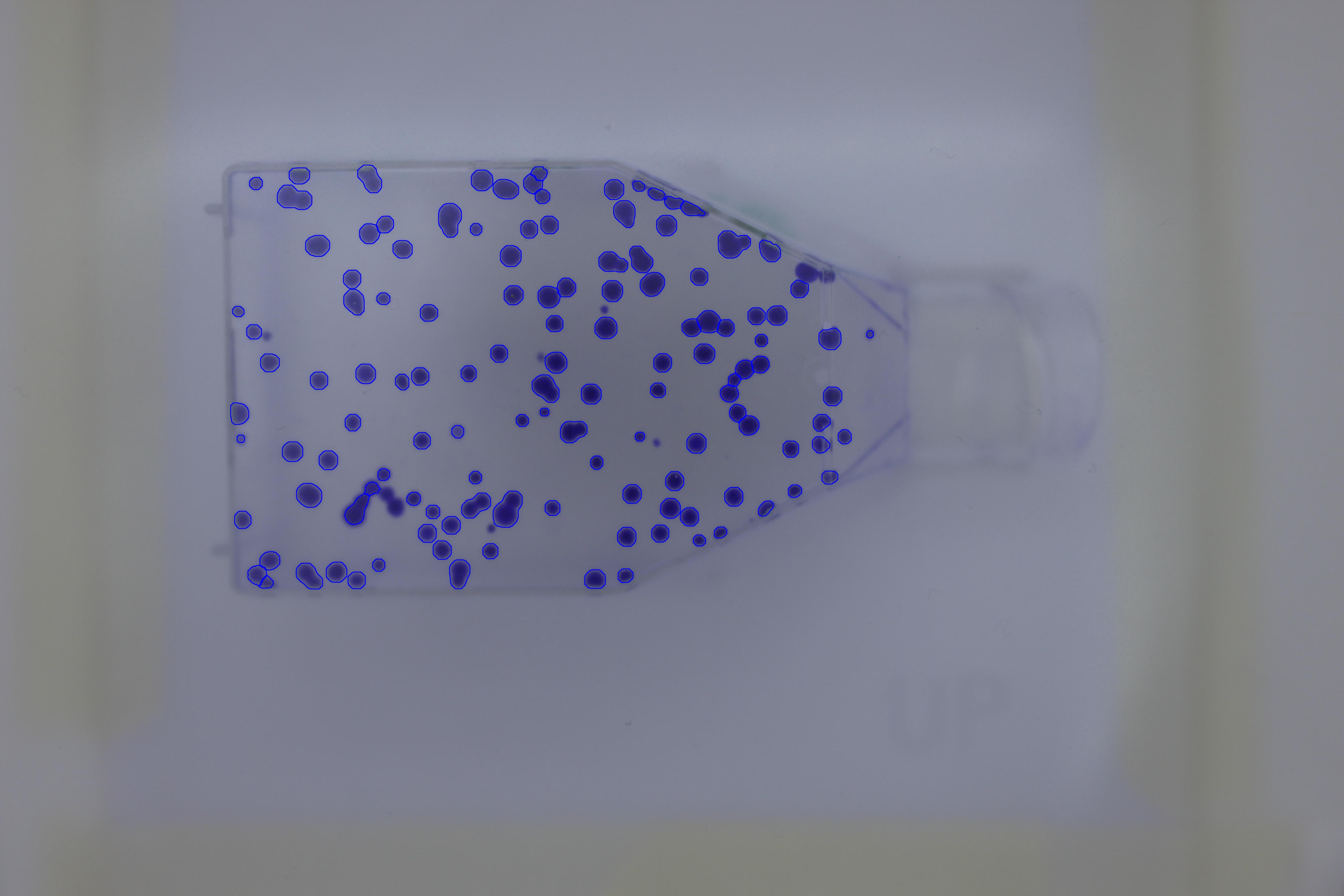

Supplement: S1 Comparison to others — (ZIP) [file pone.0205823.s007.zip › S1 Comparison to others/AutoCellSeg/171214 V79 Flask/7_seg.jpg]

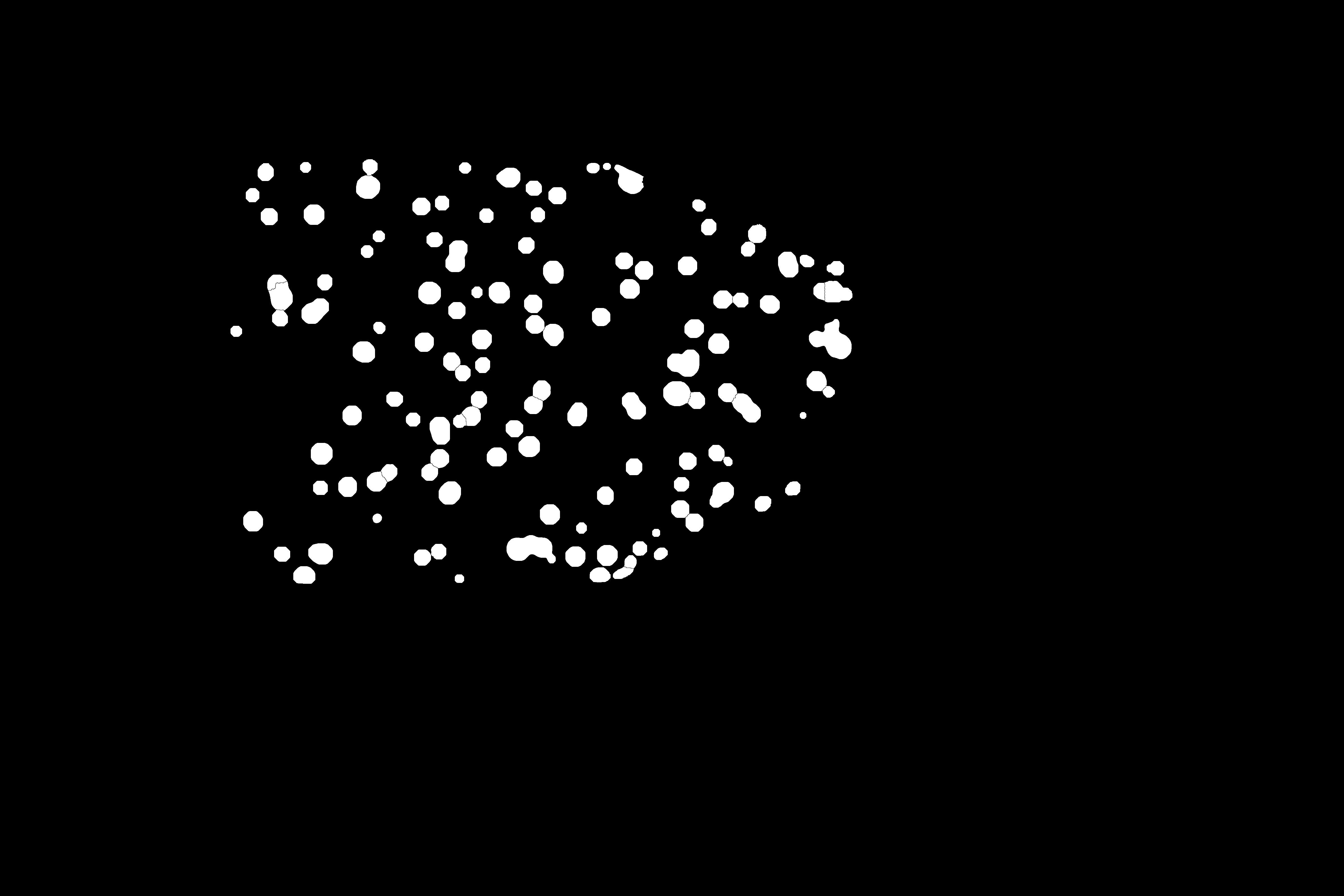

Supplement: S1 Comparison to others — (ZIP) [file pone.0205823.s007.zip › S1 Comparison to others/AutoCellSeg/171214 V79 Flask/8_mask.jpg]

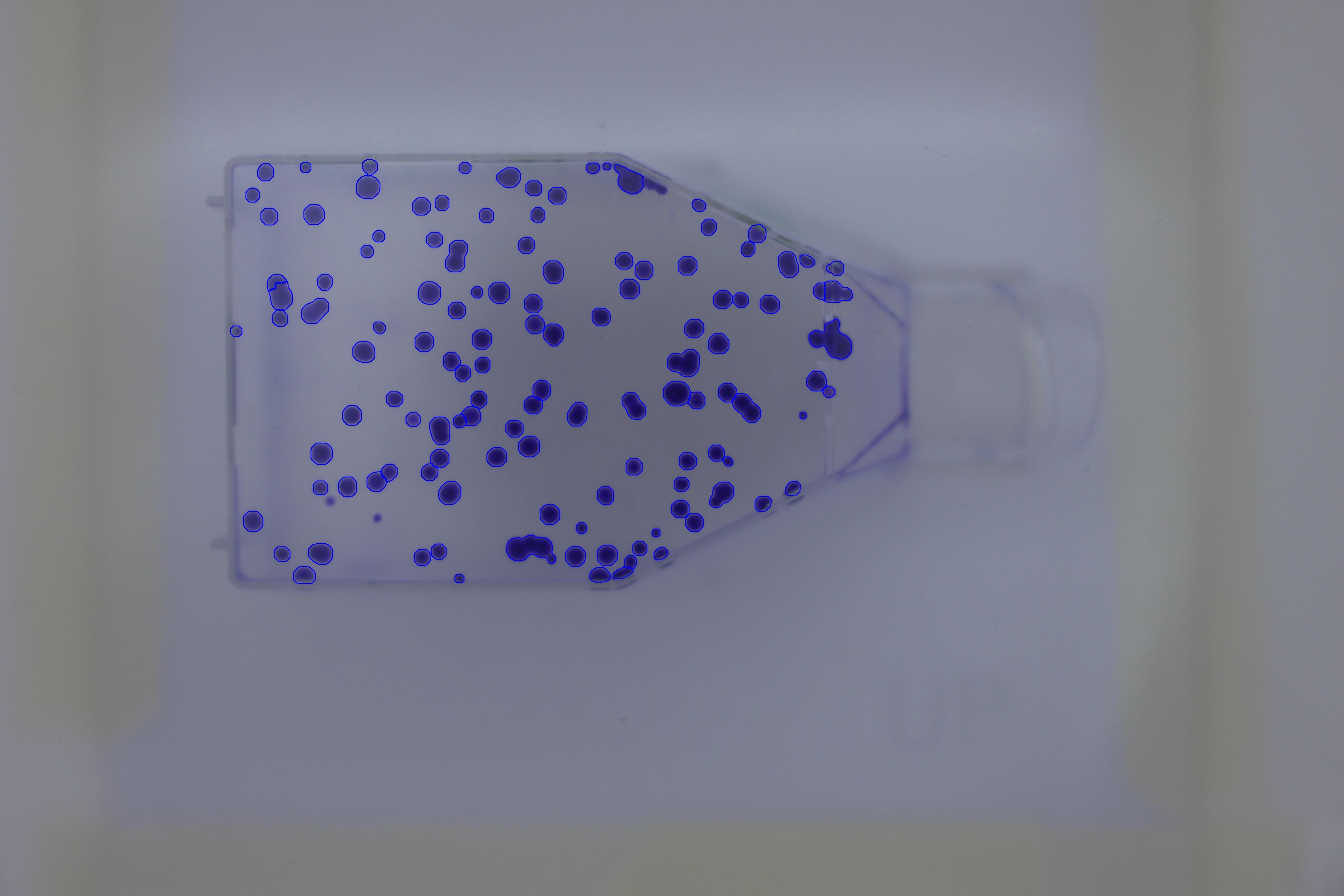

Supplement: S1 Comparison to others — (ZIP) [file pone.0205823.s007.zip › S1 Comparison to others/AutoCellSeg/171214 V79 Flask/8_seg.jpg]

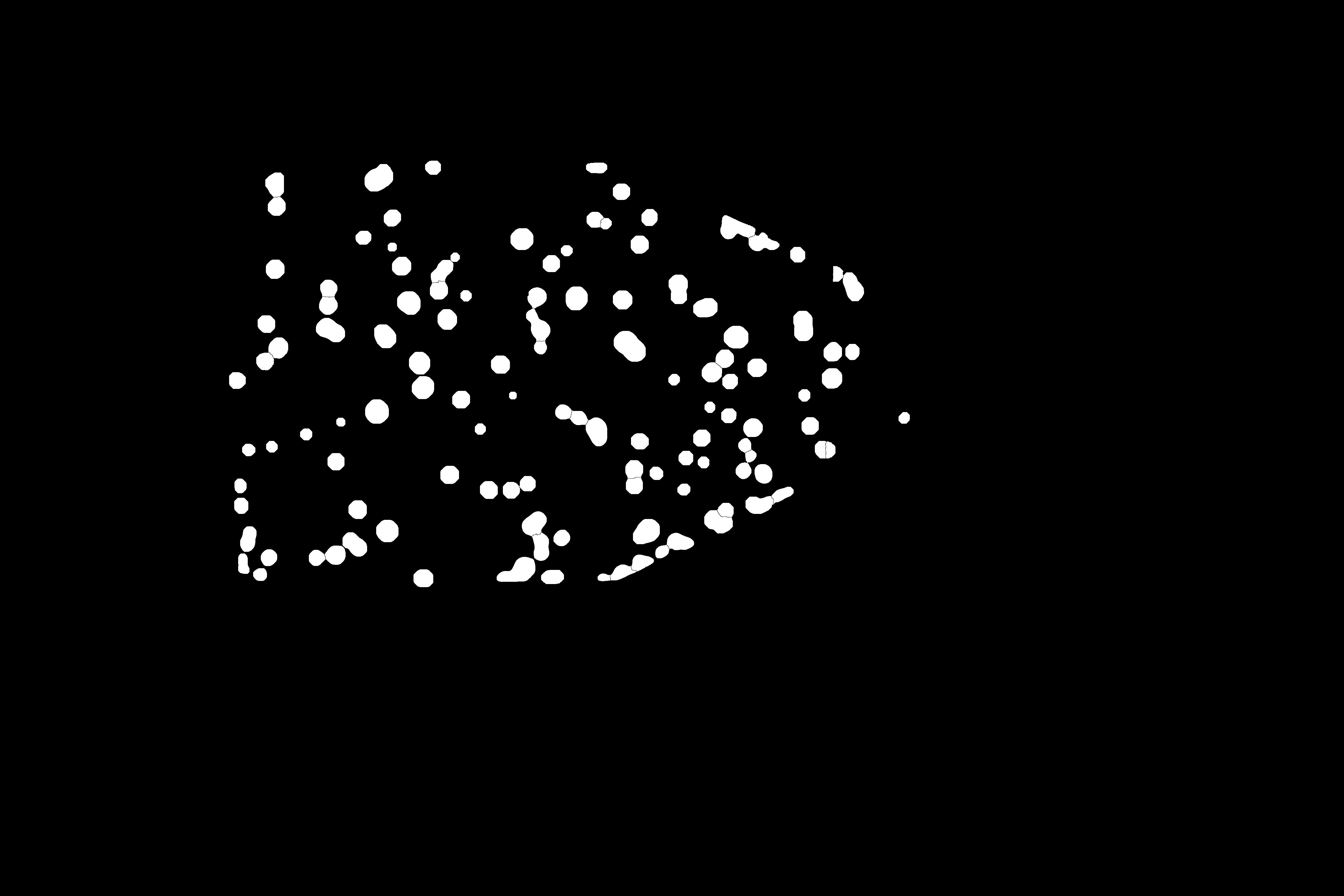

Supplement: S1 Comparison to others — (ZIP) [file pone.0205823.s007.zip › S1 Comparison to others/AutoCellSeg/171214 V79 Flask/9_mask.jpg]

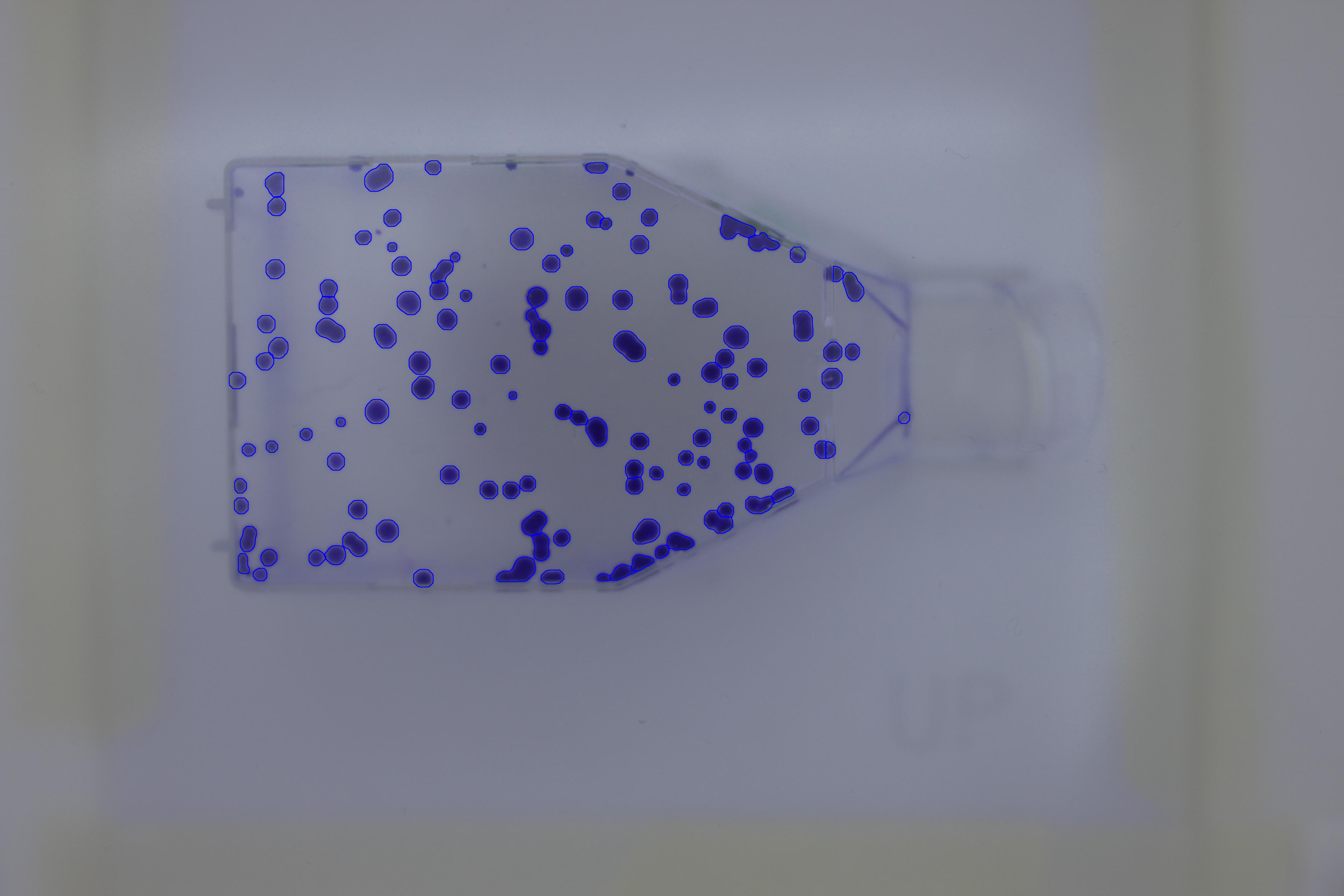

Supplement: S1 Comparison to others — (ZIP) [file pone.0205823.s007.zip › S1 Comparison to others/AutoCellSeg/171214 V79 Flask/9_seg.jpg]

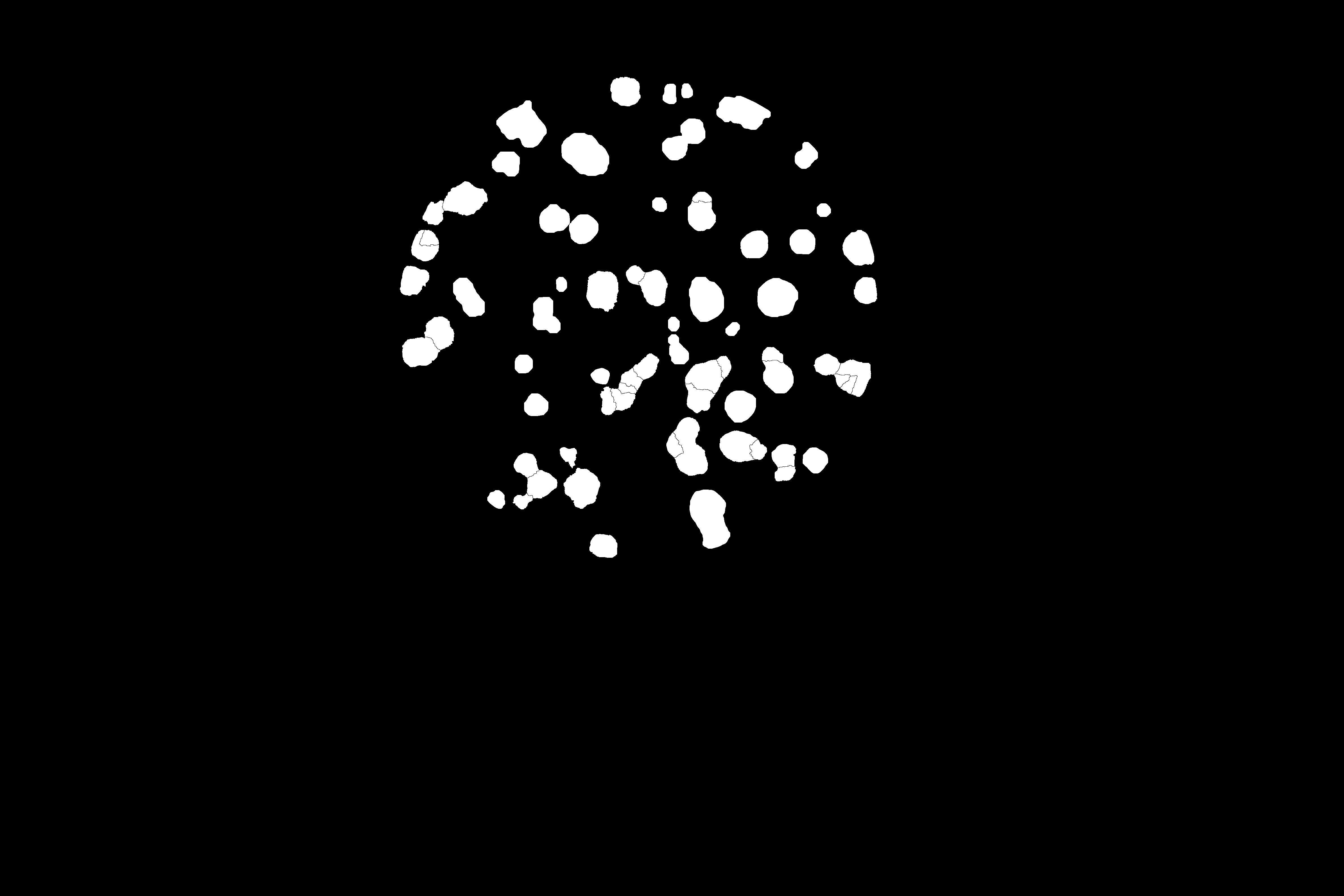

Supplement: S1 Comparison to others — (ZIP) [file pone.0205823.s007.zip › S1 Comparison to others/AutoCellSeg/180501 HeLa Dish/10_mask.jpg]

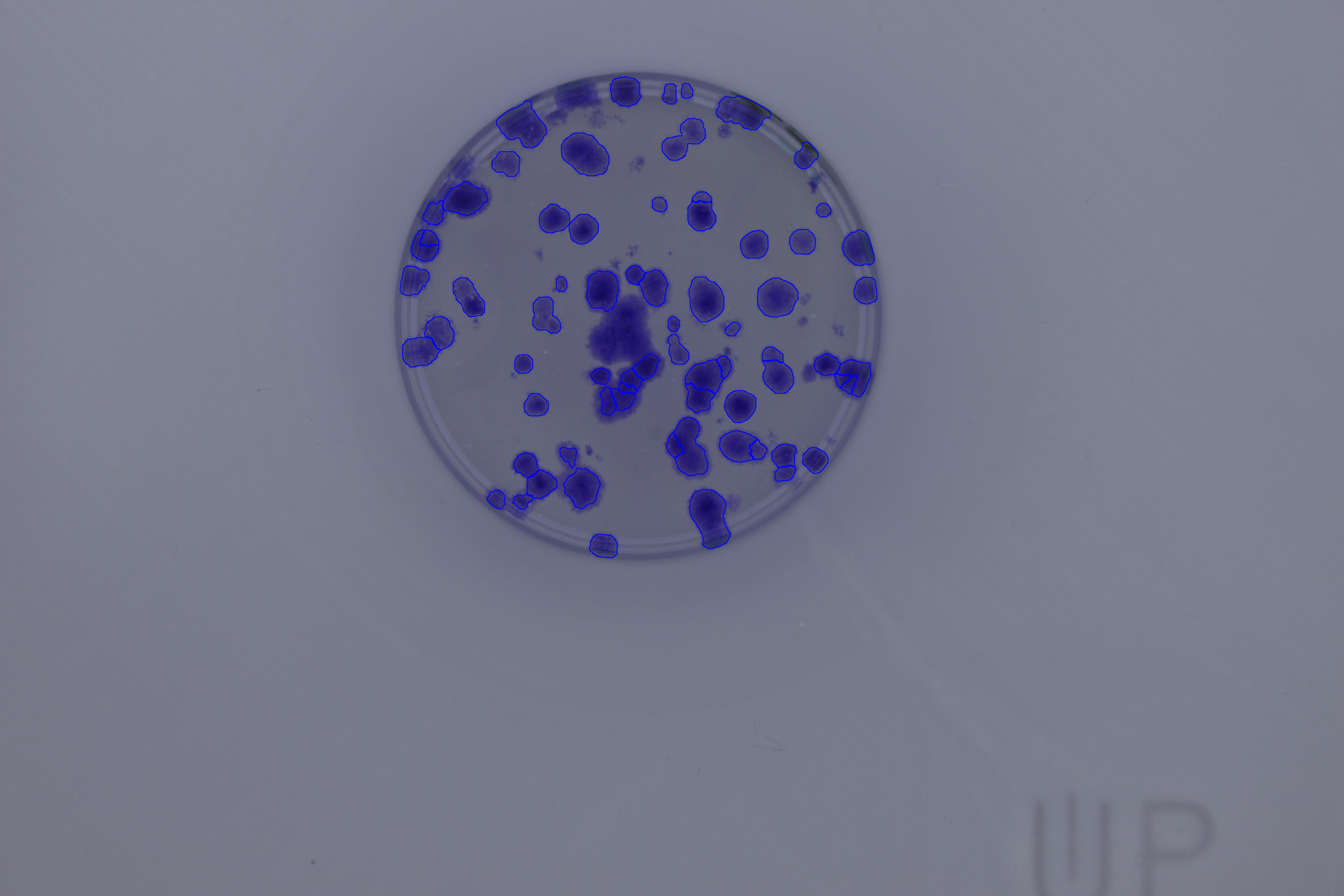

Supplement: S1 Comparison to others — (ZIP) [file pone.0205823.s007.zip › S1 Comparison to others/AutoCellSeg/180501 HeLa Dish/10_seg.jpg]

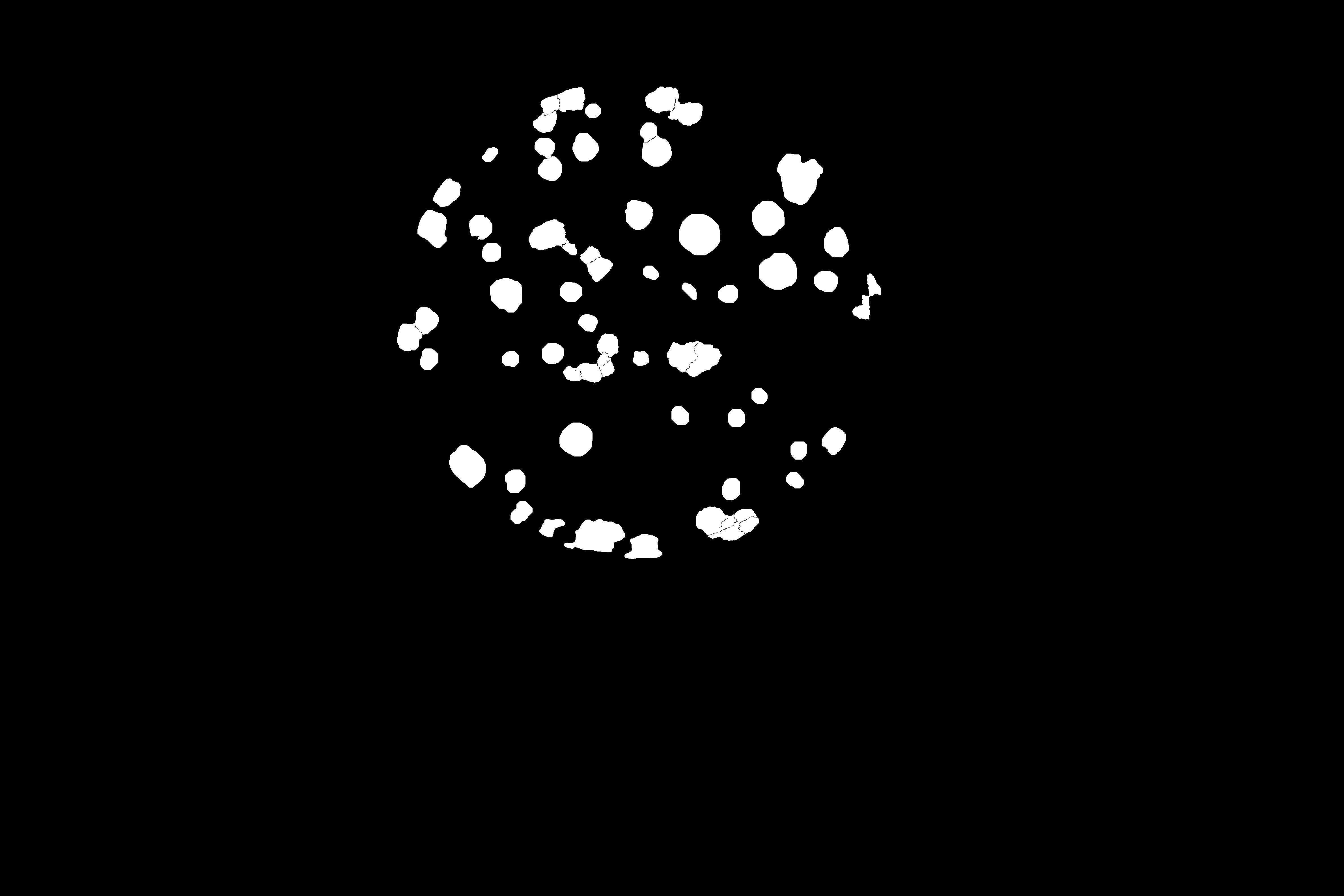

Supplement: S1 Comparison to others — (ZIP) [file pone.0205823.s007.zip › S1 Comparison to others/AutoCellSeg/180501 HeLa Dish/11_mask.jpg]

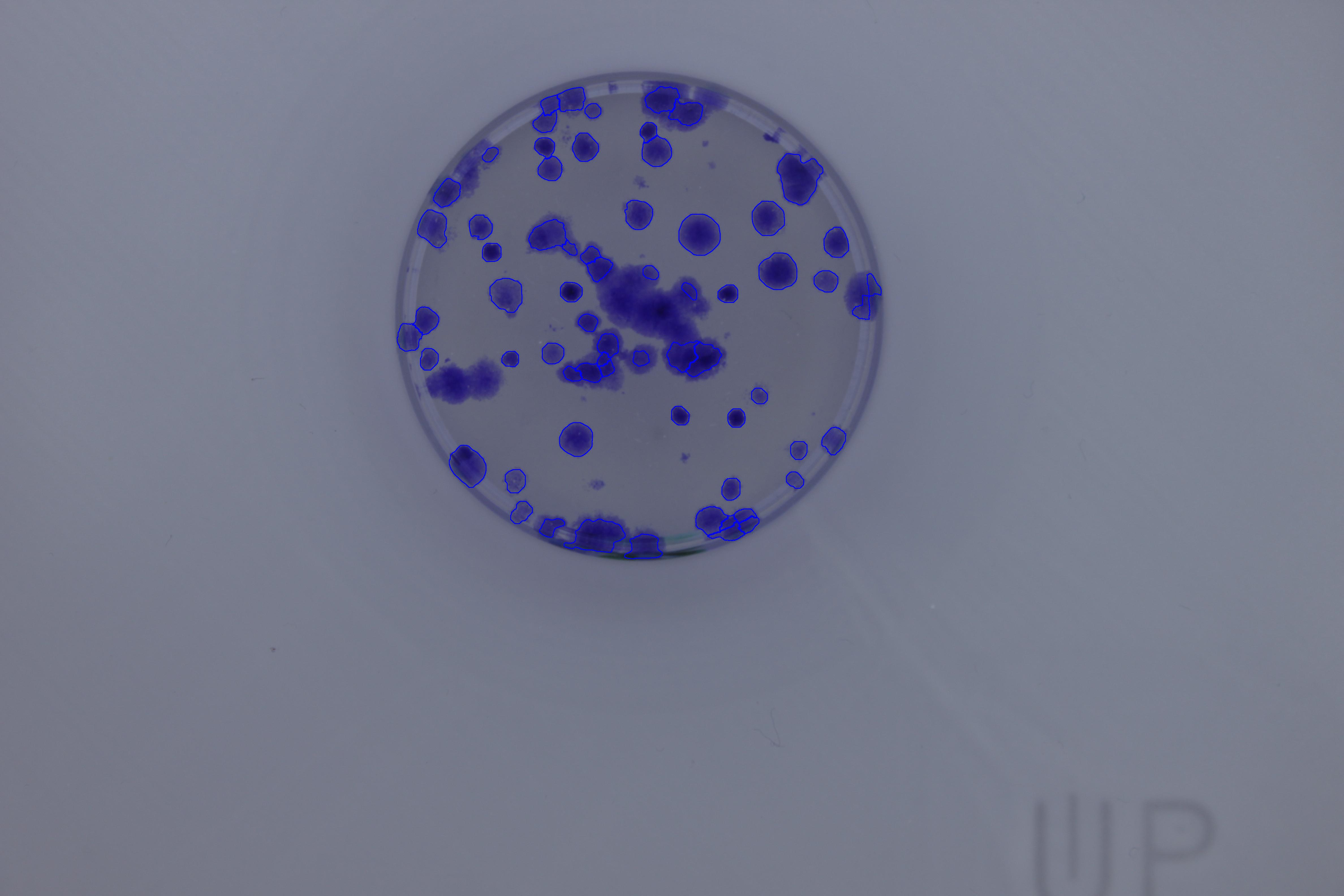

Supplement: S1 Comparison to others — (ZIP) [file pone.0205823.s007.zip › S1 Comparison to others/AutoCellSeg/180501 HeLa Dish/11_seg.jpg]

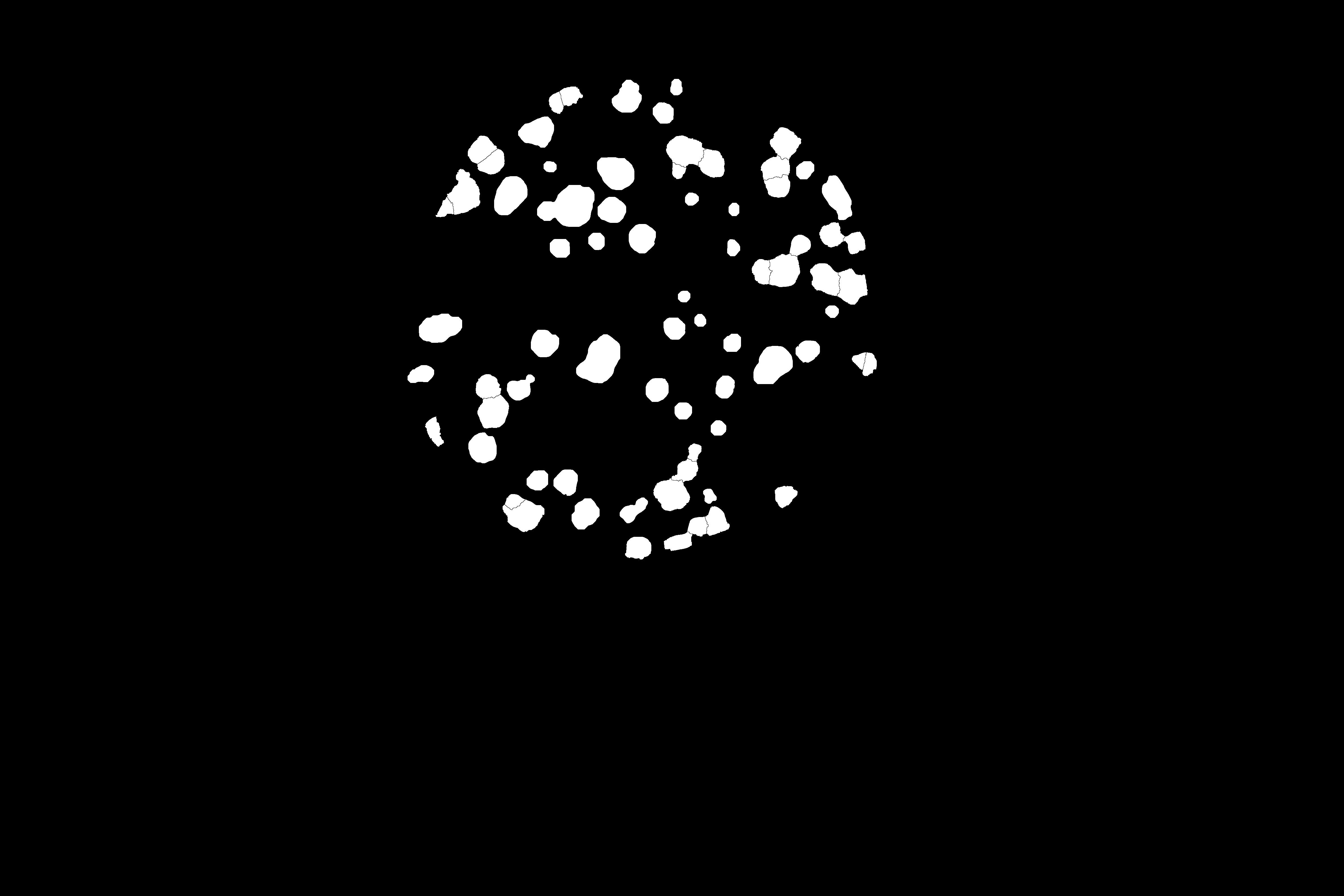

Supplement: S1 Comparison to others — (ZIP) [file pone.0205823.s007.zip › S1 Comparison to others/AutoCellSeg/180501 HeLa Dish/12_mask.jpg]

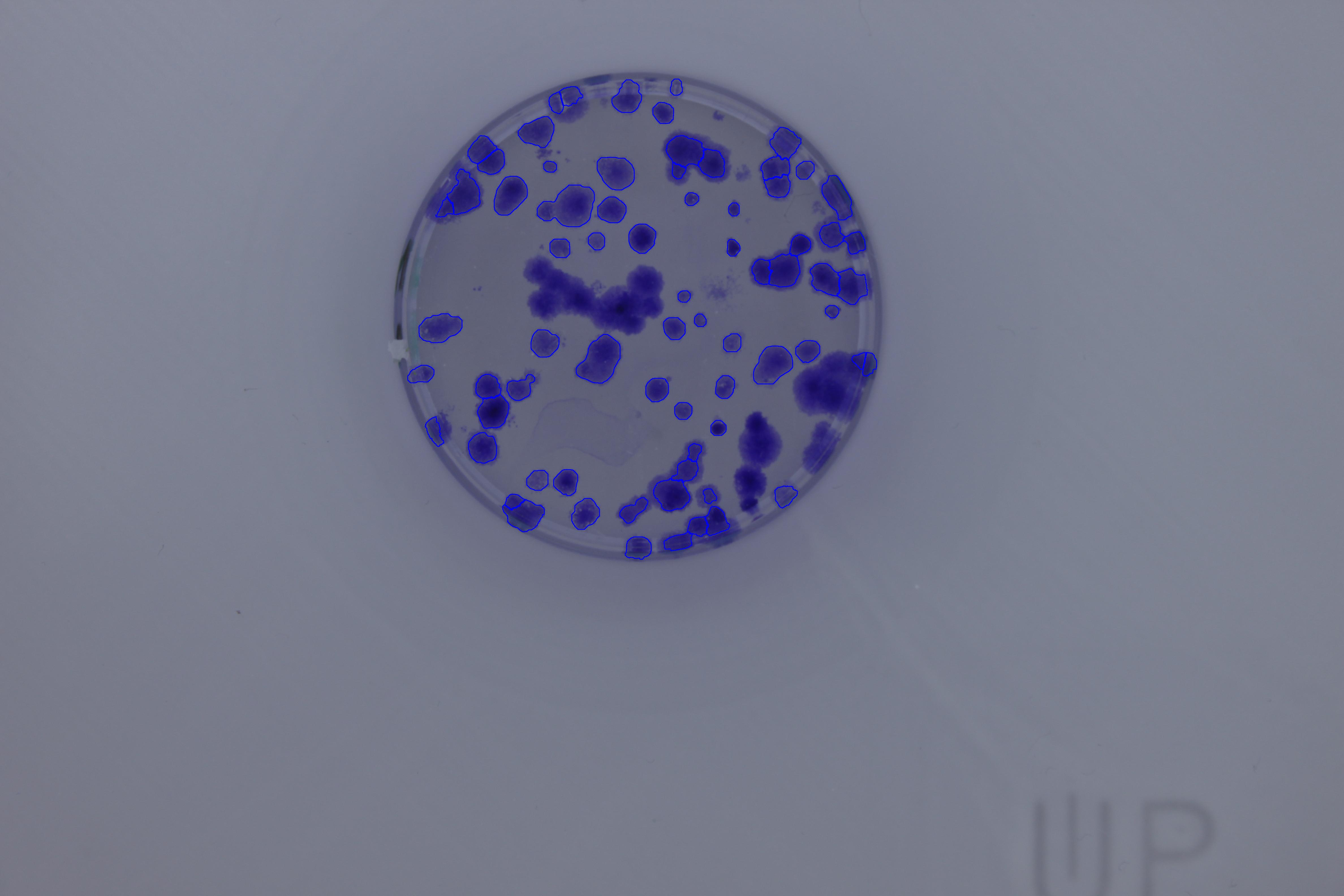

Supplement: S1 Comparison to others — (ZIP) [file pone.0205823.s007.zip › S1 Comparison to others/AutoCellSeg/180501 HeLa Dish/12_seg.jpg]

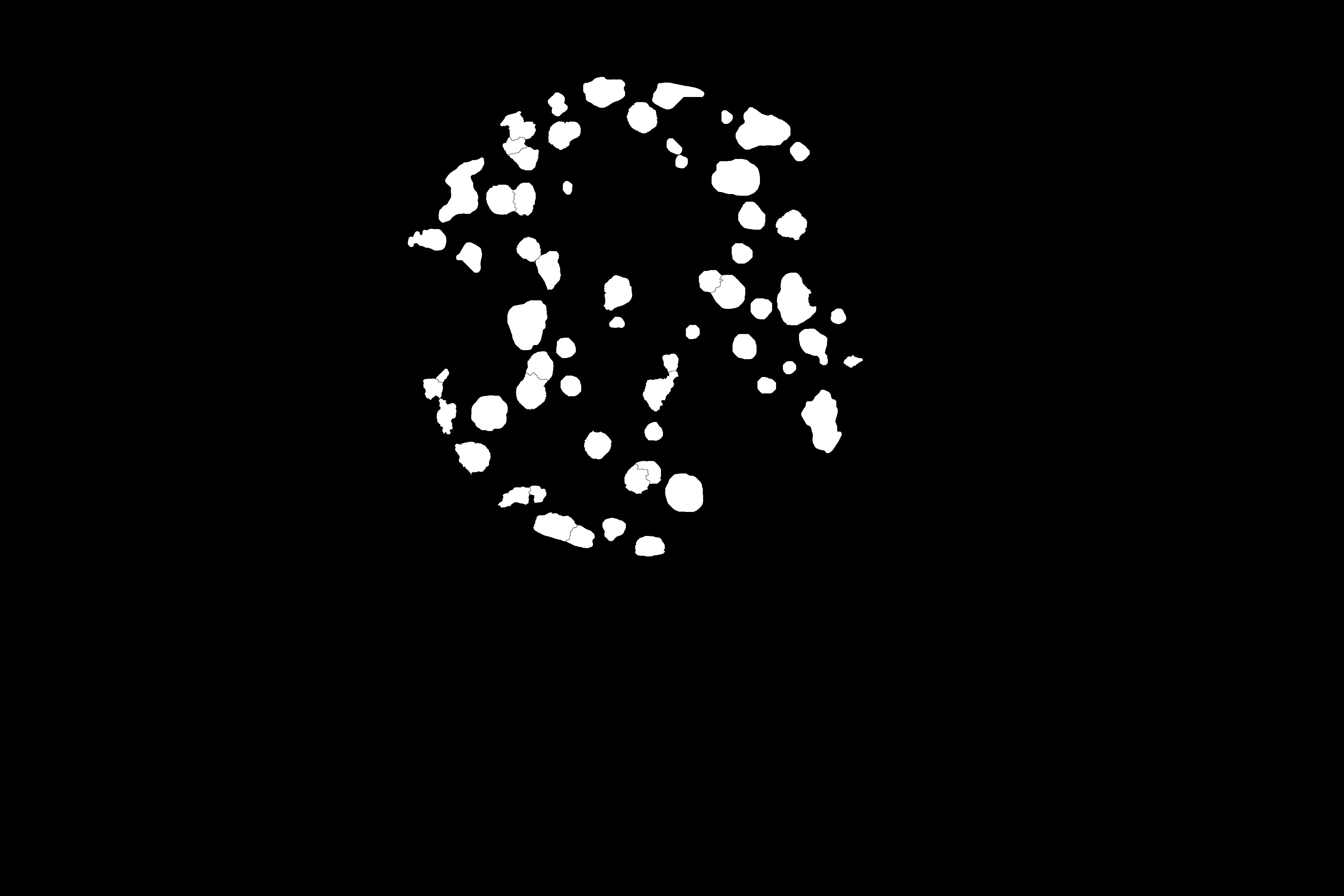

Supplement: S1 Comparison to others — (ZIP) [file pone.0205823.s007.zip › S1 Comparison to others/AutoCellSeg/180501 HeLa Dish/13_mask.jpg]

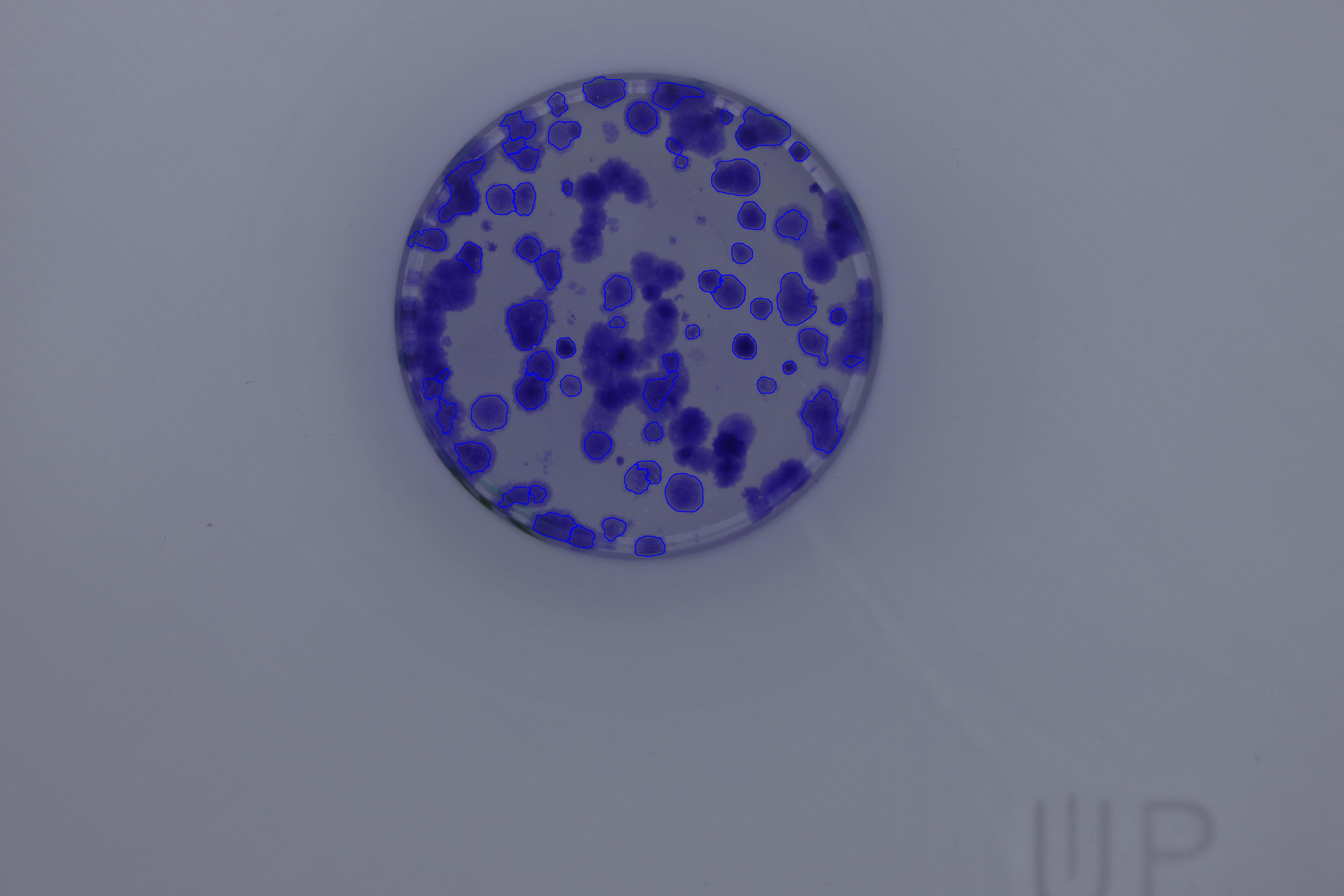

Supplement: S1 Comparison to others — (ZIP) [file pone.0205823.s007.zip › S1 Comparison to others/AutoCellSeg/180501 HeLa Dish/13_seg.jpg]

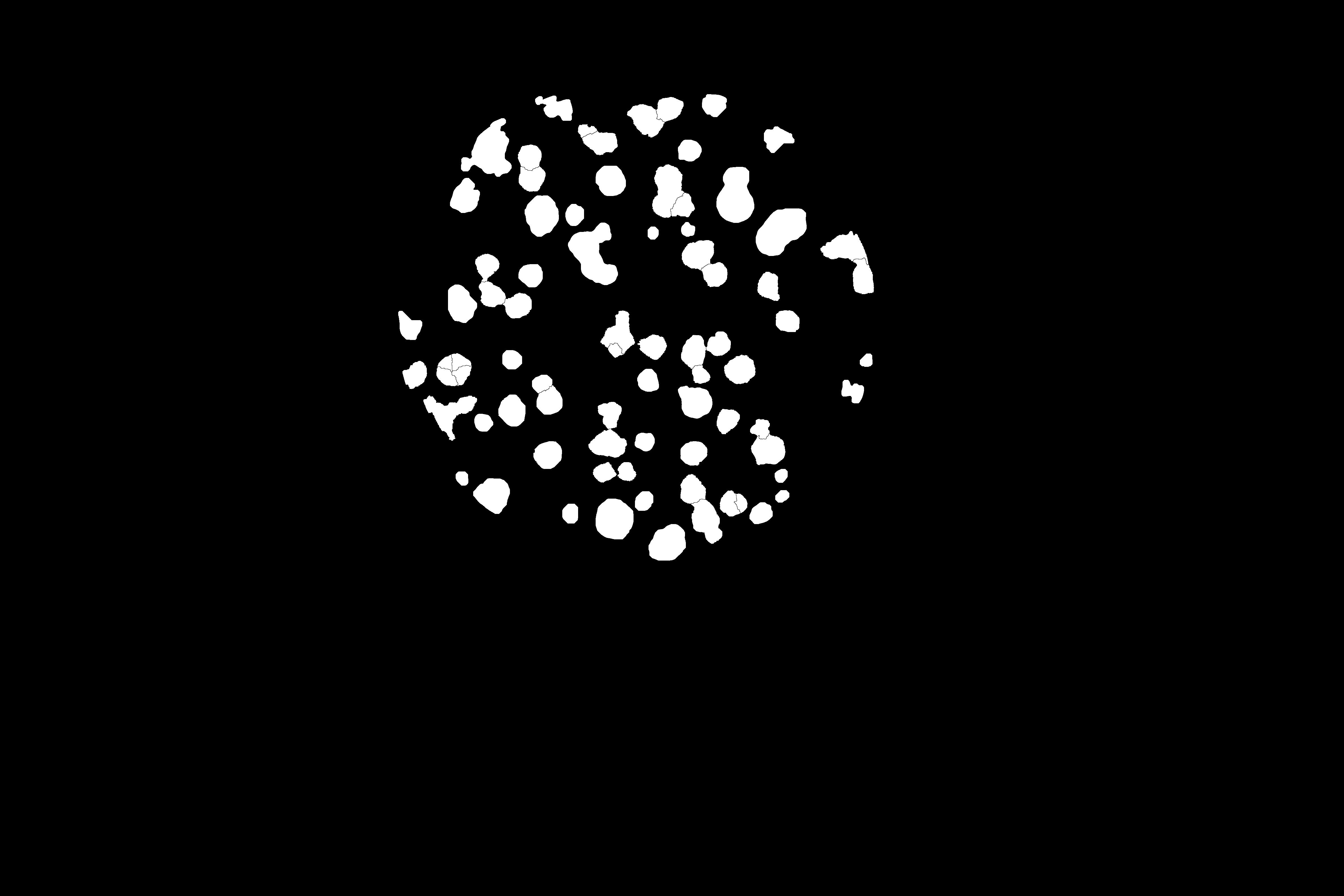

Supplement: S1 Comparison to others — (ZIP) [file pone.0205823.s007.zip › S1 Comparison to others/AutoCellSeg/180501 HeLa Dish/14_mask.jpg]

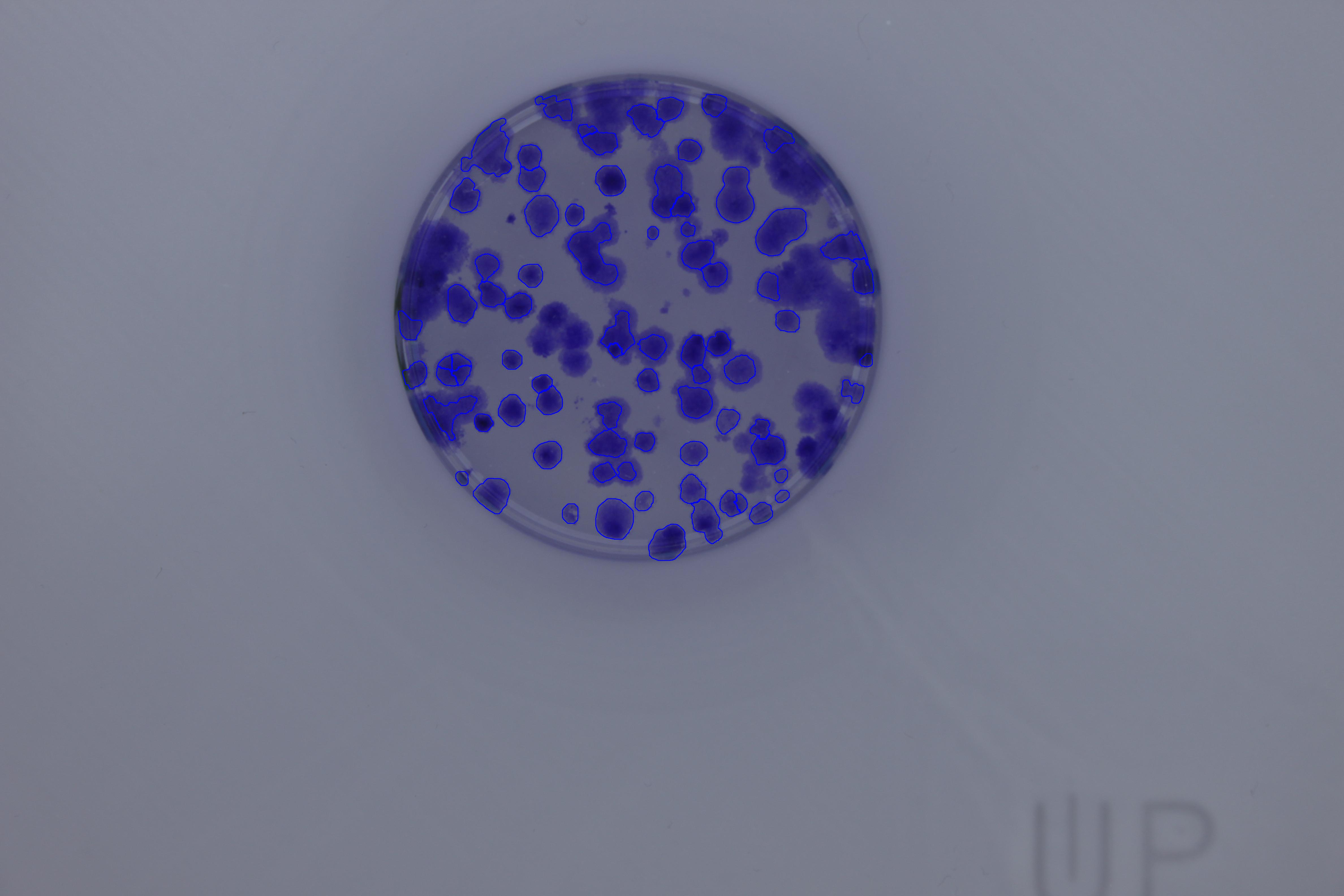

Supplement: S1 Comparison to others — (ZIP) [file pone.0205823.s007.zip › S1 Comparison to others/AutoCellSeg/180501 HeLa Dish/14_seg.jpg]

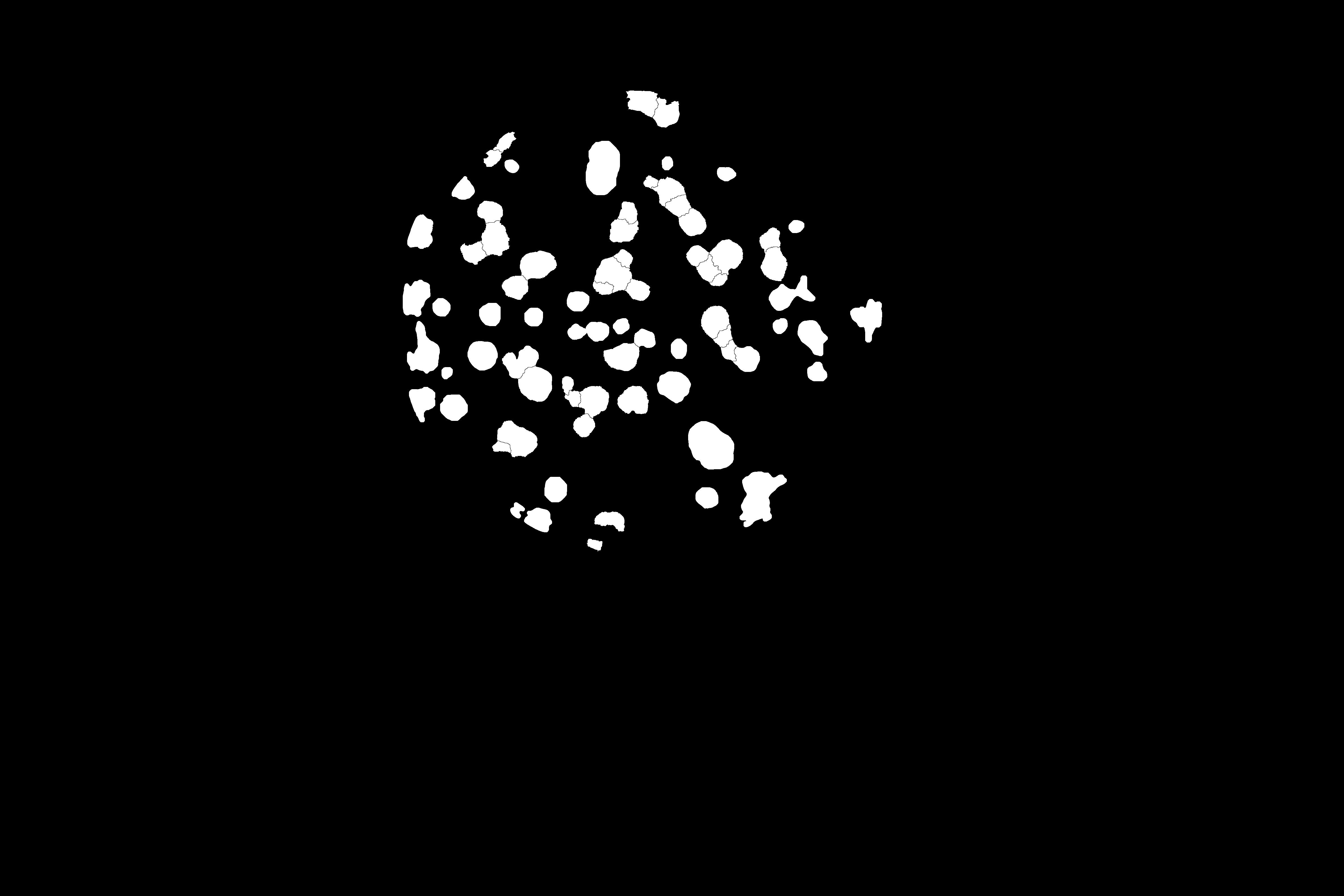

Supplement: S1 Comparison to others — (ZIP) [file pone.0205823.s007.zip › S1 Comparison to others/AutoCellSeg/180501 HeLa Dish/15_mask.jpg]

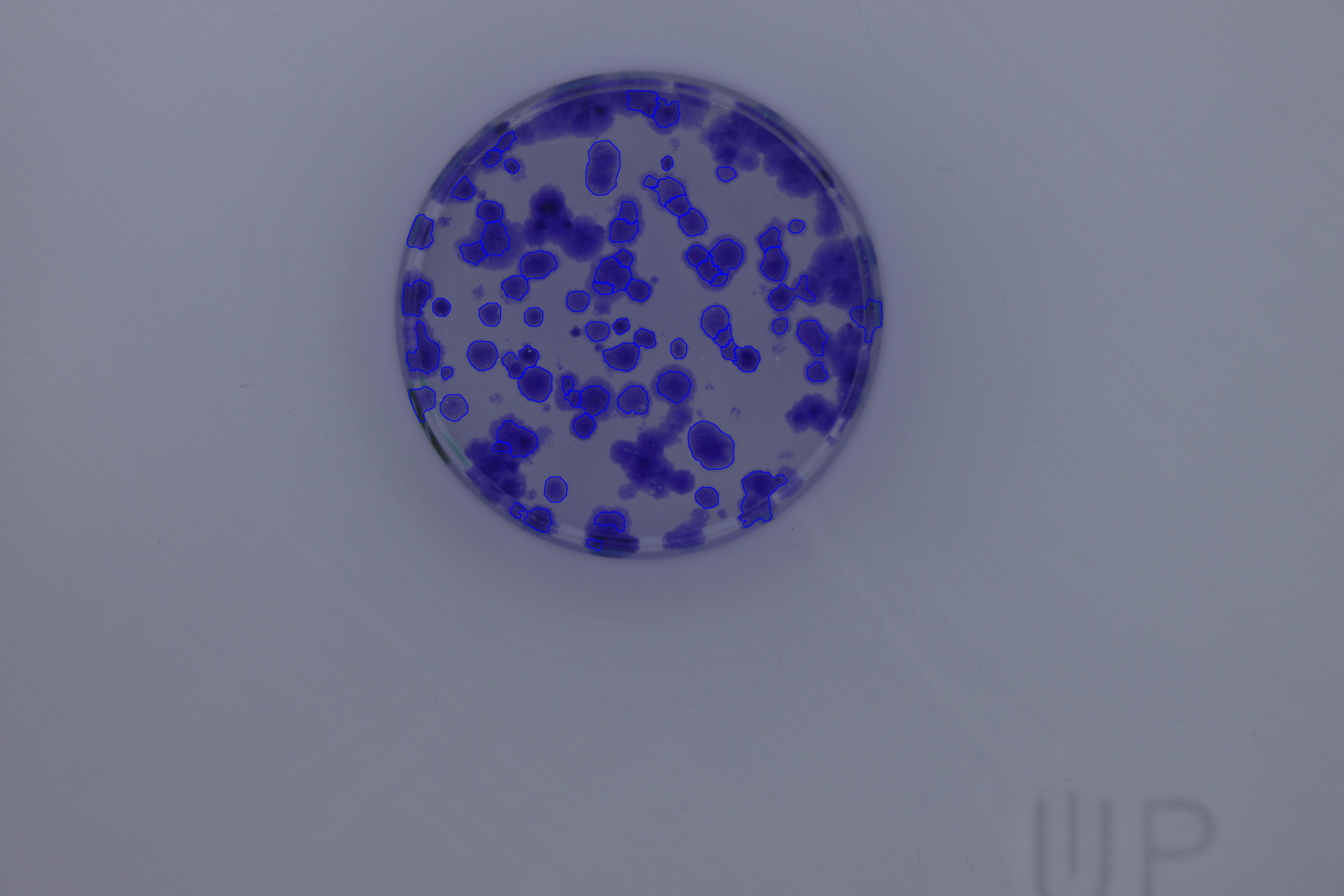

Supplement: S1 Comparison to others — (ZIP) [file pone.0205823.s007.zip › S1 Comparison to others/AutoCellSeg/180501 HeLa Dish/15_seg.jpg]

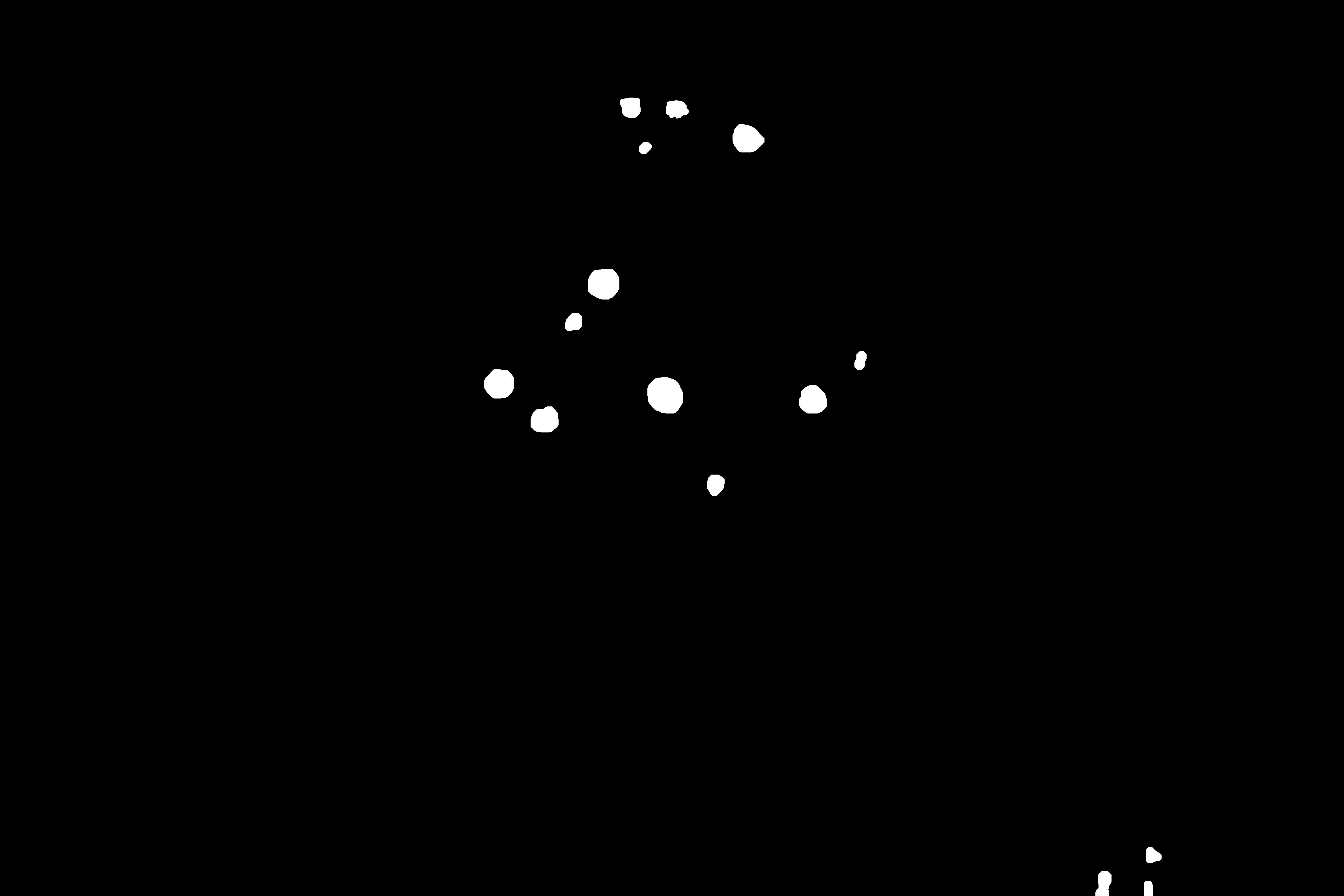

Supplement: S1 Comparison to others — (ZIP) [file pone.0205823.s007.zip › S1 Comparison to others/AutoCellSeg/180501 HeLa Dish/1_mask.jpg]

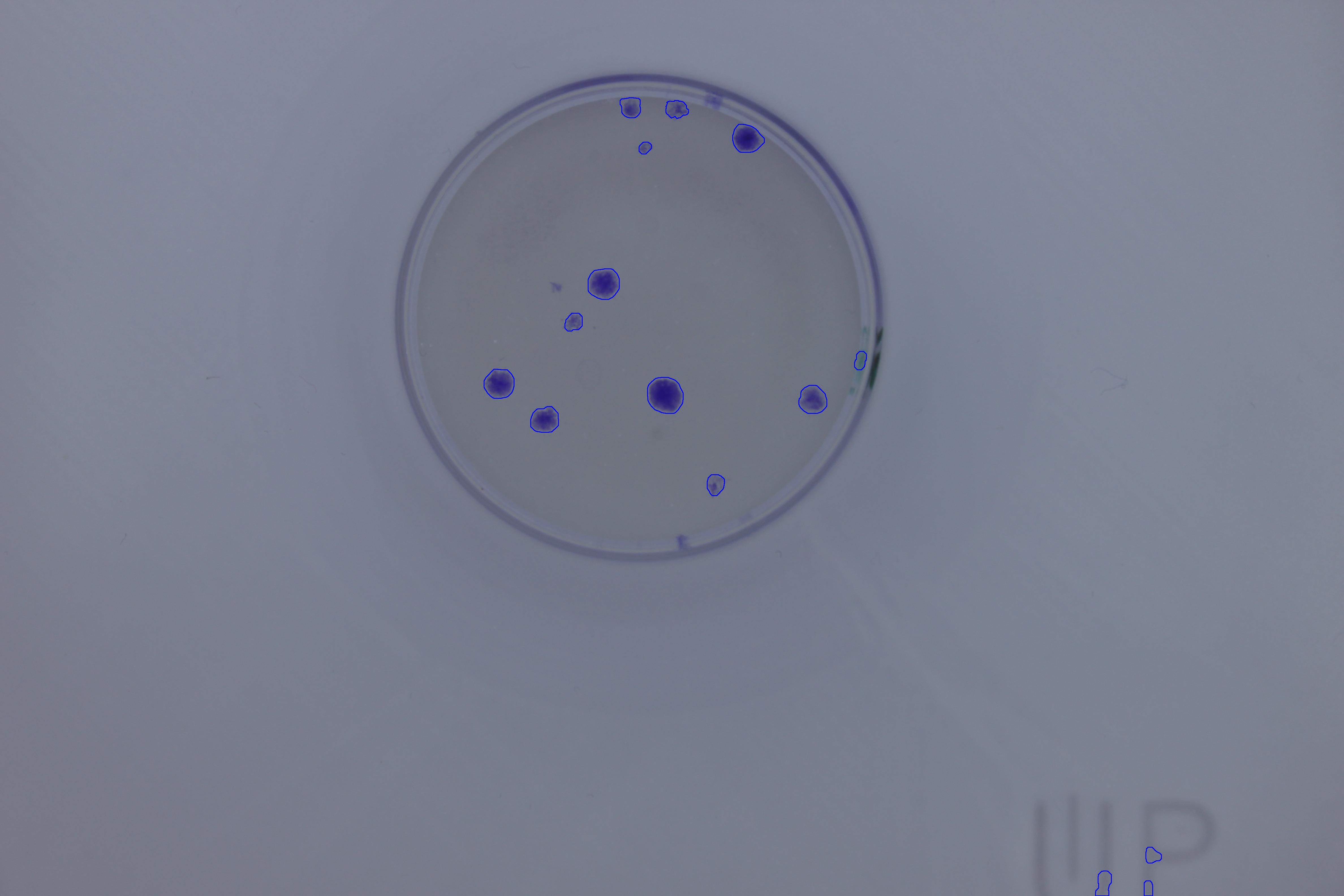

Supplement: S1 Comparison to others — (ZIP) [file pone.0205823.s007.zip › S1 Comparison to others/AutoCellSeg/180501 HeLa Dish/1_seg.jpg]

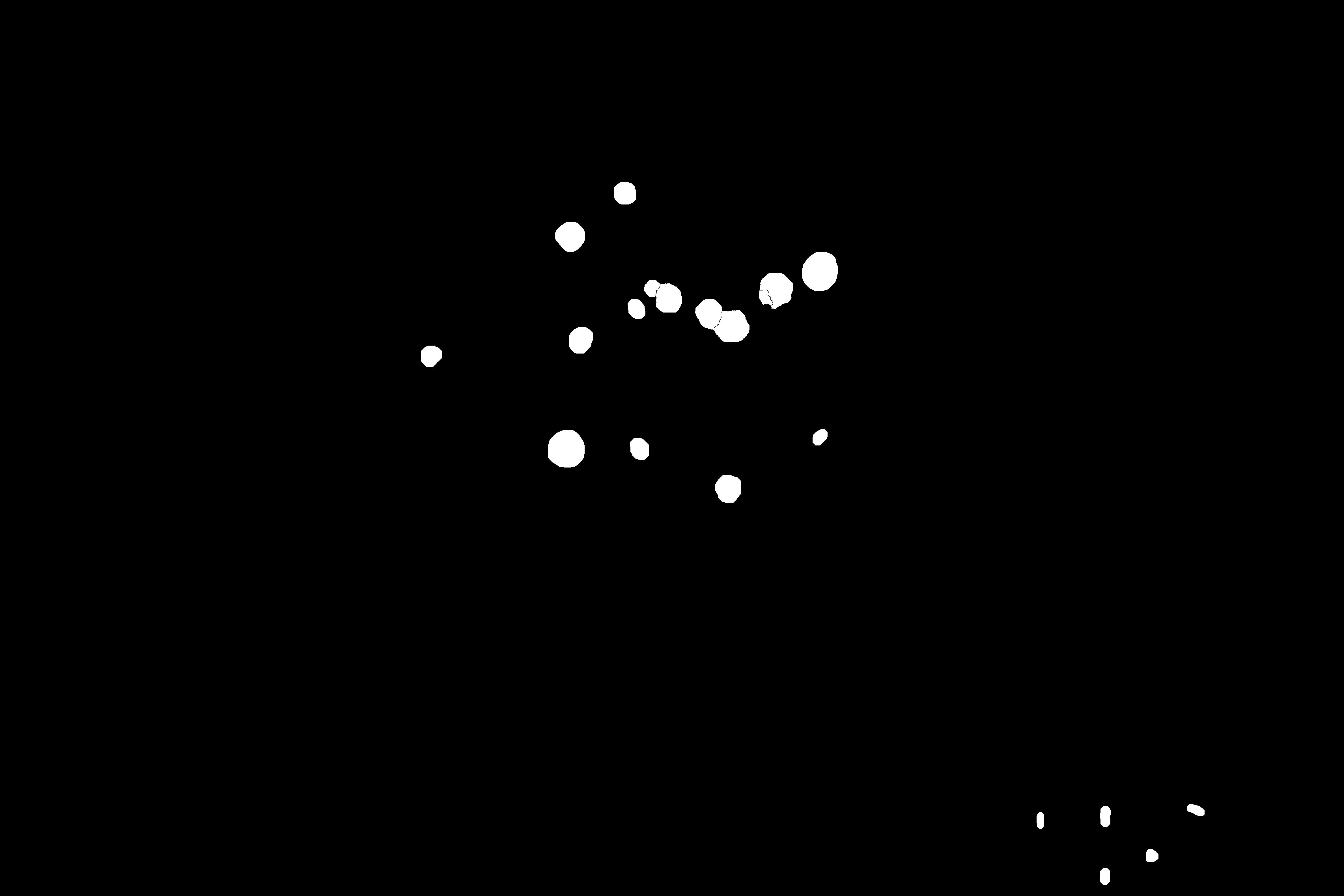

Supplement: S1 Comparison to others — (ZIP) [file pone.0205823.s007.zip › S1 Comparison to others/AutoCellSeg/180501 HeLa Dish/2_mask.jpg]

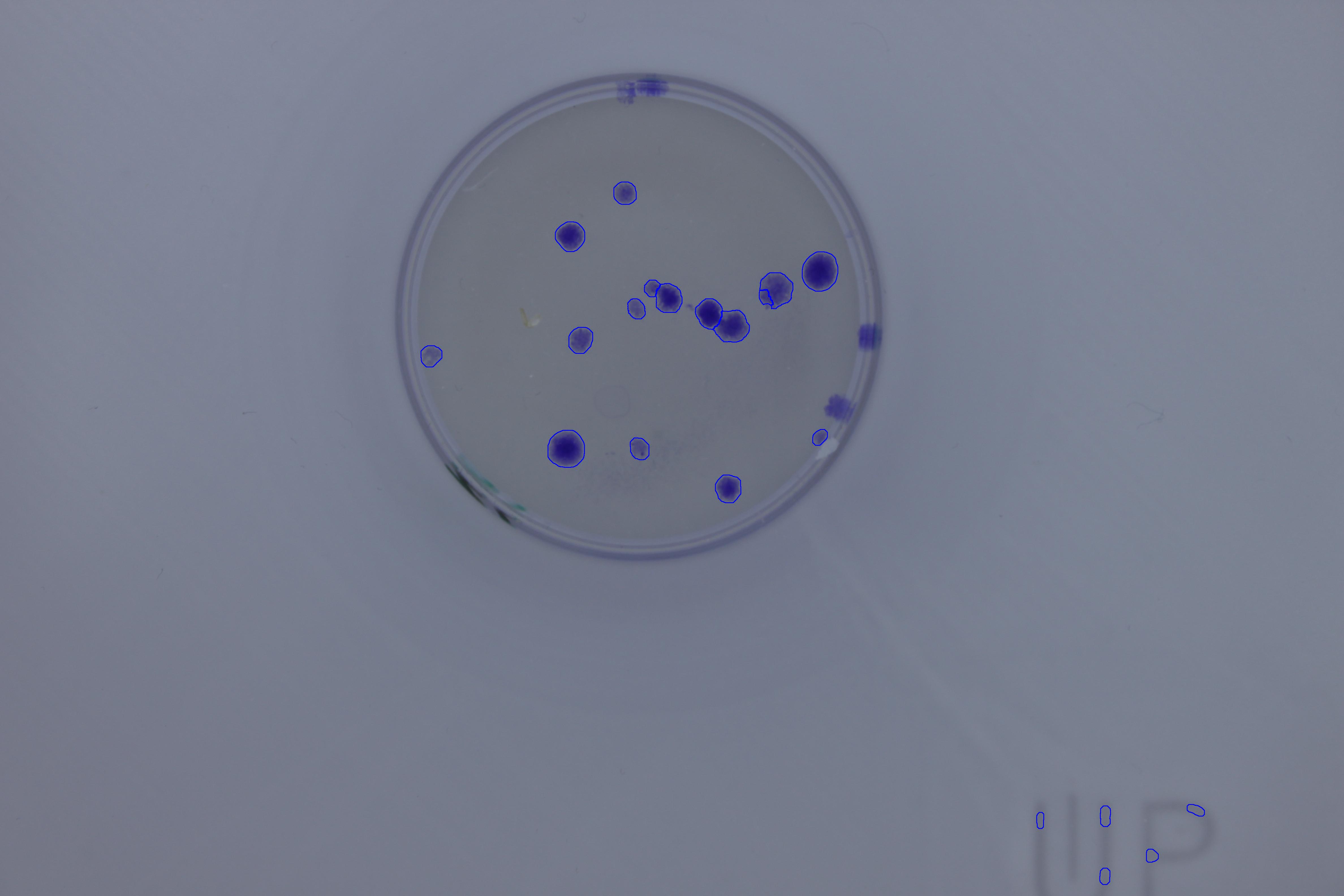

Supplement: S1 Comparison to others — (ZIP) [file pone.0205823.s007.zip › S1 Comparison to others/AutoCellSeg/180501 HeLa Dish/2_seg.jpg]

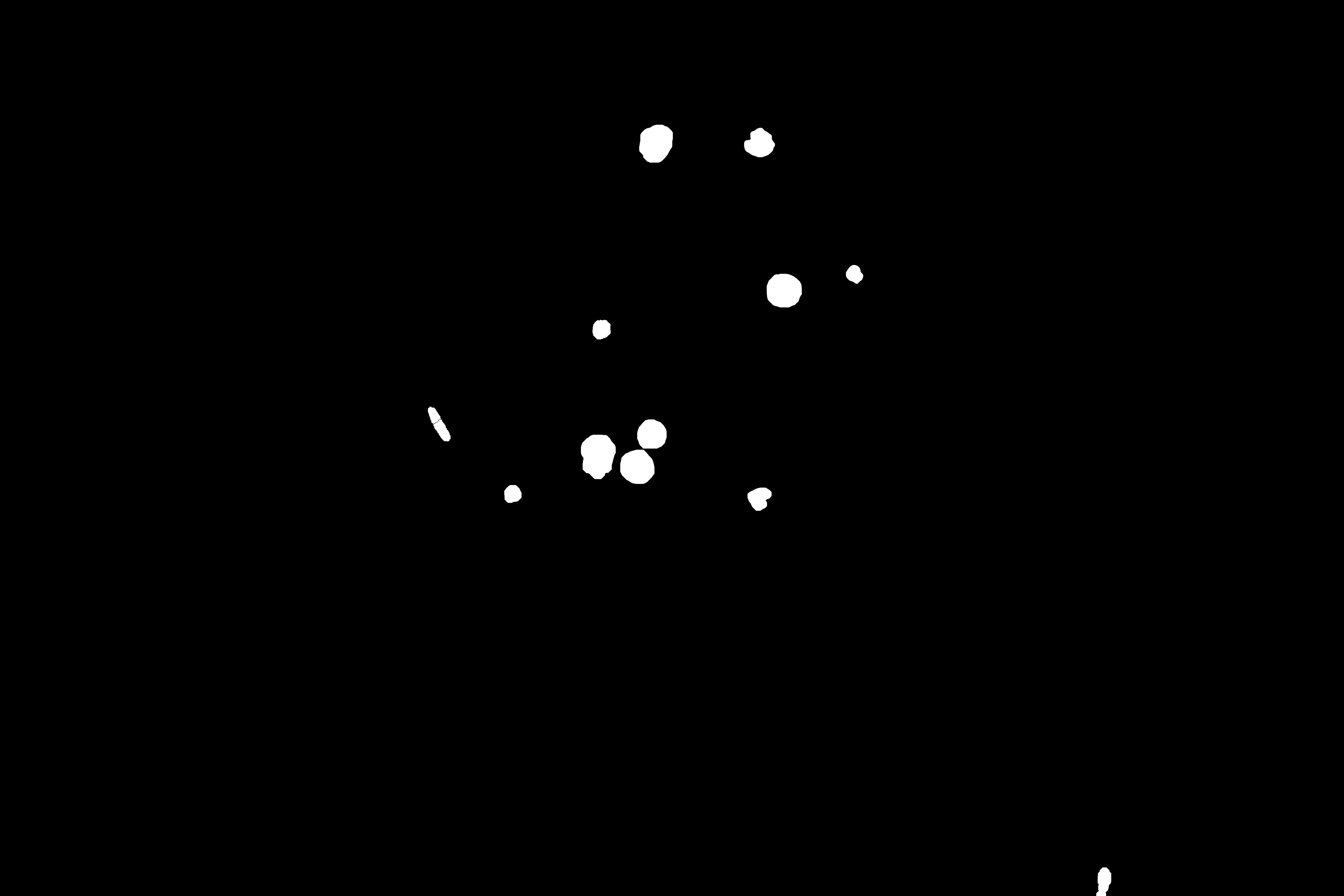

Supplement: S1 Comparison to others — (ZIP) [file pone.0205823.s007.zip › S1 Comparison to others/AutoCellSeg/180501 HeLa Dish/3_mask.jpg]

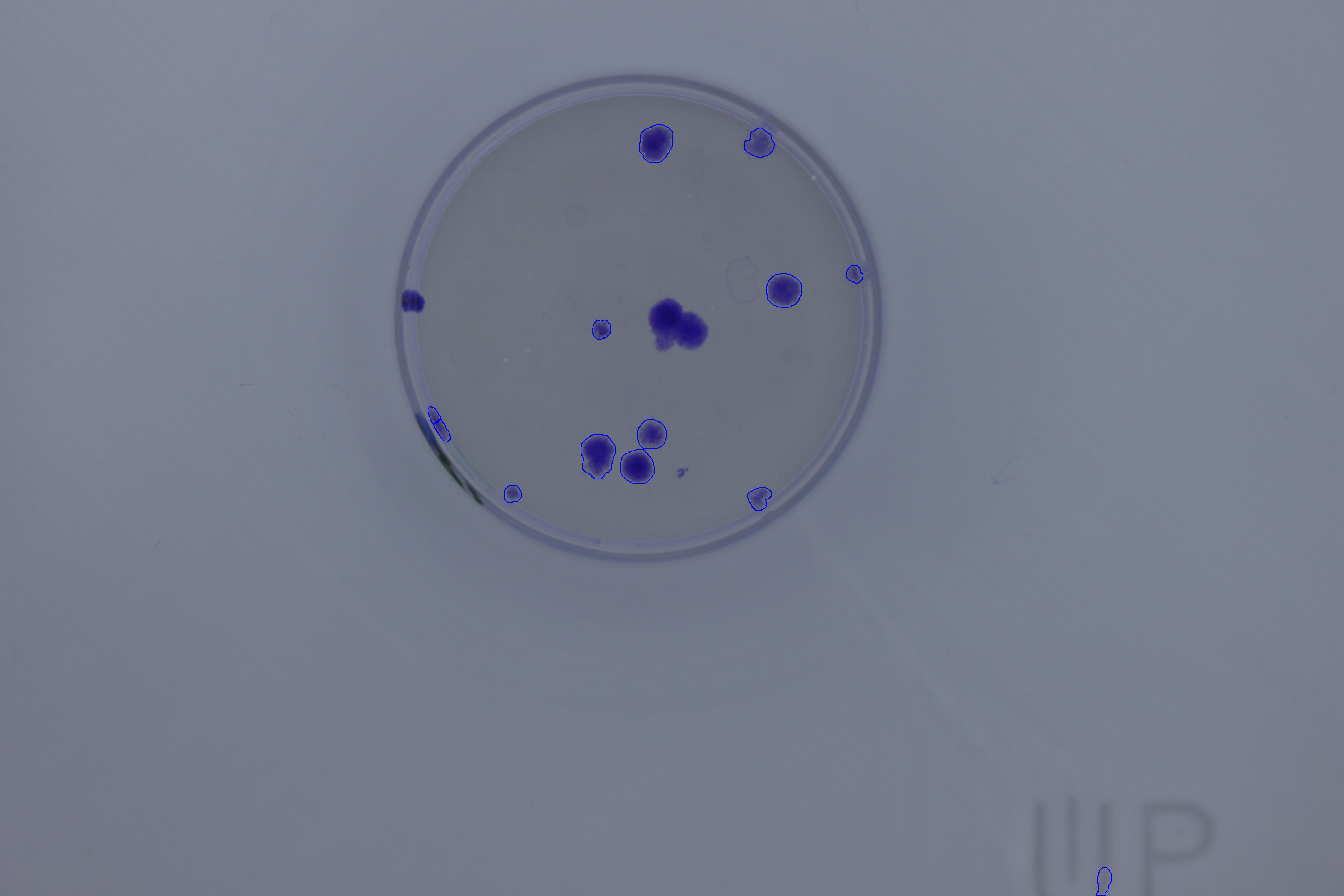

Supplement: S1 Comparison to others — (ZIP) [file pone.0205823.s007.zip › S1 Comparison to others/AutoCellSeg/180501 HeLa Dish/3_seg.jpg]

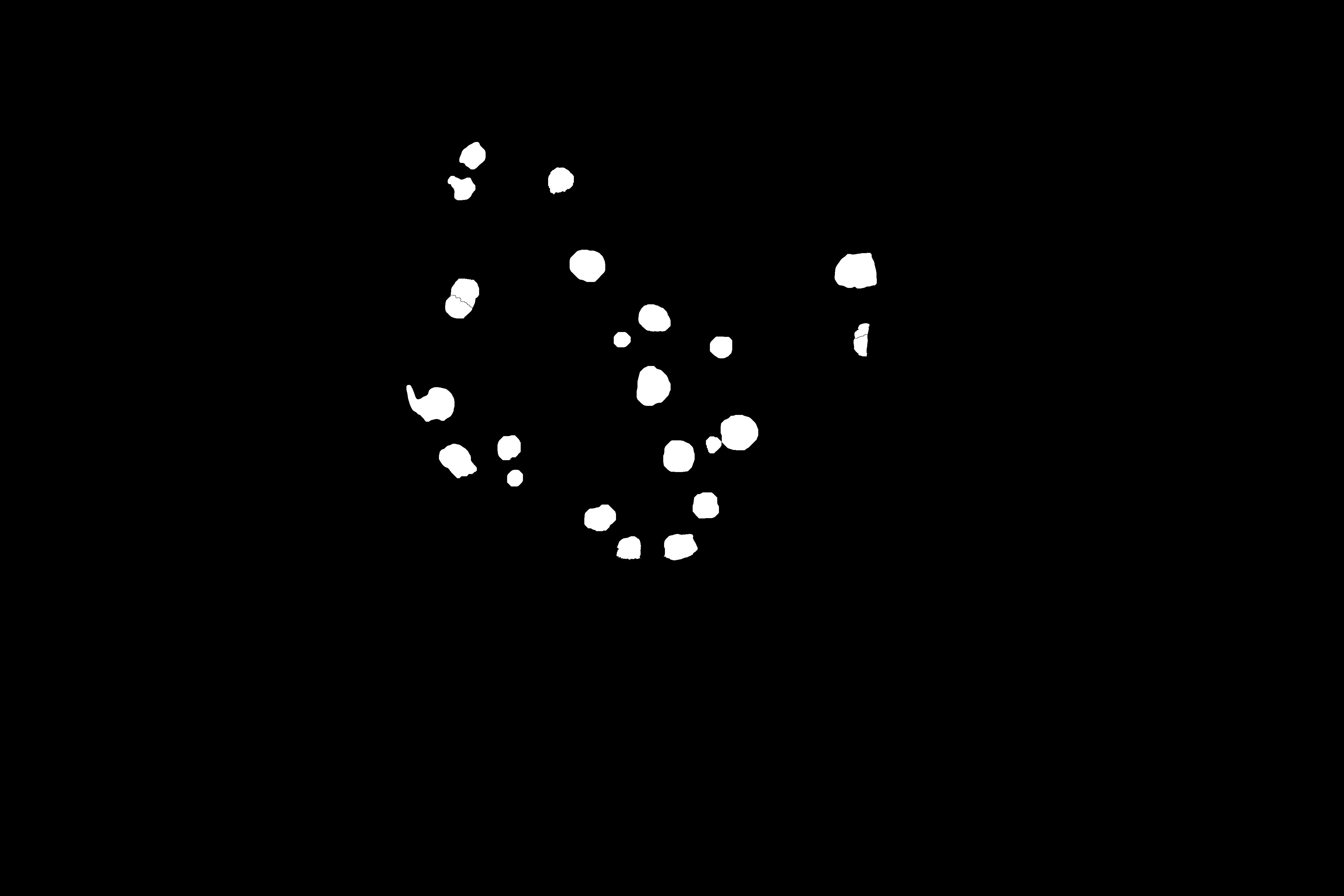

Supplement: S1 Comparison to others — (ZIP) [file pone.0205823.s007.zip › S1 Comparison to others/AutoCellSeg/180501 HeLa Dish/4_mask.jpg]

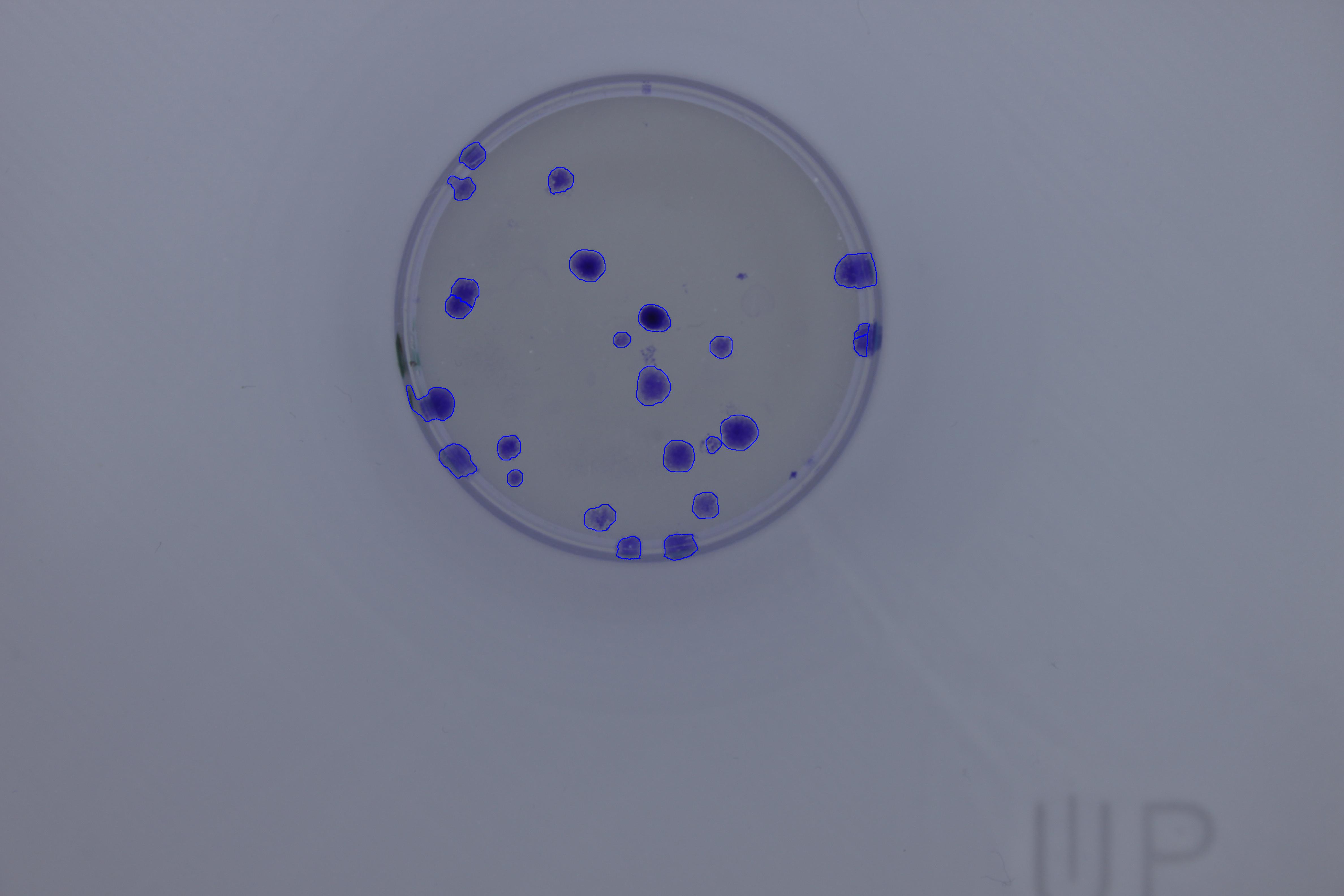

Supplement: S1 Comparison to others — (ZIP) [file pone.0205823.s007.zip › S1 Comparison to others/AutoCellSeg/180501 HeLa Dish/4_seg.jpg]

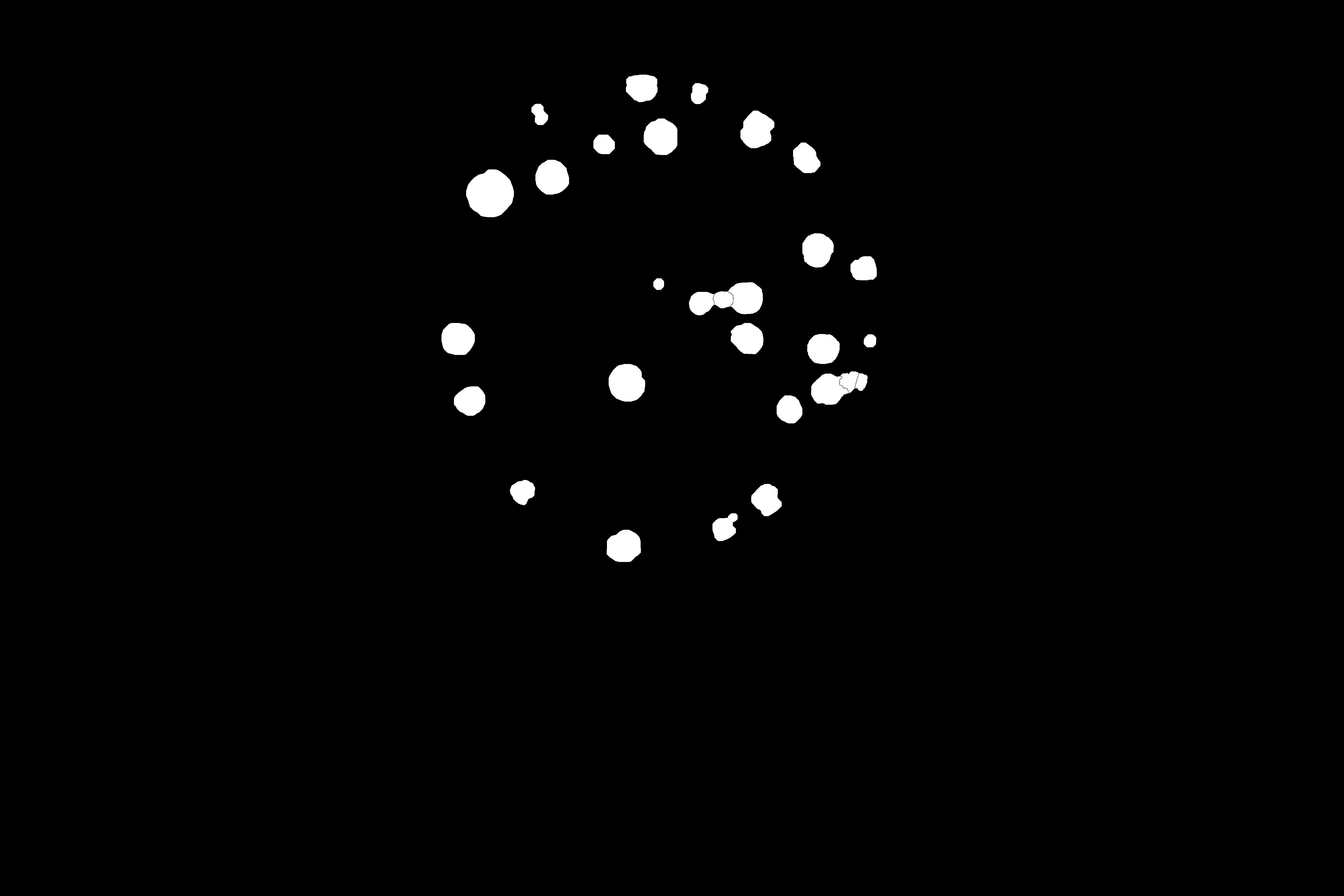

Supplement: S1 Comparison to others — (ZIP) [file pone.0205823.s007.zip › S1 Comparison to others/AutoCellSeg/180501 HeLa Dish/5_mask.jpg]

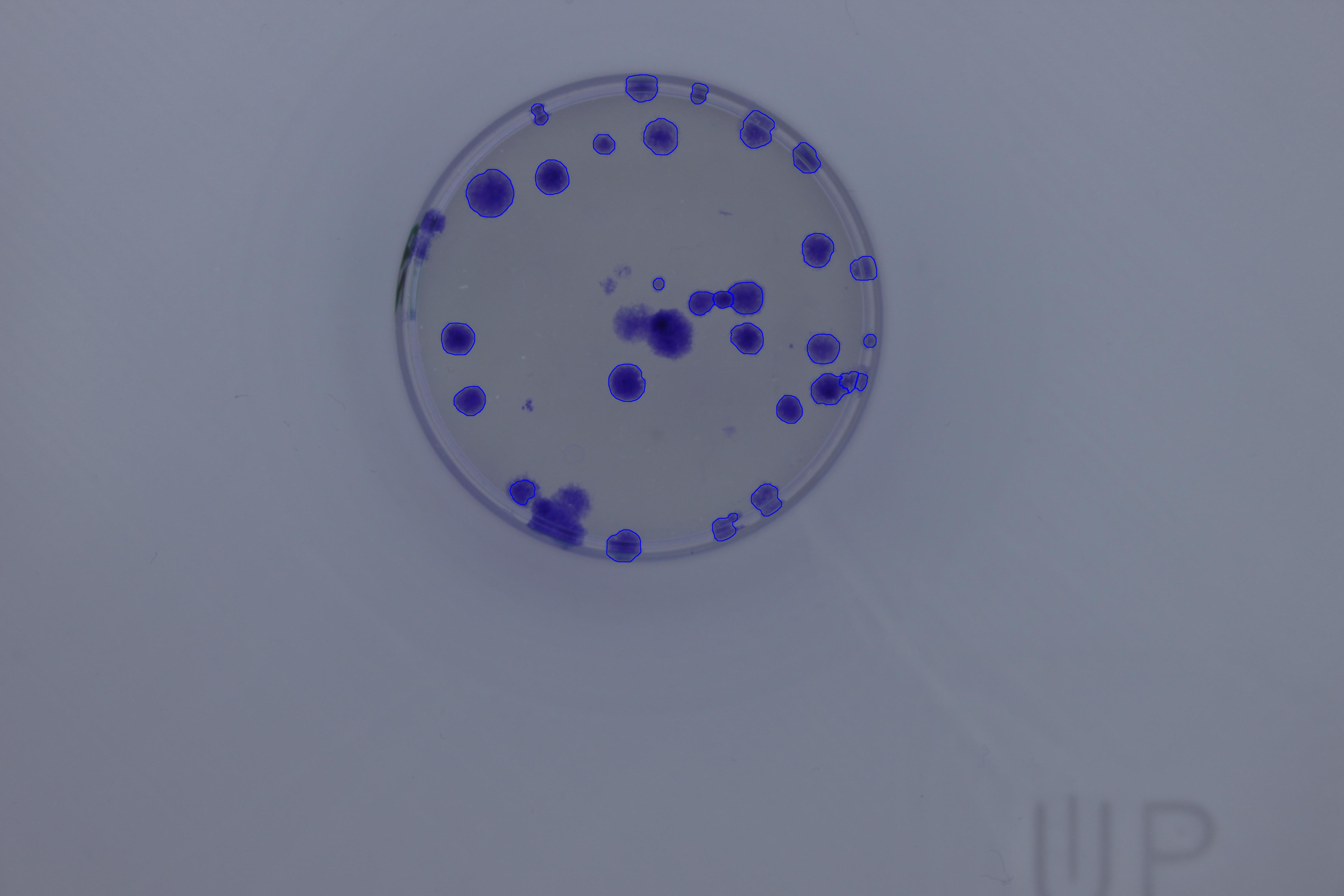

Supplement: S1 Comparison to others — (ZIP) [file pone.0205823.s007.zip › S1 Comparison to others/AutoCellSeg/180501 HeLa Dish/5_seg.jpg]

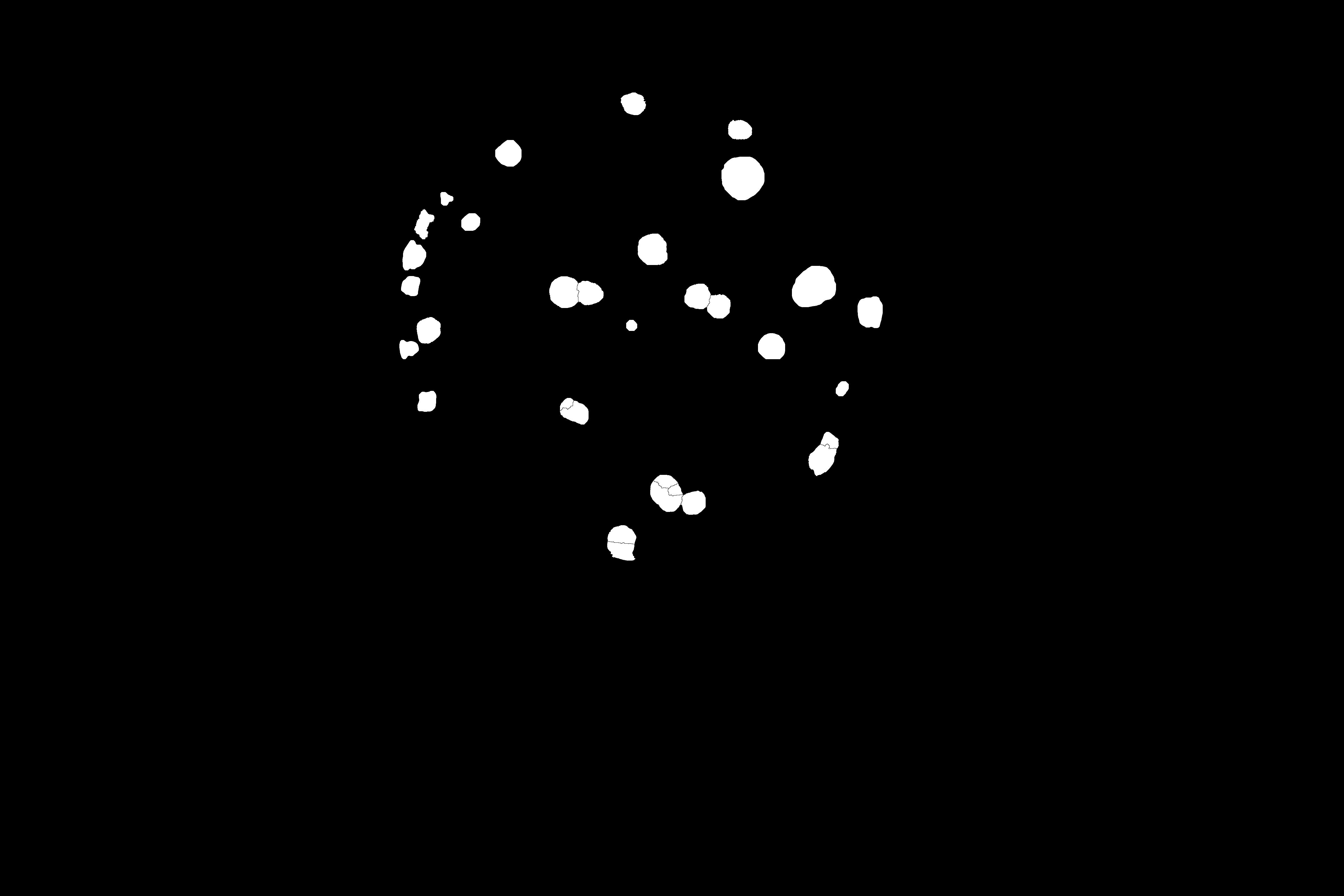

Supplement: S1 Comparison to others — (ZIP) [file pone.0205823.s007.zip › S1 Comparison to others/AutoCellSeg/180501 HeLa Dish/6_mask.jpg]

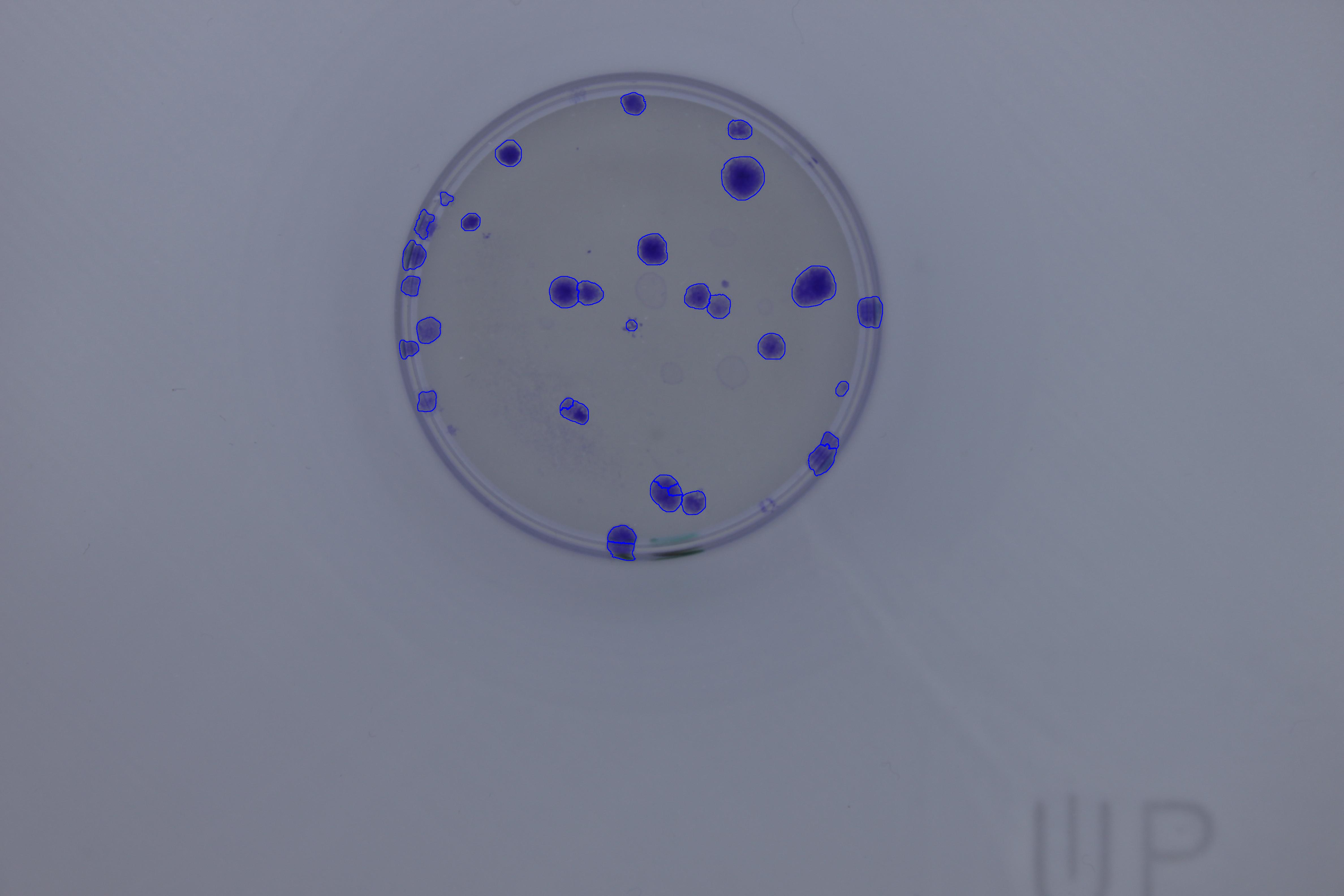

Supplement: S1 Comparison to others — (ZIP) [file pone.0205823.s007.zip › S1 Comparison to others/AutoCellSeg/180501 HeLa Dish/6_seg.jpg]

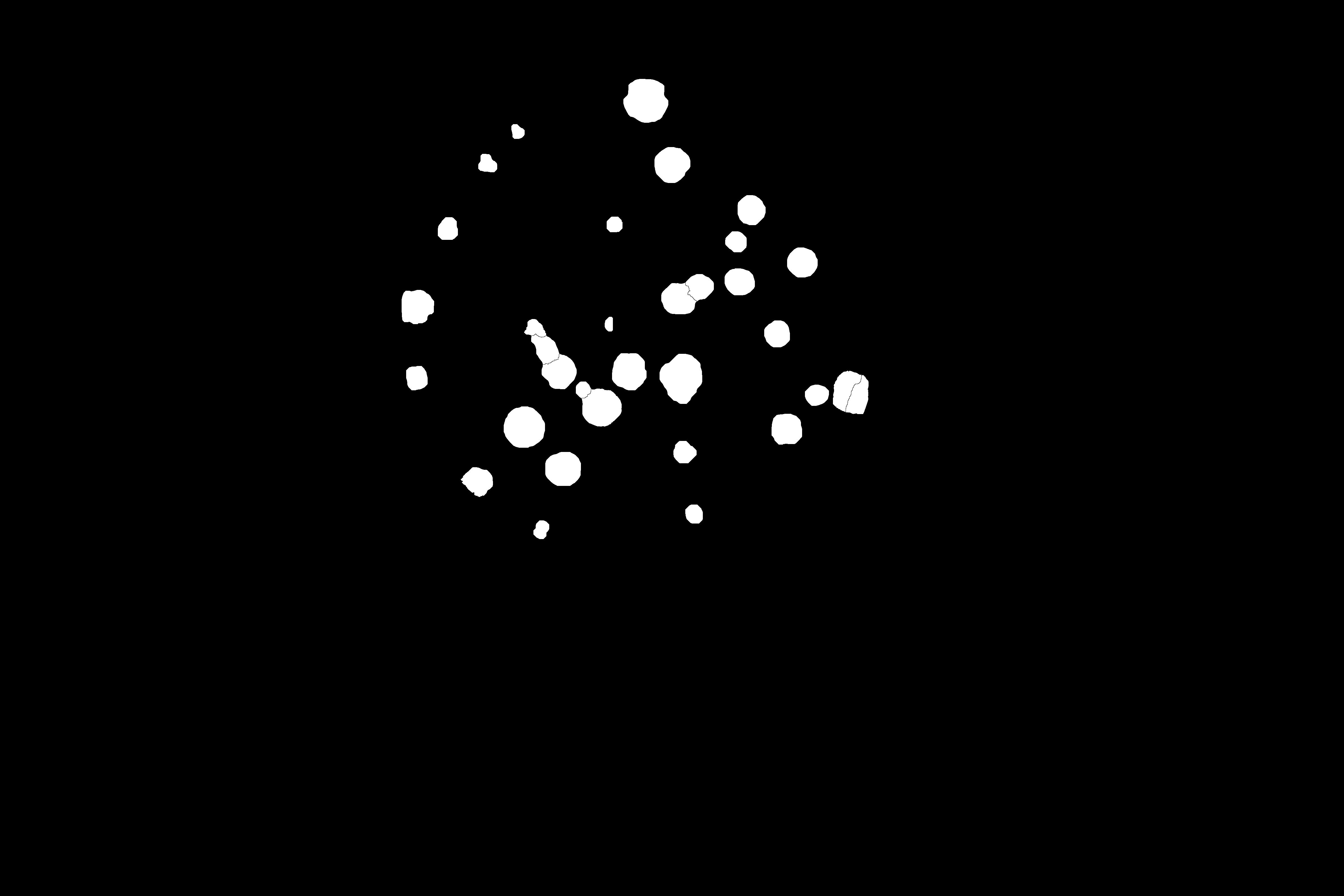

Supplement: S1 Comparison to others — (ZIP) [file pone.0205823.s007.zip › S1 Comparison to others/AutoCellSeg/180501 HeLa Dish/7_mask.jpg]

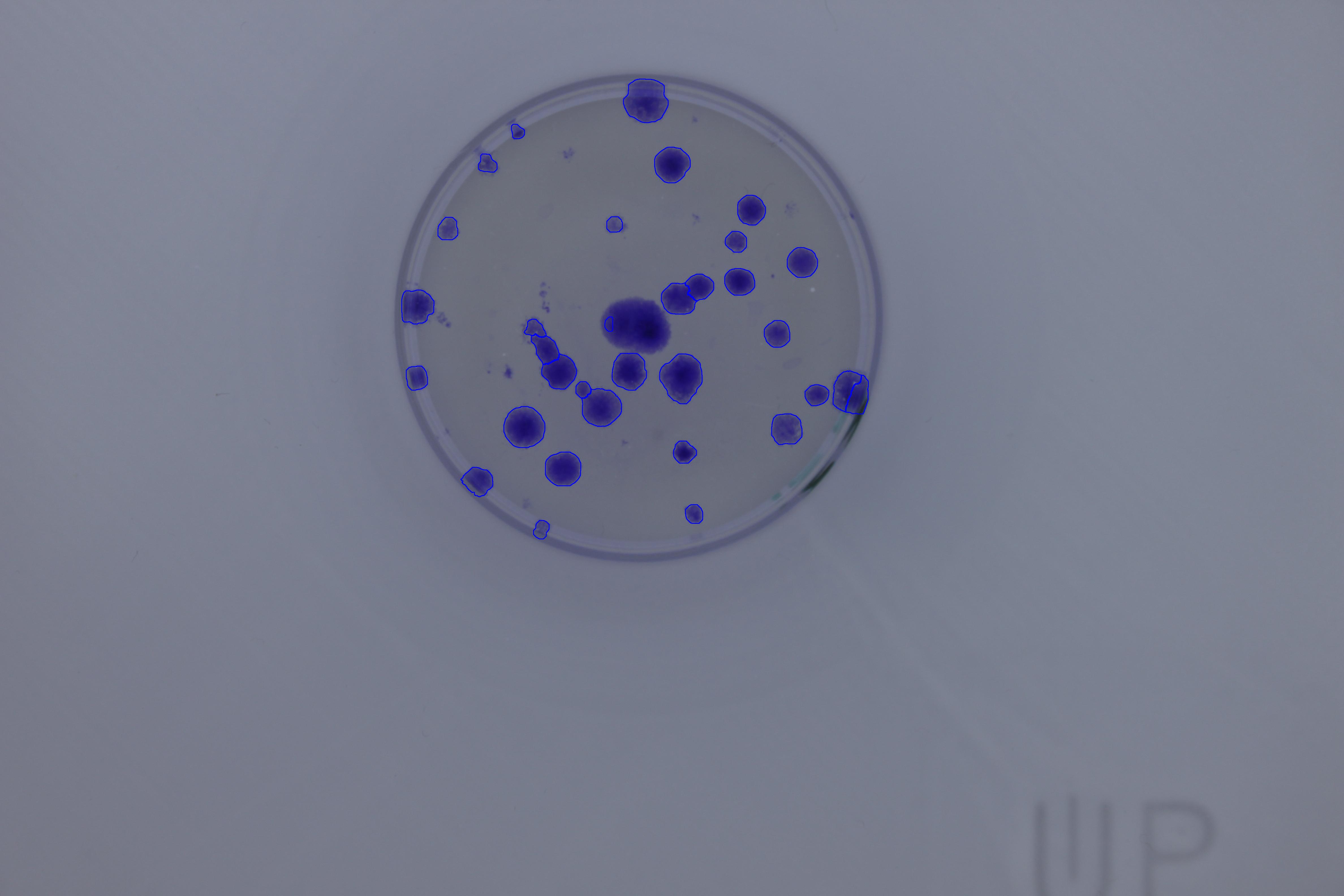

Supplement: S1 Comparison to others — (ZIP) [file pone.0205823.s007.zip › S1 Comparison to others/AutoCellSeg/180501 HeLa Dish/7_seg.jpg]

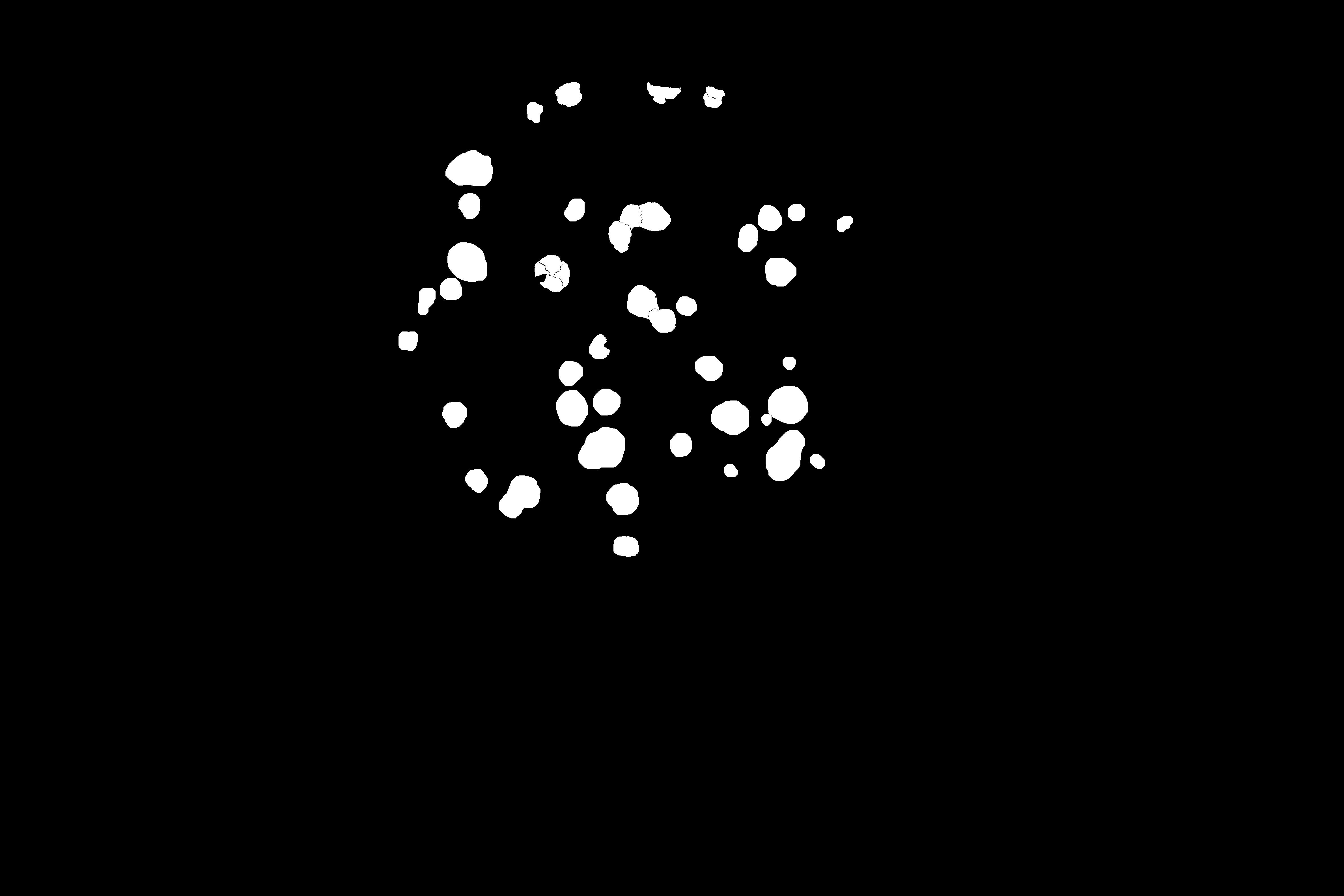

Supplement: S1 Comparison to others — (ZIP) [file pone.0205823.s007.zip › S1 Comparison to others/AutoCellSeg/180501 HeLa Dish/8_mask.jpg]

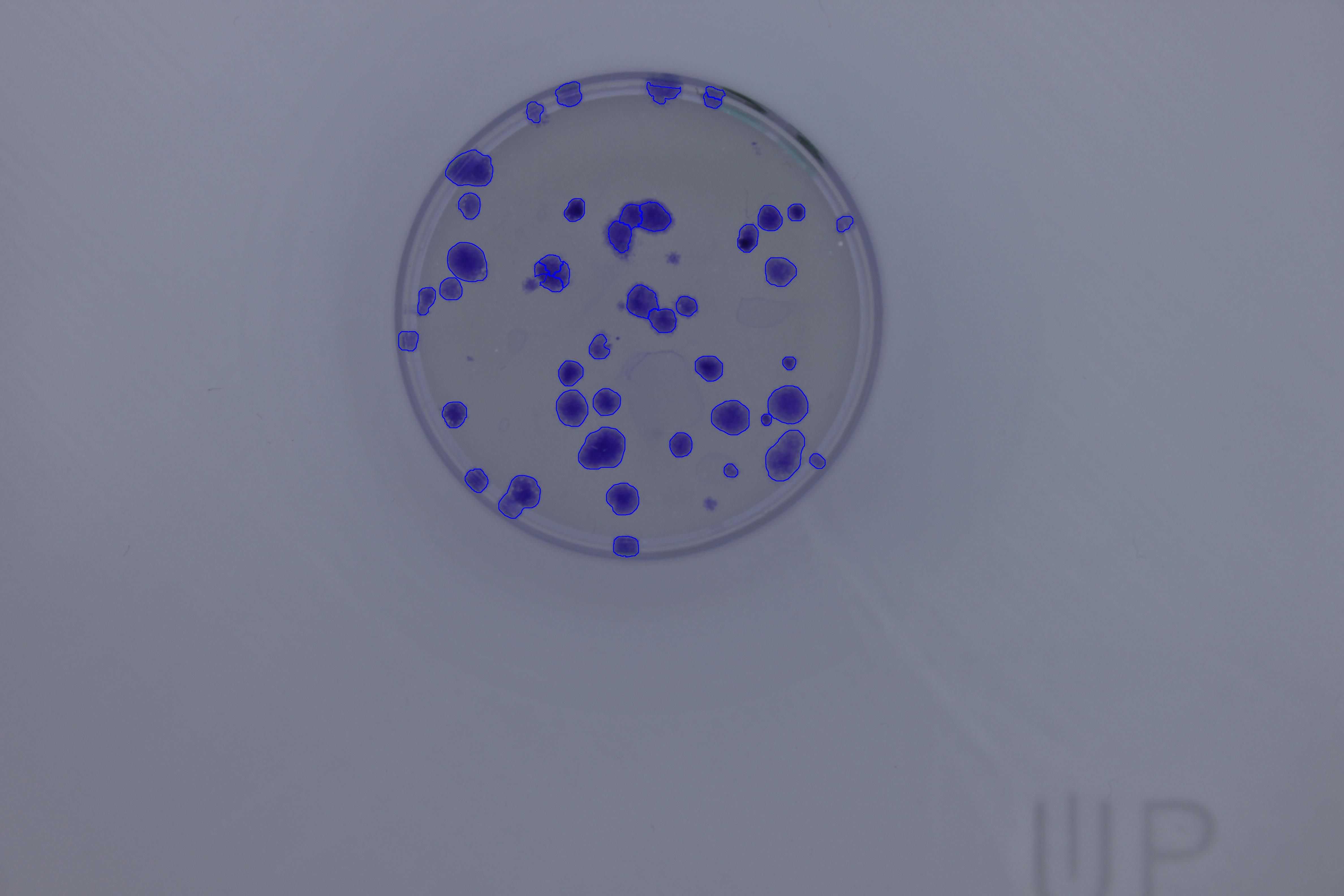

Supplement: S1 Comparison to others — (ZIP) [file pone.0205823.s007.zip › S1 Comparison to others/AutoCellSeg/180501 HeLa Dish/8_seg.jpg]

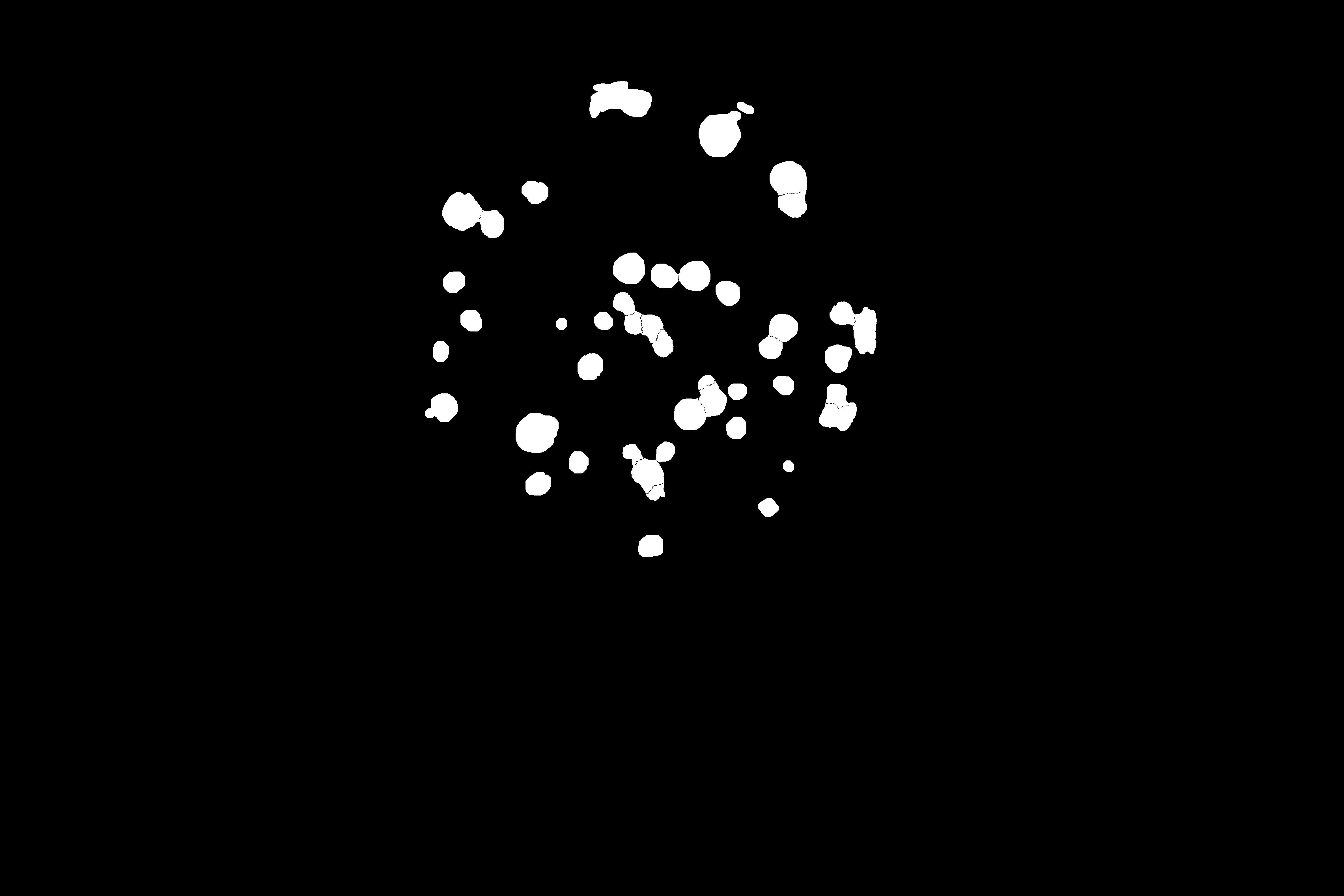

Supplement: S1 Comparison to others — (ZIP) [file pone.0205823.s007.zip › S1 Comparison to others/AutoCellSeg/180501 HeLa Dish/9_mask.jpg]

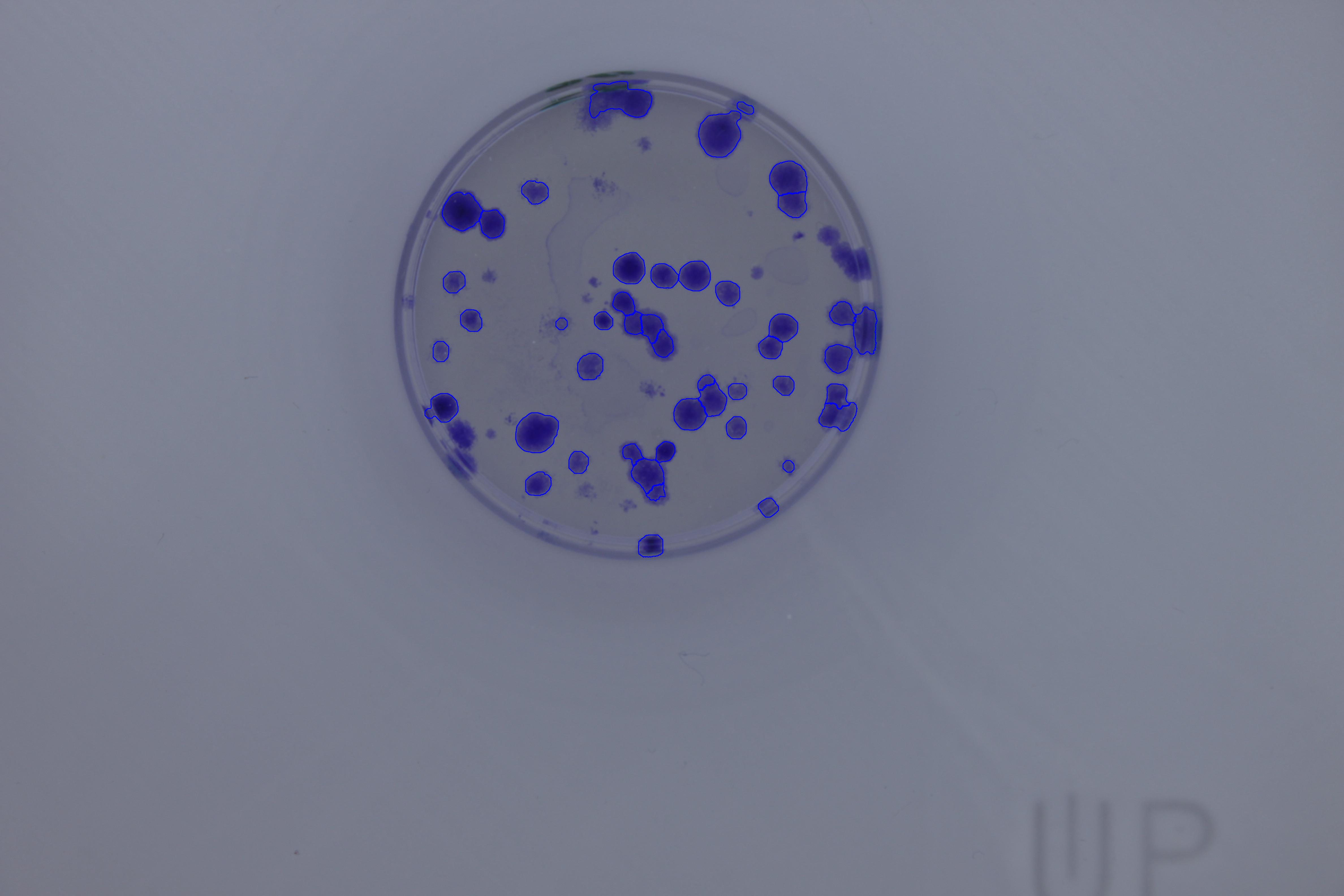

Supplement: S1 Comparison to others — (ZIP) [file pone.0205823.s007.zip › S1 Comparison to others/AutoCellSeg/180501 HeLa Dish/9_seg.jpg]

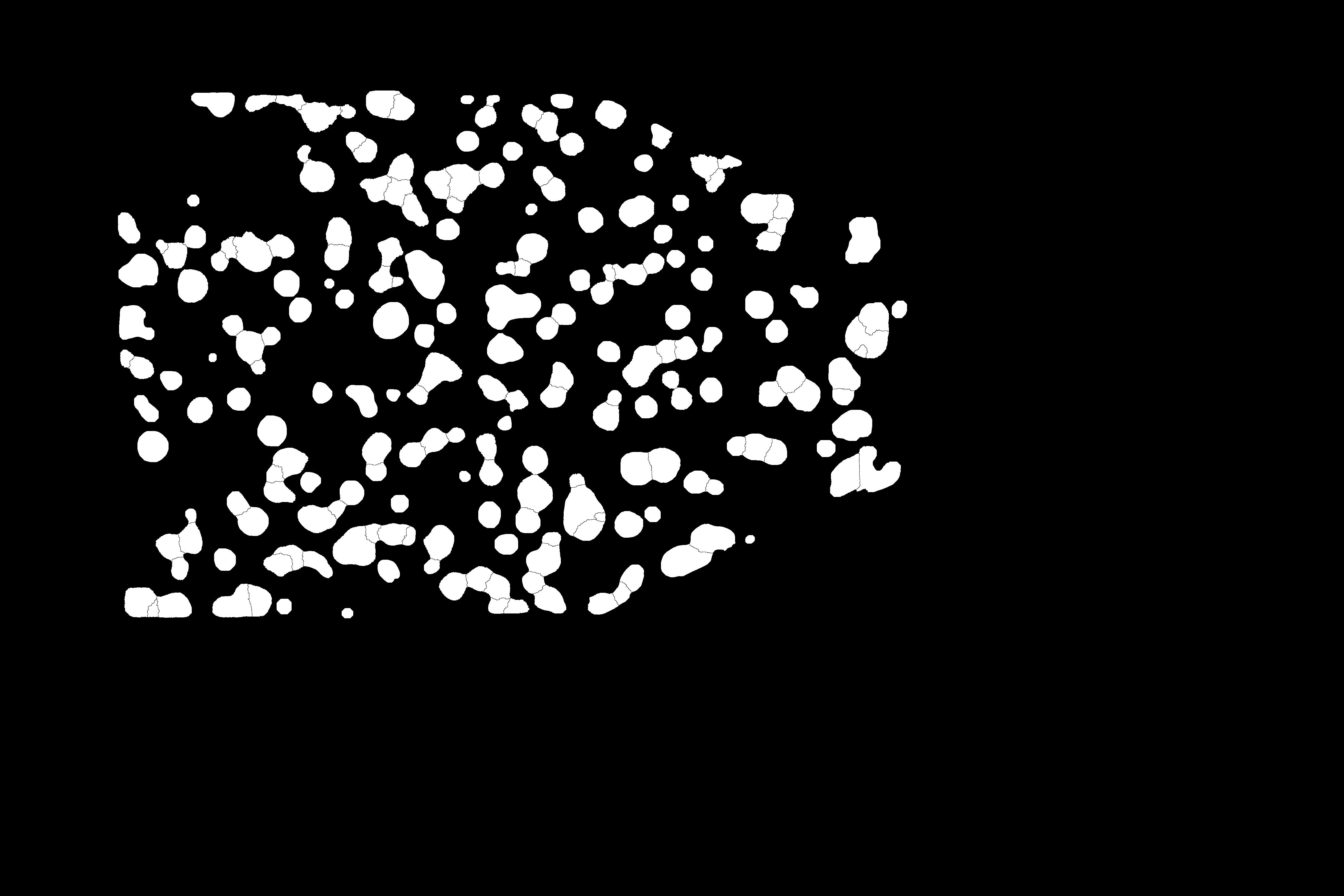

Supplement: S1 Comparison to others — (ZIP) [file pone.0205823.s007.zip › S1 Comparison to others/AutoCellSeg/180501 HeLa Flask/10_mask.jpg]

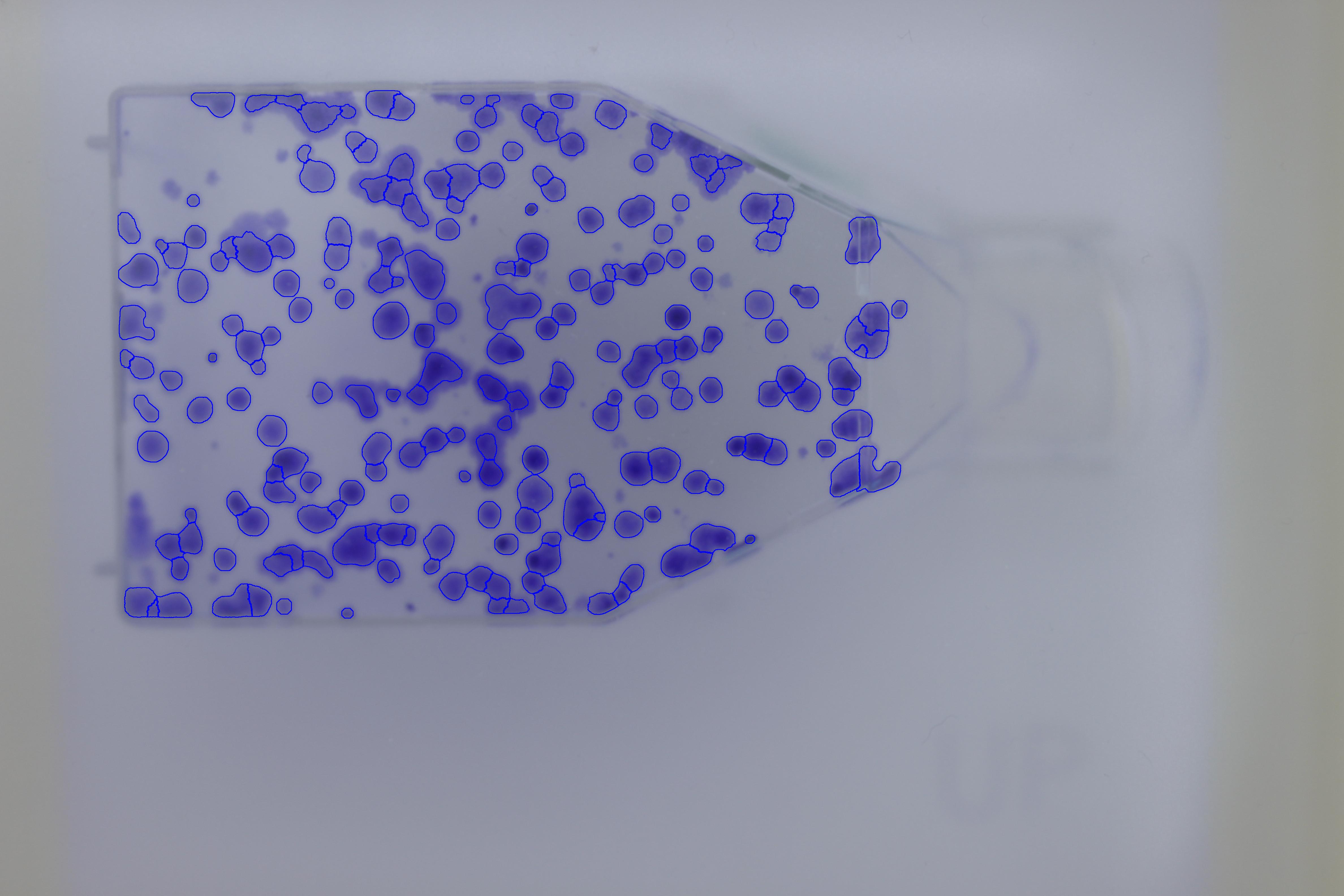

Supplement: S1 Comparison to others — (ZIP) [file pone.0205823.s007.zip › S1 Comparison to others/AutoCellSeg/180501 HeLa Flask/10_seg.jpg]

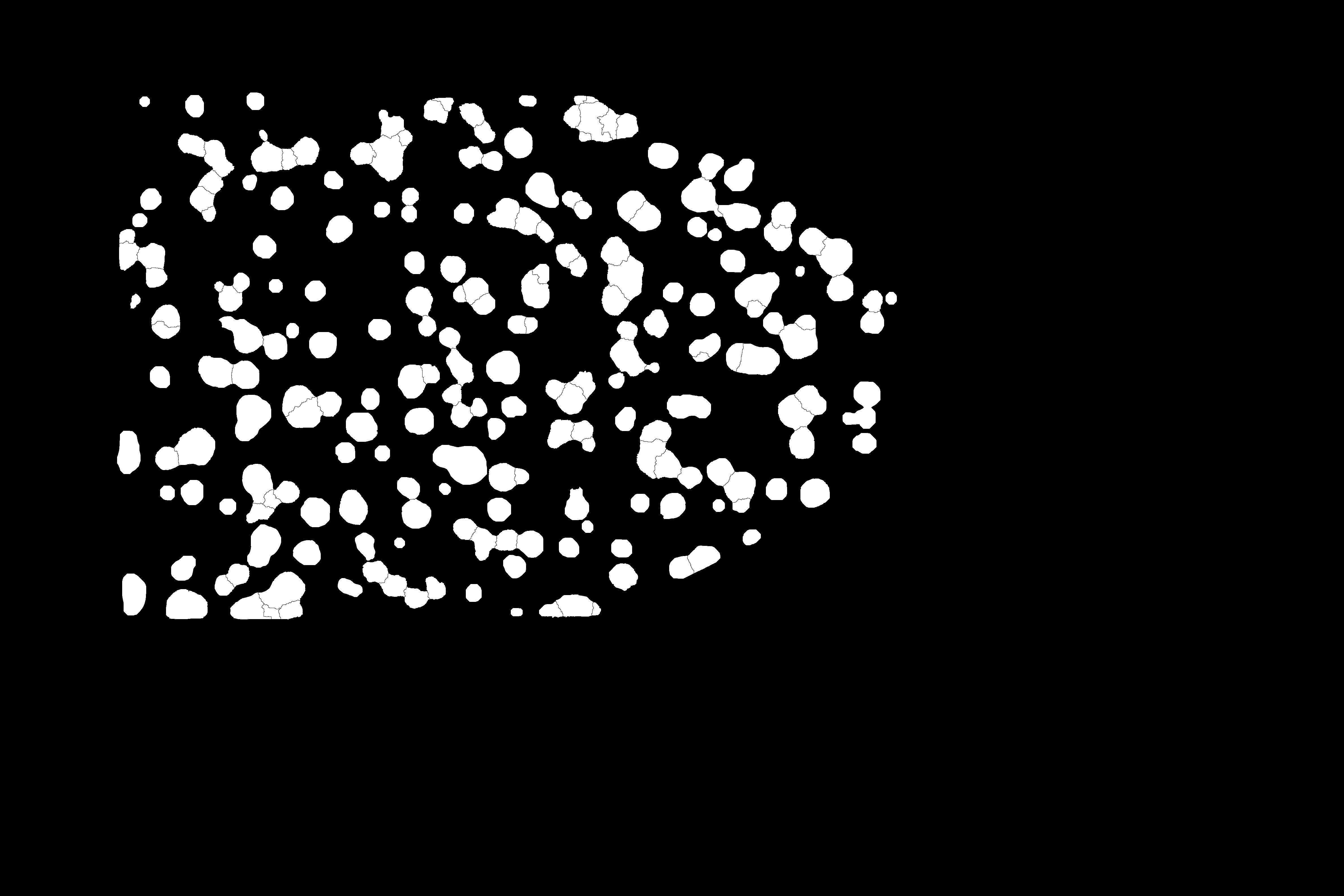

Supplement: S1 Comparison to others — (ZIP) [file pone.0205823.s007.zip › S1 Comparison to others/AutoCellSeg/180501 HeLa Flask/11_mask.jpg]

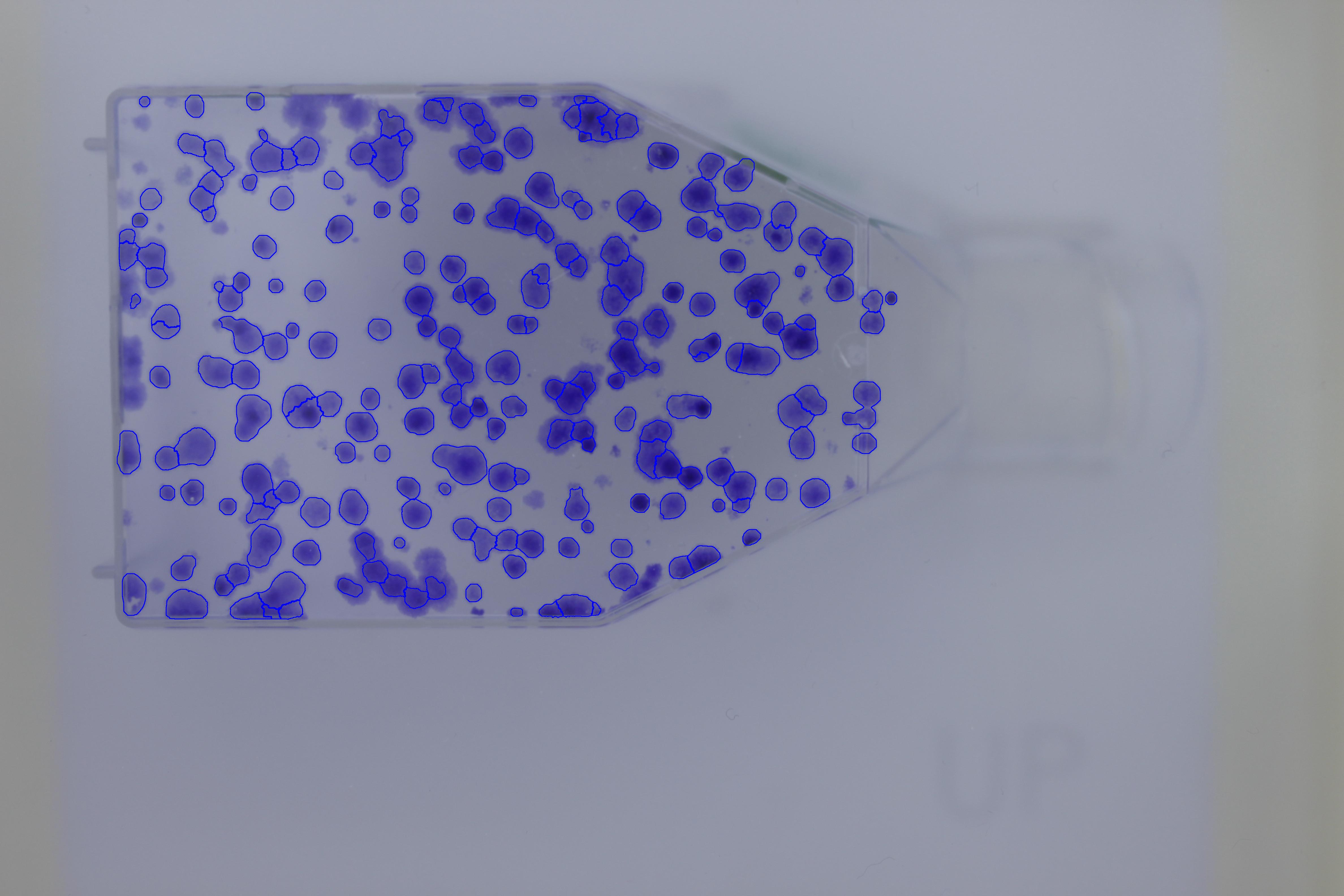

Supplement: S1 Comparison to others — (ZIP) [file pone.0205823.s007.zip › S1 Comparison to others/AutoCellSeg/180501 HeLa Flask/11_seg.jpg]

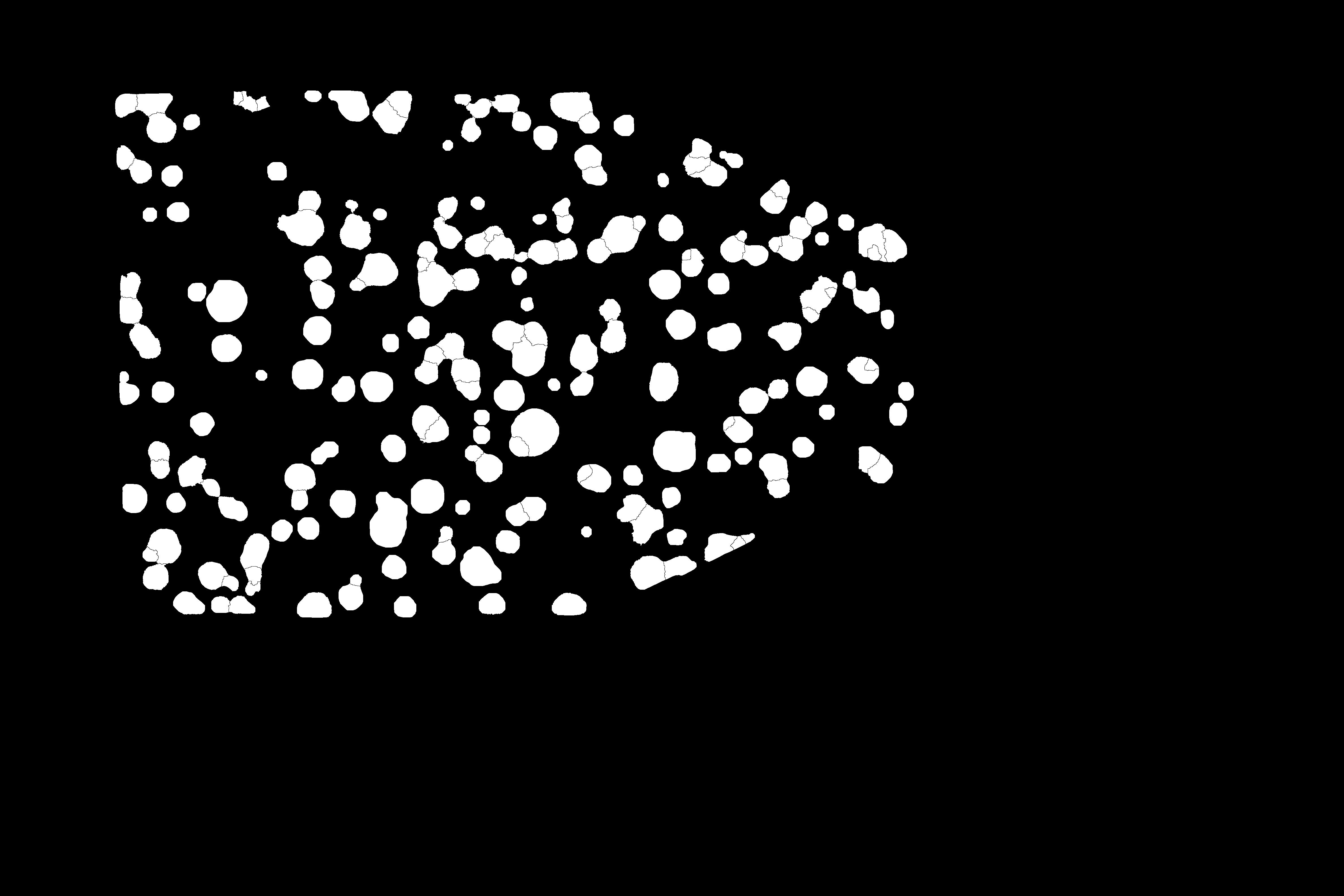

Supplement: S1 Comparison to others — (ZIP) [file pone.0205823.s007.zip › S1 Comparison to others/AutoCellSeg/180501 HeLa Flask/12_mask.jpg]

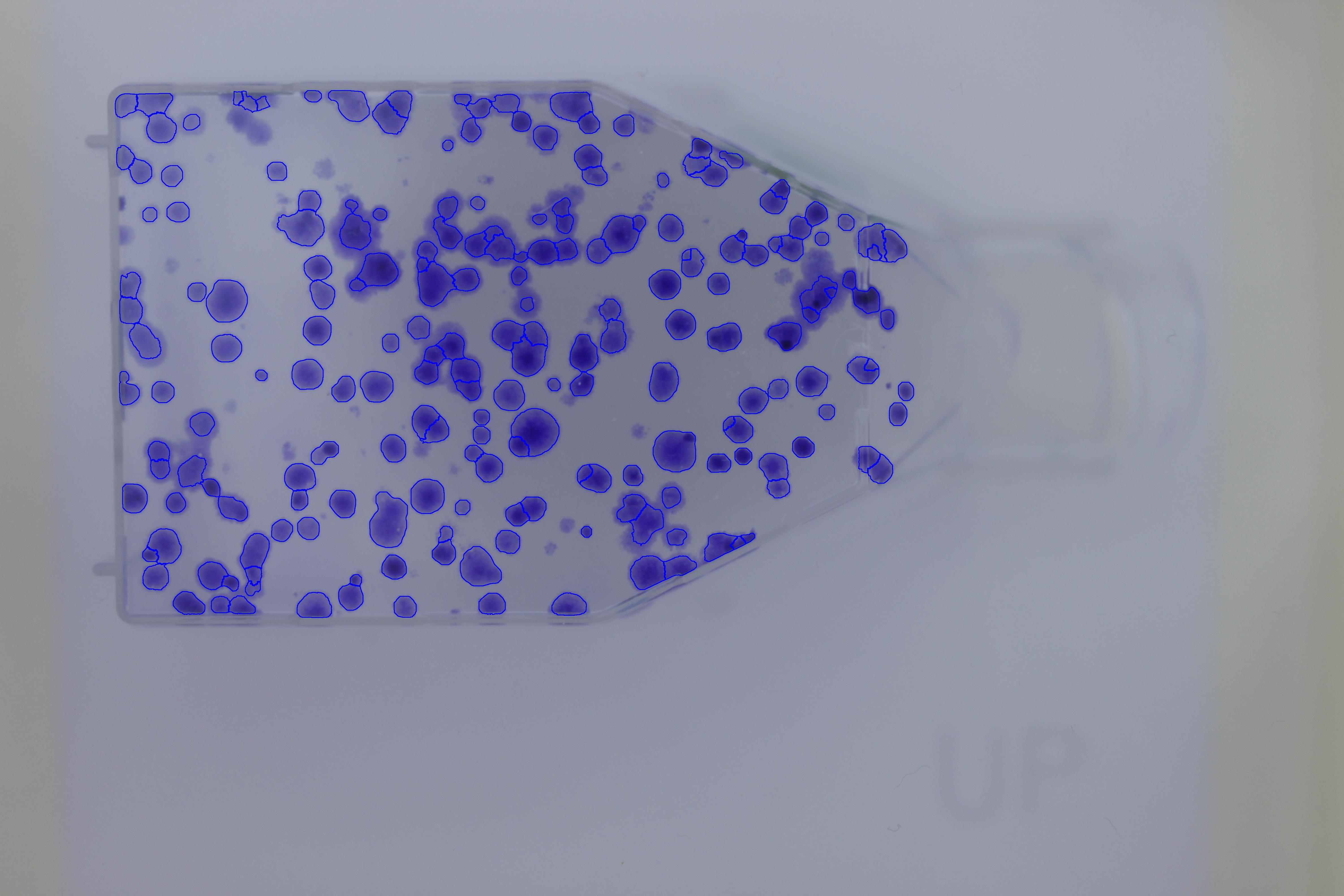

Supplement: S1 Comparison to others — (ZIP) [file pone.0205823.s007.zip › S1 Comparison to others/AutoCellSeg/180501 HeLa Flask/12_seg.jpg]

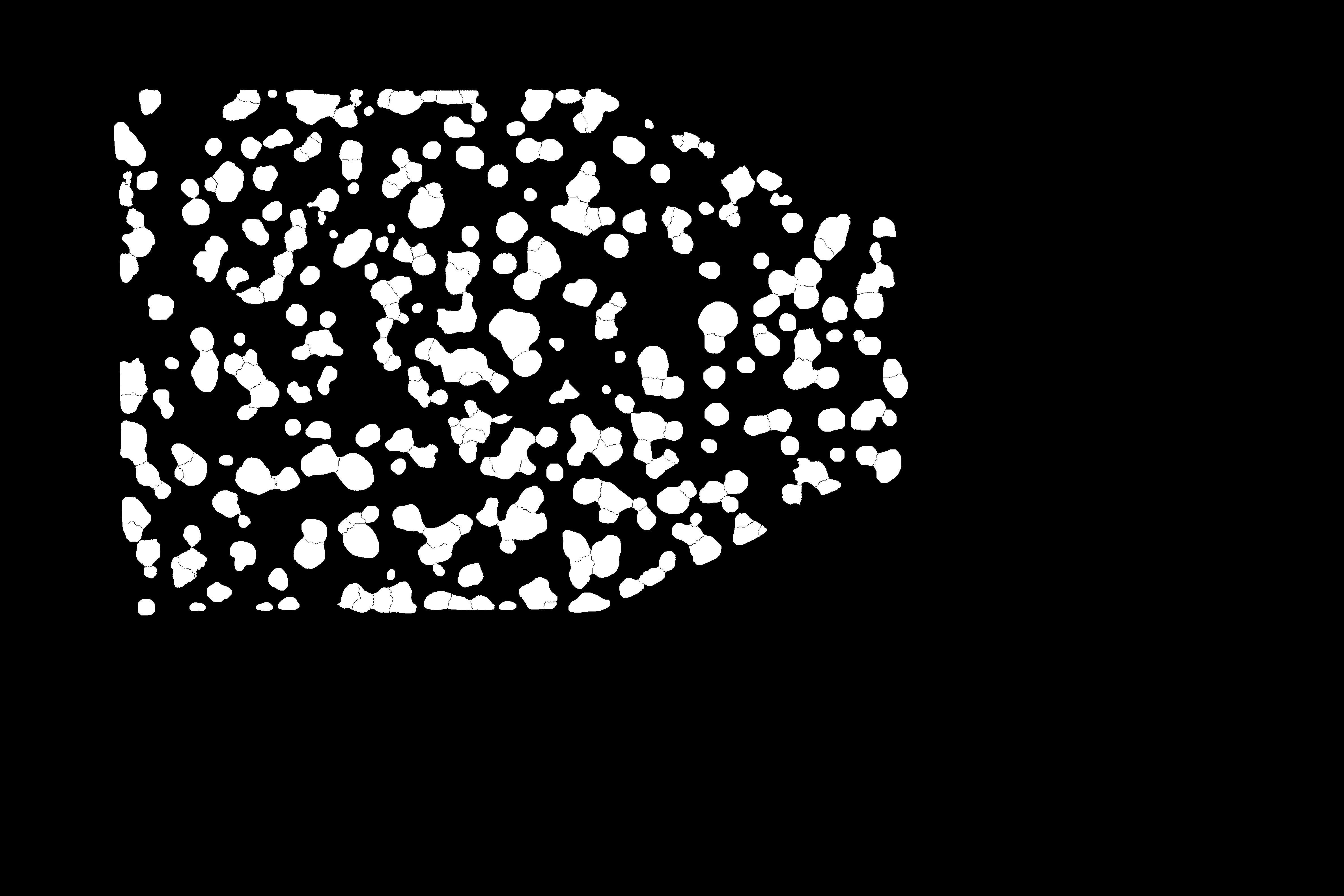

Supplement: S1 Comparison to others — (ZIP) [file pone.0205823.s007.zip › S1 Comparison to others/AutoCellSeg/180501 HeLa Flask/13_mask.jpg]

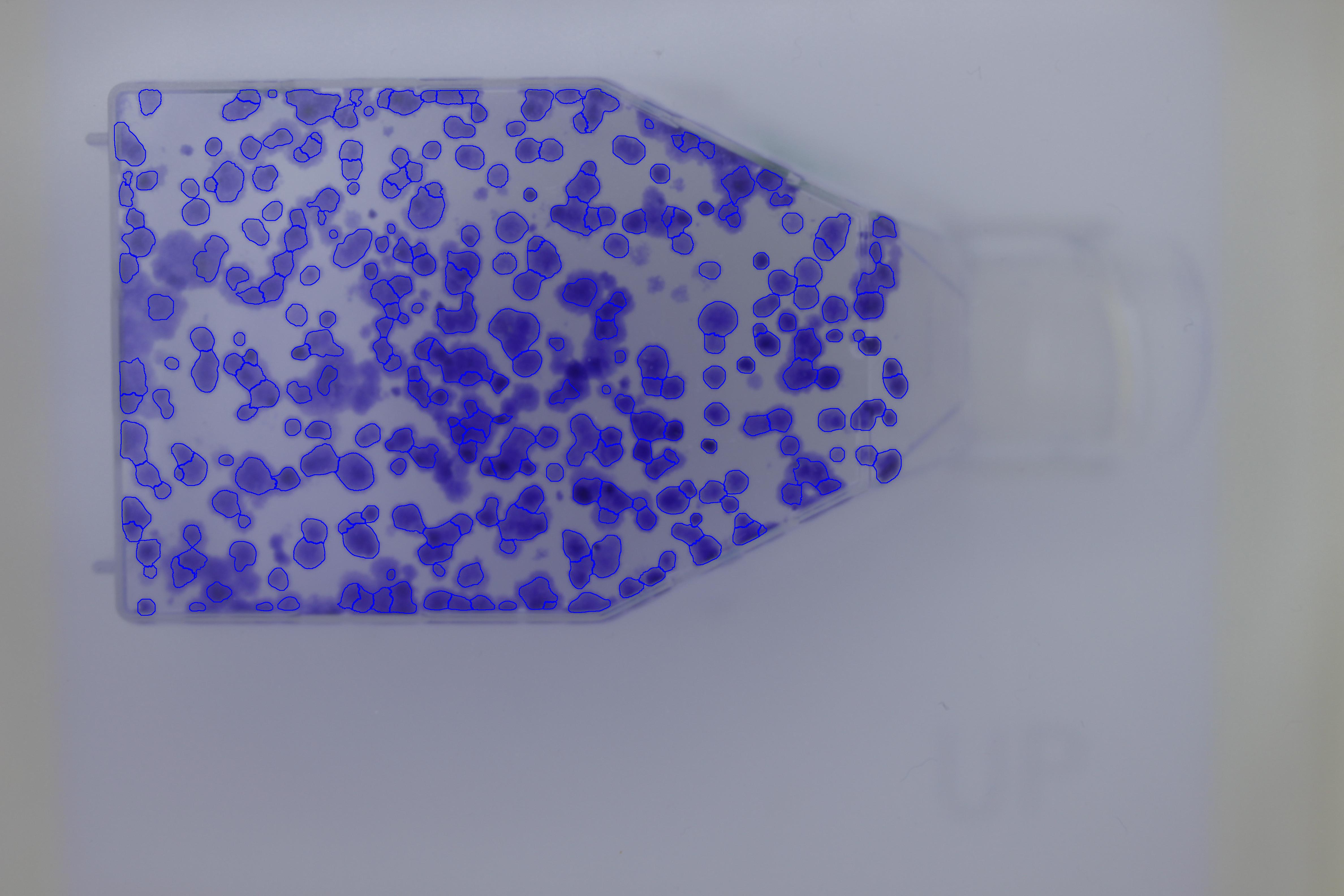

Supplement: S1 Comparison to others — (ZIP) [file pone.0205823.s007.zip › S1 Comparison to others/AutoCellSeg/180501 HeLa Flask/13_seg.jpg]

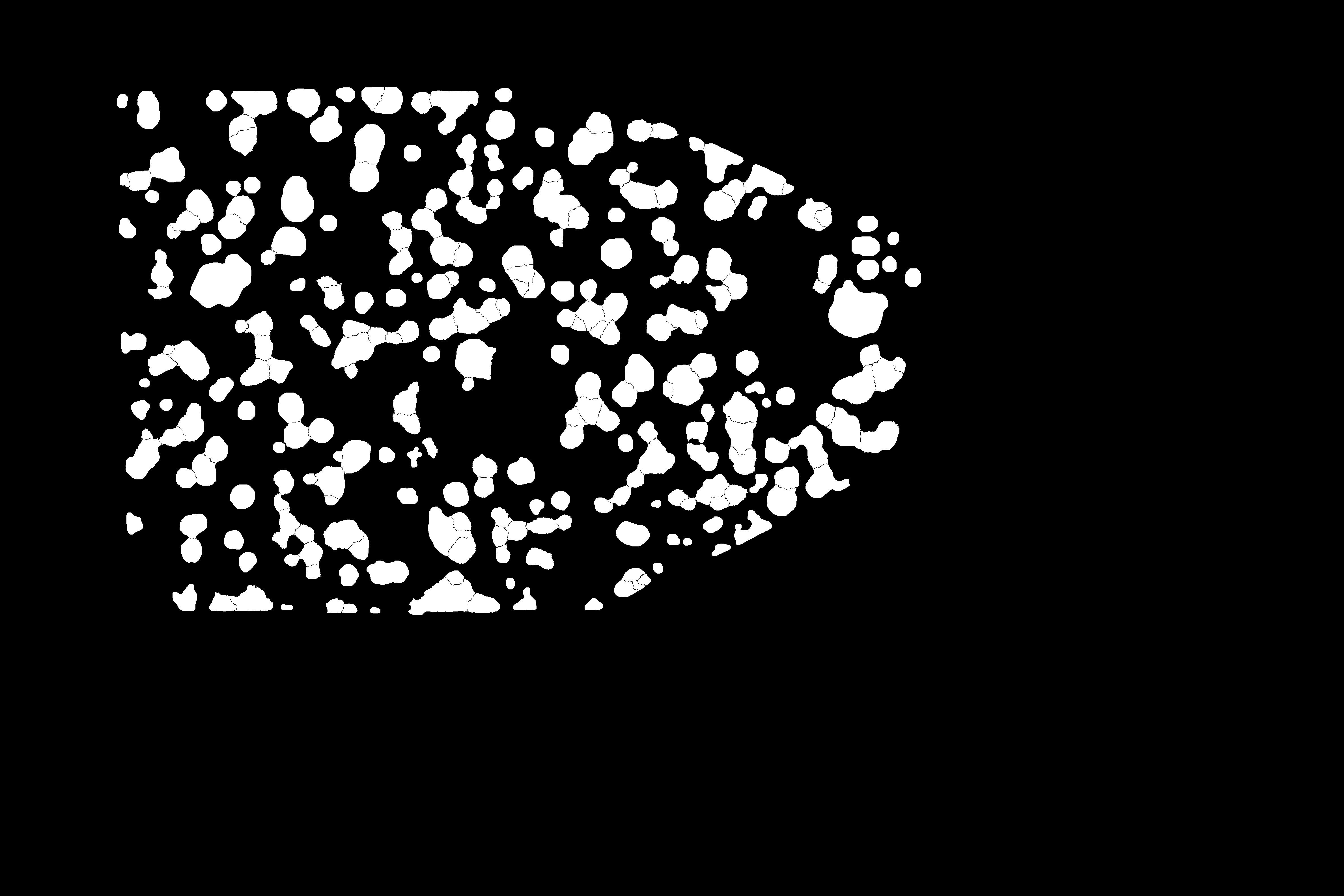

Supplement: S1 Comparison to others — (ZIP) [file pone.0205823.s007.zip › S1 Comparison to others/AutoCellSeg/180501 HeLa Flask/14_mask.jpg]

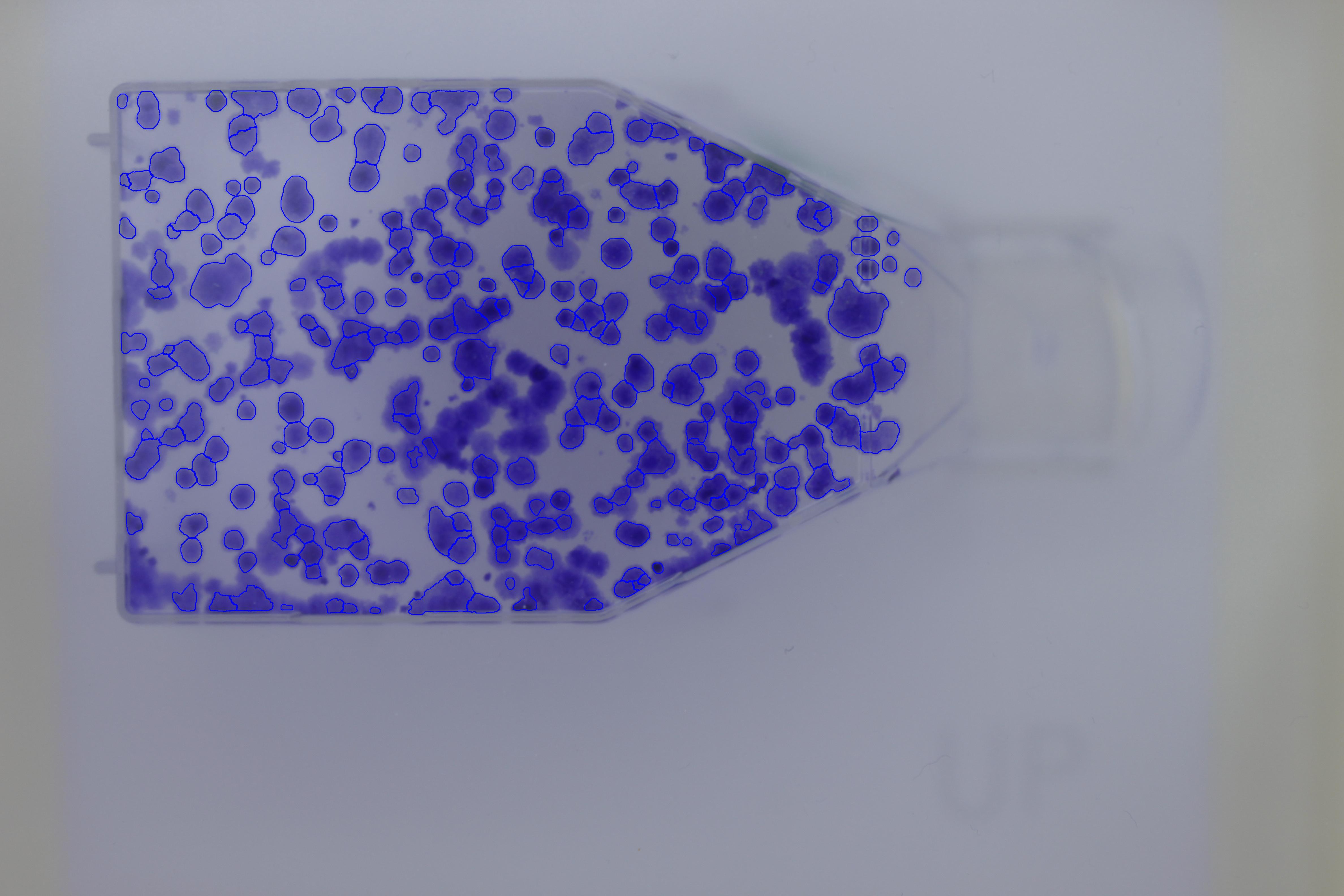

Supplement: S1 Comparison to others — (ZIP) [file pone.0205823.s007.zip › S1 Comparison to others/AutoCellSeg/180501 HeLa Flask/14_seg.jpg]

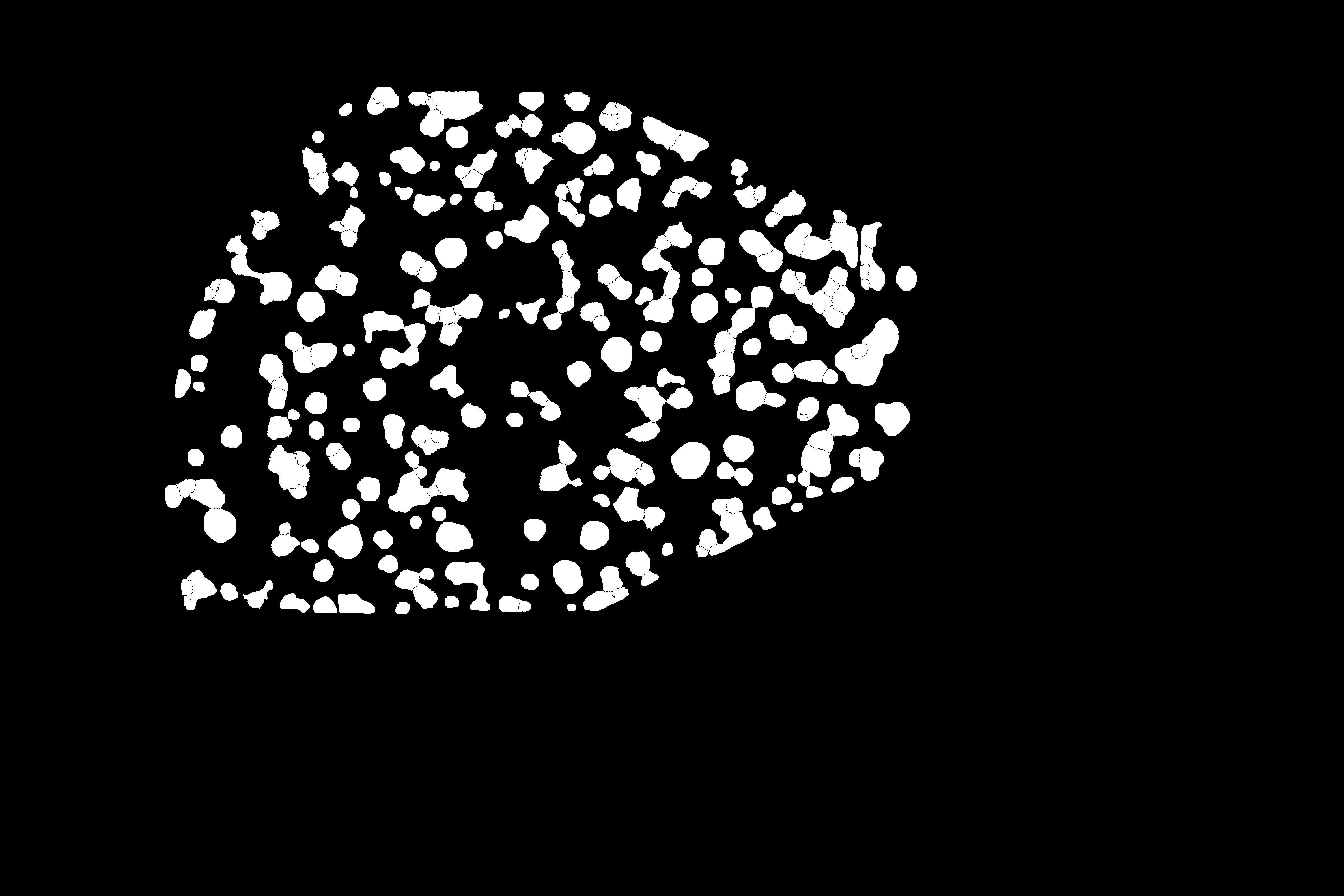

Supplement: S1 Comparison to others — (ZIP) [file pone.0205823.s007.zip › S1 Comparison to others/AutoCellSeg/180501 HeLa Flask/15_mask.jpg]

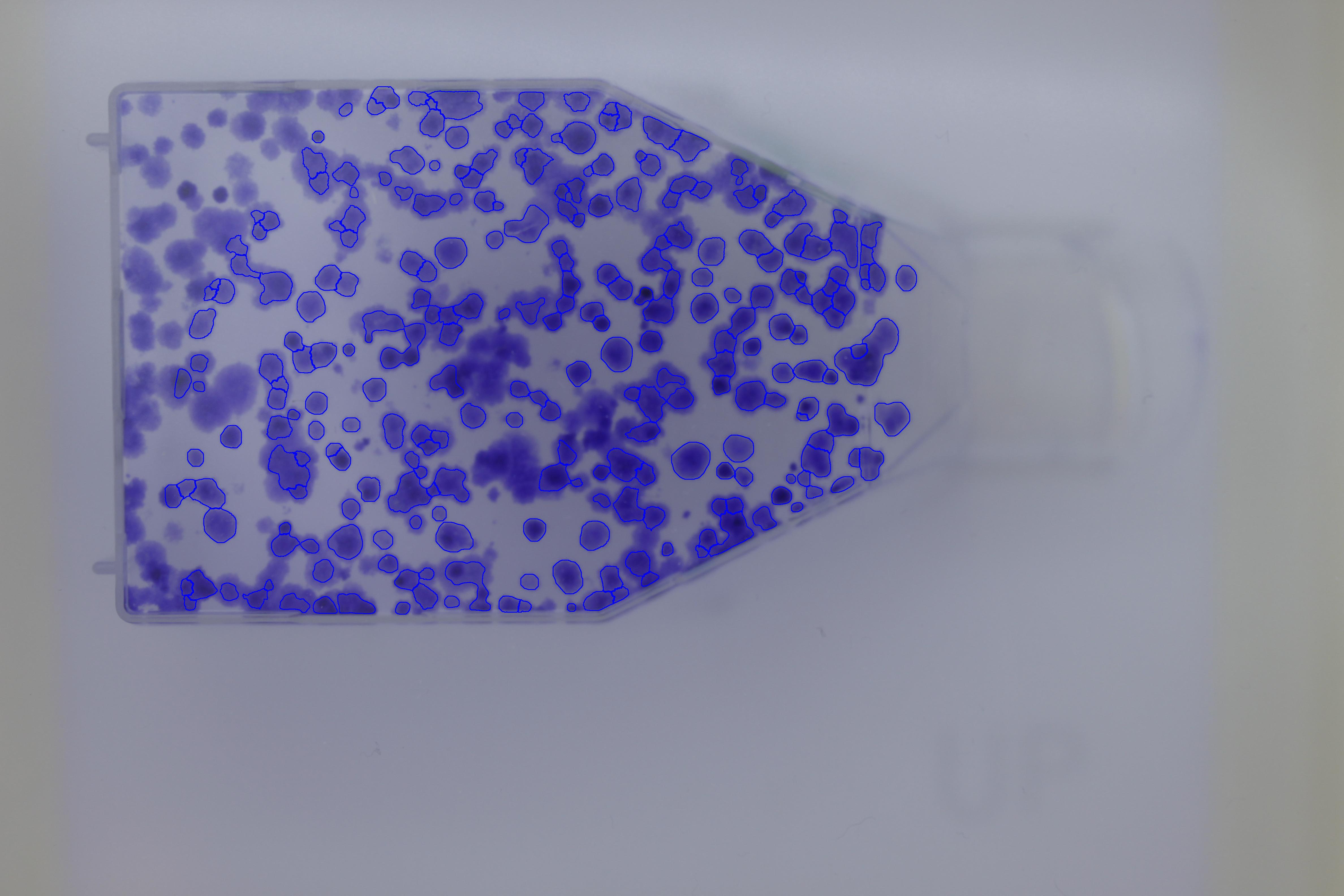

Supplement: S1 Comparison to others — (ZIP) [file pone.0205823.s007.zip › S1 Comparison to others/AutoCellSeg/180501 HeLa Flask/15_seg.jpg]

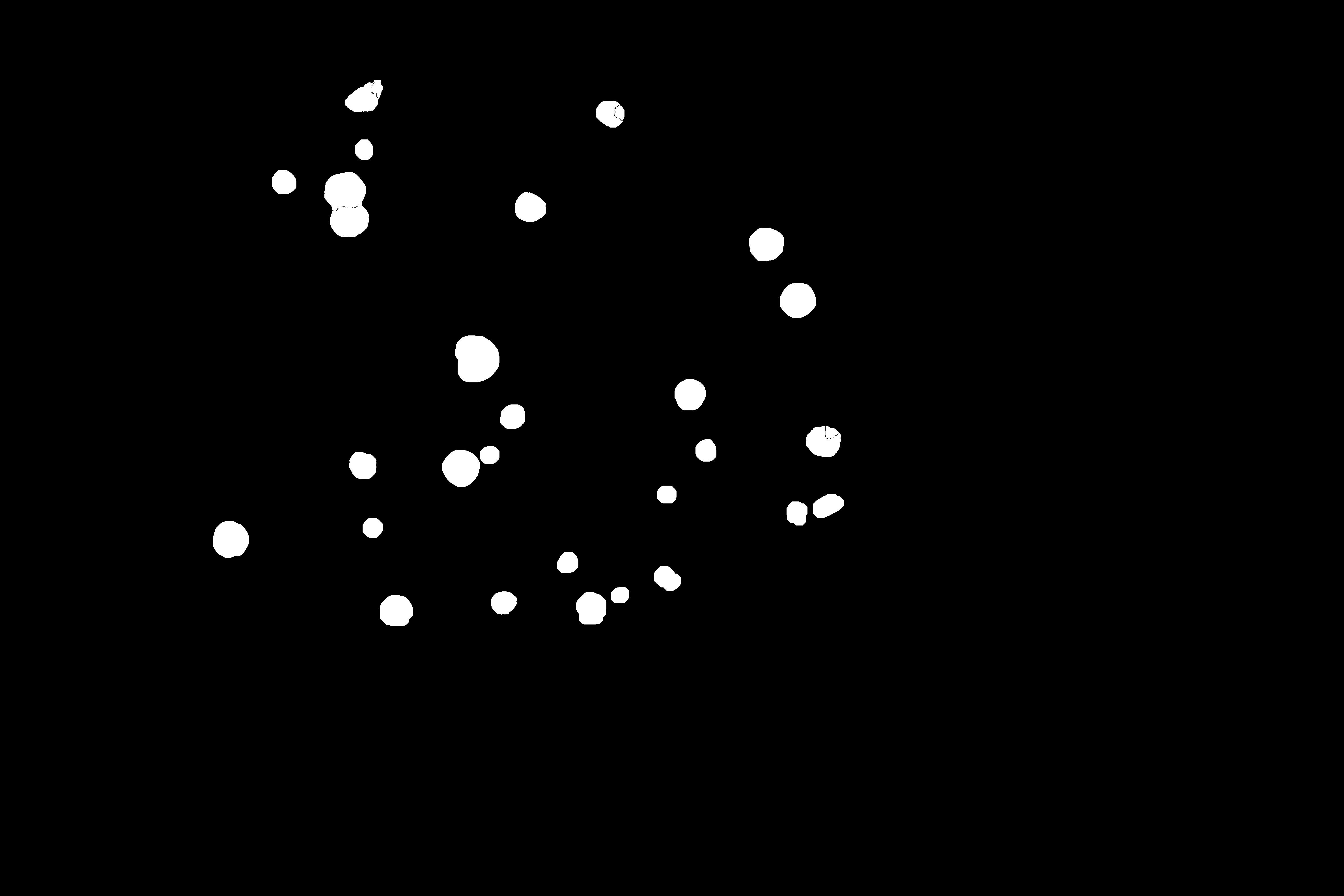

Supplement: S1 Comparison to others — (ZIP) [file pone.0205823.s007.zip › S1 Comparison to others/AutoCellSeg/180501 HeLa Flask/1_mask.jpg]

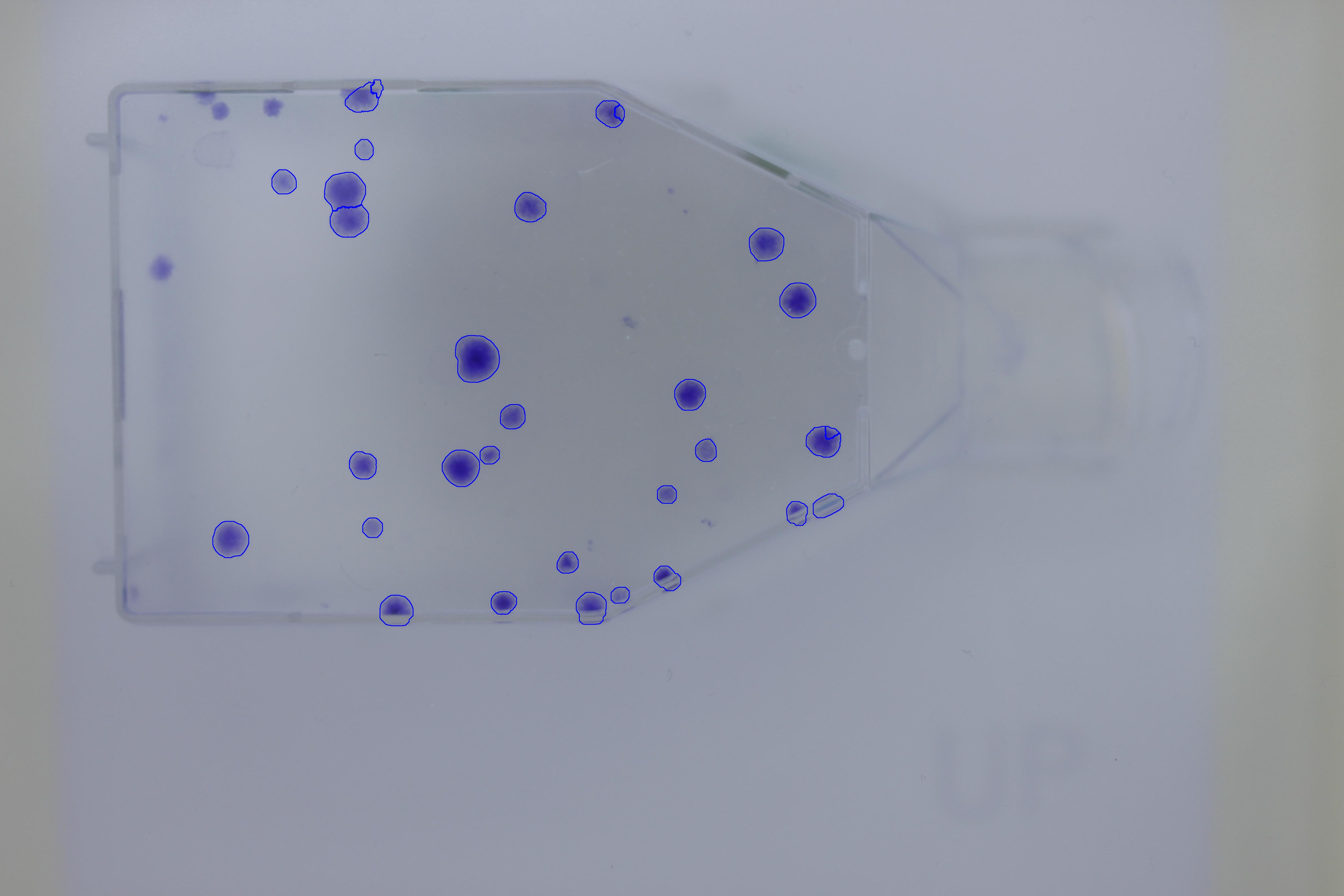

Supplement: S1 Comparison to others — (ZIP) [file pone.0205823.s007.zip › S1 Comparison to others/AutoCellSeg/180501 HeLa Flask/1_seg.jpg]

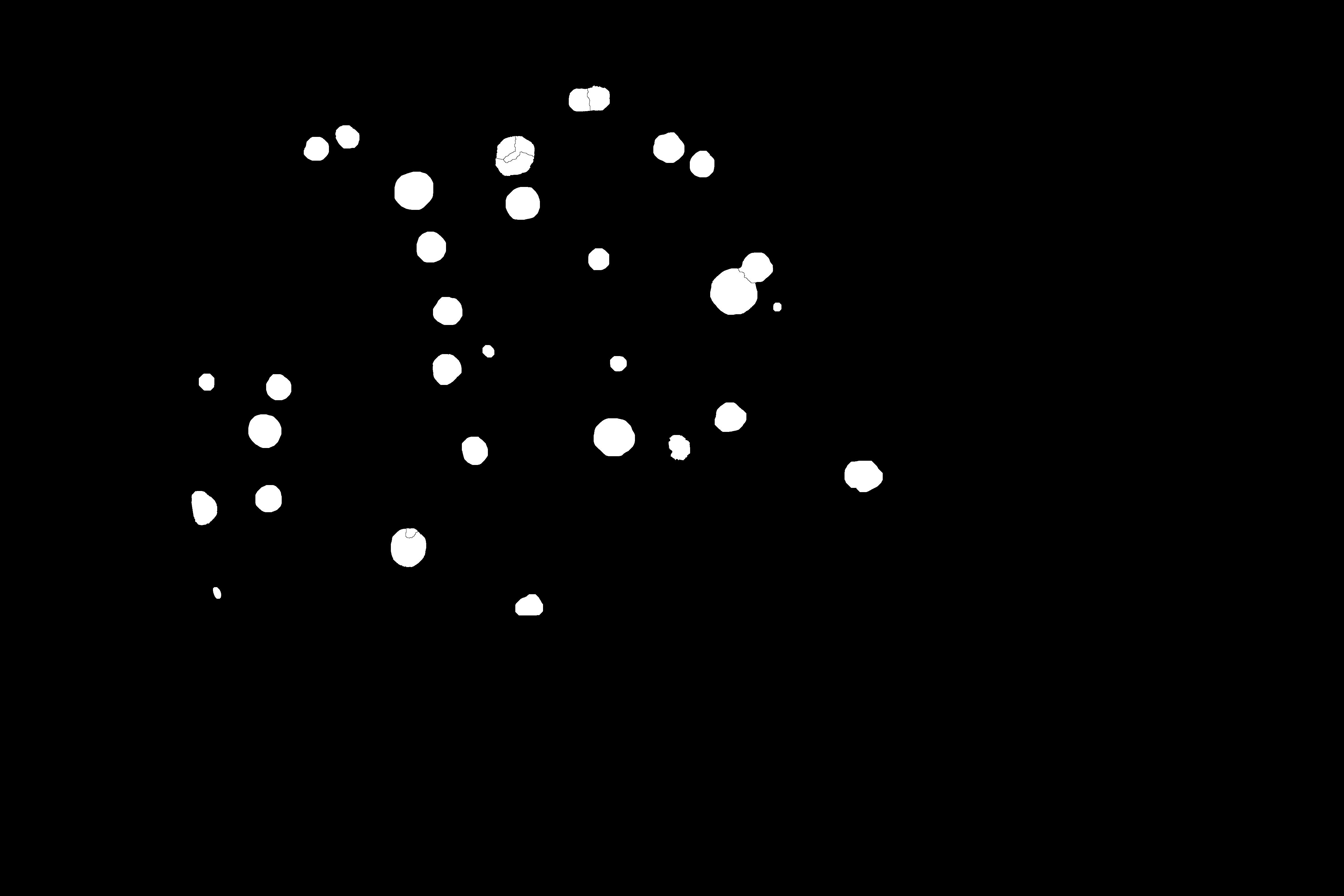

Supplement: S1 Comparison to others — (ZIP) [file pone.0205823.s007.zip › S1 Comparison to others/AutoCellSeg/180501 HeLa Flask/2_mask.jpg]

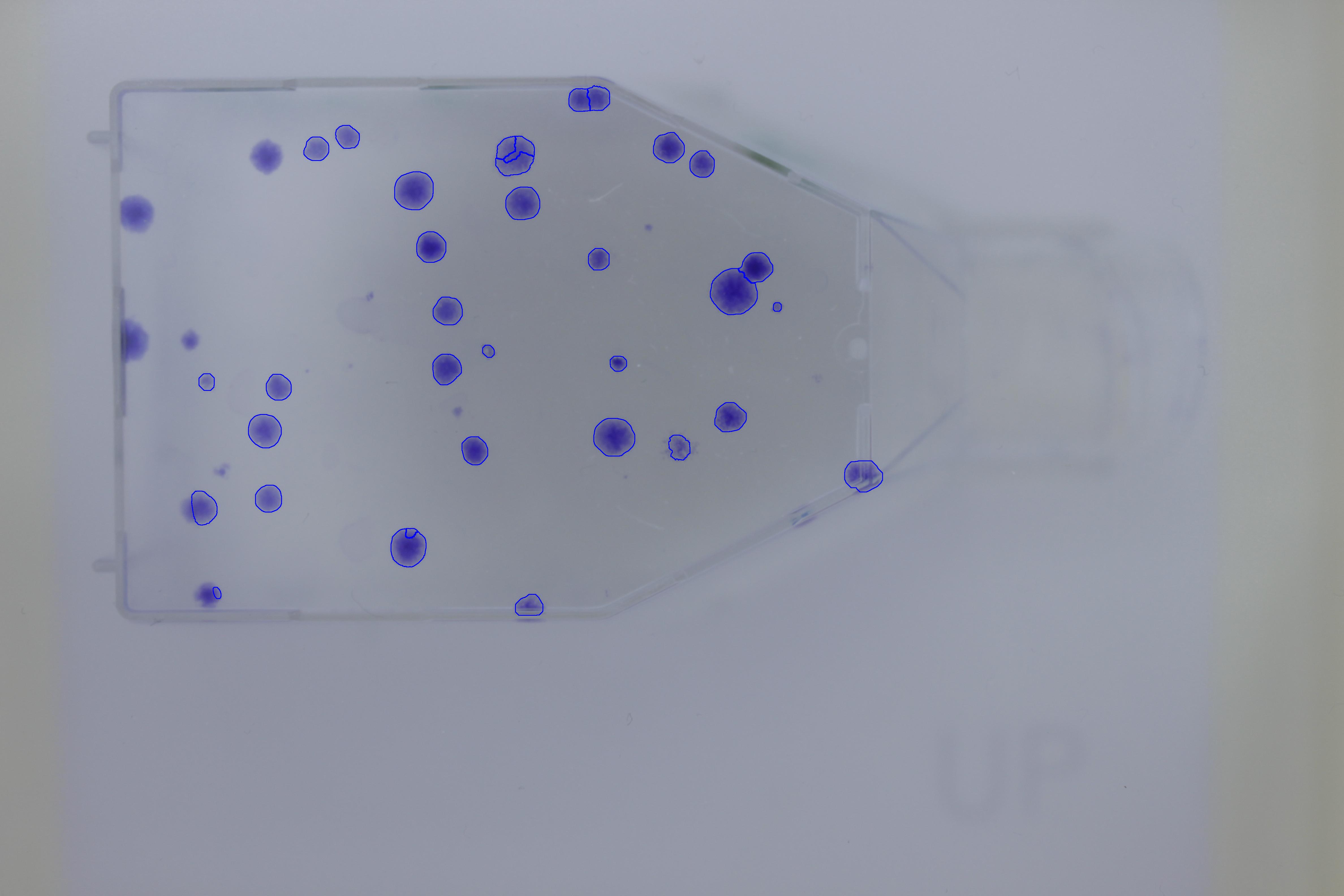

Supplement: S1 Comparison to others — (ZIP) [file pone.0205823.s007.zip › S1 Comparison to others/AutoCellSeg/180501 HeLa Flask/2_seg.jpg]

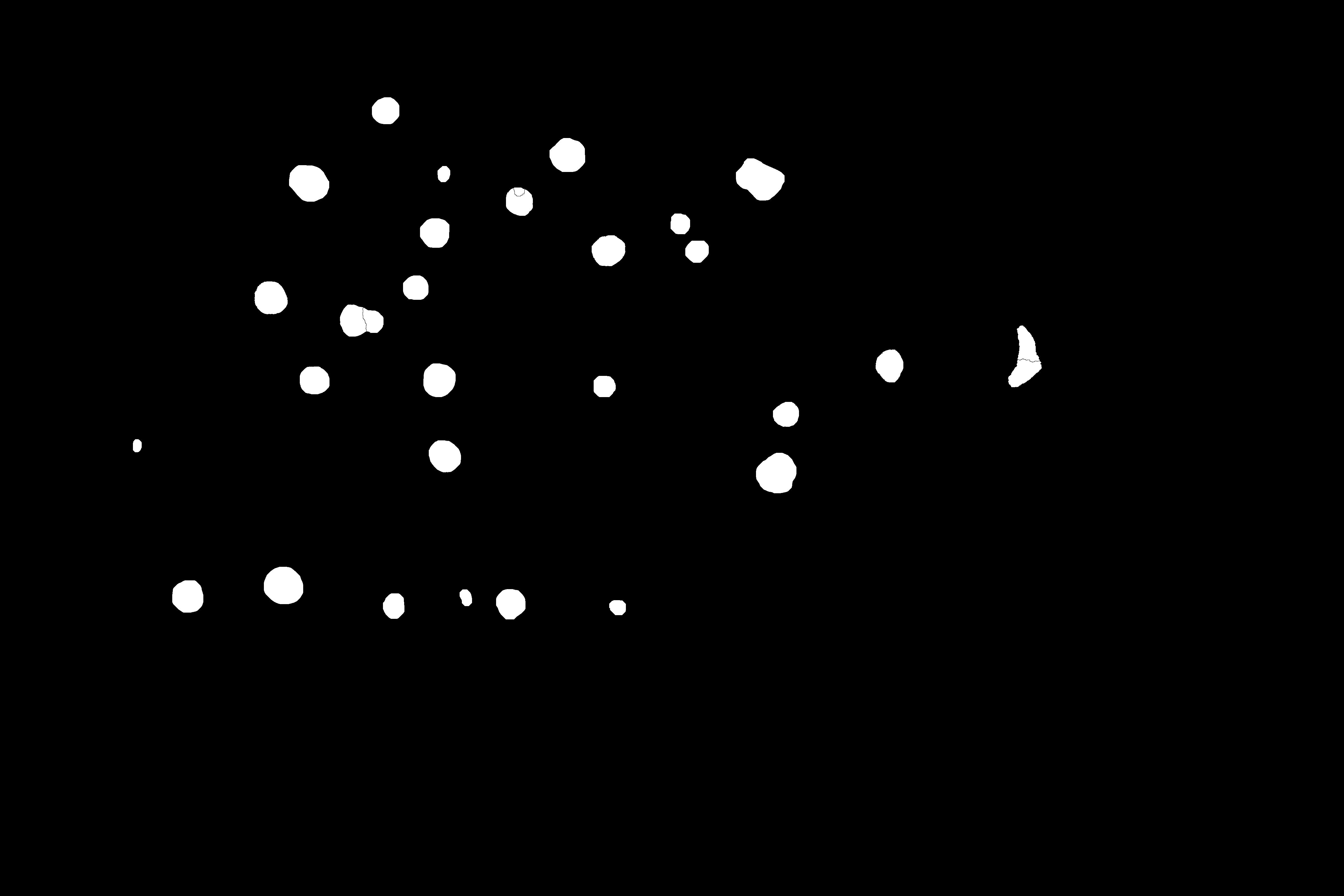

Supplement: S1 Comparison to others — (ZIP) [file pone.0205823.s007.zip › S1 Comparison to others/AutoCellSeg/180501 HeLa Flask/3_mask.jpg]

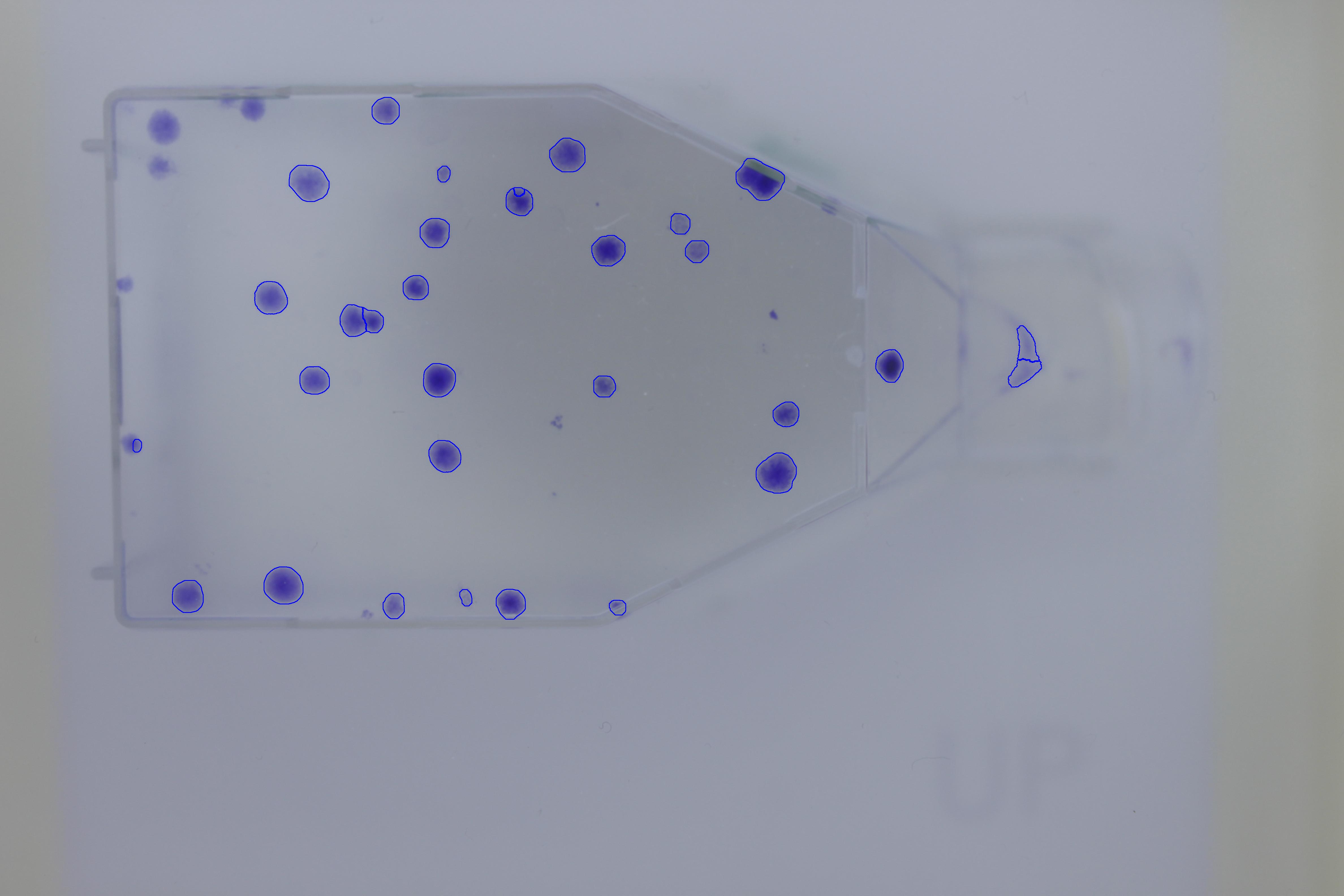

Supplement: S1 Comparison to others — (ZIP) [file pone.0205823.s007.zip › S1 Comparison to others/AutoCellSeg/180501 HeLa Flask/3_seg.jpg]

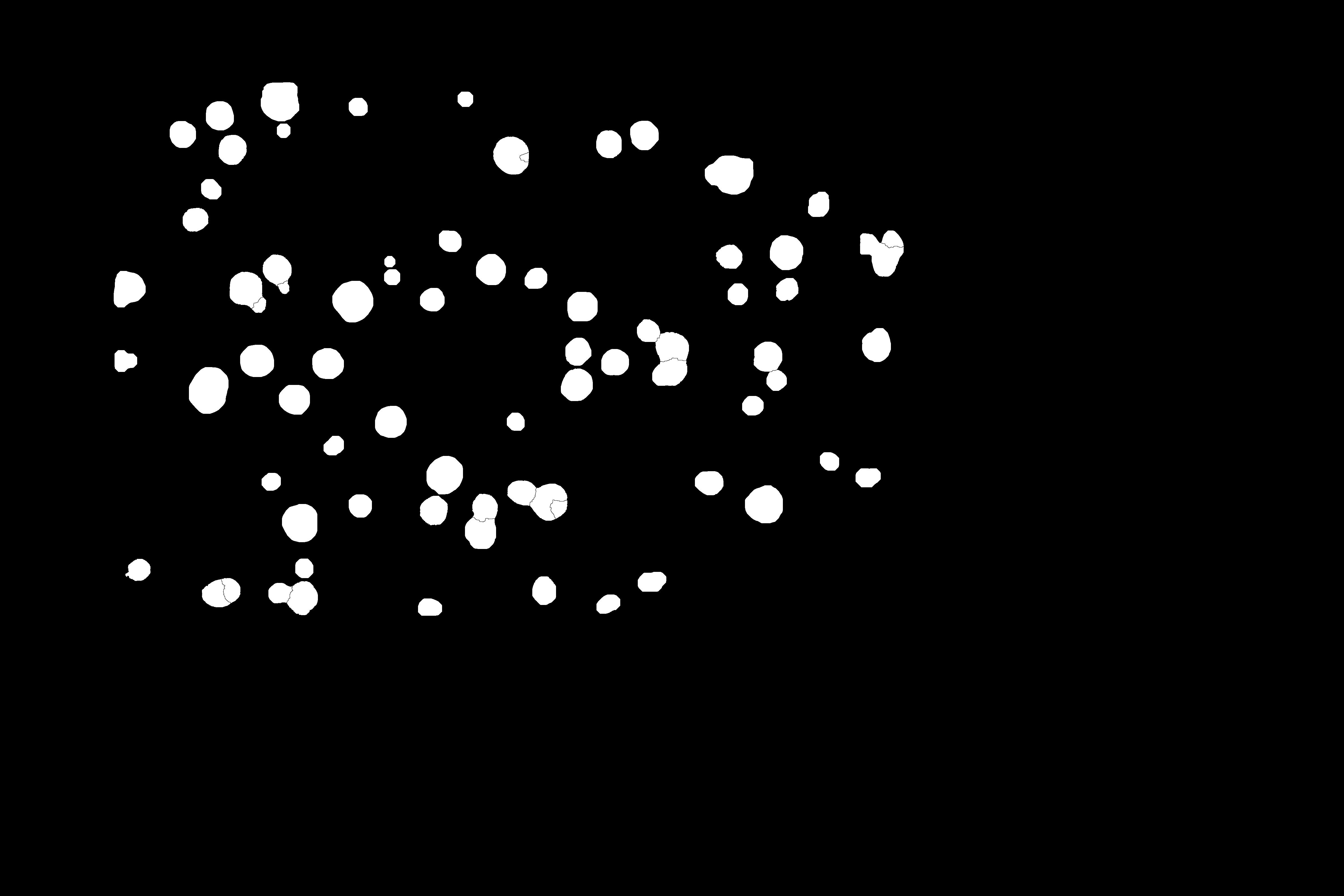

Supplement: S1 Comparison to others — (ZIP) [file pone.0205823.s007.zip › S1 Comparison to others/AutoCellSeg/180501 HeLa Flask/4_mask.jpg]

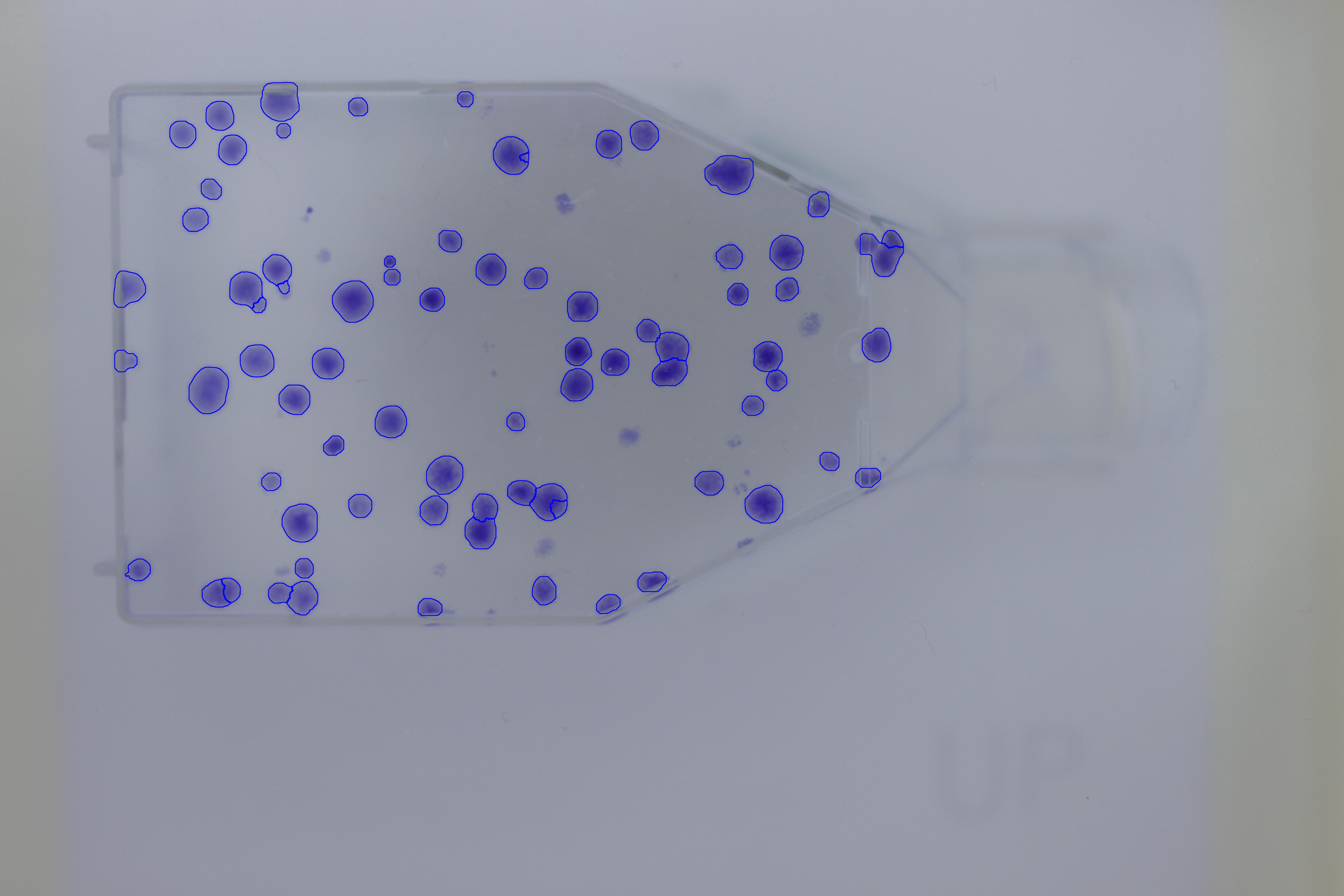

Supplement: S1 Comparison to others — (ZIP) [file pone.0205823.s007.zip › S1 Comparison to others/AutoCellSeg/180501 HeLa Flask/4_seg.jpg]

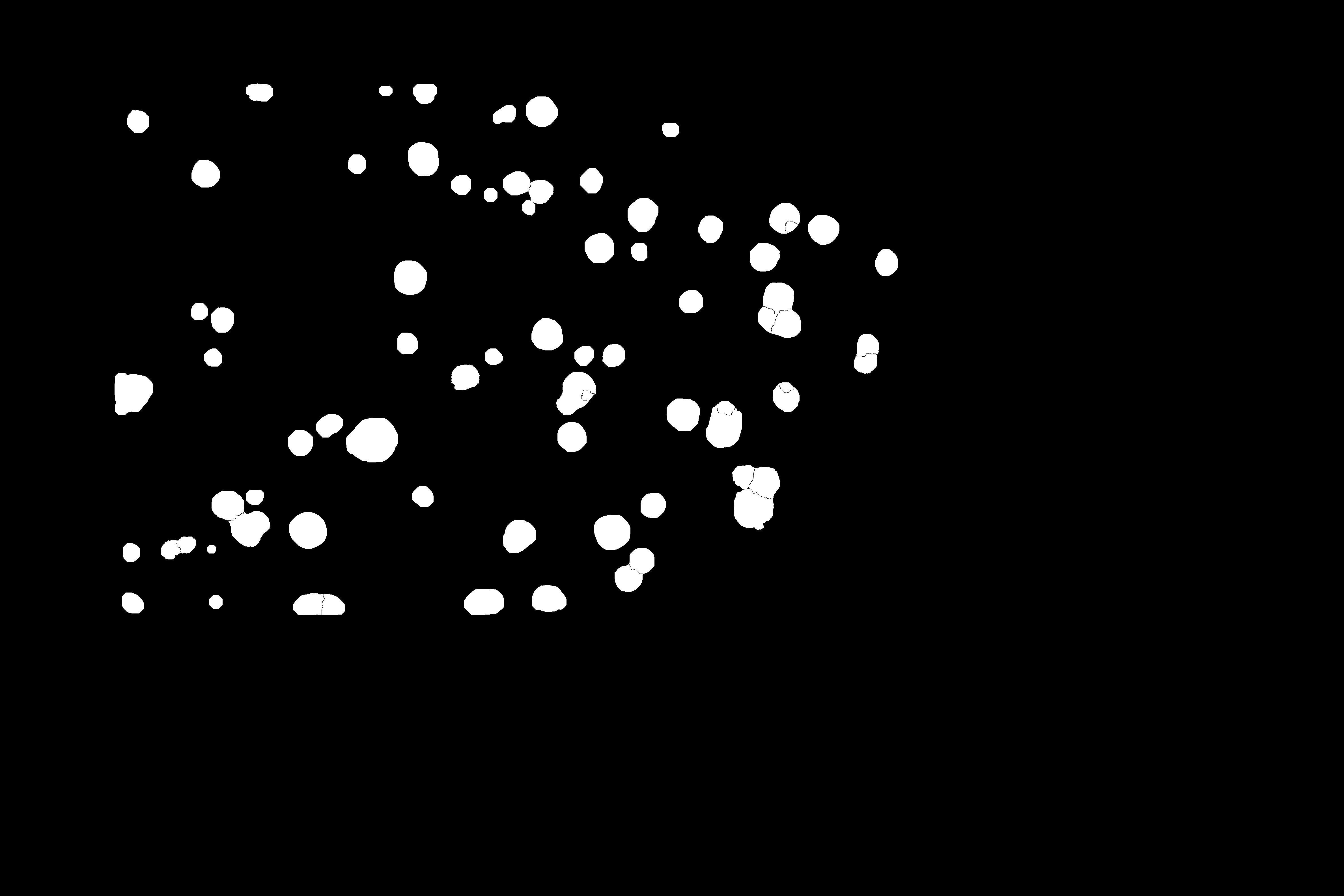

Supplement: S1 Comparison to others — (ZIP) [file pone.0205823.s007.zip › S1 Comparison to others/AutoCellSeg/180501 HeLa Flask/5_mask.jpg]

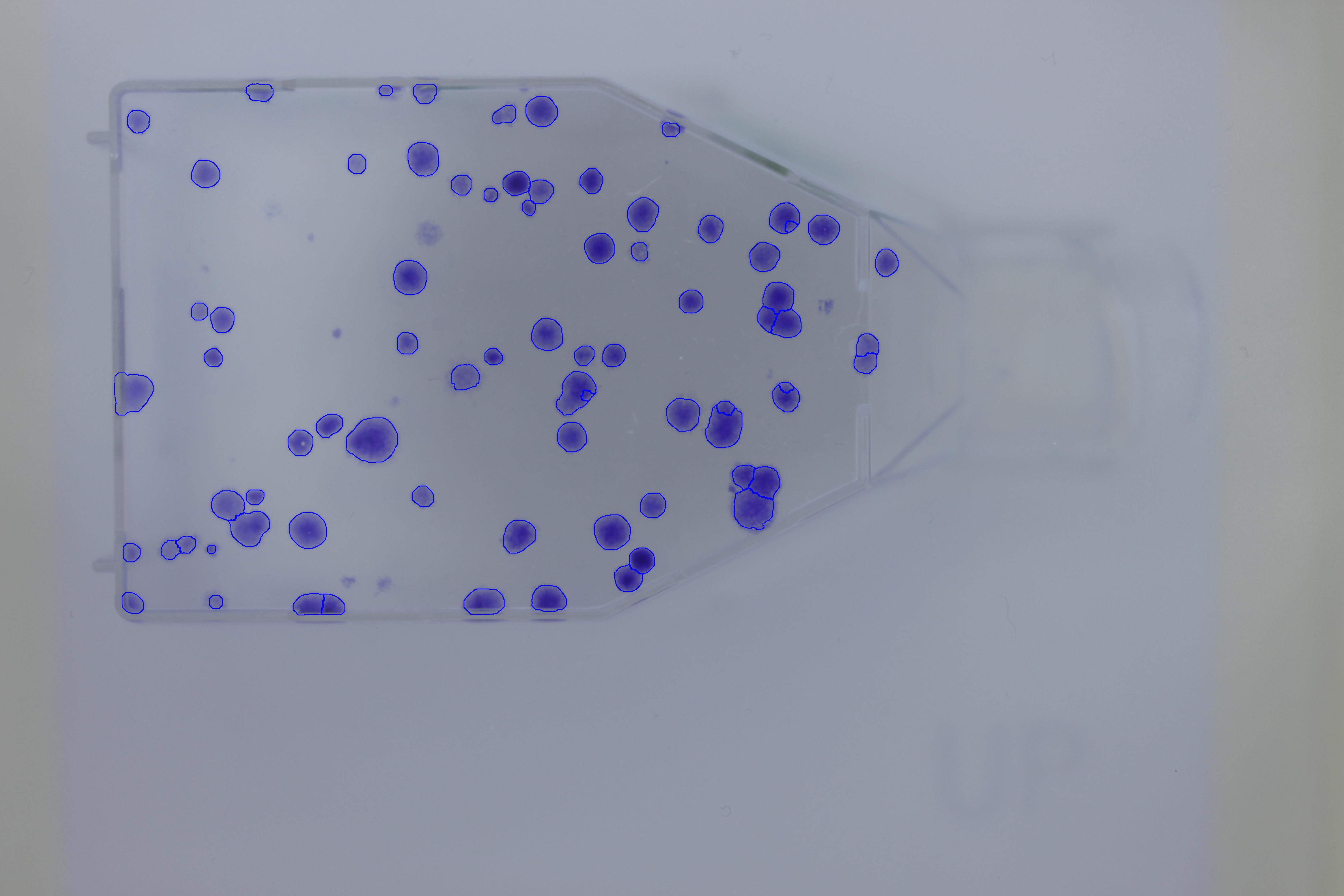

Supplement: S1 Comparison to others — (ZIP) [file pone.0205823.s007.zip › S1 Comparison to others/AutoCellSeg/180501 HeLa Flask/5_seg.jpg]

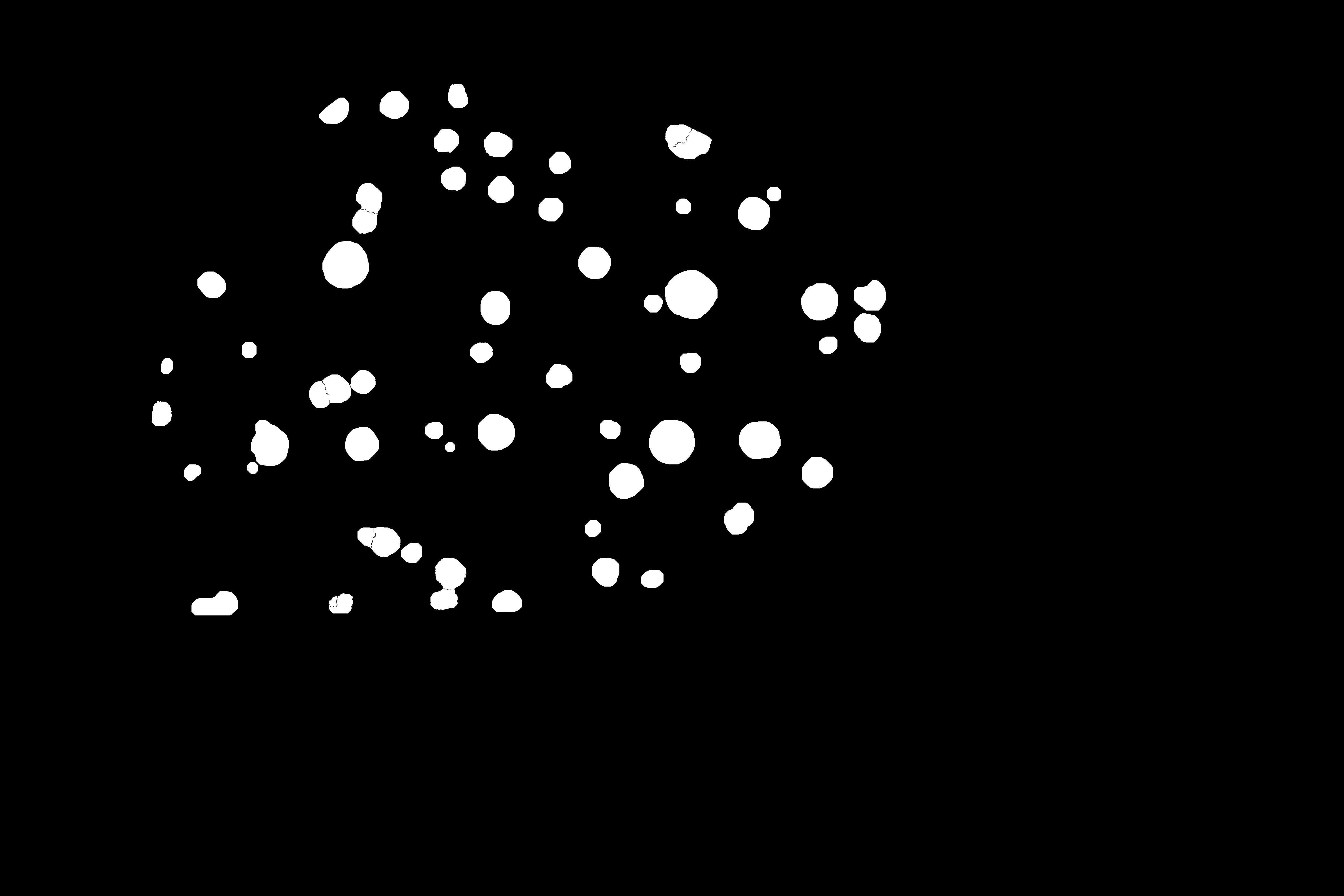

Supplement: S1 Comparison to others — (ZIP) [file pone.0205823.s007.zip › S1 Comparison to others/AutoCellSeg/180501 HeLa Flask/6_mask.jpg]

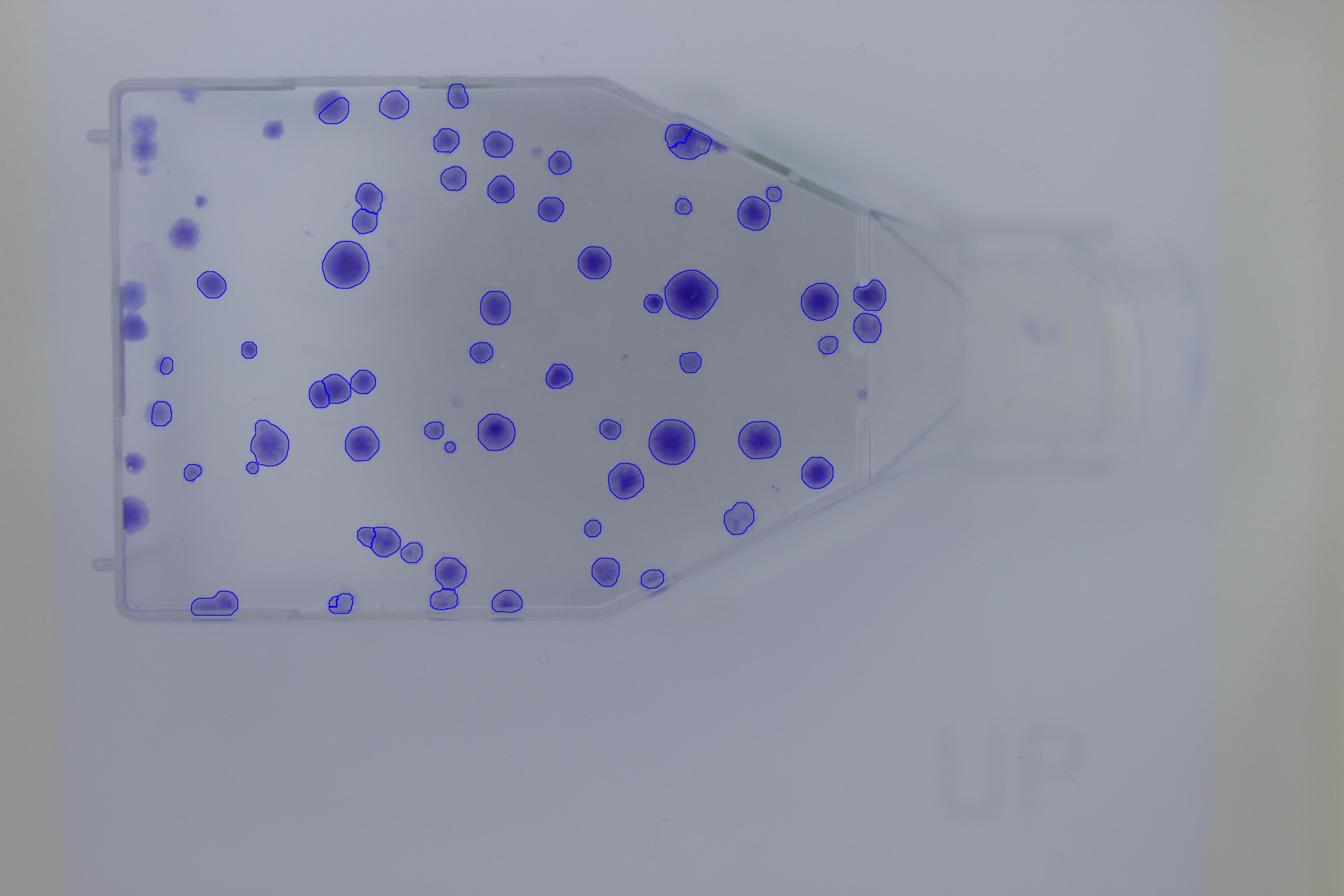

Supplement: S1 Comparison to others — (ZIP) [file pone.0205823.s007.zip › S1 Comparison to others/AutoCellSeg/180501 HeLa Flask/6_seg.jpg]

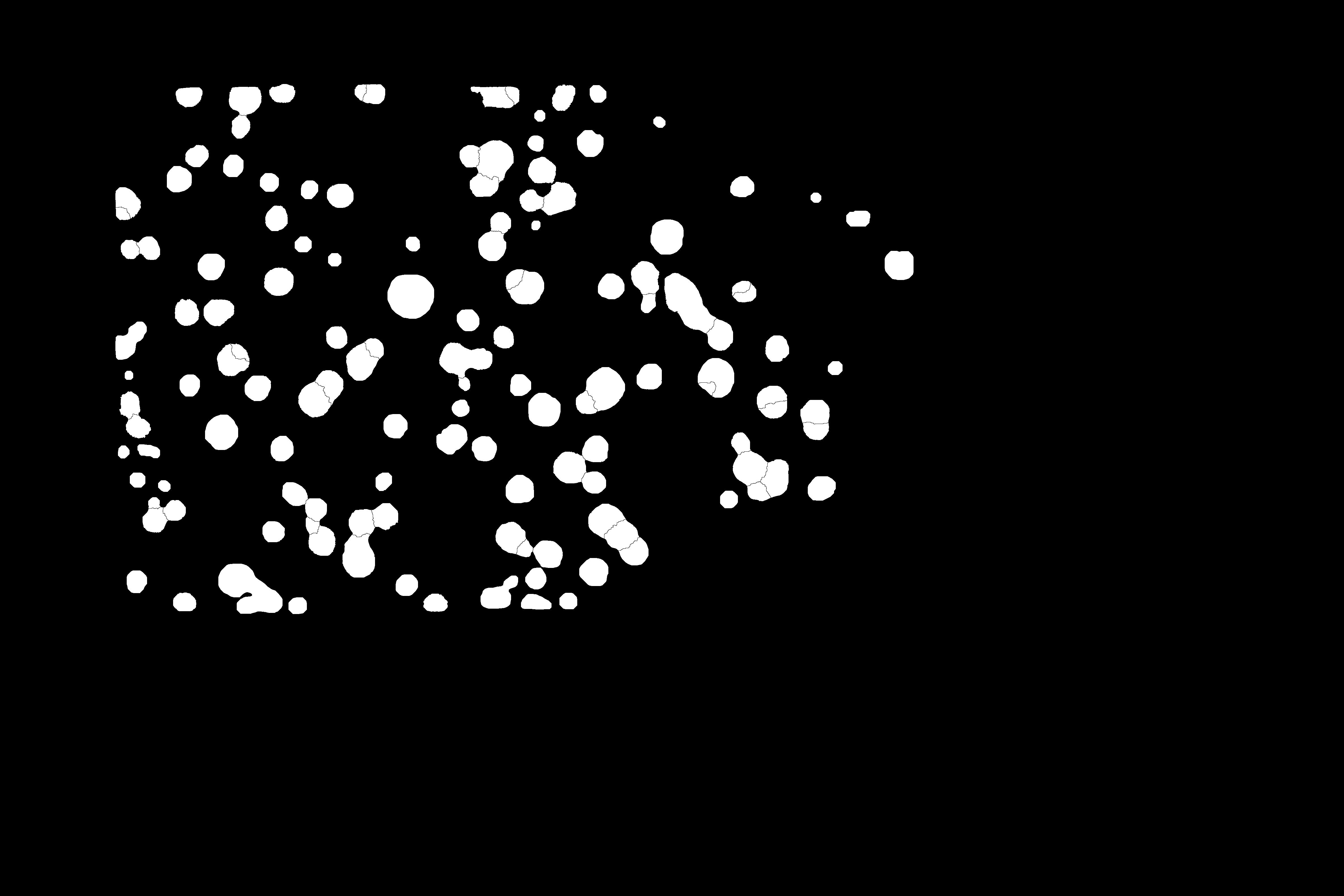

Supplement: S1 Comparison to others — (ZIP) [file pone.0205823.s007.zip › S1 Comparison to others/AutoCellSeg/180501 HeLa Flask/7_mask.jpg]

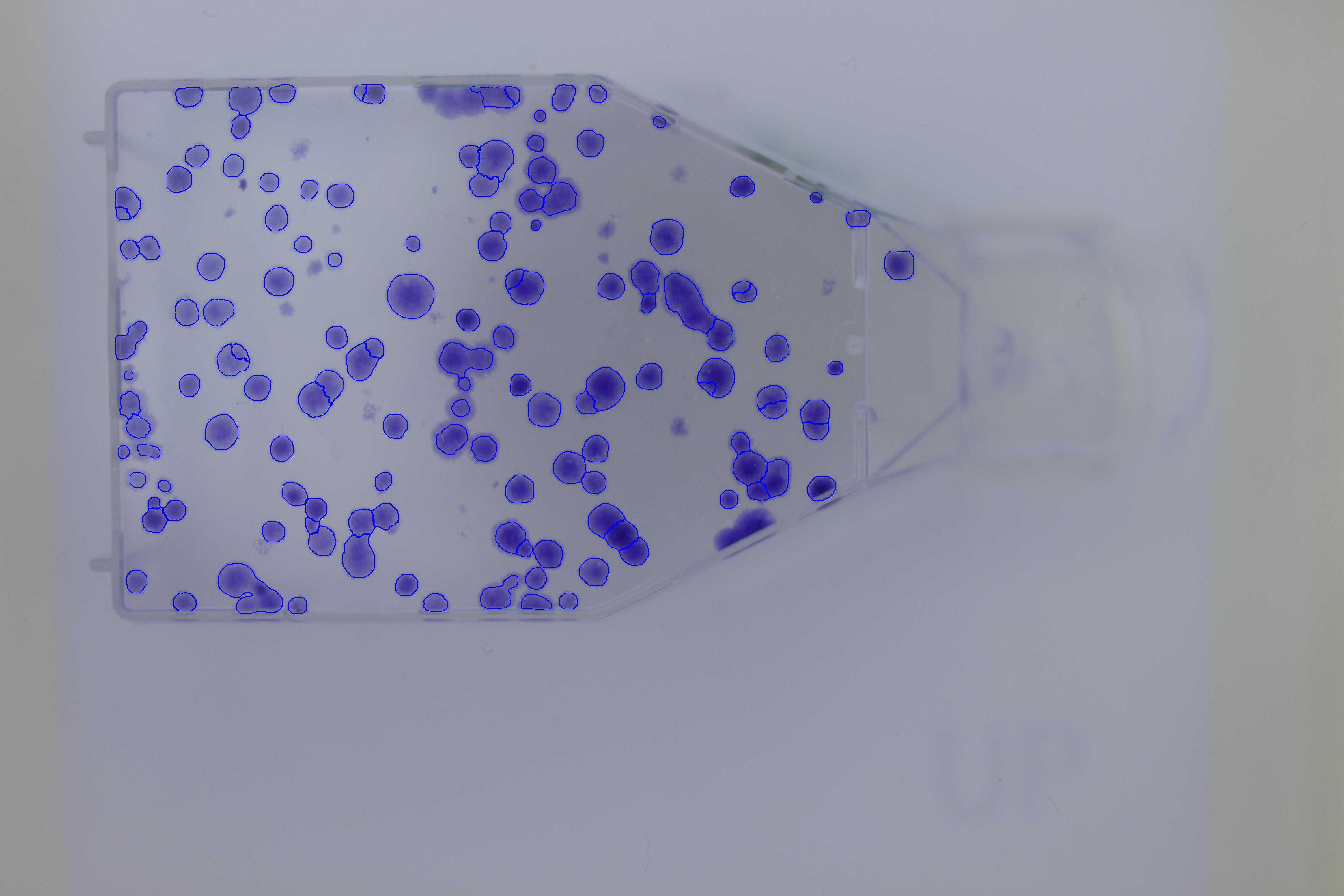

Supplement: S1 Comparison to others — (ZIP) [file pone.0205823.s007.zip › S1 Comparison to others/AutoCellSeg/180501 HeLa Flask/7_seg.jpg]

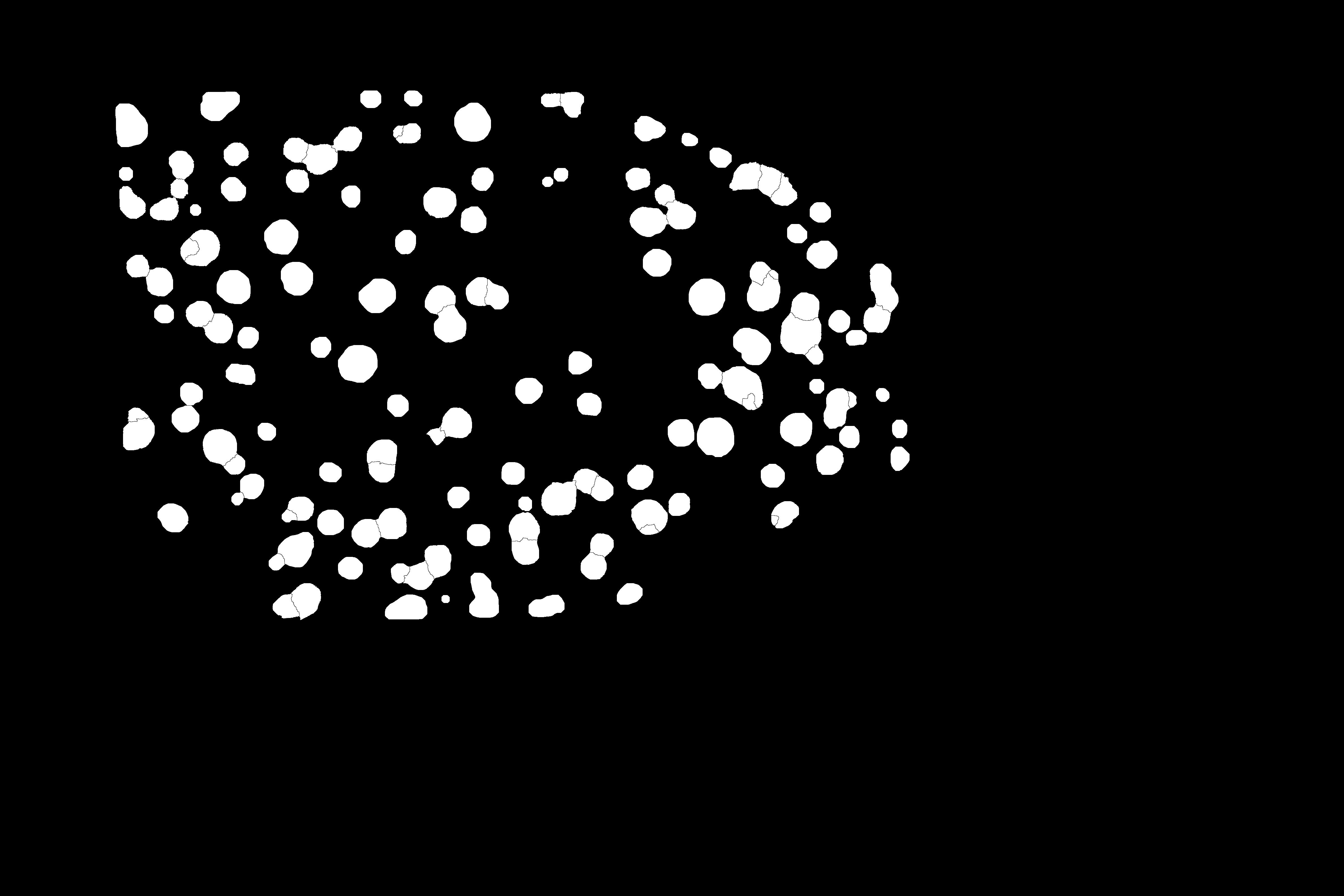

Supplement: S1 Comparison to others — (ZIP) [file pone.0205823.s007.zip › S1 Comparison to others/AutoCellSeg/180501 HeLa Flask/8_mask.jpg]

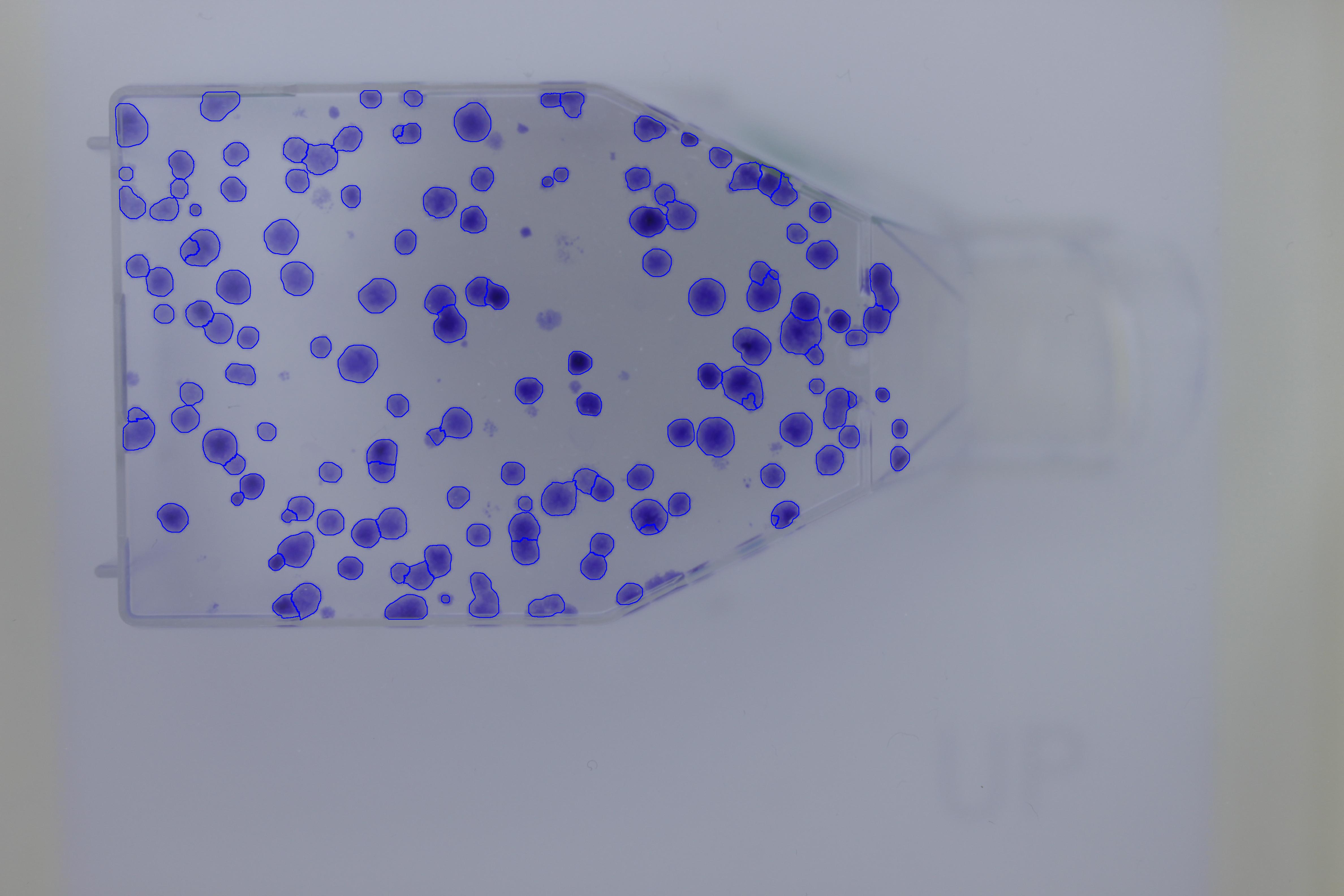

Supplement: S1 Comparison to others — (ZIP) [file pone.0205823.s007.zip › S1 Comparison to others/AutoCellSeg/180501 HeLa Flask/8_seg.jpg]

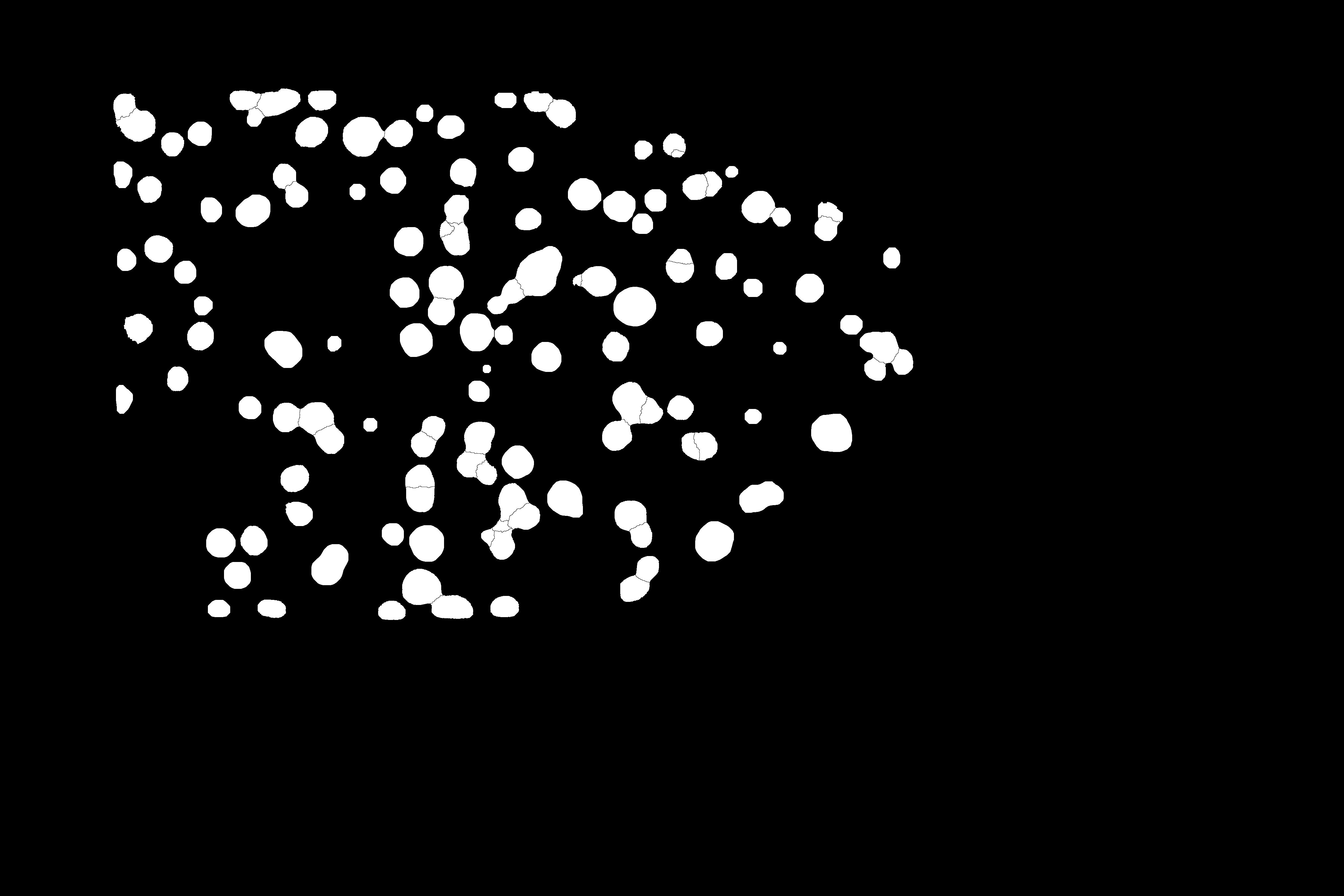

Supplement: S1 Comparison to others — (ZIP) [file pone.0205823.s007.zip › S1 Comparison to others/AutoCellSeg/180501 HeLa Flask/9_mask.jpg]

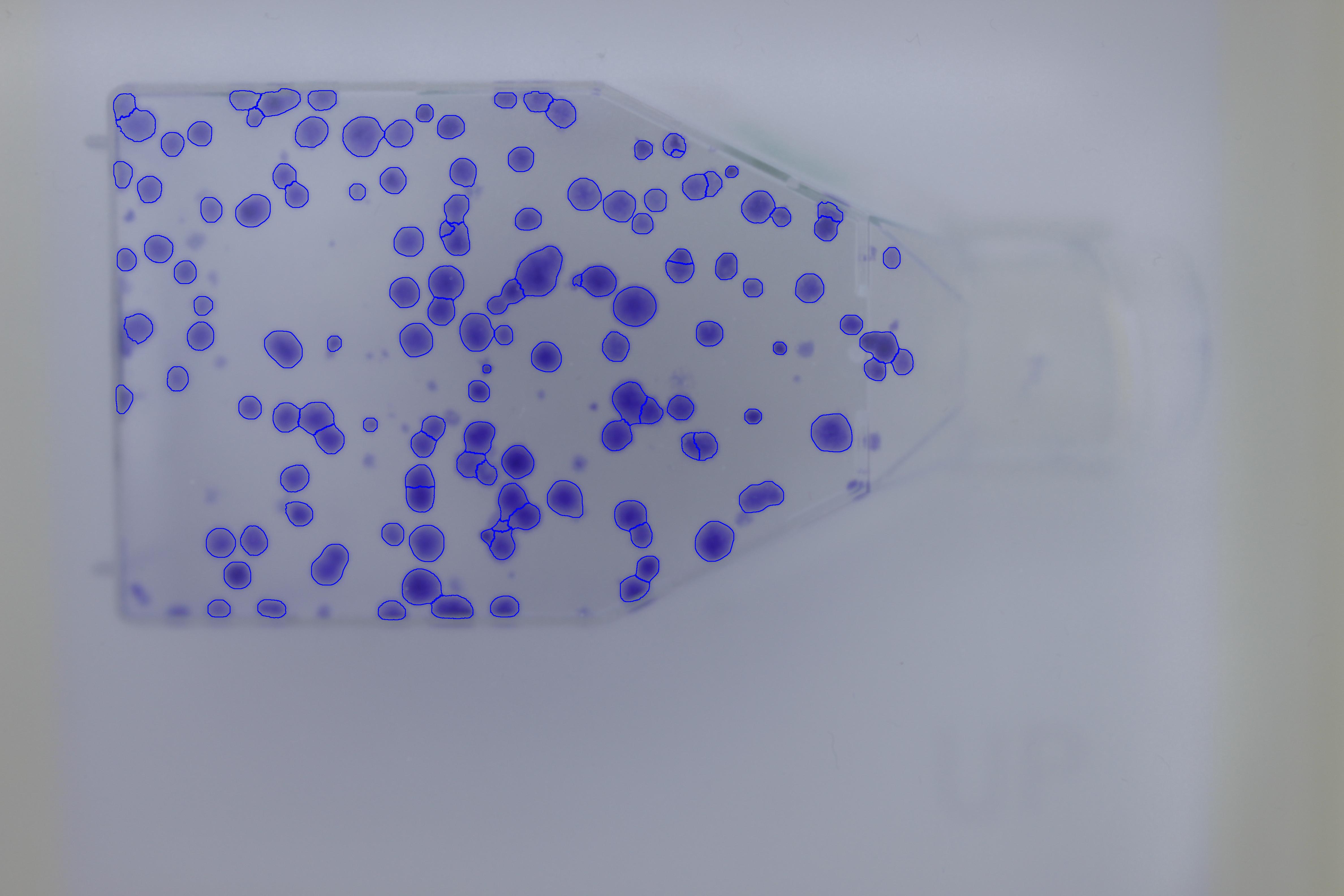

Supplement: S1 Comparison to others — (ZIP) [file pone.0205823.s007.zip › S1 Comparison to others/AutoCellSeg/180501 HeLa Flask/9_seg.jpg]

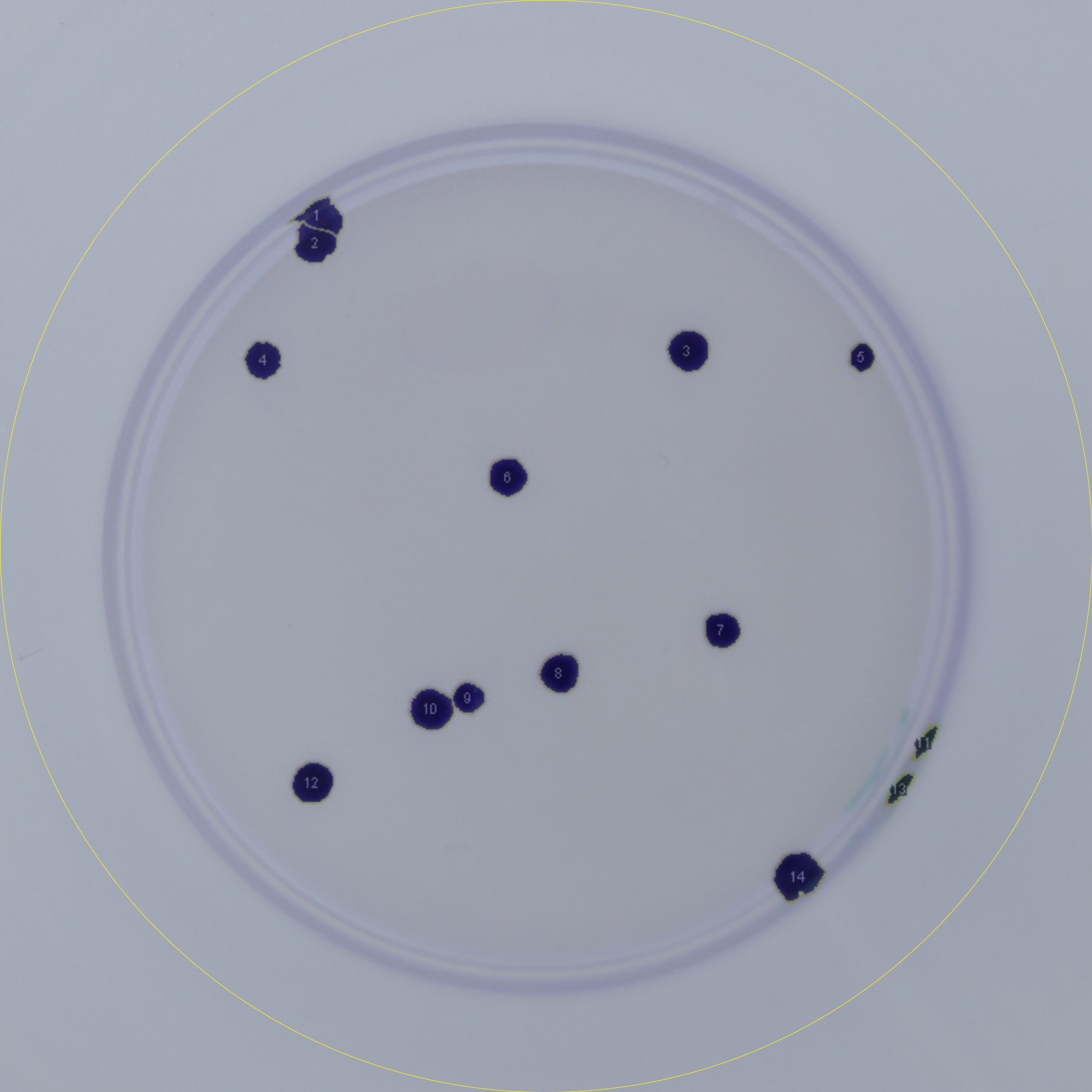

Supplement: S1 Comparison to others — (ZIP) [file pone.0205823.s007.zip › S1 Comparison to others/CAI/171214 V79 Dish/1 Results.jpg]

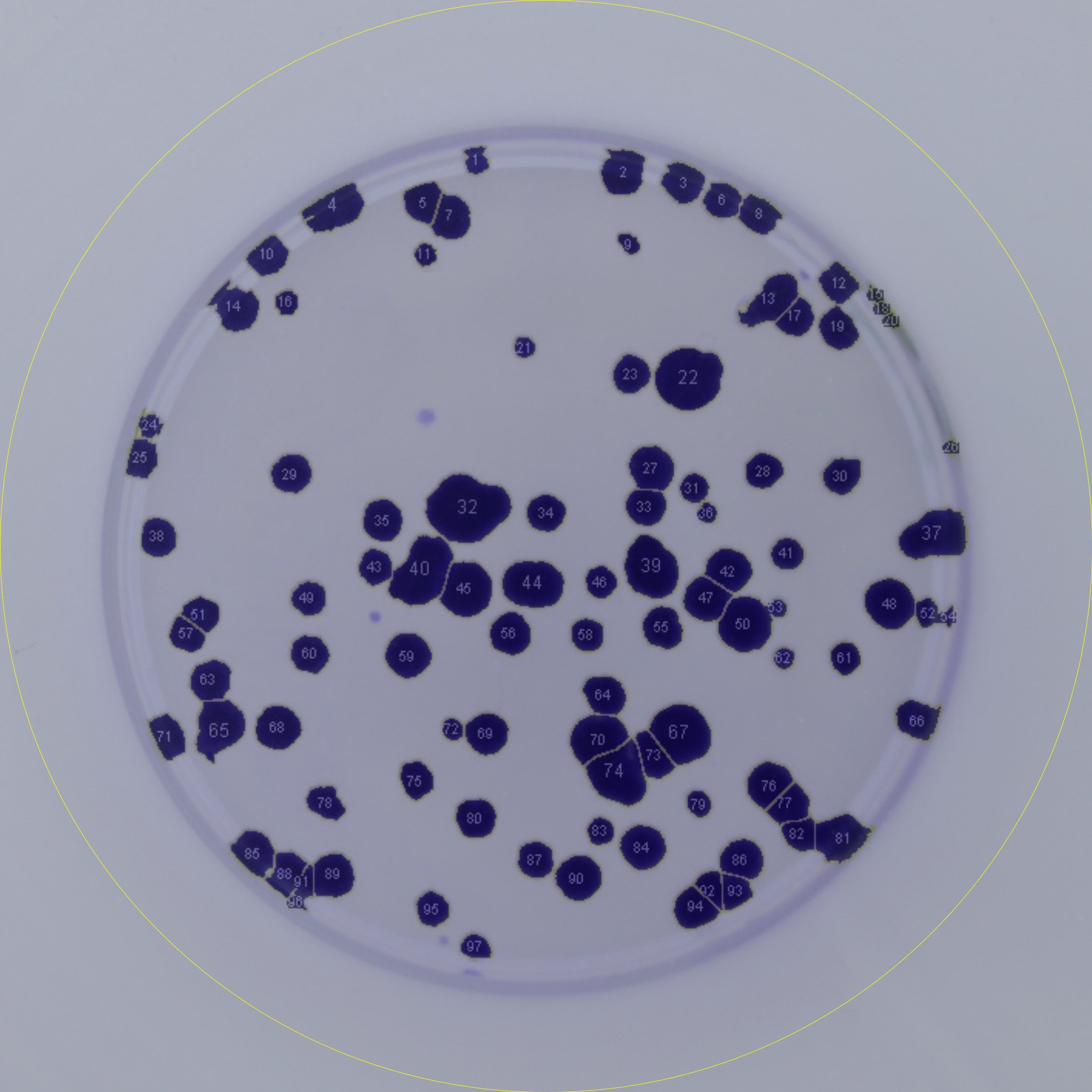

Supplement: S1 Comparison to others — (ZIP) [file pone.0205823.s007.zip › S1 Comparison to others/CAI/171214 V79 Dish/10 Results.jpg]

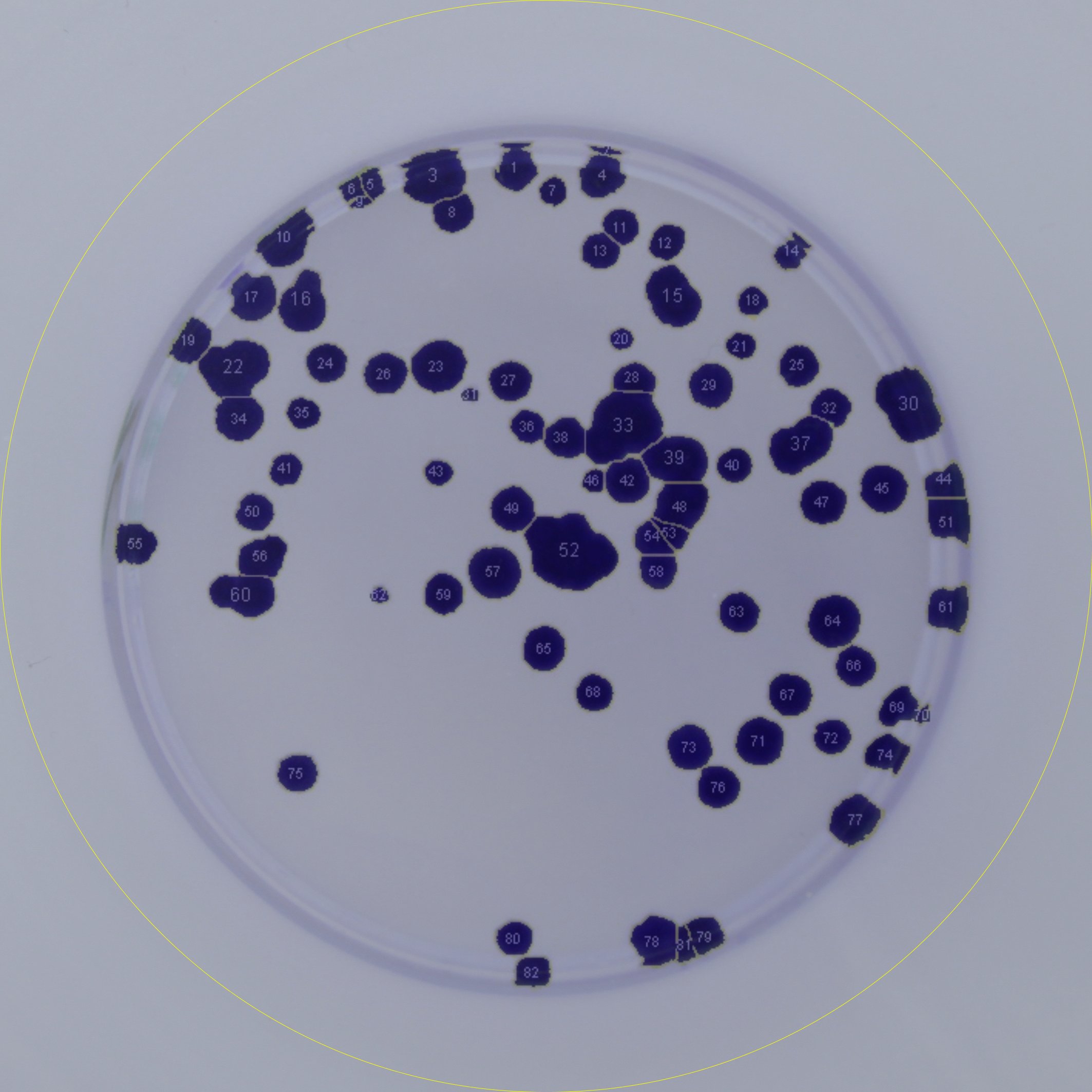

Supplement: S1 Comparison to others — (ZIP) [file pone.0205823.s007.zip › S1 Comparison to others/CAI/171214 V79 Dish/11 Results.jpg]

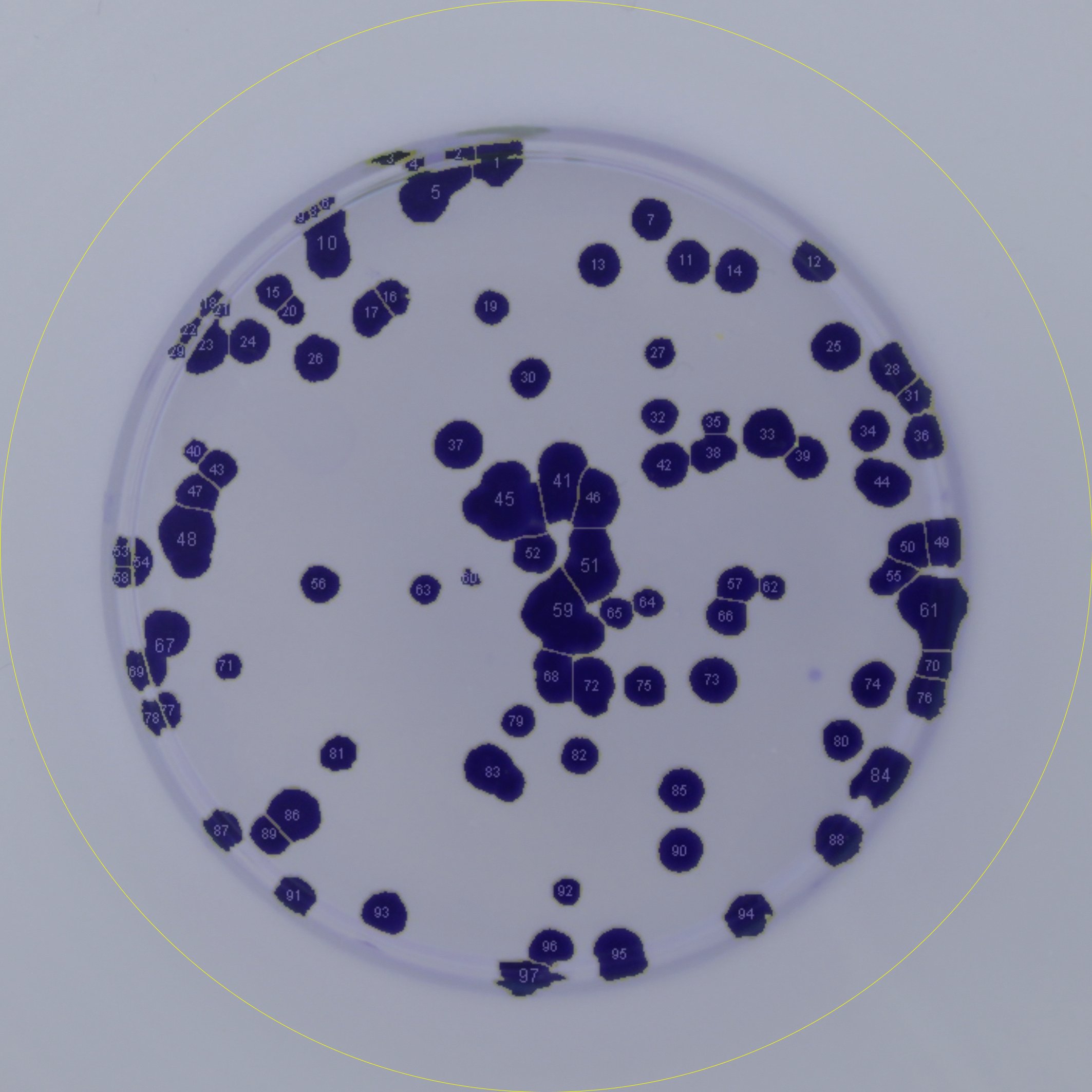

Supplement: S1 Comparison to others — (ZIP) [file pone.0205823.s007.zip › S1 Comparison to others/CAI/171214 V79 Dish/12 Results.jpg]

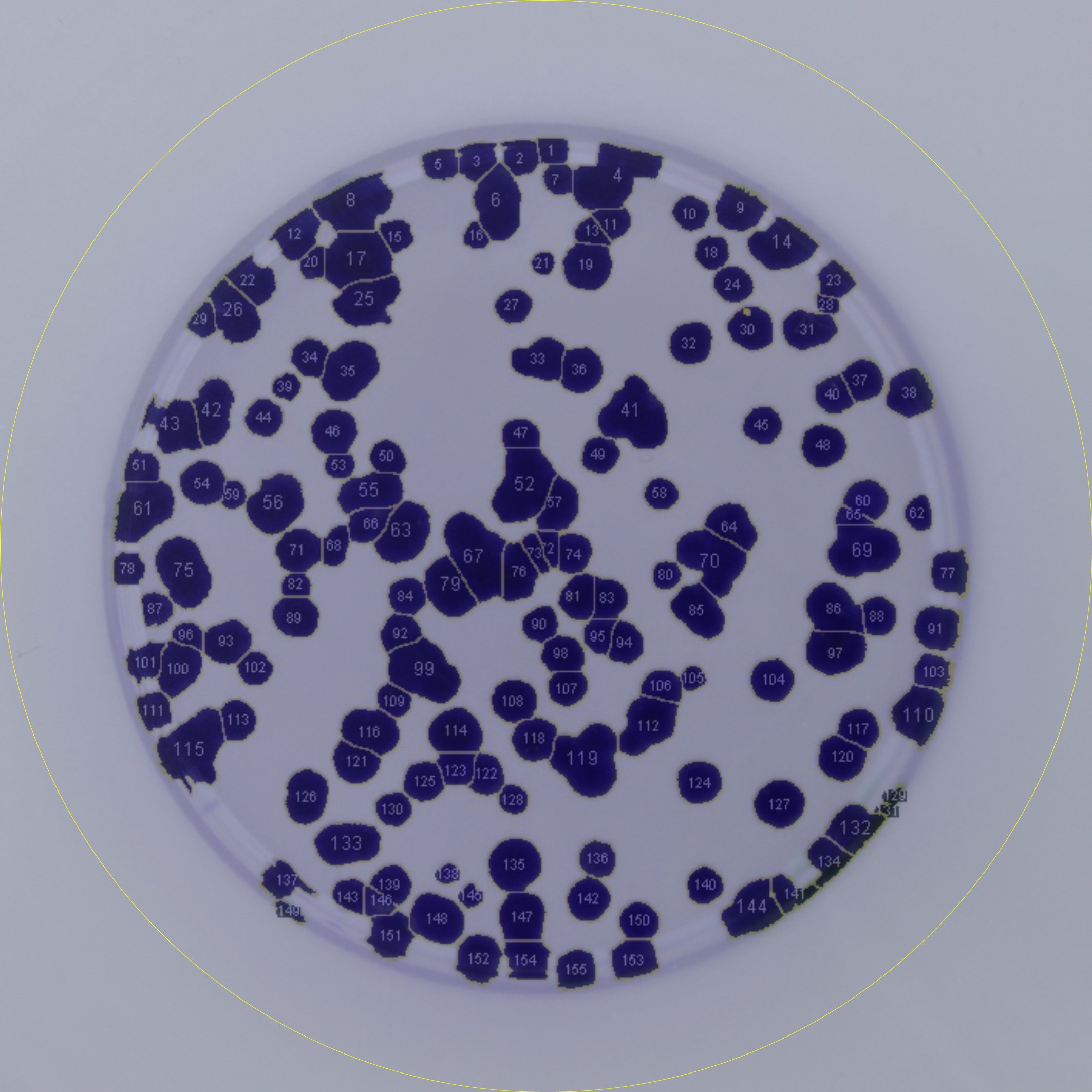

Supplement: S1 Comparison to others — (ZIP) [file pone.0205823.s007.zip › S1 Comparison to others/CAI/171214 V79 Dish/13 Results.jpg]

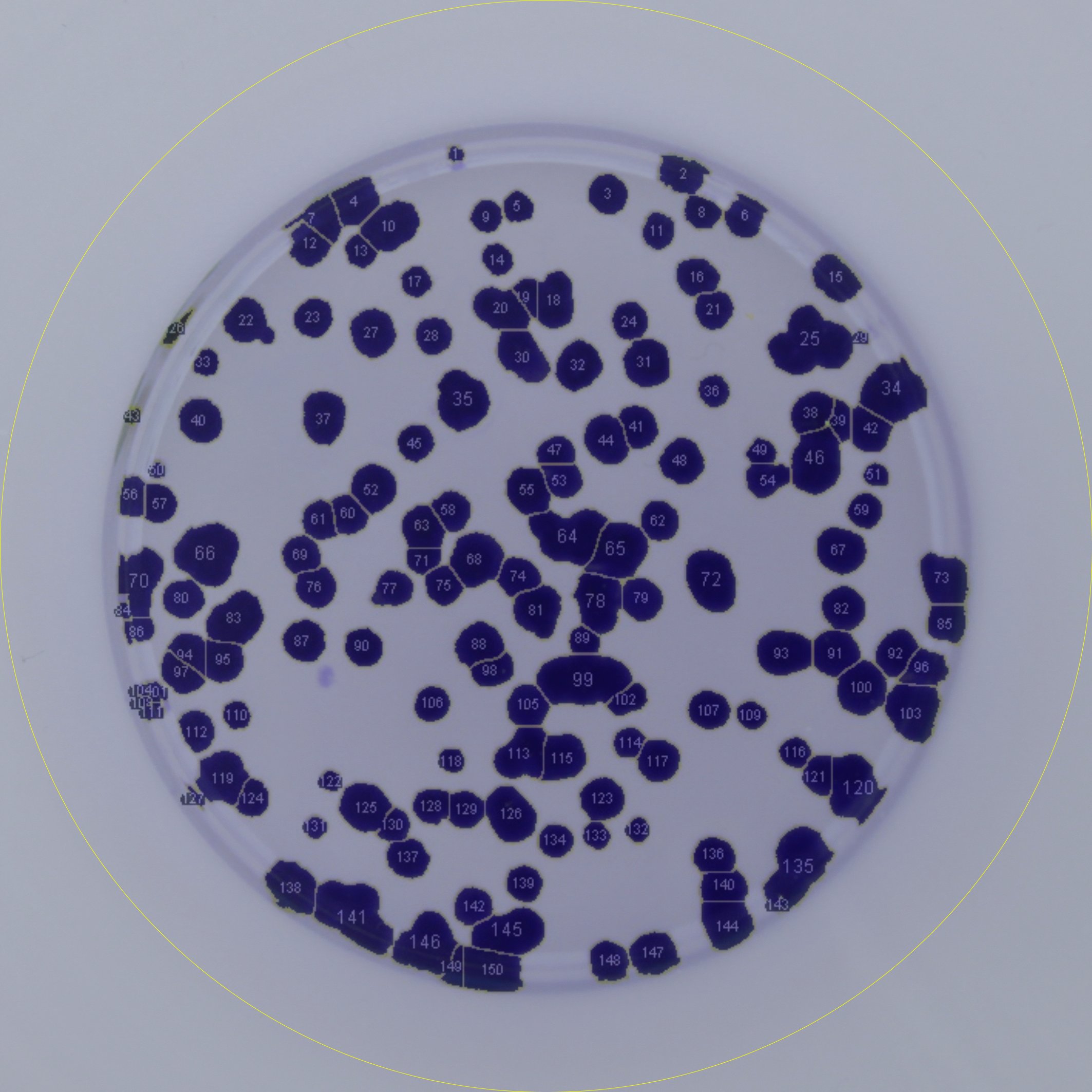

Supplement: S1 Comparison to others — (ZIP) [file pone.0205823.s007.zip › S1 Comparison to others/CAI/171214 V79 Dish/14 Results.jpg]

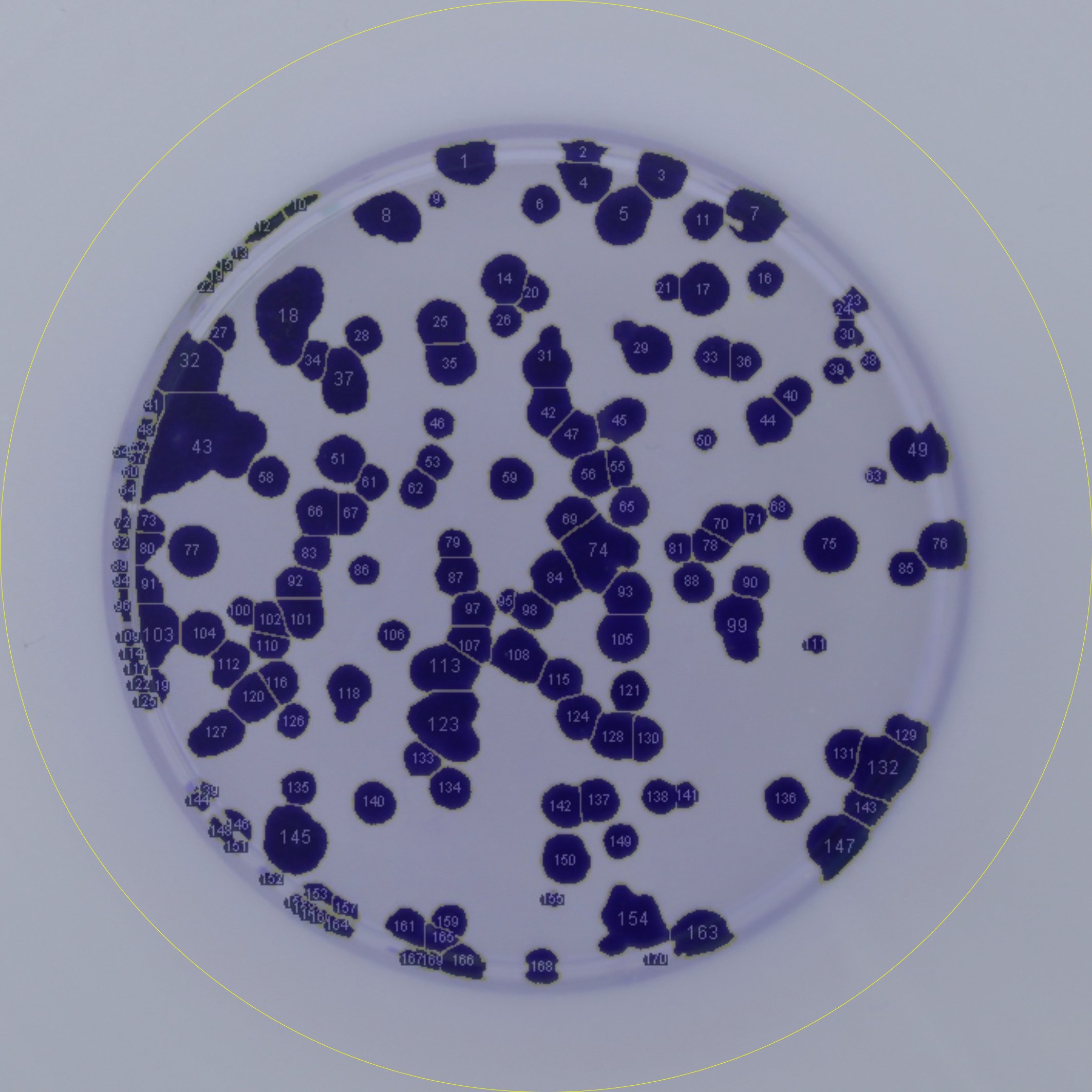

Supplement: S1 Comparison to others — (ZIP) [file pone.0205823.s007.zip › S1 Comparison to others/CAI/171214 V79 Dish/15 Results.jpg]

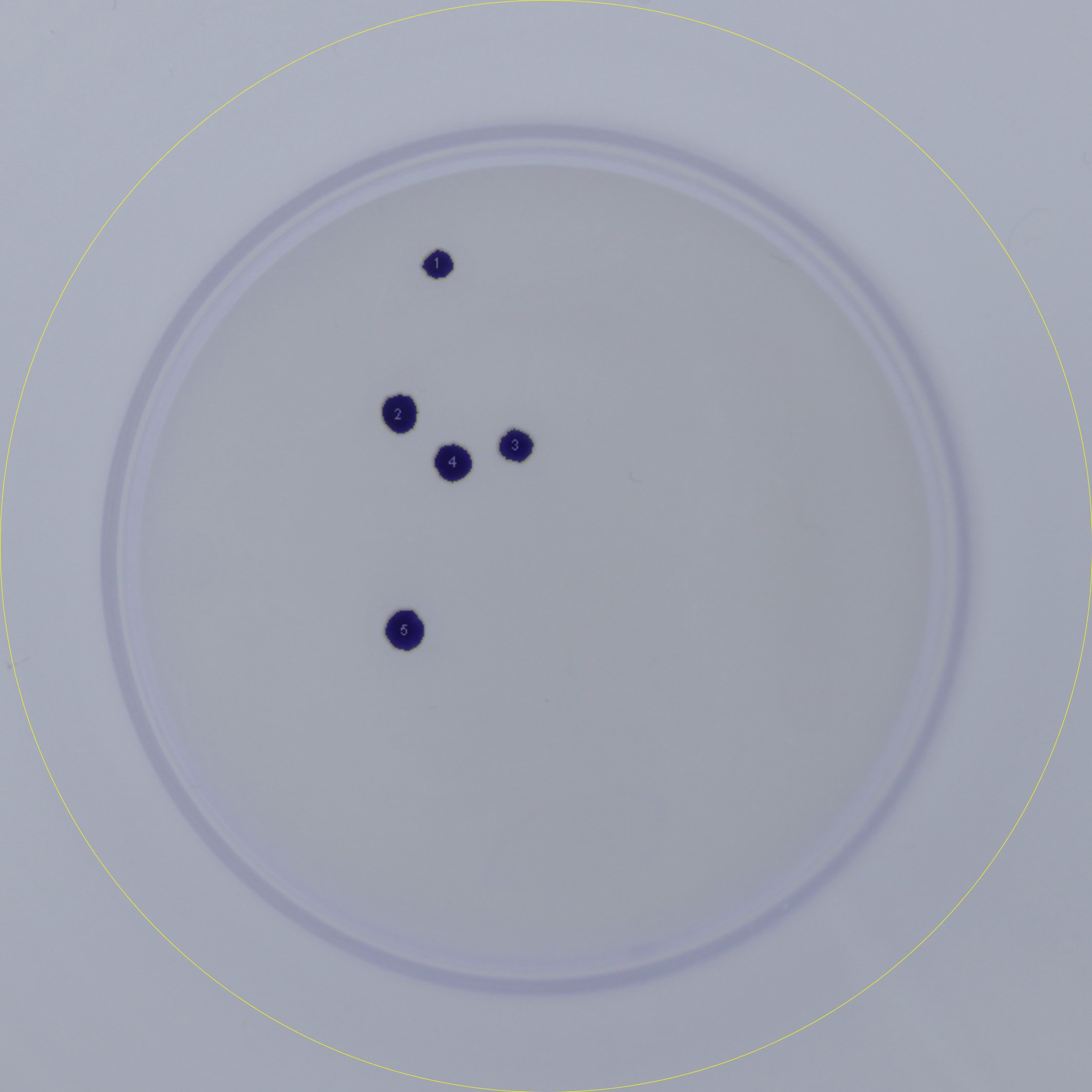

Supplement: S1 Comparison to others — (ZIP) [file pone.0205823.s007.zip › S1 Comparison to others/CAI/171214 V79 Dish/2 Results.jpg]

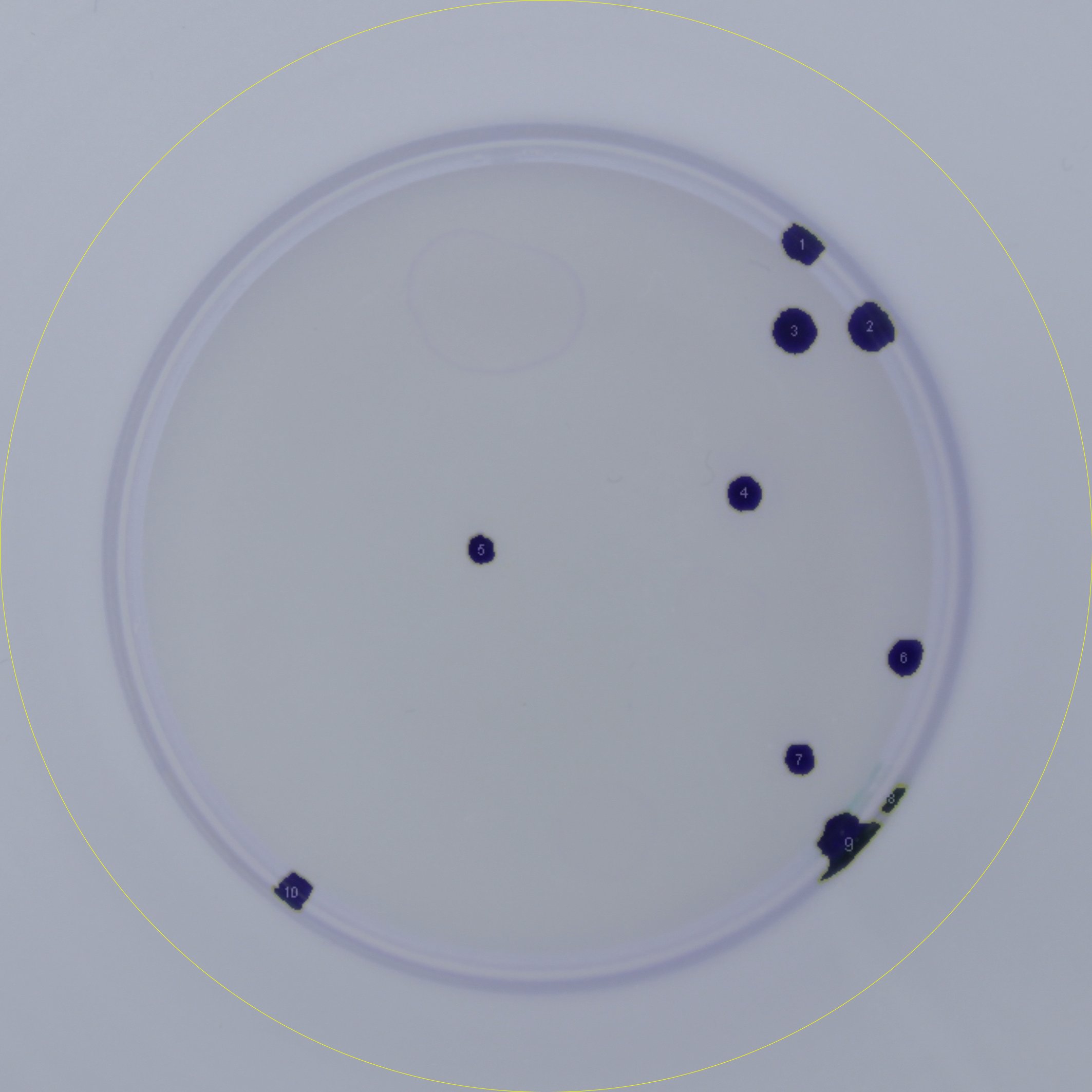

Supplement: S1 Comparison to others — (ZIP) [file pone.0205823.s007.zip › S1 Comparison to others/CAI/171214 V79 Dish/3 Results.jpg]

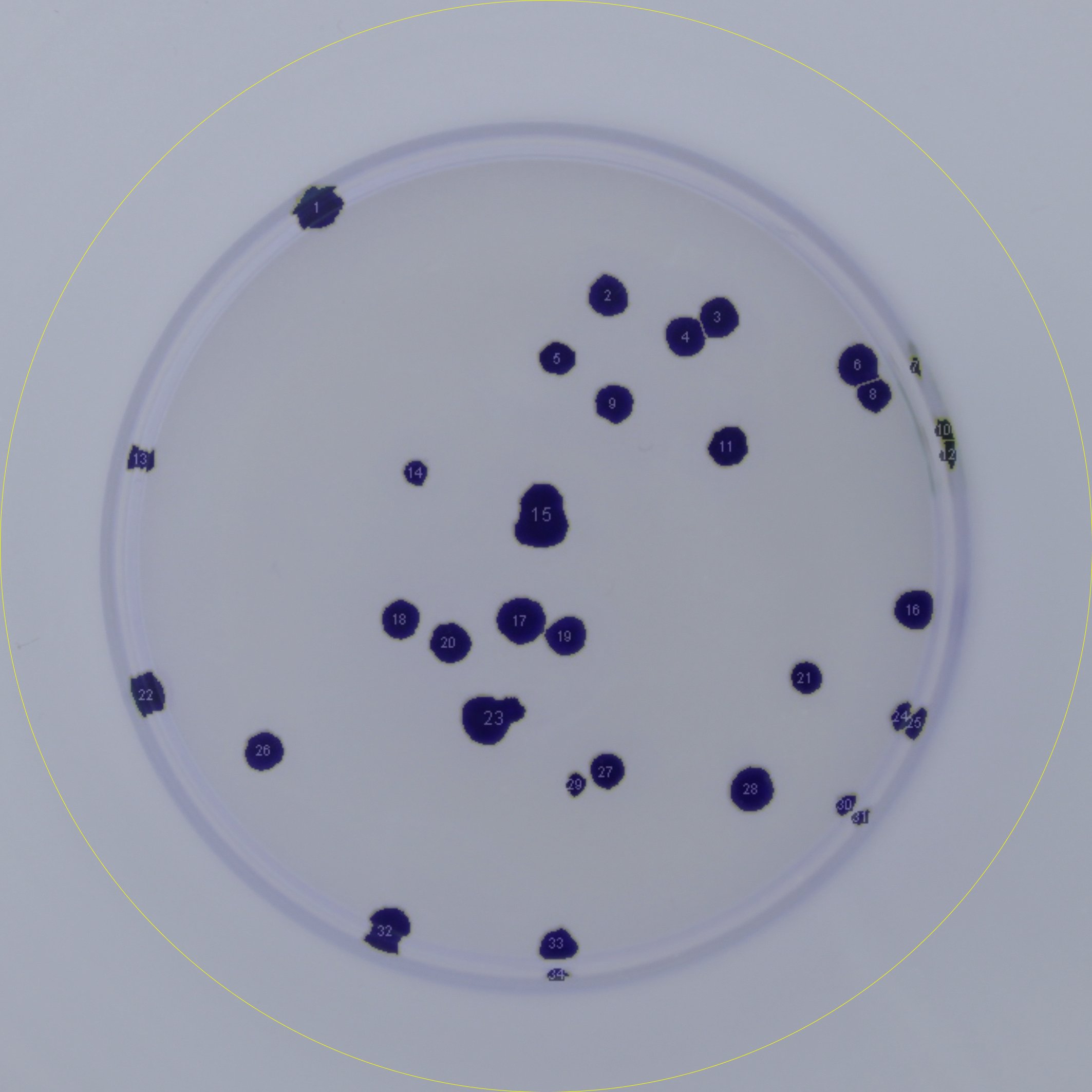

Supplement: S1 Comparison to others — (ZIP) [file pone.0205823.s007.zip › S1 Comparison to others/CAI/171214 V79 Dish/4 Results.jpg]

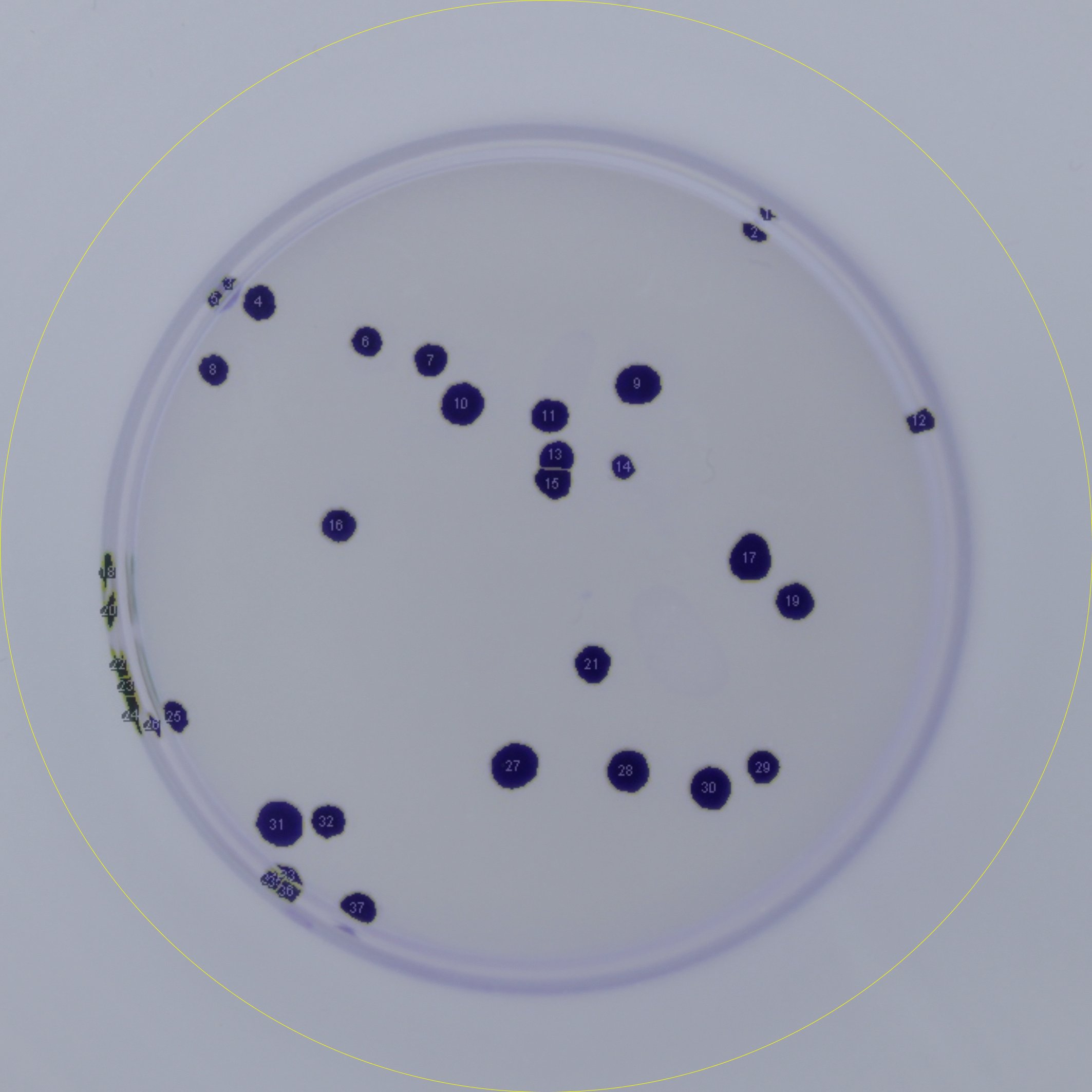

Supplement: S1 Comparison to others — (ZIP) [file pone.0205823.s007.zip › S1 Comparison to others/CAI/171214 V79 Dish/5 Results.jpg]

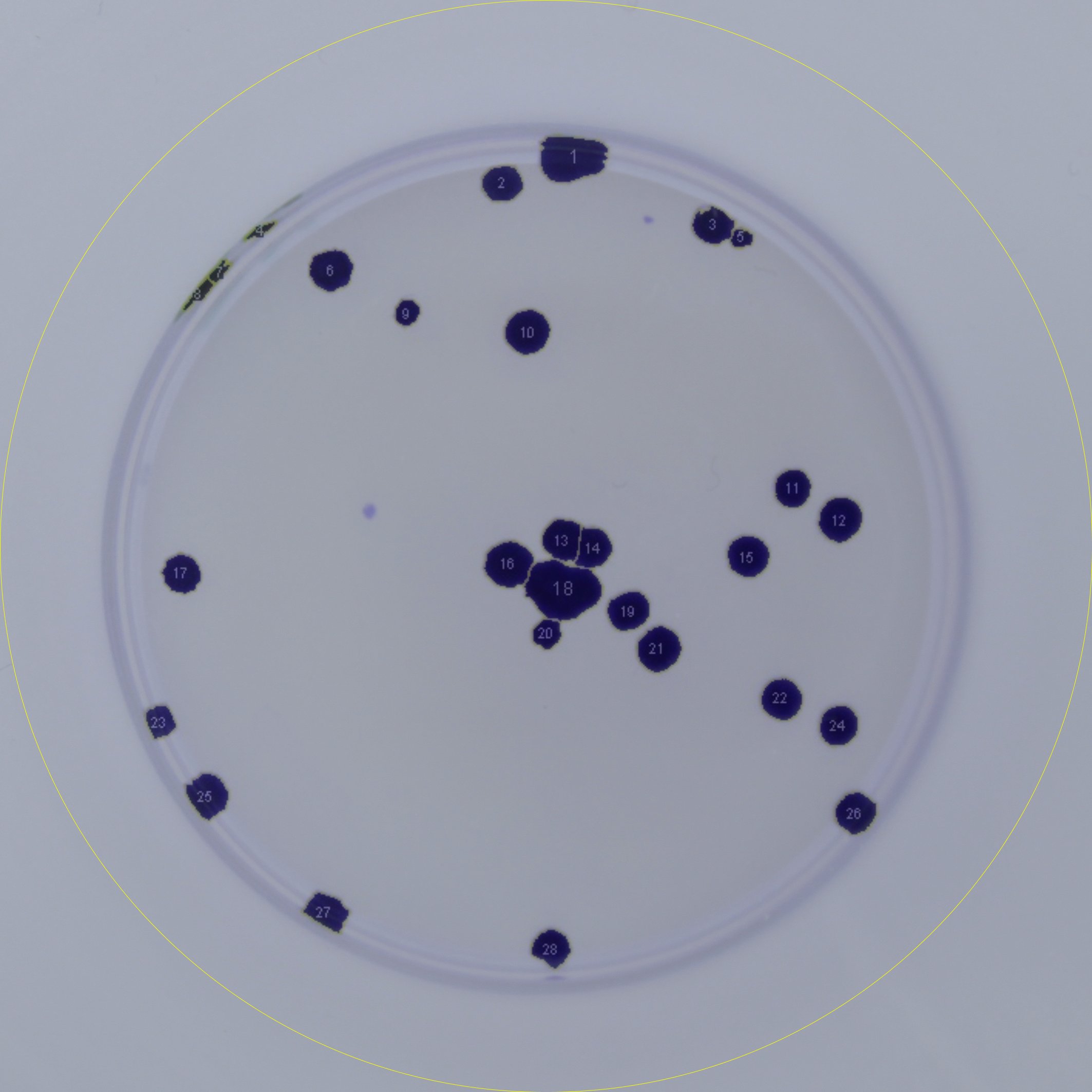

Supplement: S1 Comparison to others — (ZIP) [file pone.0205823.s007.zip › S1 Comparison to others/CAI/171214 V79 Dish/6 Results.jpg]

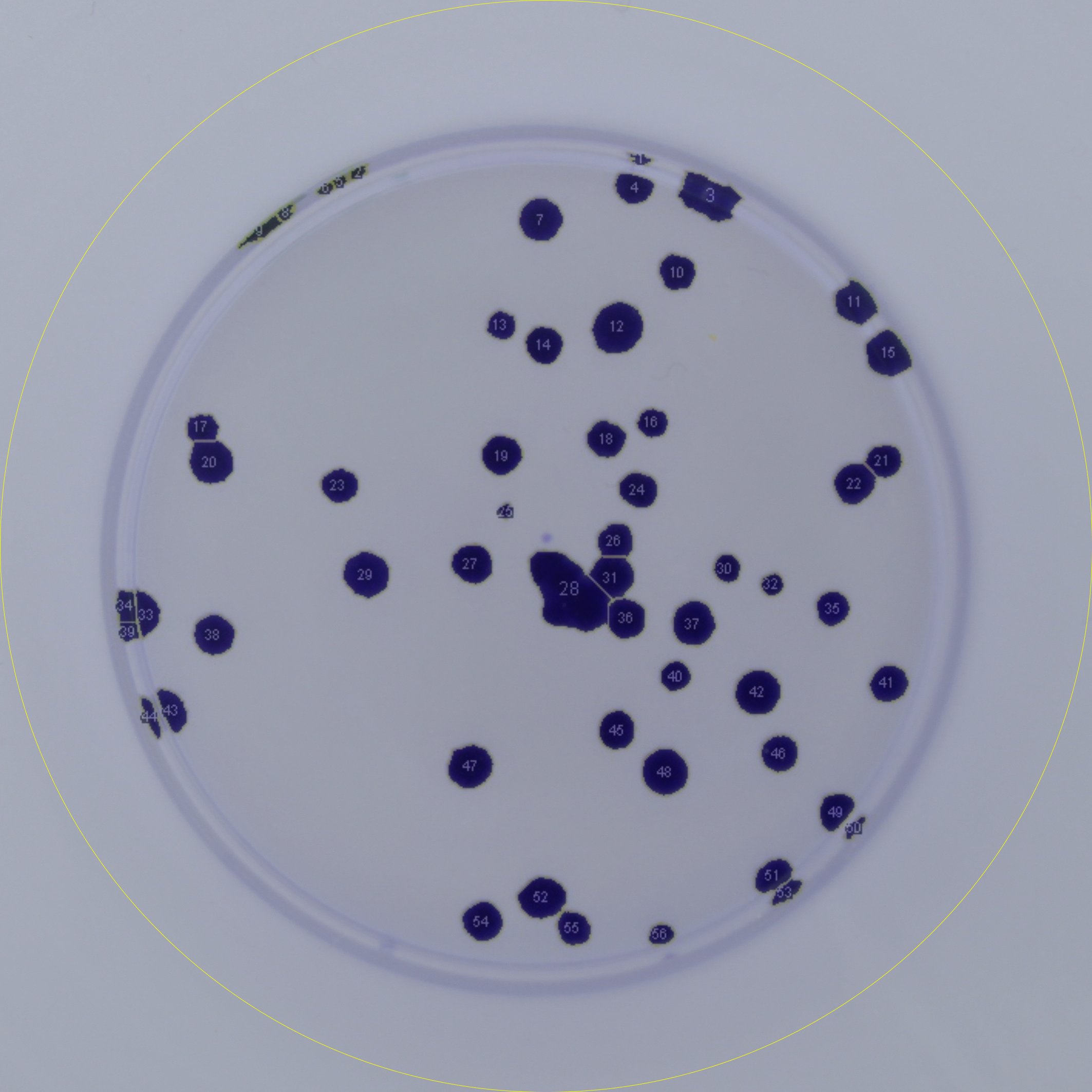

Supplement: S1 Comparison to others — (ZIP) [file pone.0205823.s007.zip › S1 Comparison to others/CAI/171214 V79 Dish/7 Results.jpg]

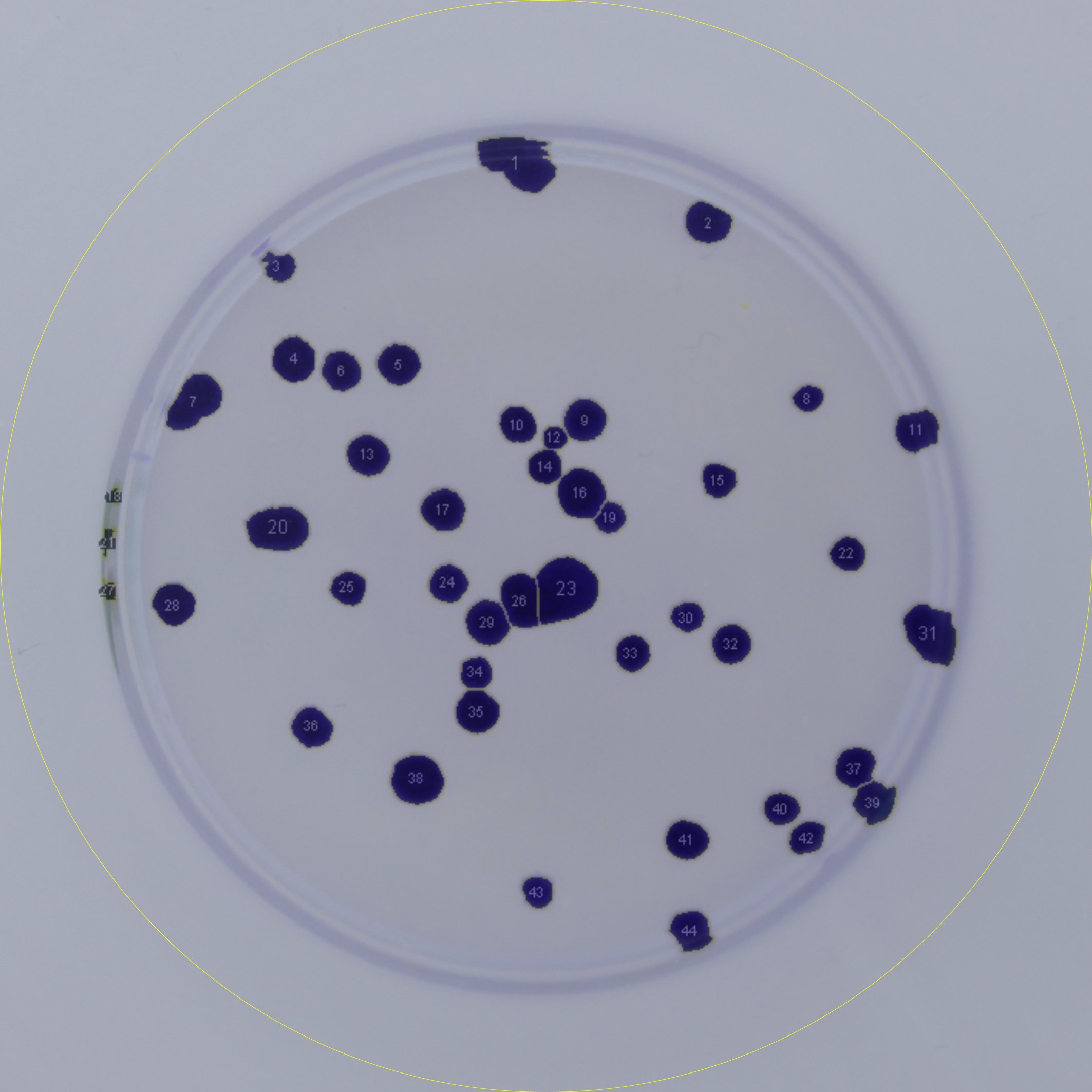

Supplement: S1 Comparison to others — (ZIP) [file pone.0205823.s007.zip › S1 Comparison to others/CAI/171214 V79 Dish/8 Results.jpg]

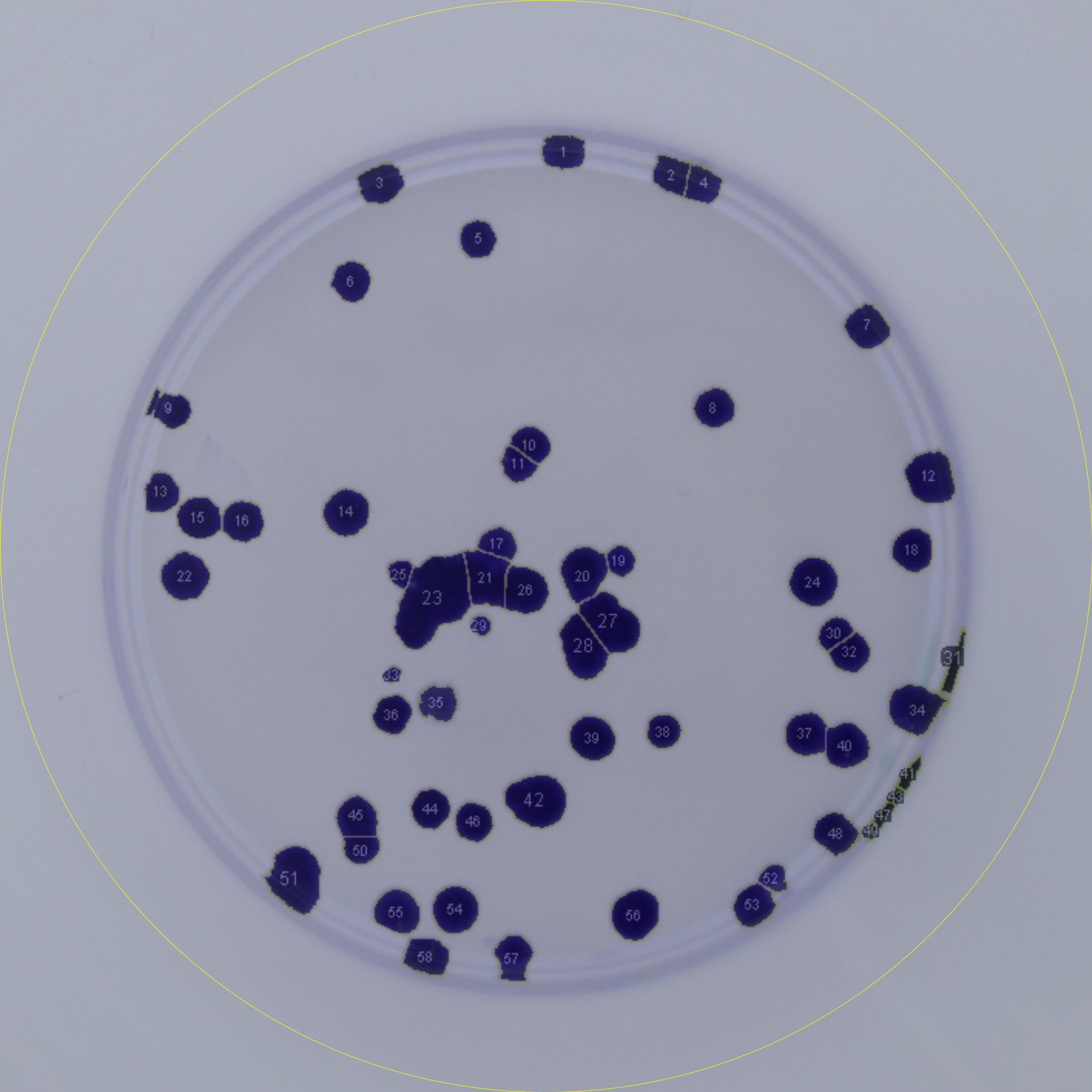

Supplement: S1 Comparison to others — (ZIP) [file pone.0205823.s007.zip › S1 Comparison to others/CAI/171214 V79 Dish/9 Results.jpg]

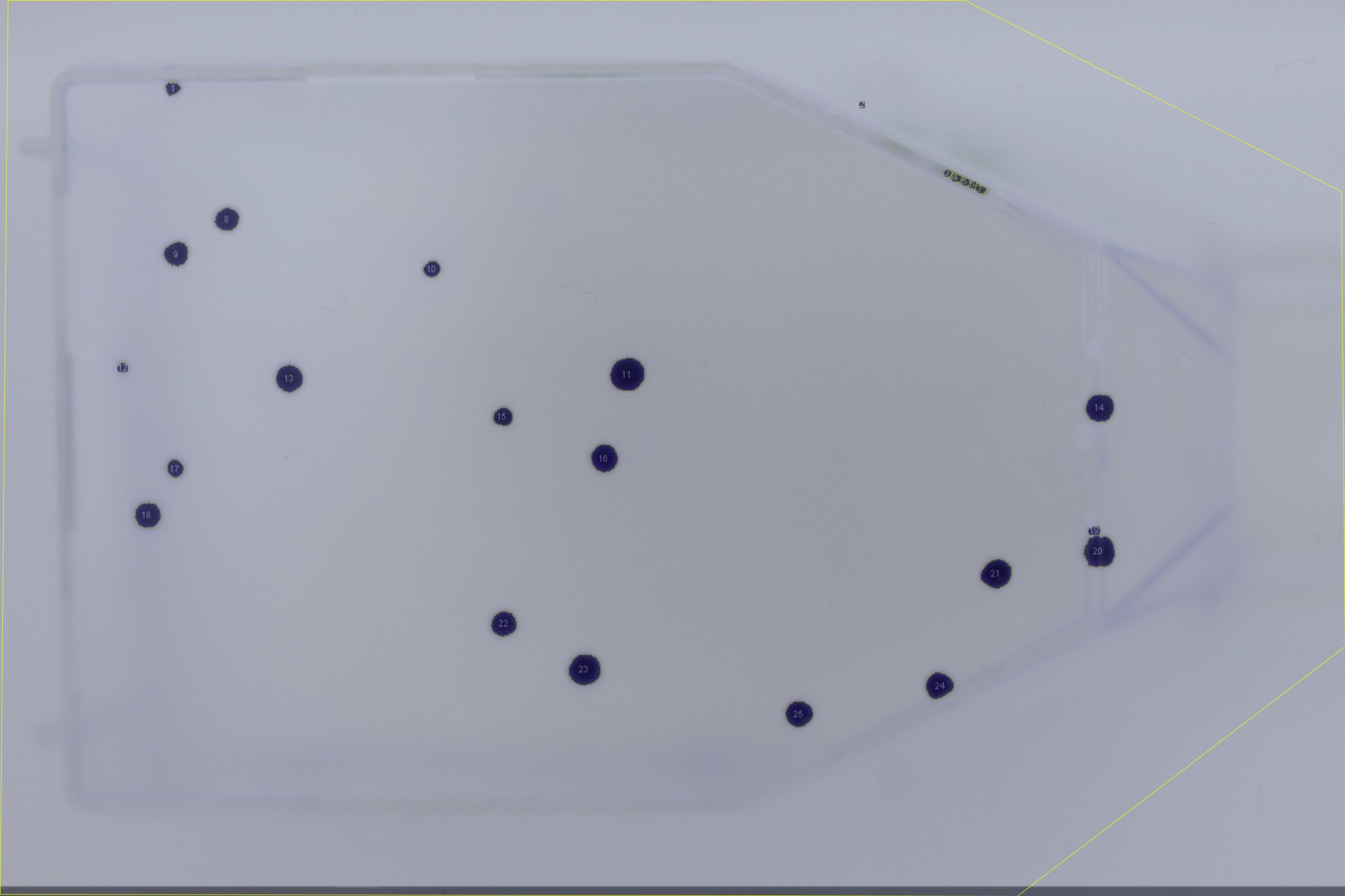

Supplement: S1 Comparison to others — (ZIP) [file pone.0205823.s007.zip › S1 Comparison to others/CAI/171214 V79 Flask/1 Results.jpg]

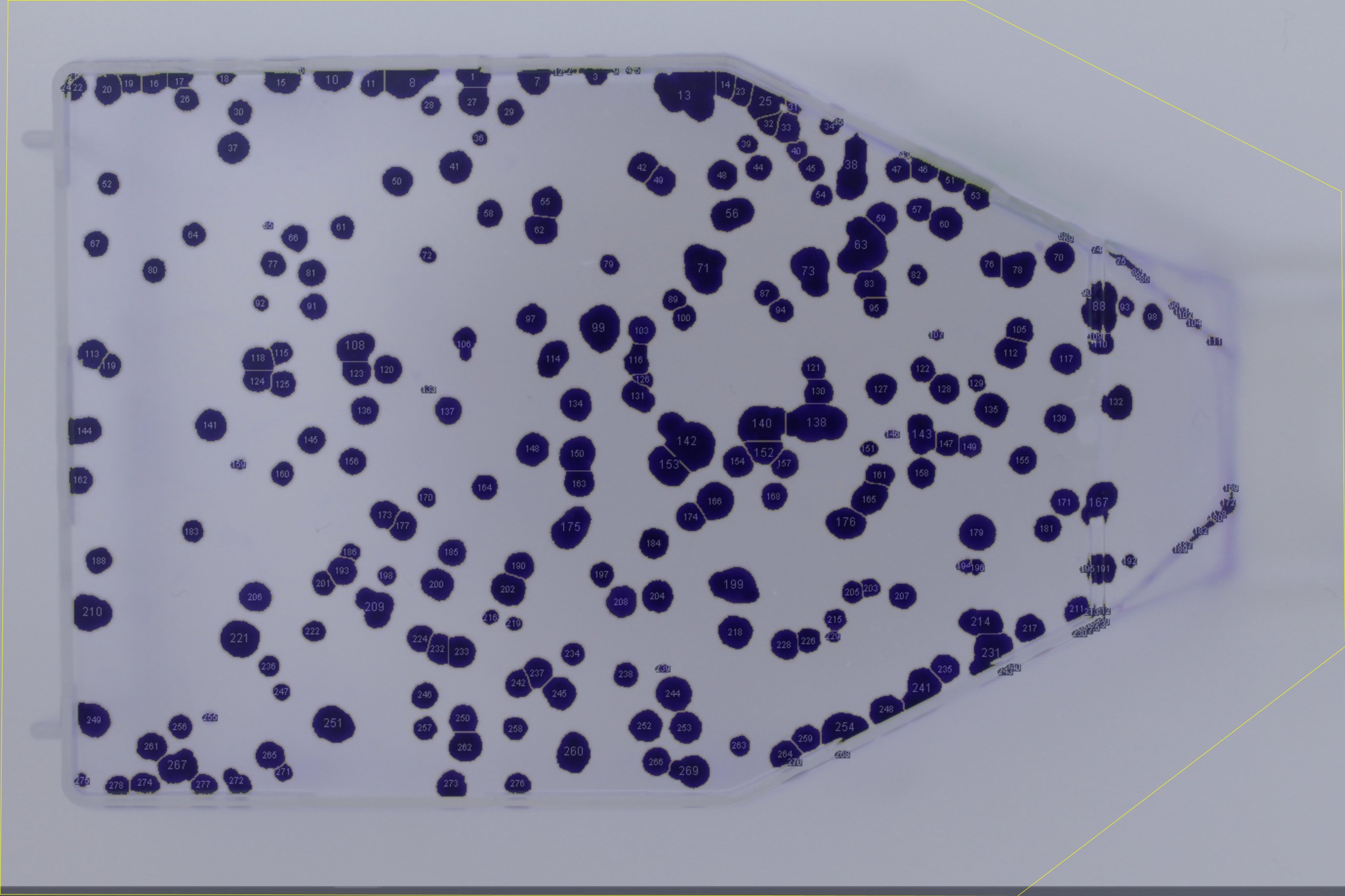

Supplement: S1 Comparison to others — (ZIP) [file pone.0205823.s007.zip › S1 Comparison to others/CAI/171214 V79 Flask/10 Results.jpg]

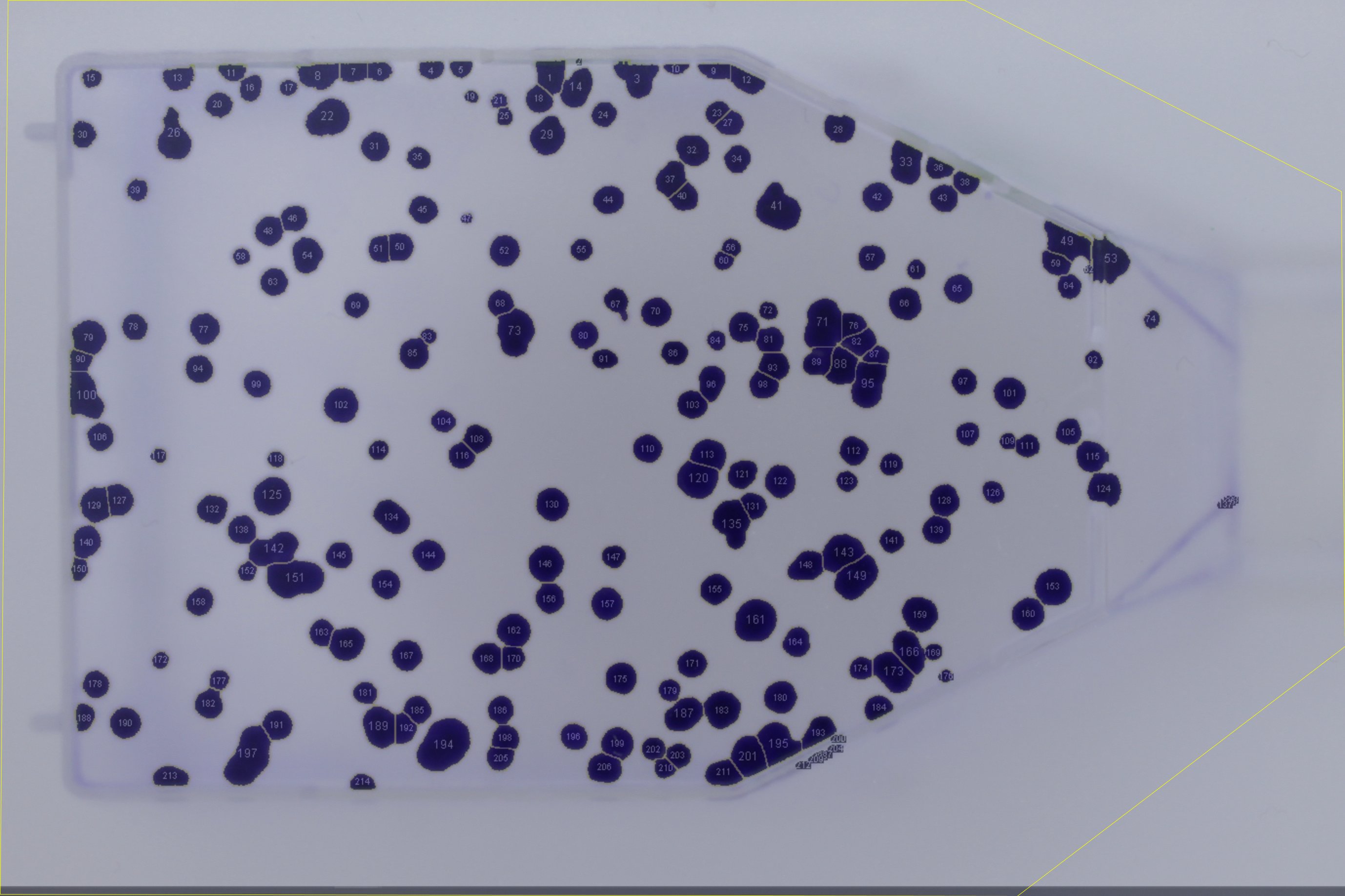

Supplement: S1 Comparison to others — (ZIP) [file pone.0205823.s007.zip › S1 Comparison to others/CAI/171214 V79 Flask/11 Results.jpg]
